# Combination decoction of Astragalus mongholicus and *Salvia miltiorrhiza* mitigates pressure-overload cardiac dysfunction by inhibiting multiple ferroptosis pathways

**DOI:** 10.3389/fphar.2024.1447546

**Published:** 2024-12-16

**Authors:** Qiyao Xu, Xuan Liu, Zhaoyang Chen, Can Guo, Pengyu Lu, Sujie Zhang, Xindong Wang, Jianping Shen

**Affiliations:** ^1^ Affiliated Hospital of Integrated Traditional Chinese and Western Medicine, Nanjing University of Chinese Medicine, Nanjing, Jiangsu, China; ^2^ Graduate School, Nanjing University of Chinese Medicine, Nanjing, Jiangsu, China

**Keywords:** ferroptosis, heart failure, Astragalus mongholicus, Salvia miltiorrhiza, GPX4, FSP1, DHODH

## Abstract

**Background:**

Astragalus mongholicus (AM) and Salvia miltiorrhiza (SM) are commonly used in traditional Chinese medicine to treat heart failure (HF). Ferroptosis has been studied as a key factor in the occurrence of HF. It remains unclear whether the combined use of AM and SM can effectively improve HF and the underlying mechanisms.

**Objective:**

This study aims to explore whether the combined use of AM and SM can improve HF by inhibiting ferroptosis. It also examines the roles and interactions of the pathways associated with GPX4, FSP1, and DHODH.

**Methods:**

*In vitro* experiments used angiotensin II-induced (4 μM for 48 h) hypertrophic H9c2 cells, while *in vivo* studies employed a rat model of transverse aortic constriction-induced (to 1 mm for 8 weeks) HF. Interventions included decoctions of AM and SM (for animal experiments) and medicated serum (for cell experiments), along with specific pathway inhibitors such as erastin, FSP1 inhibitor and brequinar. Subsequently, various molecular biology methods were used to measure the protein levels of GPX4, FSP1, and DHODH, as well as each sample group’s ferroptosis-related and HF-related indicators, to elucidate the underlying mechanisms.

**Results:**

The combined use of AM and SM can effectively restore the levels of GPX4, FSP1, and DHODH that are reduced after HF, as well as improve indicators related to ferroptosis and HF. When GPX4, FSP1, or DHODH is inhibited, the ferroptosis-inhibiting effect and the ability of AM and SM to improve HF are both weakened. When two of the three proteins are inhibited, the protective effect of HDC is strongest when GPX4 is retained, followed by FSP1, and weakest when DHODH is retained.

**Conclusion:**

This study confirms that the combined use of AM and SM inhibits ferroptosis and alleviates HF by increasing GPX4, FSP1, and DHODH levels. It shows that the protective effect is strongest through GPX4, followed by FSP1, and weakest through DHODH. These findings provide new insights into the therapeutic mechanisms of this combination of botanical drugs.

## 1 Introduction

Heart failure (HF) is a prevalent and debilitating cardiac condition, involves the pathophysiological process of diminished cardiac pumping function, leading to systemic tissue ischemia and hypoxia. While modern medicine has made significant strides in the treatment of HF, it continues to pose a serious threat to health, diminishing the quality of life and significantly reducing patients’ life expectancy. The research (Owen et al., 2023) suggests that HF can reduce the average life expectancy by 12.38 years. In a 5-year follow-up of acute HF patients, the median survival time was only 34 months (Li et al., 2021). The recurring exacerbation and worsening of the condition, resulting in repeated hospitalizations, also place a substantial burden on patients’ families and the healthcare system. The study indicates a 30-day readmission rate of 13.2% and a 1-year readmission rate of 23.3% among global HF patients (Foroutan et al., 2023). Therefore, to mitigate the global burden imposed by HF, beyond managing underlying conditions contributing to HF such as coronary artery disease and hypertension, further controlling the symptoms of established HF, improving the prognosis of HF, thereby enhancing patients’ quality of life, and reducing the readmission rate, remain viable and effective measures.

Ferroptosis is a form of programmed cell death that was identified in 2012. Distinguished from other programmed cell death mechanisms such as apoptosis and necroptosis, ferroptosis is characterized by iron-dependent lipid peroxidation (Dixon et al., 2012). Currently, the main regulatory pathways identified in lipid peroxidation during ferroptosis in the cytoplasm include the nuclear factor erythroid 2-related factor 2 (Nrf2)/cytosolic glutathione peroxidase 4 (cGPX4)/reduced glutathione (GSH) pathway, the ferroptosis suppressor protein 1 (FSP1)/coenzyme Q10 (CoQ10)/reduced nicotinamide adenine dinucleotide phosphate (NADPH) pathway, and the mitochondria-localized dihydroorotate dehydrogenase (DHODH)/CoQ10 pathway and GPX4/GSH pathway (Mao et al., 2021). Numerous animal studies on HF/myocardial injury and ferroptosis have consistently observed an increase in ferroptosis, whether induced by physical pressure overload (Wang et al., 2020) drug-induced pressure overload (Chen et al., 2023), cardiotoxic drugs (Liu J. et al., 2022), or myocardial infarction models (Hou et al., 2021). Concurrently, HF also induces alterations in ferroptosis-related regulatory pathways, primarily manifested by the downregulation of GPX4 and FSP1 (Liu B. et al., 2018; Chen et al., 2019; Liang et al., 2023; Zhang et al., 2023). Upregulating GPX4 (Zhang et al., 2023), FSP1(Zhang et al., 2023), or directly inhibiting lipid peroxidation (Zhang et al., 2022; Chen et al., 2023) can also reduce ferroptosis and inhibit the development of HF. This further confirms that ferroptosis is a crucial factor in the occurrence and progression of HF. DHODH was confirmed in 2021 to participate in the regulation of ferroptosis, serving as an inhibitor of ferroptosis (Mao et al., 2021). However, whether DHODH is involved in the occurrence and progression of HF through the regulation of ferroptosis remains unexplored.

*Astragalus mongholicus* Bunge [Fabaceae; Astragali radix] (AM) [checked with http://www.worldfloraonline.org on (2024-06-05)] and *Salvia miltiorrhiza* Bunge [Lamiaceae; Salviae miltiorrhizae radix et rhizoma] (SM) [checked with http://www.worldfloraonline.org on (2024-06-05)] are both traditional Chinese botanical drugs commonly used in the treatment of HF. AM, belonging to the legume family under the Astragalus genus (Chinese Pharmacopeia Commission, 2020) (The official website of Pharmacopoeia of the People’s Republic of China: https://ydz.chp.org.cn/#/main), is extensively utilized in traditional Chinese medicine (TCM). Its primary active metabolites include various flavonoids, saponins (such as Astragaloside IV, AS-IV), polysaccharides, among others, endowing it with diverse pharmacological activities such as immunomodulation, antioxidant, anti-inflammatory, anti-tumor, and cardiovascular protection (Zhang et al., 2021). SM belongs to the Lamiaceae family under the Salvia genus (Chinese Pharmacopeia Commission, 2020). Its major active metabolites include tanshinone and salvianolic acids (such as Salvianolic Acid B, SAB), exhibiting various pharmacological activities (Ma et al., 2022). Clinically, SM is utilized for the treatment of various ailments, particularly demonstrating significant effects in the cardiovascular system and blood circulation. It is considered to have multiple actions such as promoting blood circulation, vasodilation, lowering blood pressure, and antioxidant effects and has therapeutic effects in conditions like diabetes, liver diseases, and tumors (Ding et al., 2021). AS-IV and SAB are designated as quality control metabolites of AM and SM in the Chinese Pharmacopeia, respectively.

TCM holds that the fundamental pathogenesis of HF involves Qi and blood stasis. Treatment for HF in TCM primarily focuses on invigorating Qi and promoting blood circulation (Project Group of Traditional Chinese Medicine Guideline for Diagnosis and Treatment of Chronic Heart Failure and China Association of Chinese Medicine, 2023). Currently, among the commercial Chinese polyherbal preparation recommended for treating HF, those with the highest evidence grade and recommendation strength are Qishen Yiqi Dripping Pills and Qili Qiangxin Capsules (Project Group of Traditional Chinese Medicine Guideline for Diagnosis and Treatment of Chronic Heart Failure and China Association of Chinese Medicine, 2023). Both of these commercial Chinese polyherbal preparation contain AM (In Chinese, it is called HuangQi) for Qi invigoration and SM (In Chinese, it is called Danshen) for promoting blood circulation. It is evident that the combined use of AM and SM demonstrates significant efficacy in treating HF and is foundational in TCM treatment for this condition (Guo et al., 2022; Wei et al., 2022). Numerous studies have confirmed that AM or its active metabolites can improve myocardial hypertrophy, inhibit myocardial fibrosis, and promote blood vessel generation, thereby ameliorating HF (Liu Z. H. et al., 2018; Sui et al., 2020; Lin et al., 2021). SM, as one of the most commonly used botanical drugs in TCM for treating HF, has its active metabolites similarly confirmed by various studies to further improve ejection fraction, reduce N-terminal pro B-type natriuretic peptide (NT-proBNP) levels, and exhibit positive therapeutic effects on HF (Chen et al., 2014; Chung et al., 2018; Li et al., 2023).

In this study, we aim to simplify the complex formula to a combination of AM and SM, using a series of molecular biology techniques to explore whether this simplified combination can effectively improve cardiac function, and to investigate the therapeutic mechanisms of the AM-SM combination (HDC) in HF treatment, particularly in the context of ferroptosis. We will examine the regulatory effects of HDC on the three known pathways involved in inhibiting lipid peroxidation during ferroptosis (GPX4, FSP1, DHODH), and investigate potential interactions among these pathways during the treatment process with HDC.

## 2 Materials and methods

All manuals or guidelines mentioned in the procedures or methods can be found in Supplementary Material 1.

### 2.1 Materials and reagents

*Astragalus mongholicus* Bunge [Fabaceae; Astragali radix] (Batch No. 2301001, Wan Zhen Chinese Herbal Pieces Factory, Bozhou, China; procured via Jiangsu Province Academy of Traditional Chinese Medicine), *S. miltiorrhiza* Bunge [Lamiaceae; Salviae miltiorrhizae radix et rhizoma] (Batch No. 20230202-01, Guizhou Tongde Pharmaceutical Co., Ltd., Guizhou, China; procured via Jiangsu Province Academy of Traditional Chinese Medicine), Angiotensin II (AngII) (S25704, Yuanye, China), Ferrostatin-1 (Fer-1) (S7243, Selleck, United States), Brequinar (BQR) (HY108325, MedChemExpress), Erastin (Era) (S7242, Selleck, United States), inhibitor of ferroptosis suppressor protein 1 (iFSP1) (MFCD01572665, Macklin, China), dimethyl sulfoxide (DMSO) (D8371, Solarbio, China). Sodium Penicillin for injection (42220302, Shandong Lukang, China), anhydrous ethanol (100092683, Sinopharm Chemical Reagent Co., Ltd., China), xylene (1002341922, Sinopharm Chemical Reagent Co., Ltd., China), 0.1 M phosphate buffer (PB) (T16865, saint-Bio, China), 1% osmium tetroxide (18,456, Ted Pella Inc), ×5 loading buffer (P0015L, P0285, Beyotime, China), Quick Color Pre-Stained Gel kit 12.5% (S6172, Uelandy, China), Tris-glycine SDS-PAGE running buffer (G2027-1L, Solarbio, China), NC membrane (66,485, Bio Trace, United States), non-fat milk powder (P0216, Beyotime, China), TBST (T1085, Solarbio, China), Anti-GPX4 mAb (67763-1-Ig, Proteintech, China), Anti-FSP1 mAb (68049-1-Ig, Proteintech, China), Anti-DHODH mAb (67977-1-Ig, Proteintech, China), Anti-GAPDH Rabbit pAb (GB11002, Servicebio, China), Horseradish Peroxidase-conjugated secondary antibody (A0208, A0216, Beyotime, China), SuperSignal ECL chemiluminescence kit (P0018M, Beyotime, China), Antibody Stripping Solution (WB6200, NCM biotech, China).

### 2.2 Animal sources and routine husbandry

The animal experiments in this study were conducted in accordance with the guidelines and international standards for animal welfare issued by the Ethics Committee of the Jiangsu Province Academy of Traditional Chinese Medicine (Ethical Review Number: AEWC-20230310-272, approved on 10 March 2023). Specific Pathogen Free (SPF)-grade Sprague-Dawley (SD) rats used in this research were uniformly acquired from Nantong University [Production License Number: SCXK (Su) 2019-0001] or SiPeiFu (Beijing) Biotechnology Co., Ltd. [Production License Number: SCXK (Jing) 2019-0010], under the management of the Experimental Animal Center of the Jiangsu Province Academy of Traditional Chinese Medicine [Use License Number: SYXK (Su) 2021-0025]. These rats were free to move within their cages and had unrestricted access to food and water.

### 2.3 Cell source and routine culture

The cells used in this research were H9c2 cells, obtained from Procell (Catalog number CL-0089, China). Routine culture was carried out in a medium composed of 89% Dulbecco’s Modified Eagle’s Medium (NaHCO_3_ 1.5 g/L) (iCell-128-0001, Cellverse, China), 10% fetal bovine serum (10,270-106, Gibco, Thermo Fisher Scientific, United States), and 1% penicillin/streptomycin (C0222, Beyotime, China) in an incubator set at 37°C with 5% CO_2_.

### 2.4 Preparation of HDC decoction and HDC drug-containing serum

Both AM and SM were procured by the pharmacy department of the Jiangsu Province Academy of Traditional Chinese Medicine from manufacturers of Chinese herbal pieces, with quality control managed by the manufacturer, in compliance with the quality control standards for AM and SM specified in the *Chinese Pharmacopeia* (Chinese Pharmacopeia Commission, 2020). According to the recommended human dosages, these two botanical drugs were combined at a 2:1 weight ratio of AM to SM (Chinese Pharmacopeia Commission, 2020). To closely align with clinical medication practices, the AM-SM mixture was prepared as a decoction rather than as a freeze-dried powder. After two rounds of decoction and rotary evaporation, the final concentration of the decoction is 0.938 g/mL, equivalent to 0.625 g of AM and 0.313 g of SM per mL. The decoction at 0.938 g/mL concentration was considered a high-dose HDC (HDC-H). A suitable amount of this high-dose decoction was then diluted with an equal volume of distilled water to obtain a low-dose preparation (HDC-L) with a concentration of 0.469 g/mL. The Astragalus-Salvia decoction was analyzed using liquid chromatography-mass spectrometry to determine the content of AS-IV and SAB. The content of AS-IV was 36.86 ± 0.21 μg/mL, and the content of SAB was 7.72 ± 0.05 μg/mL.

SPF-grade SD rats were stratified by sex and then randomly assigned to three groups: control, low-dose, and high-dose. The maximum human dose for a 60 kg individual, which is 30 g/day of AM and 15 g/day of SM (Chinese Pharmacopeia Commission, 2020), was used as the high dose. Accordingly, 15.00 g/day of AM and 7.50 g/day of SM constituted the low dose. Based on the dosage conversion methods for experimental animals in “*Experimental Methodology of Pharmacology (4th edition)*” (Wei et al., 2020) the drug dosage per kg body weight for SD rats was determined to be 6.25 times that of the human dosage. As a result, the daily required dose for the SD rats in the high-dose group was HDC-H 5 mL/kg, and for the low-dose group, it was HDC-L 5 mL/kg. The daily dose was administered by gavage twice per day for 7 consecutive days. After the dosing period, rats were anesthetized with isoflurane, and blood was collected from the abdominal aorta. After centrifugation, the serum was collected and pooled within the same experimental group, resulting in three types of serum: control rat serum, low-dose HDC-containing serum (HDCL-S), and high-dose HDC-containing serum (HDCH-S).

### 2.5 Metabolite identification of HDC and molecular docking of the main active metabolites with target proteins

The obtained serum and the HDC decoction were both subjected to liquid chromatography-mass spectrometry for metabolite identification. Liquid chromatography-mass spectrometry data acquisition method referenced previous literature (Yuan et al., 2023), with some methods or parameters slightly modified. Detection was performed using an Orbitrap Exploris 120 mass spectrometer (Thermo Fisher Scientific). Detailed parameters are as follows: Sheath gas flow rate: 35 Arb, Aux gas flow rate: 15 Arb, Ion Transfer Tube Temp: 350°C, Vaporizer Temp: 350°C, Full ms resolution: 60,000, MS/MS resolution: 15,000, Collision energy: 16/32/48 in NCE mode, Spray Voltage: 5.5 kV (positive) or −4 kV (negative). Additionally, multiple reaction monitoring (MRM) was used to detect AS-IV and SAB in the Astragalus-Salvia decoction. Carbamazepine and salidroside, both at concentrations of 1 μg/mL, were used as internal standards for positive and negative ion modes, respectively. AS-IV standard (HY-N0431R, MCE, United States) was used at a concentration of 1,088 μg/mL, and SAB standard (HY-N1362R, MCE, United States) was used at a concentration of 1,136 μg/mL. For AS-IV detection, the conditions were as follows: parent ion mass-to-charge ratio (m/z) of 802.6, daughter ion m/z of 785.1, declustering potential (DP) of 136.1 V, collision energy (CE) of 11.32 eV, and collision cell exit potential (CXP) of 35.11 V. The second condition for AS-IV was parent ion m/z 802.4, daughter ion m/z 785.4, DP 135.93 V, CE 10.63 eV, and CXP 30.92 V. The third condition was parent ion m/z 807.5, daughter ion m/z 627.5, DP 249.78 V, CE 61.76 eV, and CXP 26.94 V. For SAB detection, the first condition was parent ion m/z 717.5, daughter ion m/z 519.3, DP -175.7 V, CE -25.29 eV, and CXP -19.93 V. The second condition was parent ion m/z 717.1, daughter ion m/z 519.4, DP -152.13 V, CE -27.3 eV, and CXP -20.38 V. The third condition was parent ion m/z 717.3, daughter ion m/z 519.6, DP -160.3 V, CE -30 eV, and CXP -21.3 V. This complies with the ConPhyMP requirements (Heinrich M, et al., 2022).

Select the main metabolites of AM (AS-IV, CAS: 84,687-43-4) and SM (SAB, CAS: 121,521-90-2) for molecular docking with target proteins FSP1 (PDB ID: 8JSC), DHODH (PDB ID: 5K9D), and GPX4 (PDB ID: 6ELW). First, convert the 2D structures of AS-IV and SAB obtained from PubChem into 3D structures using Open Babel (version 3.1.0, Open Babel Development Team). Then, preprocess the protein structures using AutoDockTools (version 1.5.7, Scripps Research), including removing water molecules, adding polar hydrogen atoms, and assigning Gasteiger charges. In AutoDockTools, select the entire protein as the grid region and set appropriate grid sizes to cover the whole protein. Perform molecular docking using AutoDock Vina (version 1.2.5, Scripps Research) with 10 iterations and an exhaustiveness of 1.0, outputting the 9 lowest binding energy modes. Finally, analyze the docking results to select the conformation with the lowest binding energy as the optimal binding mode, and visualize the binding mode using PyMOL (version 2.6.0 Open-Source, Schrodinger LLC) to evaluate the interactions between the metabolites and proteins.

### 2.6 Cell intervention methods

Twenty-four hours after cell passage, cells were cultured for 24–72 h with AngII at different concentrations (0μM, 1μM, 2μM, 4μM, 8μM, 12μM, and 16 μM). A combination of AngII concentration and incubation time that caused significant cell enlargement without notable cytotoxicity (4 μM for 48 h, Supplementary Figures 1A–C) was chosen for subsequent modeling of a HF cell model. After model establishment, drug interventions were performed. The serum in the intervention culture medium was replaced with an equal volume of the corresponding rat serum (blank serum, HDCL-S, or HDCH-S), each constituting 10% of the culture medium volume. The final concentrations of other intervention substances in the culture medium were as follows: AngII at 4 μM, Fer-1 at 1 μM, Era at 5 μM, iFSP1 at 1 μM, and BQR at 10 μM. The concentrations of the aforementioned agents were determined based on similar dosages used in previous research studies (Dixon et al., 2012; Sykes et al., 2016; Horwath et al., 2017; Cheu et al., 2023; Nakamura et al., 2023). All substances were dissolved in DMSO, with the maximum concentration in the culture medium being 0.1%. An appropriate amount of DMSO was added to each of the other culture media to standardize the DMSO concentration to 0.1% across all groups, ensuring comparability. Detailed medium preparation methods are provided in the Supplementary Table 1.

### 2.7 Animal model and intervention method

After 1 week of acclimatization, 78 SPF-grade mature male SD rats (180–200 g) were randomly assigned into two groups using a random number method: the sham operation group (n = 6) and the transverse aortic constriction (TAC) model group (n = 72). The TAC model group underwent TAC surgery as previously described (Garrott et al., 2017; Carley et al., 2021). In this study, the aortic arch was transversely constricted to 1.0 mm. The sham operation group underwent the same procedures without the final step of transverse aortic arch ligation. All rats were administered an intramuscular injection of 100,000 units of penicillin for anti-infection on the second and third days post-operation.

After 2 months of standard rearing, left ventricular ejection fraction (LVEF) and serum (from the tail vein) NT-proBNP was assessed in all rats. The sham-operated rats formed one group, while the TAC model rats were stratified based on LVEF and then randomly grouped using a random number method, aiming to ensure comparability between groups as much as possible. Except for HDC-L and HDC-H, which were administered via gavage at a volume of 5 mL/kg as described in Section 2.4 of the methods, other intervention agents were administered via intraperitoneal injection. The injection concentrations and dosages of each agent were as follows: Fer-1 (0.21875 mM, 0.875 μmol/kg), Era (2.1875 mM, 8.75 μmol/kg), BQR (2.1875 mM, 8.75 μmol/kg), and iFSP1 (0.21875 mM, 0.875 μmol/kg). The dosages used for the aforementioned agents were based on the amounts referenced from previous studies (Sykes et al., 2016; Li et al., 2022; Cheon et al., 2023; Zhou et al., 2023). Based on the dosages, the aforementioned preparation concentrations were adopted to ensure that each agent was administered at a volume of 4 mL/kg. The TAC + HDC-H + Era + iFSP1+BQR group required injection of all three formulations, thus having the highest total injection volume at 12 mL/kg. Therefore, for the other groups, the injection volume was supplemented with solvent to reach 12 mL/kg. Intraperitoneal injections were given every other day for 4 weeks. The Sham group receiving pure water by gavage according to body weight for 4 weeks. The detailed preparation methods of reagents are provided in Supplementary Table 2.

### 2.8 Cell size determination

Following the completion of H9c2 cell culture, crystal violet staining was performed using the crystal violet staining kit (C0121, Beyotime, China) according to the manufacturer’s instructions. Subsequently, under an inverted microscope, 6 fields of view at ×100 and ×400 magnification were randomly selected for photography. The images at ×400 magnification were used for direct visual observation, while the images at ×100 magnification were used for calculating cell size. The ImageJ 1.54f software (Wayne Rasband, National Institutes of Health, United States, https://imagej.nih.gov/ij/) was utilized to select all purple regions as the total cell area. Additionally, deeply stained purple cell nuclei were selected for nucleus counting, representing the cell count. The average cell area within each field was determined by dividing the total area by the cell count.

### 2.9 Cell viability and vitality determination

The cell viability and vitality of each group relative to the control group were measured using the widely used cell counting kit-8 (C0042, Beyotime, China). The specific procedures were carried out according to the manual provided with the kit.

### 2.10 Enzyme-linked immunosorbent assay (Elisa) detection of substance levels

The levels of NT-proBNP in cells and rat serum were measured using a rat NT-proBNP Elisa kit (DreamBio, China). CoQ10H_2_ levels were determined using a rat CoQ10H_2_ Elisa kit (DreamBio, China). The specific procedures were carried out according to the manual provided with the kit.

### 2.11 Determination of GSH, MDA, and Fe^2+^ content

The contents of GSH, MDA, and Fe^2+^ were measured using commercial assay kits. Specifically, the GSH and GSSG assay kit (S0053, Beyotime, China), lipid peroxidation (MDA) assay kit (S0131S, Beyotime, China), tissue Fe^2+^ colorimetric assay kit (E-BC-K773-M, Elabscience, China), and cellular Fe^2+^ colorimetric assay kit (E-BC-K881-M, Elabscience, China) were employed. The specific operations were conducted according to the instructions provided in the manuals. It should be noted that for MDA measurement, the manual recommended mixing the sample with reagent and boiling it sealed for 15 min. However, preliminary experiments indicated that boiling for 15 min was ineffective; therefore, the boiling duration was extended to 1 h, which was also adopted for subsequent measurements.

### 2.12 Determination of LVEF in rats

After anesthetization with isoflurane, rats were placed in a supine position on the ultrasound examination table. The chest area was prepared by shaving and applying coupling gel. Echocardiography was performed by the same operator using a high-resolution ultrasound system (Vevo 3,100, VisualSonics, Canada) equipped with a specialized small animal ultrasound probe (21 MHz). Measurements were taken from both the short-axis and parasternal long-axis, capturing at least three consecutive cardiac cycles. The ventricular wall thickness and internal diameter during systole and diastole were manually selected, following which the ejection fraction was automatically calculated by the ultrasound machine.

### 2.13 Masson’s trichrome staining

For tissue preparation, The ventricle was divided into three sections along the longitudinal axis of the heart, namely, upper, middle, and lower. Approximately 2 mm thick myocardial tissue was obtained from the middle section. This tissue sample was then fixed in 4% paraformaldehyde at ten times its volume. The fixed tissue was embedded in paraffin wax, and sections of 5 μm thickness were prepared using a microtome. masson’s trichrome staining was performed using a masson’s trichrome stain kit (G1340, Solarbio, China). Additionally, anhydrous ethanol, xylene, a constant temperature oven, glycerol gelatin sealing tablets, and cover slips were prepared. Following the instructions provided in the masson’s trichrome stain kit. The prepared slides were observed under a upright optical microscope (E100, Nikon, Japan) for pathological analysis and imaging. The left ventricular section was divided into four parts (upper, lower, left, right), and one image (×20) was randomly selected from each part for fibrosis area quantification. Quantitative analysis of the fibrosis area was performed using ImageJ software (version 1.53t, National Institutes of Health, United States), and the average value of these four measurements was used to represent the fibrosis level for that sample. The same procedure was applied to all samples within the same group, and the average of these individual sample values was calculated to represent the fibrosis level for the entire group. This quantitative fibrosis data was then used to select representative images that accurately reflect the fibrosis extent, allowing for more intuitive visualization of fibrosis severity.

### 2.14 Detection of mitochondrial membrane potential in cells

Mitochondrial membrane potential was assessed using JC-1 staining and flow cytometry analysis. Post-cultivation, H9c2 cells were harvested into tubes, and a pre-prepared JC-1 staining solution (C2006, Beyotime, China) was added to each tube. The cells were then incubated at 37°C for 30 min. This was followed by centrifugation and resuspension of the cells in phosphate buffered saline (PBS). After a second round of centrifugation to pellet the cells, the precipitate was resuspended in 1 mL of PBS to reconstitute a single-cell suspension. Cell concentration was adjusted to approximately 1*10^6 cells/mL based on cell counting and the addition of varying volumes of PBS. Analysis was performed using a CytoFLEX S flow cytometer (Beckman Coulter, United States). The fluorescein isothiocyanate (FITC) channel detected green fluorescence of JC-1 monomers, while the phycoerythrin (PE) channel detected red fluorescence of JC-1 aggregates. The degree of reduction in mitochondrial membrane potential was quantified using the formula: Green Fluorescence/(Red Fluorescence + Green Fluorescence).

### 2.15 Transmission electron microscopy (TEM) analysis

Transmission electron microscopy is used to examine the morphological structure of rat cardiac mitochondria. In brief, Myocardial tissue samples (approximately 2–3 mm in length, 1 mm in diameter) were excised from rat hearts’ apical region and fixed in electron microscopy fixative (G1102, Servicebio, China) for 24 h. After fixation, tissues were rinsed thrice with 0.1 M PB for 15 min each. Next, samples were incubated in 1% osmium tetroxide in 0.1 M PB for 2 h, followed by three rinses in 0.1 M PB. Dehydration was done in graded ethanol series (30%-100%) and two immersions in 100% acetone. Infiltration was performed with acetone and EPON812 epoxy resin, followed by embedding in EPON812 epoxy resin. Ultra-thin sections (80 nm) were prepared using an ultramicrotome (PT-PC, RMC, United States) and stained with uranyl acetate and lead citrate. The mitochondrial ultrastructure in myocardial tissue was observed under a transmission electron microscope (HT7800, HITACHI, Japan).

### 2.16 Western blotting (WB)

After cultivation, H9c2 cells were lysed using lysis buffer. The supernatant, representing the cellular protein, was collected post-centrifugation. Total protein concentration was determined using a BCA assay. After quantification, 1/4 volume of ×5 loading buffer was added to the remaining protein samples, followed by boiling for 5 min to prepare for WB loading. Unused samples were stored at −80°C for future use.

Cardiac muscle tissue was harvested from the same region of each rat heart, homogenized, lysed, and then centrifuged to collect the supernatant, following the same procedure as for cell samples.

The WB process was conducted according to the Bio-Rad company protein blotting guide (Supplementary Material 2), with specific reagents and parameters as follows: The gel concentration was 5% stacking gel +12.5% separating gel. The electrophoresis conditions are as follows: constant voltage of 80 V for the stacking gel and constant voltage of 120 V for the resolving gel. The semi-dry transfer conditions are as follows: GPX4 transferred at 20 V for 10 min, while GAPDH, FSP1, and DHODH transferred at 25 V for 15 min. Antibodies (GPX4 diluted 1:1,000, FSP1 1:5,000, DHODH 1:2000, GAPDH 1:2000) were incubated overnight at 4°C. The horseradish peroxidase-conjugated secondary antibodies were diluted 1,000 times. For DHODH and FSP1, imaging of one protein was done first, followed by antibody stripping on the NC membrane, re-blocking, incubation with the other antibody, and re-imaging. When performing whole-membrane development, it is also necessary to develop one protein at a time, followed by antibody stripping. Then, the membrane is re-blocked and incubated with another antibody, developed again, and this process is repeated until all target proteins on the membrane have been developed.

### 2.17 Statistical analysis

In this study, data are reported as the mean ± SD (standard deviation) of multiple sample data, with each experiment being repeated three times to ensure reproducibility. Statistical analysis was conducted using GraphPad Prism 9.0 (GraphPad Software, Inc., United States) and SPSS 26.0 software (IBM, United States). For comparisons between two independent groups, an unpaired two-tailed Student’s t-test was used. When assessing differences among multiple groups, one-way analysis of variance (ANOVA) was utilized. A *p*-value of less than 0.05 was considered statistically significant.

## 3 Results

### 3.1 HDC metabolite identification and molecular docking

**FIGURE 1:**
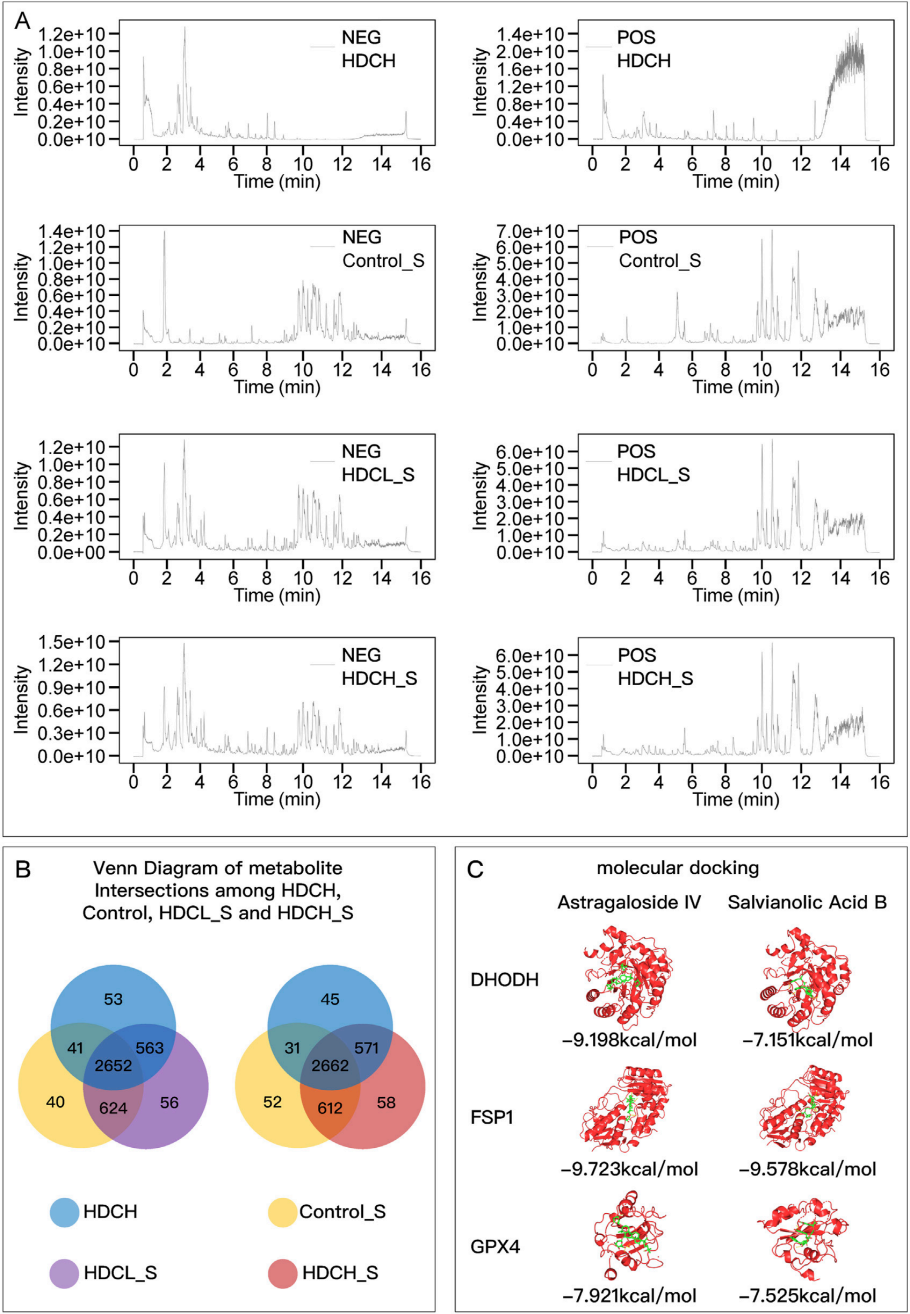
HDC metabolite identification and molecular docking. **(A)** Chromatograms of HDCH, Control_S, HDCH_S, and HDCL_S under NEG and POS ion modes; **(B)** Venn diagram of metabolites in different samples. **(C)** Molecular Docking Results of Astragaloside IV and Salvianolic Acid B with DHODH, FSP1, and GPX4. HDCH, High Dosage Astragalus mongholicus and Salvia miltiorrhiza Decoction; Control_S, Normal Rat Serum; HDCH_S, High Dosage Astragalus mongholicus and Salvia miltiorrhiza Medication-Containing Serum; HDCL_S, Low Dosage Astragalus mongholicus and Salvia miltiorrhiza Medication-Containing Serum; NEG, Negative Ion Mode; POS, Positive Ion Mode.

The main metabolites of the HDC decoction and the drug-containing serum were detected using liquid chromatography-mass spectrometry (Figure 1A; Supplementary Table 3). These primarily include various flavonoids, isoflavones, terpenes, alkaloids, saponins, phenols, amino acids, steroids, and their derivatives. Notably, this includes the main metabolites of AM, such as Astragalus polysaccharides, Astragaloside I-IV, and Cycloastragenol, as well as the key metabolites of SM, including Salvianolic acid A, B, C, Tanshinone I, and Tanshinone IIA. The Venn diagram (Figure 1B) indicates that 3,215 metabolites found in the low-dose drug-containing serum were also present in the decoction. Of these, 563 metabolites not found in the control group’s serum suggest direct entry into the blood from the HDC decoction, including major active metabolites of AM and SM. Additionally, 56 metabolites, not present in both the decoction and the control serum, might be metabolites produced in the body from HDC. In the high-dose drug-containing serum, 3,233 metabolites were identified as same as those in the decoction, with 571 metabolites directly entering the blood from the decoction, including major active metabolites of AM and SM, and another 58 metabolites possibly being metabolites produced in the body from HDC.

Using MRM under different parameter settings, chromatographic fingerprinting analysis was performed for AS-IV and SAB in both standard samples and the HDC-H. Three different detection conditions were applied, ensuring a complete and comprehensive characterization of the main active metabolites in the decoction from multiple perspectives. The MRM chromatograms for both the standard samples and the decoction samples are shown in Supplementary Figure 2. The results clearly demonstrate the separation and quantification of AS-IV and SAB, effectively illustrating the content characteristics of these two key metabolites in the decoction, ensuring a thorough evaluation of the chemical composition of the preparation.

Figure 1C shows the lowest binding energy modes of AS-IV and SAB when docked with DHODH, FSP1, and GPX4, respectively. The binding energies were all less than −7 kcal/mol, indicating that the active metabolites of AM and SM can stably bind to the key regulatory proteins of ferroptosis.

### 3.2 Optimal concentration and induction time of AngII in H9c2 cells

The relative viability of H9c2 cells induced by different concentrations of AngII for varying durations was assessed using the cell counting kit-8, and changes in cell size were observed. There was no significant toxicity at any concentration after 24–48 h of culture, but apparent cell death was observed after 72 h (Supplementary Figure 1A). Concentrations of 4μM, 8μM, and 12 μM AngII caused hypertrophy in H9c2 cells within 24–72 h of culture, while 2 μM required at least 48 h, and 1 μM required at least 72 h (Supplementary Figures 1B, C). Ultimately, this study selected 4 μM AngII concentration with 48 h of induction as the optimal conditions for subsequent experiments.

### 3.3 Reduction in cardiac function and subsequent grouping in TAC model rats

Eight weeks post-TAC surgery in SD rats, 55 rats survived (6 in sham group and 49 in TAC model group). Standard M-mode echocardiography was used for assessment, revealing significantly reduced left ventricular contractility in the TAC model group compared to the Sham group (Supplementary Figure 1D). Measurement of LVEF further confirmed a significant reduction in cardiac function in SD rats 8 weeks post-TAC surgery (Supplementary Figure 1E). After modeling, the rats were stratified and randomized into groups based on LVEF. Comparison of LVEF and NT-proBNP among groups revealed that the LVEF in the Sham group was significantly higher than in any other group, and the NT-proBNP was significantly lower, while there was no inter-group difference in LVEF and NT-proBNP in all TAC model groups (Supplementary Figure 1F), indicating a consistent level of HF across groups.

### 3.4 HDC effectively inhibits increased ferroptosis associated with worsening HF

**FIGURE 2:**
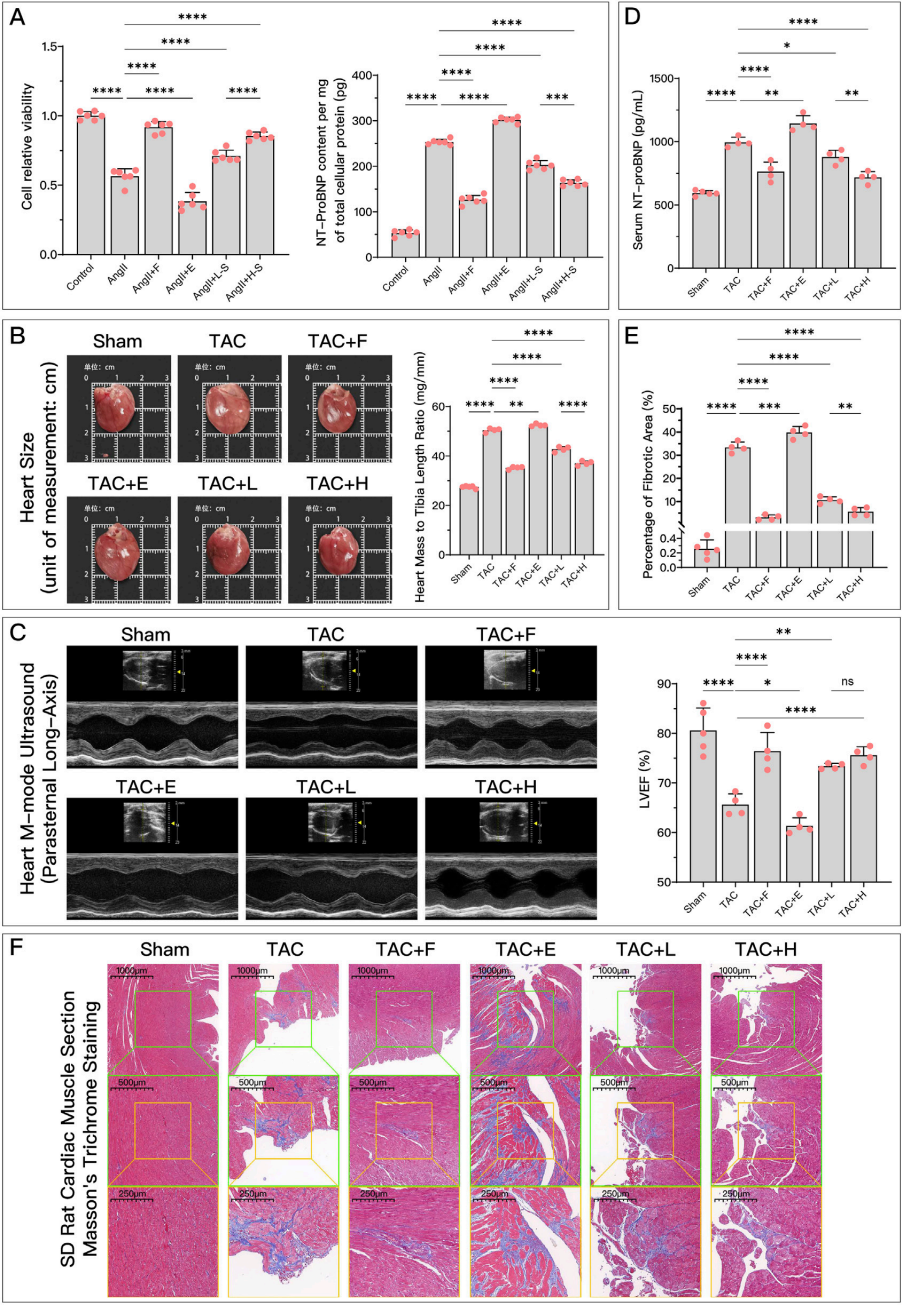
HDC effectively improves AngII-induced cell dysfunction and HF due to TAC. **(A)** Relative cell viability of different groups of H9c2 cells compared to the control group, and NT-proBNP content per mg of total cellular protein; **(B)** Gross images of rat hearts from each group for direct comparison of heart size, and comparison of heart weight to tibia length ratio in each group; **(C)** Parasternal long-axis M-mode echocardiographic images of the left ventricle in rats from each group, and quantitative comparison of LVEF; **(D)** Comparison of serum NT-proBNP levels in rats from each group; **(F)** Microscopic images of Masson’s trichrome-stained myocardium in rats from each group (×50, ×100, ×200), where the blue-stained areas represent collagen deposition, indicating myocardial fibrosis; **(E)** Quantification of myocardial fibrosis as indicated by Masson’s trichrome staining, expressed as the percentage of fibrotic area. p^ns^ ≥ 0.05, p* < 0.05, p** < 0.01, p*** < 0.001, p**** < 0.0001. 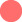 represents an individual sample data point. HDC, Astragalus mongholicus and Salvia miltiorrhiza Combination; AngII, Angiotensin II; HF, Heart failure; F, Ferrostatin-1; E, Erastin; L-S, Low Dosage Astragalus mongholicus and Salvia miltiorrhiza Medication-Containing Serum; H-S, High Dosage Astragalus mongholicus and Salvia miltiorrhiza Medication-Containing Serum; LVEF, Left Ventricular Ejection Fraction; NT-proBNP, N-terminal pro B-type Natriuretic Peptide; TAC, Transverse Aortic Constriction; L, Low Dosage Astragalus mongholicus and Salvia miltiorrhiza Decoction; H, High Dosage Astragalus mongholicus and Salvia miltiorrhiza Decoction.

As shown in Figure 2, both in animal and cell experiments, the model group exhibited significant HF compared to the control or Sham group. This was manifested by decreased cell viability, increased NT-ProBNP, enlarged heart, reduced LVEF, and aggravated myocardial fibrosis. When the ferroptosis inducer Era was used, HF symptoms further worsened, while the ferroptosis inhibitor Fer-1 alleviated these symptoms. Different concentrations of HDC had similar effects to the ferroptosis inhibitor Fer-1, with high-dose HDC generally being more effective than low-dose HDC, although there was no significant difference in improving LVEF between the doses. This validated that increased ferroptosis is a key factor in HF, and inhibiting ferroptosis can improve HF. Additionally, HDC effectively improved HF.

**FIGURE 3:**
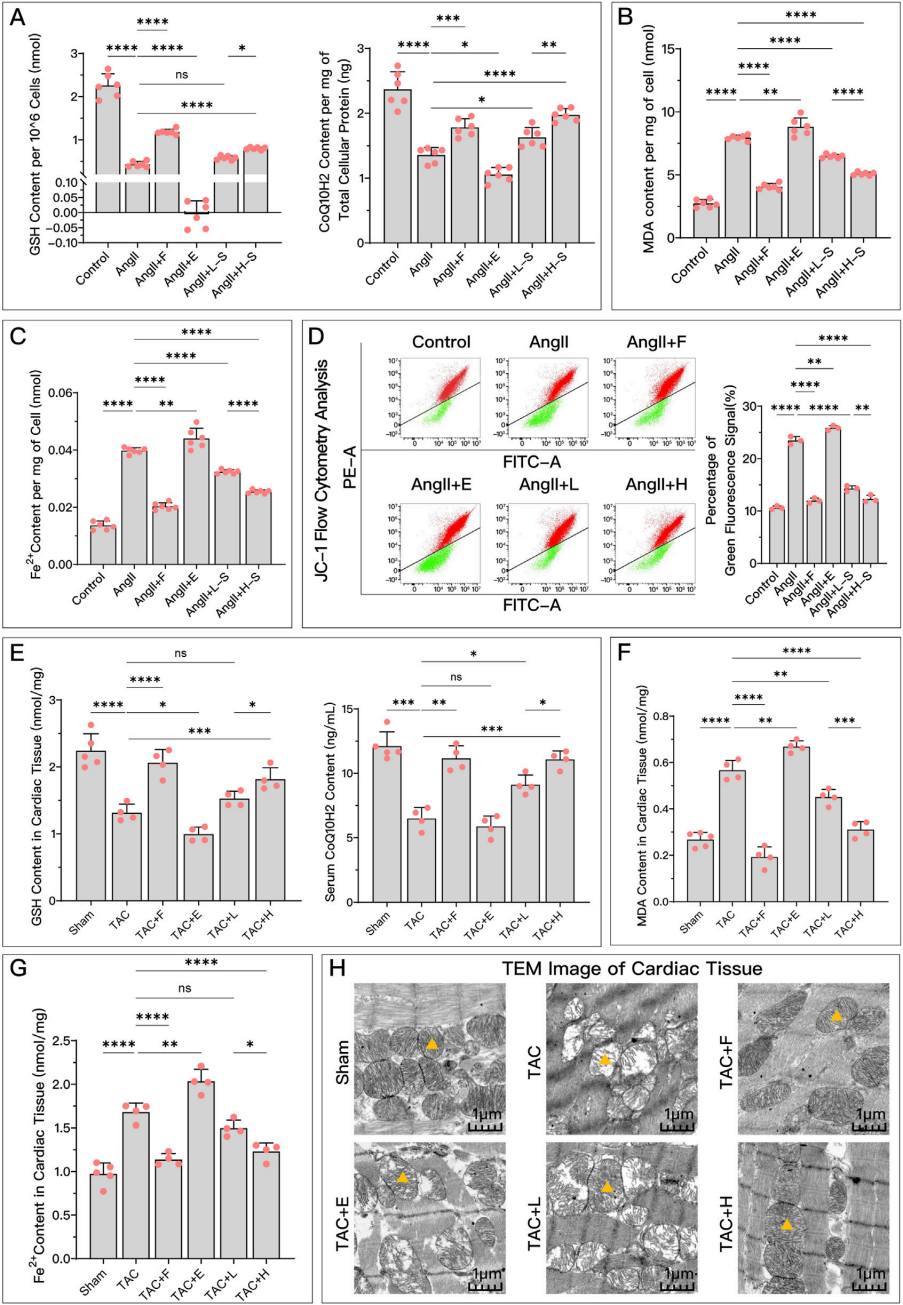
HDC effectively inhibits ferroptosis in HF. **(A)** Levels of antioxidants GSH and CoQ10H2 in H9c2 cells of each group; **(B)** Extent of lipid peroxidation (MDA content) in H9c2 cells of each group; **(C)** Levels of Fe^2+^ in H9c2 cells of each group; **(D)** Flow cytometry plots showing red and green fluorescence intensities after JC-1 staining of H9c2 cells from each group, and quantitative analysis of the proportion of green fluorescence signal; **(E)** GSH levels in myocardial tissue homogenates and serum CoQ10H2 levels in SD rats from each group; **(F)** MDA content in myocardial tissue homogenates of SD rats from each group; **(G)** Levels of Fe^2+^ in myocardial tissues of SD rats from each group; **(H)** TEM images of myocardial tissue from each group, with 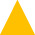 indicating one of the mitochondria in the image. p^ns^ ≥ 0.05, p* < 0.05, p** < 0.01, p*** < 0.001, p**** < 0.0001. 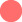 represents an individual sample data point. HDC, Astragalus mongholicus and Salvia miltiorrhiza Combination; HF, Heart failure; AngII, Angiotensin II; F, Ferrostatin-1; E, Erastin; L-S, Low Dosage Astragalus mongholicus and Salvia miltiorrhiza Medication-Containing Serum; H-S, High Dosage Astragalus mongholicus and Salvia miltiorrhiza Medication-Containing Serum; TAC, Transverse Aortic Constriction; L, Low Dosage Astragalus mongholicus and Salvia miltiorrhiza Decoction; H, High Dosage Astragalus mongholicus and Salvia miltiorrhiza Decoction; GSH, Reduced Glutathione; CoQ10H2, Reduced Coenzyme Q10; MDA, Malondialdehyde; PE-A, Phycoerythrin Area; FITC-A, Fluorescein Isothiocyanate Area.

As shown in Figure 3, compared to the control or Sham group, the model group exhibited significant changes in ferroptosis-related indicators. Antiferroptosis-related GSH and CoQ10H2 levels (Figures 3A, E) were significantly reduced, while ferroptosis-related lipid peroxidation indicator MDA (Figures 3B, F) and F^2+^ (Figures 3C, G) increased. Additionally, mitochondrial membrane potential significantly decreased (Figure 3D), and mitochondria became smaller with cristae fusion loss (Figure 3H), which are signs of ferroptosis. Compared to the model group, different concentrations of serum containing HDC generally improved these ferroptosis-related indicators, confirming that HDC, like the positive control drug Fer-1, has an inhibitory regulatory effect on ferroptosis. The regulatory effect of high-dose HDC was better than that of low-dose HDC, mainly because low-dose serum could not effectively restore GSH levels or significantly reduce iron deposition in myocardial tissue.

### 3.5 HDC’s GPX4-dependent role in improving HF via ferroptosis inhibition

**FIGURE 4:**
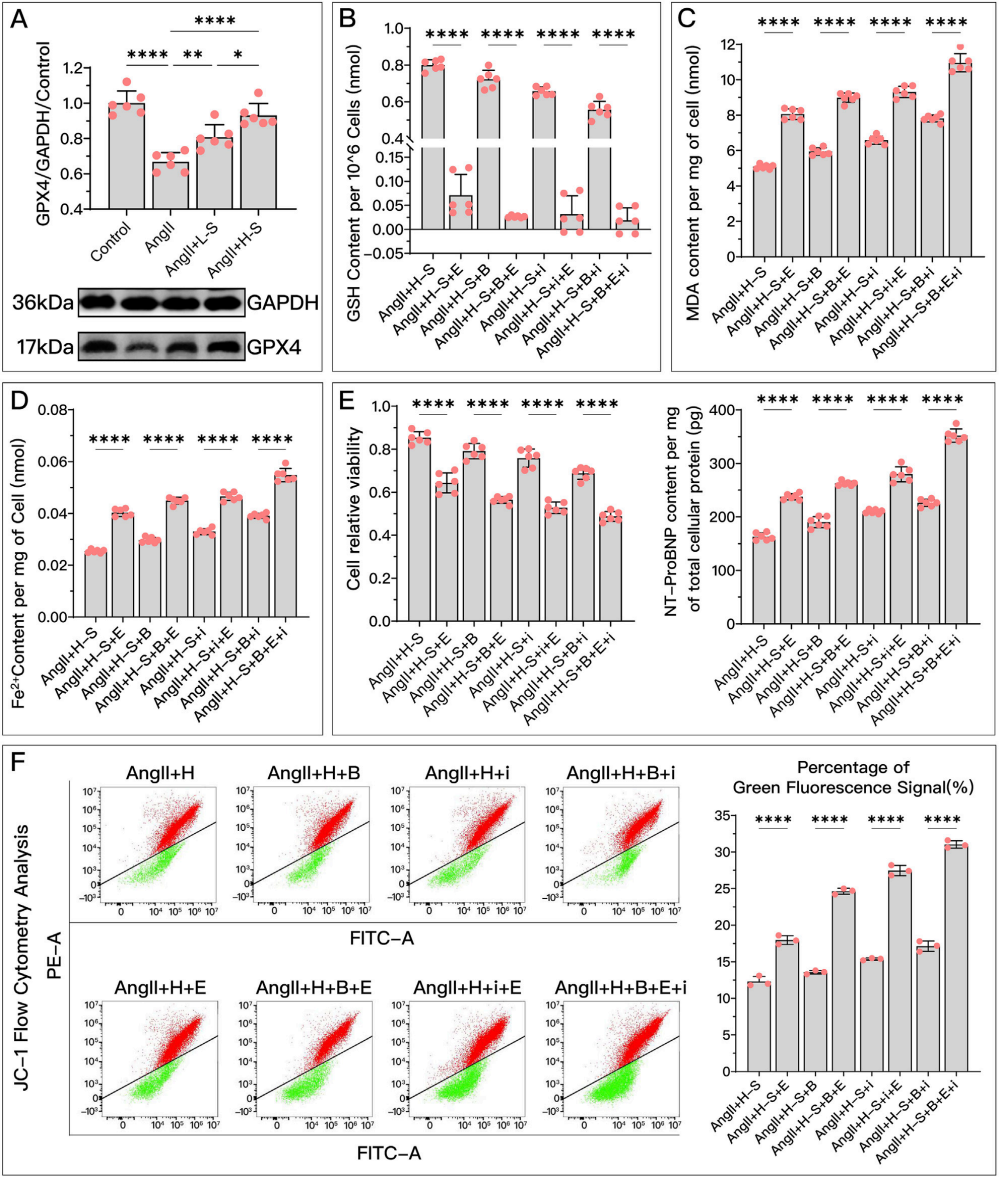
HDC ameliorates H9c2 cell dysfunction by inhibiting ferroptosis through the elevation of GPX4 levels. **(A)** Western blotting analysis of GPX4 levels in H9c2 cells from various groups to evaluate the effect of AngII and various doses of HDC on GPX4 content. **(B–F)** All panels represent the conditions of H9c2 cells under HDC intervention, assessing the effects of GPX4 inhibition and non-inhibition in varying FSP1 and DHODH states. **(B–E)** Measurements in H9c2 cells include GSH levels, MDA content, Fe^2+^ levels, cell viability, and NT-proBNP levels. **(F)** Flow cytometry images of H9c2 cells post JC-1 staining and quantitative analysis of the proportion of green fluorescence signal in flow cytometry. p* < 0.05, p** < 0.01, p**** < 0.0001. 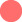 represents an individual sample data point. HDC, Astragalus mongholicus and Salvia miltiorrhiza Combination; GPX4, Glutathione Peroxidase 4; GAPDH, Glyceraldehyde 3-Phosphate Dehydrogenase; AngII, Angiotensin II; L-S, Low Dosage Astragalus mongholicus and Salvia miltiorrhiza Medication-Containing Serum; H-S, High Dosage Astragalus mongholicus and Salvia miltiorrhiza Medication-Containing Serum; GSH, Reduced Glutathione; FSP1, Ferroptosis Suppressor Protein 1; DHODH, Dihydroorotate Dehydrogenase; E, Erastin; B, Brequinar; i, Inhibitor of FSP1; MDA, Malondialdehyde; NT-proBNP, N-terminal pro B-type Natriuretic Peptide; PE-A, Phycoerythrin Area; FITC-A, Fluorescein Isothiocyanate Area.

**FIGURE 5:**
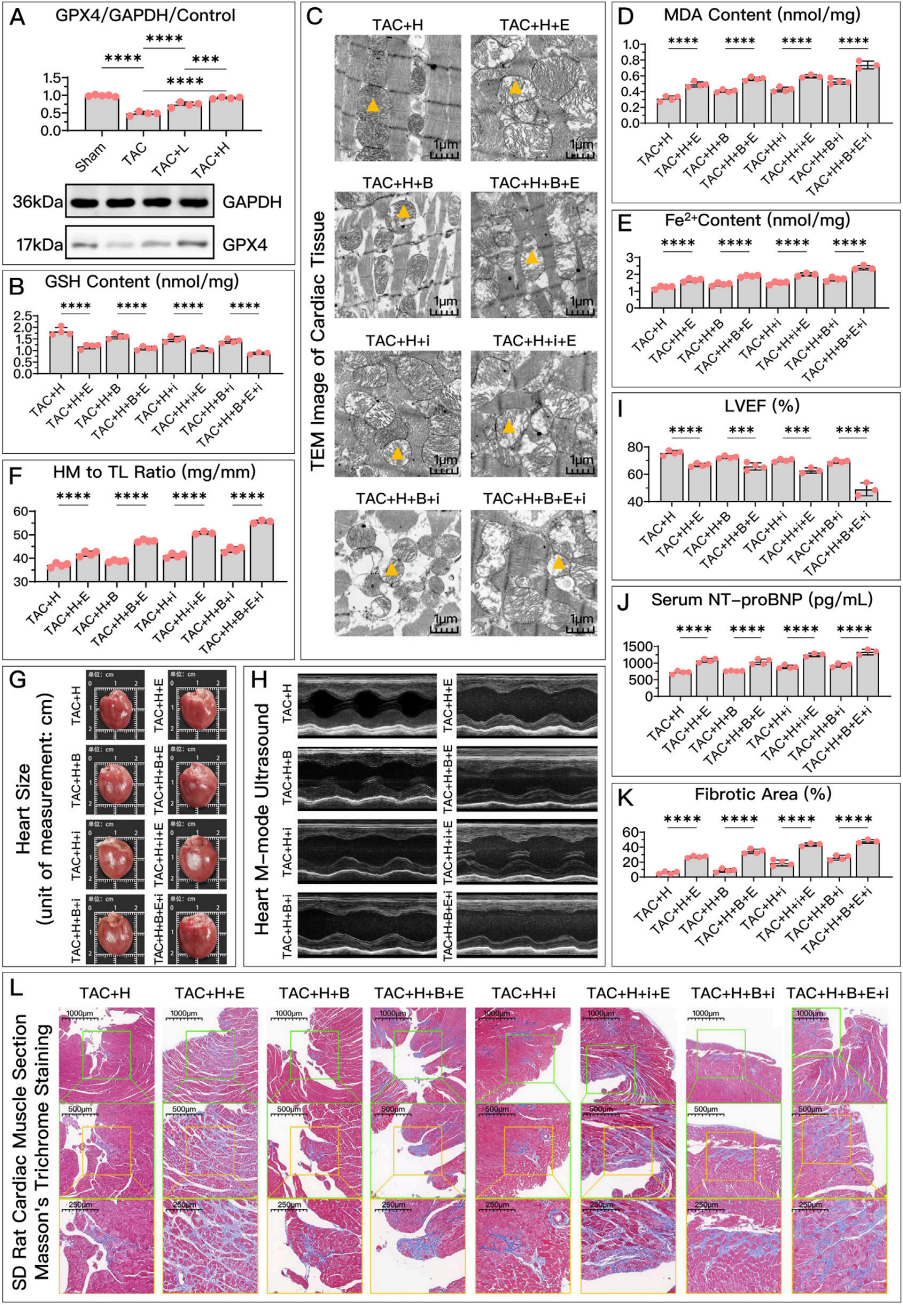
HDC improves HF by inhibiting ferroptosis via an increase in GPX4 levels. **(A)** Western blotting analysis of GPX4 levels in the myocardium from SD rats of various groups to evaluate the impact of HDC on GPX4 content. **(B–L)** All panels depict the effects of GPX4 inhibition and non-inhibition under HDC treatment in rats with various FSP1 and DHODH states. **(B–E, J)** In HF rats, measurements include myocardial GSH levels, mitochondrial morphology (TEM images, 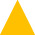 indicates one of the mitochondria), MDA levels, Fe^2+^ content, and serum NT-proBNP levels. **(F, G)** The heart mass to tibia length ratio and gross cardiac images in HF rats. **(H, I)** M-mode echocardiography images of the left ventricle adjacent to the sternum and quantitative analysis of LVEF in HF rats. **(L)** Masson’s trichrome staining of myocardial tissue in HF rats (×50, ×100, ×200 magnifications), with blue staining indicating collagen deposition, suggestive of myocardial fibrosis. **(K)** Quantitative analysis of fibrotic area based on Masson’s trichrome staining. p*** < 0.001, p**** < 0.0001. 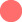 represents an individual sample data point. HDC, Astragalus mongholicus and Salvia miltiorrhiza Combination; HF, Heart failure; GPX4, Glutathione Peroxidase 4; GAPDH, Glyceraldehyde 3-Phosphate Dehydrogenase; TAC, Transverse Aortic Constriction; L, Low Dosage Astragalus mongholicus and Salvia miltiorrhiza Decoction; H, High Dosage Astragalus mongholicus and Salvia miltiorrhiza Decoction; FSP1, Ferroptosis Suppressor Protein 1; DHODH, Dihydroorotate Dehydrogenase; GSH, Reduced Glutathione; E, Erastin; B, Brequinar; i, Inhibitor of FSP1; TEM, Transmission Electron Microscopy; MDA, Malondialdehyde; NT-proBNP, N-terminal pro B-type Natriuretic Peptide; HM, Heart Mass; TL, Tibia Length; LVEF, Left Ventricular Ejection Fraction.

Both *in vivo* and *in vitro*, HDC significantly restored the decreased GPX4 levels after HF, with the effect of high-dose HDC being more pronounced (Figures 4A, 5A).

Compared to the high-dose HDC group, the inhibition of GPX4 pathway using Era significantly weakened the ferroptosis inhibition and HF protection effects of HDC. To explore whether different states of FSP1 and DHODH affect the mediation of HDC’s protective effects by GPX4, we compared the HF protection effects of HDC when GPX4 was inhibited, under the conditions of FSP1 inhibition with iFSP1 and/or DHODH inhibition with BQR. The results showed that, compared to the groups without Era, blocking the GPX4 pathway led to reduced GSH (Figures 4B, 5B), increased MDA (Figures 4C, 5D), elevated Fe^2+^ (Figures 4D, 5E), increased mitochondrial membrane potential (Figure 4F), decreased cell viability and elevated NT-proBNP (Figures 4E, 5J), enlarged heart (Figure 5G), increased heart mass (Figure 5F), reduced LVEF (Figures 5H, I), and increased myocardial fibrosis (Figures 5K, L), with aggravated mitochondrial shrinking and cristae fusion (Figure 5C). This confirmed the important role of GPX4 in HDC’s inhibition of ferroptosis and improvement of HF.

### 3.6 HDC’s FSP1-dependent role in improving HF via ferroptosis inhibition

**FIGURE 6:**
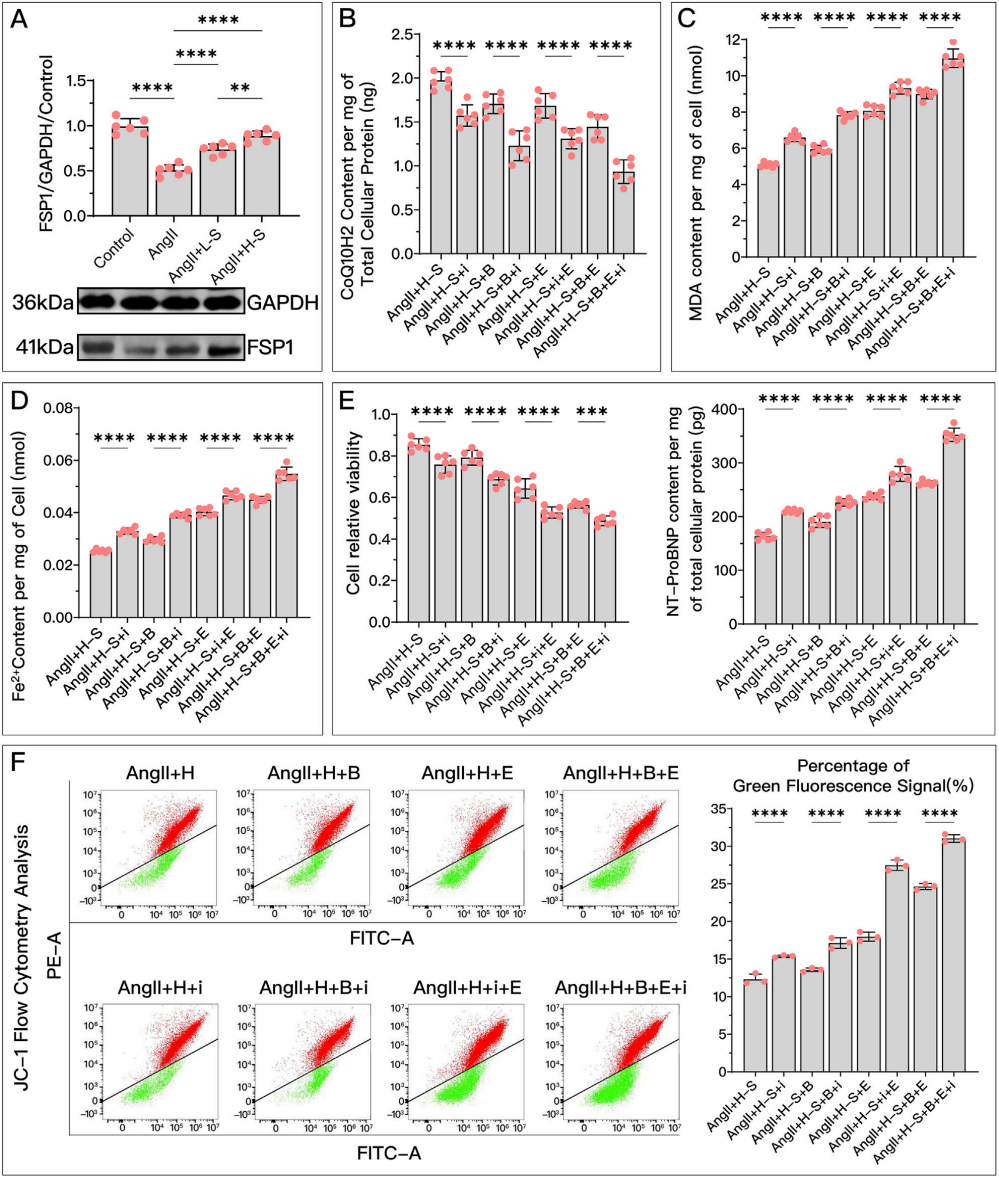
HDC ameliorates H9c2 cell dysfunction by increasing FSP1 levels to inhibit ferroptosis. **(A)** Western blotting analysis of FSP1 levels in H9c2 cells from various groups to assess the effects of AngII and different doses of HDC on FSP1 content. **(B–F)** These panels represent the HDC-treated cells, examining the effects of FSP1 inhibition and non-inhibition under varying GPX4 and DHODH states. **(B–E)** Measurements in H9c2 cells include CoQ10H2 levels, MDA levels, Fe^2+^ content, cell viability, and NT-proBNP levels. **(F)** Flow cytometry images of H9c2 cells post JC-1 staining and quantitative analysis of the green fluorescence signal proportion in flow cytometry charts. p** < 0.01, p**** < 0.0001. 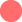 represents an individual sample data point. HDC, Astragalus mongholicus and Salvia miltiorrhiza Combination; FSP1, Ferroptosis Suppressor Protein 1; GAPDH, Glyceraldehyde 3-Phosphate Dehydrogenase; AngII, Angiotensin II; L-S, Low Dosage Astragalus mongholicus and Salvia miltiorrhiza Medication-Containing Serum; H-S, High Dosage Astragalus mongholicus and Salvia miltiorrhiza Medication-Containing Serum; CoQ10H2, Reduced Coenzyme Q10; GPX4, Glutathione Peroxidase 4; DHODH, Dihydroorotate Dehydrogenase; i, Inhibitor of FSP1; B, Brequinar; E, Erastin; MDA, Malondialdehyde; NT-proBNP, N-terminal pro B-type Natriuretic Peptide; PE-A, Phycoerythrin Area; FITC-A, Fluorescein Isothiocyanate Area.

**FIGURE 7:**
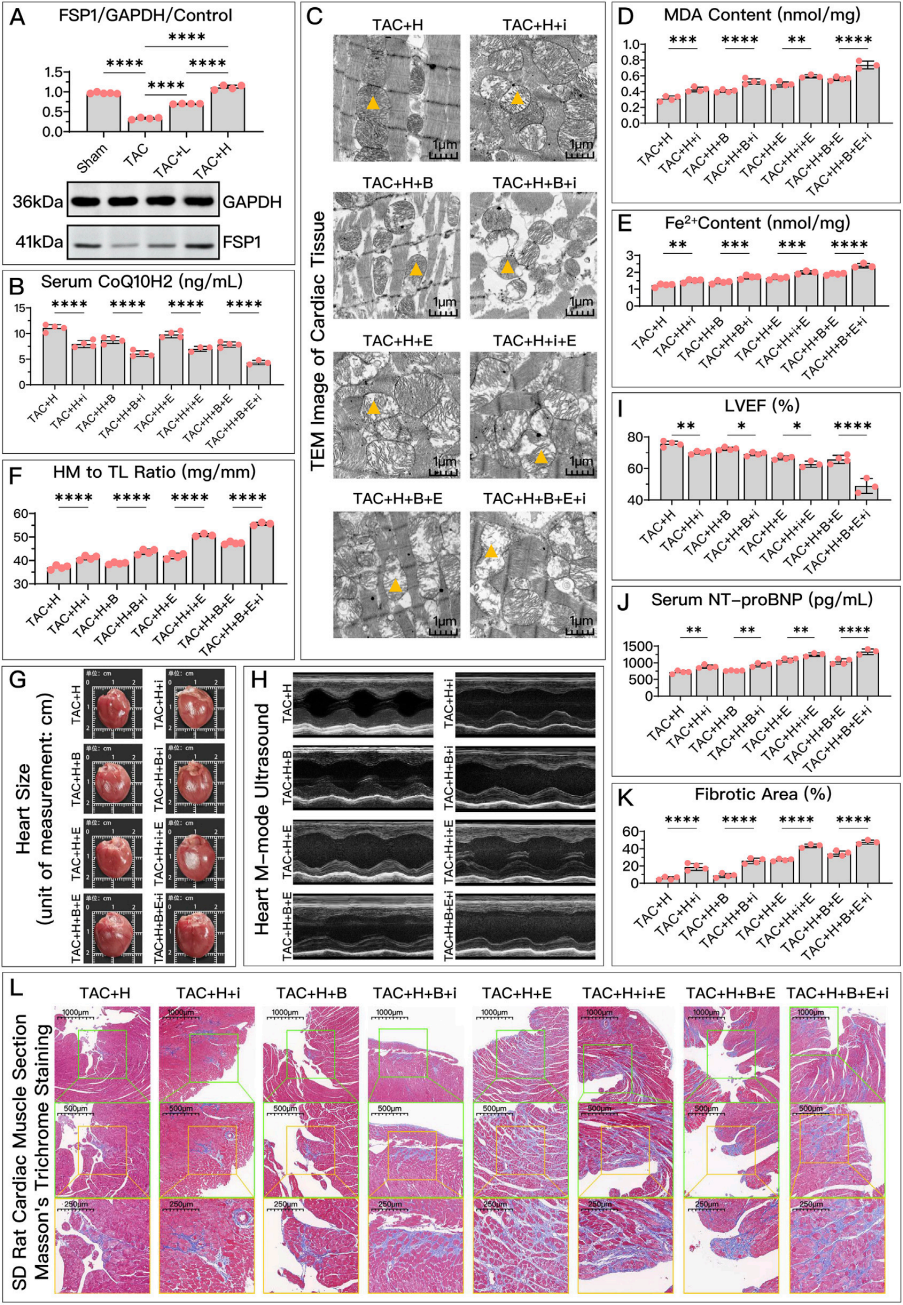
HDC improves HF by inhibiting ferroptosis through the elevation of FSP1 levels. **(A)** Western blotting analysis of FSP1 levels in the myocardium from SD rats of various groups to evaluate the impact of HDC on FSP1 content. **(B–K)** Analysis in HDC-treated rats under various GPX4 and DHODH states, assessing the effects of both FSP1 inhibition and non-inhibition. **(B–E, J)** Measurements in HF rats include myocardial CoQ10H2 levels, mitochondrial morphology (TEM images, 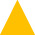 indicates one of the mitochondria), MDA levels, Fe^2+^ content, and serum NT-proBNP levels. **(F, G)** Heart mass to tibia length ratio and gross cardiac images in HF rats. **(H, I)** Parasternal long-axis M-mode echocardiography images of the left ventricle and quantitative analysis of LVEF in rats. **(L)** Masson’s trichrome staining of myocardial tissue in HF rats (×50, ×100, ×200 magnifications), where blue staining indicates collagen deposition, indicative of myocardial fibrosis. **(K)** Quantitative analysis of the fibrotic area based on Masson’s trichrome staining. p* < 0.05, p** < 0.01, p*** < 0.001, p**** < 0.0001. 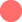 represents an individual sample data point. HDC, Astragalus mongholicus and Salvia miltiorrhiza Combination; HF, Heart failure; FSP1, Ferroptosis Suppressor Protein 1; GAPDH, Glyceraldehyde 3-Phosphate Dehydrogenase; TAC, Transverse Aortic Constriction; L, Low Dosage Astragalus mongholicus and Salvia miltiorrhiza Decoction; H, High Dosage Astragalus mongholicus and Salvia miltiorrhiza Decoction; CoQ10H2, Reduced Coenzyme Q10; GPX4, Glutathione Peroxidase 4; DHODH, Dihydroorotate Dehydrogenase; i, Inhibitor of FSP1; B, Brequinar; E, Erastin; TEM, Transmission Electron Microscopy; MDA, Malondialdehyde; NT-proBNP, N-terminal pro B-type Natriuretic Peptide; HM, Heart Mass; TL, Tibia Length; LVEF, Left Ventricular Ejection Fraction.

Both *in vivo* and *in vitro* experiments, as shown in Figures 6A, 7A, the FSP1 levels in the model group were significantly reduced compared to the control or sham group. After treatment with HDC, FSP1 levels were restored, with the high-dose HDC group showing more significant effects than the low-dose HDC group.

Compared to the high-dose HDC group, inhibiting FSP1 with iFSP1 significantly weakened the inhibitory effects of HDC on ferroptosis and its protective effects on HF. Under the conditions of Era inhibiting the GPX4 pathway and/or BQR inhibiting DHODH, the protective effects of HDC were still significantly suppressed due to the inactivation of FSP1. Specifically, after inhibiting FSP1, there was a significant decrease in CoQ10H2 (Figures 6B, 7B), an increase in MDA (Figures 6C, 7D), an increase in Fe^2+^ (Figures 6D, 7E), a greater reduction in mitochondrial membrane potential (Figure 6F), decreased cell viability, and elevated NT-proBNP (Figures 6E, 7J), increased myocardial hypertrophy (Figures 7F, G), reduced LVEF (Figures 7H, I), increased myocardial fibrosis (Figures 7K, L), and aggravated mitochondrial shrinkage and cristae fusion (Figure 7C). Both cell and animal experiments indicate that HDC can restore FSP1 levels after HF, and FSP1 plays an important role in the inhibition of ferroptosis and the improvement of HF by HDC.

### 3.7 HDC’s conditionally DHODH-dependent role in improving HF via ferroptosis inhibition

**FIGURE 8:**
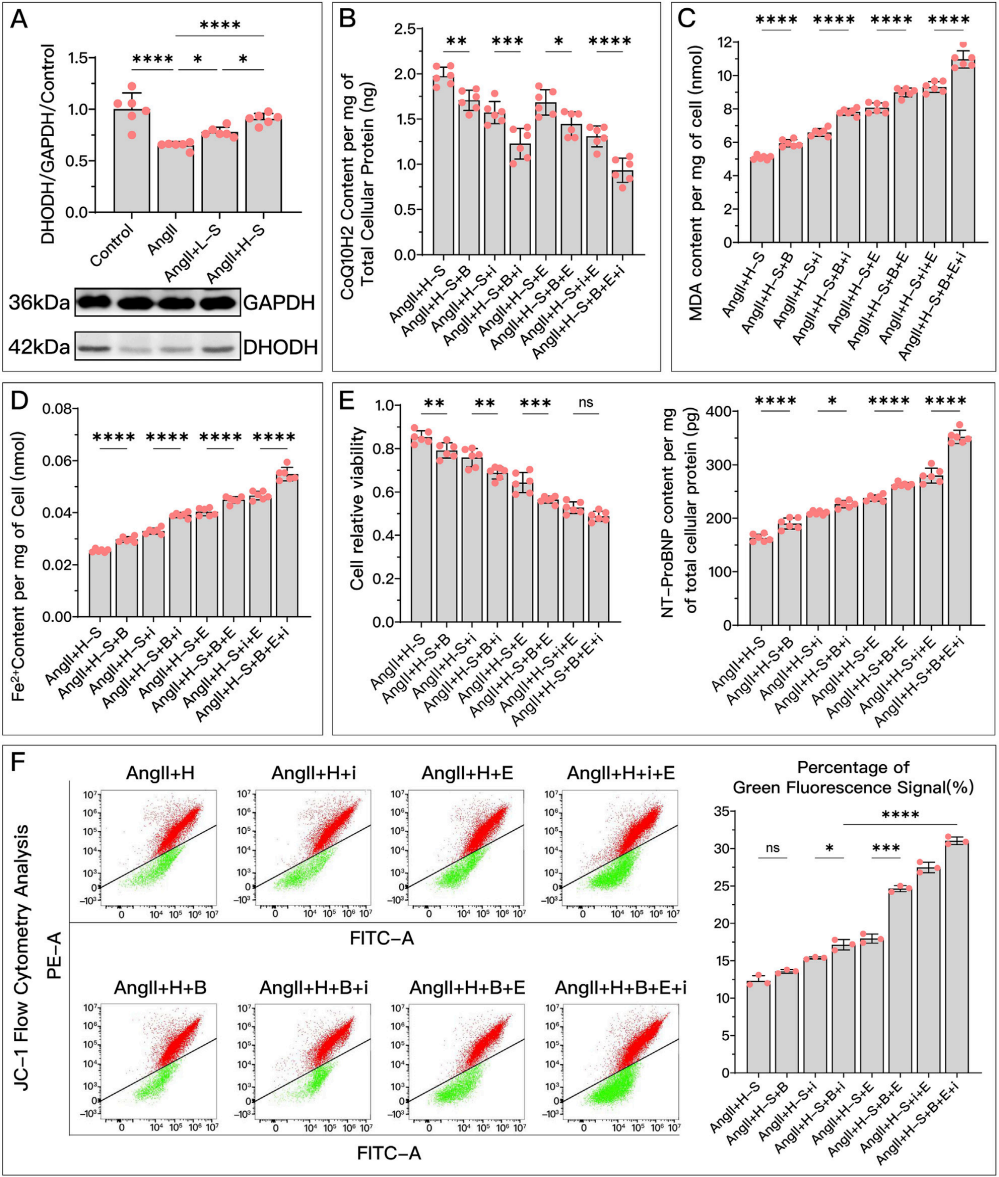
HDC ameliorates H9c2 cell dysfunction by inhibiting ferroptosis through the elevation of DHODH levels. **(A)** "Western blotting analysis of DHODH levels in H9c2 cells from various groups, assessing the effects of AngII and different doses of HDC on DHODH content. **(B–F)** Experiments conducted on cells treated with HDC, evaluating the impact of inhibiting or not inhibiting DHODH under different GPX4 and iFSP1 states. **(B–E)** Measurements in H9c2 cells include CoQ10H2 levels, MDA levels, Fe^2+^ content, cell viability, and NT-proBNP levels. Flow cytometry images of H9c2 cells post JC-1 staining and quantitative analysis of the green fluorescence signal proportion in flow cytometry charts. p^ns^ ≥ 0.05, p* < 0.05, p** < 0.01, p*** < 0.001, p**** < 0.0001. 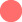 represents an individual sample data point. HDC, Astragalus mongholicus and Salvia miltiorrhiza Combination; DHODH, Dihydroorotate Dehydrogenase; WB, Western blotting; GAPDH, Glyceraldehyde 3-Phosphate Dehydrogenase; AngII, Angiotensin II; L-S, Low Dosage Astragalus mongholicus and Salvia miltiorrhiza Medication-Containing Serum; H-S, High Dosage Astragalus mongholicus and Salvia miltiorrhiza Medication-Containing Serum; CoQ10H2, Reduced Coenzyme Q10; GPX4, Glutathione Peroxidase 4; FSP1, Ferroptosis Suppressor Protein 1; B, Brequinar; i, Inhibitor of FSP1; E, Erastin; MDA, Malondialdehyde; NT-proBNP, N-terminal pro B-type Natriuretic Peptide; PE-A, Phycoerythrin Area; FITC-A, Fluorescein Isothiocyanate Area.

**FIGURE 9:**
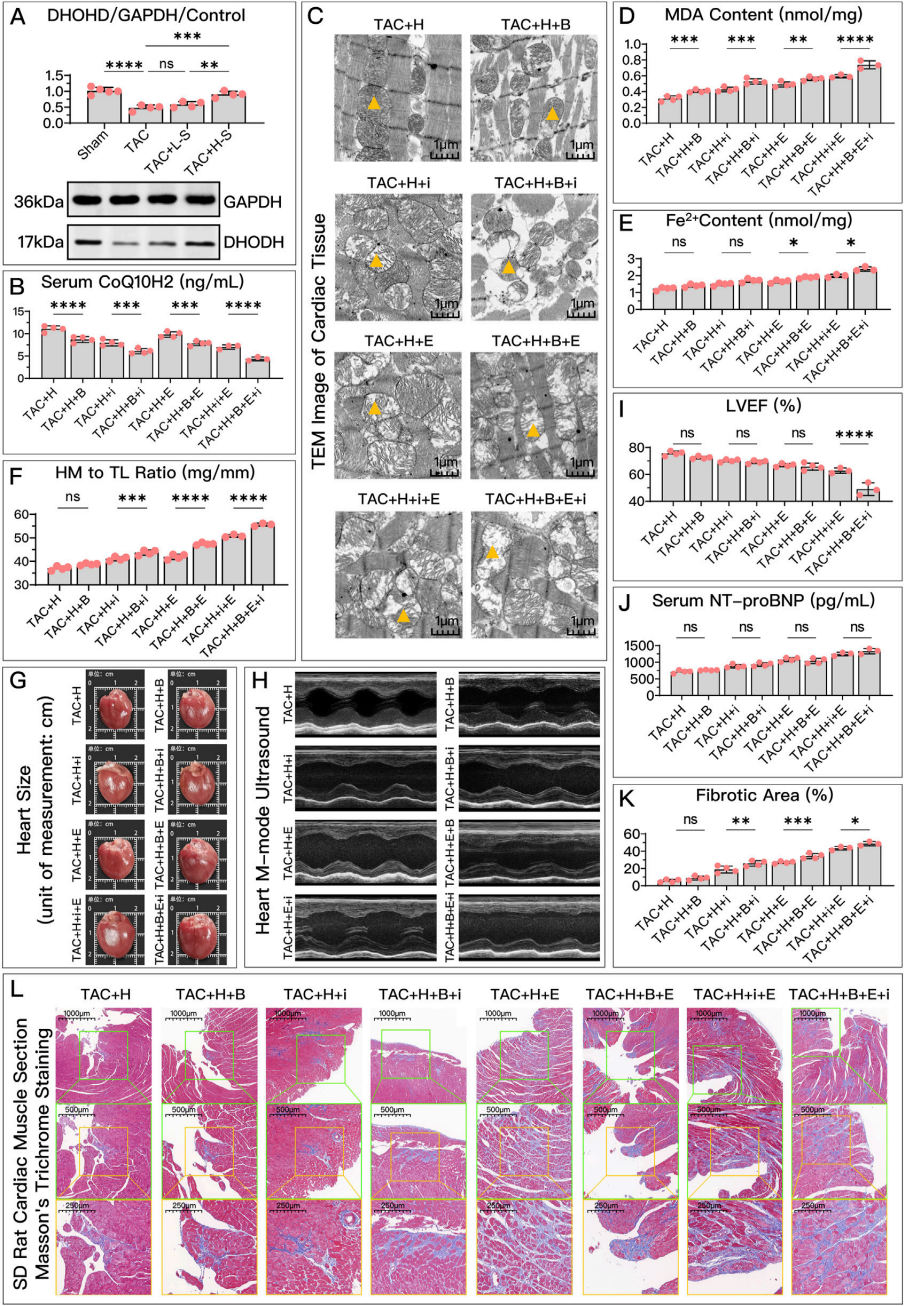
HDC mitigates HF by inhibiting ferroptosis through the elevation of DHODH levels. **(A)** Western blotting analysis of DHODH levels in the myocardium from SD rats of various groups to assess the impact of HDC on DHODH content. **(B–L)** Analysis in HDC-treated rats under different GPX4 and FSP1 states, evaluating the effects of DHODH inhibition and non-inhibition. **(B–E, J)** Measurements in HF rats include myocardial CoQ10H2 levels, mitochondrial morphology (TEM images, 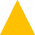 indicates one of the mitochondria), MDA levels, Fe^2+^ content, and serum NT-proBNP levels. **(F, G)** Heart mass to tibia length ratio and gross cardiac images in HF rats. **(H, I)** Parasternal long-axis M-mode echocardiography images of the left ventricle and quantitative analysis of LVEF in rats. **(L)** Masson’s trichrome staining of myocardial tissue in HF rats (×50, ×100, ×200 magnifications), where blue staining indicates collagen deposition, suggestive of myocardial fibrosis. **(K)** Quantitative analysis of the fibrotic area based on Masson’s trichrome staining. p^ns^≥0.05, p*<0.05, p**<0.01, p***<0.001, p****<0.0001. 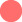 represents an individual sample data point. HDC, Astragalus mongholicus and Salvia miltiorrhiza Combination; HF, Heart failure; DHODH, Dihydroorotate Dehydrogenase; GAPDH, Glyceraldehyde 3-Phosphate Dehydrogenase; TAC, Transverse Aortic Constriction; L, Low Dosage Astragalus mongholicus and Salvia miltiorrhiza Decoction; H, High Dosage Astragalus mongholicus and Salvia miltiorrhiza Decoction; CoQ10H2, Reduced Coenzyme Q10; GPX4, Glutathione Peroxidase 4; FSP1, Ferroptosis Suppressor Protein 1; B, Brequinar; i, Inhibitor of FSP1; E, Erastin; TEM, Transmission Electron Microscopy; MDA, Malondialdehyde; NT-proBNP, N-terminal pro B-type Natriuretic Peptide; HM, Heart Mass; TL, Tibia Length; LVEF, Left Ventricular Ejection Fraction.

As shown in Figures 8A, 9A, compared to the control group, the DHODH content in the model group significantly decreased. HDC treatment effectively restored DHODH levels, but the restorative effect of low-dose HDC on DHODH was not apparent in *in vivo* experiments. Inhibiting DHODH with BQR did not always weaken all of HDC’s protective effects.

Specifically, in *in vitro* experiments, HDC’s ability to increase CoQ10H2 (Figure 8B), reduce MDA (Figure 8C), lower Fe^2+^ (Figure 8D), and decrease NT-proBNP (Figure 8E) was diminished due to DHODH inhibition. In *in vivo* experiments, HDC’s effects in increasing CoQ10H2 (Figure 9B), reducing MDA (Figure 9D), and improving mitochondrial morphology and structure (Figure 9C) were also inhibited. Regardless of whether the FSP1 and GPX4 pathways were blocked, HDC’s protective effects mentioned above were weakened due to DHODH inhibition.

As shown in Figure 8E, in terms of improving cell viability, DHODH inhibition could weaken HDC’s effect, but this inhibitory effect was no longer obvious when both the FSP1 and GPX4 pathways were blocked. As shown in Figure 8F, inhibiting DHODH alone could not weaken HDC’s effect on restoring mitochondrial membrane potential, but when the FSP1 and/or GPX4 pathways were blocked, HDC’s effect on restoring mitochondrial membrane potential was weakened due to DHODH inhibition.

In *in vivo* experiments, DHODH’s mediating ability in HDC’s inhibition of ferroptosis and improvement of HF was more unstable. DHODH inactivation alone did not weaken HDC’s effects in reducing Fe^2+^ (Figure 9E), alleviating myocardial hypertrophy (Figures 9F, G), reducing myocardial fibrosis (Figures 9K, L), and improving LVEF (Figures 9H, I). However, DHODH’s role was not completely ineffective. As shown in Figures 9F, G, K, L, when the FSP1 and/or GPX4 pathways were blocked, compared to when DHODH was not inhibited, inhibiting DHODH aggravated myocardial hypertrophy and myocardial fibrosis. As shown in Figure 9E, once the GPX4 pathway was blocked, inhibiting DHODH weakened HDC’s effect on reducing Fe^2+^. As shown in Figure 9I, when both the GPX4 and FSP1 pathways were blocked, DHODH’s mediating role in HDC’s improvement of ejection fraction became apparent. As shown in Figure 9J, in terms of reducing serum NT-proBNP, regardless of the status of the FSP1 and GPX4 pathways, DHODH inhibition did not weaken HDC’s effect on lowering NT-proBNP.

Overall, DHODH’s mediating role in HDC’s inhibition of ferroptosis and subsequent improvement of HF seems to depend on the status of the pathways involving GPX4 and FSP1. It is possible that DHODH’s mediating role is weaker and is overshadowed by the strong mediating roles of GPX4 and FSP1. Therefore, we hope to further compare the strengths of the mediating roles of these three key proteins.

### 3.8 In the process of HDC improving HF by inhibiting ferroptosis, the mediating strengths of GPX4, FSP1, and DHODH vary

**FIGURE 10:**
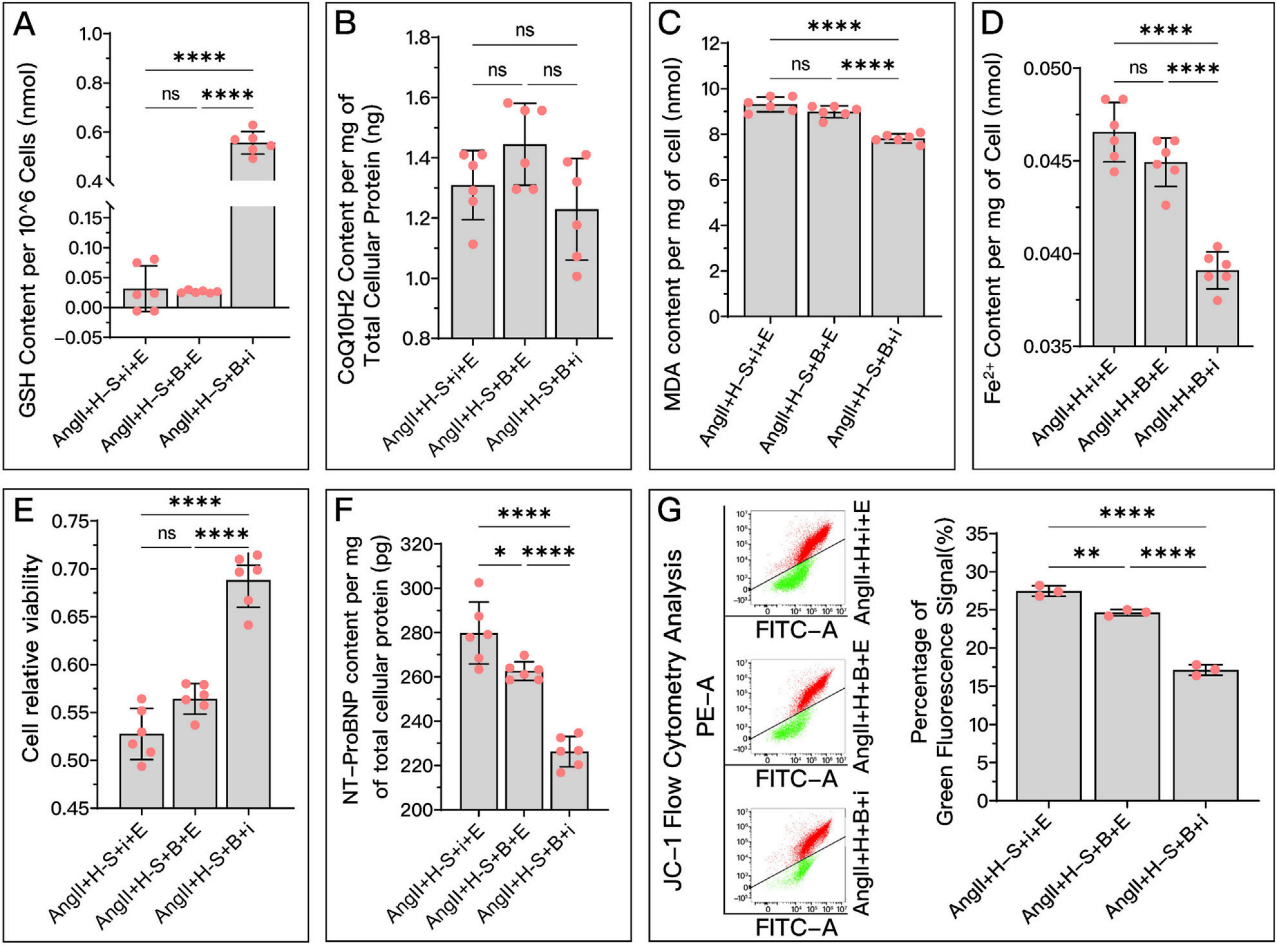
Comparative analysis of the effectiveness of GPX4, FSP1, and DHODH in mediating HDC’s improvement of H9c2 cell dysfunction. In this figure, H9c2 cells are divided into three groups: the AngII + H-S + i + E group, primarily mediated by DHODH for HDC’s protective effect; the AngII + H-S + B + E group, primarily mediated by FSP1 for HDC’s protective effect; and the AngII + H-S + B + i group, primarily mediated by GPX4 for HDC’s protective effect. **(A–F)** Measurements in these three groups of H9c2 cells include GSH levels, CoQ10H2 levels, MDA levels, Fe^2+^ content, cell viability, and NT-proBNP levels. **(G)** Flow cytometry images post JC-1 staining of the H9c2 cells and the quantitative analysis of the proportion of green fluorescence signal. p^ns^≥0.05, p*<0.05, p**<0.01, p****<0.0001. 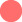 represents an individual sample data point. GPX4, Glutathione Peroxidase 4; FSP1, Ferroptosis Suppressor Protein 1; DHODH, Dihydroorotate Dehydrogenase; HDC, Astragalus mongholicus and Salvia miltiorrhiza Combination; AngII, Angiotensin II; H-S, High Dosage Astragalus mongholicus and Salvia miltiorrhiza Medication-Containing Serum; i (iFSP1), Inhibitor of FSP1; E, Erastin; B, Brequinar; GSH, Reduced Glutathione; CoQ10H2, Reduced Coenzyme Q10; MDA, Malondialdehyde; NT-proBNP, N-terminal pro B-type Natriuretic Peptide; PE-A, Phycoerythrin Area; FITC-A, Fluorescein Isothiocyanate Area.

**FIGURE 11:**
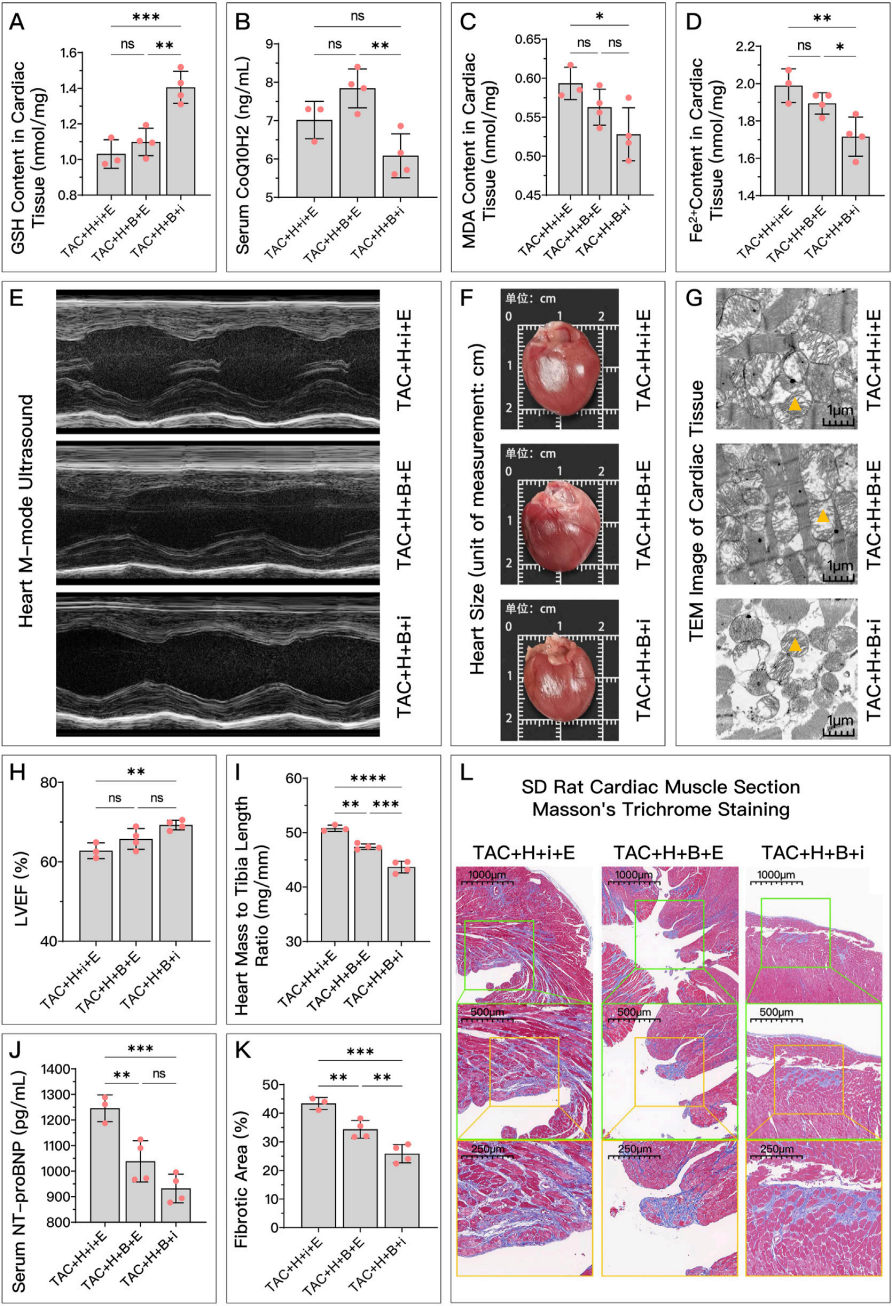
Comparative analysis of the effectiveness of GPX4, FSP1, and DHODH in mediating HDC’s amelioration of HF in SD rats. In this study, SD rats with HF were divided into three groups: TAC + H + i + E group, primarily mediated by DHODH for HDC’s protective effect; TAC + H + B + E group, primarily mediated by FSP1 for HDC’s protective effect; and TAC + H + B + I group, primarily mediated by GPX4 for HDC’s protective effect. **(A–D)** Measurements in these groups include myocardial GSH levels, MDA levels, Fe^2+^ content, and serum CoQ10H2 levels in SD rats. **(E)** Parasternal long-axis echocardiography images of the left ventricle in these groups of SD rats. **(H)** Quantitative calculation of LVEF based on echocardiography images. **(F)** Gross cardiac images of the rats in these groups. **(I)** Heart mass to tibia length ratio in these groups of SD rats. **(G)** TEM images of myocardial tissue, 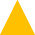 indicates one of the mitochondria. **(J)** Serum NT-proBNP levels in these groups of SD rats. (L) Masson’s trichrome staining of myocardial tissue (×50, ×100, ×200 magnifications), where blue staining indicates collagen deposition, suggestive of myocardial fibrosis. **(K)** Quantitative analysis of the fibrotic area ratio based on Masson’s trichrome staining images. p^ns^≥0.05, p*<0.05, p**<0.01, p****<0.0001. 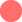 represents an individual sample data point. GPX4, Glutathione Peroxidase 4; FSP1, Ferroptosis Suppressor Protein 1; DHODH, Dihydroorotate Dehydrogenase; HDC, Astragalus mongholicus and Salvia miltiorrhiza Combination; HF, Heart failure; TAC, Transverse Aortic Constriction; H, High Dosage Astragalus mongholicus and Salvia miltiorrhiza Decoction; i, Inhibitor of FSP1; E, Erastin; B, Brequinar; GSH, Reduced Glutathione; CoQ10H2, Reduced Coenzyme Q10; MDA, Malondialdehyde; LVEF, Left Ventricular Ejection Fraction; NT-proBNP, N-terminal pro B-type Natriuretic Peptide; PE-A, Phycoerythrin Area; FITC-A, Fluorescein Isothiocyanate Area.

As shown in Figures 10, 11, each group uses two of the three protein inhibitors to retain the function of one pathway, minimizing the influence of the other two pathways. By comparing the three groups, we can determine the relative strength of the three key proteins in mediating HDC’s inhibition of ferroptosis and improvement of HF.

In *in vitro* experiments, Figures 10A, C–E show that in terms of HDC increasing GSH, decreasing MDA, reducing Fe^2+^, and improving cell viability, the mediating effect of GPX4 (AngII + H + B + i) is stronger than that of FSP1 (AngII + H + B + E) and DHODH (AngII + H + i + E), while the mediating ability of FSP1 and DHODH is similar. Figure 10B shows that there is no significant difference among the three proteins in mediating HDC’s ability to increase COQ10H2. Figure 10E shows that GPX4 has a stronger mediating effect on HDC’s improvement of cell viability compared to FSP1. GPX4 has a stronger mediating effect on HDC’s reduction of NT-proBNP (Figure 10F) and restoration of mitochondrial membrane potential (Figure 10G) compared to FSP1, and FSP1 is stronger than DHODH.

*In vivo* experiments, Figures 11A, D, G show that in terms of HDC increasing GSH, reducing Fe^2+^, and improving mitochondrial structure, the mediating effect of GPX4 (TAC + H + B + i) is stronger than that of FSP1 (TAC + H + B + E) and DHODH (TAC + H + i + E), while the mediating ability of FSP1 and DHODH is similar. In mediating HDC’s increase of CoQ10H2 (Figure 11B), FSP1’s mediating effect is stronger than that of GPX4, and DHODH’s mediating effect is intermediate, but there is no statistical difference between GPX4 and FSP1. As shown in Figures 11C, E, H, GPX4 has a stronger mediating effect on HDC’s reduction of MDA and improvement of LVEF compared to DHODH, with FSP1’s effect being intermediate but not significantly different from GPX4 and DHODH. Figures 11F, I, K, L show that in mediating HDC’s improvement of myocardial hypertrophy and myocardial fibrosis, GPX4 is stronger than FSP1 and DHODH, and FSP1 is stronger than DHODH. As shown in Figure 11J, in mediating HDC’s reduction of serum NT-proBNP, DHODH is weaker than GPX4 and FSP1, with no significant difference between GPX4 and FSP1.

## 4 Discussion

Currently, HF remains a serious global health challenge. One of the fundamental pathological mechanisms of HF is myocardial remodeling, which, once established, is difficult to reverse (Zhang et al., 2022). Therefore, despite continuous innovation and progress in treatment methods, HF remains an unresolved medical challenge. Even with ongoing treatment, patients with HF often experience recurrent episodes and worsening of the condition (Greene et al., 2023). This underscores the importance of early prevention and treatment. TCM offers many approaches that have been proven effective in treating HF. Combining Western medicine with TCM in the prevention of HF shows great promise. TCM can complement and enhance Western treatment modalities, potentially increasing efficacy and reducing side effects. AM and SM, traditional Chinese botanical drugs frequently used in cardiovascular diseases, are often employed together in complex formulas as primary and auxiliary agents in the treatment of HF (Project Group of Traditional Chinese Medicine Guideline for Diagnosis and Treatment of Chronic Heart Failure and China Association of Chinese Medicine, 2023). Ferroptosis, a mode of programmed cell death discovered over a decade ago, characterized by iron-dependent lipid peroxidation and structural changes like mitochondrial membrane thickeningexp, volume reduction, and typical cristae fusion and loss (Dixon et al., 2012), has been established as a significant contributor to the progression of HF. Research indicates that inhibiting ferroptosis can halt the advancement of HF (Zhang et al., 2022; Sahebkar et al., 2023). The effectiveness of simplifying complex traditional formulas to just HDC in preventing and treating HF, and the underlying mechanisms of such an approach, however, remain to be elucidated.

In our study, we further explored the impact of HDC on HF, particularly in the context of heart function decline caused by overload stress. Through *in vitro* and *in vivo* experiments, we found that HDC significantly ameliorates heart function decline induced by overload stress. This includes improving cell viability, reducing NT-proBNP, alleviating myocardial remodeling, and maintaining ejection fraction. By comparing with ferroptosis inducers and inhibitors, we further clarified the key role of ferroptosis in the process of overload-induced HF and confirmed for the first time the important role of inhibiting ferroptosis in HDC’s improvement of HF. Specifically, HDC, like Fer-1, can increase the levels of GSH and CoQ10H2, thereby enhancing antioxidant capacity, inhibiting lipid peroxidation (reducing MDA), restoring mitochondrial membrane potential, and improving mitochondrial morphology and function, ultimately inhibiting ferroptosis. The improvement of these ferroptosis markers is synchronous with the alleviation of HF. When ferroptosis was induced using Era, an exacerbation of HF was observed. Previous studies (Sui et al., 2020; Lin et al., 2021; Li et al., 2023) support our findings, as they found that AM and SM, particularly their active metabolites such as Astragaloside and Tanshinone IIA, improve myocardial hypertrophy, myocardial fibrosis, and ejection fraction through multiple pathways. Additionally, pharmacological interventions that reduce ferroptosis have been shown to inhibit the progression of HF (Chen et al., 2023).

The Nrf2/cGPX4/GSH pathway, FSP1/CoQ10/NADPH pathway, mitochondrial DHODH/CoQ10 pathway, and mGPX4/GSH pathway are the primary regulatory pathways of ferroptosis (Mao et al., 2021), highlighting GPX4, FSP1, and DHODH as key proteins in regulating ferroptosis. These proteins primarily inhibit ferroptosis by suppressing lipid peroxidation (Mao et al., 2021). Our study uncovers for the first time that HDC can inhibit ferroptosis and improve HF by simultaneously regulating the GPX4/GSH, FSP1/CoQ10, and DHODH/CoQ10 pathways. In both *in vitro* and *in vivo* experiments, HDC increased the levels of these key proteins and downstream antioxidants (GSH, CoQ10H2). When we inhibited these key regulatory proteins, the inhibitory effects of HDC on ferroptosis and the improvement in HF were reduced to varying degrees. This further confirms that these pathways are important targets for HDC in inhibiting ferroptosis. However, in *in vivo* experiments, the effect of HDC on reducing NT-proBNP levels through the regulation of DHODH was not observed, which is inconsistent with our cellular experiment results. This discrepancy may be due to several factors: firstly, DHODH’s role in regulating ferroptosis is relatively minor, as evidenced by our findings in result 3.8; secondly, HDC has numerous targets for improving HF (Wang et al., 2018; Nandi et al., 2020), not limited to ferroptosis regulated by GPX4, FSP1, and DHODH; and lastly, the systemic metabolic regulation in animals is more complex. These three factors combined lead to the insufficient inhibition of DHODH-mediated ferroptosis to significantly improve NT-proBNP levels post-HF, or the effects may be too weak to observed.

While exploring the key targets of HDC in inhibiting ferroptosis and improving HF, we found that inhibiting DHODH alone in certain situations did not weaken the protective effects of HDC. However, this does not necessarily mean that DHODH does not mediate the protective effects of HDC; it is possible that the effects of GPX4 and FSP1 are stronger, overshadowing the role of DHODH. Therefore, we used a combination of inhibitors for the three key proteins to assess their interactions during HDC treatment of HF. We found that during HDC treatment of HF, the pathways involving GPX4 and the pathways involving FSP1 were not affected by the other pathways. However, the role of DHODH in mediating the effects of HDC on inhibiting ferroptosis and improving HF was somewhat influenced by GPX4 and FSP1, or its effects were overshadowed by those of GPX4 and FSP1, particularly in inhibiting iron accumulation, improving mitochondrial membrane potential, reducing myocardial fibrosis, and improving LVEF. Once FSP1 and GPX4 were inhibited, the mediating effect of DHODH on the protective effects of HDC became apparent. Therefore, conclusions drawn from studies focusing solely on DHODH should be approached with caution. Studies (Mishima et al., 2023) have shown that DHODH plays a minor role in tumor cell ferroptosis, which questions its role as a ferroptosis-regulating protein and supports our findings.

We retained the function of one pathway by inhibiting two of the pathways involving GPX4, FSP1, and DHODH. This innovative approach allowed us to compare the relative strengths of these three key proteins in the process of HDC treatment for HF. Overall, GPX4 was found to mediate the protective effects of HDC most strongly, followed by FSP1, with DHODH having the weakest mediating effect. Previous research has generally considered GPX4 to be the most crucial regulatory protein in ferroptosis (Liu M. et al., 2022). However, in terms of increasing CoQ10H2 levels (Figures 10B, 11B), the effect of FSP1 was more significant than that of GPX4, while the effect of DHODH was comparable to both GPX4 and FSP1. This is because FSP1 and DHODH are the primary upstream proteins regulating CoQ10H2, rather than GPX4 (Liu et al., 2023). These findings provide valuable insights for personalized clinical treatments.

HF patients are often elderly and have multiple coexisting conditions. Currently or in the future, many drugs used to treat cancer, rheumatoid diseases, or other conditions work by promoting ferroptosis. For HF patients who also suffer from these conditions, it is crucial to choose treatments that minimize adverse impacts on HF. For example, if these ferroptosis-promoting drugs have comparable therapeutic effects, it would be prudent to consider using drugs that inhibit DHODH to achieve the desired outcomes. From our research, inhibiting DHODH has the least impact on HF treatment compared to inhibiting GPX4 or FSP1. Therefore, using DHODH inhibitors could potentially offer a dual benefit: treating cancer or rheumatoid diseases while minimally compromising HF management. This strategic approach underscores the importance of understanding the interplay between ferroptosis pathways and disease states, allowing for more nuanced and effective individualized treatment plans.

Due to budget constraints, conditional knockout models were not feasible, and specific inhibitors were used to block the pathways. While this approach may result in incomplete pathway inhibition, it does not affect the validity of the final conclusions. We strictly controlled variables during the study and ensured that there was only a single variable difference between the comparison groups. This ensured the high accuracy of the comparison results and conclusions. Additionally, in our experiments comparing the strengths of regulatory proteins, we used HDC treatment of HF as a unified background. This limits our results and conclusions to this specific context and cannot be extrapolated to other cells, diseases, or drug treatments. Moreover, since the decoction used in this study was prepared manually, we were only able to ensure the specific component content for this particular experiment. As the quality of botanical drugs is influenced by factors such as environment and processing methods, we cannot completely guarantee the consistency of the decoction. However, this more closely simulates the real-world situation of human consumption of herbal decoctions and does not affect the results and conclusions of this study. In future research, industrially processed decoctions could be used to ensure greater consistency and reproducibility. We also hope to conduct more direct comparisons of the relative strengths of ferroptosis regulatory proteins in future experiments, not limited to specific diseases or drug interventions, to better generalize the results.

## 5 Conclusion

This study unveils a novel mechanism where the combined use of the traditional Chinese botanical drugs AM and SM inhibits ferroptosis, thereby exerting cardioprotective effects. We demonstrate that this combination of botanical drugs mitigates lipid peroxidation, iron accumulation, and mitochondrial dysfunction by simultaneously modulating three key regulatory pathways: GPX4/GSH, FSP1/CoQ10, and DHODH/CoQ10, thereby inhibiting ferroptosis. Concomitantly, HF markers such as myocardial hypertrophy, fibrosis, reduced ejection fraction, and elevated NT-proBNP levels are also improved. Comparative analysis indicates that HDC primarily inhibit iron dysregulation through GPX4, followed by FSP1, while the role of DHODH is comparatively minor. The effectiveness of DHODH is contingent upon the functional status of the other pathways. Overall, these findings emphasize ferroptosis inhibition as a key mechanism underlying the therapeutic efficacy of HDC in treating HF, providing new molecular evidence for the benefits of this combination of traditional botanical drugs. Additionally, this study suggests future research directions, recommending that DHODH may not be a suitable primary target in studies aimed at inhibiting ferroptosis to improve HF.

## Data Availability

The original contributions presented in the study are publicly available. This data can be found here: https://zenodo.org/records/14418483.
