# Supplementary material for: Combination decoction of Astragalus mongholicus and Salvia miltiorrhiza mitigates pressure-overload cardiac dysfunction by inhibiting multiple ferroptosis pathways
Source: Front Pharmacol. 2024 Dec 16;15:1447546. doi: 10.3389/fphar.2024.1447546 (PMC11683366; doi:10.3389/fphar.2024.1447546)
Supplement: Supplementary file 1 [file DataSheet2.pdf]

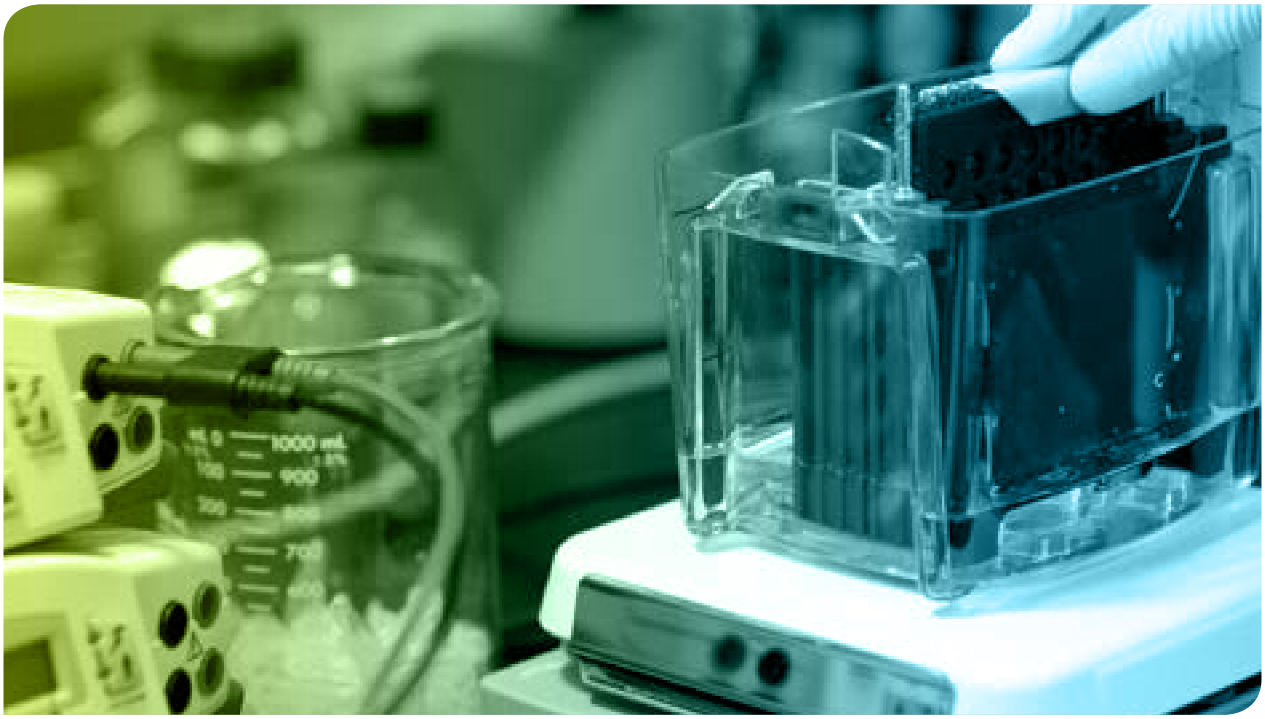

Protein Blotting Guide

Part 1 Theory and Products 5
Chapter 1 Overview of Protein Blotting 5
Transfer 6
Detection 6
General Considerations and Workflow 6
Chapter 2 Methods and Instrumentation 9
Protein Blotting Methods 10
Electrophoretic Transfer 10
Tank Blotting 10
Semi-Dry Blotting 11
Microfiltration (Dot Blotting)
Blotting Systems and Power Supplies 12
Tank Blotting Cells 12
Mini Trans-Blot® Cell and Criterion™ Blotter 12
Trans-Blot® Cell 12
Trans-Blot® Plus Cell 13
Semi-Dry Blotting Cells 13
Trans-Blot® SD Semi-Dry Cell 14
Trans-Blot® Turbo™ System 14
Microfiltration Apparatus 14
Bio-Dot® and Bio-Dot® SF Apparatus 14
Power Supplies for Electrophoretic Transfers 15
PowerPac™ HC Power Supply 15
PowerPac™ Universal Power Supply 15
Chapter 3 Membranes and Transfer Buffers 17
Membranes and Blotting Papers 18
Nitrocellulose and Supported Nitrocellulose 18
Polyvinylidene Difluoride (PVDF) 18
Immun-Blot® and Immun-Blot LF PVDF for Western Blotting 18
Sequi-Blot™ PVDF for Protein Sequencing 18
Blotting Filter Papers 19
Membrane/Filter Paper Sandwiches 19
Transfer Buffers 19
Towbin and Bjerrum Schafer-Nielsen Buffers (Tris/Glycine Buffers) 20
CAPS Buffer 20
Discontinuous Tris-CAPS Buffer System (for Semi-Dry Transfer) 20
Dunn Carbonate Buffer 21
Other Buffers 21
Chapter 4 Transfer Conditions 23
General Workflow – Electrophoretic Transfer 24
Power Conditions 24
Useful Equations 24
Joule Heating and Other Factors Affecting Transfer 24
Relationship Between Power Settings and Transfer Times 24
High-Intensity Field Transfers 24
Standard Field Transfers 26
Selecting Power Supply Settings 26
Transfers Under Constant Voltage 26
Transfers Under Constant Current 26
Transfers Under Constant Power 26
General Guidelines for Transfer Buffers and Transfer Conditions 26
Chapter 5 Detection and Imaging 29
Total Protein Detection 31
Anionic Dyes 31
Fluorescent Protein Stains 31
Stain-Free Technology 32
Colloidal Gold 32
Immunodetection 32
Immunodetection Workflow 33
Blocking 33
Antibody Incubations 33
Washes 33
Antibody Selection and Dilution 34
Primary Antibodies 34
Species-Specific Secondary Antibodies 34
Antibody-Specific Ligands 34
Detection Methods 35
Colorimetric Detection 36
Premixed and Individual Colorimetric Substrates 38
Immun-Blot® Assay Kits 38
Immun-Blot Amplified AP Kit 38
Opti-4CN™ and Amplified Opti-4CN Substrate and Detection Kits 38
Chemiluminescence Detection 38
Immun-Star™ AP Kits, Clarity™ and Clarity Max™ Western ECL Substrates 40
Fluorescence Detection 40
Other Detection Methods 41
Bioluminescence 41
Chemifluorescence 42
Autoradiography 42
Immunogold Labeling 42
Stripping and Reprobing 42
Imaging — Analysis and Documentation 43
Luminescence Detection 43
Digital Imaging for Fluorescence, Chemifluorescence, and Colorimetric Detection 44
Autoradiography 44
Analysis Software 44
Part 2 Methods 47
Protocols 48
Electrophoretic Transfers 48
Reagent and Materials Preparation 48
Tank Blotting Procedure 49
Prepare the Gel and Membrane Sandwich 49
Assemble the Tank and Program the Power Supply 50
Semi-Dry Blotting Procedure 51
Trans-Blot® Turbo™ Blotting Procedure 52
Microfiltration 53
Blot Stripping and Reprobing 54
Total Protein Detection 55
SYPRO Ruby Stain 55
Ponceau S Stain 55
Colloidal Gold Total Protein Stain 55
Immunodetection 56
Notes for Multiplex Detection 56
Notes for Chemiluminescence Detection 57
Notes for Fluorescence Detection 57
Note for Protein G-HRP Detection 57
Notes for Amplified Opti-4CN™ Detection 57
Notes for Amplified AP Detection 57
Transfer Buffer Formulations 58
Towbin Buffer 58
Towbin Buffer with SDS 58
Bjerrum Schafer-Nielsen Buffer 58
Bjerrum Schafer-Nielsen Buffer with SDS 58
CAPS Buffer 58
Dunn Carbonate Buffer 58
0.7% Acetic Acid 58
Detection Buffer Formulations 58
General Detection Buffers 58
Total Protein Staining Buffers and Solutions 59
Substrate Buffers and Solutions 60
Stripping Buffer 60
Part 3 Troubleshooting 63
Transfer 64
Electrophoretic Transfer 64
Microfiltration 65
Detection 66
Immunodetection 66
Multiscreen Apparatus 68
Total Protein Detection 68
Appendix 70
Protein Standards for Blotting 70
Unstained Standards for Protein Blotting 71
Precision Plus Protein Unstained Standards 71
Prestained Standards for Western Blotting 72
Precision Plus Protein Prestained Standards 72
Precision Plus Protein™ WesternC™ Standards 73
Glossary 74
References and Related Reading 78
Ordering Information 80

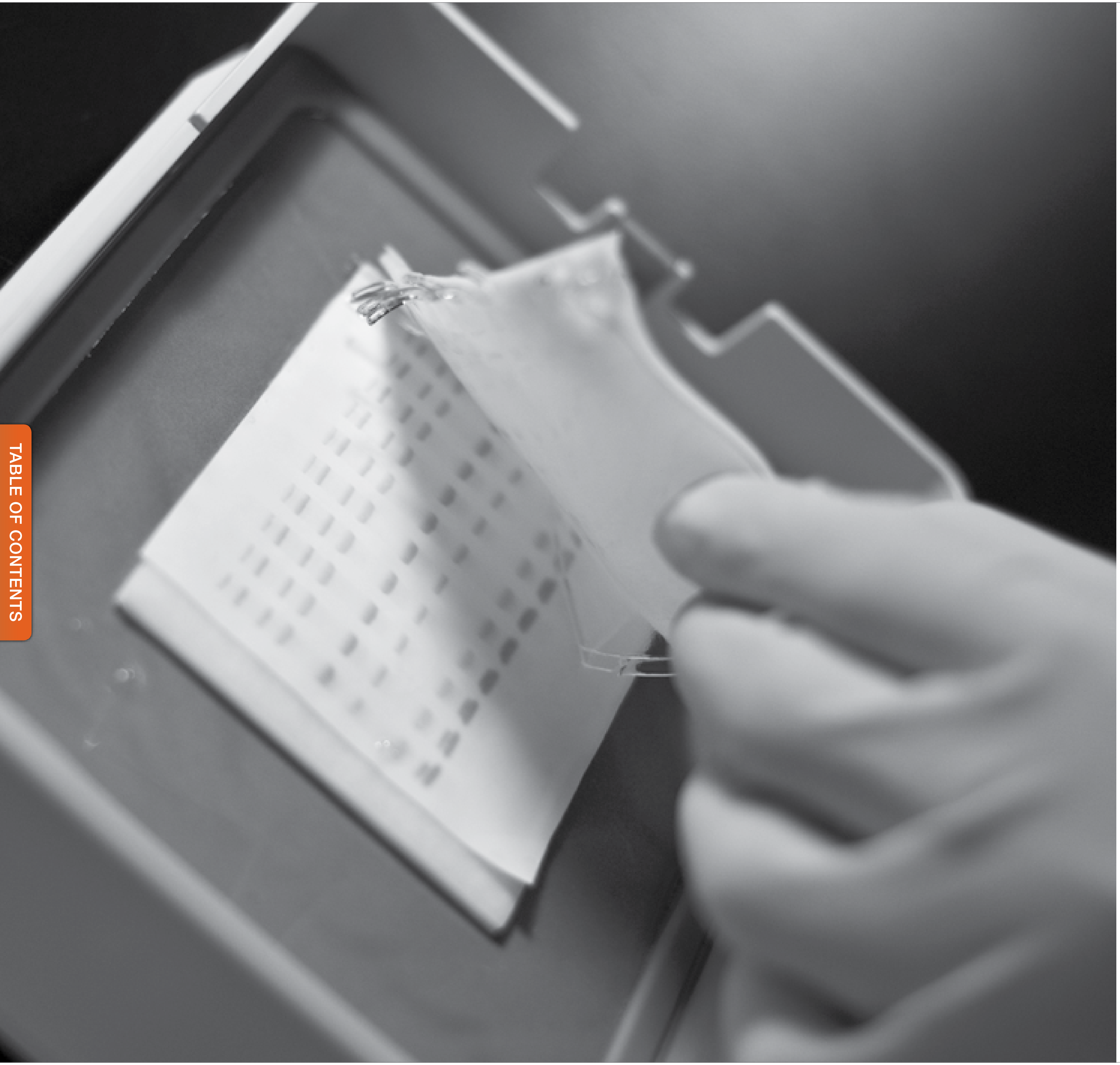

TABLE OF CONTENTS

PART 1
Theory and Products

CHAPTER 1
Overview of Protein Blotting

Protein blotting, the transfer of proteins to solid-phase membrane supports, is a powerful and popular technique for the visualization and identification of proteins. When bound to membranes, proteins are readily accessible for immunological or biochemical analyses, quantitative staining, or demonstration of protein-protein or protein-ligand interactions. This chapter provides an overview of the methods and workflow of protein blotting, which involves two phases: transfer and detection.

TABLE OF CONTENTS

Transfer

The first phase of protein blotting is the transfer step, which involves moving the proteins from a solution or gel and immobilizing them on a synthetic membrane support (blot). Proteins can be transferred to membranes using a number of methods but the most common are electrophoretic transfer and microfiltration (dot blotting). Though diffusion or capillary blotting methods may also be used to transfer proteins from gels, generally electrophoretic transfer is used to transfer proteins following electrophoretic separation by native or SDS-polyacrylamide gel electrophoresis (SDS-PAGE) and microfiltration is used to transfer proteins that are in solution.

Detection

The second phase, detection, entails probing the membrane with either a total protein stain or primary antibody specific to the protein of interest and subsequent visualization of the labeled proteins. This involves a number of steps, including the selection of the appropriate method, reagents, and imaging equipment.

The most commonly used protein blotting technique, western blotting (immunoblotting), was developed as a result of the need to probe for proteins that were inaccessible to antibodies while in polyacrylamide gels. Western blotting involves the transfer of proteins that have been separated by gel electrophoresis onto a membrane, followed by immunological detection of these proteins. Western blotting combines the resolution of gel electrophoresis with the specificity of immunoassays, allowing individual proteins in mixtures to be identified and analyzed.

Since the development of immunoblotting techniques, other probing and detection techniques have been developed for functional protein characterization (for a review, see Kurien and Scofield 2003). This manual summarizes the most commonly used techniques, provides information about the wide selection of blotting apparatus and detection reagents available from Bio-Rad, and offers troubleshooting tips and technical advice.

General Considerations and Workflow

The protein blotting workflow involves selection of the appropriate method, apparatus, membrane, buffer, and transfer conditions. Once proteins are immobilized on a membrane, they are available for visualization, detection, and analysis.

Protein Blotting Workflow

Select the method

Method selection depends largely on the starting sample (liquid protein sample or gel)

↓

Select the equipment

Consider the experimental approach, sample format, and desired resolution and throughput

↓

Prepare the reagent

Selecting the appropriate membrane and transfer buffer is critical to successful protein transfer. Consider the size and charge of the proteins, the transfer method, and the binding properties of the membrane

↓

Perform the transfer

Set up the transfer apparatus. For electrophoretic transfer, select the transfer conditions; use the highest electric field strength (V/cm) possible within the heat dissipation capabilities of the system

↓

Detect and image the protein

The choice of staining or detection technique is determined by sensitivity requirements and the imaging equipment available

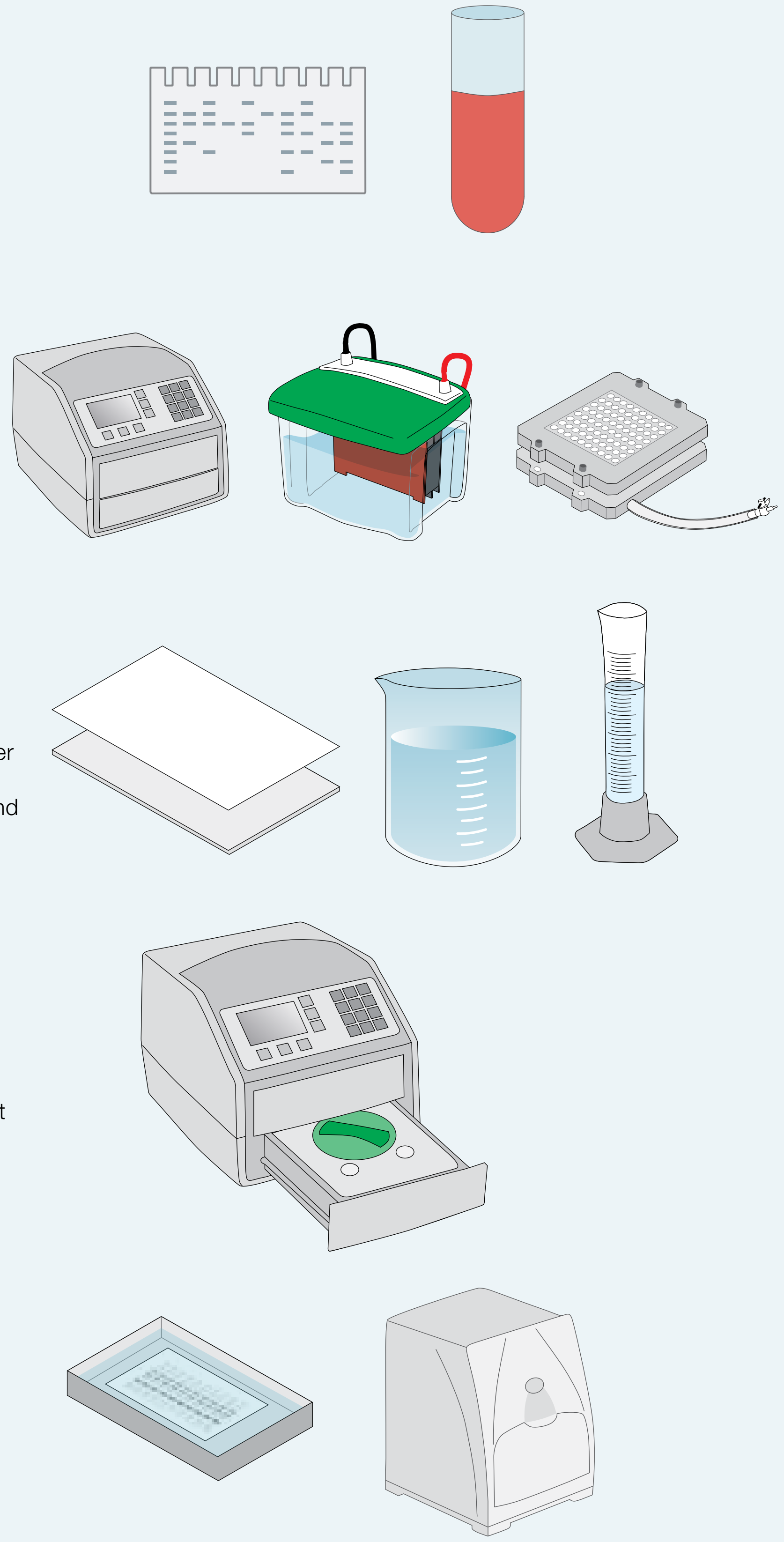

TABLE OF CONTENTS

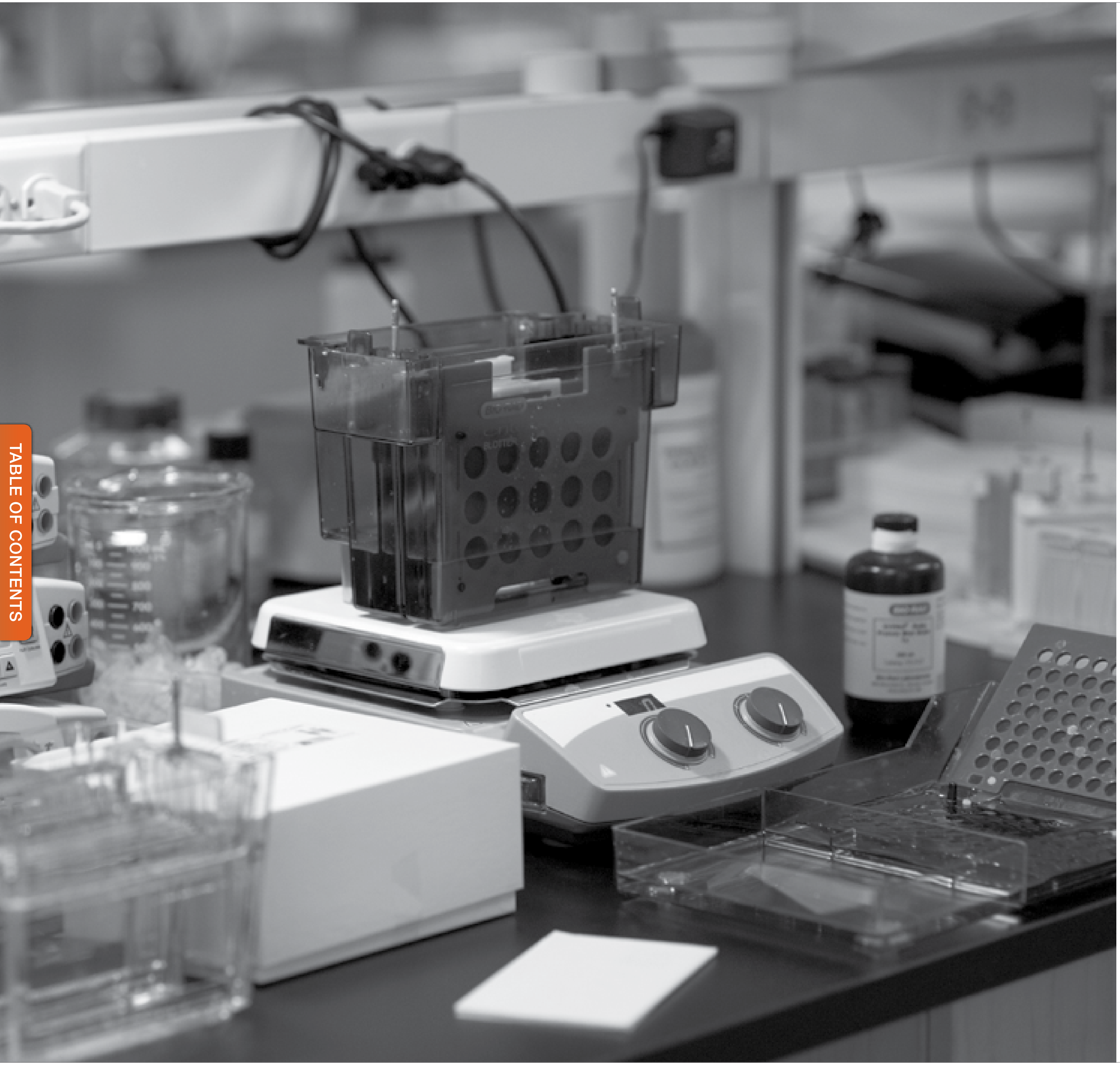

CHAPTER 2

Methods and Instrumentation

The initial step in any blotting experiment is the selection of transfer method and appropriate transfer instrumentation. Method selection depends largely on the starting sample (liquid protein sample or gel); the instrumentation depends on the sample format and desired resolution and throughput. This chapter describes a number of the most common techniques and systems used today.

TABLE OF CONTENTS

Protein Blotting Methods

The two most common methods for protein transfer are (Fig. 2.1):

- Electrophoretic transfer — for proteins already separated in gels (for example, following polyacrylamide gel electrophoresis, or PAGE), electrophoretic transfer preserves the high-resolution separation of proteins by PAGE
- Microfiltration — for proteins in solution, microfiltration is fast and useful for determining working conditions for a new blotting assay or any other situation where the resolving power of gel electrophoresis is not needed

Electrophoretic Transfer

In electrophoretic transfer, an electric field is used to elute proteins from gels and transfer them to membranes. Electrophoretic transfer is the most widely used blotting method because of its speed and precision in replicating the pattern of separated proteins from a gel to a membrane.

In an electrophoretic transfer, the membrane and protein-containing gel are placed together, with filter paper between two electrodes (Figure 2.2). Proteins migrate to the membrane following a current (I) that is generated by applying a voltage (V) across the electrodes* following Ohm's law:

$$V = I \times R$$

where R is the resistance generated by the materials placed between the electrodes (that is, the transfer buffer, gel, membrane, and filter papers).

The electric field strength (E, measured in V/cm) that is generated between the electrodes is the driving force for transfer. Both the applied voltage and the distance between the electrodes then play a major role in governing the rate of elution of the proteins from the gel. A number of other factors, including the size, shape, and charge of the protein* and the pH, viscosity, and ionic strength of the transfer buffer, as well as gel composition also influence the elution of particular proteins from gels.

There are practical limits on field strength, however, due to the production of heat during transfer. The heat generated (Joule heating) is proportional to the power consumed by the electrical elements (P), which is equal to the product of the current (I) and voltage (V):

$$P = I \times V = I^2 \times R$$

Joule heating increases temperature and decreases resistance of the transfer buffer. Such changes in resistance may lead to inconsistent field strength and transfer or may cause the transfer buffer to lose its buffering capacity. In addition, excessive heat may cause the gel to deteriorate and stick to the membrane. The major limitation of any electrophoretic transfer method is the ability of the chamber to dissipate heat.

There are two main types of electrophoretic blotting apparatus and transfer procedures (Table 2.1):

- Tank transfer systems — gels and membranes are submerged under transfer buffer in tanks; these systems are useful for most routine protein work, for efficient and quantitative protein transfers, and for transfers of proteins of all sizes. Tank transfer systems offer the most flexibility in choosing voltage settings, blotting times, and cooling options
- Semi-dry systems — gels and membranes are sandwiched between buffer-wetted filter papers that are in direct contact with flat-plate electrodes; these systems are typically easier to set up than tank systems and are useful when high-throughput is necessary and extended transfer times are not required or when discontinuous buffer systems are used. Active cooling options are limited with semi-dry blotting

Tank Blotting

In tank blotting systems, the gel and membrane sandwich is entirely submerged under transfer buffer within a buffer tank. A nonconducting cassette holds the membrane in close contact with the gel and the cassette assembly is placed in the tank between the electrodes, transverse to the electrical field and submerged under conducting transfer buffer (Burnette 1981, Gershoni et al. 1985, Towbin et al. 1979). Although the large volumes of buffer in the tank dissipate the heat generated during transfer and provide the conducting capacity for extended transfer conditions, additional cooling mechanisms are offered by the various tank blotter systems.

Semi-Dry Blotting

In a semi-dry transfer, the gel and membrane are sandwiched between two stacks of filter paper that are in direct contact with plate electrodes (Bjerrum and Schafer-Nielsen 1986, Kyhse-Andersen 1984, Tovey and Baldo 1987). The term "semi-dry" refers to the limited amount of buffer, which is confined to the two stacks of filter paper.

In semi-dry systems, the distance between the electrodes is limited only by the thickness of the gel and membrane sandwich. As a result, high electric field strengths and high-intensity blotting conditions are achieved. Under semi-dry conditions, some small proteins may be driven through the membrane in response to the high field strengths. Moreover, because low buffer capacity limits run times, some large proteins may be poorly transferred. Use of a discontinuous buffer system (see Chapter 3) may enhance semi-dry transfer of high molecular weight proteins (>80 kD). As semi-dry transfers require considerably less buffer and are easier to set up than the tank method, laboratories performing large numbers of blots often favor them.

Novel buffer and material formulations have been developed that can be used with higher electric field strengths than those used in typical semi-dry blotting. These conditions yield complete and extremely rapid transfer, with some systems completing transfer in 3–10 min. Such rapid blotting systems do not incorporate external cooling mechanisms, so the high power dissipation may generate more heat than other techniques. Rapid blotting systems are intended for extremely rapid transfers where heat-induced protein denaturation will not affect downstream applications.

Microfiltration (Dot Blotting)

Simple bulk transfer of proteins that are in solution may be achieved by manual application (dotting) to a membrane from a pipet or syringe, or by vacuum-assisted microfiltration. Manual dot-blotting with a pipet or syringe is generally used for small sample volumes. Microfiltration devices, on the other hand, enable application of larger volumes, multiple assays with different probes, and quick, reproducible screening of a large number of samples. Microfiltration facilitates the determination of working conditions for a new blotting assay and is a convenient method in any other situation where the resolving power of gel electrophoresis is not needed.

* Proteins denatured with sodium dedecyl sulfate (SDS) carry a net negative charge and migrate toward the anode.

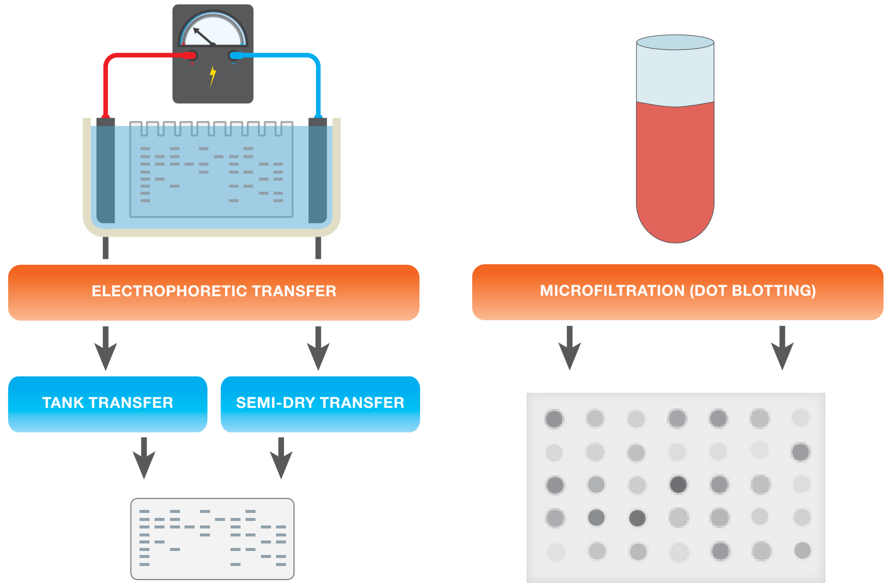

Fig. 2.1. Protein transfer methods.

Table 2.1. Comparison of electrophoretic protein transfer systems.

	Tank Blotting	Semi-Dry Blotting	
		Traditional	Rapid
**Transfer time**	30 min–overnight	15–60 min	3–10 min
**Handling convenience**	Manual assembly of transfer components	Manual assembly of transfer components	Prepackaged, presaturated components
**Transfer parameters**	Widest range of power settings and transfer times	Power and transfer time limited due to lack of cooling options	Preinstalled, customizable programs for transfers of most proteins, user-programmable settings for traditional semi-dry techniques
**Molecular weight range**	Broad range	Best for 30–120 kD	Broad range
**Temperature control**	Cooling with ice pack or refrigerated water recirculator	None	None
**Buffer requirement**	1–12 L, system-dependent	250 ml per blot	No additional buffer required

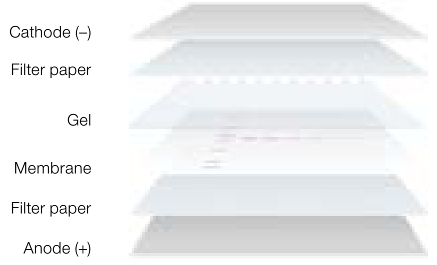

Fig. 2.2. Gel and membrane setup for electrophoretic transfer.

Links

Mini-PROTEAN® Tetra Cell

Mini-PROTEAN® TGX™ Gels

Gel Doc™ EZ Imager

TABLE OF CONTENTS

Blotting Systems and Power Supplies

Once the transfer method has been selected, choose the appropriate transfer cell or apparatus for that application.

Tank Blotting Cells

The tank transfer systems offered by Bio-Rad are described below, and their specifications are summarized in Table 2.2. Selection of the appropriate system is largely dictated by the gel format used for separation and the desired throughput.

Tank transfer systems contain the following elements:

- Buffer tank and lid — the buffer tank and lid combine to fully enclose the inner chamber during electrophoresis. On the inside, the tank has slots for placement of the electrode cards, gel holder cassettes, and cooling element. Ports on the lid allow connection points for the electrodes and are energized using an external power supply
- Gel holder cassette — the gel and membrane sandwich is held together between two foam pads and filter paper sheets, and placed into the tank within a gel holder cassette. Cassettes are made of nonconducting material and are designed to permit unimpeded flow of current and buffer through the gel and membrane sandwich
- Electrodes — tank transfer systems use either plate or wire electrode cards. Plate electrodes offer greater field strength than wire electrodes but wire electrodes may be more economical and generate less heat
- Cooling mechanism — cooling systems consist of an in ice block, a sealed ice unit, or a cooling coil that is coupled to an external cooling mechanism. These cooling systems prevent temperature fluctuations and overheating during high-intensity, extended, or native protein transfers

Mini Trans-Blot® Cell and Criterion™ Blotter

The Mini Trans-Blot cell and the Criterion blotter accommodate mini- and midi-format gels. The Mini Trans-Blot cell (Figure 2.3) can transfer up to two mini gels (10 x 7.5 cm) in an hour and is available either as a complete apparatus or as a module that uses the buffer tank and lid of the Mini-PROTEAN® Tetra cell for operation. The Criterion blotter (Figure 2.4) can transfer up to two Criterion gels (15 x 9.4 cm) or four mini gels in 30–60 min. A self-contained Bio-Ice™ cooling unit absorbs the heat generated during transfer in the Mini Trans-Blot cell, and the Criterion blotter uses a sealed ice block or optional cooling coil to regulate temperature during transfer.

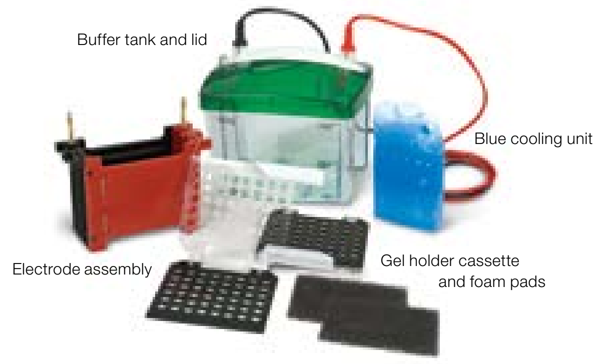

Fig. 2.3. Mini Trans-Blot cell.

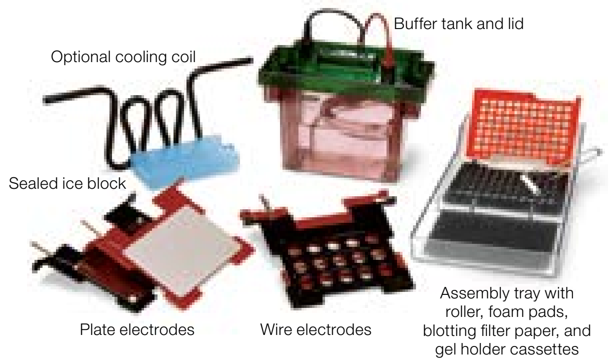

Fig. 2.4. Criterion blotter.

Trans-Blot® Cell

The Trans-Blot cell (Figure 2.5) offers a choice of plate or wire electrodes and variable placement of the electrodes for both standard and high-intensity blotting options. The Trans-Blot cell accommodates three gel holder cassettes, each with a 16 x 20 cm blotting area. Use this system for transfer of large-format gels or of multiple mini- or midi format gels. Standard field transfers are performed with the electrodes placed 8 cm apart; with this arrangement, all three of the gel holder cassettes can be used simultaneously. High-intensity transfers are performed with the electrodes placed 4 cm apart, with a single gel holder cassette between them. Temperature regulation can be achieved using the super cooling coil (included) and a refrigerated water recirculator (purchased separately).

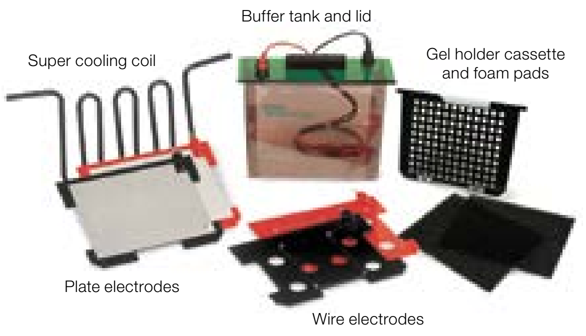

Fig. 2.5. Trans-Blot cell.

Table 2.2. Specifications for Bio-Rad's tank blotting cells.

	Mini Trans-Blot®	Criterion™ Blotter	Trans-Blot®	Trans-Blot® Plus
**Blotting area**	10 x 7.5 cm	15 x 9.4 cm	16 x 20 cm	28 x 26.5 cm
**Gel capacity**	2 Mini-PROTEAN® gels	4 Mini-PROTEAN or 2 Criterion™ gels	3 PROTEAN® II xi, 6 Criterion, or 12 Mini-PROTEAN gels	Three 26.5 x 28 cm gels or 12 Criterion gels
**Number of gel holders**	2	2	3	3
**Buffer requirement**	1.2 L	1.3 L	3–4 L	10–12 L
**Electrode distance**	4 cm	4.3 cm	2 positions: 4 and 8 cm	3 positions: 4, 7, and 10 cm
**Electrode materials**	Platinum wire	Platinum-coated titanium anode with stainless-steel cathode plates or platinum wire	Platinum-coated titanium anode with stainless-steel cathode plates or platinum wire	Platinum-coated titanium anode and stainless-steel cathode plates
**Transfer time**				
**Wire electrodes**	Standard: 16 hr<br>High-intensity: 1 hr	Standard: 60 min to overnight	Standard: 5 hr<br>Overnight: 16 hr<br>High-intensity: 30 min–4 hr	
**Plate electrodes**		Standard: 30 min to overnight	Standard: 1–5 hr<br>Overnight: 16 hr<br>High-intensity: 30 min–1 hr	Standard: 16 hr<br>High-intensity: 15 min–1 hr
**Cooling**	Blue cooling unit	Sealed ice block or optional Criterion blotter cooling unit	Super cooling coil	Super cooling coil
**Overall dimensions (W x L x H)**	12 x 16 x 18 cm	21.8 x 11.8 x 15 cm	18 x 9.5 x 24 cm	30 x 17.3 x 39.4 cm

Trans-Blot® Plus Cell

With a 28 x 26.5 cm blotting area, the Trans-Blot Plus cell (Figure 2.6) has the capacity to transfer three large-format gels or multiple smaller format gels simultaneously in as little as 15–30 min. Plate electrodes provide a strong and uniform electrical field and are movable — up to three gel cassettes can be placed in the tank with the minimum electrode distance between them, increasing the field strength and efficiency of transfer. A cooling coil coupled to a refrigerated water recirculator provides temperature regulation.

Semi-Dry Blotting Cells

Semi-dry transfers allow fast, efficient, economical blotting without a buffer tank or gel cassettes. Semi-dry systems do not offer external cooling. See Table 2.3 for detailed specifications.

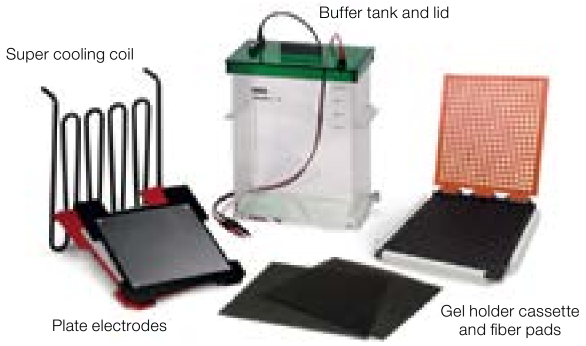

Fig. 2.6. Trans-Blot Plus cell.

Table 2.3. Specifications for Bio-Rad's semi-dry blotting cells.

	Trans-Blot® SD	Trans-Blot® Turbo™
**Blotting area**	24 x 16 cm	15 x 11 cm
**Gel capacity**	2 PROTEAN II gel sandwiches, stacked and separated by dialysis membrane; 4 Mini-PROTEAN gels side by side; 3 Criterion gels side by side	2 midi gels (13.5 x 8.5 cm), 4 mini gels (7 x 8.5 cm) or similar
**Transfer time**	~30 min	3–10 min
**Buffer requirement**	200 ml	N/A
**Electrode dimensions**	25 x 18 cm	16 x 12 cm
**Electrode distance**	Determined by thickness of the gel and membrane sandwich and filter paper stack	~8 mm depending on gel thickness
**Electrode materials**	Platinum-coated titanium anode and stainless-steel cathode	Platinum-coated titanium anode and stainless-steel cathode
**Cooling**	N/A	N/A
**Overall dimensions (W x L x H)**	37 x 24 x 11 cm	26 x 21 x 20 cm

Links

Mini-PROTEAN Tetra Cell

Mini-PROTEAN® TGX™ Gels

Mini Trans-Blot Cell

Criterion Blotter

Trans-Blot Cell

Trans-Blot Plus Cell

Trans-Blot SD
Semi-Dry System

Trans-Blot Turbo System

TABLE OF CONTENTS

Trans-Blot® SD Semi-Dry Cell

The Trans-Blot SD semi-dry cell (Figure 2.7) performs electrophoretic transfers in less than 30 min. Plate electrodes and a single-step locking system make assembly easy and ensure uniform contact across the entire electrode surface.

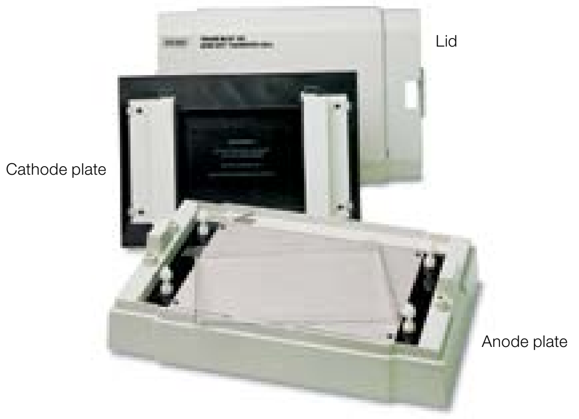

Fig. 2.7. Trans-Blot SD cell.

Trans-Blot® Turbo™ System

The Trans-Blot Turbo system (Figure 2.8) performs semi-dry transfers in as little as 3 min. The system uses prepackaged transfer packs containing a prewet membrane (nitrocellulose or polyvinylidene difluoride [PVDF]) and filter paper stacks soaked with a proprietary buffer. The base unit contains an integrated power supply that drives two independent transfer cassettes, allowing transfer of a total of four mini-format or two midi-format gels.

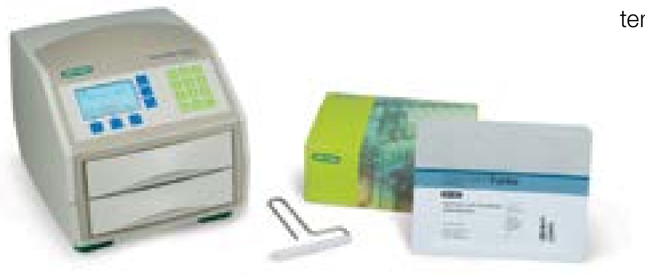

Fig. 2.8. Trans-Blot Turbo system.

Microfiltration Apparatus

Microfiltration units use easy, reproducible methods for binding proteins in solution onto membranes.

Bio-Dot® and Bio-Dot SF Apparatus

The Bio-Dot and the Bio-Dot SF (slot-format) microfiltration units (Figure 2.9) provide reproducible binding of proteins in solution onto membranes.

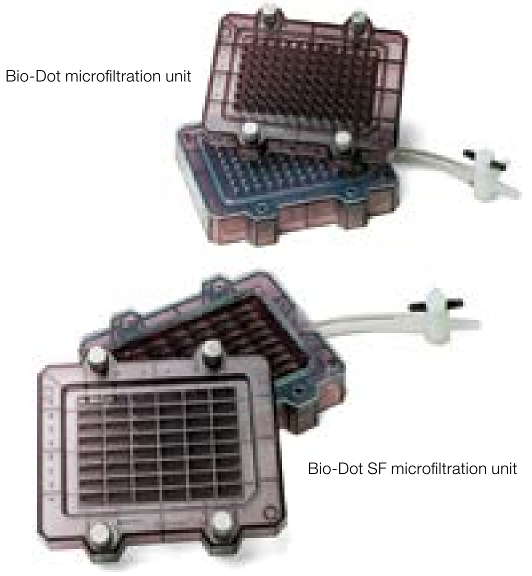

Fig. 2.9. Microfiltration apparatus.

Samples are loaded into the wells of the templates and proteins are trapped on the membrane by filtration using either vacuum or gravity flow. Once samples are loaded, incubations, wash steps, and detection may all be performed without removing the membrane from the unit.

The 96-well Bio-Dot apparatus performs traditional dot-blot comparisons and the 48-well Bio-Dot SF apparatus focuses the applied samples into thin lines instead of circles (Figure 2.10). The slot format makes it easier to use a densitometer for quantitation. The Bio-Dot and Bio-Dot SF sample templates are interchangeable; each uses the same microfiltration manifold. Each apparatus is available as an independent unit containing both the microfiltration manifold and the sample template, and also as a modular template without the manifold base.

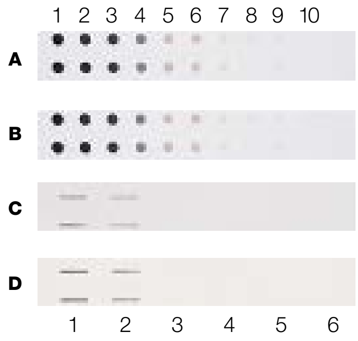

Fig. 2.10. Multiple sample comparisons are simplified with the Bio-Dot and Bio-Dot SF microfiltration units. A and B, antigen (human transferrin) applied to nitrocellulose in each row of the Bio-Dot apparatus. 1, 100 ng; 2, 50 ng; 3, 25 ng; 4, 10 ng; 5, 5 ng; 6, 2.5 ng; 7, 1 ng; 8, 0.5 ng; 9, 0.25 ng; 10, 1% BSA in TBS. C and D, antigen applied to each row of the Bio-Dot SF apparatus. 1, 100 ng; 2, 50 ng; 3, 10 ng; 4, 5 ng; 5, 1 ng; 6, 0.1 ng. The membranes were incubated with rabbit anti-human transferrin. In A and C, Bio-Rad's goat anti-rabbit gold conjugate and gold enhancement kit were used to visualize the antigen. In B and D, Bio-Rad's goat anti-rabbit AP conjugate and the color development reagents BCIP and NBT were used to visualize the antigen.

The Bio-Dot® and Bio-Dot SF units can be easily sterilized by autoclaving or by washing in alcohol or sodium hydroxide. The units feature a unique, patented sealing gasket that eliminates lateral leakage and possible cross-contamination among wells. Both sample templates are spaced similarly to microplates, so samples can be applied with a standard or a multichannel pipet. Specifications for the Bio-Dot units are listed in Table 2.4.

Table 2.4. Bio-Dot apparatus specifications.

	Bio-Dot	Bio-Dot SF
**Sample format**	96-well, 8 x 12 format	48-slot, 6 x 8 format
**Well size**	3 mm diameter	7 x 0.75 mm
**Sample volume**	50–600 µl	50–500 µl
**Membrane size (W x L)**	9 x 12 cm	9 x 12 cm
**Autoclavability**	Yes	Yes

Power Supplies for Electrophoretic Transfers

Electrophoretic transfer cells require high currents that not all power supplies are equipped to deliver. Table 2.5 compares the two Bio-Rad power supplies that accommodate the needs of electrophoretic transfer systems.

Table 2.5. PowerPac™ HC and PowerPac Universal power supply specifications.

	PowerPac HC	PowerPac Universal
**Voltage**	5–250 V	5–500 V
**Current**	0.01–3.0 A	0.01–2.5 A
**Power**	1–300 W	1–500 W

PowerPac HC Power Supply

The PowerPac HC (high current) power supply (Figure 2.11), is capable of driving all transfer cells to their maximum performance. The PowerPac HC power supply offers high power output and the flexibility of choosing transfer under constant voltage, constant current, or constant power settings. The PowerPac HC power supply also offers highly regulated voltage settings, fine adjustment of current limits, and a convenient pause function. Safety features include overload/short circuit detection, automatic crossover, arc and ground leak detection, programmable multistep methods, and a programmable timer.

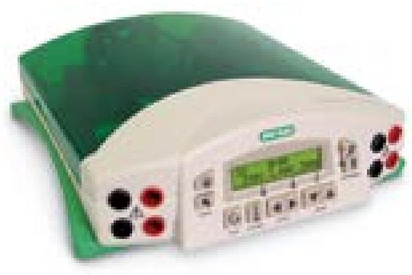

Fig. 2.11. PowerPac HC power supply.

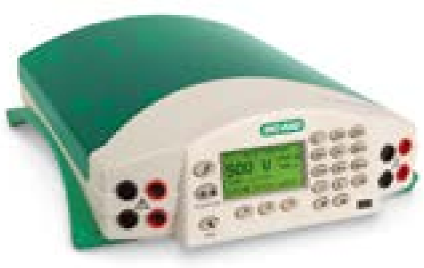

Fig. 2.12 PowerPac Universal power supply.

PowerPac™ Universal Power Supply

The PowerPac Universal power supply (Figure 2.12) is designed to drive all of the most common electrophoretic applications, with the exception of high-voltage applications such as isoelectric focusing and DNA sequencing. Like the PowerPac HC power supply, the PowerPac Universal power supply provides the choice of transfer under constant voltage, constant current, or constant power settings with all of the other features listed above. In addition, the PowerPac Universal stores up to nine methods, each with up to nine steps, and is equipped to enable wireless transfer of run data and protocols for instrument validation for regulatory purposes (for example, installation qualification and operational qualification, or IQ/OQ).

Links

Trans-Blot Turbo System

Trans-Blot Turbo Transfer Packs

Bio-Dot Microfiltration Apparatus

Trans-Blot SD Semi-Dry System

PowerPac HC Power Supply

PowerPac Universal Power Supply

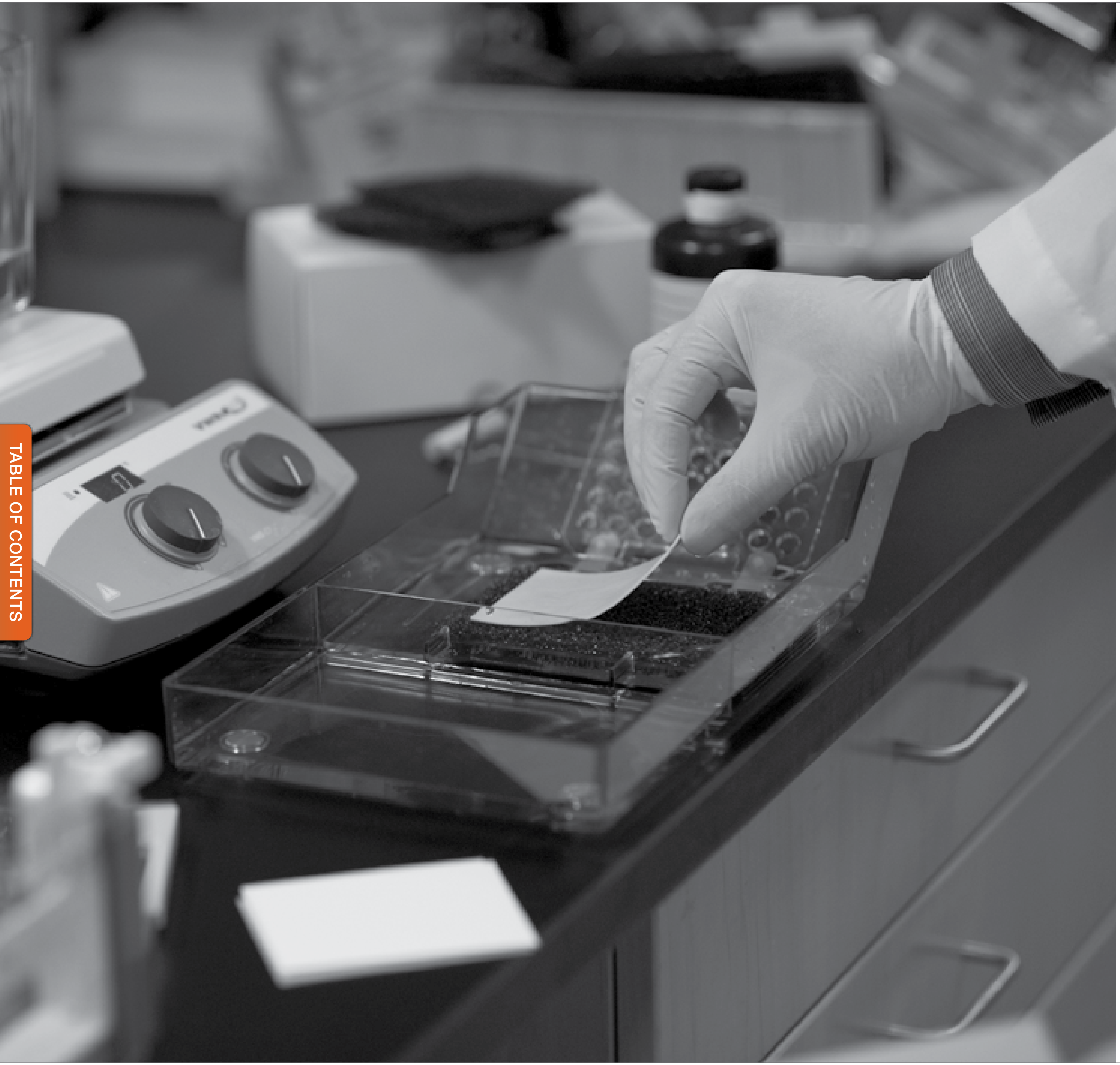

CHAPTER 3

Membranes and Transfer Buffers

Selecting the appropriate membrane and buffer is critical to successful protein transfer. The size and charge of the proteins, the transfer method, and the binding properties of the membrane all must be considered. This chapter provides technical information and advice for selecting among the various conditions that are available for protein transfer.

Membranes and Blotting Papers

A variety of membrane types is available, each offering key attributes to suit particular experimental conditions. Evaluate the physical properties and performance characteristics of a membrane when selecting a membrane for your application (Table 3.1). Membranes are commonly available in two pore sizes:

- 0.45 µm pore size membranes are recommended for most analytical blotting experiments
- 0.2 µm pore size membranes are most suitable for transfer of low molecular weight (<15,000 kD) proteins that might move through larger membrane pores

Nitrocellulose and Supported Nitrocellulose

Nitrocellulose was one of the first membranes used for western blotting and is still a popular membrane for this procedure. Protein binding to nitrocellulose is instantaneous, nearly irreversible, and quantitative to 80–100 µg/cm^2. Nitrocellulose is easily wetted in water or transfer buffer and is compatible with a wide range of protein detection systems. Unsupported nitrocellulose is innately fragile and is not recommended for stripping and reprobing.

Supported nitrocellulose is an inert support structure with nitrocellulose applied to it. The support structure gives the membrane increased strength and resilience. Supported nitrocellulose can withstand reprobing and autoclaving (121°C) and retains the ease of wetting and protein binding features of nitrocellulose.

Polyvinylidene Difluoride (PVDF)

PVDF membrane is an ideal support for N-terminal sequencing, amino acid analysis, and immunoassay of blotted proteins. PVDF retains proteins during exposure to acidic or basic conditions and in the presence of organic solvents. Greater protein retention during sequencing manipulations enhances the likelihood of obtaining information from rare, low-abundance proteins by increased initial coupling and higher repetitive yields. In addition, PVDF membrane exhibits better binding efficiency of electroblotted material in the presence of SDS in the transfer buffer.

PVDF membrane must be wetted in 100% methanol prior to use but once wet may be used with a transfer buffer that contains no methanol. Bio-Rad offers PVDF membrane specifically designed for protein sequencing and for immunodetection. Both are available in precut sheets, rolls, and sandwich formats.

Immun-Blot® and Immun-Blot LF PVDF for Western Blotting

Immun-Blot PVDF membrane retains target protein but resists nonspecific protein binding that can obscure high-sensitivity chemiluminescence and colorimetric detection. Immun-Blot PVDF has a strong binding capacity of 150–160 µg/cm^2 (roughly twice that of nitrocellulose), will not crack or tear in common handling, and can withstand repeated stripping and reprobing.

Immun-Blot LF PVDF membranes combine the advantages of Immun-Blot PVDF membranes with low autofluorescence across a wide range of excitation and emission wavelengths. This low autofluorescence allows longer exposure times without increasing background fluorescence levels, allowing fluorescent detection of faint signals.

Sequi-Blot™ PVDF for Protein Sequencing

Sequi-Blot PVDF membrane withstands the conditions of N-terminal sequencing while providing the binding capacity to sequence even low-abundance samples.

Table 3.1. Guide to protein blotting membranes.

Membrane	Pore Size	Binding Capacity (µg/cm^2)	Compatible Detection Methods	Notes
Nitrocellulose	0.45 µm<br>0.2 µm	80–100	Colorimetric<br>Chemiluminescence<br>Chemifluorescence<br>Fluorescence<br>Radioactive	General-purpose protein blotting membrane
Supported nitrocellulose	0.45 µm<br>0.2 µm	80–100	Colorimetric<br>Chemiluminescence<br>Chemifluorescence<br>Fluorescence<br>Radioactive	Pure nitrocellulose cast on an inert synthetic support; increased strength for easier handling and for reprobing
Immun-Blot PVDF	0.2 µm	150–160	Colorimetric<br>Chemiluminescence<br>Radioactive	High mechanical strength and chemical stability; recommended for western blotting
Immun-Blot LF PVDF	0.45 µm	155–300	Colorimetric<br>Chemiluminescence<br>Chemifluorescence<br>Fluorescent	High mechanical strength and chemical stability; low autofluorescence; recommended for western blotting using fluorescent detection
Sequi-Blot™ PVDF	0.2 µm	170–200	Colorimetric<br>Radioactive	High mechanical strength and chemical stability; recommended for protein sequencing

Blotting Filter Papers

Blotting filter paper, made of 100% cotton fiber, provides a uniform flow of buffer through the gel and contains no additives that might interfere with the transfer process. Precut filter paper is available in a wide range of convenient sizes to eliminate waste and save time (Table 3.2). Extra thick absorbent filter paper is recommended for semi-dry transfers because of its additional fluid capacity.

Table 3.2. Guide to precut membranes and filter paper.

Blotting Cells	Precut Membranes	Precut Blot Filter Papers
Mini Trans-Blot® cell	7 x 8.5 cm	7.5 x 10 cm
Criterion™ blotter	8.5 x 13.5 cm	9.5 x 15.2 cm
Trans-Blot® cell	13.5 x 16.5 cm	15 x 20 cm
Trans-Blot Plus cell	26.5 x 28 cm	26.5 x 28 cm
Trans-Blot SD cell	7 x 8.5 cm<br>11.5 x 16 cm<br>15 x 15 cm<br>15 x 9.2 cm<br>20 x 20 cm	15 x 15 cm<br>(extra thick)
Trans-Blot® Turbo™	7 x 8.5 cm and 8.5 x 13.5 cm<br>Transfer packs include precut membrane and filter paper	
Bio-Dot® apparatus	9 x 12 cm	N/A
Bio-Dot SF apparatus	9 x 12 cm	11.3 x 7.7 cm

Membrane/Filter Paper Sandwiches

Precut and preassembled sandwiches save time and effort during western blot preparation. In Bio-Rad's membrane sandwiches, a precut membrane (nitrocellulose or PVDF) and two sheets of 100% cotton-fiber thick filter paper are preassembled into a blotting membrane/filter paper sandwich.

Transfer Buffers

Different gel types and blotting applications call for different transfer buffers (Tables 3.3 and 3.4), but in general, transfer buffer must enable both effective elution of proteins from the gel matrix and binding of the protein to the membrane. The choice of buffer depends on the type of gel and membrane being used as well as the physical characteristics of the protein of interest.

Transfer buffers contain a conductive, strong buffering agent (for example, Tris, CAPS, or carbonate) in order to maintain the conductivity and pH of the system during transfer. In addition, alcohol (for example, methanol or ethanol) may be included in the transfer buffer to promote binding of proteins to membranes, and SDS may be added to promote elution of proteins from gels.

Regardless of the transfer buffer selected, when preparing and using transfer buffers:

- Do not use the same batch of transfer buffer more than once, as the buffer will likely lose its capacity to maintain a stable pH during transfer
- Do not dilute transfer buffers; this will also decrease buffering capacity
- Do not adjust the pH of transfer buffers when not indicated, as this increases buffer conductivity, which is manifested by higher initial current output and decreased resistance

Recipes for all of the buffers described in this section are provided in Part 2 of this guide.

A Note About SDS and Alcohol

SDS and alcohol play opposing roles in a transfer.

SDS in the gel and in the SDS-protein complexes promotes elution of the protein from the gel but inhibits binding of the protein to membranes. In cases where certain proteins are difficult to elute from the gel, SDS may be added to the transfer buffer to improve transfer. SDS in the transfer buffer decreases the binding efficiency of protein to nitrocellulose membrane; PVDF membrane can be substituted for nitrocellulose when SDS is used in the transfer buffer. Addition of SDS increases the relative current, power, and heating during transfer and may affect the antigenicity of some proteins.

Alcohol (methanol or ethanol), on the other hand, removes the SDS from SDS-protein complexes and improves the binding of protein to nitrocellulose membrane but has some negative effects on the gel itself. Alcohol may cause a reduction in pore size, precipitation of some proteins, and some basic proteins to become positively charged or neutral. All of these factors will affect blotting efficiency.

Note: Only high-quality, analytical grade methanol should be used in transfer buffer; impure methanol can increase transfer buffer conductivity and result in poor transfer.

TABLE OF CONTENTS

Links
Nitrocellulose Membrane, 0.45 µm
Nitrocellulose Membrane, 0.2 µm
Supported Nitrocellulose Membrane, 0.45 µm
Supported Nitrocellulose Membrane, 0.2 µm
Immun-Blot PVDF Membrane
Sequi-Blot PVDF Membrane

Table 3.3. General guidelines for transfer buffer and membrane selection by gel type.

Gel Type	Transfer Buffer	Membrane	Notes
SDS-PAGE	Towbin with or without SDS, CAPS, carbonate, Bjerrum Schafer-Nielsen	Nitrocellulose, supported nitrocellulose, or PVDF (0.45 or 0.2 µm)	Tank blotting or semi-dry blotting
Tris-Tricine	Towbin, CAPS	Nitrocellulose, supported nitrocellulose, or PVDF (0.2 µm)	Tank blotting recommended; needs high-capacity, small pore-size membrane; pH of buffer may be critical
Two-dimensional	Towbin with or without SDS, CAPS, carbonate, Bjerrum Schafer-Nielsen	Nitrocellulose, supported nitrocellulose, or PVDF (0.45 or 0.2 µm)	Tank blotting or semi-dry blotting
Native, nondenaturing	Depends on pH of gel buffer and pI of protein of interest	Nitrocellulose or PVDF (0.45 or 0.2 µm)	Tank blotting recommended; temperature regulation may be needed to maintain activity
Acid urea	0.7% acetic acid	Nitrocellulose (0.45 or 0.2 µm)	Tank blotting or semi-dry blotting; use acid-gel transfer protocol (membrane toward cathode)
Isoelectric focusing	0.7% acetic acid	Nitrocellulose, supported nitrocellulose, or PVDF (0.45 or 0.2 µm)	Tank blotting or semi-dry blotting; use acid-gel transfer protocol (membrane toward cathode)

Table 3.4. General guidelines for transfer buffer and membrane selection by application.

Application	Transfer Buffer	Membrane	Notes
Protein sequencing	Towbin*, CAPS	Nitrocellulose, 0.45 or 0.2 µm, or PVDF	Tank blotting recommended
High molecular weight proteins	Towbin with SDS	Nitrocellulose, 0.45 or 0.2 µm, or PVDF	Tank or rapid semi-dry blotting recommended; needs high-capacity, small pore-size membrane; pH of buffer may be critical
Small proteins and peptides	Towbin, CAPS	Nitrocellulose, 0.2 µm, or PVDF	Tank or rapid semi-dry blotting recommended; pH of buffer may be critical
Basic proteins (pI >9) in denaturing gels	CAPS, carbonate, Bjerrum Schafer-Nielsen	Nitrocellulose, 0.45 or 0.2 µm, or PVDF	Tank blotting, semi-dry blotting, or rapid semi-dry blotting
Basic proteins (pI >9) in native or nondenaturing gels	0.7% acetic acid	Nitrocellulose, 0.45 or 0.2 µm, or PVDF	Tank blotting recommended
Glycoproteins	Towbin with or without SDS, CAPS, carbonate, Bjerrum Schafer-Nielsen nondenaturing gels	Nitrocellulose, 0.45 or 0.2 µm, or PVDF	Tank blotting or semi-dry blotting
Proteoglycans	Towbin, Bjerrum Schafer-Nielsen	Nitrocellulose, 0.45 or 0.2 µm, or PVDF	Tank blotting or semi-dry blotting

*Towbin buffer may be used for protein sequencing but extra care must be exercised to rinse Tris and glycine from the membrane after transfer.

Towbin and Bjerrum Schafer-Nielsen Buffers (Tris/Glycine Buffers)

The most common transfers are from SDS-PAGE gels using the buffer systems originally described by Towbin (1979). Standard Towbin buffer contains 25 mM Tris, 192 mM glycine, pH 8.3, 20% (v/v) methanol and, occasionally, 0.025–0.1% (w/v) SDS.

A buffer similar in composition to the standard Towbin buffer is the Bjerrum Schafer-Nielsen buffer (48 mM Tris, 39 mM glycine, pH 9.2, 20% methanol), which was developed for use in semi-dry applications.

CAPS Buffer

CAPS-based transfer buffer (10 mM CAPS, pH 11, 10% methanol) may be preferable for transfers of high molecular weight proteins (for example, >50 kD) and in cases where the glycine component of Towbin buffer may interfere with downstream protein sequencing applications.

Discontinuous Tris-CAPS Buffer System (for Semi-Dry Transfer)

A unique feature of semi-dry blotting is the ability to use two different buffers during transfer; this is known as a discontinuous buffer system. In a semi-dry transfer, the buffer reservoirs are the filter paper on either side of the gel, which are independent (discontinuous). In a discontinuous system, methanol is included in the buffer on the membrane (anode) side of the blot assembly and SDS is used on the gel (cathode) side, taking advantage of the positive effects of each buffer component. A discontinuous buffer system using a Tris-CAPS buffer can greatly increase the efficiency of protein transfer by semi-dry blotting. This system uses 60 mM Tris, 40 mM CAPS, pH 9.6, plus 15% methanol in the filter paper on the anode side and 0.1% SDS on the cathode side. Concentrated, premixed anode and cathode buffers are available for purchase. For more information about the use of a discontinuous buffer system in semi-dry transfer, request bulletin 2134.

Dunn Carbonate Buffer

In some cases, using a carbonate buffer (10 mM $NaHCO_3$, 3 mM Na_2CO_3, pH 9.9, 20% methanol) may produce higher efficiency transfers and improve the ability of antibodies to recognize and bind to proteins. Carbonate buffer has also been recommended for the transfer of basic proteins (Garfin and Bers 1989).

Other Buffers

The mobility of proteins during electrophoretic transfer from native gels will depend on the size and pI of the protein of interest relative to the pH of the buffer used.

- If the pI of the protein is greater than the pH of the transfer buffer, the protein carries a positive charge and will migrate toward the negative electrode
- If the pI of the protein is close to the pH of the transfer buffer, the migration of the protein out of the gel is decreased. Use a more basic or acidic buffer to increase protein mobility

Proteins in native gels, as well as acidic and neutral proteins, require buffers that do not contain methanol. Gels for isoelectric focusing, native PAGE, and those containing basic proteins or acid-urea may be transferred in 0.7% acetic acid. When using acetic acid for transfer, the proteins will be positively charged, so the membrane should be placed on the cathode side of the gel.

Links

10x Tris/Glycine

10x Tris/CAPS

10x Tris/Glycine/SDS

10x Phosphate Buffered Saline

20x SSC

TABLE OF CONTENTS

TABLE OF CONTENTS

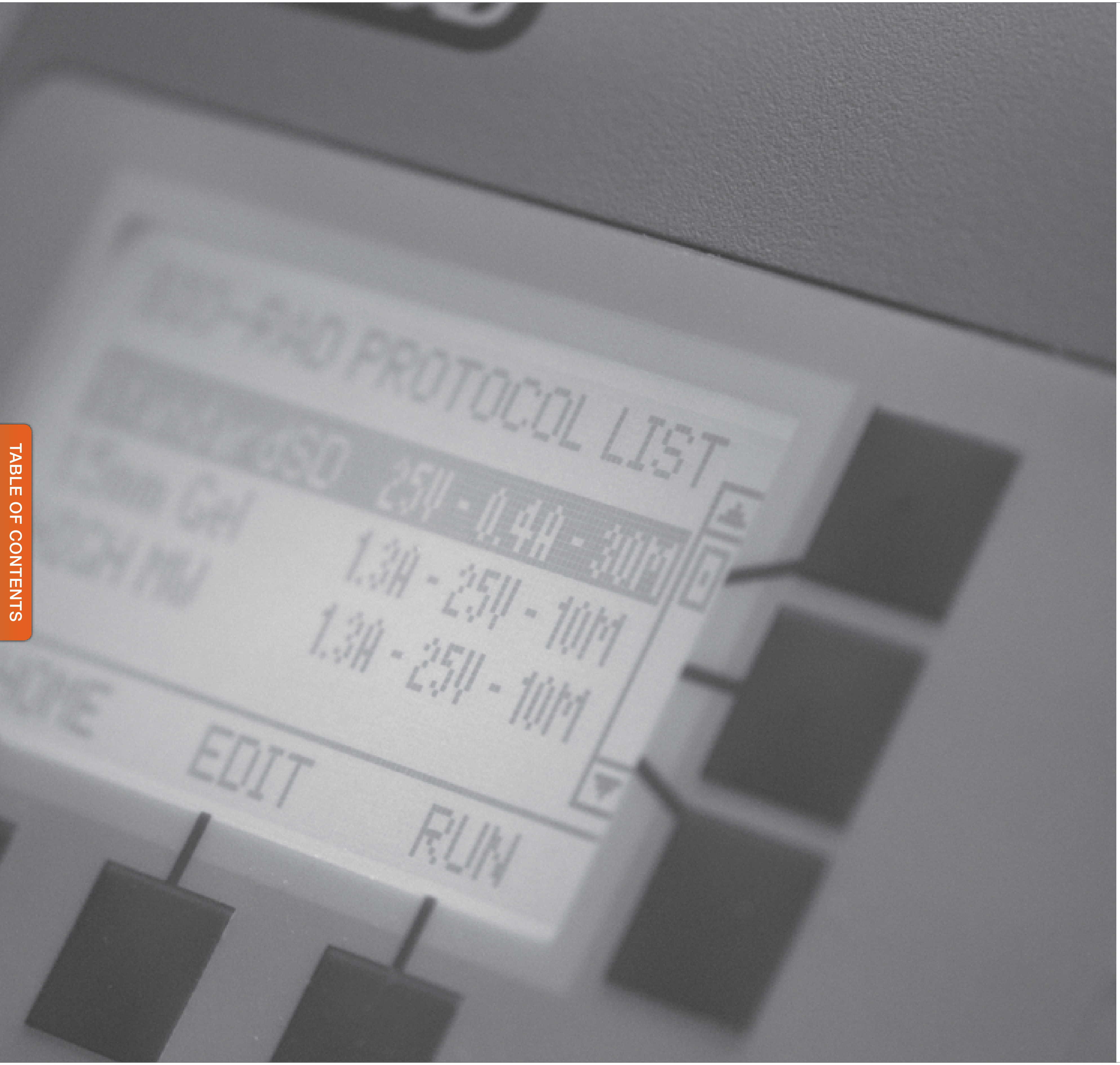

CHAPTER 4

Transfer Conditions

This chapter provides an overview of the transfer conditions required for performing electrophoretic protein transfer. Detailed protocols and advice for each transfer method are available in Part 2 of this guide.

TABLE OF CONTENTS

General Workflow — Electrophoretic Transfer

Overall, the procedures and principles for semi-dry and tank transfers are the same. Gels and membranes are prewet and equilibrated with transfer buffer, and the gel/membrane sandwich is placed into the transfer apparatus in the correct orientation to ensure transfer of proteins to the membrane. For electrophoretic transfers, the appropriate power conditions must also be selected.

Power Conditions

For best transfer results, use the highest electric field strength (E) possible within the heat dissipation capabilities of the system. For most proteins, the most rapid transfer occurs under conditions where the applied voltage (V) is maximized and the distance between the electrodes is minimized. Though rapid blotting experiments may seem to be the most convenient, a number of factors must be considered when choosing the appropriate power conditions for a given electrophoretic transfer.

Useful Equations

Two basic equations are important in electrophoresis. The first is Ohm's law, which relates the applied voltage (V) with the current (I) and resistance (R) of the system:

$$V = I \times R$$

The applied voltage and current are determined by the user and the power supply settings; the resistance is inherent in the system.

The second equation, the power equation, describes the power (P) used by a system, which is proportional to the voltage (V), current (I), and resistance (R) of the system.

$$P = I \times V = I^2 \times R = V^2/R$$

Understanding the relationships among power, voltage, current, resistance, and heat is central to understanding the factors that influence the efficiency and efficacy of transfer.

Joule Heating and Other Factors Affecting Transfer

The power that is dissipated is also equivalent to the amount of heat, known as Joule heating, generated by the system. According to the power equation, the amount of Joule heating that occurs depends on the conductivity of the transfer buffer used, the magnitude of the applied field, and the total resistance within the transfer system. During an electrophoretic transfer, the transfer buffer warms as a result of Joule heating. Consequently, its resistance drops. Such heating and changes in resistance may lead to inconsistent field strength and transfer, may cause the transfer buffer to lose its buffering capacity, or may cause the gel to melt and stick to the membrane. Under normal running conditions, the transfer buffer absorbs most of the heat that is generated; during extended transfer periods or high-power conditions, active buffer cooling is required to prevent uncontrolled temperature increases.

The following variables also change the resistance of the transfer system and, therefore, also affect transfer efficiency and current and voltage readings:

- Alterations to buffer composition; that is, addition of SDS or changes in ion concentration due to addition of acid or base to adjust the pH of a buffer
- Gel pH, ionic strength, and percentage of acrylamide, especially if the gel has not been properly equilibrated
- Number of gels (current increases slightly as the number of gels increases)
- Volume of buffer (current increases when volume increases)
- Transfer temperature (current increases when temperature increases)

Relationship Between Power Settings and Transfer Times

In theory, increasing the power input and duration of an electrophoretic transfer results in the transfer of more protein out of a gel. In practice, however, test runs should be used to evaluate transfer efficiency at various field strengths (by modulating both power input and, if applicable, interelectrode distance) and transfer times for each set of proteins of interest. The optimum transfer conditions depend on a number of factors, including the size, charge, and electrophoretic mobility of the protein, the type of transfer buffer used, and the type of transfer system being used.

High-Intensity Field Transfers

As their name suggests, high-intensity field transfers use high-strength electrical fields that are generated by increased voltage and closer positioning of electrodes. High-intensity transfers often produce satisfactory transfer of proteins in less time than standard transfers; however, in some cases the high field strength causes small proteins to be transferred through the membrane. In addition, high molecular weight proteins and other proteins that are difficult to transfer may not have sufficient time to be transferred completely. Because more heat is generated in high-intensity field transfers than in standard field transfers, a cooling device may be needed.

General Workflow for Electrophoresis Transfer

Prepare transfer buffer

Prepare transfer buffer sufficient for the transfer cell and for equilibration of gels and membranes

↓

Equilibrate gels and membranes

Equilibrate gels and membranes in transfer buffer

↓

Assemble the gel and membrane sandwich

Place the membrane and gel between buffer-soaked filter papers

↓

Set up the transfer cell

Place the gel, filter paper, and membrane sandwich in the transfer cell. Fill the cell with transfer buffer and add the cooling unit. Connect the cell to the power supply and set the power supply for optimal power and time

↓

Start the transfer

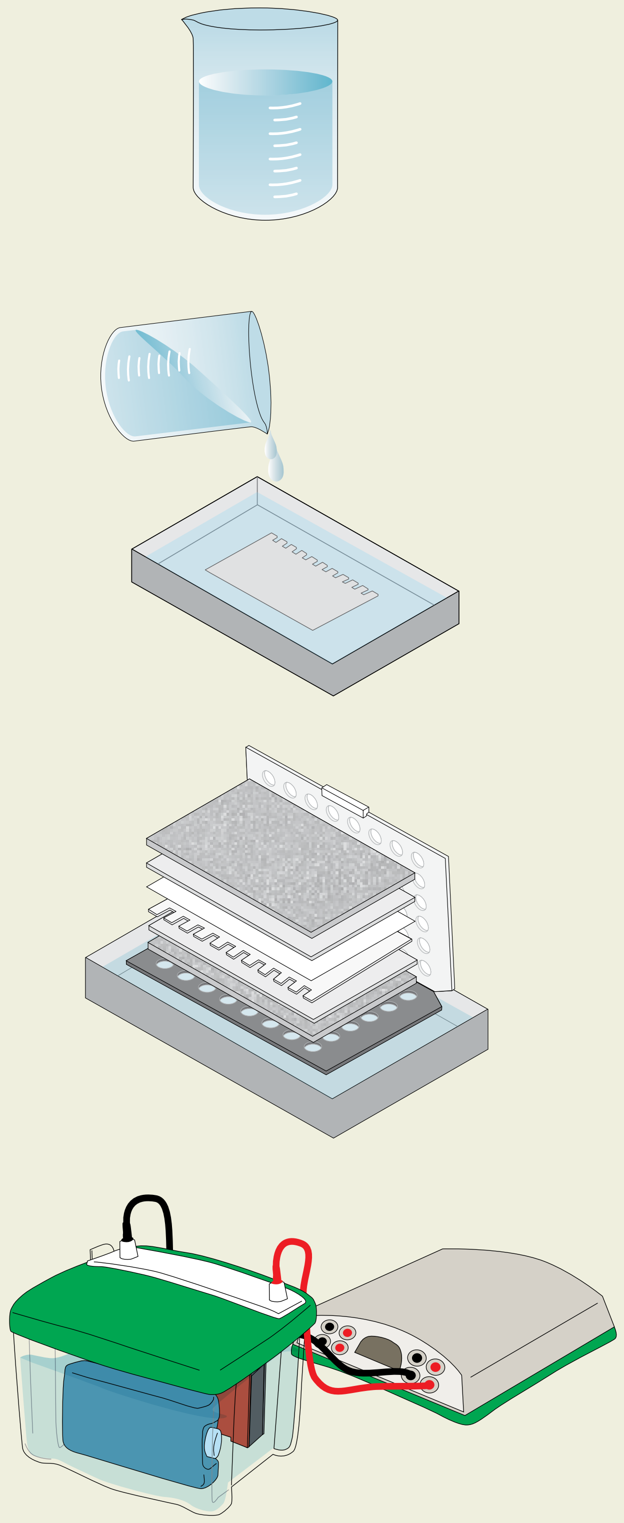

TABLE OF CONTENTS

Standard Field Transfers

Standard field transfers require less power input and more time to complete; they are generally run overnight. Standard transfers often produce more complete, quantitative transfer of proteins across a broad molecular weight range; the slower transfer conditions allow large proteins sufficient time to move through the gel matrix while the lower intensity allows smaller proteins to remain attached to the membrane after transfer.

Tank transfer systems offer the capacity for both high-intensity and standard-field transfers. Increased buffering capacity and additional cooling mechanisms enable longer transfer times than are feasible with semi-dry transfers. Some tank transfer systems offer flexible electrode positions that, when combined with variable voltages, provide a choice of high-intensity, rapid transfer or longer, more quantitative transfer over a broad range of molecular weights.

Semi-dry transfers, on the other hand, are necessarily rapid and of high intensity. In a semi-dry transfer system, the distance between electrodes is determined only by the thickness of the gel-membrane sandwich, and buffering and cooling capacity is limited to the buffer in the filter paper. As a result, the field strength is maximized in semi-dry systems, and the limited buffering and cooling capacity restricts the transfer time. Though power conditions may be varied with the power supply, semi-dry transfers often operate best within a narrow range of settings.

Selecting Power Supply Settings

Power supplies that are used for electrophoresis hold one parameter constant (either voltage, current, or power). The PowerPac™ HC and PowerPac Universal power supplies also have an automatic crossover capability that allows the power supply to switch over to a variable parameter if a set output limit is reached. This helps prevent damage to the transfer cell. During transfer, if the resistance in the system decreases as a result of Joule heating, the consequences are different and depend on which parameter is held constant.

Transfers Under Constant Voltage

If the voltage is held constant throughout a transfer, the current in most transfer systems increases as the resistance drops due to heating (the exception is most semi-dry systems, where current actually drops as a result of buffer depletion). Therefore, the overall power increases during transfer, and more heating occurs. Despite the increased risk of heating, a constant voltage ensures that field strength remains constant, providing the most efficient transfer possible for tank blotting methods. Use of the cooling elements available with the various tank blotting systems should prevent problems with heating.

Transfers Under Constant Current

If the current is held constant during a run, a decrease in resistance results in a decrease in voltage and power over time. Though heating is minimized, proteins are transferred more slowly due to decreased field strength.

Transfers Under Constant Power

If the power is held constant during a transfer, changes in resistance result in increases in current, but to a lesser degree than when voltage is held constant. Constant power is an alternative to constant current for regulating heat production during transfer.

General Guidelines for Transfer Buffers and Transfer Conditions

Different transfer apparatus, when used with different gel and buffer systems, require different power settings. Table 4.1 provides general guidelines for the voltage and current settings recommended for selected gel and buffer systems. Increase transfer times for gradient gels and decrease transfer times for low molecular weight proteins. The values presented in Table 4.1 are guidelines — transfer conditions should be optimized for every transfer application. Cooling is generally required for all high-intensity transfers (except when using the Trans-Blot® SD cell) and is recommended for long, unsupervised runs.

Table 4.1. Guide to power setttings for different gel types.

	Standard (Overnight)	High-Intensity
**SDS-PAGE Gels (Towbin Buffer)**		
Trans-Blot® cell		
Plate electrodes	10 V/100 mA, 16 hr	50–100 V/700–1,600 mA, 30–60 min
Wire electrodes	30 V/100 mA, 16 hr	100–200 V/300–800 mA, 30 min–4 hr
Trans-Blot Plus cell	30 V/0.5 A, 16 hr	100 V/1,500 mA, 60 min
Mini Trans-Blot® cell	30 V/90 mA, 16 hr	100 V/350 mA, 60 min
Criterion™ blotter		
Plate electrodes	10 V/50–80 mA, 16 hr	100 V/750–1,000 mA, 30 min
Wire electrodes	10 V/30–40 mA, 16 hr	100 V/380–500 mA, 60 min
Trans-Blot SD cell	N/A	Mini gels: 10–15 V/5.5 mA/cm², 10–30 min<br>Large gels: 15–25 V/3 mA/cm², 30–60 min
Trans-Blot® Turbo™	N/A	Mini gels: 25 V/1,300 mA, 7 min<br>Midi gels: 25 V/2,500 mA, 7 min
**Isoelectric Focusing Gels, Native Gels, Basic Proteins, and Acid-Urea Gels (0.7% acetic acid)**		
	**Standard (Overnight)**	**High-Intensity**
Trans-Blot cell		
Plate electrodes	15 V/200 mA, 16 hr	30–60 V/600–1,000 mA, 30–60 min
Wire electrodes	30 V/200 mA, 16 hr	100–150 V/550–850 mA, 30 min–4 hr
Trans-Blot Plus cell	10–30 V/0.15–0.55 A, 16 hr	100–125 V/1.9–2.4 A, 15–60 min
Mini Trans-Blot cell	30 V/10 mA, 16 hr	100 V/350 mA, 1 hr
Criterion blotter		
Plate electrodes	10 V/50 mA, 16 hr	100 V/980–1,200 mA, 30 min
Wire electrodes	10 V/50 mA, 16 hr	100 V/500–800 mA, 30 min
Trans-Blot SD cell	N/A	Mini gels: 10–15 V/5.5 mA/cm², 10–30 min<br>Large gels: 15–25 V/3 mA/cm², 30–60 min
Trans-Blot Turbo	N/A	Mini gels: 25 V/1,300 mA, 7 min<br>Midi gels: 25 V/2,500 mA, 7 min

Links
Mini Trans-Blot Cell
Criterion Blotter
Trans-Blot Cell
Trans-Blot Plus Cell
Trans-Blot SD Semi-Dry System
Trans-Blot Turbo Transfer System
PowerPac HC Power Supply
PowerPac Universal Power Supply

TABLE OF CONTENTS

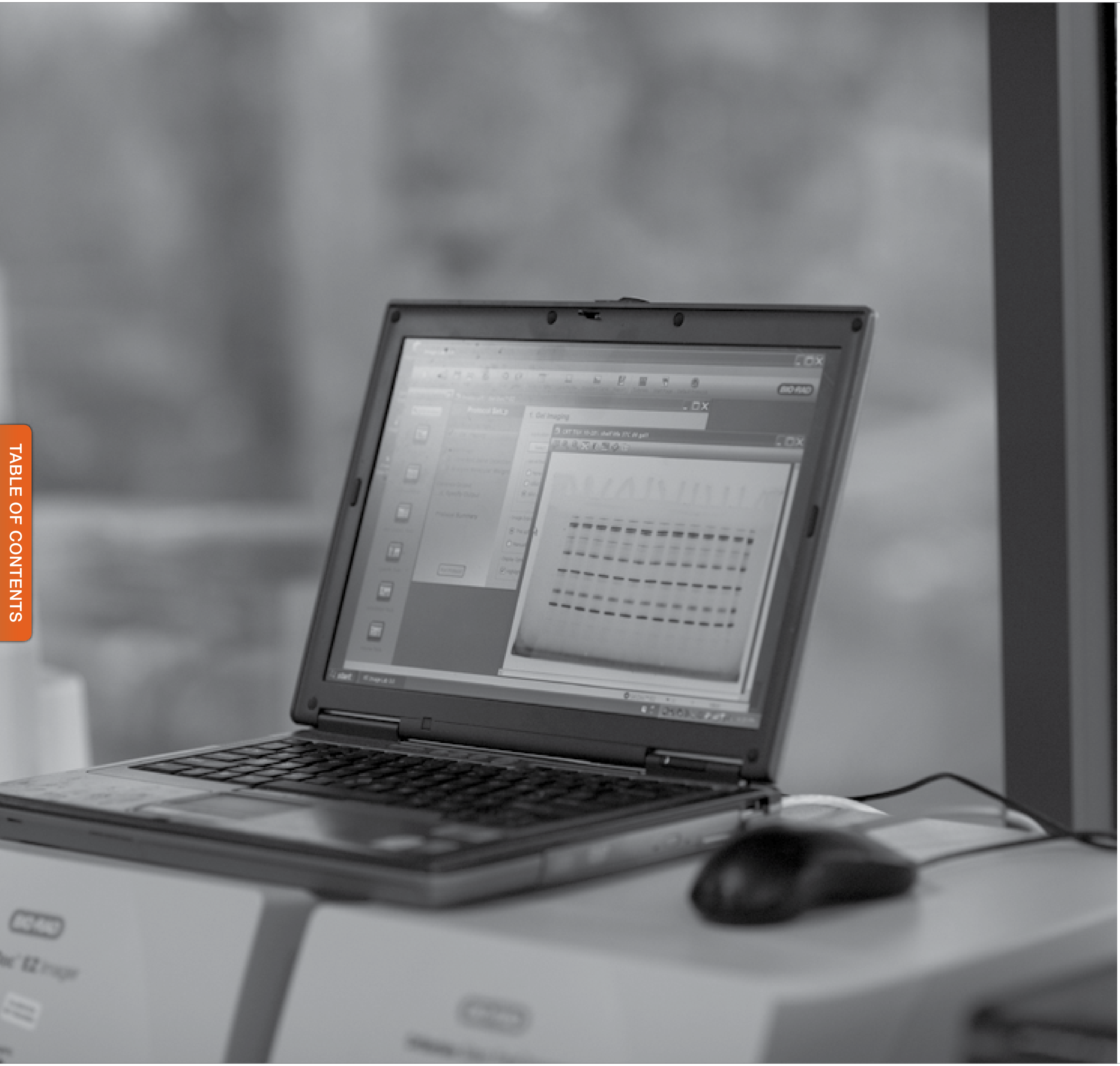

CHAPTER 5

Detection and Imaging

Total protein detection and immunodetection can be performed using colorimetric, chemiluminescence, and fluorescence development and imaging techniques.

TABLE OF CONTENTS

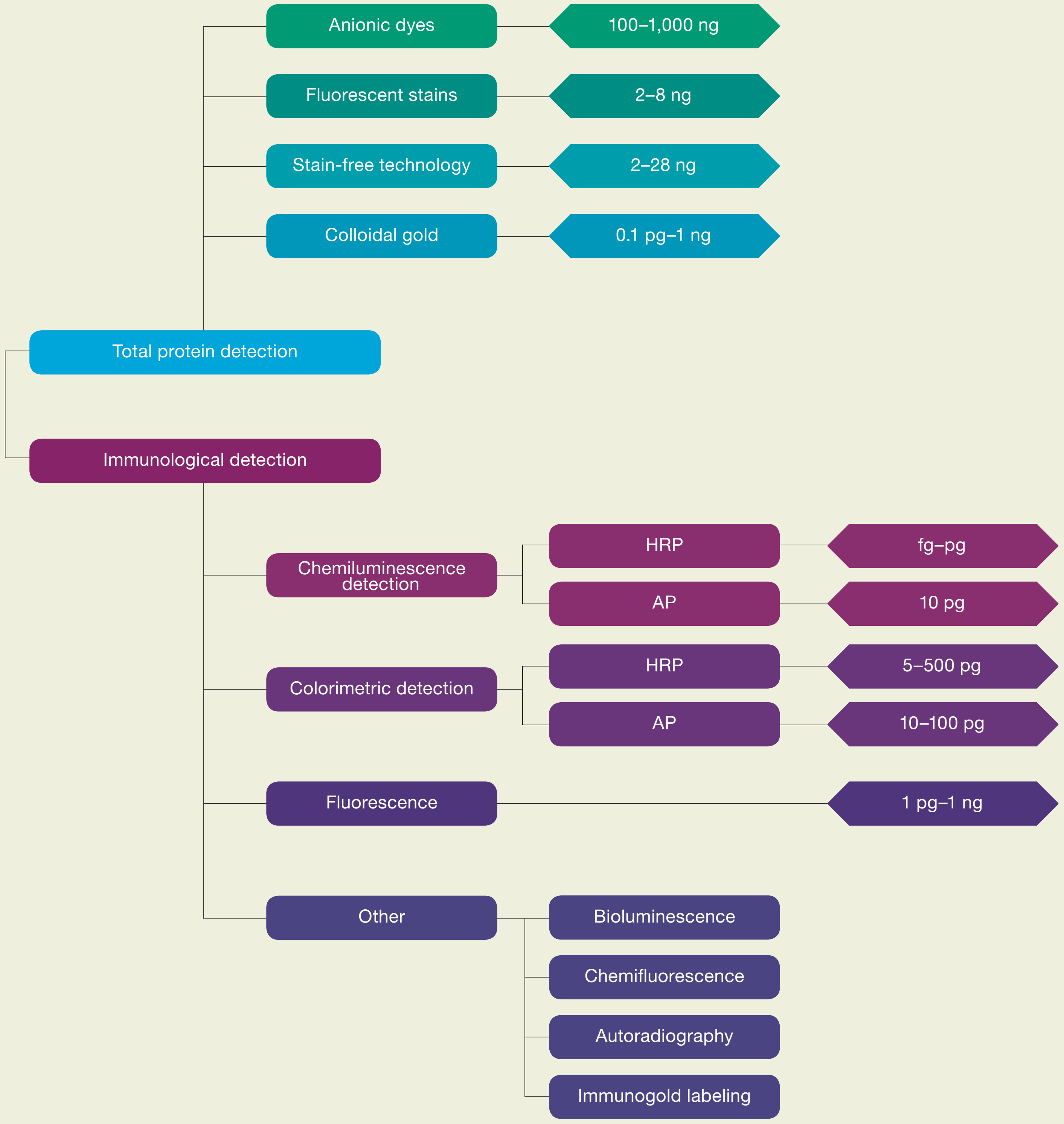

Fig. 5.1. Protein detection systems.

Once proteins have been transferred to a membrane, they can be visualized using a variety of specialized detection reagents (Figure 5.1). Total protein stains allow visualization of all the proteins on the blot while immunological detection (immunodetection) methods employ antibody or ligand conjugates for visualization of specific proteins of interest. This chapter reviews the various total protein stains and immunological detection methods available.

Total Protein Detection

Total protein staining provides an image of the complete protein pattern on the blot (Figure 5.2). This information helps determine transfer efficiency and the molecular weight, relative quantity, and other properties of the transferred proteins.

A

B

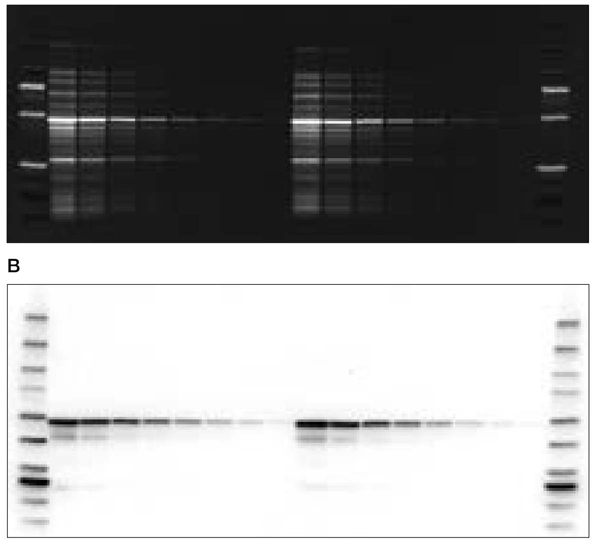

Fig. 5.2. Total protein and immunological detection. A, blot stained with SYPRO Ruby blot stain showing the total protein pattern of an *E. coli* lysate containing an overexpressed GST fusion protein on the blot. **B**, same blot probed for the GST-fusion protein in the lysate and detected using chemiluminescence.

Table 5.1 compares the advantages and disadvantages of several total protein staining techniques. When performing total protein blot staining, note that:

- Protein standards are useful for monitoring transfer efficiency and serve as molecular weight markers for calibration of blot patterns. For information about protein standards that are useful in blotting applications, refer to the Appendix in this guide
- Polyacrylamide gels shrink during staining, so comparison of an immunologically probed membrane to a stained gel is not practical. To determine the exact location of a specific antigen in relation to other proteins, compare two blotted membranes, one that has been probed with an antibody and the other stained for total protein

Table 5.1. Comparison of total protein staining methods.

Method	Sensitivity	Advantages	Disadvantages	Imaging
Anionic dyes (Ponceau S, Coomassie Brilliant Blue R-250, amido black, Fast Green FCF)	100–1,000 ng	Inexpensive, rapid	Low sensitivity, shrink membrane	Photography with epi-illumination or reflectance densitometry
Fluorescence	2–8 ng	Sensitive, mass spectrometry-compatible	Fluorescence detection system required	Fluorescence visualization with UV, LED epi-illumination, or laser scanning
Stain-free	2–28 ng	Rapid – no additional staining or destaining required	Special gels and imaging equipment required	Gel Doc™ EZ system
Colloidal gold (enhanced)	100 pg–1 ng	Very sensitive, rapid; optional enhancement increases sensitivity	Expensive	Photography with epi-illumination or reflectance densitometry

Anionic Dyes

The first techniques developed for total protein staining of blotted membranes used the same anionic dyes commonly used for staining proteins in polyacrylamide gels. These dyes include amido black (Towbin et al. 1979), Coomassie (Brilliant) Blue R-250 (Burnette 1981), Ponceau S, and Fast Green FCF (Reinhart and Malamud 1982). Of these:

Amido black destains rapidly in acetic acid/isopropanol solution and produces very little background staining. Amido black may interfere with downstream immunodetection.

Coomassie (Brilliant) Blue may show high background staining, even after long destaining procedures, and is not compatible with subsequent immunodetection.

Ponceau S and **Fast Green** are compatible with downstream immunodetection methods, and Fast Green can be easily removed after visualization to allow subsequent immunological probing.

These dyes are easy to prepare and they stain proteins quickly, but they are relatively insensitive when compared to other stains (Table 5.1). The stains that require alcohol-containing solutions for solubility (for example, amido black, Coomassie Brilliant Blue, and Fast Green FCF) can shrink nitrocellulose membranes, making direct comparison of an immunologically detected antigen to the total protein on the stained membrane difficult. Coomassie Blue R-250 stain is available from Bio-Rad.

Fluorescent Protein Stains

Fluorescent stains such as SYPRO Ruby and Deep Purple provide highly sensitive detection of proteins on blots as well as in gels. SYPRO Ruby blot stain allows detection as low as 2 ng. After staining, target proteins can be detected by colorimetric or chemiluminescence immunodetection methods, or analyzed by microsequencing or mass spectrometry with no interference from the protein stain.

Links

Coomassie Brilliant Blue R-250

SYPRO Ruby Protein Gel Stain

TABLE OF CONTENTS

Stain-Free Technology

A haloalkane compound in Mini-PROTEAN® TGX Stain-Free™ and Criterion™ TGX Stain-Free™ gels covalently binds to protein tryptophan residues when activated with UV light. This allows protein detection (with a Gel Doc™ EZ imager) in a gel both before and after transfer, as well as total protein detection on a blot when using wet PVDF membranes. Stain-free technology is compatible with downstream immunodetection, though some antibodies may show a slightly lower affinity for the haloakane-modified proteins.

Colloidal Gold

Colloidal gold is an alternative to anionic dyes that provides detection sensitivities rivaling those of immunological detection methods (Moeremans et al. 1987, Rohringer and Holden 1985). When a solution of colloidal gold particles is incubated with proteins bound to a nitrocellulose or PVDF membrane, the gold binds to the proteins through electrostatic adsorption. The resulting gold-protein complex produces a transient, reddish-pink color due to the optical properties of colloidal gold. This gold-protein interaction is the basis for total protein staining with colloidal gold as well as for specific, immunogold detection (see Immunogold Labeling on page 42).

Bio-Rad's colloidal gold total protein stain provides sensitivity to 100 pg of protein.

Immunodetection

Immunodetection (immunological detection) is used to identify specific proteins blotted to membranes. The steps used for immunological detection vary little; the steps are summarized in Figure 5.3.

After the proteins have been transferred to the membrane, the membrane is blocked, incubated with a primary antibody, washed, incubated with a secondary antibody, and washed again (Figure 5.3). The primary antibody is specific for the protein of interest and the secondary antibody enables its detection. The secondary antibody can be radiolabeled, labeled with a fluorescent compound or gold particles, or conjugated to an enzyme such as alkaline phosphatase (AP) or horseradish peroxidase (HRP) for subsequent detection.

For many years, radiolabeled secondary antibodies were the method of choice for detection, but newer methods have evolved that are less hazardous and easier to use than radioactivity, yet maintain the same degree of sensitivity. Available detection methods now also include colorimetric, chemiluminescence, fluorescence, bioluminescence, chemifluorescence, and immunogold detection.

Stain-Free Technology

Bio-Rad's stain-free technology allows direct visualization, analysis, and documentation of protein samples in PAGE gels and on blots, without staining or destaining. It also provides equal or better sensitivity compared to Coomassie staining and eliminates organic waste disposal concerns.

The stain-free system comprises the Gel Doc EZ imager with stain-free tray, Image Lab™ software, and unique precast gels (Criterion™ and Mini-PROTEAN® formats) that include unique trihalo compounds that allow rapid fluorescent detection of proteins — without staining. The trihalo compounds in the gels react with tryptophan residues in a UV-induced reaction to produce fluorescence, which can be easily detected by the imager either within gels or on low-fluorescence PVDF membranes. Activation of the trihalo compounds in the gels adds 58 Da moieties to available tryptophan residues and is required for protein visualization. Proteins that do not contain tryptophan residues cannot be detected using this system. The sensitivity of the system is comparable to staining with Coomassie (Brilliant) Blue for proteins with a tryptophan content >1.5%; sensitivity superior to Coomassie staining is possible for proteins with a tryptophan content >3%.

Benefits of stain-free technology include:

- Elimination of staining and destaining steps for faster results
- Automated gel and blot imaging and analysis
- No background variability (as is often seen with standard Coomassie staining)
- Reduced organic waste through elimination of the need for acetic acid and methanol in staining and destaining
- Visualization of transferred (blotted) proteins on low-fluorescence PVDF membranes

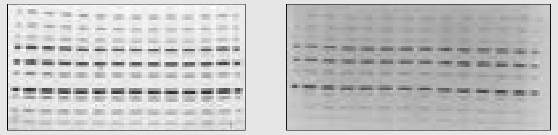

Gel Blot

Immunodetection Workflow

Transfer proteins to a membrane
▼
Block unbound membrane sites
▼
Incubate with primary antibody
▼
Wash away excess primary antibody
▼
Incubate with conjugated secondary antibody or ligand
▼
Wash away excess secondary antibody
▼
Develop signal based on the detection method
▼
Document and analyze results

Fig. 5.3. Immunodetection workflow.

Blocking

Following transfer, unoccupied binding sites on the membranes must be blocked to prevent nonspecific binding of probes; failure to completely block these sites can lead to high background. A variety of blocking reagents is available, including gelatin, nonfat milk, and bovine serum albumin (BSA), which are compared in Table 5.2. Optimize the detection system for minimal background with no loss of signal by testing several blocking agents. The type of membrane also affects the selection of blocker. Formulations for different blocking solutions are available in Part 2 of this guide.

Table 5.2. Comparison of blocking reagents.

Blocking Reagent	Membrane Compatibility	Recommended Concentration	Notes
Gelatin	Nitrocellulose	1–3%	Requires heat to solubilize
Nonfat dry milk, BLOTTO, blotting-grade blocker	Nitrocellulose, PVDF	0.5–5%	PVDF requires higher concentrations of nonfat milk than nitrocellulose
Bovine serum albumin (BSA)	Nitrocellulose, PVDF	1–5%	PVDF requires higher concentrations of BSA than nitrocellulose
Tween 20	Nitrocellulose	0.05–0.3%	May strip some proteins from the blot

Antibody Incubations

A typical immunodetection experimental system utilizes two rounds of antibody incubation:

- The primary antibody, which is directed against the target antigen; the antigen may be a ligand on a protein, the protein itself, a specific epitope on a protein, or a carbohydrate group
- The secondary antibody, which recognizes and binds to the primary antibody; it is usually conjugated to an enzyme such as AP or HRP, and an enzyme-substrate reaction is part of the detection process (Figure 5.4)

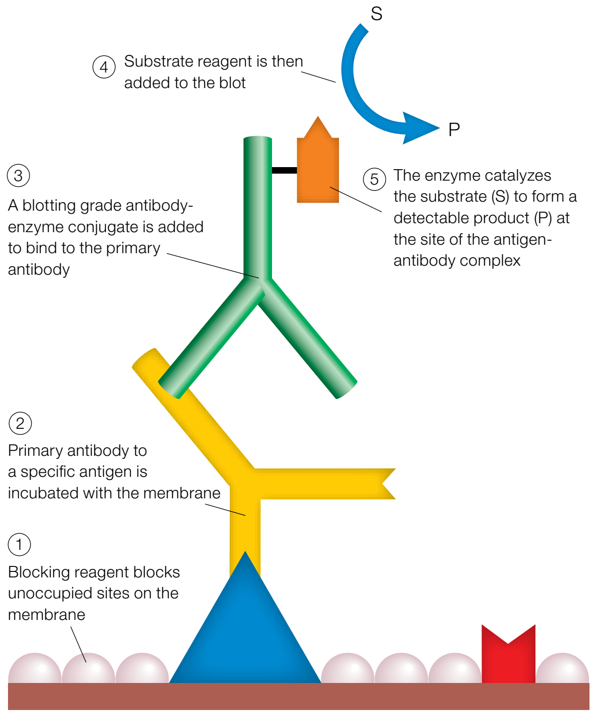

Fig. 5.4. Specific enzymatic detection of membrane-bound antigens.

Antibody incubations are generally carried out in antibody buffer containing Tris-buffered saline with Tween and a blocking reagent. Various formulations of antibody buffer are provided in Part 2 of this guide.

Washes

Washing the blot between the two antibody incubations and prior to detection removes excess antibody and prevents nonspecific binding. Though washing solutions and times may vary (depending on the antibodies and detection systems used), washes generally involve Tris-buffered saline (TBS) or phosphate-buffered saline (PBS). A detergent such as Tween 20 may be added to decrease background, but detergents may inhibit certain detection reactions (see the instruction manuals for details). Wash buffer formulations are described in Part 2 of this guide.

Links:
Criterion TGX Stain-Free Gels
Gel Doc EZ Imager
Colloidal Gold Total Protein Stain
Mini-PROTEAN II Multiscreen Apparatus
Image Lab Software

TABLE OF CONTENTS

Antibody Selection and Dilution

An antibody is an immunoglobulin protein such as IgG that is synthesized by an animal in response to exposure to a foreign substance, or antigen. Antibodies have specific affinity for the antigens that elicited their synthesis. Structurally, most IgG class antibodies contain four polypeptide chains (two identical heavy chains of ~55 kD and two identical light chains of ~25 kD) oriented in a "Y" shape (Figure 5.5). These are held together by disulfide bridges and noncovalent interactions. These proteins contain an F_{ab} region with specific affinity for the antigens that elicited their synthesis. In addition, a constant region (F_c) on the antibody provides binding sites for proteins needed during an immune response.

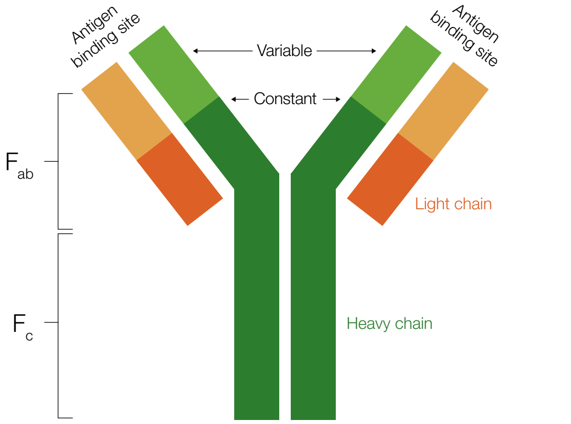

Fig. 5.5. Antibody structure. The components of a typical IgG molecule are highlighted and include the F_{ab} fragment containing the variable region responsible for antigen binding and the F_c, constant region, necessary for binding other proteins involved in the immune response.

Primary Antibodies

The primary antibody recognizes and binds to the target antigen on the membrane. For incubations with primary antibody, the entire blot must be covered with antibody-containing solution. The optimum antibody concentration is the greatest dilution of antibody that still yields a strong positive signal without background or nonspecific reactions. Instructions for antibodies obtained from a manufacturer typically suggest a starting dilution range. For custom antibodies or for those where a dilution range is not suggested, good starting points are:

- 1:100–1:1,000 dilution of the primary antibody in buffer when serum or tissue culture supernatants are the source of primary antibody
- 1:500–1:10,000 dilution of chromatographically purified monospecific antibodies
- 1:1,000–1:100,000 dilution may be used when ascites fluid is the source of antibody

Determine the appropriate concentration or dilution (titer) of the primary antibody empirically for each new lot of primary antibody. The Mini-PROTEAN® II multiscreen apparatus and mini incubation trays (described in the sidebar on page 35) are useful tools for determining antibody titer.

Species-Specific Secondary Antibodies

Secondary antibodies are specific for the isotype (class) and the species of the primary antibody (for instance, a goat anti-rabbit secondary antibody is an antibody generated in goat for detection of a primary antibody generated in a rabbit). Secondary antibodies bind to multiple sites on primary antibodies to increase detection sensitivity. For immunodetection, use only blotting-grade species-specific secondary antibodies.

Secondary antibodies can be labeled and detected in a variety of ways. The antibody can be radiolabeled or linked to a fluorescent compound or to gold particles, but most commonly the antibody is conjugated to an enzyme, such as alkaline phosphatase or horseradish peroxidase. If the secondary antibody is biotinylated, biotin-avidin-AP or -HRP complexes can be formed. Addition of a suitable enzyme substrate results in production of a colored precipitate or fluorescent or chemiluminescent product through dephosphorylation (by AP) or oxidation (by HRP).

Since the purity of the reagents is critical to the success of the experiment, the following steps are critical if the antibodies used are not blotting-grade:

- Purify all sera by affinity chromatography to obtain only those antibodies directed against the particular IgG; otherwise, background staining and false positive reactions due to nonspecific antibody binding may occur
- Cross-adsorb the purified antibody solution against an unrelated species; for example, human IgG for anti-rabbit and anti-mouse antibodies, and bovine IgG for anti-human reagents, to remove antibodies that are not specific for the species of interest

Blotting-grade antibodies are directed to both heavy and light chains of the IgG molecules, so the reagents can be used to identify other classes and subtypes of immunoglobulins.

Antibody-Specific Ligands

Protein A and protein G are bacterial cell surface proteins that bind to the F_c regions of immunoglobulin molecules (Akerstrom et al. 1985, Boyle and Reis 1987, Goding 1978, Langone 1982). The advantage of using protein A or protein G is their ability to bind to antibodies of many different species (Table 5.3). This is often desirable for laboratories using antibody probes from many different species or for those using one of the less common primary antibody systems in their experiments; that is, rat, goat, or guinea pig. In addition, these reagents bind only to antibody molecules; this can reduce the background from nonspecific binding of antibodies to membrane-bound proteins when a low-titer, poorly purified second antibody is used.

The major limitation of protein A and protein G conjugates is their lower sensitivity. Because only one ligand molecule binds to each antibody, the enhancement of a multiple-binding detection system, such as a species-specific polyclonal antibody, is lost. Generally, the species-specific antibody is 10–50 times more sensitive than the ligand reagent when the same detection system is used.

Detection Methods

Blotted proteins are generally detected using secondary antibodies that are labeled with radioisotopes or colloidal gold, or that are conjugated to fluorescent molecules (fluorophores) or an enzyme such as AP or HRP. Early blotting systems used ^{125}I-labeled reagents similar to those used in radioimmunoassay. These systems provide sensitive results but the special handling and disposal problems of ^{125}I reagents have discouraged continued use of this technique. Instead, a number of enzyme systems and detection reagents have evolved (Figure 5.6).

Table 5.3. Immunoglobulin-binding specificities of protein A and protein G.

Immunoglobulin	Protein A	Protein G
Human IgG_1	●●	●●
Human IgG_2	●●	●●
Human IgG_3	—	●●
Human IgG_4	●●	●●
Mouse IgG_1	●	●
Mouse IgG_2	●●	●●
Mouse IgG_3	●●	●●
Mouse IgG_4	●●	●●
Rat IgG_1	●	●
Rat IgG_{2a}	—	●●
Rat IgG_{2b}	●●	●
Rat IgG_{2c}	●●	●●
Pig IgG	●	●●
Rabbit IgG	●●	●●
Bovine IgG_1	—	●●
Bovine IgG_2	●●	●●
Sheep IgG_1	—	●●
Sheep IgG_2	●	●●
Goat IgG_1	●	●●
Goat IgG_2	●●	●●
Horse $IgG_{(ab)}$	●	●●
Horse $IgG_{(c)}$	●	●●
Horse $IgG_{(t)}$	—	●
Dog IgG	●●	●

●● = Strong binding   ● = Weak binding   — = No binding

Screening Antibodies

In some experiments, protein blots must be screened for a number of different antigens or under a number of different conditions.

Mini Incubation Trays

Mini incubation trays allow safe, simple, and economical screening of different antigens on protein blot strips. Each tray has eight 10.5 cm x 5 mm channels to accommodate strips cut from a particular protein blot. Because the trays are disposable, the potential contamination associated with washing reusable trays is eliminated. Ribs in the tray lids combine with the overall design of the sample channels to ensure that no cross-contamination occurs.

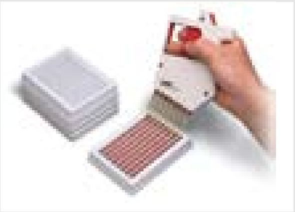

Mini incubation tray.

Mini-PROTEAN® II Multiscreen Apparatus

When proteins are resolved by SDS-PAGE and blotted onto a membrane for analysis, the Mini-PROTEAN II multiscreen apparatus simplifies the screening process. Instead of being cut into individual strips for incubation, the entire blot is simply clamped into the multiscreen unit for assay. Two separate, detachable sample templates allow up to 40 different antibody or serum samples to be screened. The unique molded gasket ensures a leakproof seal, preventing cross-contamination among samples.

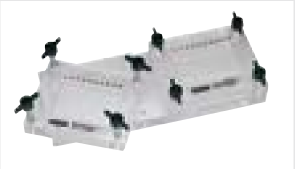

Multiscreen apparatus.

Links:

Mini-PROTEAN II Multiscreen Apparatus

Mini Incubation Trays

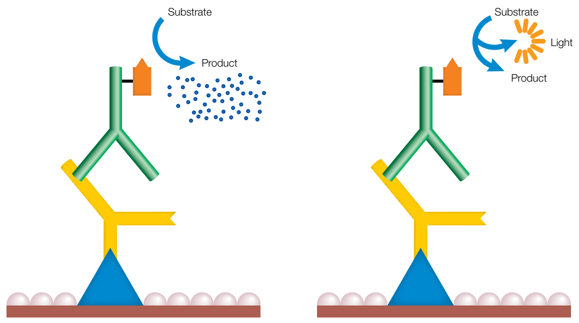

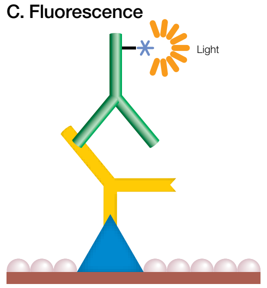

Fig.5.6. Mechanism of detection chemistries. In each method of western blot detection, a detectable signal is generated following binding of an antibody specific for the protein of interest. In colorimetric detection (**A**), the signal is a colored precipitate. In chemiluminescence (**B**) the reaction itself emits light. In fluorescence detection (**C**), the antibody is labeled with a fluorophore.

The most commonly used detection methods use secondary antibodies conjugated to alkaline phosphatase or horseradish peroxidase. With these methods, when the enzyme substrate is added, either a colored precipitate is deposited on the blot (colorimetric detection) or a chemiluminescent or fluorescent product is formed and the light signal is captured on film or with a digital imaging system (Figure 5.6). Secondary antibodies conjugated to fluorophores are gaining popularity and can be directly visualized on the blot and captured with a compatible imager, without the need for additional liquid substrate (see the sidebar Fluorescence Detection on page 40).

Colorimetric Detection

Enzymes such as AP and HRP convert several substrates to a colored precipitate (Table 5.4). As the precipitate accumulates on the blot, a visible colored signal develops that is visible on the blot (Figure 5.6A). The enzyme reaction can be monitored and stopped when the desired signal over background is produced. Colorimetric detection is easier to use than any film-based detection method, which must be developed by trial and error and uses costly materials such as X-ray film and darkroom chemicals. Colorimetric detection is considered a medium-sensitivity method, compared to radioactive or chemiluminescence detection.

- Colorimetric HRP systems — the first enzyme conjugates used for immunological detection of blotted proteins. The advantage of HRP systems was that both the enzyme conjugate and colorimetric detection substrates were economical. The most common color substrates for HRP are 4-chloro-1-naphthol (4CN) (Hawkes et al. 1982) and 3,3'-diaminobenzidine (DAB) (Tsang et al. 1985) (Figure 5.7). HRP colorimetric detection systems are not as sensitive as AP colorimetric detection systems. Fading of blots upon exposure to light, inhibition of HRP activity by azide, and nonspecific color precipitation are additional limitations of HRP colorimetric detection systems
- Colorimetric AP systems — use soluble 5-bromo-4-chloro-3-indolyl phosphate (BCIP) and nitroblue tetrazolium (NBT) as substrates to produce a stable reaction product that will not fade (Figures 5.8 and 5.9A). AP can easily be inactivated by exposure to acidic solutions. Multiple probing of the same membrane with alternative antibody probes can be performed using substrates that produce different colors, such as blue and red (Blake et al. 1984, Turner 1983, Kurien 2003)

Table 5.4. Colorimetric detection systems.

Detection Method	Substrate	Detection Sensitivity	Signal Color	Product Options	Advantages	Disadvantages
Colorimetric HRP	4CN	500 pg	Purple	Dry powder, liquid substrate, Immun-Blot® kits	Fast color development, low cost, low background	Results fade over time, azide inhibits enzyme activity
	DAB	500 pg	Brown	Dry powder	Fast color development, low background	More safety precautions than for other substrates<br>Azide inhibits enzyme activity
	Opti-4CN™	100 pg	Purple	Liquid substrate; Opti-4CN kit	High sensitivity, nonfading color, low background	More expensive than 4CN
	Amplified Opti-4CN	5 pg	Purple	Amplified Opti-4CN kit	Best sensitivity available, no extra materials (such as X-ray film) needed	More steps than unamplified protocol
Colorimetric AP	BCIP/NBT	100 pg	Purple	Dry powder, liquid substrate, Immun-Blot kits	Stable storage of data	Detects endogenous phosphatase activity
	Amplified BCIP/NBT	10 pg	Purple	Amplified AP Immun-Blot kit	High sensitivity	More steps than unamplified protocol

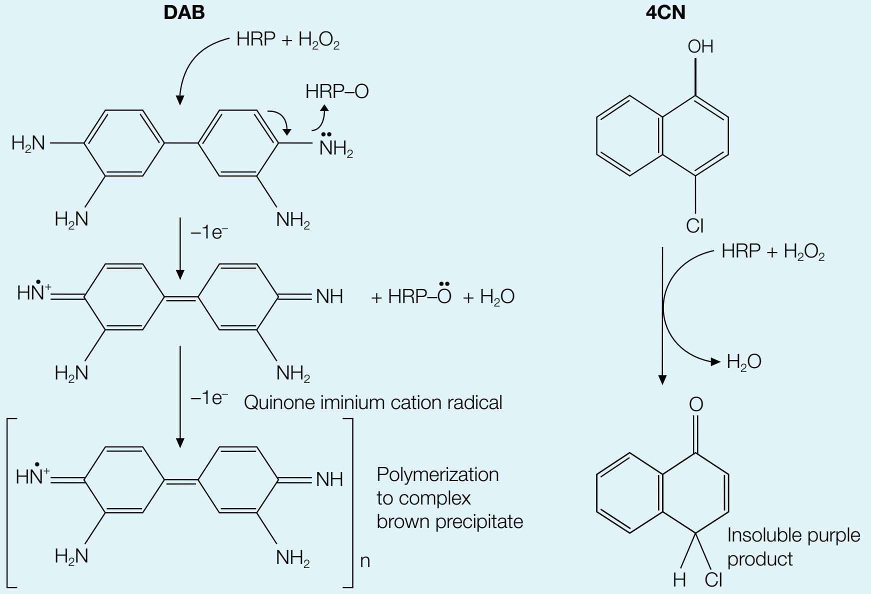

Fig. 5.7. Colorimetric detection options with HRP. DAB and 4CN are commonly used chromogenic substrates for HRP. In the presence of H_2O_2, HRP catalyzes the oxidation of the substrate into a product that is visible on a blot. **Left**, reaction with DAB; right, reaction with 4CN.

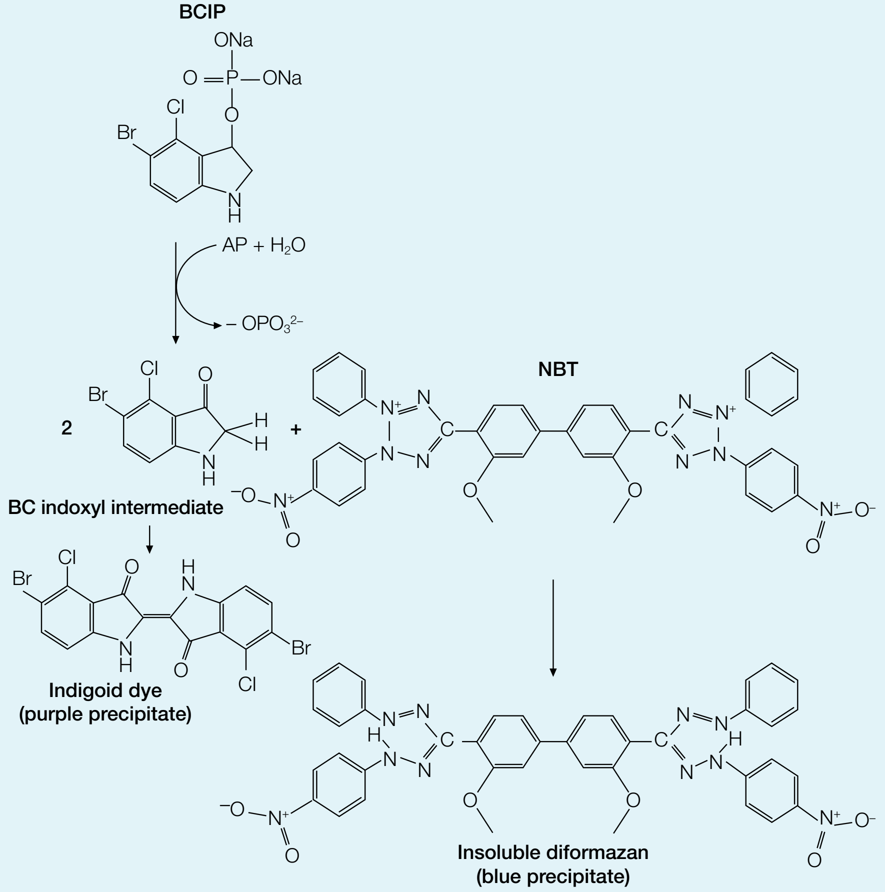

Fig. 5.8. AP colorimetric development. In the colorimetric system, AP catalyzes the substrates BCIP and NBT to produce a colored precipitate visualizing the protein on a western blot. First the dephosphorylation of BCIP by AP occurs, yielding a bromochloro indoxyl intermediate. The indoxyl is then oxidized by NBT to produce an indigoid dye (purple precipitate). The NBT is also reduced by the indoxyl, opening the tetrazole ring to produce an insoluble diformazan (blue precipitate). The combination of the indigoid dye of the BCIP and the insoluble formazan of the NBT forms a purple-blue colored precipitate.

TABLE OF CONTENTS

Premixed and Individual Colorimetric Substrates

Premixed enzyme substrate kits and development reagents, including powdered 4CN and DAB color development reagents, are also available. The premixed kits are convenient and reliable, and they reduce exposure to hazardous reagents used in the color development of protein blots.

Immun-Blot® Assay Kits

Immun-Blot assay kits provide the reagents required for standard HRP/4CN or AP colorimetric detection on western blots with the added convenience of premixed buffers and enzyme substrates. In addition, these kits contain a secondary antibody conjugated to either HRP or AP. All kit components are individually tested for quality in blotting applications. Included in each kit is an instruction manual with a thoroughly tested protocol and troubleshooting guide that simplifies immunological detection.

Immun-Blot Amplified AP Kit

Increased sensitivity in western blot experiments can be achieved by utilizing an amplified AP procedure (Bayer and Wilchek 1980, Chaiet and Wolf 1964, Guesdon et al. 1979, Hsu et al. 1981). This detection system (Figure 5.10A) begins by using a biotinylated secondary antibody. Relying on the specific binding properties of biotin and avidin, a complex of streptavidin and biotinylated AP is then added to the membrane. Because streptavidin will bind more than one molecule of biotin, the initial site of the primary antibody-to-antigen binding is effectively converted into multiple AP binding sites available for color development (Figure 5.10A). Color development is performed using conventional AP substrates, as discussed previously. The Immun-Blot amplified AP kit increases the detection sensitivity of colorimetric western blotting to ≥10 pg of protein.

Opti-4CN™ and Amplified Opti-4CN Substrate and Detection Kits

Colorimetric HRP detection with 4CN presents very low background and a detection sensitivity of about 500 pg of antigen. Bio-Rad's Opti-4CN kit improves this detection sensitivity to 100 pg. Opti-4CN is available as a premixed substrate kit or combined with an HRP-conjugated antibody in a detection kit.

Amplified Opti-4CN substrate and detection kits are based on proprietary HRP-activated amplification reagents from Bio-Rad. These kits allow colorimetric detection to 5 pg, which is comparable to or greater than the sensitivity achieved with radiometric or some chemiluminescence systems but without the cost or time involved in darkroom development of blots.

Chemiluminescence Detection

Chemiluminescence is a chemical reaction in which a chemical substrate is catalyzed by an enzyme, such as AP or HRP, and produces light as a by-product (Figures 5.6B, 5.9B, and 5.10B). The light signal can be captured on X-ray film or by a charge-coupled device (CCD) imager such as the ChemiDoc™ XRS+ and ChemiDoc™ MP systems. This technology is easily adapted to existing western blotting procedures because chemiluminescence uses enzyme-conjugated antibodies to activate the light signal. The blocking and wash methods are familiar procedures.

The advantages of chemiluminescent western blotting over other methods are its speed and sensitivity (Table 5.5). This method is perfect for CCD imaging, which avoids the slow film step. Exposure times with average blots are usually 5 sec to 5 min, depending on the sensitivity of the substrate. This is a large improvement over ^{125}I systems, which can require up to 48 hr for film exposure. Detection of protein down to femtogram amounts is possible with these systems. This is more sensitive than most colorimetric systems and approximately equal to radioisotopic detection. The detection sensitivity depends on the affinity of the protein, primary antibody, secondary antibody, and HRP substrate, and can vary from one sample to another.

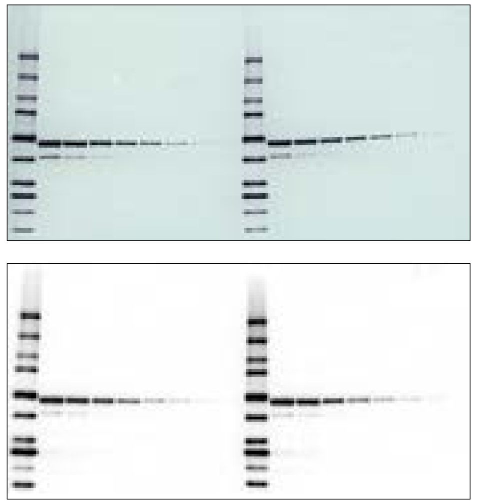

Fig. 5.9. Colorimetric and chemiluminescent blots. A dilution of a GST fusion protein was immunodetected using a monoclonal antibody specific to GST followed by **A,** an AP-conjugated secondary antibody and BCIP/NBT substrate for colorimetric detection, or **B,** an HRP-conjugated secondary antibody and chemiluminescent substrate for chemiluminescence detection.

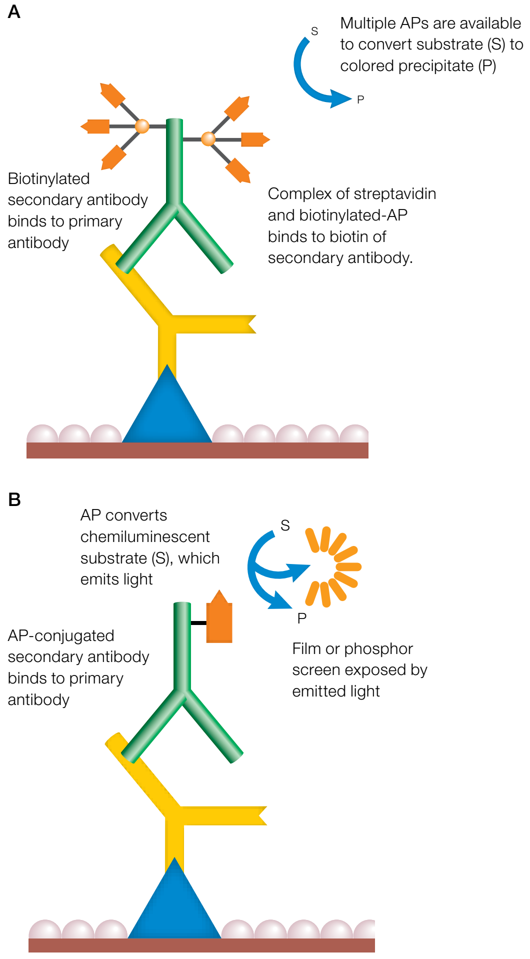

Fig. 5.10. Colorimetric and chemiluminescence detection methods. **A**, Detection using Immun-Blot amplified AP kit; **B**, Detection using Immun-Star™ AP chemiluminescent kit.

Table 5.5. Chemiluminescence detection systems.

Method	Detection Substrate	Sensitivity	Detection Product Options	Advantages	Disadvantages
**Chemiluminescent HRP**					
Clarity™ western ECL substrate	Luminol	Mid-femtogram	HRP substrate	Short (30 sec) exposure<br>Signal duration up to 24 hr<br>Compatible with PVDF and nitrocellulose<br>Stable at room temperature	Azide inhibits enzyme activity
Clarity Max™ western ECL substrate	Luminol	Low-femtogram	HRP substrate	Compatible with PVDF and nitrocellulose<br>Optimized for CCD imagers<br>High sensitivity<br>Stable at room temperature	Azide inhibits enzyme activity
**Chemiluminescent AP**					
Immun-Star™ AP kit	CDP-*Star*	10 pg	Conjugates<br>AP substrate<br>Immun-Blot kits	30 sec to 5 min exposure<br>Signal duration up to 24 hr<br>Blot can be reactivated	Endogenous phosphatase activity may lead to false positives

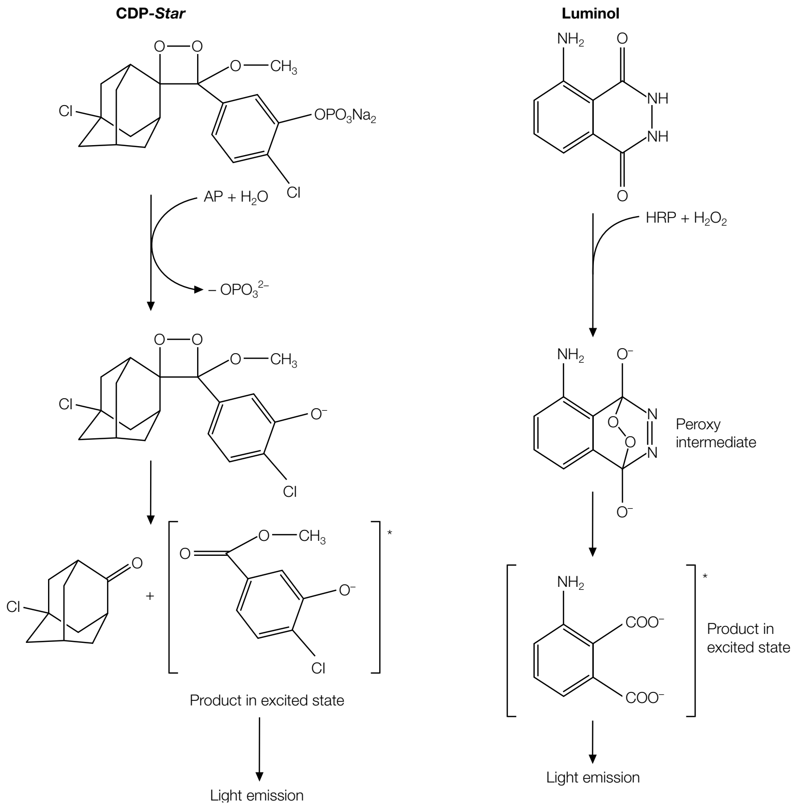

Fig. 5.11. Chemiluminescence detection. The secondary antibody is linked to an enzyme, which catalyzes a reaction leading to light emission. **Left**, CDP-*Star* or another 1,2-dioxetane AP substrate is dephosphorylated by AP, resulting in formation of an anion in an excited state that emits light. **Right**, luminol oxidized by HRP in the presence of H_2O_2 leads to the formation of a 3-aminophthalate dianion and the release of light.

TABLE OF CONTENTS

Links:

Immun-Blot AP Colorimetric Kits

Immun-Blot Opti-4CN Colorimetric Kits

Clarity and Clarity Max Western ECL Substrates

Immun-Star AP Chemiluminescence Kits

ChemiDoc MP System

ChemiDoc XRS+ System

Fluorescence Detection

In fluorescence, a high-energy photon ($h\nu_{ex}$) excites a fluorophore, causing it to leave the ground state (S_0) and enter a higher energy state (S'_1). Some of this energy dissipates, allowing the fluorophore to enter a relaxed excited state (S_1). A photon of light is emitted ($h\nu_{em}$), returning the fluorophore to the ground state. The emitted photon is of a lower energy (longer wavelength) due to the dissipation of energy while in the excited state.

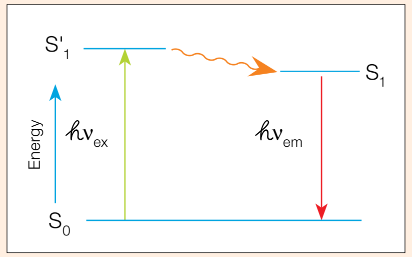

When using fluorescence detection, consider the following optical characteristics of the fluorophores to optimize the signal:

- **Quantum yield** — efficiency of photon emission after absorption of a photon. Processes that return the fluorophore to the ground state but do not result in the emission of a fluorescence photon lower the quantum yield. Fluorophores with higher quantum yields are generally brighter
- **Extinction coefficient** — measure of how well a fluorophore absorbs light at a specific wavelength. Since absorbance depends on path length and concentration (Beer's Law), the extinction coefficient is usually expressed in cm^{-1} M^{-1}. As with quantum yield, fluorophores with higher extinction coefficients are usually brighter
- **Stokes shift** — difference in the maximum excitation and emission wavelengths of a fluorophore. Since some energy is dissipated while the fluorophore is in the excited state, emitted photons are of lower energy (longer wavelength) than the light used for excitation. Larger Stokes shifts minimize overlap between the excitation and emission wavelengths, increasing the detected signal
- **Excitation and emission spectra** — excitation spectra are plots of the fluorescence intensity of a fluorophore over the range of excitation wavelengths; emission spectra show the emission wavelengths of the fluorescing molecule. Choose fluorophores that can be excited by the light source in the imager and that have emission spectra that can be captured by the instrument. When performing multiplex western blots, choose fluorophores with minimally overlapping spectra to avoid channel crosstalk

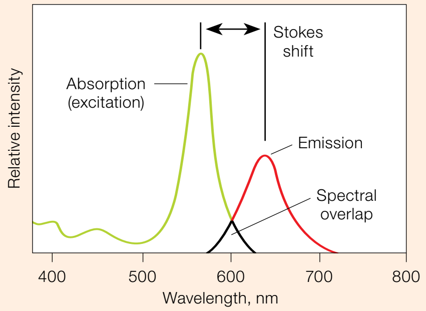

Safety is another advantage of chemiluminescence detection. It does not have the disadvantages related to isotope detection, such as exposure of personnel to radiation, high disposal costs, and environmental concerns.

Immun-Star™ AP Kits, Clarity,™ and Clarity Max™ Western ECL Substrates

Bio-Rad offers substrates for activation by AP or HRP. The Clarity and Clarity Max™ western ECL substrates are activated by HRP and are compatible with any HRP-conjugated antibody. Clarity western ECL substrate provides strong signal intensity with low background and lasts for 24 hrs. Clarity Max western ECL substrate provides very bright signal, offering femtogram-level sensitivity to enable the detection of low abundance proteins, or compensate for poor affinity antibodies.

The Immun-Star™ AP kits include CDP-Star substrate, which is activated by AP. These kits also provide strong signal for fast exposures and have signal that lasts 24 hours. All of the Bio-Rad chemiluminescent substrates work well with Precision Plus Protein™ WesternC™ standards (Figure 5.12) on nitrocellulose and PVDF membranes, and work for detection on film or CCD imagers.

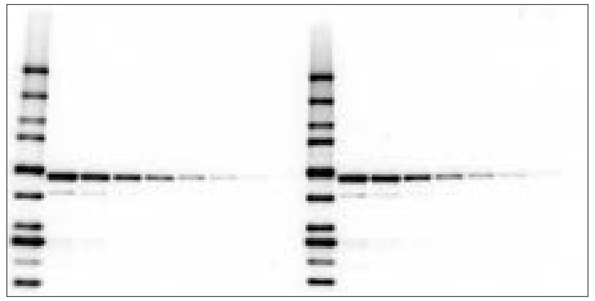

Fig. 5.12. Detection of antigen and Precision Plus Protein WesternC standards using the chemiluminescence detection. Proteins and 5 µl standards (lane 1) and a dilution series of an *E. coli* cell lysate (lanes 2–6) were electrophoresed on a 4–20% Criterion™ gel and transferred to a nitrocellulose membrane. The blot was probed with an antibody specific for GST fusion proteins followed by an HRP-conjugated secondary antibody and StrepTactin-HRP conjugate. After a 5 min incubation in chemiluminescent detection solution, the blot was imaged on a ChemiDoc™ XRS+ imager for 5 sec.

Fluorescence Detection

In fluorescence detection, a primary or secondary antibody labeled with a fluorophore is used during immunodetection. A light source excites the fluorophore and the emitted fluorescent signal is captured by a camera to produce the final image (see sidebar at left).

Several fluorophores spanning a wide range of excitation and emission wavelengths are now available, including some based on organic dyes (for example, cyanine and fluorescein), nanocrystals of semiconductor material (for example, Qdot nanocrystals), and naturally fluorescent proteins (for example, phycobiliproteins such as phycoerythrin and allophycocyanin).

Fluorescence detection (Figure 5.13) offers several advantages over other methods:

- Multiplexing — use of multiple and differently colored fluorophores for simultaneous detection of several target proteins on the same blot. When detecting multiple proteins in a fluorescent multiplex western blot, ensure the fluorescent signals generated for each protein can be differentiated. Use primary antibodies from different host species (for example, mouse and rabbit) and secondary antibodies that are cross-absorbed against other species to avoid cross-reactivity. Use fluorophores conjugated to secondary antibodies with distinct spectra so they can be optically distinguished from each other to avoid cross-channel fluorescence
- Dynamic range — a 10-fold greater dynamic range over chemiluminescence detection and, therefore, better linearity within detection limits
- Stability — many fluorescent molecules are stable for a long period of time, allowing blots to be stored for imaging at a later date — often weeks or months later — without significant signal loss. Most fluorescence techniques are also compatible with stripping and reprobing protocols (provided the blots are not allowed to dry out between successive western detection rounds)

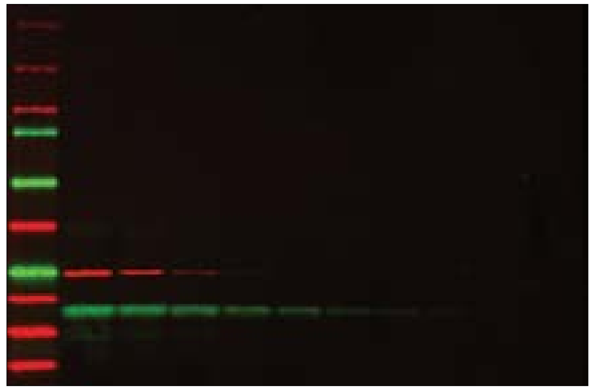

Fig. 5.13. Multiplex fluorescence detection of a two-fold dilution series of two proteins, GST (red) and soybean trypsin inhibitor (green). Starting concentration was 500 ng of each protein. Precision Plus Protein™ WesternC™ standards were used as markers.

A drawback of fluorescence detection is its reduced sensitivity compared to chemiluminescence methods, such that detection using low-affinity antibodies or of low-abundance proteins may yield lower signals. Photostable fluorophores, improved instrumentation, and membranes with low autofluorescence characteristics are available to allow fluorescence detection to approach the sensitivities seen with chemiluminescence techniques.

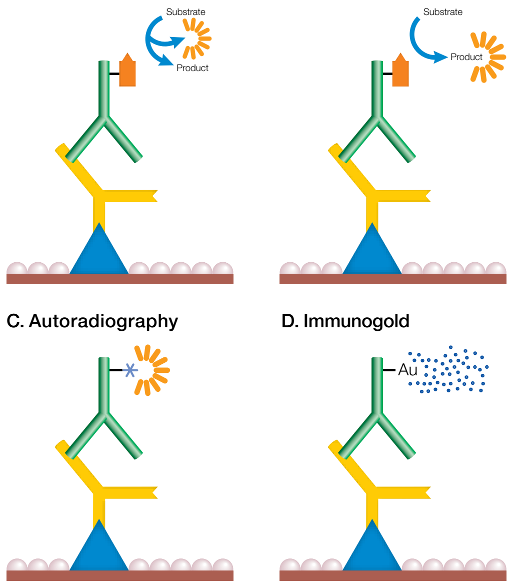

Fig. 5.14. Mechanism of detection chemistries. In bioluminescence detection (**A**), the enzyme reaction itself emits light, while in chemifluorescence (**B**), the product of the reaction is fluorescence. In autoradiography (**C**), the secondary antibody itself carries a radioactive label, and in immunogold labeling (**D**), the secondary antibody is labeled with gold and signal is enhanced by silver precipitation.

Other Detection Methods

Bioluminescence

Bioluminescence is the natural light emission by many organisms. Bioluminescence systems differ in the structure and function of enzymes and cofactors involved in the process as well as the mechanism of the light-generating reactions. Bioluminescence is also used as a detection method for proteins and nucleic acids on a membrane.

Bioluminescence detection involves incubation of the membrane (with bound antigen-antibody-enzyme complex) in a bioluminogenic substrate and simultaneous measurement of emitted light (Figure 5.14A). The substrate involved in this detection system is a luciferin-based derivative. Light detection is performed using a photon-counting camera and the blotted proteins are visualized as bright spots.

This technique is similar to chemiluminescence in its sensitivity and speed of detection, but it is not widely used and few bioluminogenic substrates

Links

Clarity and Clarity Max Western ECL Substrates

Immun-Star AP Chemiluminescence Kits

Precision Plus Protein Western C Standards

TABLE OF CONTENTS

TABLE OF CONTENTS

are commercially available. PVDF is the preferred membrane for bioluminescence detection because nitrocellulose membranes may contain substances that inhibit luciferase activity.

Chemifluorescence

Chemifluorescence is the enzymatic conversion of a substrate to a fluorescent product (Figure 5.14B). Fluorogenic compounds (nonfluorescent or weakly fluorescent substances that can be converted to fluorescent products) are available to use with a wide variety of enzymes, including AP and HRP. The enzyme cleaves a phosphate group from a fluorogenic substrate to yield a highly fluorescent product. The fluorescence can be detected using a fluorescence imager such as the ChemiDoc MP system. Chemifluorescence can provide a stable fluorescent reaction product so blots can be scanned at a convenient time. The method is compatible with standard stripping and reprobing procedures.

Autoradiography

The gamma-emitting radioisotope ^{125}I can be used to label lysines in immunoglobulins for radiometric antigen detection (Figure 5.14C). Direct immunological detection (using labeled secondary antibodies) of as little as 1 pg of dotted immunoglobulin is possible with high specific activity ^{125}I probes. Radiolabeled blots can be detected using X-ray film, a method known as autoradiography. Due to the hazards associated with radiolabeled conjugates, autoradiography is declining in popularity in favor of colorimetric and chemiluminescence methods.

Immunogold Labeling

Immunogold detection methods utilize gold-labeled secondary antibodies for antigen detection. Because this method has relatively low sensitivity and the signal is not permanent, silver enhancement methods similar to those described on page 32 for colloidal gold total protein stains were developed as a means of enhancing the signal (Figure 5.14D). With silver enhancement, a stable dark brown signal with little background is produced on the blot and sensitivity is increased 10-fold, equivalent to colorimetric AP detection and several times more sensitive than autoradiography.

Stripping and Reprobing

Membranes that have been detected with noncolorimetric methods such as chemiluminescent or fluorescent techniques can be stripped of antibodies for use in subsequent rounds of Western detection (Figure 5.15). This allows reuse of the same blot for investigation of different proteins and saves both time and sample material.

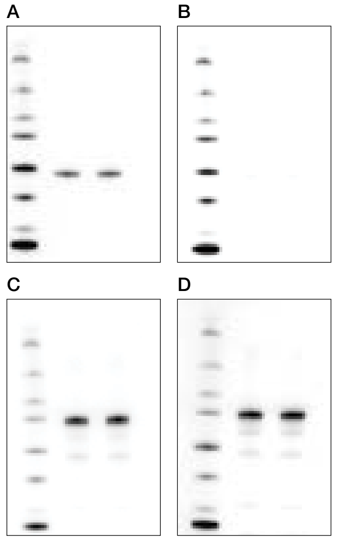

Fig. 5.15. Stripping and reprobing PVDF membranes. *E. coli* lysate containing human transferrin and a GST-tagged protein was loaded on a gel and blotted onto PVDF membranes. **A**, the blot probed with an anti-GST antibody and developed with chemiluminescent substrate. **B**, the same blot subsequently stripped of antibody and reprobed with the secondary antibody and developed with chemiluminescent substrate to demonstrate removal of primary antibody. This blot was also reprobed with StrepTactin-HRP to visualize the ladder. **C**, the stripped blot reprobed with an anti-human transferrin antibody. **D**, a control blot that did not undergo the stripping procedure probed with the anti-human transferrin antibody.

Blots can be stripped and reprobed several times but each round of stripping removes some sample from the blot. This decreases the sensitivity of later rounds of detection and may necessitate longer exposure times or more sensitive detection methods.

When probing a blot multiple times:

- If detecting proteins of different abundance or when using antibodies with very different binding affinities, first detect the protein with the lower expected signal sensitivity
- Comparisons of target protein abundance among different rounds of detection will be unreliable, as some sample is removed during the stripping process
- If possible, use PVDF membranes. PVDF is more durable and resists loss of sample better than nitrocellulose membranes
- After stripping a blot, test it for complete removal of the antibody. If chemiluminescent detection methods were used, confirm removal of the secondary antibody by incubation with fresh chemiluminescent substrate. Detect any remaining primary antibodies by incubation with an HRP-labeled secondary antibody followed by incubation with fresh chemiluminescent substrate. If any antibody is detected using these tests, restrip the blot before subsequent experiments

Table 5.6. Comparison of western blot documentation and analysis methods.

	Imaging System		
	ChemiDoc™	ChemiDoc MP™	ChemiDoc™XRS+
**Immunodetection**			
Chemiluminescence	•	•	•
Colorimetric	•	•	•
Fluorescence	—	•	—
**Total Protein Stain**			
Colorimetric	•	•	•
Fluorescent	•	•	•
Stain-free	•	•	•
**Imager Type**			
Supercooled CCD	•	•	•
**Excitation Type**			
Trans UV/Vis	•	•	•
Epi white	•	•	•
LED RGB	—	•	—
LED NIR	—	•	—

Note: The ChemiDoc MP is capable of up to 3-channel multiplex fluorescence detection.

Imaging — Analysis and Documentation

Several methods are employed to document western blotting results (Table 5.6).

- Densitometers — based on high-performance document scanners with minor modifications (leak-resistant scanning surface, built-in calibration tool) and utilize visible light for analysis of electrophoresis gels (transmission mode) and blots (reflective mode) stained with visible dyes
- CCD (charge-coupled device) cameras — versatile systems that image both gels and blots, and operate with either trans-illumination provided by light boxes (visible or UV) positioned underneath the gel for imaging a variety of stains (Coomassie, silver, fluorescence) or epi-illumination of blots detected using colorimetric or fluorescence techniques. Different illumination wavelengths are available for multiplex fluorescence immunodetection. CCD cameras can also be used without illumination to detect luminescent signals. Supercooled CCD cameras reduce image noise, allowing detection of faint luminescent signals
- Laser-based imagers — offer the highest sensitivity, resolution, and linear dynamic range and are powerful image acquisition tools for blots stained with fluorescent dyes such as SYPRO Ruby. These imagers can be configured with lasers of different wavelengths, allowing single- or multiplex fluorescence immunoblot detection
- Phosphor imagers — laser-based systems capable of imaging storage phosphor screens. These screens have a large dynamic range and offer excellent sensitivity and quantitative accuracy with imaging times that are a fraction of those for film. These imagers can be used for autoradiographic detection techniques
- X-ray film — widely used for imaging autoradiographic and chemiluminescent blots but suffers from a limited dynamic range as well as a nonlinear response through this range. This method also requires the investment in and maintenance of a film developer and often requires processing multiple film sheets to obtain a usable image. Decreasing costs, higher resolution, better ease of use, and a larger, more linear dynamic range are making CCD cameras and imagers preferred over film for these detection techniques

Luminescence Detection

For chemiluminescence detection, CCD imaging is the easiest, most accurate, and rapid method. Traditionally, the chemiluminescent signal from blots was detected by X-ray film. Film is a sensitive medium for capturing the chemiluminescent signal but suffers from a sigmoidal response to light with a narrow region or linear response, which limits its dynamic range. To gather information from a blot that has both intense and weak signals, multiple exposures are required to produce data for all samples in the linear range of the film. A process termed preflashing can improve linearity but this requires extra equipment and effort. Additionally, quantitation of data collected by exposure to film requires digitization (that is, scanning of X-ray film with a densitometer).

CCD cameras have a linear response over a broad dynamic range — 2–5 orders of magnitude — depending on the bit depth of the system. CCD cameras also offer convenience by providing a digital record of experiments for data analysis, sharing, and archiving, and by eliminating the need to continually purchase consumables for film development. CCD cameras also approach the limit of signal detection in a relatively short time.

Links:

Gel Doc EZ Imager

Gel Doc XR+ System

ChemiDoc XRS+ System

ChemiDoc MP Systems

GS-900 Calibrated Densitometer

For example, the ChemiDoc MP™ imaging system can reach the limit of detection of a given experiment in <1 min, compared to 30 min required by Kodak Bio-Max film for the same experiment.

Digital Imaging for Fluorescence, Chemifluorescence, and Colorimetric Detection

Fluorescence, chemifluorescence, and colorimetric detection all benefit from the advantages of digital imaging: convenience, digital records of experiments, sensitive limits of detection, and wide dynamic ranges. Fluorescent and chemifluorescent signals can be detected with a wide range of imaging systems, including both CCD and laser-based technologies. The decision to use one type of technology over another depends on budget and requirements for limit of detection and resolution. CCD systems are generally less expensive than laser-based systems. While the dynamic range of CCD imaging systems varies from 2 to 5 orders of magnitude, laser-based systems typically provide a wide dynamic range of 4.8 orders of magnitude. The resolution of CCD and laser-based systems are similar, with the finest resolution settings generally being 50 µm or less. Another advantage of fluorescence and chemifluorescence detection is that CCD and laser-based detection limits generally far exceed the dynamic ranges of the fluorescence assays currently used for protein detection.

Colorimetric samples can be easily recorded and analyzed with a densitometer such as the GS-900™ calibrated densitometer. The densitometer provides a digital record of the blot, excellent resolution, reproducible results, and accurate quantitation. The GS-900 also uses red, green, and blue color CCD technology to greatly improve the imaging of a wide range of colorimetric detection reagents.

Autoradiography

To detect the commonly used radioisotopes, ^{35}S, ^{32}P, ^{33}P, ^{12}C, and ^{125}I, the most widely used method is autoradiography on X-ray film. Autoradiography provides a good combination of sensitivity and resolution without a large investment in detection substrates or imaging systems. For direct autoradiography without intensifying screens or scintillators, the response of the film is linear only within a range of 1–2 orders of magnitude. When intensifying screens or fluorographic scintillators are used to increase sensitivity, the response of the film is nonlinear but it can easily be made linear by preexposing the film to a flash of light. Phosphor imagers offer an alternative to film detection methods. The initial investment in instrumentation offers increased sensitivity and dynamic range compared to X-ray film, and exposure times are 10 to 20 times shorter than those for film. The ability to accurately quantitate data is also much greater with storage phosphor screens because the linear dynamic range of phosphor imagers is significantly greater — 5 orders of magnitude — enabling accurate quantitation and the elimination of overexposure and saturated signals.

Analysis Software

Blot detection using an imaging system needs a robust software package for image acquisition. In addition, a good software package can magnify, rotate, resize, overlay, and annotate the corresponding gel and blot images, allowing export of the images to common documentation software. A good software package also allows analysis of the blot image and comparisons of relative signal intensities, protein molecular weight, or other aspects.

Image Lab™ software (Figure 5.16) controls a variety of Bio-Rad imaging systems. It automatically determines the image with the best signal-to-noise ratio and generates a report. It also provides sophisticated algorithms to determine the number of lanes and bands in the image. The software can measure total and average quantities and determine relative and actual amounts of protein. Gel imaging software is also capable of determining the presence/absence and up/down regulation of bands, their molecular weights, isoelectric points, and other values. Signal intensities can be quantitated and compared to determine relative signal and to generate other data such as R_f values for molecular weight determinations

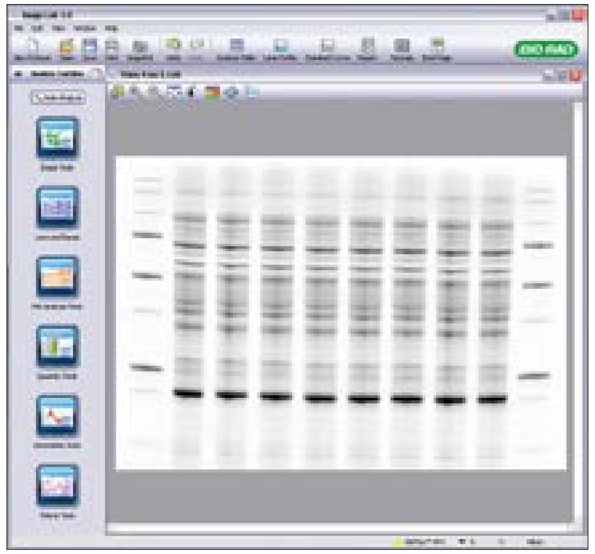

Fig. 5.16. Image Lab software. *E. coli* lysate was separated and activated on a 4–20% Criterion™ TGX Stain-Free™ gel and transferred onto a PVDF membrane. The membrane was imaged on a Gel Doc™ EZ system and analyzed using Image Lab 3.0 software.

TABLE OF CONTENTS

Links:

Image Lab Software

TABLE OF CONTENTS

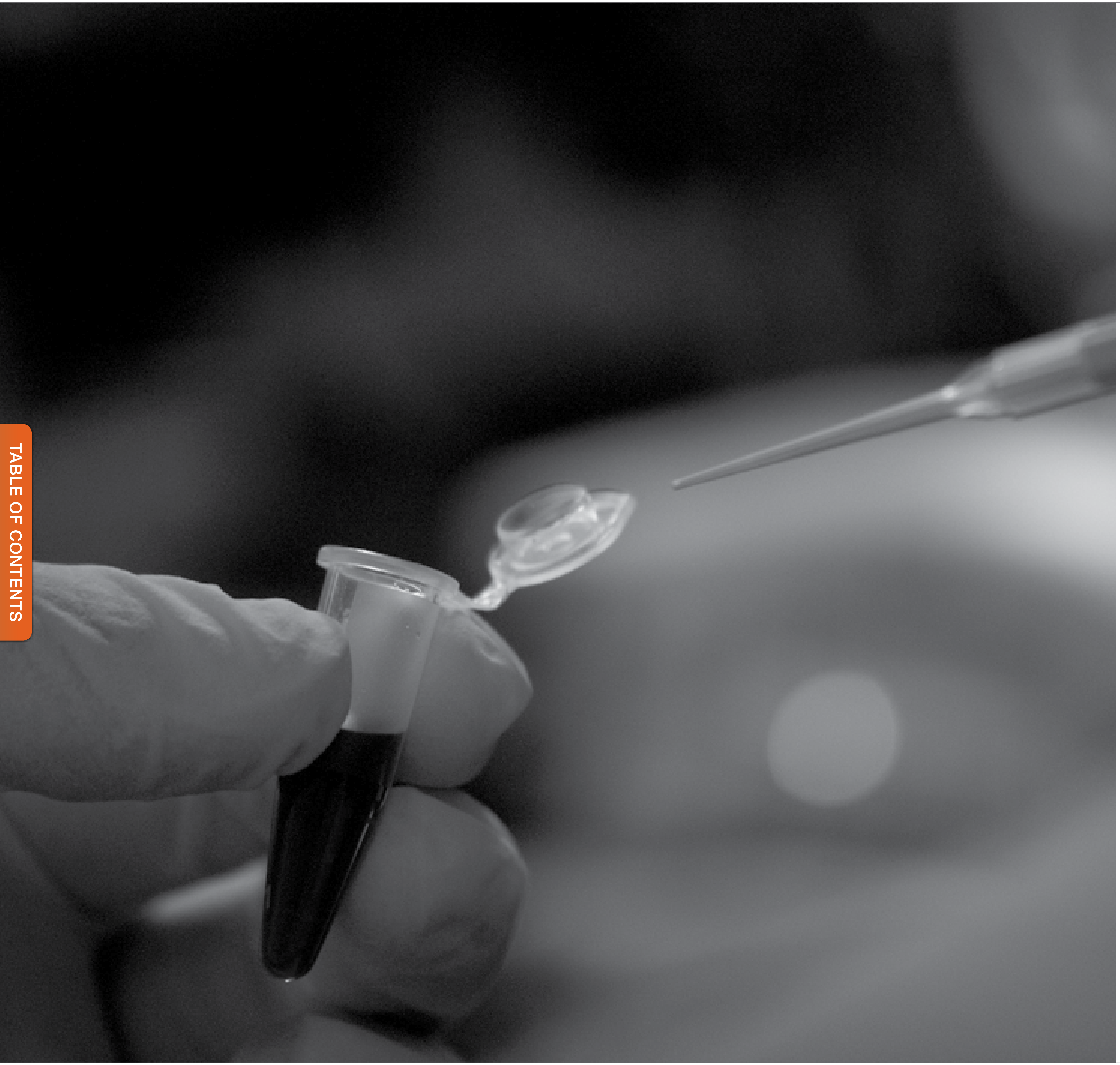

PART 2

Methods

Part 2 presents general protocols for transfers of proteins to membranes, total protein detection, and immunodetection.

Protocols

Electrophoretic Transfers

Reagent and Materials Preparation

TIPS

Gel equilibration removes contaminating electrophoresis buffer salts. If not removed, these salts increase the conductivity of the transfer buffer and the amount of heat generated during transfer.

Equilibration also allows the gel to adjust to its final size prior to electrophoretic transfer. Gels shrink or swell to various degrees in the transfer buffer depending on the acrylamide percentage and the buffer composition.

Equilibration is not necessary (i) when the same buffer is used for both electrophoresis and transfer (for example, native gel transfers), or (ii) when using rapid semi-dry transfer systems such as the Trans-Blot® Turbo™ system (consult the user manual for the system you are using).

TABLE OF CONTENTS

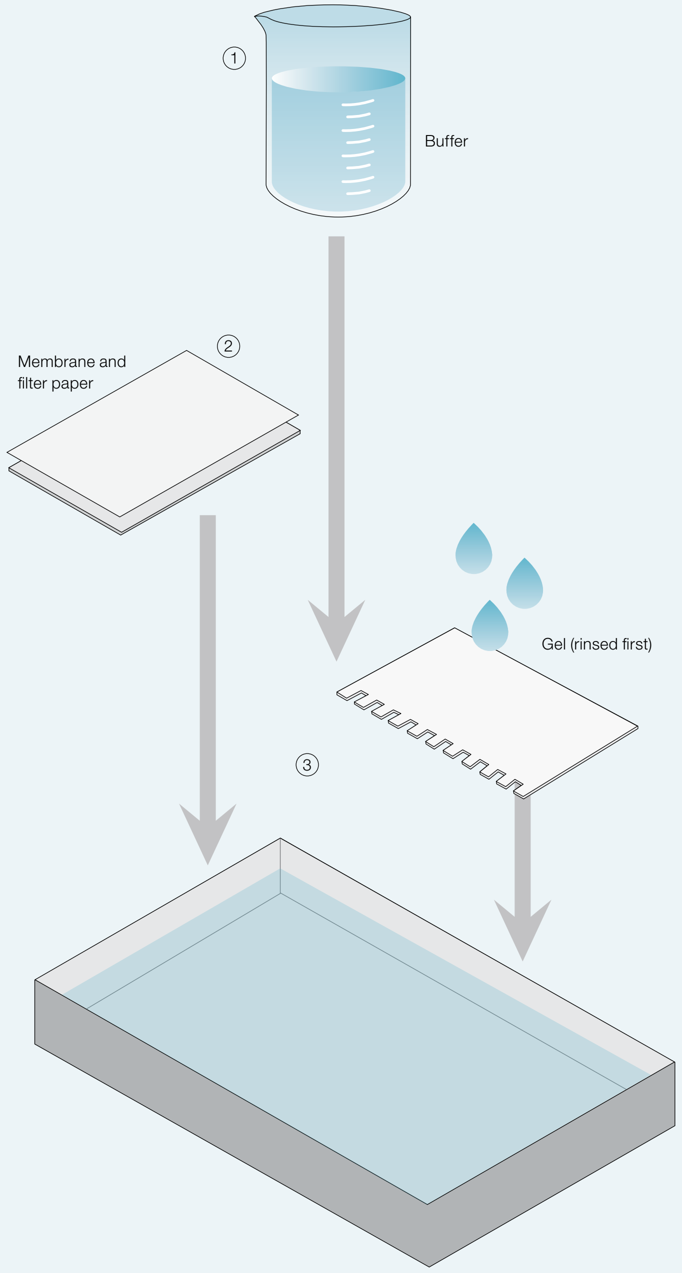

1. Prepare transfer buffer. Refer to Table 2.2 or the instruction manual for the transfer cell you are using for the buffer requirements of each transfer unit. Add 200 ml of buffer per gel for equilibration of gels and transfer materials.

2. Determine the proper membrane for the specific experiment. Select a precut membrane and filter papers or cut the membrane and filter paper to match the size of the gel.

3. Equilibate (if necessary). Wet and equilibrate membranes in transfer buffer for at least 5 min. For PVDF membranes, wet in 100% methanol for ~1 min prior to equilibration in transfer buffer. Rinse gels in diH_2O and equilibrate in transfer buffer for 15 min.

Protocols

Electrophoretic Transfers

Tank Blotting Procedure Part I

Prepare the Gel and Membrane Sandwich

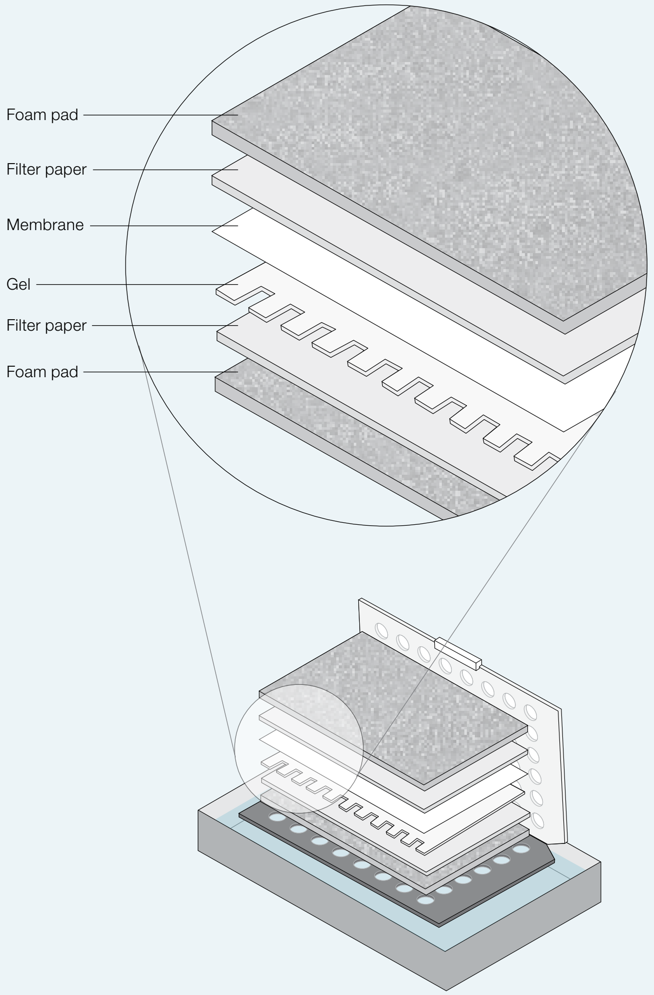

Images and material shown are based on Bio-Rad tank blotting products. Materials may differ based on your blotting apparatus manufacturer.

1. Open a gel holder cassette and submerge the cathode (black) side in transfer buffer.

2. Wet a foam pad in transfer buffer and place it on the submerged side of the cassette.

3. Wet a piece of filter paper in transfer buffer and place it on top of the foam pad. Use a blot roller to remove trapped air.

4. Place the equilibrated gel on top of the filter paper. If needed, gently use a blot roller to remove trapped air.

5. Place the equilibrated membrane on top of the gel. Use a blot roller to remove trapped air.

6. Wet a second piece of filter paper in transfer buffer and place it on top of the membrane. Again, roll to remove trapped air.

7. Soak a foam pad in transfer buffer and place it on top of the filter paper, then close and lock the cassette.

Protocols

Electrophoretic Transfers

Tank Blotting Procedure Part II

Assemble the Tank and Program the Power Supply

TIPS

Stirring during transfer helps maintain uniform conductivity and temperature. Failure to properly control buffer temperature may result in poor transfer and poses a potential safety hazard.

Electrophoretic transfer entails large power loads and consequently, heat generation. The tanks are effective thermal insulators and limit the efficient dissipation of heat; thus, simply placing the tank in the cold room is not enough to remove all of the heat generated during transfer.

Effective cooling required for high-intensity field transfers and recommended for long, unsupervised runs can be provided using the cooling coil or Bio-Ice™ units included with your transfer device.

Evaluate transfer efficiency at various field strengths (V/cm), staying within the recommendations for each instrument.

For transfers using high power, monitor the transfer carefully and use cooling as needed.

Perform a test run to determine the time required for complete transfer. Times may vary from 15 min to overnight, depending on many factors, including the power setting, gel percentage, and the size, shape, and charge of the protein.

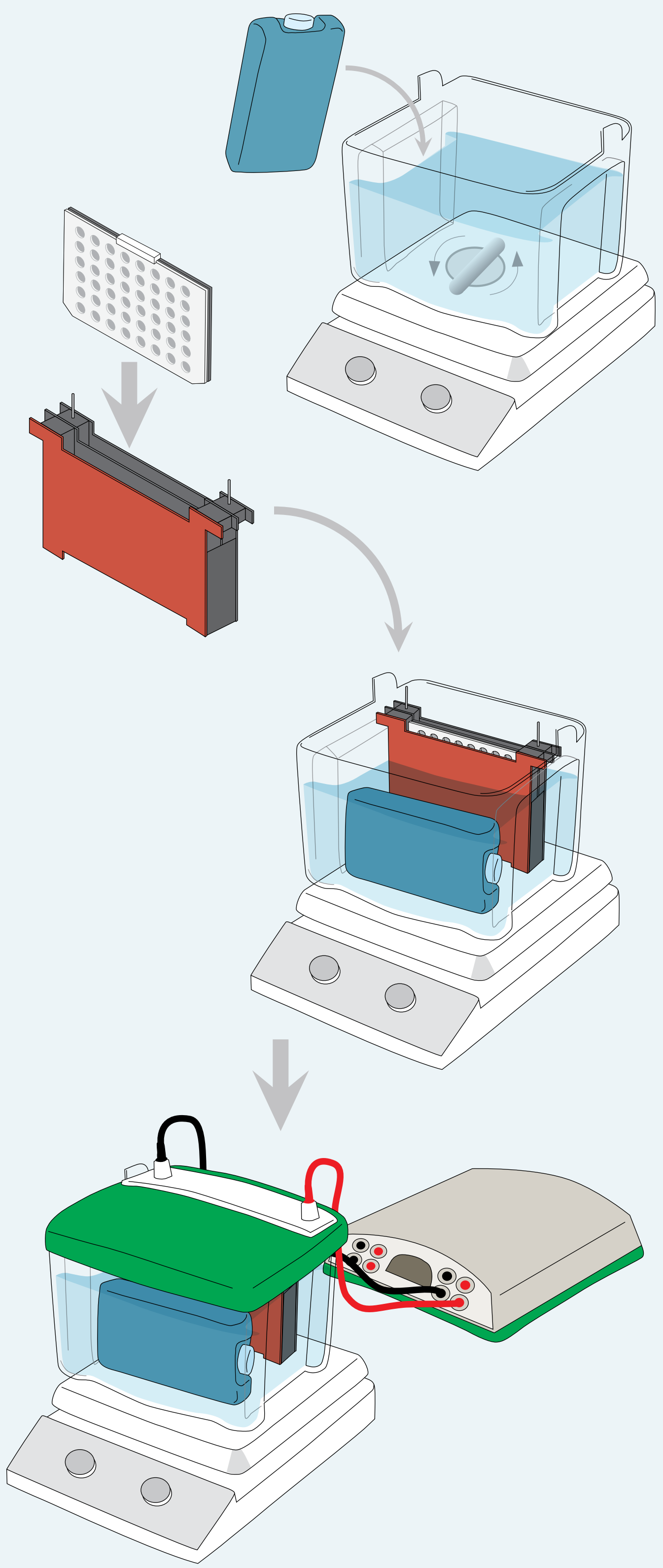

1. Place the transfer tank on a magnetic stir plate and fill the tank halfway with transfer buffer.
2. Add a stir bar and begin stirring. If needed, begin cooling the transfer tank with an ice pack or cooling coil.
3. Insert the gel holder cassette into the blotting module latch side up, with the black side of the cassette facing the black side of the blotting module. Repeat with additional casettes if needed. Place blotting module with cassettes in the tank.
4. Add transfer buffer to the tank until the buffer level reaches the fill line.
5. Place the lid on top of the cell, making sure that the color-coded cables on the lid are attached to the proper electrode cards.
6. Connect the cables to the power supply, making sure to match the colors on the cables to those on the power supply inputs. Program the power supply and start the run.

   Note: Reversing field polarity by switching cable colors will cause irreversible damage to the electrodes.
7. Upon completion of the run, remove the cassettes and disassemble the gel and membrane sandwich.

Protocols

Electrophoretic Transfers

Semi-Dry Blotting Procedure

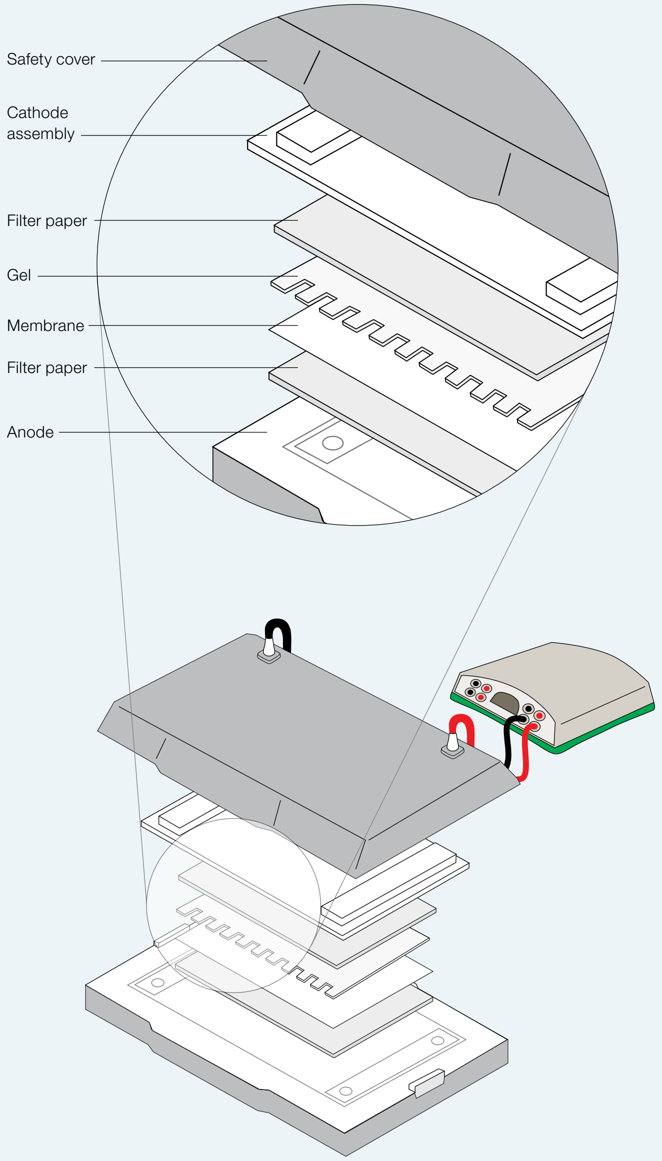

1. Soak the filter paper in transfer buffer (two sheets of extra thick or six sheets of thick filter paper).
2. Remove the safety cover and stainless-steel cathode assembly and place the presoaked filter paper (one sheet of extra thick or three sheets of thick paper) onto the platinum anode. Remove air trapped between the paper and the anode using a blot roller.
3. Carefully place the equilibrated membrane on top of the filter paper. Roll out any air trapped between the transfer materials.
4. Gently place the equilibrated gel on top of the membrane and roll out trapped air.
5. Place filter paper (one sheet of extra thick or three sheets of thick) onto the gel and roll out trapped air.
6. Carefully place the cathode assembly onto the transfer stack and then place the safety cover back onto the unit.
7. Connect the cables to the power supply, making sure to match the colors on the cables to those on the power supply inputs. Program the power supply (see Chapter 4) and start the run.
8. Upon completion of the run, remove the cathode assembly and disassemble the gel and membrane sandwich. If needed, rinse the gel briefly with diH_2O.

TIPS

Evaluate transfer efficiency at various field strengths (V/cm), staying within the recommendations for each instrument.

Bio-Rad semi-dry systems place the anode on the bottom electrode. If using a different system, consult the owner's manual for the proper orientation of the gel and membrane.

For transfers using high power, monitor the transfer carefully and use cooling as needed.

Perform a test run to determine the time required for complete transfer. Times may vary from 15 min to 1 hr, depending on many factors, including the power setting, and the size, shape, and charge of the protein.

TABLE OF CONTENTS

Protocols

Electrophoretic Transfers

Trans-Blot® Turbo™ Blotting Procedure

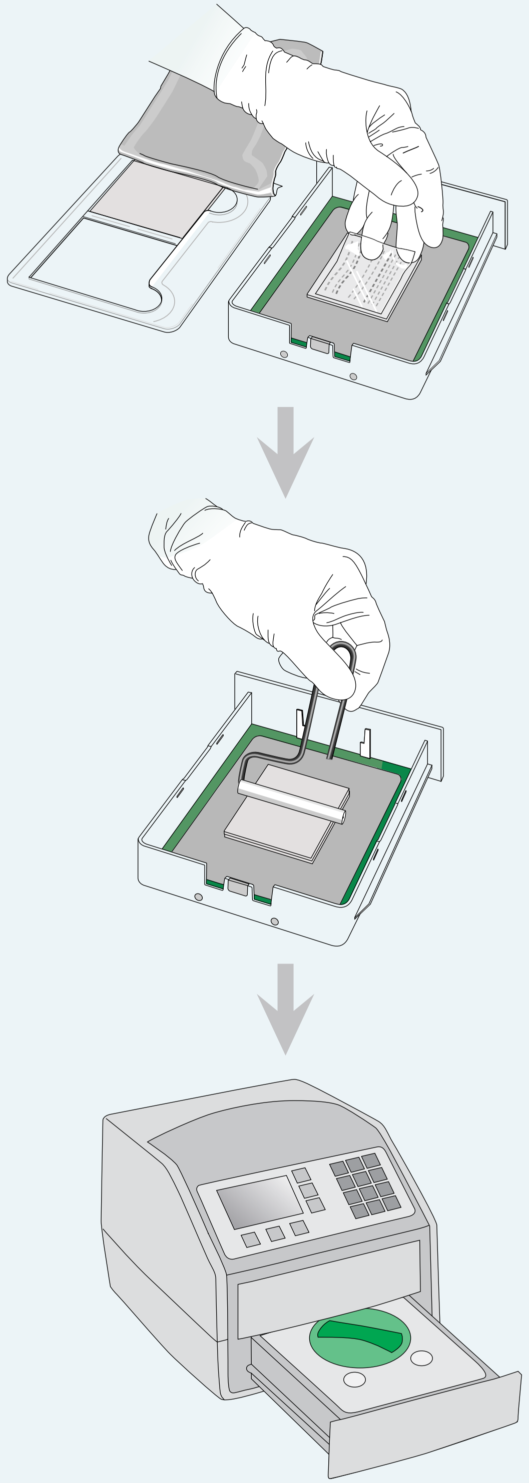

1. After gel electrophoresis, open the transfer pack that matches your gel (mini or midi) and place the anode stack on the cassette base. Place single mini or midi stacks in the middle of the cassette base; two mini gels can be placed on a midi stack with each gel bottom facing the center. Use the blot roller to remove any air trapped between the pad and membrane. No equilibration is required.

2. Place the gel on the anode stack (which includes the membrane) and the cathode stack on the gel. Roll to remove trapped air.

3. Place the lid on the cassette and lock it into place by turning the green knob clockwise. Ensure the locking pins fully engage their locking slots.

4. Turn the instrument on and slide the cassette into either cassette bay. If using two cassettes, each must be using the same size transfer pack.

5. Start the transfer. With the cassette inserted into the instrument, press TURBO and select the gel type. Press A:RUN to start the top tray, B:RUN for the bottom tray. Select LIST to select a preprogrammed protocol or NEW to create and run a new protocol.

6. At the end of the run, RUN COMPLETE appears on the screen. Remove the cassette from the instrument and unlock the lid. (Caution: the cassette may be warm.) Remove the membrane from the transfer sandwich and discard the remaining transfer pack materials.

Protocols

Microfiltration

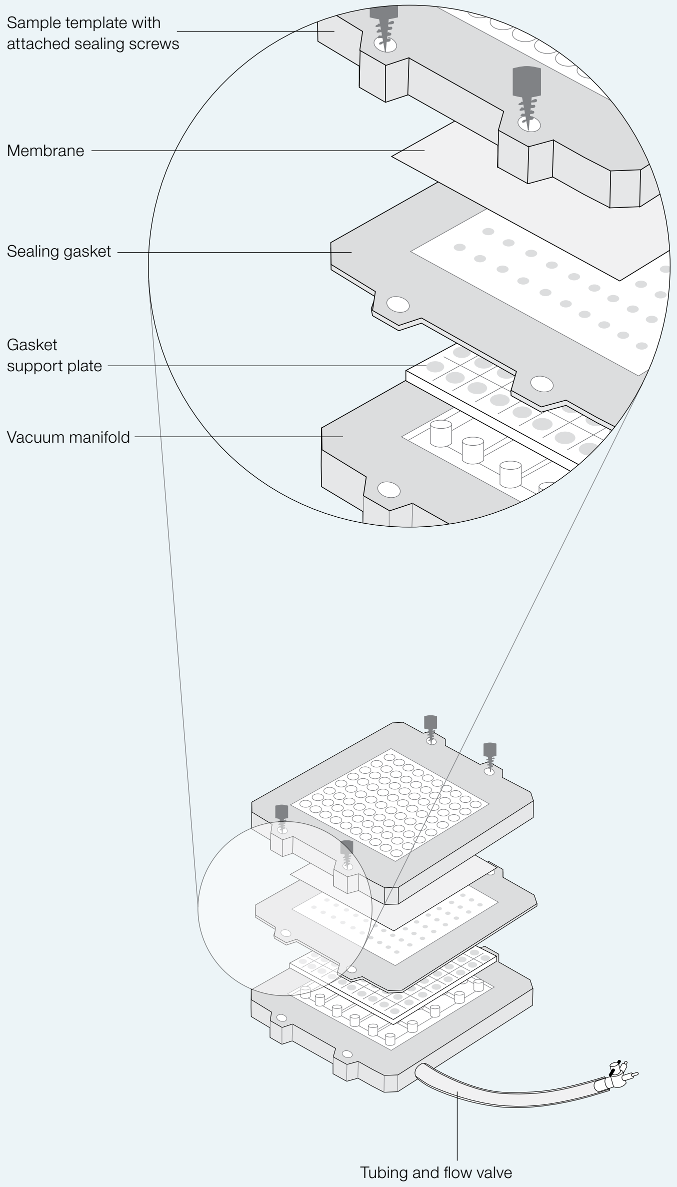

1. Prepare the samples. For best results, filter or centrifuge samples to remove particulate matter that might restrict the flow of solutions through the membrane.

2. Assemble the unit as shown in the illustration at left.

3. Adjust the flow valve so the unit is exposed only to atmospheric pressure. Add samples. Remove any air bubbles trapped in the wells by gently pipetting the solution up and down.

4. For best sample binding, the entire sample should be filtered by gravity flow without vacuuming.

5. The membrane may be washed by adding a volume of buffer equal to the sample volume in each well.

6. After application of sample, the membrane may be blocked and then probed for the protein of interest. Refer to the product instruction manual for detailed instructions.

7. To remove the membrane, leave the vacuum on while loosening the screws and removing the sample template. Then turn off the vacuum and remove the membrane.

TIPS

Application of the Vacuum

During the assay, do not leave the unit with the vacuum on. This may dehydrate the membrane and may cause halos around the wells.

Flow Valve

Proper positioning of the flow valve relative to the level of the apparatus is important for proper drainage. The speed of drainage is determined by the difference in hydrostatic pressure between the fluid in the sample wells and the opening of the flow valve that is exposed to the atmosphere. When the flow valve is positioned below the sample wells, proper drainage may be achieved.

If a prolonged or overnight incubation is desired, adjust the flow valve so that the vacuum manifold is closed off from both the vacuum source and atmosphere before applying the samples. In this configuration, solutions will remain in the sample wells with less than a 10% loss of volume during extended incubations.

To apply a gentle vacuum to the apparatus, adjust the flow valve so that it is open to the atmosphere, the vacuum source, and the vacuum manifold while the vacuum is on. Then, use a finger to cover the valve port that is exposed to the atmosphere. The pressure of your finger on the valve will regulate the amount of vacuum reaching the manifold.

TABLE OF CONTENTS

Protocols

Blot Stripping and Reprobing

Based on Legocki and Verma 1981

TIPS

This protocol (based on Legocki and Verma 1981) uses low pH to gently remove antibody from the membrane. The protocol removes little of the sample proteins but may not remove all antibodies with high affinities for their targets.

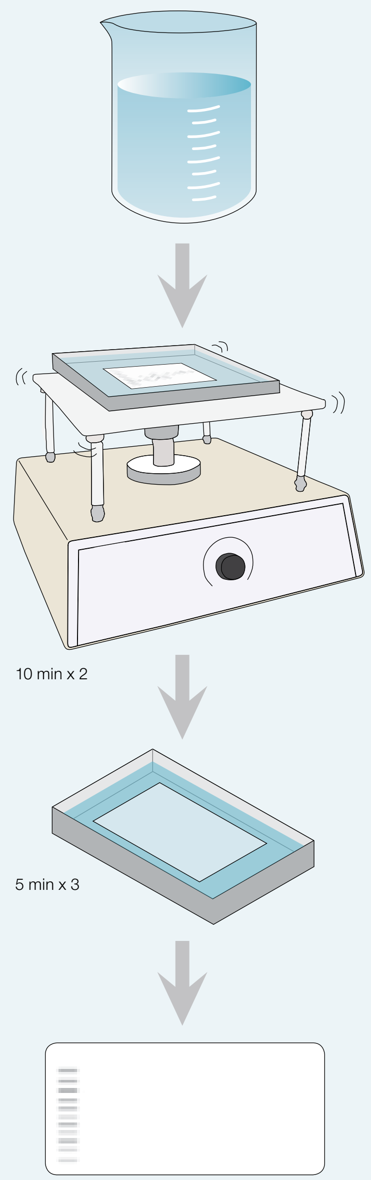

1. Prepare acidic glycine stripping buffer (0.1 M glycine, 20 mM magnesium acetate, 50 mM KCl, pH 2.2; for recipe, see page 60).
2. Add enough acidic glycine stripping buffer to completely cover the developed membrane and incubate at room temperature for 10 min with gentle agitation.
3. Repeat step 2 with fresh acidic glycine stripping buffer.
4. Wash the blot three times in TBS-T for 5 min each with gentle agitation.
5. Test for complete removal of primary antibody by reprobing with only the secondary antibody and redeveloping. No signal should be detectable. Re-block the membrane and proceed to the next detection protocol.

Protocols

Total Protein Detection

General protocols are described below. For more details, refer to the instruction manual for the stain you are using.

SYPRO Ruby Stain

Consult the SYPRO Ruby Protein Stains Instruction Manual (bulletin 4006173) for complete instructions. Membranes stained with SYPRO Ruby protein blot stain are best preserved by allowing membranes to air dry.

1. *Pretreat* — after electroblotting proteins to a nitrocellulose membrane, completely immerse the membrane in 7% acetic acid and 10% methanol, and incubate for 15 min.
2. *Wash* — wash the membrane in diH_2O four times for 5 min each.
3. *Stain* — completely immerse the membrane in SYPRO Ruby protein blot stain for 15 min.
4. *Wash* — wash the membrane in diH_2O four to six times for 1 min each. Monitor the membrane periodically using UV epi-illumination to determine the level of background fluorescence.
5. *Image* — image using epi UV or green excitation.
6. Proceed to immunodetection (if needed).

Ponceau S Stain

1. *Stain* — incubate the membrane for 1–2 min in the staining solution.
2. *Destain* — destain the membrane in water until the background clears. Ponceau S staining is reversible and will disappear after extended destains in diH_2O.
3. *Wash* — rinse the membrane in TBS or deionized H_2O before drying.
4. Proceed with immunodetection.

Colloidal Gold Total Protein Stain

Consult the Bio-Rad enhanced colloidal gold total protein detection kit manual for complete instructions.

1. *Wash* — following transfer or protein application, wash the membrane three times for 20 min in high-Tween TBS (TBS-T with 0.3% Tween 20).
2. *Water wash* — wash the membrane for 2 min in deionized H_2O to remove interfering buffer salts.
3. *Stain* — incubate the membrane with colloidal gold stain, completely covering the blot. Incubation times will vary with the concentration of protein present on the membrane. Most bands will be visible in 1–2 hr. If increased sensitivity is required, continue the assay using the gold enhancement procedure.

TABLE OF CONTENTS

Protocols

Immunodetection

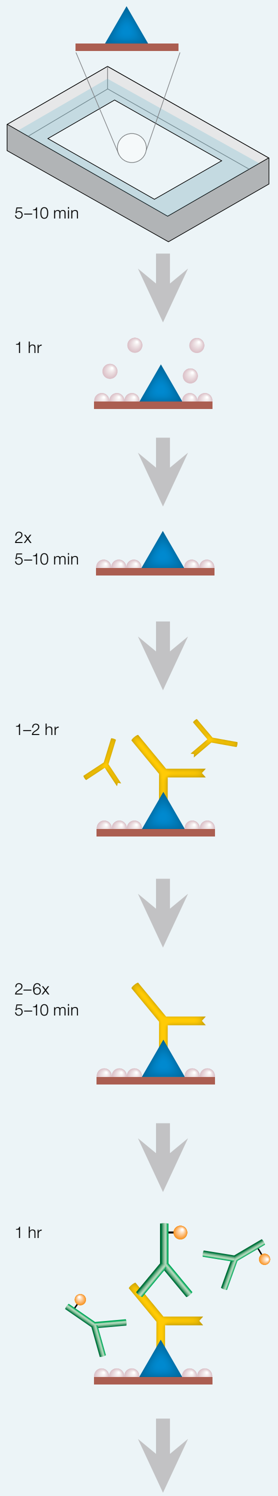

1 *Wash* — following transfer or protein application, wash the membrane for 5–10 min in TBS.

2 *Block* — incubate the membrane for 1 hr in blocking solution.

3 *Wash* — wash the membrane twice in TBS-T, 5–10 min per wash.

4 *Primary antibody* — dilute the antibody in antibody dilution or blocking solution (refer to the instructions for the antibody for the recommended final concentration). Incubate the membrane for 1–2 hr in the primary antibody solution with gentle agitation.

5 *Wash* — wash the membrane 2–6 times in TBS-T, 5–10 min per wash.

6 *Antibody conjugate* — dilute the conjugate in TBS-T (refer to the instructions for the conjugate for the recommended final concentration). Incubate the membrane for 1 hr in the enzyme conjugate solution with gentle agitation.

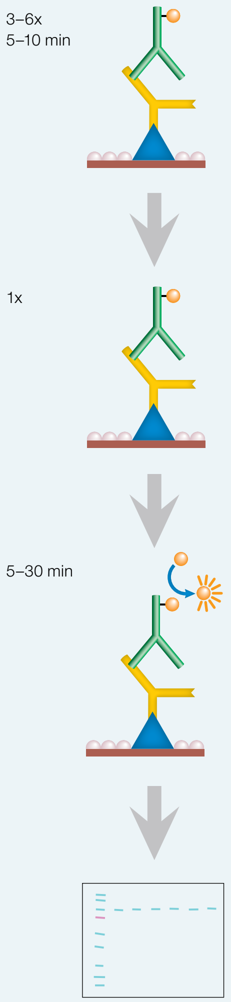

7 *Wash* — wash the membrane 3–6 times in TBS-T, 5–10 min per wash.

8 *Final wash* — wash the membrane in TBS to remove the Tween 20 from the membrane surface prior to blot development and imaging.

9 *Signal development* — for colorimetric development, add detection substrate and incubate for 5–30 min depending on the specific reagents used. For chemiluminescence/fluorescence, see next page.

10 *Image, dry, and store* — image the blot on a CCD laser-based imager, or expose to X-ray film or instant photographic film. Develop the film according to the manufacturer's instructions.

Notes for Multiplex Detection

Primary antibodies must be from different host species in order to be detected in separate channels (for example, mouse and rabbit).

Test primary antibodies individually to determine the banding pattern for each on the membrane prior to multiplexing. Once known, these antibodies can be diluted and incubated simultaneously.

Secondary antibody conjugates should possess specificity to the primary antibody host species (for example, goat anti-mouse and goat anti-rabbit).

Secondary antibody conjugates should be highly cross-absorbed against other species to minimize cross-reactivity.

Protocols

Immunodetection

Notes for Chemiluminescence Detection

Follow steps 1–8 of the immunodetection assay, except use more stringent washes (steps 5 and 7). Wash the membrane six times for 10 min each at these steps, with strong agitation and a large volume of buffer to reduce background. Then follow below for step 9:

A Place the membrane protein-side up on a clean piece of plastic wrap or a plastic sheet protector.

B Add chemiluminescent substrate solution. Use at least 0.1 ml per cm^2 of membrane (about 6 ml for a standard 7 x 8.5 cm gel).

C Incubate the membrane for 3–5 min in the chemiluminescent substrate solution.

D Drain excess liquid from the blot and seal the membrane in a bag or sheet protector.

E Image the blot on a CCD imager such as a ChemiDoc™ system, or expose to X-ray film (for example, Kodak XAR or BioMax) or instant photographic film, such as Polaroid Type 667 or 612. Typical exposure times are 30 sec to 5 min. Develop the film according to the manufacturer's instructions.

Notes for Fluorescence Detection

Follow steps 1–8 of the immunodetection assay. Imaging of most fluorescent dye conjugates (Cy, Dylight, Alexa Fluor, and IRDye dyes) can be performed on wet or dry membranes. Imaging of fluorescent protein conjugates (phycoerythrin, allophycocyanin) should be performed on wet membranes for maximum sensitivity.

Note for Protein G-HRP Detection

Follow steps 1–4 on page 56. For step 5 (wash), use CBS-T instead of TBS-T and then continue with steps 6–10.

Notes for Amplified Opti-4CN™ Detection

Follow steps 1–8 of the immunological assay on page 56. Then:

A Incubate the membrane in diluted BAR for 10 min.

B Wash the membrane 2–4 times in 20% DMSO/PBST for 5 min each time.

C Wash 1–2 times in PBST for 5 min. each time.

D Incubate the membrane and diluted streptavidin-HRP for 30 min.

E Wash the membrane twice in PBST for 5 min each time.

F Continue with steps 9–10.

Notes for Amplified AP Detection

Follow steps 1–5 of the immunodetection assay on page 56. Then:

A Incubate the membrane for 1–2 hr in biotinylated secondary antibody solution.

B While the blot is incubating in the biotinylated antibody solution, prepare the streptavidin-biotinylated AP complex. Allow the complex to form for 1 hr at room temperature.

C Wash the membrane twice in TBS-T, 5–10 min per wash.

D Incubate the membrane for 1–2 hr in the streptavidin complex solution.

E Continue with steps 7–10.

TIPS

If kept wet, blots using HRP or AP conjugates can be stored for several days prior to development and imaging. Leave blot in TBS, or place membrane between two pieces of filter paper soaked in TBS, and place in a sealable container.

Transfer Buffer Formulations

The following buffers are recommended for use with all of Bio-Rad's electrophoretic transfer cells. Care should be taken when preparing these buffers because incorrect formulation can result in a current that exceeds the recommended conditions.

Use only high-quality, analytical grade methanol. Impure methanol can increase transfer buffer conductivity and yield a poor transfer.

In many cases, ethanol can be substituted for methanol in the transfer buffer with minimal impact on transfer efficiency. Check this using your samples.

Do not reuse transfer buffer since the buffer will likely lose its ability to maintain a stable pH during transfer.

Do not dilute transfer buffers below their recommended levels since this decreases their buffering capacity.

Do not adjust the pH of transfer buffers unless specifically indicated. Adjusting the pH of transfer buffers can result in increased buffer conductivity, manifested by higher initial current output and decreased resistance.

Increasing SDS in the transfer buffer increases protein transfer from the gel but decreases binding of the protein to nitrocellulose membrane. PVDF membrane can be substituted for nitrocellulose when SDS is used in the transfer buffer.

Addition of SDS increases the relative current, power, and heating during transfer, and may also affect antigenicity of some proteins.

Increasing methanol in the transfer buffer decreases protein transfer from the gel and increases binding of the protein to nitrocellulose membrane.

1x Towbin Buffer, 1 L

25 mM Tris, 192 mM glycine, 20% (v/v) methanol (pH 8.3) (catalog #161-0734, without methanol, 1 L, 10x)

Tris base	3.03 g
Glycine	14.4 g
diH_2O	500 ml
Methanol	200 ml

Adjust volume to 1 L with diH_2O.

The pH will range from pH 8.1 to 8.5 depending on the quality of the Tris, glycine, methanol, and diH_2O.

1x Towbin Buffer with SDS, 1 L

25 mM Tris, 192 mM glycine, 20% methanol (v/v), 0.025–0.1% SDS (pH 8.3)

Add 2.5 to 10 ml 10% SDS to 1 L buffer prepared above.

1x Bjerrum Schafer-Nielsen Buffer, 1 L

48 mM Tris, 39 mM glycine, 20% methanol (pH 9.2)

Tris base	5.82 g
Glycine	2.93 g
diH_2O	500 ml
Methanol	200 ml

Adjust volume to 1 L with diH_2O.

1x Bjerrum Schafer-Nielsen Buffer with SDS, 1 L

48 mM Tris, 39 mM glycine, 20% methanol, 1.3 mM SDS (pH 9.2)

Add 0.0375 g SDS (or 3.75 ml 10% SDS) to 1 L buffer prepared above.

1x CAPS Buffer, 1 L

10 mM 3-(cyclohexylamino)-1-propanesulfonic acid, 10% methanol (pH 11.0)

CAPS	2.21 g
diH_2O	500 ml
Methanol	100 ml

Adjust volume to 1 L with diH_2O.
Measure the pH and adjust as needed with NaOH.

1x Dunn Carbonate Buffer, 1 L

10 mM $NaHCO_3$, 3 mM $NaCO_3$, 20% methanol (pH 9.9)

$NaHCO_3$	0.84 g
$NaCO_3$ (anhydrous)	0.318 g
diH_2O	500 ml
Methanol	200 ml

Adjust volume to 1 L with diH_2O.

0.7% Acetic Acid

Add 7 ml glacial acetic acid to 993 ml diH_2O.

Detection Buffer Formulations

General Detection Buffers

1x Tris-buffered saline (TBS), 2 L
20 mM Tris-HCl, 500 mM NaCl (pH 7.5) (catalog #170-6435, 1 L, 10x)

Tris base	4.84 g
NaCl	58.48 g
diH_2O	1.5 L

Adjust pH to 7.5 with HCl.
Adjust volume to 2 L with diH_2O.

1x TBS-T wash solution, 1 L
20 mM Tris-HCl, 500 mM NaCl, 0.05% Tween 20 (pH 7.5)

0.5 ml Tween 20
1 L TBS

1x Citrate-buffered saline (CBS)
20 mM citrate, 500 mM NaCl (pH 5.5)

Included in Immun-Blot® protein G kits.

CBS-T wash solution, 1 L
20 mM citrate, 500 mM NaCl, 0.05% Tween 20 (pH 5.5)

0.5 ml Tween 20
1 L 1x CBS

1x Blocking solution, 100 ml
1x TBS, 3% (w/v) gelatin
Add 3.0 g gelatin to 100 ml TBS.
Heat to 50°C; stir to dissolve.

or

1x TBS, 3% (w/v) BSA
Add 1.0 g BSA to 100 ml TBS; stir to dissolve.

or

1x TBS, 5% (w/v) nonfat dry milk
Add 5.0 g nonfat dry milk to 100 ml TBS; stir to dissolve.

Note: Gelatin can clog membranes and cut off the vacuum flow of microfiltration units; use an alternative blocking solution with the Bio-Dot® or Bio-Dot SF apparatus.

Note: Nonfat milk is not recommended for avidin/biotin systems as milk contains endogenous biotin and may cross-react with avidin-containing components in the detection system.

Antibody dilution buffer, 200 ml
1x TBS-T, 1% (w/v) gelatin
Add 2.0 g gelatin to 200 ml TBS-T.
Heat to 50°C; stir to dissolve.

or

1x TBS-T, 3% (w/v) BSA
Add 6.0 g BSA to 200 ml 1x TBS-T; stir to dissolve.

or

1x TBS-T, 5% nonfat milk
Add 10.0 g nonfat dry milk to 200 ml 1x TBS-T; stir to dissolve.

Note: Gelatin can clog membranes and cut off the vacuum flow of microfiltration units; use an alternative blocking solution with the Bio-Dot or Bio-Dot SF apparatus.

Note: Nonfat milk is not recommended for avidin/biotin systems as milk contains endogenous biotin and may cross-react with avidin-containing components in the detection system.

Antibody buffer (for chemiluminescence, ImmunStar™ AP only)
1x TBS-T, 0.2% nonfat milk
Add 0.4 g nonfat milk to 200 ml TBS-T; stir to dissolve.

Antibody buffer for protein G-HRP, 100 ml
CBS-T, 1% (w/v) gelatin
Add 1.0 g gelatin to 100 ml CBS-T.
Heat to 50°C; stir to dissolve.

Protein G-HRP conjugate solution, 100 ml
Mix 33 µl protein G conjugate solution in 100 ml 1% gelatin in CBS-T.

Streptavidin-biotinylated AP complex, 100 ml
33 µl streptavidin
100 ml 1x TBS-T
33 µl biotinylated AP

Incubate the complex 1–3 hr at room temperature before use.

Total Protein Staining Buffers and Solutions

Amido black staining solution, 1 L
For nitrocellulose:

Amido black	5 g
Methanol	400 ml

Adjust volume to 1 L with diH_2O.

or

Amido black	5 g
Isopropanol	250 ml
Acetic acid	100 ml

Adjust volume to 1 L with diH_2O.

For PVDF:

Amido black	1 g
Methanol	400 ml
Acetic acid	100 ml

Adjust volume to 1 L with diH_2O.

Amido black destain solution, 1 L
For nitrocellulose:

Isopropanol	250 ml
Acetic acid	100 ml

Adjust volume to 1 L with diH_2O.

For PVDF:

Methanol	400 ml
Acetic acid	100 ml

Adjust volume to 1 L with diH_2O.

Coomassie Blue R-250 staining solution, 1 L

Coomassie Blue R-250	1 g
Methanol	400 ml
Acetic acid	100 ml

Adjust volume to 1 L with diH_2O.

Coomassie Blue R-250 destaining solution, 1 L

Methanol	400 ml
Acetic acid	100 ml

Adjust volume to 1 L with diH_2O.

Ponceau S staining solution

Ponceau S	2 g
Trichloracetic acid (TCA)	30 g
Sulfosalicylic acid	30 g
diH_2O	80 ml

Ponceau S destaining solution

1% acetic acid or 1x PBS

SYPRO Ruby blot pretreatment solution

Acetic acid	70 ml
Methanol	100 ml
diH_2O	830 ml

Colloidal gold blot staining solution

Use TBS-T wash solution (see page 59).

Substrate Buffers and Solutions

HRP Substrate Buffers

4-(chloro-1-naphthol)	4CN	60 mg
	Methanol	20 ml
	Protect mixture from light	
	3% H_2O_2	600 µl
	Substrate solution	100 ml
	Mix the two solutions together. Use immediately. Alternatively, use HRP conjugate substrate solution in kit format.	
HRP conjugate substrate solution	Dissolve contents of premixed color development buffer in diH_2O to 1 L	
	Color reagent B	600 µl
	Development buffer	100 ml
	HRP color reagent A	20 ml
	Use immediately.	
Diaminobenzidine (DAB)	DAB	50 mg
	TBS	100 ml
	3% H_2O_2	100 µl
	Use immediately.	

AP Substrate Buffers

AP color development buffer	$MgCl_2$	0.233 g
	Tris base	12.1 g
	diH_2O	800 ml
	Adjust pH to 9.5 with HCl; adjust volume to 1 L with diH_2O.	

5-bromo-4-chloroindolyl phosphate/nitroblue tetrazolium (BCIP/NBT)

Dimethylformamide	0.7 ml
diH_2O	0.3 ml
NBT	30 mg
Dimethylformamide	1 ml
BCIP	15 mg

Add both solutions to 100 ml AP color development buffer. Use immediately. Alternatively, use AP conjugate substrate solution in kit format.

Immun-Star™ AP substrate solution (kit format)

Use 5 ml chemiluminescent substrate per 100 cm2.

For nitrocellulose membrane blots:	Add 500 µl enhancer reagent to 10 ml Immun-Star chemiluminescent substrate Store at 4°C for up to 1 week.
For PVDF membrane blots:	Immun-Star AP generates a very fast light signal on PVDF membrane; therefore, the use of an enhancer is not necessary. The substrate is provided ready to use.

Clarity and Clarity Max western ECL substrate solutions

For nitrocellulose and PVDF membrane blots:	A 1:1 mixture of luminol/ enhancer to peroxide buffer is recommended. Use 10 ml per 100 cm2 of membrane (12 ml for one 8.5 x 13.5 cm Criterion™ blot).

Stripping Buffer

Acidic glycine stripping buffer

Glycine	7.5 g
$Mg(CH_3COO)_2{\cdot}4H_2O$	4.3 g
KCl	3.7 g
diH_2O	800 ml

Adjust pH to 2.2 with HCl.

diH_2O	to 1 L

TABLE OF CONTENTS

TABLE OF CONTENTS

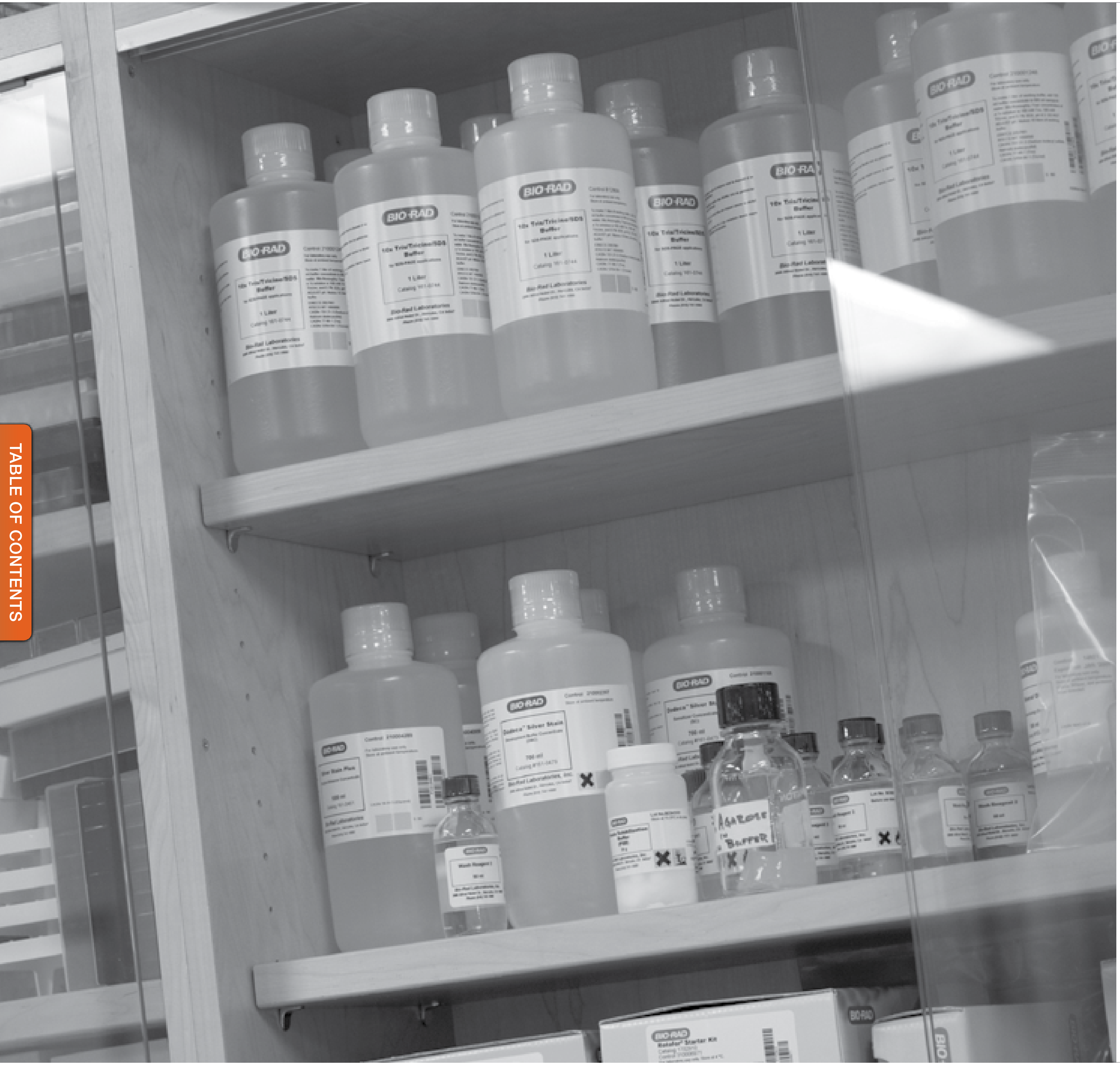

PART 3

Troubleshooting

The protocols included in this guide are general recommendations for transferring and detecting proteins on blots. To help you optimize your protein blotting results, this chapter includes troubleshooting tips to improve both transfer and detection.

Transfer

Electrophoretic Transfer

Problem	Cause	Solution
Poor electrophoretic transfer; bands appear weak on blot (ensure proteins have been transferred by staining both the gel and blot with a total stain. For example, stain the gel with Bio-Safe™ Coomassie or SYPRO Ruby stain, and stain the blot with Ponceau S stain). Alternatively, one could use stain-free technology and PVDF membranes	Power conditions were inadequate or transfer time too short	• Increase the transfer time (thicker gels require longer transfer times)<br>• Check the current at the beginning of the run; it may be too low for a particular voltage setting, indicating incorrect buffer composition. See the power guidelines for specific applications in Chapter 4<br>• Use high-intensity blotting<br>• Use a power supply with a high current limit. If an incorrect power supply is used, it is possible to not reach the set voltage if the current of the power supply is at its maximum limit
	Power conditions were too high or transfer time too long (proteins may transfer through the membrane and into the filter paper)	• Shorten transfer time<br>• Reduce transfer voltage<br>• See "Overall poor binding to the membrane" on page 65 for hints on how to improve binding
	Transfer buffer was incorrect or prepared incorrectly	• Prepare fresh transfer buffer (never reuse transfer buffer)
	Proteins moved in the wrong direction (the gel/membrane sandwich may have been assembled in the wrong order, the cassette inserted in the tank in the wrong orientation, or polarity of the connections may be incorrect)	• Check the gel/membrane sandwich assembly<br>• Check the assembly of the transfer cell<br>• Check the polarity of the connections to the power supply
	The charge-to-mass ratio is incorrect (native transfers)	• Use a more basic or acidic transfer buffer to increase protein mobility. A protein near its isoelectric point (pI) will transfer poorly (buffer pH should be 2 pH units higher or lower than the pI of the protein of interest for optimal transfer efficiency)
	Protein precipitated in the gel	• Use SDS in the transfer buffer. SDS can increase transfer efficiency but it can also reduce binding efficiency to nitrocellulose and affect reactivity of some proteins with antibodies<br>• Reduce or eliminate the alcohol in the transfer buffer
	The power supply circuit is inoperative or an inappropriate power supply was used	• Check the fuse<br>• Make sure the voltage and current output of the power supply match the needs of the blotting instrument<br>• Check the output capacity of the power supply
	The gel percentage was too high (decreasing %T or %C increases gel pore size and increases transfer efficiency)	• Reduce %T (total monomer) or %C (crosslinker). Using 5%C (with bis-acrylamide as the crosslinker) produces the smallest pore size
Regions of poor protein binding on the blot	The membrane was not uniformly wet before transfer	• Ensure that membranes are uniformly wet before transfer<br>• Because of the hydrophobic nature of PVDF, the membrane must be completely soaked in methanol prior to equilibration in aqueous transfer buffer. A completely wet PVDF membrane has a gray, translucent appearance
	Buffer tank not filled to correct level	• Completely fill transfer tank with buffer. Transfer tank must contain sufficient buffer to entirely cover blot area
Swirls or missing bands; bands appear diffuse on the blot	Contact between the membrane and the gel was poor; air bubbles or excess buffer remain between the blot and gel	• Carefully move the roller over the membrane in both directions until air bubbles or excess buffer are removed from between gel and membrane and complete contact is established<br>• Use thicker filter paper in the gel/membrane sandwich<br>• Replace the foam pads. Pads compress and degrade with time and will not hold the membrane to the gel

Problem	Cause	Solution
White spots on membrane	The membrane was not properly wetted or had dried out	• White spots on the nitrocellulose membrane indicate dry areas where protein will not bind. If wetting does not occur immediately by immersion of the sheet in transfer buffer, heat distilled water until just under the boiling point and soak the membrane until completely wet. Equilibrate in transfer buffer until ready for use<br>• White spots on the PVDF membrane indicate areas where the membrane was either improperly prewetted or allowed to dry out. Because of the hydrophobic nature of PVDF, the membrane must be prewet in methanol prior to equilibration in aqueous transfer buffer. Once wet, do not allow membrane to dry out. If the membrane dries, rewet in methanol and re-equilibrate in TBS-T (this may adversely effect downstream detection processes)
Broad or misshapen bands	Poor gel electrophoresis	• Artifacts of electrophoresis may occur as a result of poor gel polymerization, inappropriate running conditions, contaminated buffers, sample overload, etc. Consult your manual for more details
Gel cassette pattern transferred to blot	Foam pads are contaminated or too thin	• Clean or replace the foam pads
	Excessive amounts of protein were loaded on the gel or too much SDS was used in the transfer buffer. Proteins can pass through the membrane without binding and recirculate through tank blotting systems	• Reduce the amount of protein on the gel<br>• Reduce the amount of SDS in the transfer buffer<br>• Add a second sheet of membrane to bind excess protein
	The transfer buffer was contaminated	• Prepare fresh transfer buffer
Overall poor binding to the membrane	Methanol in the transfer buffer is restricting elution	• Reduce the amount of methanol. This may improve transfer efficiency of proteins from the gel but it also may decrease binding to nitrocellulose membranes; 20% methanol is generally optimal for protein binding
	SDS in the transfer buffer reduces the binding efficiency of proteins	• Reduce or eliminate SDS from the transfer buffer
	Proteins passed through the membrane. Proteins <15 kD may show decreased binding to 0.45 µm membranes	• Use PVDF or 0.2 µm nitrocellulose (smaller pore size)<br>• Decrease the voltage if using the high-intensity option<br>• Place an additional membrane in the gel sandwich to detect proteins that are being transferred through the membrane

Microfiltration

Problem	Cause	Solution
Leakage or cross-well contamination	The instrument was assembled incorrectly	• Retighten the screws under vacuum following initial assembly to form a proper seal
	The membrane was not rehydrated after assembly	• Rehydrate the membrane prior to loading samples<br>• Apply vacuum only until solutions are removed from the sample wells, then disconnect the vacuum
Uneven or no filtration	The membrane became clogged with particulates	• Centrifuge samples or filter solutions prior to application to remove particulates
	The flow valve was positioned higher than the apparatus	• Position the flow valve lower than the level of the sample wells or drainage will not occur
	Bubbles obstructed the flow of liquid	• Use a needle to carefully break any bubbles, being careful not to puncture the membrane<br>• Pipet liquid up and down to dislodge the bubbles
	Improper blocking or antibody buffers were used	• Gelatin clogs the membrane; substitute BSA or Tween 20 for gelatin in the detection procedure
	Fluid pressure was not uniform	• Seal off unused wells or add solution to unused wells

TABLE OF CONTENTS

TABLE OF CONTENTS

Problem	Cause	Solution
Halos around the wells	The membrane was not rehydrated after assembly	• Rehydrate the membrane prior to loading samples<br>• Apply vacuum only until solutions are removed from the sample wells, then disconnect the vacuum
	Too much protein was loaded, overloading the capacity of the membrane	• Determine optimum loading conditions by analyzing serial dilutions of samples
	The blocking step was too short	• Use a blocking step that is as long as the longest incubation period
	Loading volume was too low	• The meniscus contacted the center of the well, causing uneven distribution of protein sample. The minimum loading volume is 100 µl

Detection

Immunodetection

Problem	Cause	Solution
Overall high background	Blocking was incomplete	• Increase the concentration of blocker<br>• Increase the duration of the blocking step<br>• Use a different blocking agent
	Blocker was impure. NFDM is not pure. The blocker may be contaminated with material that nonspecifically binds probes	• Use a pure protein such as BSA or casein as a blocker
	Wash protocols were insufficient	• Increase the number, duration, or stringency of the washes<br>• Include progressively stronger detergents in the washes; for example, SDS is stronger than Nonidet P-40 (NP-40), which is stronger than Tween 20<br>• Include Tween 20 in the antibody dilution buffers to reduce nonspecific binding
	The blot was left in the enzyme substrate too long (colorimetric detection)	• Remove the blot from the substrate solution when the signal-to-noise level is acceptable, and immerse in diH_2O
	Contamination occurred during electrophoresis or transfer	• Discard and prepare fresh gels and transfer solutions<br>• Replace or thoroughly clean contaminated foam pads if a tank blotter was used
	Excessive amounts of protein were loaded on the gel or too much SDS was used in the transfer buffer. Proteins can pass through the membrane without binding and recirculate through a tank blotting system	• Reduce the amount of protein on the gel or SDS in the transfer buffer<br>• Add a second sheet of membrane to bind excess protein
	The primary or secondary antibody was too concentrated	• Increase antibody dilutions<br>• Perform a dot-blot experiment to optimize working antibody concentration
	Incubation trays were contaminated	• Clean the trays or use disposable trays
Nonspecific reactions between bound proteins and probes	The primary or secondary antibody is contaminated with nonspecific IgG or with IgG cross-reactive among species	• Use purified IgG primary antibody fractions and affinity-purified blotting-grade cross-adsorbed secondary antibody
	Monoclonal antibodies reacted nonspecifically with SDS-denatured proteins	• Compare the binding of other monoclonal or polyclonal antibodies<br>• Blot native proteins as a comparison
	Nonspecific interactions are occurring due to ionic associations. For example, avidin, a glycosylated protein, may bind to more acidic proteins on blots	• Increase the ionic strength of the incubation buffers<br>• Increase the number, duration, or stringency of the washes<br>• Include progressively stronger detergents in the washes; for example, SDS is stronger than Nonidet P-40 (NP-40), which is stronger than Tween 20<br>• Include Tween 20 in the antibody dilution buffers to reduce nonspecific binding

Problem	Cause	Solution
No reaction or weak signal	The sample load was insufficient	• Increase the amount of protein applied<br>• Concentrate the sample prior to loading
	The detection system is not working or is not sensitive enough	• Use a more sensitive assay system<br>• Include proper positive and negative control antigen lanes to test for system sensitivity; consult manual
	Proteins may be washed from the membrane during assays	• Reduce the number of washes or reduce the stringency of washing conditions during subsequent assay steps
	Antigen binding to the membrane was insufficient	• Stain the blot after transfer or use prestained standards to assess transfer efficiency. Alternatively, use stain-free technology to assess sample binding on the blot. See the previous section for suggestions on improving transfer-related problems
	Antigen denaturation occurred during electrophoresis or transfer	• Antibodies, especially monoclonals, may not recognize denatured antigens<br>• Electrophorese and transfer proteins under native conditions. Use a cooling coil and a refrigerated recirculating bath to transfer heat-sensitive proteins
	Epitope may be blocked by total protein stain	• Some total protein stains (such as amido black and colloidal gold) interfere with antibody recognition of the antigen. Do not use a total protein stain or use a different stain or stain-free technology
	The primary or secondary antibody was inactive or nonsaturating	• Store the reagents at recommended conditions. Avoid repeated freeze-thaw cycles, bacterial contamination, and heat inactivation<br>• Detergents may affect the binding of some antibodies. Eliminate them from the assay, except for the wash after blocking<br>• If the antibody titer is too low, optimize the concentration using a dot-blot experiment<br>• Increase the antibody incubation times
	The enzyme conjugate was inactive or nonsaturating	• Test the reagent for activity*<br>• Store the reagents at recommended conditions. Avoid repeated freeze-thaw cycles, bacterial contamination, and heat inactivation<br>• Sodium azide is a potent inhibitor of horseradish peroxidase. Use a different biocide such as gentamicin sulfate<br>• Undistilled water may cause inactivation of the enzyme. Use only distilled, deionized water<br>• If the conjugate concentration is too low, optimize using a dot-blot experiment
	The color development reagent was inactive	• Test the reagent for activity* and remake if necessary
Regions of poor or uneven signal during detection	The membrane was allowed to dry during handling	• High intensity or rapid transfer methods generate heat. Ensure that warm membranes are not allowed to dry after transfer

*** Tests for Monitoring Reagent Activity**

1. Test the activity of the color development solution. Combine 1.0 ml of the color development solution with 10 µl of full-strength secondary antibody conjugate. The color reaction should occur immediately. If color fails to develop within a few minutes, the color development solution is inactive. Prepare a fresh working solution and repeat the color development assay.
2. Test the activity of the conjugate solution. Combine 1.0 ml of the color development solution tested above and 1.0 ml of the 1:3,000 dilution conjugate solution. A light-blue tinge should develop within 15 min. If color fails to develop within 25 min, the conjugate solution is suspect. Repeat the procedure with a freshly prepared dilution of conjugate.
3. Test the activity of the first antibody solution. Use an ELISA, RID, Ouchterlony immunodiffusion, or precipitation test to determine reactivity of the antibody with the antigen. If possible, repeat the assay procedure with a more concentrated primary antibody solution.

Multiscreen Apparatus

Problem	Cause	Solution
Leakage or cross-well contamination	The instrument was assembled incorrectly	• Tighten the screws using a diagonal crossing pattern to ensure uniform pressure on the membrane surface. Do not overtighten because this will cause the channels to cut into the membrane
	The sample template has warped and can no longer provide a proper seal. (Heating the apparatus to >50°C will warp the acrylic plates.)	• Replace the sample template
Incomplete or uneven filtration	Bubbles trapped within the channels	• Tilt the instrument backward during sample application to help bubbles rise to the top<br>• Use slow and careful delivery of reagent to prevent trapping bubbles inside the channels
Halos around the wells	The membrane was not rehydrated after assembly	• Rehydrate the membrane prior to loading samples. Apply vacuum only until solutions are removed from the sample wells, then disconnect the vacuum
	Too much protein was loaded, overloading the capacity of the membrane	• Determine optimal loading conditions by performing serial dilutions of samples
	The blocking step was too short	• Make sure blocking step is as long as the longest incubation period

Total Protein Detection

Problem	Cause	Solution
Colloidal gold total protein stain — high background	The blocking step was insufficient or was omitted	• Block with 0.3% Tween 20 in TBS using 3 washes of 20 min each
	Contamination occurred during electrophoresis or transfer	• Discard and remake the gel and transfer solutions<br>• Replace or thoroughly clean contaminated fiber pads if a tank blotter was used
	Excessive amounts of protein were loaded on the gel or too much SDS was used in the transfer buffer. Proteins can pass through the membrane without binding and recirculate through a tank blotting system	• Reduce the amount of protein on the gel or SDS in the transfer buffer<br>• Add a second sheet of membrane to bind excess protein
	The colloidal gold stain solution was contaminated	• Use a separate, clean plastic container to store previously used reagent in the refrigerator<br>• Discard any reagent that has a viscous sediment at the bottom of the bottle<br>• If the solution is no longer dark burgundy but light blue, discard it. The stain is contaminated with buffer salts, which react with the gold solution, causing nonspecific precipitation of the reagent onto the membrane
	The development step was too long	• Overnight development may slightly increase sensitivity but may also increase background. Reduce development step to 1–2 hr
Colloidal gold total protein stain — low sensitivity	The incubation time was insufficient	• Increase the incubation time for detection of low-level signals. Overnight incubation is possible, although background staining can increase
	Transfer was incomplete	• See "Poor electrophoretic transfer" on page 64
	The stain was exhausted, as evidenced by the loss of the dark burgundy color and longer staining times	• Discard the reagent
	Buffer salt contamination has occurred; the solution is light blue instead of dark burgundy	• Discard the reagent
Anionic dyes — high background	Destaining was insufficient	Increase the number and duration of washes with the destaining solution
	The dye solution was too concentrated	Prepare new solution

Problem	Cause	Solution
Anionic dyes — low sensitivity	Anionic dye stains do not detect protein bands below ~100 ng	• Use a more sensitive stain such as colloidal gold stain or a fluorescent stain<br>• Increase the sample load
Fluorescent blot stains — low sensitivity	Proteins with low hydrophobicity	• Only highly hydrophobic proteins will retain enough SYPRO stain to be visible on a membrane.SDS is stripped off proteins during transfer, resulting in very little retention of the SYPRO stain on most proteins
	Incorrect excitation and emission settings were used	• Refer to the product literature for correct excitation wavelengths and emission filters

TABLE OF CONTENTS

Appendix A

TABLE OF CONTENTS

Protein Standards for Blotting

Protein standards are mixtures of well-characterized or recombinant proteins that are loaded alongside protein samples in a gel. Properties and applications of Bio-Rad's blotting standards are summarized in Table A.1 and Figure A.1.

Protein standards:

- Provide a reference for determining the molecular weight of proteins identified by antibody or ligand probes
- Are useful for monitoring electrophoresis and transfer efficiency
- Serve as controls to ensure proper location of transferred bands in repetitive screening experiments

Protein standards are also available either prestained or unstained:

- Unstained protein standards offer the most accurate size determinations

Table A.1. Protein standards selection guide.

Protein Standard	Type	Features	Molecular Weight Range (on Tris-HCl or TGX™ gels)	Molecular Weight Determination	Monitoring Electrophoresis	Monitoring Transfer Efficiency	Chemiluminescence Detection	Singleplex Fluorescence Detection	Multiplex Fluorescence Detection
Precision Plus Protein™ unstained standards	Unstained recombinant	Integrated *Strep*-tag for chemiluminescence visualization	10–250 kD	•			•		
Precision Plus Protein™ WesternC™ standards	Prestained recombinant	Prestained multicolored fluorescent bands with integrated *Strep*-tag for chemiluminescence visualization	10–250 kD		•	•	•	•	•
Precision Plus Protein Dual Color standards	Prestained recombinant	Prestained multicolored fluorescent bands, 2-color band pattern	10–250 kD		•	•		•	•
Precision Plus Protein Dual Xtra standards	Prestained recombinant	Prestained multicolored fluorescent bands, 2-color band pattern, extended MW range	2–250 kD		•	•		•	•
Precision Plus Protein™ Kaleidoscope™ standards	Prestained recombinant	Prestained multicolored fluorescent bands, 5-color band pattern	10–250 kD		•	•		•	•
Precision Plus Protein All Blue standards	Prestained recombinant	Prestained fluorescent bands	10–250 kD		•	•			
Prestained SDS-PAGE standards (natural)	Prestained natural	Prestained fluorescent bands	Broad: 7.2–208 kD<br>High: 47–205 kD<br>Low: 19–107 kD					•	

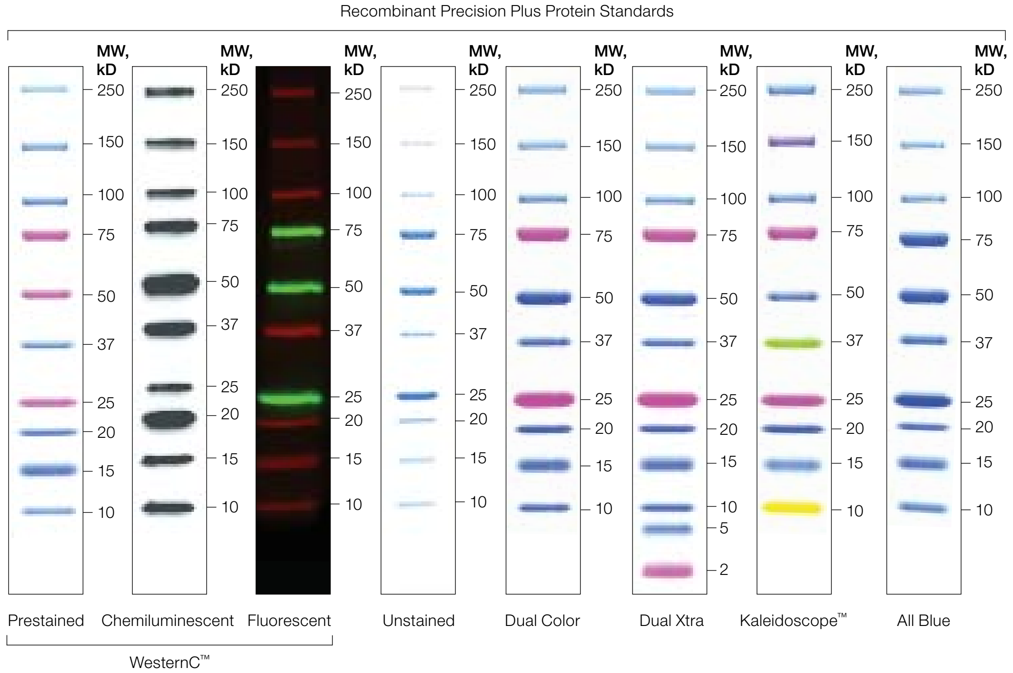

Fig. A.1. Bio-Rad's protein standards for western blotting applications.

- Prestained standards allow easy and direct visualization of the separation during electrophoresis and of their subsequent transfer to membranes

Protein standards are available as recombinant proteins:

- Recombinant standards are engineered with attributes such as evenly spaced molecular weights or affinity tags for easy detection; Bio-Rad's recombinant standards are available as the Precision Plus Protein™ standards family.

Unstained Standards for Protein Blotting

Unstained standards contain only purified proteins, so they do not exhibit the variability in molecular weight sometimes observed with prestained standards. Therefore, unstained standards or standards with affinity tags for blot detection deliver high molecular weight accuracy across a linear fit to a standard migration curve (r^2 >0.99) and are recommended for the most accurate molecular weight determinations for gels or blots. Figure A.1 summarizes molecular weights of Bio-Rad's unstained standards. These standards also image well after activation on stain-free gels.

Precision Plus Protein Unstained Standards

Precision Plus Protein unstained standards provide a recombinant ten-band, broad range molecular weight ladder (10–250 kD). These standards contain an affinity *Strep*-tag peptide that displays an intrinsic binding affinity towards StrepTactin, a genetically modified form of streptavidin. It is the high-affinity binding of the *Strep*-tag sequence to StrepTactin that allows convenient and simultaneous detection of both proteins and standards on western blots (Figure A.2) using either colorimetric or chemiluminescence methods.

TABLE OF CONTENTS

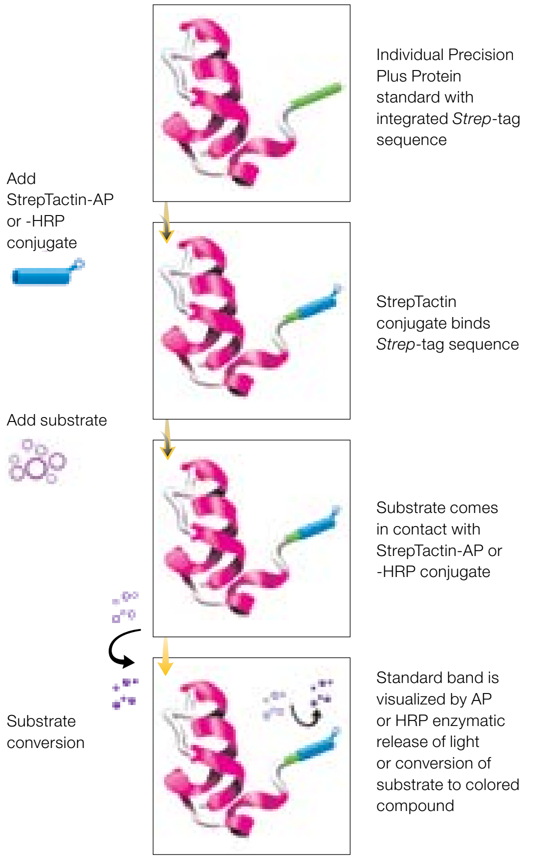

Fig. A.2. Overview of the StrepTactin detection system.

Prestained Standards for Western Blotting

The ability to visualize prestained standards during electrophoresis makes them ideal for monitoring protein separation during PAGE. The ease in transferring to the blot also make them popular for monitoring transfer efficiency and the general location of antigens in repetitive screening assays (Tsang et al. 1984). This, combined with recent improvements made in their design and manufacture, has made prestained standards an excellent choice for estimations of molecular weights on western blots.

Precision Plus Protein™ Prestained Standards

Precision Plus Protein prestained standards are a blend of ten recombinant proteins and provide a ten-band, broad range molecular weight ladder (10–250 kD) with single (all blue), dual (dual color), or multicolored (Kaleidoscope™) protein bands (Figure A.1); Precision Plus Protein Dual Xtra protein standards provide an extended molecular weight range of 2–250 kD (12 bands). The colors allow easy band referencing and blot orientation.

Because the proteins in the Precision Plus Protein standards are recombinant and the staining technology is optimized, molecular weights do not vary from lot to lot. Dye labeling can be controlled more effectively, delivering homogeneous staining and tight, sharp bands. All Precision Plus Protein prestained standards deliver the most linear fit to a standard migration curve (r^2 >0.99) available for prestained standards (Figure A.3). As a result these standards may be used for highly accurate estimation of molecular weight across a broad size range.

Kaleidoscope Standards

Kaleidoscope prestained standards contain individually colored proteins that allow instant band recognition on western blots or gels. The proteins are labeled with fluorescent dyes and so can be used in fluorescence detection applications.

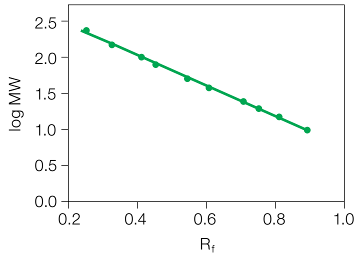

Fig. A.3. Exceptional linearity of Precision Plus Protein™ standards. The standard curve was generated by plotting the log molecular weight (MW) vs. the relative migration distance (R_f) of each protein standard band through an SDS-PAGE gel. Precision Plus Protein™ Kaleidoscope™ standards showed r^2 = 0.996, demonstrating a very linear standard curve.

Precision Plus Protein™ WesternC™ Standards

Precision Plus Protein WesternC standards were designed for western blotting applications. Like the rest of the Precision Plus Protein family of standards, the WesternC standards contain ten bands of 10–250 kD (Figure A.1). Unique to WesternC standards is the combination of both unstained and prestained bands that migrate in identical fashion. Having both unstained and prestained bands enables:

- Monitoring of the progression of gel electrophoresis
- Monitoring transfer efficiency
- Molecular weight determination (after blot development)

These standards have a *Strep*-tag affinity peptide to enable chemiluminescence detection when probed with StrepTactin-HRP conjugates (Figure A.2), so the protein standard appears directly on a film or CCD image. In addition, the prestained bands have fluorescence properties and so can be used in fluorescence detection applications.

Links

Precision Plus Protein Unstained Standards

Precision Plus Protein Prestained Standards

Precision Plus Protein WesternC Standards

Glossary

**4-Chloro-1-naphthol (4CN)**	Color development reagent used with horseradish peroxidase (HRP), which produces an insoluble purple reaction product at the site of an enzyme-antibody complex
**5-Bromo-4-chloro-indolyl phosphate (BCIP)**	Color development reagent used with alkaline phosphatase (AP), which in the presence of NBT produces an insoluble purple reaction product at the site of the enzyme-antibody complex
**Alkaline phosphatase (AP)**	Enzyme used as a detection reagent, usually conjugated to a secondary antibody probe
**Amido black 10B**	Anionic dye used in the total protein detection of blots
**Amplified AP kit**	Highly sensitive detection kit that utilizes a streptavidin-biotin system
**Anionic dye**	Negatively charged compound used as a stain; used in blotting to stain proteins immobilized on nitrocellulose or PVDF membranes
**Antibody**	Immunoglobulin (IgG); protein produced in response to an antigen that specifically binds the portion of the antigen that initiated its production
**Antigen**	Molecule that specifically binds with an antibody
**Assay**	Analysis of the quantity or characteristics of a substance
**Avidin**	Glycoprotein found in egg white that binds biotin with high specificity
**Background**	Nonspecific signal or noise that can interfere with the interpretation of valid signals
**Biotin**	Small molecule that binds specifically to avidin or streptavidin
**Bjerrum Schafer-Nielsen buffer**	Commonly used transfer buffer (48 mM Tris, 39 mM glycine, 20% methanol, pH 9.2)
**Blocking reagent**	Protein used to saturate unoccupied binding sites on a blot to prevent nonspecific binding of antibody or protein probes to the membrane
**Blot**	Immobilization of proteins or other molecules onto a membrane; or, the membrane that has the molecules adsorbed onto its surface
**BLOTTO**	Formulation of nonfat milk used to block nonspecific binding of proteins to membranes
**Chemiluminescence**	Emission of light due to a chemical reaction; used in the specific detection of blotted molecules
**Colloidal gold**	Stabilized solution of gold particles; used as a blot detection reagent when conjugated to antibodies or ligands. It produces a rose-red color on the membrane at the site of deposition
**Color development reagent**	Enzyme substrate used in blotting to visualize the location of an enzyme-antibody complex
**Colorimetric detection**	Detection of molecules of interest by formation of a colored product
**Conjugate**	Enzyme-antibody compound used in blotting
**Coomassie Blue**	Anionic dye used in the total protein staining of gels and blots
**Diaminobenzidine (DAB)**	Color development reagent used with HRP and other peroxidases that produces an insoluble brown reaction product at the site of the peroxidase-antibody complex
**Dot blot**	Direct application of proteins in free solution to a membrane
**Dunn buffer**	Commonly used transfer buffer (10 mM $NaHCO_3$, 3 mM Na_2CO_3, 20% methanol, pH 9.9)
**Electrophoretic blotting**	Use of the driving force of an electric field to move proteins from gels to membranes
**Enzyme conjugate**	Enzyme covalently attached to another protein; in blotting, usually an antibody
**Foam pad**	Pad used in blotter cassettes that helps hold the gel and membrane sandwich in place
**Filter paper**	Cotton fiber paper used in blotting applications and gel drying
**Gelatin**	Protein commonly used as a blocking reagent in western blotting procedures
**High-intensity transfer**	High-power blotting option. These transfers speed up the blotting process but produce heat and may cause proteins to migrate through the membrane
**Horseradish peroxidase (HRP)**	Enzyme used in the specific detection of molecules on blots, usually conjugated to a secondary antibody probe
**Immunoassay**	Test for a substance by its reactivity with an antibody
**Immunoblotting**	Blot detection by antibody binding
**Immunodetection**	Detection of a molecule by its binding to an antibody
**Immunoglobulin (IgG)**	Antibody; protein produced in response to an antigen that specifically binds the portion of the antigen that initiated its production
**Ligand**	Molecule that binds another in a complex
**Membrane**	Immobilizing support medium used in blotting, generally in the form of a sheet that has high affinity for biological molecules; for example, nitrocellulose or PVDF
**Membrane/filter paper sandwiches**	Blotting membrane and filter paper precut for a specific gel size
**Microfiltration blotting**	Use of a microfiltration device, such as the Bio-Dot® apparatus, to immobilize protein in free solution onto a membrane
**Multiplexing**	Blotting technique that allows identification of two or more bands on a membrane without having to strip and reprobe
**Multiscreen apparatus**	Instrument that allows the screening of two blots with up to 40 different antibody samples
**Native PAGE**	Version of PAGE that retains native protein configuration, performed in absence of SDS and other denaturing agents
**NHS-biotin**	N-hydroxysuccinimide-biotin, a reagent that biotinylates proteins
**Nitroblue tetrazolium (NBT)**	Color development reagent used with AP, which with BCIP produces an insoluble purple reaction product at the site of the AP-antibody complex
**Nitrocellulose**	General-purpose blotting membrane
**Nonenzymatic probe**	Molecule used in blot detection that does not involve an enzyme-catalyzed reaction; for example, a radioactive, chemiluminescent, or colloidal gold-labeled molecule

TABLE OF CONTENTS

**Nonfat dry milk**	Material used in solution as a blocking reagent for western blots
**Nonspecific binding**	Interaction between bound proteins and probes that is not a result of a specific reaction; results in spurious signals on the membrane
**PAGE**	Polyacrylamide gel electrophoresis, a common method of separating proteins
**Phycobiliprotein**	Protein from the light-harvesting complex of some algae. The fluorescent properties of these proteins make ideal fluorescence detection agents for blotting, when coupled to immunoglobulin
**Polyvinylidene difluoride (PVDF)**	Membrane used in protein blotting that has high chemical resistance, tensile strength, binding, and retentive capacity, making it ideal for use in protein sequencing
**Power supply**	Instrument that provides the electric power to drive electrophoresis and electrophoretic blotting experiments
**Primary antibody**	Antibody that binds a molecule of interest
**Prestained standards**	Mixture of molecular weight marker proteins that have covalently attached dye molecules, which render the bands visible during electrophoresis and transfer; used to assess the transfer efficiency of proteins onto a membrane
**Probe**	A molecule used to specifically identify another molecule
**Protein A**	Protein derived from *Staphylococcus aureus* that binds a wide range of immunoglobulins from various species
**Protein G**	Protein derived from *Streptococcus* that binds a wide range of immunoglobulins from various species and has a wider range of binding capabilities than protein A
**Rapid semi-dry blotting**	Semi-dry blotting technique that uses increased current density to transfer biomolecules more efficiently than other techniques
**SDS-PAGE**	Separation of molecules by molecular weight in a polyacrylamide gel matrix in the presence of a denaturing detergent, sodium dodecyl sulfate (SDS)
**Secondary antibody**	Antibody that binds a primary antibody; used to facilitate detection
**Semi-dry blotting**	Use of a semi-dry blotting apparatus, which consists of two horizontally oriented plate electrodes. The gel and membrane sandwich is positioned between the electrodes with buffer-soaked filter paper on either side of the sandwich which serve as buffer reservoirs
**Signal-to-noise ratio**	Relative difference in detection level between the specific and background signals
**Stain-free technology**	Protein detection technology involving UV-induced haloalkane modification of protein tryptophan residues. Continued exposure to UV light causes fluorescence of the modified proteins, which are then detected by a CCD imager. Sensitivity of this technique is generally equal to or better than Coomassie staining
**StrepTactin**	Genetically engineered form of streptavidin, used with the Precision Plus Protein™ Unstained standards for detection
***Strep*-tag sequence**	Amino acid sequence that can be used to tag a protein, enabling its detection by StrepTactin binding; this sequence is present in Precision Plus Protein Unstained and WesternC™ standards
**Streptavidin**	Protein that binds biotin with high affinity; generally regarded as superior to avidin because it is not glycosylated
**Substrate**	Substance that is reacted upon by an enzyme; for example, a color development reagent
**Super cooling coil**	Optional accessory of the Trans-Blot® cell that can be attached to a refrigerated water recirculator to cool the buffer during high-intensity transfers
**Supported nitrocellulose**	High tensile–strength blotting membrane; nitrocellulose that has been cast on an inert high-strength support
**Tank blotting**	Use of a tank blotting apparatus, which consists of a tank of buffer with vertically oriented platinum wire or plate electrodes; the gel and membrane are held in place between the electrodes by a porous cassette
**Total protein stain**	Reagent that binds nonspecifically to proteins; used to detect the entire protein pattern on a blot or gel
**Towbin buffer**	Common protein blotting transfer buffer (25 mM Tris, pH 8.5, 192 mM glycine, 20% methanol)
**Transfer**	Immobilization of proteins or other molecules onto a membrane by electrophoretic or passive means
**Tween 20**	Nonionic detergent; used in blot detection procedures as a blocking reagent or added to wash buffers to minimize nonspecific binding and background
**Western blotting**	Immobilization of proteins onto a membrane and subsequent detection by protein-specific binding and detection reagents

TABLE OF CONTENTS

References and Related Reading

References

Akerstrom B et al. (1985). Protein G: a powerful tool for binding and detection of monoclonal and polyclonal antibodies. J Immunol 135, 2589–2592.

Bayer EA and Wilchek M (1980). The use of the avidin-biotin complex as a tool in molecular biology. Methods Biochem Anal 26, 1–45.

Beisiegel U (1986). Protein blotting. Electrophoresis 7, 1–18.

Bers G and Garfin D (1985). Protein and nucleic acid blotting and immunobiochemical detection. Biotechniques 3, 276–288.

Bjerrum OJ and Schafer-Nielsen C (1986). Buffer systems and transfer parameters for semidry electroblotting with a horizontal apparatus. In Electrophoresis '86: Proceedings of the Fifth Meeting of the International Electrophoresis Society, M.J. Dunn, ed. (Weinheim, Germany: Wiley-VCH Verlag GmbH), 315–327.

Blake MS et al. (1984). A rapid, sensitive method for detection of alkaline phosphatase-conjugated anti-antibody on Western blots. Anal Biochem 136, 175–179.

Boyle MDP and Reis KJ (1987). Bacterial Fc receptors. Biotechnology 5, 697–703.

Burnette WN (1981). "Western blotting": electrophoretic transfer of proteins from sodium dodecyl sulfate-polyacrylamide gels to unmodified nitrocellulose and radiographic detection with antibody and radioiodinated protein A. Anal Biochem 112, 195–203.

Carr DW and Scott JD (1992). Blotting and band-shifting: techniques for studying protein-protein interactions. Trends Biochem Sci 17, 246–249.

Chaiet L and Wolf FJ (1964). The properties of streptavidin, a biotin-binding protein produced by *Streptomycetes*. Arch Biochem Biophys 106, 1–5.

Crisp SJ and Dunn MJ (1994). Detection of proteins on protein blots using chemiluminescent systems. Methods Mol Biol 32, 233–237.

Dunn MJ (1994). Detection of proteins on blots using the avidin-biotin system. Methods Mol Biol 32, 227–232.

Dunn MJ (1999). Detection of total proteins on western blots of 2-D polyacrylamide gels. Methods Mol Biol 112, 319–329.

Egger D and Bienz K (1994). Protein (western) blotting. Mol Biotechnol 1, 289–305.

Garfin DE and Bers G (1989). Basic aspects of protein blotting. In Protein Blotting: Methodology, Research and Diagnostic Applications, B.A. Baldo et al., eds. (Basel, Switzerland: Karger), 5–42.

Gershoni JM (1985). Protein blotting: developments and perspectives. Trends Biochem Sci 10, 103–106.

Gershoni JM (1987). Protein blotting: a tool for the analytical biochemist. In Advances in Electrophoresis, Vol 1, A. Chrambach et al., eds. (Weinheim, Germany: Wiley-VCH Verlag GmbH), 141–175.

Gershoni JM (1988). Protein blotting: a manual. Methods Biochem Anal 33, 1–58.

Gershoni JM and Palade GE (1983). Protein blotting: principles and applications. Anal Biochem 131, 1–15.

Gershoni JM et al. (1985). Protein blotting in uniform or gradient electric fields. Anal Biochem 144, 32–40.

Goding JW (1978). Use of staphylococcal protein A as an immunological reagent. J Immunol Methods 20, 241–253.

Gooderham K (1984). Transfer techniques in protein blotting. Methods Mol Biol 1, 165–178.

Guesdon J-L et al. (1979). The use of avidin-biotin interaction in immunoenzymatic techniques. J Histochem Cytochem 27, 1131–1139.

Harper DR et al. (1990). Protein blotting: ten years on. J Virol Methods 30, 25–39.

Hawkes R et al. (1982). A dot-immunobinding assay for monoclonal and other antibodies. Anal Biochem 119, 142–147.

Hsu SM et al. (1981). Use of avidin-biotin-peroxidase complex (ABC) in immunoperoxidase techniques: a comparison between ABC and unlabeled antibody (PAP) procedures. J Histochem Cytochem 29, 577–580.

Kurien BT and Scofield RH (2003). Protein blotting: a review. J Immunol Methods 274, 1–15.

Kyhse-Andersen J (1984). Electroblotting of multiple gels: a simple apparatus without buffer tank for rapid transfer of proteins from polyacrylamide to nitrocellulose. J Biochem Biophys Methods 10, 203–209.

Langone JJ (1982). Use of labeled protein A in quantitative immunochemical analysis of antigens and antibodies. J Immunol Methods 51, 3–22.

Legocki and Verma (1981). Multiple immunoreplica technique: screening for specific proteins with a series of different antibodies using one polyacrylamide gel. Anal Biochem 111, 385–392.

Moeremans M et al. (1987). The use of colloidal metal particles in protein blotting. Electrophoresis 8, 403–409.

Reinhart MP and Malamud D (1982). Protein transfer from isoelectric focusing gels: the native blot. Anal Biochem 123, 229–235.

Rohringer R and Holden DW (1985). Protein blotting: detection of proteins with colloidal gold, and of glycoproteins and lectins with biotin-conjugated and enzyme probes. Anal Biochem 144, 118–127.

Tovey ER and Baldo BA (1987). Comparison of semi-dry and conventional tank-buffer electrotransfer of proteins from polyacrylamide gels to nitrocellulose membranes. Electrophoresis 8, 384–387.

Tovey ER and Baldo BA (1987). Characterisation of allergens by protein blotting. Electrophoresis 8, 452–463.

Towbin H et al. (1979). Electrophoretic transfer of proteins from polyacrylamide gels to nitrocellulose sheets: procedure and some applications. Proc Natl Acad Sci USA 76, 4350–4354.

Tsang VC et al. (1984). Calibration of prestained protein molecular weight standards for use in the "Western" or enzyme-linked immunoelectrotransfer blot techniques. Anal Biochem 143, 304–307.

Tsang VC et al. (1985). Enzyme-linked immunoelectrotransfer blot (EITB). In Enzyme-Mediated Immunoassay. T.T. Ngo and H.M. Lenhoff, eds. (New York: Plenum Press), 389–414.

Turner BM (1983). The use of alkaline-phosphatase-conjugated second antibody for the visualization of electrophoretically separated proteins recognized by monoclonal antibodies. J Immunol Methods 63, 1–6.

Wisdom GB (1994). Protein blotting. Methods Mol Biol 32, 207–213.

Related Reading

Protein Electrophoresis: A guide to polyacrylamide gel electrophoresis (PAGE) and detection. Bio-Rad Bulletin 6040.

Tan A (1999). Increased transfer efficiency using a discontinuous buffer system with the Trans-Blot SD semi-dry electrophoretic transfer cell. Bio-Rad Bulletin 2134.

Sansan L et al. (2010). Precision Plus Protein Dual Xtra standards — new protein standards with an extended range from 2 to 250 kD. Bio-Rad Bulletin 5956.

Western Blotting Troubleshooter. Bio-Rad Bulletin 1529.

Chimento DP et al. (2009). Enhanced multiplex fluorescent western blotting. Bio-Rad Bulletin 5881.

McDonald K et al. (2005). Fluorescent nanoparticles for western blotting. Bio-Rad Bulletin 3179.

Elbaggari A et al. (2008). Evaluation of the Criterion Stain Free gel imaging system for use in western blotting applications. Bio-Rad Bulletin 5781.

Elbaggari A et al. (2008). Imaging of chemiluminescent western blots: comparison of digital imaging and X-ray film. Bio-Rad Bulletin 5809.

Taylor S et al. (2008). The dynamic range effect of protein quantitation in polyacrylamide gels and on western blots. Bio-Rad Bulletin 5792.

Increase western blot throughput with multiplex fluorescent detection. (2010). Bio-Rad Bulletin 5723.

Strep-tag technology for molecular weight (MW) determinations on blots using Precision Plus Protein™ standards. (2010). Bio-Rad Bulletin 2847.

Urban M and Woo L (2010). Molecular weight estimation and quantitation of protein samples using Precision Plus Protein WesternC standards, the Immun-Star WesternC chemiluminescent detection kit, and the ChemiDoc XRS imaging system. Bio-Rad Bulletin 5576.

Lai L et al. (2010). Molecular weight estimation using Precision Plus Protein WesternC standards on Criterion Tris-HCl and Criterion XT Bis-Tris gels. Bio-Rad Bulletin 5763.

Ordering Information

TABLE OF CONTENTS

Ordering Information

Electrophoretic Transfer Cells

Catalog #	Description
**Trans-Blot® Cells and Systems**	
1703939	Trans-Blot Cell with Plate Electrodes and Super Cooling Coil, includes 2 gel holder cassettes, buffer tank, lid with power cables, 4 foam pads, 1 pack precut blot absorbent filter paper (15 x 20 cm)
1703853	Trans-Blot Cell with Plate Electrodes, Super Cooling Coil, and PowerPac™ HC Power Supply (100–120/220–240V)
1703946	Trans-Blot Cell with Plate Electrodes, includes 2 gel holder cassettes, buffer tank, lid with power cables, 4 foam pads, 1 pack precut blot absorbent filter paper (15 x 20 cm)
1703850	Trans-Blot Cell with Plate Electrodes and PowerPac HC Power Supply (100–120/220–240V)
1703910	Trans-Blot Cell with Wire Electrodes, includes 2 gel holder cassettes, buffer tank, lid with power cables, 4 foam pads, 1 pack precut blot absorbent filter paper (15 x 20 cm)
1703825	Trans-Blot Cell with Wire Electrodes and PowerPac HC Power Supply (100–120/220–240V)
**Trans-Blot Cell Accessories**	
1703914	Foam Pads, 15.5 x 20.5 cm, 6 pads
1703956	Thick Blot Paper, 15 x 20 cm, for Trans-Blot cassette, 25 sheets
1703960	Extra Thick Blot Paper, 15 x 20 cm, 30 sheets
1703943	Trans-Blot Platinum Anode Plate Electrode
1703944	Trans-Blot Stainless-Steel Cathode Plate Electrode
1703945	Trans-Blot Plate Electrode Pair, platinum anode and stainless-steel cathode
1703920	Trans-Blot Standard Wire Electrode Card, cathode
1703921	Trans-Blot Standard Wire Electrode Card, anode
1703912	Super Cooling Coil, 1 required for all high-intensity transfers
1703913	Gel Holder Cassette, includes 2 fiber pads
1703922	Trans-Blot Cell Buffer Tank
1703923	Trans-Blot Cell Lid with Power Cables
**Trans-Blot Plus Cell and Systems**	
1703990	Trans-Blot Plus Cell with Plate Electrodes and Super Cooling Coil, includes 3 gel holder cassettes, buffer tank, lid with power cables, 6 foam pads, 1 pack blot absorbent filter paper (26.5 x 28 cm, 30 sheets), roller, stirbar
1703991	Trans-Blot Plus Cell with Plate Electrodes, Super Cooling Coil, and PowerPac HC Power Supply (100–120/220–240V)
1703992	Trans-Blot Plus Cell with Plate Electrodes, Super Cooling Coil, and PowerPac Universal Power Supply (100–120/220–240V)
**Trans-Blot Plus Cell Accessories**	
1703994	Trans-Blot Plus Gel/Cassette Assembly Tray
1703995	Foam Pads, 27 x 28.5 cm, 2 pads
1703997	Stirbar
1703998	Trans-Blot Plus Roller, 6 in. wide
1704990	Trans-Blot Plus Super Cooling Coil
1704991	Trans-Blot Plus Platinum Anode Plate Electrode
1704992	Trans-Blot Plus Stainless-Steel Cathode Plate Electrode
**Mini Trans-Blot® Cell and Systems**	
1703930	Mini Trans-Blot Electrophoretic Transfer Cell, includes 2 gel holder cassettes, 4 foam pads, modular electrode assembly, blue cooling unit, lower buffer tank, lid with cables
1703935	Mini Trans-Blot Module, without lower buffer tank and lid
1703989	Mini Trans-Blot Cell and PowerPac Basic Power Supply (100–120/220–240V)
1703836	Mini Trans-Blot Cell and PowerPac HC Power Supply (100–120/220–240V)
1658029	Mini-PROTEAN® Tetra Cell and Mini Trans-Blot Module, includes 10-well, 1.0 mm, 4-gel system and blotting module without lower buffer tank and lid; gel casting accessories
1658033	Mini-PROTEAN Tetra Cell, Mini Trans-Blot Module, and PowerPac Basic Power Supply
1658034	Mini-PROTEAN Tetra Cell for Mini Precast Gels, Mini Trans-Blot Module, and PowerPac Basic Power Supply
1658036	Mini-PROTEAN Tetra Cell for Mini Precast Gels, Mini Trans-Blot Module, and PowerPac HC Power Supply
1658035	Mini-PROTEAN Tetra Cell, Mini Trans-Blot Module, and PowerPac HC Power Supply
**Mini Trans-Blot Cell Accessories**	
1703931	Mini Gel Holder Cassette
1703932	Thick Blot Paper, 7.5 x 10 cm, for Mini Trans-Blot cassette, 50 sheets
1703933	Foam Pads, 8 x 11 cm, 4 pads
1703812	Mini Trans-Blot Central Core
1703919	Blue Cooling Unit, for Mini-PROTEAN Tetra tanks
1703934	Bio-Ice™ Cooling Unit, for Mini-PROTEAN 3 tanks
**Criterion™ Blotters and Systems**	
1704070	Criterion Blotter with Plate Electrodes, includes buffer tank assembled with plate electrodes, lid with cables, 2 gel holder cassettes, 4 foam pads, 1 pack precut blot absorbent filter paper, gel/blot assembly tray, roller, sealed ice block
1704071	Criterion Blotter with Wire Electrodes, includes buffer tank assembled with wire electrodes, lid with cables, 2 gel holder cassettes, 4 foam pads, 1 pack precut blot absorbent filter paper, gel/blot assembly tray, roller, sealed ice block
1656024	Criterion Cell/Plate Blotter System, includes Criterion cell and Criterion blotter with plate electrodes
1656025	Criterion Cell/Wire Blotter System, includes Criterion cell and Criterion blotter with wire electrodes
1703872	Criterion Blotter with Plate Electrodes and PowerPac HC Power Supply
1703874	Criterion Blotter with Wire Electrodes and PowerPac HC Power Supply
**Criterion Blotter Accessories**	
1704076	Optional Criterion Blotter Cooling Coil
1704080	Criterion Blotter Gel Holder Cassette
1704081	Criterion Blotter Platinum Anode Plate Electrode
1704082	Criterion Blotter Stainless-Steel Cathode Plate Electrode
1704083	Criterion Blotter Wire Electrode Card, anode
1704084	Criterion Blotter Wire Electrode Card, cathode
1704085	Thick Blot Paper, 9.5 x 15.2 cm, 50 sheets
1704086	Criterion Blotter Foam Pad, 9.5 x 15.2 cm, 4 pads
1704087	Sealed Ice Block, 2 blocks
1704089	Criterion Gel/Blot Assembly Tray
1651279	Roller
1704077	Criterion Blotter Buffer Tank
1704079	Criterion Blotter Lid with Cables
**Trans-Blot SD Semi-Dry Cell and Systems**	
1703940	Trans-Blot SD Semi-Dry Electrophoretic Transfer Cell, includes Trans-Blot SD transfer cell, Trans-Blot SD agarose gel support frame, extra thick blot paper
1703848	Trans-Blot SD Cell and PowerPac HC Power Supply
1703849	Trans-Blot SD Cell and PowerPac Universal Power Supply
**Trans-Blot SD Cell Accessories**	
1703947	Cathode Plate, stainless-steel upper electrode
1703942	Anode Plate, platinum-coated lower electrode
1703966	Extra Thick Blot Paper, for Mini-PROTEAN 3 or Ready Gel® precast gels, 7 x 8.4 cm, 60 sheets
1703967	Extra Thick Blot Paper, for Criterion gels, 8 x 13.5 cm, 60 sheets
1703968	Extra Thick Blot Paper, for PROTEAN® II xi gels, 14 x 16 cm, 30 sheets
1703969	Extra Thick Blot Paper, for PROTEAN II XL gels, 19 x 18.5 cm, 30 sheets
**Trans-Blot® Turbo™ Blotting System**	
17001917	Trans-Blot Turbo Starter System, Mini PVDF, includes, imaging system, Precision Plus Protein™ WesternC™ standards, Precision Protein™ streptactin-HRP conjugate, and Trans-Blot Turbo mini PVDF transfer pack
17001919	Trans-Blot Turbo Starter System, Midi PVDF, includes, imaging system, Precision Plus Protein™ WesternC™ standards, Precision Protein™ streptactin-HRP conjugate, and Trans-Blot Turbo midi PVDF transfer pack
17001918	Trans-Blot Turbo Starter System, Mini Nitrocellulose, includes, imaging system, Precision Plus Protein™ WesternC™ standards, Precision Protein streptactin-HRP conjugate, and Trans-Blot Turbo mini nitrocellulose transfer pack
17001915	Trans-Blot Turbo Starter System, Midi Nitrocellulose includes, imaging system, Precision Plus Protein™ WesternC™ standards, Precision Protein™ streptactin-HRP conjugate, and Trans-Blot Turbo midi nitrocellulose transfer pack
**Trans-Blot Turbo Accessories**	
1704156	Trans-Blot Turbo Mini PVDF Transfer Packs, pkg of 10, 7 x 8.5 cm, precut blotting transfer pack, includes filter paper, buffer, PVDF membrane
1704157	Trans-Blot Turbo Midi PVDF Transfer Packs, pkg of 10, 8.5 x 13.5 cm, precut blotting transfer pack, includes filter paper, buffer, PVDF membrane
1704158	Trans-Blot Turbo Mini Nitrocellulose Transfer Packs, pkg of 10, 7 x 8.5 cm, precut blotting transfer pack, includes filter paper, buffer, nitrocellulose membrane
1704159	Trans-Blot Turbo Midi Nitrocellulose Transfer Packs, pkg of 10, 8.5 x 13.5 cm, precut blotting transfer pack, includes filter paper, buffer, nitrocellulose membrane
1704152	Trans-Blot Turbo Base
1704151	Trans-Blot Turbo Cassette

Microfiltration Apparatus

Catalog #	Description
**Bio-Dot® Apparatus and Systems**	
1703938	Bio-Dot Microfiltration System, includes Bio-Dot apparatus and Bio-Dot SF module templates, vacuum manifold base, gasket support plates, gasket
1706545	Bio-Dot Apparatus, includes Bio-Dot sample template, vacuum manifold base, gasket support plate, gasket
1706547	Bio-Dot Module, without vacuum manifold base; for conversion of Bio-Dot SF to Bio-Dot apparatus
1706542	Bio-Dot SF Apparatus, includes Bio-Dot SF sample template, vacuum manifold base, gasket support plate, gasket, filter paper
1706543	Bio-Dot SF Module, without vacuum manifold base, for conversion of Bio-Dot to Bio-Dot SF apparatus
**Bio-Dot System Accessories**	
1706546	Bio-Dot Gaskets, 3 gaskets
1706544	Bio-Dot SF Gaskets, 2 gaskets
1620161	Bio-Dot/Bio-Dot SF Filter Paper, 11.3 x 7.7 cm, 60 sheets

Power Supplies

Catalog #	Description
1645050	PowerPac Basic Power Supply (100–120/220–240V)
1645052	PowerPac HC Power Supply (100–120/220–240V)
1645070	PowerPac Universal Power Supply

Membranes

Catalog #	Description
**Nitrocellulose Membrane (0.45 µm)**	
1620115	Nitrocellulose Membrane, 0.45 µm, 30 cm x 3.5 m, 1 roll
1620251	Nitrocellulose Membranes, 0.45 µm, 26.5 x 28 cm, 10 sheets
1620113	Nitrocellulose Membranes, 0.45 µm, 20 x 20 cm, 5 sheets
1620116	Nitrocellulose Membranes, 0.45 µm, 15 x 15 cm, 10 sheets
1620114	Nitrocellulose Membranes, 0.45 µm, 15 x 9.2 cm, 10 sheets
1620148	Nitrocellulose Membranes, 0.45 µm, 11.5 x 16 cm, 10 sheets
1620117	Nitrocellulose Membranes, 0.45 µm, 9 x 12 cm, 10 sheets
1620167	Nitrocellulose Membranes, 0.45 µm, 8.5 x 13.5 cm, 10 sheets
1620145	Nitrocellulose Membranes, 0.45 µm, 7 x 8.4 cm, 10 sheets
1620234	Nitrocellulose/Filter Paper Sandwiches, 0.45 µm, 8.5 x 13.5 cm, 20 pack
1620235	Nitrocellulose/Filter Paper Sandwiches, 0.45 µm, 8.5 x 13.5 cm, 50 pack
1620214	Nitrocellulose/Filter Paper Sandwiches, 0.45 µm, 7 x 8.5 cm, 20 pack
1620215	Nitrocellulose/Filter Paper Sandwiches, 0.45 µm, 7 x 8.5 cm, 50 pack
**Nitrocellulose Membrane (0.2 µm)**	
1620112	Nitrocellulose Membrane, 0.2 µm, 30 cm x 3.5 m, 1 roll
1620252	Nitrocellulose Membranes, 0.2 µm, 26.5 x 28 cm, 10 sheets

1620150	Nitrocellulose Membranes, 0.2 µm, 20 x 20 cm, 5 sheets
1620147	Nitrocellulose Membranes, 0.2 µm, 13.5 x 16.5 cm, 10 sheets
1620168	Nitrocellulose Membranes, 0.2 µm, 8.5 x 13.5 cm, 10 sheets
1620146	Nitrocellulose Membranes, 0.2 µm, 7 x 8.4 cm, 10 sheets
1620232	Nitrocellulose/Filter Paper Sandwiches, 0.2 µm, 8.5 x 13.5 cm, 20 pack
1620233	Nitrocellulose/Filter Paper Sandwiches, 0.2 µm, 8.5 x 13.5 cm, 50 pack
1620212	Nitrocellulose/Filter Paper Sandwiches, 0.2 µm, 7 x 8.5 cm, 20 pack
1620213	Nitrocellulose/Filter Paper Sandwiches, 0.2 µm, 7 x 8.5 cm, 50 pack

Supported Nitrocellulose Membrane (0.45 µm)

1620094	Supported Nitrocellulose Membrane, 0.45 µm, 30 cm x 3 m, 1 roll
1620070	Supported Nitrocellulose Membranes, 0.45 µm, 8.5 x 13.5 cm, 10 sheets
1620090	Supported Nitrocellulose Membranes, 0.45 µm, 7 x 8.4 cm, 10 sheets

Supported Nitrocellulose Membrane (0.2 µm)

1620097	Supported Nitrocellulose Membrane, 0.2 µm, 30 cm x 3 m, 1 roll
1620071	Supported Nitrocellulose Membranes, 0.2 µm, 8.5 x 13.5 cm, 10 sheets
1620095	Supported Nitrocellulose Membranes, 0.2 µm, 7 x 8.4 cm, 10 sheets

Immun-Blot® LF PVDF Membrane

1620264	Immun-Blot LF PVDF Membrane, pkg of 1 roll, 0.2 µm, 26.5 cm x 3.75 m
1620260	Immun-Blot LF PVDF/Filter Paper, pkg of 10, 7 x 8.5 cm
1620261	Immun-Blot LF PVDF/Filter Paper, pkg of 20, 7 x 8.5 cm
1620262	Immun-Blot LF PVDF/Filter Paper, pkg of 10 8.5 x 13.5 cm
1620263	Immun-Blot LF PVDF/Filter Paper, pkg of 20, 8.5 x 13.5 cm

Immun-Blot PVDF Membrane

1620177	Immun-Blot PVDF Membrane, 26 cm x 3.3 m, 1 roll
1620255	Immun-Blot PVDF Membrane, 25 x 28 cm, 10 sheets
1620176	Immun-Blot PVDF Membranes, 20 x 20 cm, 10 sheets
1620175	Immun-Blot PVDF Membranes, 10 x 15 cm, 10 sheets
1620174	Immun-Blot PVDF Membranes, 7 x 8.4 cm, 10 sheets
1620238	Immun-Blot PVDF/Filter Paper Sandwiches, 8.5 x 13.5 cm, 20 pack
1620239	Immun-Blot PVDF/Filter Paper Sandwiches, 8.5 x 13.5 cm, 50 pack
1620218	Immun-Blot PVDF/Filter Paper Sandwiches, 7 x 8.5 cm, 20 pack
1620219	Immun-Blot PVDF/Filter Paper Sandwiches, 7 x 8.5 cm, 50 pack

Sequi-Blot™ PVDF Membrane

1620184	Sequi-Blot PVDF Membrane, 26 cm x 3.3 m, 1 roll
1620182	Sequi-Blot PVDF Membranes, 20 x 20 cm, 10 sheets
1620181	Sequi-Blot PVDF Membranes, 15 x 15 cm, 10 sheets
1620180	Sequi-Blot PVDF Membranes, 10 x 15 cm, 10 sheets
1620186	Sequi-Blot PVDF Membranes, 7 x 8.4 cm, 10 sheets
1620236	Sequi-Blot PVDF/Filter Paper Sandwiches, 8.5 x 13.5 cm, 20 pack
1620237	Sequi-Blot PVDF/Filter Paper Sandwiches, 8.5 x 13.5 cm, 50 pack
1620216	Sequi-Blot PVDF/Filter Paper Sandwiches, 7 x 8.5 cm, 20 pack
1620217	Sequi-Blot PVDF/Filter Paper Sandwiches, 7 x 8.5 cm, 50 pack

Blotting Membrane/Filter Paper Sandwiches
Mini-PROTEAN Membrane/Filter Paper Sandwiches

1620212	0.2 µm Nitrocellulose/Filter Paper Sandwiches, 7 x 8.5 cm, 20 pack
1620213	0.2 µm Nitrocellulose/Filter Paper Sandwiches, 7 x 8.5 cm, 50 pack
1620214	0.45 µm Nitrocellulose/Filter Paper Sandwiches, 7 x 8.5 cm, 20 pack
1620215	0.45 µm Nitrocellulose/Filter Paper Sandwiches, 7 x 8.5 cm, 50 pack
1620260	Immun-Blot LF PVDF/Filter Paper, 7 x 8.5 cm, 10 pack
1620261	Immun-Blot LF PVDF/Filter Paper, 7 x 8.5 cm, 20 pack
1620218	Immun-Blot PVDF/Filter Paper Sandwiches, 7 x 8.5 cm, 20 pack
1620219	Immun-Blot PVDF/Filter Paper Sandwiches, 7 x 8.5 cm, 50 pack
1620216	Sequi-Blot PVDF/Filter Paper Sandwiches, 7 x 8.5 cm, 20 pack
1620217	Sequi-Blot PVDF/Filter Paper Sandwiches, 7 x 8.5 cm, 50 pack

Criterion Membrane/Filter Paper Sandwiches

1620232	0.2 µm Nitrocellulose/Filter Paper Sandwiches, 8.5 x 13.5 cm, 20 pack
1620233	0.2 µm Nitrocellulose/Filter Paper Sandwiches, 8.5 x 13.5 cm, 50 pack
1620234	0.45 µm Nitrocellulose/Filter Paper Sandwiches, 8.5 x 13.5 cm, 20 pack
1620235	0.45 µm Nitrocellulose/Filter Paper Sandwiches, 8.5 x 13.5 cm, 50 pack
1620262	Immun-Blot LF PVDF/Filter Paper, 8.5 x 13.5 cm, 10 pack
1620263	Immun-Blot LF PVDF/Filter Paper, 8.5 x 13.5 cm, 20 pack
1620238	Immun-Blot PVDF/Filter Paper Sandwiches, 8.5 x 13.5 cm, 20 pack
1620239	Immun-Blot PVDF/Filter Paper Sandwiches, 8.5 x 13.5 cm, 50 pack
1620236	Sequi-Blot PVDF/Filter Paper Sandwiches, 8.5 x 13.5 cm, 20 pack
1620237	Sequi-Blot PVDF/Filter Paper Sandwiches, 8.5 x 13.5 cm, 50 pack

Filter Paper
Blot Absorbent Filter Paper (Extra Thick)

1703965	Extra Thick Blot Paper, 7.5 x 10 cm, for Mini-PROTEAN gels, 60 sheets
1703966	Extra Thick Blot Paper, 7 x 8.4 cm, for Mini-PROTEAN gels, 60 sheets
1703967	Extra Thick Blot Paper, 8 x 13.5 cm, for Criterion precast gels, 60 sheets
1703968	Extra Thick Blot Paper, 14 x 16 cm, for PROTEAN II xi gels, 30 sheets
1703969	Extra Thick Blot Paper, 19 x 18.5 cm, for PROTEAN II XL gels, 30 sheets
1703958	Extra Thick Blot Paper, 10 x 15 cm, 30 sheets
1703959	Extra Thick Blot Paper, 15 x 15 cm, 30 sheets
1703960	Extra Thick Blot Paper, 15 x 20 cm, 30 sheets

Blot Absorbent Filter Paper (Thick)

1703932	Thick Blot Paper, 7.5 x 10 cm, for Mini Trans-Blot cassette, 50 sheets
1704085	Thick Blot Paper, 9.5 x 15.2 cm, for Criterion blotter, 50 sheets
1703955	Thick Blot Paper, 14 x 16 cm, for PROTEAN II xi gels, 25 sheets
1703956	Thick Blot Paper, 15 x 20 cm, for Trans-Blot cassette, 25 sheets
1650921	Thick Blot Paper, 18 x 34 cm, for Model 224, 443, and 543 slab gel dryers, 25 sheets
1620161	Bio-Dot/Bio-Dot SF Filter Paper, 7.7 x 11.3 cm, 60 sheets
1650962	Filter Paper Backing, 35 x 45 cm, for stained gels, 25 sheets

Blot Absorbent Filter Paper (Thin)

1620118	Thin Blot Paper, 33 cm x 3 m, 1 roll

Buffer Reagents
Electrophoresis Buffer Reagents

1610610	Dithiothreitol (DTT), 1 g
1610611	Dithiothreitol (DTT), 5 g
1610729	EDTA, 500 g
1706537	Gelatin, EIA grade, 200 g
1610717	Glycine, 250 g
1610718	Glycine, 1 kg
1610724	Glycine, 2 kg
1632109	Iodoacetamide, 30 g
1610710	2-Mercaptoethanol, 25 ml
1632101	Tributylphosphine (TBP), 200 mM, 0.6 ml
1610713	Tricine, 500 g
1610716	Tris, 500 g
1610719	Tris, 1 kg
1610730	Urea, 250 g
1610731	Urea, 1 kg

Premixed Buffers
Electrophoresis Buffers

1610732	10x Tris/Glycine/SDS, 1 L
1610772	10x Tris/Glycine/SDS, 5 L cube
1610734	10x Tris/Glycine, 1 L
1610771	10x Tris/Glycine, 5 L cube

Blot Transfer and Processing Buffers

1610734	10x Tris/Glycine, 1 L
1610771	10x Tris/Glycine, 5 L cube
1610778	10x Tris/CAPS, 1 L
1610774	20x SSC, 1 L
1610775	20x SSC, 5 L cube
1610780	10x Phosphate Buffered Saline, 1 L
1706435	10x Tris Buffered Saline, 1 L

Detergents/Blocking Reagents

1706537	Gelatin, EIA grade, 200 g
1706404	Blotting-Grade Blocker, nonfat dry milk, 300 g
1706531	Tween 20, EIA grade, 100 ml
1610781	10% (w/v) Tween 20, for easy pipetting, 1 L
1610418	SDS Solution, 20% (w/v), 1 L
1610783	1x Phosphate Buffered Saline With 1% Casein, 1 L
1610782	1x Tris Buffered Saline With 1% Casein, 1 L

Blotting Standards

1610376	Precision Plus Protein™ WesternC™ Standards, 250 µl, 50 applications
1610385	Precision Plus Protein WesternC Pack, includes 50 applications of WesternC standards and 50 applications of StrepTactin-HRP
1610363	Precision Plus Protein Unstained Standards, 1 ml, 100 applications
1610374	Precision Plus Protein Dual Color Standards, 500 µl
1610394	Precision Plus Protein Dual Color Standards Value Pack, 2.5 ml, 250 applications
1610377	Precision Plus Protein Dual Xtra Standards, 500 µl, 50 applications
1610375	Precision Plus Protein™ Kaleidoscope™ Standards, 500 µl
1610395	Precision Plus Protein Kaleidoscope Standards Value Pack, 2.5 ml, 250 applications
1610373	Precision Plus Protein All Blue Standards, 500 µl
1610393	Precision Plus Protein All Blue Standards Value Pack, 2.5 ml, 250 applications

Accessory Reagents

1706528	Avidin-HRP, 2 ml
1706533	Avidin-AP, 1 ml

StrepTactin Conjugates

1610380	Precision Protein™ StrepTactin-HRP Conjugate, 0.3 ml, 150 applications
1610382	Precision Protein StrepTactin-AP Conjugate, 0.3 ml, 150 applications

Detection Reagents
Total Protein Stains

1703127	SYPRO Ruby Protein Blot Stain, 200 ml
1610400	Coomassie Brilliant Blue R-250, 10 g
1706527	Colloidal Gold Total Protein Stain, 500 ml

Colorimetric Immun-Blot AP Assay Kits, with BCIP/NBT

1706460	Goat Anti-Rabbit IgG (H + L)-AP Assay Kit
1706461	Goat Anti-Mouse IgG (H + L)-AP Assay Kit
1706462	Goat Anti-Human IgG (H + L)-AP Assay Kit

Immun-Blot AP kits include 0.5 ml blotting-grade conjugate, blotting-grade TBS, Tween 20, gelatin, and BCIP and NBT susbstrate solution.

Colorimetric Immun-Blot HRP Assay Kits, with 4CN

1706463	Goat Anti-Rabbit IgG (H + L)-HRP Assay Kit
1706464	Goat Anti-Mouse IgG (H + L)-HRP Assay Kit
1706465	Goat Anti-Human IgG (H + L)-HRP Assay Kit
1708235	Opti-4CN™ Substrate Kit
1708237	Opti-4CN Goat Anti-Mouse Detection Kit
1708238	Amplified Opti-4CN Substrate Kit
1708240	Amplified Opti-4CN Goat Anti-Mouse Detection Kit
1708239	Amplified Opti-4CN Goat Anti-Rabbit Detection Kit

Immun-Blot HRP kits include 0.5 ml blotting-grade conjugate, blotting-grade TBS, Tween 20, gelatin, and 4CN susbstrate solution.

Blotting-Grade Conjugates, AP

1706518	Goat Anti-Rabbit IgG-AP, 1 ml
1706520	Goat Anti-Mouse IgG-AP, 1 ml
1706521	Goat Anti-Human IgG-AP, 1 ml

Blotting-Grade Conjugates, HRP

1706515	Goat Anti-Rabbit IgG (H + L)-HRP, 2 ml
1706516	Goat Anti-Mouse IgG (H + L)-HRP, 2 ml
1721050	Goat Anti-Human IgG (H + L)-HRP, 2 ml
1706522	Protein A-HRP, 1 ml
1706425	Protein G-HRP, 1 ml
1706528	Avidin-HRP, 2 ml

Blotting Substrate Reagents

1706432	AP Conjugate Substrate Kit, contains premixed BCIP and NBT solutions, color development buffer; makes 1 L color development solution
1706431	HRP Conjugate Substrate Kit, contains premixed 4CN, hydrogen peroxide solutions, color development buffer; makes 1 L color development solution
1706534	HRP Color Development Reagent, 4CN, 5 g
1706535	HRP Color Development Reagent, DAB, 5 g

Immun-Star™ AP Chemiluminescence Kits

1705010	Goat Anti-Mouse-AP Detection Kit, includes substrate, enhancer, antibody
1705011	Goat Anti-Rabbit-AP Detection Kit, includes substrate, enhancer, antibody
1705012	AP Substrate Pack, includes substrate, enhancer
1705018	AP Substrate, 125 ml

Each kit contains enough reagent for 2,500 cm^2 of membrane, or approximately 50 mini blots.

Chemiluminescence Kits

1705060	Clarity™ western ECL substrate, 200 ml
1705061	Clarity western ECL substrate, 500 ml
1705062	Clarity Max™ western ECL substrate, 100 ml
1705047	Goat Anti-Mouse-HRP Conjugate, 2 ml
1705046	Goat Anti-Rabbit-HRP Conjugate, 2 ml

500 ml substrate provides enough reagents for 4,000 cm^2 of membrane; 100 ml substrate provides enough for 800 cm^2 of membrane.

Detection Accessories

Mini Incubation Trays

1703902	Mini Incubation Trays, 20 trays
1703903	Mini Incubation Trays, 100 trays

Mini-PROTEAN II Multiscreen Apparatus

1704017	Mini-PROTEAN II Multiscreen Apparatus, includes 2 sample templates, 2 gaskets, base plate
1704018	Multiscreen Gaskets, 2 gaskets

Documentation Systems

1707991	GS-900™ calibrated densitometry system
1708195	Gel Doc™ XR+ system with Image Lab™ software, PC or Mac, includes darkroom, UV transilluminator, epi-white illumination, camera, cables, Image Lab software
1708270	Gel Doc EZ system with Image Lab software, PC or Mac, includes darkroom, camera, cables, Image Lab software; samples trays (cat#1708271, 1708272, 1708273, or 1708274) are sold separately; sample trays are required to use the system
1708265	ChemiDoc™ XRS+ system with Image Lab software, PC or Mac, includes darkroom, UV transilluminator, epi-white illumination, camera, power supply, cables, Image Lab software
17001401	ChemiDoc™ imaging system, blot and gel imaging system, UV/visible light, chemiluminscence; upgradeable for fluorescence detection
17001402	ChemiDoc™ MP imaging system, blot and gel imaging system, UV/visible light imaging, chemiluminescence, 5 fluorescence channels (RGB, NIR, IR)

TABLE OF CONTENTS

Nonidet is a trademark of Shell International Petroleum Co. Qdot is a trademark of Life Technologies Corporation. SYPRO is a trademark of Invitrogen Corporation. StrepTactin and *Strep*-tag are trademarks of the Institut für Bioanalytik GmbH. Tween is a trademark of ICI Americas Inc.

Precision Plus Protein standards are sold under license from Life Technologies Corporation, Carlsbad, CA for use only by the buyer of the product. The buyer is not authorized to sell or resell this product or its components.

Strep-tag technology for western blot detection is covered by U.S. Patent Number 5,506,121 and by UK Patent Number 2,272,698.

StrepTactin is covered by German patent application P 19641876.3. Bio-Rad Laboratories, Inc. is licensed by Institut für Bioanalytik GmbH to sell these products for research use only. Clarity Max western ECL substrate is manufactured by Cyanagen Srl and is the subject of patent application numbers US7855287, EP1950207, US9040252, AU2011202658, CA2742025, US8129136, and EP1962095, together with other equivalent granted patents and patent applications in other countries like CN102313732.

Clarity Max Western ECL Substrate is manufactured by Cyanagen Srl and is the subject of patent application numbers US7855287, EP1950207, US9040252, AU2011202658, CA2742025, US8129136, and EP1962095, together with other equivalent granted patents and patent applications in other countries like CN102313732.

Bio-Rad Laboratories, Inc.

Life Science Group

Web site *bio-rad.com* ***USA*** *1 800 424 6723* ***Australia*** *61 2 9914 2800* ***Austria*** *43 1 877 89 01 177* ***Belgium*** *32 (0)3 710 53 00* ***Brazil*** *55 11 3065 7550* ***Canada*** *1 905 364 3435* ***China*** *86 21 6169 8500* ***Czech Republic*** *420 241 430 532* ***Denmark*** *45 44 52 10 00* ***Finland*** *358 09 804 22 00* ***France*** *33 01 47 95 69 65* ***Germany*** *49 89 31 884 0* ***Hong Kong*** *852 2789 3300* ***Hungary*** *36 1 459 6100* ***India*** *91 124 4029300* ***Israel*** *972 03 963 6050* ***Italy*** *39 02 216091* ***Japan*** *81 3 6361 7000* ***Korea*** *82 2 3473 4460* ***Mexico*** *52 555 488 7670* ***The Netherlands*** *31 (0)318 540 666* ***New Zealand*** *64 9 415 2280* ***Norway*** *47 23 38 41 30* ***Poland*** *48 22 331 99 99* ***Portugal*** *351 21 472 7700* ***Russia*** *7 495 721 14 04* ***Singapore*** *65 6415 3188* ***South Africa*** *27 (0) 861 246 723* ***Spain*** *34 91 590 5200* ***Sweden*** *46 08 555 12700* ***Switzerland*** *41 026 674 55 05* ***Taiwan*** *886 2 2578 7189* ***Thailand*** *66 662 651 8311* ***United Arab Emirates*** *971 4 8187300* ***United Kingdom*** *44 020 8328 2000*

Bulletin 2895 Ver C US/EG *16-1041 1216 Sig 1215*
