# Supplementary material for: Combination decoction of Astragalus mongholicus and Salvia miltiorrhiza mitigates pressure-overload cardiac dysfunction by inhibiting multiple ferroptosis pathways
Source: Front Pharmacol. 2024 Dec 16;15:1447546. doi: 10.3389/fphar.2024.1447546 (PMC11683366; doi:10.3389/fphar.2024.1447546)
Supplement: Supplementary file 4 [file DataSheet1.ZIP › C0042 Enhanced Cell Counting Kit-8.pdf]

碧云天 Beyotime
碧云天网站 微信公众号
碧云天生物技术/Beyotime Biotechnology
订货热线: 400-1683301或800-8283301
订货e-mail: order@beyotime.com
技术咨询: info@beyotime.com
网址: http://www.beyotime.com

# Enhanced Cell Counting Kit-8 (增强型CCK-8试剂盒)

| 产品编号 | 产品名称 | 包装 |
|---|---|---|
| C0042 | Enhanced Cell Counting Kit-8 (增强型CCK-8试剂盒) | 500次 |

## 产品简介：

- Enhanced Cell Counting Kit-8，简称增强型CCK-8试剂盒或增强型CCK8试剂盒，是一种基于WST-8而广泛应用于细胞增殖和细胞毒性的快速、高灵敏度检测的试剂盒，比普通的CCK-8试剂盒具有更好的检测灵敏度和更宽的线性范围。
- WST-8是一种类似于MTT的化合物，在电子耦合试剂存在的情况下，可以被线粒体内的一些脱氢酶还原生成橙黄色的formazan (参考图1)。细胞增殖越多越快，则颜色越深；细胞毒性越大，则颜色越浅。对于同样的细胞，颜色的深浅和细胞数目呈线性关系。

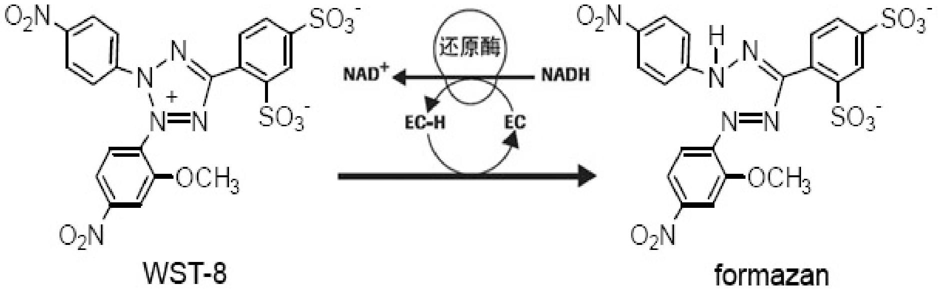


图1. WST-8检测原理图 (EC=electron coupling reagent，即电子耦合试剂)

- WST-8是MTT的一种升级替代产品，和MTT或其它MTT类似产品如XTT、MTS等相比有明显的优点。首先，MTT被线粒体内的一些脱氢酶还原生成的formazan不是水溶性的，需要有特定的溶解液来溶解；而WST-8和XTT、MTS产生的formazan都是水溶性的，可以省去后续的溶解步骤。其次，WST-8产生的formazan比XTT和MTS产生的formazan更易溶解。再次，WST-8比XTT和MTS更加稳定，使实验结果更加稳定。另外，WST-8和MTT、XTT等相比线性范围更宽，灵敏度更高。
- WST-8和WST-1相比，检测灵敏度更高，更易溶解，并且更加稳定。
- 本试剂盒可以用于细胞因子等诱导的细胞增殖检测，也可以用于抗癌药物等对细胞有毒试剂诱导的细胞毒性检测，或一些药物诱导的细胞生长抑制检测。
- 本试剂盒检测非常便捷。试剂盒仅一管已经配制好的含有WST-8的增强型CCK-8溶液，无须再进行任何配制等操作。无须使用同位素，所有的检测步骤仅在同一块96孔板内完成。不必洗涤细胞，不必收集细胞，也不必采用额外的步骤去溶解formazan。可以用于大批量样品的检测。
- 酚红和血清对本试剂盒的测定无明显影响。
- WST-8对细胞无明显毒性。加入增强型CCK-8溶液显色后，可以在不同时间反复用酶标仪读板，使检测时间更加灵活，便于找到最佳测定时间。
- 碧云天各种细胞增殖和细胞毒性检测试剂盒的比较和选择，请参考http://www.beyotime.com/support/cell-proliferation.htm。
- 本试剂盒可以测定500个样品。

## 包装清单：

| 产品编号 | 产品名称 | 包装 |
|---|---|---|
| C0042 | 增强型CCK-8溶液 | 5ml |
| — | 说明书 | 1份 |

## 保存条件：

4ºC避光保存一年有效，-20ºC避光保存两年有效。

## 注意事项：

- 由于使用96孔板进行检测，如果细胞培养时间较长，一定要注意蒸发问题。一方面，由于96孔板周围一圈最容易蒸发，可以采取弃用周围一圈的办法，改加相同量的PBS、水或培养液；另一方面，可以把96孔板置于靠近培养箱内水源的地方，以缓解蒸发。
- 本试剂盒的检测依赖于脱氢酶催化的反应，所以还原剂(例如一些抗氧化剂)会干扰检测，如果待检测体系中存在较多的还原剂，需设法去除。

- 用酶标仪检测前需确保每个孔内没有气泡，否则会干扰测定。
- 本产品仅限于专业人员的科学研究用，不得用于临床诊断或治疗，不得用于食品或药品，不得存放于普通住宅内。
- 为了您的安全和健康，请穿实验服并戴一次性手套操作。

## 使用说明：

1. 通常细胞增殖实验每孔加入100微升2000个细胞，细胞毒性实验每孔加入100微升5000个细胞(具体每孔所用的细胞的数目，需根据细胞的大小、细胞增殖速度的快慢等因素决定)。按照实验需要，进行培养并给予0-10微升特定的药物刺激。
2. 每孔加入10微升增强型CCK-8溶液。如果起始的培养体积为200微升，则需加入20微升增强型CCK-8溶液，其它情况以此类推。可以用加了相应量细胞培养液和增强型CCK-8溶液但没有加入细胞的孔作为空白对照。如果担心所使用的药物会干扰检测，需设置加了相应量细胞培养液、药物和增强型CCK-8溶液但没有加入细胞的孔作为空白对照。
3. 在细胞培养箱内继续孵育0.5-4小时，对于大多数情况孵育1小时就可以了。时间的长短根据细胞的类型和细胞的密度等实验情况而定，初次实验时可以在0.5、1、2和4小时后分别用酶标仪检测，然后选取吸光度范围比较适宜的一个时间点用于后续实验。
4. 在450nm测定吸光度。如无450nm滤光片，可以使用420-480nm的滤光片。可以使用大于600nm的波长，例如650nm，作为参考波长进行双波长测定。
5. 将不同数量的HeLa细胞按照每孔100微升培养液接种到96孔板中，培养至细胞贴壁充分后，每孔加入10微升的增强型CCK-8溶液孵育2小时后测定A450的检测效果图参考图2。检测效果仅供参考，实测数据会因检测仪器等的不同而存在差异。

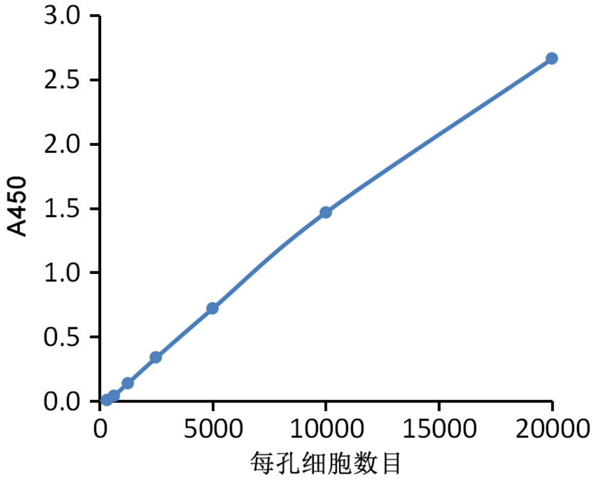


图2. 增强型CCK-8试剂盒测定不同数量HeLa细胞的检测效果图。实测数据会因检测仪器等的不同而存在差异，图中数据仅供参考。

## 常见问题：

**1. 增强型CCK-8溶液对细胞的毒性大小如何？**

增强型CCK-8溶液对细胞的毒性非常低，和普通的CCK-8溶液相当或者毒性更低，通常观察不到细胞毒性。细胞在CCK-8法检测后仍然可以正常生长，并可以用于其它的细胞实验。但为了避免增强型CCK-8溶液可能带来的对于后续检测的影响，除非该细胞极难获得，否则不推荐把增强型CCK-8溶液孵育过的细胞用于其它实验。

**2. 如果吸光度值太低，可以采取什么办法？**

a. 适当增加细胞数量。
b. 延长加入增强型CCK-8溶液后的孵育时间。

## 相关产品：

| 产品编号 | 产品名称 | 包装 |
|---|---|---|
| C0009 | MTT细胞增殖及细胞毒性检测试剂盒 | 500次 |
| C0035 | WST-1细胞增殖及细胞毒性检测试剂盒 | 100次 |
| C0036 | WST-1细胞增殖及细胞毒性检测试剂盒 | 500次 |
| C0037 | Cell Counting Kit-8 (CCK-8试剂盒) | 100次 |
| C0038 | Cell Counting Kit-8 (CCK-8试剂盒) | 500次 |
| C0039 | Cell Counting Kit-8 (CCK-8试剂盒) | 2500次 |
| C0040 | Cell Counting Kit-8 (CCK-8试剂盒) | 10000次 |
| C0041 | Enhanced Cell Counting Kit-8 (增强型CCK-8试剂盒) | 100次 |
| C0042 | Enhanced Cell Counting Kit-8 (增强型CCK-8试剂盒) | 500次 |
| C0043 | Enhanced Cell Counting Kit-8 (增强型CCK-8试剂盒) | 2500次 |
| C0046 | Enhanced Cell Counting Kit-8 (增强型CCK-8试剂盒) | 10000次 |

## 使用本产品的文献：


1. Pourjavadi A, Tehrani ZM, Moghanaki AA.Folate-Conjugated pH-Responsive Nanocarrier Designed for Active Tumor Targeting and Controlled Release of Gemcitabine.PHARM RES-DORDR . 2016 Feb;33(2):417-32
2. Lei Li, Beibei Lu, Qikui Fan, Lulu Wei, Jianning Wu,Jun Hou, Xuhong Guo and Zhiyong Liu.Synthesis and pH-responsive self-assembly behavior of a fluorescent amphiphilic triblock copolymer mPEG-b-PCL-b-PDMAEMA-g-PC for the controlled intracellular delivery of doxorubicin .RSC Adv .2016 Mar;32:27102-27112
3. Chen K, Wang JL, Huang SY, Yang WB, Zhu WN, Zhu XQ.Immune responses and protection after DNA vaccination against Toxoplasma gondii calcium-dependent protein kinase 2 (TgCDPK2).Parasite . 2017;24:41
4. Jia X, Xu Y, Wu W, Fan Y, Wang G, Zhang T, Su W.Aroclor1254 disrupts the blood-testis barrier by promoting endocytosis and degradation of junction proteins via p38 MAPK pathway.Cell Death Dis . 2017 May 25;8(5):e2823
5. Xiaoyu Jia,Ying Xu,Weixing Wu,Yunxia Fan,Guoli Wang,Tianbiao Zhang,Wenhui SuAroclor1254 disrupts the blood-testis barrier by promoting endocytosis and degradation of junction proteins via p38 MAPK pathway.Cell Death Dis. 2017 May 25;8(5):e2823.;doi: 10.1038/cddis.2017.224
6. Dang L, Teng M, Li HZ, Ma SM, Lu QX, Hao HF, Zhao D, Zhou EM, Zhang GP, Luo J..Marek's disease virus type 1 encoded analog of miR-155 promotes proliferation of chickenembryo fibroblast and DF-1 cells by targeting hnRNPAB.Vet Microbiol . 2017 Aug;207:210-218
7. Li H, Al-Japairai K, Tao Y, Xiang Z.RPN2 promotes colorectal cancer cell proliferation through modulating the glycosylation status of EGFR.ONCOTARGET . 2017 Aug 7;8(42):72633-72651
8. Zhu WN, Wang JL, Chen K, Yue DM, Zhang XX, Huang SY, Zhu XQ.Evaluation of protective immunity induced by DNA vaccination with genes encoding Toxoplasmagondii GRA17 and GRA23 against acute toxoplasmosis in mice.Exp Parasitol . 2017 Aug;179:20-27
9. Wang XY, Yang H, Wang MG, Yang DB, Wang ZY, Wang L.Trehalose protects against cadmium-induced cytotoxicity in primary rat proximal tubular cellsvia inhibiting apoptosis and restoring autophagic flux.Cell Death Dis . 2017 Oct 12;8(10):e3099
10. Zhao M, Wan B, Li H, He J, Chen X, Wang L, Wang Y, Xie S, Qiao S, Zhang G.Porcine 2', 5'-oligoadenylate synthetase 2 inhibits porcine reproductive and respiratorysyndrome virus replication in vitro.MICROB PATHOGENESIS . 2017 Oct;111:14-21
11. Gao Y, Li S, Wang J, Luo C, Zhao S, Zheng N.Modulation of Intestinal Epithelial Permeability in Differentiated Caco-2 Cells Exposed to Aflatoxin M1 and Ochratoxin A Individually or Collectively.TOXINS. 2017 Dec 27;10(1):13
12. Wan CX,Xu M,Huang SH,Wu QQ,Yuan Y,Deng W,Tang QZ.Baicalein protects against endothelial cell injury by inhibiting the TLR4/NF-κB signaling pathway.Mol Med Rep . 2018 Feb;17(2):3085-3091
13. Lv Y,Zhang P,Guo J,Zhu Z,Li X,Xu D,Zeng W.Melatonin protects mouse spermatogonial stem cells against hexavalent chromium-induced apoptosis and epigenetic histone modification.TOXICOL APPL PHARM . 2018 Feb 1;340:30-38
14. Shi H,Bi H,Sun X,Dong H,Jiang Y,Mu H,Liu G,Kong W,Gao R,Su J.Antitumor effects of Tubeimoside-1 in NCI-H1299 cells are mediated by microRNA-126-5p-induced inactivation of VEGF-A/VEGFR-2/ERK signaling pathway.Mol Med Rep . 2018 Mar;17(3):4327-4336
15. Xie D,Zhang H,Shang C.Long non-coding RNA CDKN2B antisense RNA 1 gene inhibits Gemcitabine sensitivity in bladder urothelial carcinoma.J Cancer . 2018 May 25;9(12):2160-2166
16. Ke S,Liu Q,Yao Y,Zhang X,Sui G.An in vitro cytotoxicities comparison of 16 priority polycyclic aromatic hydrocarbons in human pulmonary alveolar epithelial cells HPAEpiC.Toxicol Lett . 2018 Jun 15;290:10-18
17. Chen Y,Zhao K,Liu F,Li Y,Zhong Z,Hong S,Liu X,Liu L.Predicting Antitumor Effect of Deoxypodophyllotoxin in NCI-H460 Tumor-Bearing Mice on the Basis of In Vitro Pharmacodynamics and a Physiologically Based Pharmacokinetic-Pharmacodynamic Model.Drug Metab Dispos. 2018 Jun;46(6):897-907
18. Singh P,Ren X,Guo T,Wu L,Shakya S,He Y,Wang C,Maharjan A,Singh V,Zhang J.Biofunctionalization of β-cyclodextrin nanosponges using cholesterol.CARBOHYD POLYM. 2018 Jun 15;190:23-30
19. Zhang Q, Li XT, Chen Y, Chen JQ, Zhu JY, Meng Y, Wang XQ, Li Y, Geng SS, Xie CF, Wu JS, Zhong CY, Han HY..Wnt/β-catenin signaling mediates the suppressive effects of diallyl trisulfide on colorectal cancer stem cells.CANCER CHEMOTH PHARM. 2018 Jun;81(6):969-977
20. Zhang LL,Du JB,Tang CS,Jin HF,Huang YQ.Inhibitory Effects of Sulfur Dioxide on Rat Myocardial Fibroblast Proliferation and Migration.CHINESE MED J-PEKING . 2018 Jul 20;131(14):1715-1723
21. Wang Z,Dou M,Liu F,Jiang P,Ye S,Ma L,Cao H,Du X,Sun P,Su N,Lin F,Zhang R,Li C.GDF11 induces differentiation and apoptosis and inhibits migration of C17.2 neural stem cells via modulating MAPK signaling pathway.PeerJ . 2018 Sep 4;6:e5524
22. Shi H,Bi H,Sun X,Dong H,Jiang Y,Mu H,Li W,Liu G,Gao R,Su J.Tubeimoside-1 inhibits the proliferation and metastasis by promoting miR-126-5p expression in non-small cell lung cancer cells.Oncol Lett . 2018 Sep;16(3):3126-3134
23. Wang R,Ma H,Kang Y,Li C,Li H,Zhang E,Ji P,He J,Zhao M.Molecular Cloning and Identification of the 2'-5' Oligoadenylate Synthetase 2 Gene in Chinese Domestic Pigs Through Bioinformatics Analysis, and Determination of Its Antiviral Activity Against PorcineIndian J Microbiol. 2018 Sep;58(3):332-344
24. Sui M,Jiang X,Chen J,Yang H,Zhu Y.Magnesium isoglycyrrhizinate ameliorates liver fibrosis and hepatic stellate cell activation by regulating ferroptosis signaling pathway.Biomed Pharmacother . 2018 Oct;106:125-133
25. Dai C,Sun M,Wang F,Zhu J,Wei Y,Guo X,Ma S,Dong B,Wang G,Jiang F,Wang J.The Selective RNA Polymerase I Inhibitor CX-5461 Mitigates Neointimal Remodeling in a Modified Model of Rat Aortic Transplantation.Transplantation . 2018 Oct;102(10):1674-1683
26. Liu Z,Xie D,Zhang H.Long noncoding RNA neuroblastoma-associated transcript 1 gene inhibits malignant cellular phenotypes of bladder cancer through miR-21/SOCS6 axis.Cell Death Dis . 2018 Oct 11;9(10):1042
27. Liu M,Wang SM,Jiang ZX,Lauren H,Tao LM.Effects of miR-22 on viability, migration, invasion and apoptosis in retinoblastoma Y79 cells by targeting high-mobility group box 1.INT J OPHTHALMOL-CH. 2018 Oct 18;11(10):1600-1607
28. Wang J, Wang Q, Zhou F, Li J, Li Q, Zhou H, Li S, Ma S, Wen T.The antitumor effect of TAT-DCF1 peptide in glioma cells.Neuropeptides. 2018 Oct;71:21-31
29. Lin S,Liu K,Zhang Y,Jiang M,Lu R,Folts CJ,Gao X,Noble MD,Zhao T,Zhou Z,Lan X,Que J.Pharmacological targeting of p38 MAP-Kinase 6 (MAP2K6) inhibits the growth of esophageal adenocarcinoma.Cell Signal . 2018 Nov;51:222-232
30. Guan JY,Liao TT,Yu CL,Luo HY,Yang WR,Wang XZ.ERK1/2 regulates heat stress-induced lactate production via enhancing the expression of HSP70 in immature boar Sertoli cells.CELL STRESS CHAPERON. 2018 Nov;23(6):1193-1204
31. Li Q,Li QQ,Jia JN,Cao S,Wang ZB,Wang X,Luo C,Zhou HH,Liu ZQ,Mao XY.Sodium Valproate Ameliorates Neuronal Apoptosis in a Kainic Acid Model of Epilepsy via Enhancing PKC-Dependent GABAAR γ2 Serine 327 Phosphorylation.Neurochem Res . 2018 Dec;43(12):2343-2352
32. Li Y,Zhang S,Wang Y,Peng J,Fang F,Yang X.MLH1 enhances the sensitivity of human endometrial carcinoma cells to cisplatin by activating the MLH1/c-Abl apoptosis signaling pathway.BMC Cancer . 2018 Dec 29;18(1):1294
33. Sun X,Li X,Ma S,Guo Y,Li YMicroRNA-98-5p ameliorates oxygen-glucose deprivation/reoxygenation (OGD/R)-induced neuronal injury by inhibiting

Bach1 and promoting Nrf2/ARE signaling.BIOCHEM BIOPH RES CO. 2018 Dec 9;507(1-4):114-121

34. Wang Q,Shi G,Zhang Y,Lu F,Xie D,Wen C,Huang L.Deciphering the Potential Pharmaceutical Mechanism of GUI-ZHI-FU-LING-WAN on Systemic Sclerosis based on Systems Biology Approaches.SCI REP-UK. 2019 Jan 23;9(1):355
35. Wang L,Zhang Z,Li M,Wang F,Jia Y,Zhang F,Shao J,Chen A,Zheng S.P53-dependent induction of ferroptosis is required for artemether to alleviate carbon tetrachloride-induced liver fibrosis and hepatic stellate cell activation.IUBMB Life. 2019 Jan;71(1):45-56
36. Shang C,Ao CN,Cheong CC,Meng L.Long Non-coding RNA CDKN2B Antisense RNA 1 Gene Contributes to Paclitaxel Resistance in Endometrial Carcinoma.Front Oncol. 2019 Jan 29;9:27
37. Hu X,Hong Y,Shang C.Knockdown of long non-coding RNA SNHG5 inhibits malignant cellular phenotypes of glioma via Wnt/CTNNB1 signaling pathway.J Cancer. 2019 Jan 30;10(5):1333-1340
38. Yuan L,Li X,Ge L,Jia X,Lei J,Mu C,Li D.Emulsion Template Method for the Fabrication of Gelatin-Based Scaffold with a Controllable Pore Structure.ACS APPL MATER INTER. 2019 Jan 9;11(1):269-277
39. Cai Y,Wang Y,Xu H,Cao C,Zhu R,Tang X,Zhang T,Pan Y.Positive magnetic resonance angiography using ultrafine ferritin-based iron oxide nanoparticles.Nanoscale. 2019 Feb 7;11(6):2644-2654
40. Huang Y,Du KL,Guo PY,Zhao RM,Wang B,Zhao XL,Zhang CQ.IL-16 regulates macrophage polarization as a target gene of mir-145-3p.Mol Immunol. 2019 Mar;107:1-9.
41. Wang R,Kang Y,Li H,Ma H,Wang W,Cheng Y,Ji P,Zhang E,Zhao M.Molecular cloning and functional characterization of porcine 2',5'-oligoadenylate synthetase 1b and its effect on infection with porcine reproductive and respiratory syndrome virus.VET IMMUNOL IMMUNOP. 2019 Mar;209:22-30
42. Li H,Wang C,He T,Zhao T,Chen YY,Shen YL,Zhang X,Wang LL.Mitochondrial Transfer from Bone Marrow Mesenchymal Stem Cells to Motor Neurons in Spinal Cord Injury Rats via Gap Junction.Theranostics. 2019 Mar 17;9(7):2017-2035
43. Li ZX,Zhao GD,Xiong W,Linghu KG,Ma QS,Cheang WS,Yu H,Wang Y.Immunomodulatory effects of a new whole ingredients extract from Astragalus: a combined evaluation on chemistry and pharmacology.Chinese Medical Journal. 2019 Mar 27;14:12.
44. Xu Z,Huang B,Zhang Q,He X,Wei H,Zhang D.NOTCH1 regulates the proliferation and migration of bladder cancer cells by cooperating with long non-coding RNA HCG18 and microRNA-34c-5p.J Cell Biochem. 2019 Apr;120(4):6596-6604
45. Wu B,Huang L,Qiu W,Liu X,Shen Y,Lu Y,Yang Z,Li X,Cui B,Xu S,Qiao H,Qiu R,Yao L,Kan Y,Li D.Small nucleolar RNA Sf-15 regulates proliferation and apoptosis of Spodoptera frugiperda Sf9 cells.BMC Mol Biol. 2019 Apr 11;20(1):12
46. Bo Wu,Lei Huang,Wujie Qiu,Xiao Liu,Yawen Shen,Yiping Lu,Zonglin Yang,Xinmei Li,Bin Cui,Shidong Xu,Huili Qiao,Reng Qiu,Lunguang Yao,Yunchao Kan,Dandan Li.Small nucleolar RNA Sf-15 regulates proliferation and apoptosis of Spodoptera frugiperda Sf9 cells.BMC Mol Biol. 2019 Apr 11;20(1):12.;doi: 10.1186/s12867-019-0128-9
47. Fan W,Du F,Liu X.TRIM66 confers tumorigenicity of hepatocellular carcinoma cells by regulating GSK-3 β -dependent Wnt/ β -catenin signaling.Eur J Pharmacol. 2019 May 5;850:109-117
48. He C,Zhao Y,Jiang X,Liang X,Yin L,Yin Z,Geng Y,Zhong Z,Song X,Zou Y,Li L,Zhang W,Lv C.Protective effect of Ketone musk on LPS/ATP-induced pyroptosis in J774A.1 cells through suppressing NLRP3/GSDMD pathway.Int Immunopharmacol. 2019 Jun;71:328-335
49. Xiao Y,Pan J,Geng Q,Wang G.LncRNA MALAT1 increases the stemness of gastric cancer cells via enhancing SOX2 mRNA stability.FEBS Open Bio. 2019 Jul;9(7):1212-1222
50. Liu G,Wang C,Wang H,Zhu L,Zhang H,Wang Y,Pei C,Liu L.Antiviral efficiency of a coumarin derivative on spring viremia of carp virus in vivo.Virus Res. 2019 Jul 15;268:11-17
51. Xie C,Zhu J,Jiang Y,Chen J,Wang X,Geng S,Wu J,Zhong C,Li X,Meng Z.Sulforaphane Inhibits the Acquisition of Tobacco Smoke-Induced Lung Cancer Stem Cell-Like Properties via the IL-6/ΔNp63 α /Notch Axis.Theranostics. 2019 Jul 9;9(16):4827-4840
52. Zhu J,Cao D,Guo C,Liu M,Tao Y,Zhou J,Wang F,Zhao Y,Wei J,Zhang Y,Fang W,Li Y.Berberine Facilitates Angiogenesis Against Ischemic Stroke Through Modulating Microglial Polarization via AMPK Signaling.Cell Mol Neurobiol. 2019 Aug;39(6):751-768
53. Huang SY,Chen K,Wang JL,Yang B,Zhu XQ.Evaluation of protective immunity induced by recombinant calcium-dependent protein kinase 1 (TgCDPK1) protein against acute toxoplasmosis in mice.MICROB PATHOGENESIS. 2019 Aug;133:103560
54. Ji X,Tang Z,Shuai W,Zhang Z,Li J,Chen L,Cao J,Yin W.Endogenous peptide LYENRL prevents the activation of hypertrophic scar-derived fibroblasts by inhibiting the TGF- β 1/Smad pathway.Life Sci. 2019 Aug 15;231:116674
55. Qi Yao,Jun Yang,Ting Liu,Jianjiang Zhang,Yibo Zheng.Long Noncoding RNA MALAT1 Promotes the Stemness of Esophageal Squamous Cell Carcinoma by Enhancing YAP Transcriptional Activity.FEBS Open Bio. 2019 Aug;9(8):1392-1402.;doi: 10.1002/2211-5463.12676
56. He P,Yan H,Zhao J,Gou M,Li X.An evaluation of the wound healing potential of tetrahydrocurcumin-loaded MPEG-PLA nanoparticles.J Biomater Appl. 2019 Sep;34(3):315-325
57. Bai L,Gao S,Sun H,Zhao X,Yang L,Hu H,Sun J,Jiang W.Effects of Wnt10b on dermal papilla cells via the canonical Wnt/ β -catenin signalling pathway in the Angora rabbit.J Anim Physiol Anim Nutr (Berl). 2019 Sep;103(5):1602-1609.
58. Hao Y,Fan X,Shi Y,Zhang C,Sun DE,Qin K,Qin W,Zhou W,Chen X.Next-generation unnatural monosaccharides reveal that ESRRB O-GlcNAcylation regulates pluripotency of mouse embryonic stem cells.Nat Commun. 2019 Sep 6;10(1):4065
59. Yi Hao,Xinqi Fan,Yujie Shi,Che Zhang,De-En Sun,Ke Qin,Wei Qin,Wen Zhou,Xing Chen.Next-generation unnatural monosaccharides reveal that ESRRB O-GlcNAcylation regulates pluripotency of mouse embryonic stem cells.Nat Commun. 2019 Sep 6;10(1):4065.;doi: 10.1038/s41467-019-11942-y
60. Zheng F,Wang F,Xu Z.MicroRNA-98-5p prevents bone regeneration by targeting high mobility group AT-Hook 2.Exp Ther Med. 2019 Oct;18(4):2660-2666
61. Li T,Zhao P,Li Z,Wang CC,Wang YL,Gu Q.miR-200c-3p Suppresses the Proliferative, Migratory, and Invasive Capacities of Nephroblastoma Cells via Targeting FRS2.Biopreserv Biobank. 2019 Oct;17(5):444-451
62. Zhang Q,Cao WS,Wang XQ,Zhang M,Lu XM,Chen JQ,Chen Y,Ge MM,Zhong CY,Han HY.Genistein inhibits nasopharyngeal cancer stem cells through sonic hedgehog signaling.Phytother Res. 2019 Oct;33(10):2783-2791
63. Li C,Chen W,Zheng L,Zhang B,Yang X,Zhang Q,Wang N,Wang Y,Yang J,Sha J,Zhou Z,Li X,Li Y,Shen XL.Ameliorative effect of ursolic acid on ochratoxin A-induced renal cytotoxicity mediated by Lonp1/Aco2/Hsp75.Toxicon. 2019 Oct;168:141-146
64. Li Y,Qiao Z,Yu F,Hu H,Huang Y,Xiang Q,Zhang Q,Yang Y,Zhao Y.Transforming Growth Factor- β 3/Chitosan Sponge (TGF- β 3/CS) Facilitates Osteogenic Differentiation of Human Periodontal Ligament Stem Cells.Int J Mol Sci. 2019 Oct 9;20(20). pii: E4982
65. Yangfan Li,Zhifen Qiao,Fenglin Yu,Huiting Hu,Yadong Huang,Qi Xiang,Qihao Zhang,Yan Yang,Yueping Zhao.Transforming Growth Factor- β 3/Chitosan Sponge (TGF- β 3/CS) Facilitates Osteogenic Differentiation of Human Periodontal Ligament Stem Cells.Int J Mol Sci. 2019 Oct 9;20(20):4982.;doi: 10.3390/ijms20204982
66. Wang W,Wang YR,Chen J,Chen YJ,Wang ZX,Geng M,Xu DC,Wang ZY,Li JH,Xu ZD,Pan LL,Sun J.Pterostilbene Attenuates Experimental Atherosclerosis

through Restoring Catalase-Mediated Redox Balance in Vascular Smooth Muscle Cells.J AGR FOOD CHEM. 2019 Nov 20;67(46):12752-12760

67. Pei J,Xiao W,Zhu D,Ji X,Shi L,Deng X.LncRNA DSCAM-AS1 Promotes Proliferation, Migration and Invasion of Colorectal Cancer Cells via Modulating miR-144-5p/CDKL1.Life Sci. 2019 Nov 12:117050
68. Jiaping Pei,Wen Xiao,Danyan Zhu,Xiaowei Ji,Liping Shi,Xiaozhao Deng.WITHDRAWN: LncRNA DSCAM-AS1 Promotes Proliferation, Migration and Invasion of Colorectal Cancer Cells via Modulating miR-144-5p/CDKL1Life Sci. 2019 Nov 12;117050.;doi: 10.1016/j.lfs.2019.117050
69. Gong L,Chang H,Xu H.LncRNA MALAT1 knockdown alleviates oxygen-glucose deprivation and reperfusion induced cardiomyocyte apoptotic death by regulating miR-122.Exp Mol Pathol. 2019 Dec;111:104325
70. Li-Qiong Huang,Bo Zheng,Yi He.Immune Negative Regulator TIPE2 Inhibits Cervical Squamous Cancer Progression Through Erk1/2 Signaling.Open Life Sci. 2019 Dec 31;14:528-536.;doi: 10.1515/biol-2019-0059
71. Yu-Chao Zhu,Yong He,Jian-Fa Liu,Jia Chen.Adjuvantic cytokine IL-33 improves the protective immunity of cocktailed DNA vaccine of ROP5 and ROP18 against toxoplasma gondii infection in mice.Parasite. 2020;27:26.;doi: 10.1051/parasite/2020021
72. Hao Niu,Yiwei Huang,Li Yan,Li Zhang,Mengnan Zhao,Tao Lu,Xiaodong Yang,Zhengcong Chen,Cheng Zhan,Yu Shi,Qun Wang.Knockdown of SMAD3 inhibits the growth and enhances the radiosensitivity of lung adenocarcinoma via p21 in vitro and in vivo.Int J Biol Sci. 2020 Jan 30;16(6):1010-1022.;doi: 10.7150/ijbs.40173
73. Qiang Ma,Rungui Niu,Wei Huang,Liangshan Da,Yanlei Tang,Daowen Jiang,Yanfeng Xi,Congjun Zhang.Long Noncoding RNA PTPRG Antisense RNA 1 Reduces Radiosensitivity of Nonsmall Cell Lung Cancer Cells Via Regulating MiR-200c-3p/TCF4TECHNOL CANCER RES T. Jan-Dec 2020;19:1533033820942615.;doi: 10.1177/1533033820942615
74. Handong Liu,Keqi Hu.The Long Intergenic Noncoding RNA 00707 Sponges MicroRNA-613 (miR-613) to Promote Proliferation and Invasion of Gliomas.TECHNOL CANCER RES T. Jan-Dec 2020;19:1533033820962092.;doi: 10.1177/1533033820962092
75. Lei Yang,Guangping Zhou,Mingyang Li,Yan Li,Liqing Yang,Qin Fu,Ye Tian.High Glucose Downregulates Connexin 43 Expression and Its Gap Junction and Hemichannel Function in Osteocyte-like MLO-Y4 Cells Through Activation of the p38MAPK/ERK Signal Pathway.DIABET METAB SYND OB. 2020 Feb 26;13:545-557.;doi: 10.2147/DMSO.S239892
76. Li HH,Li J,Zhang XJ,Li JM,Xi C,Wang WQ,Lu YL,Xuan LJ.23,24-Dihydrocucurbitacin B promotes lipid clearance by dual transcriptional regulation of LDLR and PCSK9.Acta Pharmacol Sin. 2020 Mar;41(3):327-335
77. Ma Z,Guo J,Zhang Y,Zhang Y,Zhang M,Zong R,Chen F,Zhang J.Neuromedin B regulates steroidogenesis, cell viability and apoptosis in rabbit Leydig cells.GEN COMP ENDOCR. 2020 Mar 1;288:113371
78. Zhiyu Ma,Junpei Guo,Ying Zhang,Youwen Zhang,Miao Zhang,Rongling Zong,Fenglei Chen,Jinlong Zhang.Neuromedin B regulates steroidogenesis, cell viability and apoptosis in rabbit Leydig cells.GEN COMP ENDOCR. 2020 Mar 1;288:113371.;doi: 10.1016/j.ygcen.2019.113371
79. Tao Wang,Yuan Yang,Wei Feng,Ren Wang,Zhengxing Chen.Co-folding of hydrophobic rice proteins and shellac in hydrophilic binary microstructures for cellular uptake of apigenin.Food Chem. 2020 Mar 30;309:125695.;doi: 10.1016/j.foodchem.2019.125695
80. Hui-Hui Li,Jun Li,Xian-Jing Zhang,Jiao-Meng Li,Cong Xi,Wen-Qiong Wang,You-Li Lu,Li-Jiang Xuan.23,24-Dihydrocucurbitacin B promotes lipid clearance by dual transcriptional regulation of LDLR and PCSK9.Acta Pharmacol Sin. 2020 Mar;41(3):327-335.;doi: 10.1038/s41401-019-0274-0
81. Qian Ning,Yamei Pang,Shan Shao,Minna Luo,Lin Zhao,Tinghua Hu,Xinhan Zhao.MicroRNA-147b suppresses the proliferation and invasion of non-small-cell lung cancer cells through downregulation of Wnt/β-catenin signalling via targeting of RPS15A.CLIN EXP PHARMACOL P . 2020 Mar;47(3):449-458.;doi: 10.1111/1440-1681.13203
82. Wanjuan Li,Fei Yu,Hao Wang,Xupeng Hong,Liqun Lu.Induction of pro-viral grass carp Ctenopharyngodon idella Hsp70 instead of Hsc70 during infection of grass carp reovirus.FISH SHELLFISH IMMUN. 2020 Mar;98:1024-1029.;doi: 10.1016/j.fsi.2019.11.042
83. Chang Guo,Yu Shangguan,Meiru Zhang,Yanxin Ruan,Guoqing Xue,Jingfan Ma,Jian Yang,Longxin Qiu.Rosmarinic acid alleviates ethanol-induced lipid accumulation by repressing fatty acid biosynthesis.Food Funct. 2020 Mar 1;11(3):2094-2106.;doi: 10.1039/c9fo02357g
84. Fei Zhou,Yun Li,Yisheng Huang,Jian Wu,Qinhan Wu,Hui Zhu,Jinke Wang.Upregulation of CASP9 through NF-κB and Its Target MiR-1276 Contributed to TNFα-promoted Apoptosis of Cancer Cells Induced by Doxorubicin.Int J Mol Sci. 2020 Mar 26;21(7):2290.;doi: 10.3390/ijms21072290
85. Sun D,Luo T,Dong P,Zhang N,Chen J,Zhang S,Dong L,Janssen HLA,Zhang S.M2-polarized tumor-associated macrophages promote epithelial-mesenchymal transition via activation of the AKT3/PRAS40 signaling pathway in intrahepatic cholangiocarcinoma.J Cell Biochem. 2020 Apr;121(4):2828-2838
86. Yan Yang,Zong Xiu Yin,Zhao Yang Wang,Shu Bo Tian,Hong Chang Wang,Fang Xu Zhang,Le Ping Li,Chunning Zheng,Shuai Kong.miR-7641 depletion suppresses proliferation of gastric cancer cells by targeting ARID1A.ANTI-CANCER DRUG. 2020 Apr;31(4):368-376.;doi: 10.1097/CAD.0000000000000881
87. Qinqin Xiang,Fen Xu,Yunzhu Li,Xuanyu Liu,Qianlong Chen,Jiuzuo Huang,Nanze Yu,Ziyi Zeng,Meng Yuan,Qixu Zhang,Xiao Long,Zhou Zhou.Transcriptome analysis and functional identification of adipose-derived mesenchymal stem cells in secondary lymphedema.Gland Surg. 2020 Apr;9(2):558-574.;doi: 10.21037/gs.2020.02.09
88. Dan Wang,Bin Xue,Tymish Y Ohulchanskyy,Yubin Liu,Artem Yakovliev,Roman Ziniuk,Mengze Xu,Jun Song,Junle Qu,Zhen Yuan.Inhibiting tumor oxygen metabolism and simultaneously generating oxygen by intelligent upconversion nanotherapeutics for enhanced photodynamic therapy.Biomaterials. 2020 May 3;251:120088.;doi: 10.1016/j.biomaterials.2020.120088
89. Na Geng,Xiaozhou Wang,Xiaohui Yu,Run Wang,Yiran Zhu,Meihua Zhang,Jianzhu Liu,Yongxia Liu.Staphylococcus aureus Avoids Autophagy Clearance of Bovine Mammary Epithelial Cells by Impairing Lysosomal Function.Front Immunol. 2020 May 5;11:746.;doi: 10.3389/fimmu.2020.00746
90. Aru Ling,Lingwei Sun,Wenbo Guo,Shiyao Sun,Junhua Yang,Zhihui Zhao.Individual and combined cytotoxic effects of T-2 toxin and its four metabolites on porcine Leydig cells.Food Chem Toxicol. 2020 May;139:111277.;doi: 10.1016/j.fct.2020.111277
91. Rongwe Yangi,Huafei Huang,Qingnv Zhou.Long noncoding RNA MALAT1 sponges miR-129-5p to regulate the development of bronchopulmonary dysplasia by increasing the expression of HMGB1.J Int Med Res. 2020 May;48(5):300060520918476.;doi: 10.1177/0300060520918476
92. Songwei Huan,Tao Gui,Qiutong Xu,Songkuan Zhuang,Zhenyan Li,Yuling Shi,Jiebin Lin,Bin Gong,Guiqiang Miao,Manseng Tam,Huan-Tian Zhang,Zhengang Zha,Chunfei Wu.Combination BET Family Protein and HDAC Inhibition Synergistically Elicits Chondrosarcoma Cell Apoptosis Through RAD51-Related DNA Damage Repair.Cancer Manag Res. 2020 Jun 10;12:4429-4439.;doi: 10.2147/CMAR.S254412
93. Yin Yuan,Bo Li,Yanbin Kuang,Shuo Ni,Aoxiang Zhuge,Jing Yang,Longxian Lv,Silan Gu,Ren Yan,Yating Li,Kaicen Wang,Liya Yang,Xueling Zhu,Jingjing Wu,Xiaoyuan Bian,Lanjuan Li.The fiber metabolite butyrate reduces gp130 by targeting TRAF5 in colorectal cancer cells.Cancer Cell Int. 2020 Jun 3;20:212.;doi: 10.1186/s12935-020-01305-9
94. Mingxiang Wang,Jin Li,Shunni Dong,Xiaobo Cai,Aili Simaiti,Xin Yang,Xinqiang Zhu,Jianhong Luo,Lin-Hua Jiang,Binyang Du,Peilin Yu,Wei Yang.Silica nanoparticles induce lung inflammation in mice via ROS/PARP/TRPM2 signaling-mediated lysosome impairment and autophagy dysfunction.Part Fibre Toxicol. 2020 Jun 8;17(1):23.;doi: 10.1186/s12989-020-00353-3
95. Parbeen Singh,Li Wu,Xiaohong Ren,Wei Zhang,Yan Tang,Yongli Chen,Andrew Carrier,Xu Zhang,Jiwen Zhang.Hyaluronic-acid-based β-cyclodextrin grafted copolymers as biocompatible supramolecular hosts to enhance the water solubility of tocopherol.INT J PHARMACOL. 2020 Aug

30;586:119542.;doi: 10.1016/j.ijpharm.2020.119542

96. Jie Qu,Xiuqin Zhang,Xiying Lv.Zinc finger protein 750(ZNF750), negatively regulated by miR-17-5p, inhibits proliferation, motility and invasion of colonic cancer cells.J Gene Med. 2020 Aug;22(8):e3195.;doi: 10.1002/jgm.3195
97. Xiao-Ying Zheng,Ming-Zheng Cao,Ying Ba,Yue-Feng Li,Jun-Ling Ye.LncRNA testis-specific transcript, Y-linked 15 (TTTY15) promotes proliferation, migration and invasion of colorectal cancer cells via regulating miR-29a-3p/DVL3 axis.Cancer Biomark. 2020 Sep 22.;doi: 10.3233/CBM-201709
98. Mengze Du,Xiaodan Liu,Jiajia Xu,Shuxian Li,Shenghua Wang,Yaohong Zhu,Jiufeng Wang.Antimicrobial Effect of Zophobas morio Hemolymph against Bovine Mastitis Pathogens.Microorganisms. 2020 Sep 28;8(10):1488.;doi: 10.3390/microorganisms8101488
99. Xiao-Ping Xu,Xiang-Qun Peng,Xin-Min Yin,Yi Liu,Ze-Ya Shi.miR-34a-5p suppresses the invasion and metastasis of liver cancer by targeting the transcription factor YY1 to mediate MYCT1 upregulation.Acta Histochem. 2020 Sep;122(6):151576.;doi: 10.1016/j.acthis.2020.151576
100. Xiaoxiao Gao,Xiaolei Yao,Zhibo Wang,Xiaohe Li,Xiaodan Li,Shiyu An,Zongyou Wei,Guomin Zhang,Feng Wang.Long non-coding RNA366.2 controls endometrial epithelial cell proliferation and migration by upregulating WNT6 as a ceRNA of miR-1576 in sheep uterus.BBA-GENE REGUL MECH. 2020 Sep;1863(9):194606.;doi: 10.1016/j.bbagrm.2020.194606
101. Sunkai Ling,Yanru He,Xiaoxue Li,Mingyue Hu,Yu Ma,Yuan Li,Zipeng Lu,Shanshan Shen,Bo Kong,Xiaoping Zou,Kuirong Jiang,Peilin Huang.CircRHOT1 mediated cell proliferation, apoptosis and invasion of pancreatic cancer cells by sponging miR-125a-3p.J Cell Mol Med. 2020 Sep;24(17):9881-9889.;doi: 10.1111/jcmm.15572
102. Yuan He,Mudi Ma,Yiguang Yan,Can Chen,Hui Luo,Wei Lei.Combined pre-conditioning with salidroside and hypoxia improves proliferation, migration and stress tolerance of adipose-derived stem cells.J Cell Mol Med. 2020 Sep;24(17):9958-9971.;doi: 10.1111/jcmm.15598
103. LiPan Peng,ZeZhong Chen,GuangChuan Wang,ShuBo Tian,Shuai Kong,Tao Xu,XiaoHua An,YueZhi Chen.Long noncoding RNA LBX2-AS1-modulated miR-4766-5p regulates gastric cancer development through targeting CXCL5.Cancer Cell Int. 2020 Oct 12;20:497.;doi: 10.1186/s12935-020-01579-z
104. Conghao Zhong,Zemin Liu,Xibo Qiao,Li Kang,Yi Sun,Yunliang Jiang.Integrated Transcriptomic Analysis on Small Yellow Follicles Reveals that Sosondowah Ankyrin Repeat Domain Family Member A Inhibits Chicken Follicle Selection.ASIAN AUSTRAL J ANIM. 2020 Oct 13.;doi: 10.5713/ajas.20.0404
105. Yiru Wang,Qingyun Jia,Yifan Zhang,Jing Wei,Ping Liu.Amygdalin Attenuates Atherosclerosis and Plays an Anti-Inflammatory Role in ApoE Knock-Out Mice and Bone Marrow-Derived Macrophages.Front Pharmacol. 2020 Oct 29;11:590929.;doi: 10.3389/fphar.2020.590929
106. Man-Fei Zhao,Gong-Da Liang,Yu-Jie Zhou,Zhi-Ping Chi,He Zhuang,Song-Ling Zhu,Yao Wang,Gui-Rong Liu,Jing-Bo Zhao,Shu-Lin Liu.Novel Bacillus strains from the human gut exert anticancer effects on a broad range of malignancy types.INVEST NEW DRUG. 2020 Oct;38(5):1373-1382.;doi: 10.1007/s10637-020-00906-5
107. Pengcheng Wang,Juan Bai,Xuewei Liu,Mi Wang,Xianwei Wang,Ping Jiang.Tomatidine inhibits porcine epidemic diarrhea virus replication by targeting 3CL protease.Vet Res. 2020 Nov 11;51(1):136.;doi: 10.1186/s13567-020-00865-y
108. Yun Yu,Yalan Chen,Kexin Liu,Jinke Cheng,Jun Tu.SUMOylation enhances the activity of IDH2 under oxidative stress.BIOCHEM BIOPH RES CO. 2020 Nov 19;532(4):591-597.;doi: 10.1016/j.bbrc.2020.08.089
109. Jin Qiu,Zhiyin Zhang,Sainan Wang,Yanru Chen,Caizhi Liu,Sainan Xu,Dongmei Wang,Junlei Su,Mengshan Ni,Jian Yu,Xiangdi Cui,Lu Ma,Tianhui Hu,Yepeng Hu,Xuejiang Gu,Xinran Ma,Jiqiu Wang,Lingyan Xu.Transferrin Receptor Functionally Marks Thermogenic Adipocytes.Front Cell Dev Biol. 2020 Nov 5;8:572459.;doi: 10.3389/fcell.2020.572459
110. Xiaofen Xie,Xinying Li,Jinfeng Lei,Xi Zhao,Yongbo Lyu,Changdao Mu,Defu Li,Liming Ge,Yongbin Xu.Oxidized starch cross-linked porous collagen-based hydrogel for spontaneous agglomeration growth of adipose-derived stem cells.MAT SCI ENG C-MATER. 2020 Nov;116:111165.;doi: 10.1016/j.msec.2020.111165
111. Yiqun Ji,Jialu Yao,Yang He.Extracellular ubiquitin protects cardiomyocytes during ischemia/hypoxia by inhibiting mitochondrial apoptosis pathway through CXCR4.Biomed Pharmacother. 2020 Nov;131:110787.;doi: 10.1016/j.biopha.2020.110787
112. Anjie Min,Haofeng Xiong,Weiming Wang,Xin Hu,Can Wang,Ting Mao,Liudi Yang,Danni Huang,Kun Xia,Tong Su.CD147 promotes proliferation and migration of oral cancer cells by inhibiting junctions between E-cadherin and β-catenin.J Oral Pathol Med. 2020 Nov;49(10):1019-1029.;doi: 10.1111/jop.13088
113. Yubo Liu,Qiushi Chen,Nana Zhang,Keren Zhang,Tongyi Dou,Yu Cao,Yimin Liu,Kun Li,Xinya Hao,Xueqin Xie,Wenli Li,Yan Ren,Jianing Zhang.Proteomic profiling and genome-wide mapping of O-GlcNAc chromatin-associated proteins reveal an O-GlcNAc-regulated genotoxic stress response.Nat Commun. 2020 Nov 19;11(1):5898.;doi: 10.1038/s41467-020-19579-y
114. Shuo Wang,Wei Su,Chuanfan Zhong,Taowei Yang,Wenbin Chen,Guo Chen,Zezhen Liu,Kaihui Wu,Weibo Zhong,Bingkun Li,Xiangming Mao,Jianming Lu.An Eight-CircRNA Assessment Model for Predicting Biochemical Recurrence in Prostate Cancer.Front Cell Dev Biol. 2020 Dec 10;8:599494.;doi: 10.3389/fcell.2020.599494
115. Hao Liu,Jikun Wang,Dan Wang,Minghua Kong,Chao Ning,Xing Zhang,Jinlong Xiao,Xin Zhang,Jianfeng Liu,Xingbo Zhao.Cybrid Model Supports Mitochondrial Genetic Effect on Pig Litter Size.Front Genet. 2020 Dec 15;11:579382.;doi: 10.3389/fgene.2020.579382
116. Yu Du,Qian Wang,Na Tian,Meng Lu,Xian-Long Zhang,Sheng-Ming Dai.Knockdown of nrf2 Exacerbates TNF-α-Induced Proliferation and Invasion of Rheumatoid Arthritis Fibroblast-Like Synoviocytes through Activating JNK Pathway.J Immunol Res. 2020 Dec 22;2020:6670464.;doi: 10.1155/2020/6670464
117. Muhammad Waqqas Hasan,Muhammad Haseeb,Muhammad Ehsan,Javaid Ali Gadahi,Muhammad Ali-Ul-Husnain Naqvi,Qiang Qiang Wang,Xinchao Liu,Shakeel Ahmed Lakho,Ruofeng Yan,Lixin Xu,Xiaokai Song,Xiangrui Li.Nanoparticles (PLGA and Chitosan)-Entrapped ADP-Ribosylation Factor 1 of Haemonchus contortus Enhances the Immune Responses in ICR Mice.Vaccines (Basel). 2020 Dec 2;8(4):726.;doi: 10.3390/vaccines8040726.
118. Xianmei Liu,Xue Xia,Xifu Wang,Jing Zhou,Lanping Amy Sung,Jinhua Long,Xueyu Geng,Zhu Zeng,Weijuan Yao.Tropomodulin1 Expression Increases Upon Maturation in Dendritic Cells and Promotes Their Maturation and Immune Functions.Front Immunol. 2021 Jan 15;11:587441.;doi: 10.3389/fimmu.2020.587441
119. Xiaoqiang Du,Huan Liu,Yonghua Yue,Qingjiang Wu,Wenli Jiang,Yan Qiu,Ye Zeng.Anisodamine Hydrobromide Protects Glycocalyx and Against the Lipopolysaccharide-Induced Increases in Microvascular Endothelial Layer Permeability and Nitric Oxide Production.Cardiovasc Eng Technol. 2021 Feb;12(1):91-100.;doi: 10.1007/s13239-020-00486-8.

Version 2021.09.01
