# Supplementary material for: Combination decoction of Astragalus mongholicus and Salvia miltiorrhiza mitigates pressure-overload cardiac dysfunction by inhibiting multiple ferroptosis pathways
Source: Front Pharmacol. 2024 Dec 16;15:1447546. doi: 10.3389/fphar.2024.1447546 (PMC11683366; doi:10.3389/fphar.2024.1447546)
Supplement: Supplementary file 4 [file DataSheet1.ZIP › C0121 Crystal Violet Staining Kit.pdf]

碧云天生物技术/Beyotime Biotechnology
订货热线: 400-1683301或800-8283301
订货e-mail: order@beyotime.com
技术咨询: info@beyotime.com
网址: http://www.beyotime.com

碧云天网站 微信公众号

结晶紫染色液

产品编号	产品名称	包装
C0121-100ml	结晶紫染色液	100ml
C0121-500ml	结晶紫染色液	500ml

产品简介：

- 碧云天生产的结晶紫染色液(Crystal Violet Staining Solution)是一种组织或细胞染色时常用的可以把细胞核染成深紫色的染色液。
- 结晶紫是一种碱性染料，可以和细胞核中的DNA结合，从而产生细胞核染色。
- 一个100ml包装的本染色液至少可以染色200个样品，500ml包装的本染色液至少可以染色1000个样品。

包装清单：

产品编号	产品名称	包装
C0121-100ml	结晶紫染色液	100ml
C0121-500ml	结晶紫染色液	500ml
—	说明书	1份

保存条件：

室温避光保存，一年有效。

注意事项：

- 需自备4%多聚甲醛。如果需要脱水、透明和封片处理，还需自备二甲苯，中性树胶或其它封片剂。如果样品是石蜡切片，需自备70%和90%乙醇，无水乙醇以及二甲苯。
- 样品数量较多时，可以使用碧云天生产的染色架和染色缸，便于操作。
- 第一次使用本试剂盒时建议先取1-2个样品做预实验。
- 结晶紫对人体有害，操作时请小心，并注意有效防护以避免直接接触人体或吸入体内。
- 本产品仅限于专业人员的科学研究用，不得用于临床诊断或治疗，不得用于食品或药品，不得存放于普通住宅内。
- 为了您的安全和健康，请穿实验服并戴一次性手套操作。

使用说明：

1. 样品处理

a. 对于石蜡切片：

二甲苯中脱蜡5-10分钟。
换用新鲜的二甲苯，再脱蜡5-10分钟。
无水乙醇5分钟。
90%乙醇2分钟。
70%乙醇2分钟。
蒸馏水2分钟。

b. 对于冰冻切片：

蒸馏水2分钟。

c. 对于培养细胞：

用4%多聚甲醛固定10分钟以上。
蒸馏水洗涤2分钟。
换用新鲜的蒸馏水，再洗涤2分钟。

2. 结晶紫染色

对于上述处理好的样品：
结晶紫染色液染色10分钟(可以根据染色结果和要求调整时间)。
用蒸馏水或自来水充分洗涤后即可进行观察和拍照。

相关产品：

产品编号	产品名称	包装

C0105S	苏木素伊红(HE)染色试剂盒	>200次
C0105M	苏木素伊红(HE)染色试剂盒	>1000次
C0107-100ml	苏木素染色液	100ml
C0107-500ml	苏木素染色液	500ml
C0109	伊红染色液	100ml
C0115	甲基绿染色液	100ml
C0117	尼氏(Nissl)染色液	100ml
C0119	甲基绿-派洛宁染色液	100ml
C0121-100ml	结晶紫染色液	100ml
C0121-500ml	结晶紫染色液	500ml
C0123	中性红染色液	100ml
C0125	中性红染色液(活细胞染色用)	100ml
C0161S	盐酸乙醇慢速分化液	100ml
C0161M	盐酸乙醇慢速分化液	500ml
C0161L	盐酸乙醇慢速分化液(20X)	100ml
C0163S	盐酸乙醇快速分化液	100ml
C0163M	盐酸乙醇快速分化液	500ml
C0163L	盐酸乙醇快速分化液(20X)	100ml
C0165S	盐酸乙醇超快速分化液	100ml
C0165M	盐酸乙醇超快速分化液	500ml
C0165L	盐酸乙醇超快速分化液(20X)	100ml

使用本产品的文献：

1. Xu B, Niu X, Zhang X, Tao J, Wu D, Wang Z, Li P, Zhang W, Wu H, Feng N, Wang Z, Hua L, Wang X. miR-143 decreases prostate cancer cells proliferation and migration and enhances their sensitivityto docetaxel through suppression of KRAS. Mol Cell Biochem. 2011 Apr;350(1-2):207-13.
2. Li H, Bian C, Liao L, Li J, Zhao RC. miR-17-5p promotes human breast cancer cell migration and invasion through suppression ofHBP1. Breast Cancer Res Treat. 2011 Apr;126(3):565-75.
3. Liu HF, Zhang HJ, Hu QX, Liu XY, Wang ZQ, Fan JY, Zhan M, Chen FL.Altered polarization, morphology, and impaired innate immunity germane to resident peritoneal macrophages inmice with long-term type 2 diabetes.J BIOMED BIOTECHNOL l. 2012;2012:867023
4. Xu WS, Dang YY, Guo JJ, Wu GS, Lu JJ, Chen XP, Wang YT.Furanodiene induces endoplasmic reticulum stress and presents antiproliferative activities in lung cancer cells.EVID-BASED COMPL ALT . 2012;2012:426521
5. Wu GS, Lu JJ, Guo JJ, Li YB, Tan W, Dang YY, Zhong ZF, Xu ZT, Chen XP, Wang YT.Ganoderic acid DM, a natural triterpenoid, induces DNA damage, G1 cell cycle arrest and apoptosisin human breast cancer cells.Fitoterapia .2012 Mar;83(2):408-14
6. X Wei, T Xi, C Zhang, W Huang, MN Rahaman..Effect of Small Intestinal Submucosa on the Protection and Repair of MC3T3-E1 Cells Damaged by Dexamethasone-Induced Stress.World Congress on Medical Physics and Biomedical Engineering .2012 May;39:1957-60.
7. Xu Q, Wang L, Li H, Han Q, Li J, Qu X, Huang S, Zhao RC.Mesenchymal stem cells play a potential role in regulating the establishment and maintenance of epithelial-mesenchymal transition in MCF7 human breast cancer cells by paracrine and induced autocrine TGF-β.Int J Oncol . 2012 Sep;41(3):959-68
8. Mo WC, Zhang ZJ, Liu Y, Bartlett PF, He RQ.Magnetic shielding accelerates the proliferation of human neuroblastoma cell by promoting G1-phaseprogression.PLoS One . 2013;8(1):e54775
9. Zhang W, Tang B, Huang Q, Hua Z.Galangin inhibits tumor growth and metastasis of B16F10 melanoma.J Cell Biochem . 2013 Jan;114(1):152-61
10. Lai W, Wu J, Zou X, Xie J, Zhang L, Zhao X, Zhao M, Wang Q, Ji J.Secretome analyses of Aβ(1-42) stimulated hippocampal astrocytes reveal that CXCL10 is involved in astrocyte migration. J Proteome Res . 2013 Feb 1;12(2):832-43
11. Zhang M, Liu Y, Feng H, Bian X, Zhao W, Yang Z, Gu B, Li Z, Liu Y.CD133 affects the invasive ability of HCT116 cells by regulating TIMP-2.Am J Pathol .2013 Feb;182(2):565-76
12. Wang J, Kang WM, Yu JC, Liu YQ, Meng QB, Cao ZJ.Cadherin-17 induces tumorigenesis and lymphatic metastasis in gastric cancer through activation of NFκBsignaling pathway.Cancer Biol Ther . 2013 Mar;14(3):262-70
13. Tang W, Tang J, Qin J, Geng Q, Zhou Z, Li B, Zhang J, Chen H, Xia Y, Wang X.Involvement of down-regulated E2F3 in Hirschsprung's disease.J Pediatr Surg . 2013 Apr;48(4):813-7
14. Zhang Q, Bai X, Chen W, Ma T, Hu Q, Liang C, Xie S, Chen C, Hu L, Xu S, Liang T.Wnt/β-catenin signaling enhances hypoxia-induced epithelial-mesenchymal transition in hepatocellular carcinomavia crosstalk with hif-1α signaling.Carcinogenesis . 2013 May;34(5):962-73
15. Su N, Qiu H, Chen Y, Yang T, Yan Q, Wan X.miR-205 promotes tumor proliferation and invasion through targeting ESRRG in endometrial carcinoma.Oncol Rep . 2013 Jun;29(6):2297-302
16. Zhou DH, Wang X, Yang M, Shi X, Huang W, Feng Q.Combination of Low Concentration of (-)-Epigallocatechin Gallate (EGCG) and Curcumin Strongly Suppresses the Growth of Non-Small Cell Lung Cancer in Vitro and in Vivo through Causing Cell Cycle Arrest.Int J Mol Sci . 2013 Jun 5;14(6):12023-36
17. Liu AR, Liu L, Chen S, Yang Y, Zhao HJ, Liu L, Guo FM, Lu XM, Qiu HB.Activation of canonical wnt pathway promotes differentiation of mouse bone marrow-derived MSCs into type II alveolar epithelial cells, confers resistance to oxidative stress, and promotes their migration to injured lung tissue in vitro.J Cell Physiol . 2013 Jun;228(6):1270-83
18. Tang Q, Li G, Wei X, Zhang J, Chiu JF, Hasenmayer D, Zhang D, Zhang H.Resveratrol-induced apoptosis is enhanced by inhibition of autophagy in esophageal squamous cell carcinoma.Cancer Lett . 2013 Aug 19;336(2):325-37
19. Wu GS, Song YL, Yin ZQ, Guo JJ, Wang SP, Zhao WW, Chen XP, Zhang QW, Lu JJ, Wang YT.Ganoderiol A-enriched extract suppresses migration and adhesion of MDA-MB-231 cells by inhibiting FAK-SRC-paxillin cascade pathway.PLoS One . 2013 Oct 29;8(10):e76620
20. Kumar D, Shankar S, Srivastava RK .Rottlerin-induced autophagy leads to the apoptosis in breast cancer stem cells: molecular mechanisms.Mol Cancer . 2013 Dec 23;12(1):171
21. Ye Y, Miao S, Lu R, Xia X, Chen Y, Zhang J, Wu X, He S, Qiang F, Zhou J.Allograft inflammatory factor-1 is an independent prognostic indicator that regulates β-catenin in gastric cancer.Oncol Rep . 2014 Feb;31(2):828-34
22. Guo J, Wu G, Bao J, Hao W, Lu J, Chen X.Cucurbitacin B induced ATM-mediated DNA damage causes G2/M cell cycle arrest in a ROS-dependent manner.PLoS One . 2014 Feb 4;9(2):e88140
23. Shi Y, Chen C, Zhang X, Liu Q, Xu JL, Zhang HR, Yao XH, Jiang T, He ZC, Ren Y, Cui W, Xu C, Liu L, Cui YH, Yu SZ, Ping YF, Bian XW.Primate-specific miR-663 functions as a tumor suppressor by targeting PIK3CD and predicts the prognosis of human glioblastoma.Clin Cancer Res . 2014 Apr 1;20(7):1803-13
24. Kan J, Guo W, Huang C, Bao G, Zhu Y, Zhu YZ.S-propargyl-cysteine, a novel water-soluble modulator of endogenous hydrogen sulfide, promotes angiogenesis through activation of signal transducer and activator of transcription 3.ANTIOXID REDOX SIGN . 2014 May 20;20(15):2303-16
25. Lei H, Tang J, Li H, Zhang H, Lu C, Chen H, Li W, Xia Y, Tang W.MiR-195 affects cell migration and cell proliferation by down-regulating DIEXF in Hirschsprung's Disease.BMC Gastroenterol . 2014 Jul 9;14:123.
26. Qiu M, Liu L, Chen L, Tan G, Liang Z, Wang K, Liu J, Chen H.microRNA-183 plays as oncogenes by increasing cell proliferation, migration and invasion via targeting protein phosphatase 2A in renal cancer cells.BIOCHEM BIOPH RES CO . 2014 Sep 12;452(1):163-9
27. Zhao BJ, Liu YH.Simvastatin induces the osteogenic differentiation of human periodontal ligament stem cells.FUND CLIN PHARMACOL . 2014 Oct;28(5):583-592
28. Fei C, Zhao Y, Guo J, Gu S, Li X, Chang C.Senescence of bone marrow mesenchymal stromal cells is accompanied by activation of p53/p21 pathway in myelodysplastic syndromes.Eur J Haematol .2014 Dec;93(6):476-86
29. Gai J Q, Sheng X, Qin J M, Sun K, Zhao W, Ni L.The effect and mechanism of bufalin on regulating hepatocellular carcinoma cell invasion and metastasis via Wnt/β-catenin signaling pathway.International Journal of Oncology . 2015;48(1): 338-348
30. Ruan Z, Liu J, Kuang Y.Isolation and characterization of side population cells from the human ovarian cancer cell line SK-OV-3.Experimental and Therapeutic Medicine . 2015;10(6): 2071-8

31. Yu T, Cao R, Li S, Fu M, Ren L, Chen W, Zhu H, Zhan Q, Shi R.MiR-130b plays an oncogenic role by repressing PTEN expression in esophageal squamous cell carcinoma cells.BMC Cancer .2015 Jan 31;15:29

32. Jiang L, Wang Y, Rong Y, Xu L, Chu Y, Zhang Y, Yao Y.miR-1179 promotes cell invasion through SLIT2/ROBO1 axis in esophageal squamous cell carcinoma.INT J CLIN EXP PATHO . 2015 Jan 1;8(1):319-27

33. Shi S, Zhang M, Guo R, Miao Y, Hu J, Xi Y, Li B.In vivo Molecular Imaging and Radionuclide (131I) Therapy of Human Nasopharyngeal Carcinoma Cells Transfected with a Lentivirus Expressing Sodium iodide symporter.PLoS One . 2015 Jan 26;10(1):e0116531

34. Sharan A, Zhu H, Xie H, Li H, Tang J, Tang W, Zhang H, Xia Y.Down-regulation of miR-206 is associated with Hirschsprungdisease andsuppresses cellmigration andproliferation in cell models.SCI REP-UK . 2015 Mar 20;5:9302

35. Wang X, Jiang P, Wang P, Yang CS, Wang X, Feng Q.EGCG Enhances Cisplatin Sensitivity by Regulating Expression of the Copper and Cisplatin Influx Transporter CTR1 in Ovary Cancer.PLoS One . 2015 Apr 30;10(4):e0125402

36. Wang YF, Li T, Tang ZH, Chang LL, Zhu H, Chen XP, Wang YT, Lu JJ.Baicalein Triggers Autophagy and Inhibits the Protein Kinase B/Mammalian Target of Rapamycin Pathway inHepatocellular Carcinoma HepG2 Cells.Phytother Res . 2015 May;29(5):674-9

37. Zhu C, Zhao Y, Zhang Z, Ni Y, Li X, Yong H.MicroRNA-33a inhibits lung cancer cell proliferation and invasion by regulating the expression of β-catenin.Mol Med Rep . 2015 May;11(5):3647-51

38. Zhang X, Luo W, Zhao W, Lu J, Chen X.Isocryptotanshinone Induced Apoptosis and Activated MAPK Signaling in Human Breast Cancer MCF-7 Cells.J Breast Cancer . 2015 Jun;18(2):112-8

39. Tang W, Tang J, He J, Zhou Z, Qin Y, Qin J, Li B, Xu X, Geng Q, Jiang W, Wu W, Wang X, Xia Y.SLIT2/ROBO1 - miR - 218 - 1 - RET/PLAG1: a new disease pathway involved in Hirschsprung's disease.J Cell Mol Med . 2015 Jun;19(6):1197-207

40. Qiu M, Liang Z, Chen L, Tan G, Wang K, Liu L, Liu J, Chen H.MicroRNA-429 suppresses cell proliferation, epithelial-mesenchymal transition,and metastasis by direct targetingof BMI1 and E2F3 in renal cell carcinoma.UROL ONCOL-SEMIN ORI . 2015 Jul;33(7):332.e9-18

41. Zhu D, Xie H, Li H, Cai P, Zhu H, Xu C, Chen P, Sharan A, Xia Y, Tang W.Nidogen-1 isa common target of microRNAs MiR-192/215 in the pathogenesis of Hirschsprung's disease.J Neurochem . 2015 Jul;134(1):39-46

42. Ao N, Liu Y, Bian X, Feng H, Liu Y.Ubiquitin-specific peptidase22 inhibits colon cancer cell invasion by suppressing the signal transducer andactivator of transcription 3/matrix metalloproteinase 9 pathway.Mol Med Rep . 2015 Aug;12(2):2107-13

43. Xie H, Zhu D, Xu C, Zhu H, Chen P, Li H, Liu X, Xia Y, Tang W.Long none coding RNA HOTTIP/HOXA13 act as synergistic role by decreasing cell migration and proliferation inHirschsprung disease.BIOCHEM BIOPH RES CO . 2015 Aug 7;463(4):569-74

44. Sun H, Meng Q, Tang S, Su J, Yin Q, Chen L, Gu W, Yu H, Zhang Z, Wang S, Li Y.Inhibition of Breast Cancer Metastasis by Pluronic Copolymers with Moderate Hydrophilic - Lipophilic Balance.MOL PHARMACOL . 2015 Sep 8;12(9):3323-31

45. Huo W, Cai P, Chen M, Li H, Tang J, Xu C, Zhu D, Tang W, Xia Y.The relationship between prenatal exposure to BP-3 and Hirschsprung's disease.Chemosphere . 2015 Oct 7;144:1091-1097

46. Zhang L, Cheng X, Gao Y, Bao J, Guan H, Lu R, Yu H, Xu Q, Sun Y.Induction of ROS-independent DNA damage by curcumin leads to G2/M cell cycle arrest and apoptosis in humanpapillary thyroid carcinoma BCPAP cells.Food Funct . 2015 Oct;1:315-25

47. Qiu M, Chen L, Tan G, Ke L, Zhang S, Chen H, Liu J.A reactive oxygen species activation mechanism contributes to JS-K-induced apoptosis in human bladder cancer cells.SCI REP-UK .2015 Oct 13;5:15104

48. Shi S, Zhang M, Guo R, Zhang M, Hu J, Xi Y, Miao Y, Li B.131I therapy mediated by sodium/iodide symporter combined with kringle 5 hasa synergistic therapeutic effect onglioma.Oncol Rep .2015 Nov;35(2):691-698

49. Cao J, Liu J, Xu R, Zhu X, Liu L, Zhao X.MicroRNA-21 stimulates epithelial-to-mesenchymal transition and tumorigenesis in clear cell renal cells.Mol Med Rep .2015 Nov,13(1):75-82

50. 50. Guo Q, Qin W.DKK3 blocked translocation of β-catenin/EMT induced by hypoxia and improved gemcitabine therapeutic effect inpancreatic cancer Bxpc-3 cell.J Cell Mol Med . 2015 Dec;19(12):2832-41

51. Zou L, Chen F, Bao J, Wang S, Wang L, Chen M, He C, Wang Y.Preparation, characterization, and anticancer efficacy of evodiamine-loaded PLGA nanoparticles.Drug Deliv . 2016;23(3):908-16

52. Lei H, Li H, Xie H, Du C, Xia Y, Tang W.Role of MiR-215 in Hirschsprung's Disease Pathogenesis by Targeting SIGLEC-8.CELL PHYSIOL BIOCHEM . 2016;40(6):1646-1655

53. Gai JQ, Sheng X, Qin JM, Sun K, Zhao W, Ni L.The effect and mechanism of bufalin on regulating hepatocellular carcinoma cell invasion andmetastasis via Wnt/β-catenin signaling pathway.Int J Oncol . 2016 Jan;48(1):338-48

54. Yu Zhou, Xing-Hui Li, Cai-Cai Zhang, Ming-Jie Wang, Wen-Long Xue, Dong-Dong Wu, Fen-Fen Ma, Wen-Wen Li, Bei-Bei Tao, Yi-Chun Zhu.Hydrogen sulfide promotes angiogenesis by down-regulating miR-640 via the VEGFR2/mTOR pathway.AM J PHYSIOL-CELL PH . 2016 Feb 15;310(4):C305-17

55. Zhang H, Zhong X, Zhang X, Shang D, Zhou YI, Zhang C .Enhanced anticancer effect of ABT-737 in combination with naringenin on gastric cancer cells.Exp Ther Med . 2016 Feb;11(2):669-673

56. Shi S, Zhang M, Guo R, Zhang M, Hu J, Xi Y, Miao Y, Li B .131I therapy mediated by sodium/iodide symporter combined with kringle 5 has a synergistic therapeuticeffect on glioma.Oncol Rep . 2016 Feb;35(2):691-8.

57. Tian L, Lu ZP, Cai BB, Zhao LT, Qian D, Xu QC, Wu PF, Zhu Y, Zhang JJ, Du Q, Miao Y, Jiang KR.Activation of pancreatic stellate cells involves an EMT-like process.Int J Oncol . 2016 Feb;48(2):783-92.

58. Zhou Y, Li XH, Zhang CC, Wang MJ, Xue WL, Wu DD, Ma FF, Li WW, Tao BB, Zhu YC.Hydrogen sulfide promotes angiogenesis by downregulating miR-640 via the VEGFR2/mTOR pathway.AM J PHYSIOL-CELL PH . 2016 Feb 15;310(4):C305-17

59. Xia Y, Weng B, Wang Z, Kang Y, Shi L, Huang G, Ying S, Du X, Chen Q, Jin R, Wu J, Liang G.W346 inhibits cell growth, invasion, induces cycle arrest and potentiates apoptosis in human gastric cancer cellsin vitro through the NF-κB signaling pathway.TUMOR BIOL .2016 Apr;37(4):4791–801

60. Xia Y, Weng B, Wang Z, Kang Y, Shi L, Huang G, Ying S, Du X, Chen Q.W346 inhibits cell growth, invasion, induces cycle arrest and potentiates apoptosis in human gastriccancer cells in vitro through the NF-κB signaling pathway.TUMOR BIOL . 2016 Apr;37(4):4791-801

61. Shen Z, Peng L, Zhu Z, Xie H, Zang R, Du C, Chen G, Li H, Xia Y, Tang W.Downregulated Expression of Long Non-Coding RNA LOC101926975 Impairs both Cell Proliferation andCell Cycle and Its Clinical Implication in Hirschsprung Disease Patients.Int J Med Sci . 2016 Apr 8;13(4):292-7

62. Fu JP, Mo WC, Liu Y, He RQ.Decline of cell viability and mitochondrial activity in mouse skeletal muscle cell in a hypomagnetic field.Bioelectromagnetics . 2016 May;37(4):212-22

63. Xi Z, Yao M, Li Y, Xie C, Holst J, Liu T, Cai S, Lao Y, Tan H, Xu HX, Dong Q.Guttiferone K impedes cell cycle re-entry of quiescent prostate cancer cells via stabilization of FBXW7and subsequent c-MYC degradation.Cell Death Dis . 2016 Jun 2;7(6):e2252

64. Liu S, Sun J, Cai B, Xi X, Yang L, Zhang Z, Feng Y, Sun Y.NANOG regulates epithelial-mesenchymal transition and chemoresistance through activation of theSTAT3 pathway in epithelial ovarian cancer.TUMOR BIOL . 2016 Jul;37(7):9671-80

65. Tang W, Cai P, Huo W, Li H, Tang J, Zhu D, Xie H, Chen P, Hang B, Wang S, Xia Y.Suppressive action of miRNAs to ARP2/3 complex reduces cell migration and proliferation via RACisoforms in Hirschsprung disease.J Cell Mol Med . 2016 Jul;20(7):1266-75. (IF 4.486)

66. Jin H, Chen H, Yu K, Zhang J, Li B, Cai N, Pan J.Resveratrol inhibits phosphorylation within the signal transduction and activator oftranscription 3 signaling pathway by activating sirtuin 1 in SW1353 chondrosarcoma cells.Mol Med Rep . 2016 Sep;14(3):2685-90

67. Xiong Y, Ji W, Fei Y, Zhao Y, Wang L, Wang W, Han M, Tan C, Fei X, Huang Q, Liang Z.Cathepsin L is involved in X-ray-induced invasion and migration of human glioma U251 cells.Cell Signal . 2016 Oct 29. pii: S0898-6568(16)30256-X

68. Lang M, Zhou Z, Shi L, Niu J, Xu S, Lin W, Chen Z, Wang Y.Influence of zoledronic acid on proliferation, migration, and apoptosis of vascular endothelial cells.BRIT J ORAL MAX SURG . 2016 Oct;54(8):889-893

69. Zhang M, Shi S, Guo R, Miao Y, Li B.Use of rhenium-188 for in vivo imaging and treatment of human cervical cancer cells transfected withlentivirus expressing sodium iodide symporter.Oncol Rep . 2016 Oct;36(4):2289-97

70. Kong F, Hu W, Zhou K, Wei X, Kou Y, You H, Zheng K, Tang R.Hepatitis B virus X protein promotes interleukin-7 receptor expression via NF-κB and Notch1 pathway tofacilitate proliferation and migration of hepatitis B virus-related hepatoma cells.J EXP CLIN CANC RES . 2016 Nov 7;35(1):172

71. Du C, Xie H, Zang R, Shen Z, Li H, Chen P, Xu X, Xia Y, Tang W.Apoptotic neuron-secreted HN12 inhibits cell apoptosis in Hirschsprung's disease.INT J NANOMED . 2016 Nov 7;11:5871-5881

72. Xu X, Gao Y, Wen L, Zhai Z, Zhang S, Shan F, Feng J.Methionine enkephalin regulates microglia polarization and function.Int Immunopharmacol . 2016 Nov;40:90-97

73. Du C, Shen Z, Zang R, Xie H, Li H, Chen P, Hang B, Xu X, Tang W, Xia Y .Negative feedback circuitry between MIR143HG and RBM24 in Hirschsprung disease.BBA-BIOMEMBRANES . 2016 Nov;1862(11):2127-2136

74. Mehmood T, Maryam A, Zhang H, Li Y, Khan M, Ma T.Deoxyelephantopin induces apoptosis in HepG2 cells via oxidative stress, NF-κB inhibition andmitochondrial dysfunction.Biofactors . 2017 Jan 2;43(1):63-72

75. Peng L, Chen G, Zhu Z, Shen Z, Du C, Zang R, Su Y, Xie H, Li H, Xu X, Xia Y, Tang W.Circular RNA ZNF609 functions asa competitive endogenous RNA to regulate AKT3 expression by sponging miR-150-5p in Hirschsprung's disease.ONCOTARGET . 2017 Jan 3;8(1):808-818

76. Zhou Y, Zhang J, Wang L, Chen Y, Wan Y, He Y, Jiang L, Ma J, Liao R, Zhang X, Shi L, Qin Z, Zhou Y, Chen Z, Hu W.Interleukin-1β impedes oligodendrocyte progenitor cell recruitment and white matter repair followingchronic cerebral hypoperfusion.Brain Behav Immun . 2017 Feb;60:93-105

77. Li J, Qiu M, Chen L, Liu L, Tan G, Liu J.Resveratrol promotes regression of renal carcinoma cells via a renin-angiotensin systemsuppression-dependent mechanism.Oncol Lett . 2017 Feb;13(2):613-620

78. Zhou Y, Zhang J, Wang L, Chen Y, Wan Y, He Y, Jiang L, Ma J, Liao R, Zhang X, Shi L, Qin Z, Zhou Y, Chen Z, Hu W.Interleukin-1β impedes oligodendrocyte progenitor cell recruitment and white matter repairfollowing chronic cerebral hypoperfusion.Brain Behav Immun . 2017 Feb;60:93-105

79. Dai F, Xuan Y, Jin JJ, Yu S, Long ZW, Cai H, Liu XW, Zhou Y, Wang YN, Chen Z, Huang H.CtBP2 overexpression promotes tumor cell proliferation and invasion in gastric cancer andis associated with poor prognosis.ONCOTARGET . 2017 Apr 25;8(17):28736-28749

80. Qiu M, Liang Z, Chen L, Tan G, Liu L, Wang K, Chen H, Liu J.MicroRNA-200c suppresses cell growth and metastasis by targeting Bmi-1 and E2F3 in renalcancer cells.Exp Ther Med . 2017 Apr;13(4):1329-1336

81. Huang X, Luo C, Lin L, Zhang L, Li H, Yao K, Xu Z.UV-assisted treatment on hydrophobic acrylic IOLs anterior surface with methacryloyloxyethylphosphorylcholine: Reducing inflammation and maintaining low posterior capsular opacification properties.MAT SCI ENG C-MATER . 2017 Jun 1;75:1289-1298

82. Zhou D, Zhang L, Sun W, Guan W, Lin Q, Ren W, Zhang J, Xu G.Cytidine monophosphate kinase is inhibited by the TGF-β signalling pathway through the upregulation of miR-130b-3p in human epithelial ovarian cancer.Cell Signal . 2017 Jul;35:197-207

83. Deng L, Tang J, Yang H, Cheng C, Lu S, Jiang R, Sun B.MTA1 modulated by miR-30e

contributes to epithelial-to-mesenchymal transition in hepatocellular carcinoma through an ErbB2-dependent pathway.Oncogene . 2017 Jul 13;36(28):3976-3985

84. Li X, Jin Q, Yao Q, Zhou Y, Zou Y, Li Z, Zhang S, Tu C.Placental Growth Factor Contributes to Liver Inflammation, Angiogenesis, Fibrosis in Mice by Promoting Hepatic Macrophage Recruitment and Activation.Front Immunol . 2017 Jul 11;8:801

85. Qiu M, Ke L, Zhang S, Zeng X, Fang Z, Liu J.JS-K,a GST-activated nitric oxide donor prodrug, enhances chemo-sensitivity in renal carcinomacells and prevents cardiac myocytes toxicity induced by Doxorubicin.CANCER CHEMOTH PHARM . 2017 Aug;80(2):275-286

86. Yu K, Wang T, Li Y, Wang C, Wang X, Zhang M, Xie Y, Li S, An Z, Ye T.Niclosamide induces apoptosis through mitochondrial intrinsic pathway and inhibits migration and invasion in human thyroid cancer in vitro.Biomed Pharmacother . 2017 Aug;92:403-411

87. Wei ML, Duan P, Wang ZM, Ding M, Tu P.High glucose and high insulin conditions promote MCF 7 cell proliferation and invasion by upregulating IRS1 and activating the Ras/Raf/ERK pathway.Mol Med Rep . 2017 Nov;16(5):6690-6696

88. Tang JY,Yu CY,Bao YJ,Chen L,Chen J,Yang SL,Chen HY,Hong J,Fang JY.TEAD4 promotes colorectal tumorigenesis via transcriptionally targeting YAP1.Cell Cycle. 2018;17(1):102-109

89. Su Y,Wen Z,Shen Q,Zhang H,Peng L,Chen G,Zhu Z,Du C,Xie H,Li H,Xia Y,Tang W.Long non-coding RNA LOC100507600 functions as a competitive endogenous RNA to regulate BMI1 expression by sponging miR128-1-3p in Hirschsprung's disease.Cell Cycle. 2018;17(4):459-467

90. Xu S,Zhang L,Cheng X,Yu H,Bao J,Lu R.Capsaicin inhibits the metastasis of human papillary thyroid carcinoma BCPAP cells through the modulation of the TRPV1 channel.Food Funct . 2018 Jan 24;9(1):344-354

91. Lin Y,Chen Q,Liu QX,Zhou D,Lu X,Deng XF,Yang H,Zheng H,Qiu Y.High expression of DJ-1 promotes growth and invasion via the PTEN-AKT pathway and predicts a poor prognosis in colorectal cancer.CANCER MED-US. 2018 Mar;7(3):809-819

92. Ye C,Yu X,Liu X,Zhan P,Nie T,Guo R,Liu H,Dai M,Zhang B.Beclin-1 knockdown decreases proliferation, invasion and migration of Ewing sarcoma SK-ES-1 cells via inhibition of MMP-9.Oncol Lett. 2018 Mar;15(3):3221-3225

93. Bai E, Yang L, Xiang Y, Hu W, Li C, Lin J, Dai X, Liang G, Jin R, Zhao C.L61H46 shows potent efficacy against human pancreatic cancer through inhibiting STAT3 pathway.Cancer Manag Res. 2018 Mar 23;10:565-581

94. Fei Y,Xiong Y,Shen X,Zhao Y,Zhu Y,Wang L,Liang Z.Cathepsin L promotes ionizing radiation-induced U251 glioma cell migration and invasion through regulating the GSK-3β/CUX1 pathway.Cell Signal. 2018 Apr;44:62-71

95. Ye C,Yu X,Liu X,Dai M,Zhang B.miR-30d inhibits cell biological progression of Ewing's sarcoma by suppressing the MEK/ERK and PI3K/Akt pathways in vitro.Oncol Lett. 2018 Apr;15(4):4390-4396

96. Zhang H,Chen W,Fu X,Su X,Yang A.CBX3 promotes tumor proliferation by regulating G1/S phase via p21 downregulation and associates with poor prognosis in tongue squamous cell carcinoma.Gene . 2018 May 15;654:49-56

97. Ma W,Feng L,Zhang S,Zhang H,Zhang X,Qi X,Zhang Y,Feng Q,Xiang T,Zeng YX.Induction of chemokine (C-C motif) ligand 5 by Epstein-Barr virus infection enhances tumor angiogenesis in nasopharyngeal carcinoma.Cancer Sci. 2018 May;109(5):1710-1722

98. Qiu D,Wang Q,Wang Z,Chen J,Yan D,Zhou Y,Li A,Zhang R,Wang S,Zhou J.RNF185 modulates JWA ubiquitination and promotes gastric cancer metastasis.BBA-MOL BASIS DIS. 2018 May;1864(5 Pt A):1552-1561

99. Luo Y,Chen X,Luo L,Zhang Q,Gao C,Zhuang X,Yuan S,Qiao T.[6]-Gingerol enhances the radiosensitivity of gastric cancer via G2/M phase arrest and apoptosis induction.Oncol Rep. 2018 May;39(5):2252-2260

100. Tian X,Guan W,Zhang L,Sun W,Zhou D,Lin Q,Ren W,Nadeem L,Xu G.Physical interaction of STAT1 isoforms with TGF-β receptors leads to functional crosstalk between two signaling pathways in epithelial ovarian cancer.J EXP CLIN CANC RES. 2018 May 11;37(1):103

101. Luo T,Yan Y,He Q,Ma X,Wang W.miR-328-5p inhibits MDA-MB-231 breast cancer cell proliferation by targeting RAGE.Oncol Rep . 2018 Jun;39(6):2906-2914

102. Zheng BY,Gao WY,Huang XY,Lin LY,Fang XF,Chen ZX,Wang XZ.HBx promotes the proliferative ability of HL-7702 cells via the COX-2/Wnt/β-catenin pathway.Mol Med Rep. 2018 Jun;17(6):8432-8438

103. Zhang LL,Feng ZL,Su MX,Jiang XM,Chen X,Wang Y,Li A,Lin LG,Lu JJ.Downregulation of Cyclin B1 mediates nagilactone E-induced G2 phase cell cycle arrest in non-small cell lung cancer cells.Eur J Pharmacol. 2018 Jul 5;830:17-25

104. Jiang C, He C, Wu Z, Li F, Xiao J.Histone methyltransferase SETD2 regulates osteosarcoma cell growth and chemosensitivity by suppressing Wnt/β-catenin signaling.BIOCHEM BIOPH RES CO. 2018 Jul 20;502(3):382-388

105. Zhou K,Rao J,Zhou ZH,Yao XH,Wu F,Yang J,Yang L,Zhang X,Cui YH,Bian XW,Shi Y,Ping YF.RAC1-GTP promotes epithelial-mesenchymal transition and invasion of colorectal cancer by activation of STAT3.Lab Invest. 2018 Aug;98(8):989-998.

106. Li S,Mei Z,Hu HB,Zhang X.The lncRNA MALAT1 contributes to non-small cell lung cancer development via modulating miR-124/STAT3 axis.J Cell Physiol . 2018 Sep;233(9):6679-6688

107. Lu C,Peng K,Guo H,Ren X,Hu S,Cai Y,Han Y,Ma L,Xu P.miR-18a-5p promotes cell invasion and migration of osteosarcoma by directly targeting IRF2.Oncol Lett. 2018 Sep;16(3):3150-3156

108. Yu M,Du G,Xu Q,Huang Z,Huang X,Qin Y,Han L,Fan Y,Zhang Y,Han X,Jiang Z,Xia Y,Wang X,Lu C.Integrated analysis of DNA methylome and transcriptome identified CREB5 as a novel risk gene contributing to recurrent pregnancy loss.EBioMedicine. 2018 Sep;35:334-344

109. Xiao C,Pan Y,Zeng X,Wang L,Li Z,Yan S,Wang H.Downregulation of hypoxia-inducible factor-1α inhibits growth, invasion, and angiogenesis of human salivary adenoid cystic carcinoma cells under hypoxia.Oncol Rep. 2018 Sep;40(3):1675-1683

110. Li XQ,Cai LM,Liu J,Ma YL,Kong YH,Li H,Jiang M.Liquiritin suppresses UVB-induced skin injury through prevention of inflammation, oxidative stress and apoptosis through the TLR4/MyD88/NF-κB and MAPK/caspase signaling pathways.Int J Mol Med. 2018 Sep;42(3):1445-1459

111. Wu Z,Cai L,Lu J,Wang C,Guan J,Chen X,Wu J,Zheng W,Wu Z,Li Q,Su Z.MicroRNA-93 mediates cabergoline-resistance by targeting ATG7 in prolactinoma.J Endocrinol. 2018 Sep 1;pii: JOE-18-0203.R1

112. Zheng S, Wang X, Weng YH, Jin X, Ji JL, Guo L, Hu B, Liu N, Cheng Q, Zhang J, Bai H, Yang T, Xia XH, Zhang HY, Gao S, Huang Y.siRNA Knockdown of RRM2 Effectively Suppressed Pancreatic Tumor Growth Alone or Synergistically with Doxorubicin.MOL THER-NUCL ACIDS. 2018 Sep 7;12:805-816

113. Cai P,Li H,Huo W,Zhu H,Xu C,Zang R,Lv W,Xia Y,Tang W.Aberrant expression of LncRNA-MIR31HG regulates cell migration and proliferation by affecting miR-31 and miR-31* in Hirschsprung's disease.J Cell Biochem. 2018 Nov;119(10):8195-8203

114. Zhao T,Wei X,Yang J,Wang S,Zhang Y.Galactoside-binding lectin in Solen grandis as a pattern recognition receptor mediating opsonization.FISH SHELLFISH IMMUN. 2018 Nov;82:183-189. (IF 3.298)

115. Li Z,Lu Q,Zhu D,Han Y,Zhou X,Ren T.Lnc-SNHG1 may promote the progression of non-small cell lung cancer by acting as a sponge of miR-497.BIOCHEM BIOPH RES CO. 2018 Nov 30;506(3):632-640

116. Tian Y,Yang T,Yu S,Liu C,He M,Hu C.Prostaglandin E2 increases migration and proliferation of human glioblastoma cells by activating transient receptor potential melastatin 7 channels.J Cell Mol Med. 2018 Dec;22(12):6327-6337

117. Zhang M,Wang J,Gao T,Chen X,Xu Y,Yu X,Guo X,Zhuang R,Li Z,Wu H,Yu J.Inhibition of SOX15 Sensitizes Esophageal Squamous Carcinoma Cells to Paclitaxel.Curr Mol Med. 2019;19(5):349-356

118. Li B,Wang Z,Xie JM,Wang G,Qian LQ,Guan XM,Shen XP,Qin ZH,Shen GH,Li XQ,Gao QG.TIGAR knockdown enhanced the anticancer effect of aescin via regulating autophagy and apoptosis in colorectal cancer cells.Acta Pharmacol Sin. 2019 Jan;40(1):111-121

119. Hu T,Zhang J,Sha B,Li M,Wang L,Zhang Y,Liu X,Dong Z,Liu Z,Li P,Chen P.Targeting the overexpressed USP7 inhibits esophageal squamous cell carcinoma cell growth by inducing NOXA-mediated apoptosis.MOL CARCINOGEN. 2019 Jan;58(1):42-54.

120. Wang XL,Shi M,Xiang T,Bu YZ.Long noncoding RNA DGCR5 represses hepatocellular carcinoma progression by inactivating Wnt signaling pathway.J Cell Biochem. 2019 Jan;120(1):275-282

121. Zhou JL,Deng S,Fang HS,Yu G,Peng H.Hsa-let-7g promotes osteosarcoma by reducing HOXB1 to activate NF-kB pathway.Biomed Pharmacother. 2019 Jan;109:2335-2341

122. Xu CM,Chen LX,Gao F,Zhu MF,Dai Y,Xu Y,Qian WX.MiR-431 suppresses proliferation and metastasis of lung cancer via down-regulating DDX5.EUR REV MED PHARMACO. 2019 Jan;23(2):699-707

123. Li JF,Li WH,Xue LL,Zhang Y.Long non-coding RNA PICART1 inhibits cell proliferation by regulating the PI3K/AKT and MAPK/ERK signaling pathways in gastric cancer.EUR REV MED PHARMACO. 2019 Jan;23(2):588-597

124. Qiu M,Zhang S,Ke L,Tang H,Zeng X,Liu J.JS-K enhances chemosensitivity of prostate cancer cells to Taxol via reactive oxygen species activation..Oncol Lett. 2019 Jan;17(1):757-764

125. Ai X,Lei Q,Shen X,Li J,Cen C,Xie G,Luo B,Zhu W,Hu G,Wu H,Chen M,Li X,Lin C.Long non-coding RNA ENST00000547547 inhibits cell proliferation, invasion and migration in colorectal cancer cells.Oncol Rep. 2019 Jan;41(1):483-491

126. Zhang Y,Wang G.MicroRNA-183 inhibits A375 human melanoma cell migration and invasion by targeting Ezrin and MMP-9.Oncol Lett. 2019 Jan;17(1):548-554

127. Li D,Li X,Li G,Meng Y,Jin Y,Shang S,Li Y.Alpinumisoflavone causes DNA damage in Colorectal Cancer Cells via blocking DNA repair mediated by RAD51.Life Sci. 2019 Jan 1;216:259-270

128. Sun S,Wang N,Sun Z,Wang X,Cui H.MiR-5692a promotes proliferation and inhibits apoptosis by targeting HOXD8 in hepatocellular carcinoma.J BUON. 2019 Jan-Feb;24(1):178-186

129. Limei Zhu,Ran Wang,Li Zhang,Chunlei Zuo,Rui Zhang,Shaolin Zhao.rs187960998 Polymorphism in miR-211 Prevents Development of Human Colon Cancer by Deregulation of 3'UTR in CHD5 ONCOTARGETS THER. 2019 Jan 3;12:405-412.;doi: 10.2147/OTT.S180935

130. Li BY,He LJ,Zhang XL,Liu H,Liu B.High expression of RAB38 promotes malignant progression of pancreatic cancer.Mol Med Rep. 2019 Feb;19(2):909-918

131. Hu L,Sun F,Sun Z,Ni X,Wang J,Wang J,Zhou M,Feng Y,Kong Z,Hua Q,Yu J.Apatinib enhances the radiosensitivity of the esophageal cancer cell line KYSE-150 by inducing apoptosis and cell cycle redistribution.Oncol Lett. 2019 Feb;17(2):1609-1616.

132. Yang X,Gao J,Guo J,Zhao Z,Zhang SL,He Y.Anti-lung cancer activity and inhibitory mechanisms of a novel Calothrixin A derivative.Life Sci. 2019 Feb 15;219:20-30

133. Ma C,Gao T,Ju J,Zhang Y,Ni Q,Li Y,Zhao Z,Chai J,Yang X,Sun M.Sympathetic innervation contributes to perineural invasion of salivary adenoid cystic carcinoma via the β2-adrenergic receptor.ONCOTARGETS THER. 2019 Feb 21;12:1475-1495

134. Ye T,Yang M,Huang D,Wang X,Xue B,Tian N,Xu X,Bao L,Hu H,Lv T,Huang Y.MicroRNA-7 as a potential therapeutic target for aberrant NF-κB-driven distant metastasis of gastric cancer.J EXP CLIN CANC RES. 2019 Feb 6;38(1):55

135. Jiang W,He Y,Shi Y,Guo Z,Yang S,Wei K,Pan C,Xia Y,Chen Y.MicroRNA-1204 promotes cell proliferation by regulating PITX1 in non-small-cell lung cancer.Cell Biol Int. 2019 Mar;43(3):253-264

136. Zhang DL,Yang N.MiR-3174 functions as an oncogene in rectal cancer by targeting PCBD2.EUR REV MED PHARMACO. 2019 Mar;23(6):2417-2426

137. Du P,Gao K,Cao Y,Yang S,Wang Y,Guo R,Zhao M,Jia S.RFX1 downregulation contributes to TLR4 overexpression in CD14+ monocytes via epigenetic mechanisms in coronary artery disease.Clin Epigenetics. 2019 Mar 11;11(1):44

138. Qi Y,Zha W,Zhang W.Exosomal miR-660-5p promotes tumor growth and metastasis in non-small cell lung cancer.J BUON. 2019 Mar-Apr;24(2):599-607

139. Hu B,Shi G,Li Q,Li W,Zhou H.Long noncoding RNA XIST participates in bladder cancer by downregulating p53 via binding to TET1.J Cell Biochem. 2019 Apr;120(4):6330-6338

140. Xiao Y,Li ZH,Bi YH.MicroRNA-889 promotes cell proliferation in colorectal cancer by targeting DAB2IP.EUR REV MED PHARMACO. 2019 Apr;23(8):3326-3334
141. Zhou J,Zhang J,Xu M,Ke Z,Zhang W,Mai J.High SRC-1 and Twist1 expression predicts poor prognosis and promotes migration and invasion by inducing epithelial-mesenchymal transition in human nasopharyngeal carcinoma.PLoS One. 2019 Apr 11;14(4):e0215299
142. Li M,Liu Y,Wei Y,Wu C,Meng H,Niu W,Zhou Y,Wang H,Wen Q,Fan S,Li Z,Li X,Zhou J,Cao K,Xiong W,Zeng Z,Li X,Qiu Y,Li G,Zhou M.Zinc-finger protein YY1 suppresses tumor growth of human nasopharyngeal carcinoma by inactivating c-Myc-mediated microRNA-141 transcription.J Biol Chem. 2019 Apr 12;294(15):6172-6187
143. Li B,Sun C,Sun J,Yang MH,Zuo R,Liu C,Lan WR,Liu MH,Huang B,Zhou Y.Autophagy mediates serum starvation-induced quiescence in nucleus pulposus stem cells by the regulation of P27.Stem Cell Res Ther. 2019 Apr 15;10(1):118
144. Yu Z,Li Y,Han T,Liu Z.Demethylation of the HACE1 gene promoter inhibits the proliferation of human liver cancer cells.Oncol Lett. 2019 May;17(5):4361-4368
145. Yang D,Han Y,Liu Y,Cao R,Wang Q,Dong Z,Liu H,Zhang X,Zhang Q,Zhao J.A peptidoglycan recognition protein involved in immune recognition and immune defenses in Ruditapes philippinarum.FISH SHELLFISH IMMUN. 2019 May;88:441-448
146. Xue YN,Yu BB,Liu YN,Guo R,Li JL,Zhang LC,Su J,Sun LK,Li Y.Zinc promotes prostate cancer cell chemosensitivity to paclitaxel by inhibiting epithelial-mesenchymal transition and inducing apoptosis.Prostate. 2019 May;79(6):647-656
147. Li X,Zhang Y,Chai X,Zhou S,Zhang H,He J,Zhou R,Cai L,Chen L,Tao G.Overexpression of MEF2D contributes to oncogenic malignancy and chemotherapeutic resistance in ovarian carcinoma.Am J Cancer Res. 2019 May 1;9(5):887-905. eCollection 2019
148. Zhang G,Li X,Chen Q,Li J,Ruan Q,Chen YH,Yang X,Wan X.CD317 Activates EGFR by Regulating Its Association with Lipid Rafts.Cancer Res. 2019 May 1;79(9):2220-2231
149. Jiang B,Chen W,Qin H,Diao W,Li B,Cao W,Zhang Z,Qi W,Gao J,Chen M,Zhao X,Guo H.TOX3 inhibits cancer cell migration and invasion via transcriptional regulation of SNAI1 and SNAI2 in clear cell renal cell carcinoma.Cancer Lett. 2019 May 1;449:76-86
150. Tang X,Cai W,Cheng J,Lu P,Ma S,Chen C,Chen Y,Sun Y,Wang C,Hu P,Lv X,Sun G,Wang Y,Sheng J.The histone H3 lysine-27 demethylase UTX plays a critical role in colorectal cancer cell proliferation.Cancer Cell Int. 2019 May 22;19:144
151. Han X,Tang Y,Dai Y,Hu S,Zhou J,Liu X,Zhu J,Wu Y.MiR-889 promotes cell growth in human non-small cell lung cancer by regulating KLF9.Gene. 2019 May 30;699:94-101
152. Youjun Luo,Lin Zha,Lumeng Luo,Xue Chen,Qi Zhang,Caixia Gao,Xibing Zhuang,Sujuan Yuan,Tiankui Qiao.[6]-Gingerol Enhances the Cisplatin Sensitivity of Gastric Cancer Cells Through Inhibition of Proliferation and Invasion via PI3K/AKT Signaling Pathway Phytother Res. 2019 May;33(5):1353-1362.;doi: 10.1002/ptr.6325
153. Shi N,Shan B,Gu B,Song Y,Chu H,Qian L.Circular RNA circ-PRKCI functions as a competitive endogenous RNA to regulate AKT3 expression by sponging miR-3680-3p in esophageal squamous cell carcinoma.J Cell Biochem. 2019 Jun;120(6):10021-10030
154. Liu LD,Pang YX,Zhao XR,Li R,Jin CJ,Xue J,Dong RY,Liu PS.Curcumin induces apoptotic cell death and protective autophagy by inhibiting AKT/mTOR/p70S6K pathway in human ovarian cancer cells.Arch Gynecol Obstet. 2019 Jun;299(6):1627-1639
155. Qiu CW,Liu ZY,Zhang FL,Zhang L,Li F,Liu SY,He JY,Xiao ZC.Post-stroke gastrodin treatment ameliorates ischemic injury and increases neurogenesis and restores the Wnt/β-Catenin signaling in focal cerebral ischemia in mice.Brain Res. 2019 Jun 1;1712:7-15
156. Zhang Y,Wang SS,Tao L,Pang LJ,Zou H,Liang WH,Liu Z,Guo SL,Jiang JF,Zhang WJ,Jia W,Li F.Overexpression of MAP3K3 promotes tumour growth through activation of the NF-κB signalling pathway in ovarian carcinoma.SCI REP-UK. 2019 Jun 10;9(1):8401
157. Liu F,Lou G,Zhang T,Chen S,Xu J,Xu L,Huang C,Liu Y,Chen Z.Anti-metastasis traditional Chinese medicine monomer screening system based on perinucleolar compartment analysis in hepatocellular carcinoma cells.Am J Transl Res. 2019 Jun 15;11(6):3555-3566. eCollection 2019
158. Yuan H,Qin Y,Zeng B,Feng Y,Li Y,Xiang T,Ren G.Long noncoding RNA LINC01089 predicts clinical prognosis and inhibits cell proliferation and invasion through the Wnt/β-catenin signaling pathway in breast cancer.ONCOTARGETS THER. 2019 Jun 21;12:4883-4895
159. Xu Y,Wang P,Xu C,Shan X,Feng Q.Acrylamide induces HepG2 cell proliferation through upregulation of miR-21 expression.BIOMED RES INT. 2019 Jun 4;33(3):181-191
160. Ge D,Gao J,Han L,Li Y,Liu HH,Yang WC,Chang F,Liu J,Yu M,Zhao J.Novel effects of sphingosylphosphorylcholine on the apoptosis of breast cancer via autophagy/AKT/p38 and JNK signaling.J Cell Physiol. 2019 Jul;234(7):11451-11462
161. Wen Z,Shen Q,Zhang H,Su Y,Zhu Z,Chen G,Peng L,Li H,Du C,Xie H,Xu X,Tang W.Circular RNA CCDC66 targets DCX to regulate cell proliferation and migration by sponging miR-488-3p in Hirschsprung's disease.J Cell Physiol. 2019 Jul;234(7):10576-10587
162. Yin Z,Ma T,Huang B,Lin L,Zhou Y,Yan J,Zou Y,Chen S.Macrophage-derived exosomal microRNA-501-3p promotes progression of pancreatic ductal adenocarcinoma through the TGFBR3-mediated TGF-β signaling pathway.J EXP CLIN CANC RES. 2019 Jul 15;38(1):310
163. Duan Q,Li D,Xiong L,Chang Z,Xu G.SILAC Quantitative Proteomics and Biochemical Analyses Reveal a Novel Molecular Mechanism by Which ADAM12S Promotes the Proliferation, Migration, and Invasion of Small Cell Lung Cancer Cells through U J Proteome Res. 2019 Jul 5;18(7):2903-2914
164. He H, Qie S, Guo Q, Chen S, Zou C, Lu T, Su Y, Zong J, Xu H, He D, Xu Y, Chen B, Pan J, Sang N, Lin S.Stanniocalcin 2 (STC2) expression promotes post-radiation survival, migration and invasion of nasopharyngeal carcinoma cells.Cancer Manag Res. 2019 Jul 11;11:6411-6424
165. Zhong C,Li X,Tao B,Peng L,Peng T,Yang X,Xia X,Chen L.LIM and SH3 protein 1 induces glioma growth and invasion through PI3K/AKT signaling and epithelial-mesenchymal transition.Biomed Pharmacother. 2019 Aug;116:109013
166. Zhang XH,Li M,Kang YJ,Xie YQ,Cao YX.Long non-coding RNA LINP1 functions as an oncogene in endometrial cancer progression by regulating the PI3K/AKT signaling pathway.EUR REV MED PHARMACO. 2019 Aug;23(16):6830-6838
167. Ruan L,Qian X.MiR-16-5p inhibits breast cancer by reducing AKT3 to restrain NF-κB pathway.BIOSCIENCE REP. 2019 Aug 23;39(8). pii: BSR20191611
168. Zhu Y,Zhao YF,Liu RS,Xiong YJ,Shen X,Wang Y,Liang ZQ.Olanzapine induced autophagy through suppression of NF-κB activation in human glioma cells.CNS Neurosci Ther. 2019 Sep;25(9):911-921
169. Guo S,Zhang R,Liu Q,Wan Q,Wang Y,Yu Y,Liu G,Shen Y,Yu Y,Zhang J.2,3,7,8-Tetrachlorodibenzo-p-dioxin promotes injury-induced vascular neointima formation in mice.FASEB J. 2019 Sep;33(9):10207-10217
170. Ding X,Fan K,Hu J,Zang Z,Zhang S,Zhang Y,Lin Z,Pei X,Zheng X,Zhu F,Yang H,Li S.SCP2-mediated cholesterol membrane trafficking promotes the growth of pituitary adenomas via Hedgehog signaling activation.J EXP CLIN CANC RES. 2019 Sep 13;38(1):404
171. Zhou R,Wang C,Liang Y,Li X,Li QAnti-miR-200b promotes wound healing by regulating fibroblast functions in a novel mouse model.ACTA BIOCH BIOPH SIN. 2019 Sep 6;51(10):1049-1055
172. Zhang F,Fan B,Mao L.Radiosensitizing effects of Cyclocarya paliurus polysaccharide on hypoxic A549 and H520 human non-small cell lung carcinoma cells.Int J Mol Med. 2019 Oct;44(4):1233-1242
173. Zhang L,Li B,Zhang B,Zhang H,Suo J.miR-361 enhances sensitivity to 5-fluorouracil by targeting the FOXM1-ABCC5/10 signaling pathway in colorectal cancer.Oncol Lett. 2019 Oct;18(4):4064-4073
174. Wan W,Wan W,Long Y,Li Q,Jin X,Wan G,Zhang F,Lv Y,Zheng G,Li Z,Zhu Y.MiR-25-3p promotes malignant phenotypes of retinoblastoma by regulating PTEN/Akt pathway.Biomed Pharmacother. 2019 Oct;118:109111
175. Guo F,Yuan D,Zhang J,Zhang H,Wang C,Zhu L,Zhang J,Pan Y,Shao C.Silencing of ARL14 Gene Induces Lung Adenocarcinoma Cells to a Dormant State.Front Cell Dev Biol. 2019 Oct 15;7:238
176. Yang Q,Deng Y,Xu Y,Ding N,Wang C,Zhao X,Lou X,Li Y,Zhao H,Fang X.Knockdown of SSATX, an alternative splicing variant of the SAT1 gene, promotes melanoma progression.Gene. 2019 Oct 20;716:144010
177. Lan X,Zhao J,Song C,Yuan Q,Liu X.TRPM8 facilitates proliferation and immune evasion of esophageal cancer cells.BIOSCIENCE REP. 2019 Oct 30;39(10). pii: BSR20191878
178. Gong C, Hu Y, Zhou M, Yao M, Ning Z, Wang Z, Ren J.Identification of specific modules and hub genes associated with the progression of gastric cancer.Carcinogenesis. 2019 Oct 16;40(10):1269-1277
179. Li Q,Xu A,Chu Y,Chen T,Li H,Yao L,Zhou P,Xu M.Rap1A promotes esophageal squamous cell carcinoma metastasis through the AKT signaling pathway.Oncol Rep. 2019 Nov;42(5):1815-1824
180. Zhu Q,Wu Y,Yang M,Wang Z,Zhang H,Jiang X,Chen M,Jin T,Wang T.IRX5 promotes colorectal cancer metastasis by negatively regulating the core components of the RHOA pathway.MOL CARCINOGEN. 2019 Nov;58(11):2065-2076
181. Liu L,Qu H,Qin H,Yang Y,Liao Z,Cui J,Gao F,Cai J.NOD2 agonist murabutide alleviates radiation-induced injury through DNA damage response pathway mediated by ATR.J Cell Physiol. 2019 Nov;234(11):21294-21306
182. Jin L,Wang Q,Chen J,Wang Z,Xin H,Zhang D.Efficient Delivery of Therapeutic siRNA by Fe3O4 Magnetic Nanoparticles into Oral Cancer Cells.Pharmaceutics. 2019 Nov 17;11(11). pii: E615
183. Liu X,Hou X,Zhou Y,Li Q,Kong F,Yan S,Lei S,Xiong L,He J.Downregulation of the Helicase Lymphoid-Specific (HELLS) Gene Impairs Cell Proliferation and Induces Cell Cycle Arrest in Colorectal Cancer Cells.ONCOTARGETS THER. 2019 Nov 26;12:10153-10163
184. Chen S,Liu Y,Wang Y,Xue Z.LncRNA CCAT1 Promotes Colorectal Cancer Tumorigenesis Via A miR-181b-5p/TUSC3 Axis.ONCOTARGETS THER. 2019 Nov 5;12:9215-9225
185. Wu H,Yu J,Kong D,Xu Y,Zhang Z,Shui J,Li Z,Luo H,Wang K.Population and single-cell transcriptome analyses reveal diverse transcriptional changes associated with radioresistance in esophageal squamous cell carcinoma.Int J Oncol. 2019 Dec;55(6):1237-1248
186. Liang Q,Zhang L,Wood RW,Ji RC,Boyce BF,Schwarz EM,Wang Y,Xing L.Avian Reticuloendotheliosis Viral Oncogene Related B Regulates Lymphatic Endothelial Cells during Vessel Maturation and Is Required for Lymphatic Vessel Function in Adult Mice.Am J Pathol. 2019 Dec;189(12):2516-2530
187. 187. Lin H,Li P,Zhang N,Cao L,Gao YF,Ping F.Long non-coding RNA MIR503HG serves as a tumor suppressor in non-small cell lung cancer mediated by wnt1.EUR REV MED PHARMACO. 2019 Dec;23(24):10818-10826
188. Chen J,Jiang J,Wang W,Qin J,Chen J,Chen W,Wang Y.Low intensity pulsed ultrasound promotes the migration of bone marrow- derived mesenchymal stem cells via activating FAK-ERK1/2 signalling pathway.ARTIF CELL NANOMED B. 2019 Dec;47(1):3603-3613
189. Zhu J,Zhao L,Luo B,Sheng W.Shikonin regulates invasion and autophagy of cultured colon cancer cells by inhibiting yes-associated protein.Oncol Lett. 2019 Dec;18(6):6117-6125
190. Hua S,Li M,Zhao Q,Wang J,Zhou Y,Liu J,Fang H,Jiang M,Shen L.Mitochondrial DNA Haplogroup N9a Negatively Correlates with Incidence of Hepatocellular Carcinoma in Northern China.MOL THER-NUCL ACIDS. 2019 Dec 6;18:332-340
191. Xiaodong Yan,Zhiyao Yuan,Yifeng Bian,Lei Jin,Zhao Mao,Jiang Lei,Ning Chen.Uncoupling protein-2 regulates M1 macrophage infiltration of gingiva with periodontitis Cent Eur J Immunol. 2020;45(1):9-21.;doi: 10.5114/ceji.2020.94664
192. Lin F,Yin HB,Li XY,Zhu GM,He WY,Gou X.Bladder cancer cell-secreted exosomal miR-21 activates the PI3K/AKT pathway in macrophages to promote cancer progression.Int J Oncol. 2020 Jan;56(1):151-164
193. Cao KY,Pan Y,Yan TM,Jiang ZH.Purification, characterization and cytotoxic activities of individual tRNAs from Escherichia coli.Int J Biol Macromol. 2020 Jan 1;142:355-365
194. Dongmei Ye,Junhui Zhu,Qiang Zhao,Wei Ma,Yiyang Xiao,Gaosheng Xu,Zhiwei Zhang.LMP1 Up-regulates Calreticulin to Induce Epithelial-mesenchymal Transition via TGF-β/Smad3/NRP1 Pathway in Nasopharyngeal Carcinoma Cells J Cancer. 2020 Jan 1;11(5):1257-1269.;doi: 10.7150/jca.37415
195. Chang Liu,Jian Wang,Li Zhao,Hui He,Pan Zhao,Zheng Peng,Feiyuan Liu,Juan

Chen,Weiqing Wu,Guangsuo Wang,Fajin Dong.Knockdown of Thymidine Kinase 1 Suppresses Cell Proliferation, Invasion, Migration, and Epithelial-Mesenchymal Transition in Thyroid Carcinoma Cells Front Oncol. 2020 Jan 29;9:1475.;doi: 10.3389/fonc.2019.01475

196. Jian Xu,Feng Tian,Xingtian Chen,Zhaoxia Liu,Chuanxia Wu,Zhijuan Zhao.Ras-ERK1/2 signaling participates in the progression of gastric cancer through repressing Aurora B-mediated H1.4 phosphorylation at Ser27 J Cell Physiol. 2020 Jan 7.;doi: 10.1002/jcp.29432

197. Jun Cui,Siyi Qi,Rongheng Liao,Diansan Su,Yongyi Wang,Song Xue.MiR-574-5p promotes the differentiation of human cardiac fibroblasts via regulating ARID3A BIOCHEM BIOPH RES CO. 2020 Jan 8;521(2):427-433.;doi: 10.1016/j.bbrc.2019.09.107

198. Shanshan Du,Shuai Wang,Fengyan Zhang,Yong Lv.SKP2, positively regulated by circ_ODC1/miR-422a axis, promotes the proliferation of retinoblastoma J Cell Biochem. 2020 Jan;121(1):322-331.;doi: 10.1002/jcb.29177

199. Xiangnan Kong,Min Li,Kai Shao,Yinrong Yang,Qian Wang,Meijuan Cai.Progesterone induces cell apoptosis via the CACNA2D3/Ca2+/p38 MAPK pathway in endometrial cancer Oncol Rep. 2020 Jan;43(1):121-132.;doi: 10.3892/or.2019.7396

200. Fan G,Xu P,Tu P.MiR-1827 functions as a tumor suppressor in lung adenocarcinoma by targeting MYC and FAM83F.J Cell Biochem. 2020 Feb;121(2):1675-1689

201. Zhou P,Xiong T,Yao L,Yuan J.MicroRNA-665 promotes the proliferation of ovarian cancer cells by targeting SRCIN1.Exp Ther Med. 2020 Feb;19(2):1112-1120

202. Zhu H,Yang Y,Wang L,Xu X,Wang T,Qian H.Leptomycin B inhibits the proliferation, migration, and invasion of cultured gastric carcinoma cells.BIOSCI BIOTECH BIOCH. 2020 Feb;84(2):290-296

203. Wu J,Chen L,Chen L,Fan L,Wang Z,Dong Z,Chen Q,Wei T,Cai Y,Li W.The discovery of potent and stable short peptide FGFR1 antagonist for cancer therapy.Eur J Pharm Sci. 2020 Feb 15;143:105179

204. Simin Shao,Haowei Cao,Zhongkun Wang,Dongmei Zhou,Chaoshen Wu,Shu Wang,Dian Xia,Daoyong Zhang.CHD4/NuRD complex regulates complement gene expression and correlates with CD8 T cell infiltration in human hepatocellular carcinoma Clin Epigenetics. 2020 Feb 18;12(1):31.;doi: 10.1186/s13148-020-00827-3

205. Lin Cong,Zhile Bai,Yang Du,Yong Cheng.Citron Rho-Interacting Serine/Threonine Kinase Promotes HIF1a-CypA Signaling and Growth of Human Pancreatic Adenocarcinoma Biomed Res Int. 2020 Feb 22;2020:9210891.;doi: 10.1155/2020/9210891

206. Xing Liu,Zhiguang Yang,Yiquan Li,Yilong Zhu,Wenjie Li,Shanzhi Li,Jing Wang,Yingli Cui,Chao Shang,Zirui Liu,Gaojie Song,Ce Li,Xiao Li,Guoguang Shao,Ningyi Jin.Chemovirotherapy of Lung Squamous Cell Carcinoma by Combining Oncolytic Adenovirus With Gemcitabine Front Oncol. 2020 Feb 25;10:229.;doi: 10.3389/fonc.2020.00229

207. Wei Lie Mo,Jing Ting Jiang,Luo Zhang,Qi Cheng Lu,Jing Li,Wei Dong Gu,Yun Cheng,Hai Tao Wang.Circular RNA hsa_circ_0000467 Promotes the Development of Gastric Cancer by Competitively Binding to MicroRNA miR-326-3p Biomed Res Int. 2020 Feb 6;2020:4030826.;doi: 10.1155/2020/4030826

208. Qian Zhang,Yiqian Xing,Shan Jiang,Chunmei Xu,Xiaojun Zhou,Rui Zhang,Tianyue Xie,Zhiwei Zou,Piyun Gong,Huangao Zhu,Dongmei Zhang,Huimei Ma,Lin Liao,Jianjun Dong.Integrated analysis identifies DUSP5 as a novel prognostic indicator for thyroid follicular carcinoma Thorac Cancer. 2020 Feb;11(2):336-345.;doi: 10.1111/1759-7714.13270

209. Xin-Yu Ding,Hai-Yang Hu,Ke-Nan Huang,Rong-Qiang Wei,Jie Min,Chen Qi,Hua Tang,Xiong Qin.Ubiquitination of NOTCH2 by DTX3 suppresses the proliferation and migration of human esophageal carcinoma Cancer Sci. 2020 Feb;111(2):489-501.;doi: 10.1111/cas.14288

210. Yiling Zhang,Ningning Cui,Gang Zheng.Ubiquitination of P53 by E3 ligase MKRN2 promotes melanoma cell proliferation Oncol Lett. 2020 Mar;19(3):1975-1984.;doi: 10.3892/ol.2020.11261

211. Yan Wang,Mingzhu Liu,Shenglan Chen,Qin Wu.Plantamajoside represses the growth and metastasis of malignant melanoma Exp Ther Med. 2020 Mar;19(3):2296-2302.;doi: 10.3892/etm.2020.8442

212. Fei Guo,Jun Xue.MicroRNA-628-5p inhibits cell proliferation and induces apoptosis in colorectal cancer through downregulating CCND1 expression levels Mol Med Rep. 2020 Mar;21(3):1481-1490.;doi: 10.3892/mmr.2020.10945

213. Xiang Tong,Huangjian Zeng,Pengchen Gu,Kai Wang,Han Zhang,Xiangjin Lin.Monocyte chemoattractant protein-1 promotes the proliferation, migration and differentiation potential of fibroblast-like synoviocytes via the PI3K/P38 cellular signaling pathway Mol Med Rep. 2020 Mar;21(3):1623-1632.;doi: 10.3892/mmr.2020.10969

214. Hongqi Xiao,Ming Liu.Circular RNA hsa_circ_0053277 promotes the development of colorectal cancer by upregulating matrix metallopeptidase 14 via miR-2467-3p sequestration J Cell Physiol. 2020 Mar;235(3):2881-2890.;doi: 10.1002/jcp.29193

215. Qiuyue Ding,Wenda Zhang,Cheng Cheng,Fengbo Mo,Lei Chen,Guangfu Peng,Xianyi Cai,Jinglong Wang,Shuhua Yang,Xianzhe Liu.Dioscin inhibits the growth of human osteosarcoma by inducing G2/M-phase arrest, apoptosis, and GSDME-dependent cell death in vitro and in vivo J Cell Physiol. 2020 Mar;235(3):2911-2924.;doi: 10.1002/jcp.29197

216. H-Y Yu,S-S Pan.MiR-202-5p suppressed cell proliferation, migration and invasion in ovarian cancer via regulating HOXB2 EUR REV MED PHARMACO. 2020 Mar;24(5):2256-2263.;doi: 10.26355/eurrev_202003_20491

217. Y-Q Liu,Y-Z Cong,J Jiang,J-Z Sheng,X-H Li,M Zhao,M-Y Peng.MiR-526b suppresses cell proliferation, cell invasion and epithelial-mesenchymal transition in breast cancer by targeting Twist1 EUR REV MED PHARMACO. 2020 Mar;24(6):3113-3121.;doi: 10.26355/eurrev_202003_20678

218. J Shi,X Li,Y Hu,F Zhang,X Lv,X Zhang,Q Chen,S Hu.MiR-1203 is involved in hepatocellular carcinoma metastases and indicates a poor prognosis Neoplasma. 2020 Mar;67(2):267-276.;doi: 10.4149/neo_2019_190414N328

219. Li Z,Yang C,Li X,Du X,Tao Y,Ren J,Fang F,Xie Y,Li M,Qian G,Xu L,Cao X,Wu Y,Lv H,Hu S,Lu J,Pan J.The dual role of BI 2536, a small-molecule inhibitor that targets PLK1, in induction of apoptosis and attenuation of autophagy in neuroblastoma cells.J Cancer. 2020 Mar 5;11(11):3274-3287

220. Juan Luo,Beilei Zeng,Chunfang Tao,Mengqi Lu,Guosheng Ren.ClpP regulates breast cancer cell proliferation, invasion and apoptosis by modulating the Src/PI3K/Akt signaling pathway PeerJ. 2020 Mar 10;8:e8754.;doi: 10.7717/peerj.8754

221. Ding Li,Xiao-Feng Ni,Hengjie Tang,Jiecheng Zhang,Chenlei Zheng,Jianhu Lin,Cheng Wang,Linxiao Sun,Bicheng Chen. KRT17 Functions as a Tumor Promoter and Regulates Proliferation, Migration and Invasion in Pancreatic Cancer via mTOR/S6k1 Pathway。Cancer Manag Res. 2020 Mar 19;12:2087-2095.;doi: 10.2147/CMAR.S243129

222. Yubin Chen,Fen Liu,Fei Han,Lizhi Lv,Can-E Tang,Fanyan Luo.Omentin-1 Ameliorated Free Fatty Acid-Induced Impairment in Proliferation, Migration, and Inflammatory States of HUVECs Cardiol Res Pract. 2020 Mar 27;2020:3054379.;doi: 10.1155/2020/3054379

223. Yuwan Zhao,Qiuming Luo,Jierong Mo,Jianwei Li,Dongcai Ye,Zhixian Ao,Lixin Chen,Jianjun Liu.Metformin in combination with JS-K inhibits growth of renal cell carcinoma cells via reactive oxygen species activation and inducing DNA breaks J Cancer. 2020 Mar 31;11(13):3701-3712.;doi: 10.7150/jca.36372

224. Jing Yang,Kun Li,Jian Chen,Xiaoxiong Hu,He Wang,Xuan Zhu.Long Noncoding RNA LINC00460 Promotes Hepatocellular Carcinoma Progression via Regulation of miR-342-3p/AGR2 Axis ONCOTARGETS THER. 2020 Mar 6;13:1979-1991.;doi: 10.2147/OTT.S239258

225. Yang Liu,Yiling Ding,Yanting Nie,Mengyuan Yang.EMP1 Promotes the Proliferation and Invasion of Ovarian Cancer Cells Through Activating the MAPK Pathway ONCOTARGETS THER. 2020 Mar 9;13:2047-2055.;doi: 10.2147/OTT.S240028

226. Yang J,Li YH,He MT,Qiao JF,Sang Y,Cheang LH,Gomes FC,Hu Y,Li ZY,Liu N,Zhang HT,Zha ZG.HSP90 regulates osteosarcoma cell apoptosis by targeting the p53/TCF-1-mediated transcriptional network.J Cell Physiol. 2020 Apr;235(4):3894-3904

227. Dongqiang Song,Beili Xu,Dongmin Shi,Shuyu Li,Yu Cai.S100A6 promotes proliferation and migration of HepG2 cells via increased ubiquitin-dependent degradation of p53 Open Med (Wars). 2020 Apr 20;15(1):317-326.;doi: 10.1515/med-2020-0101.

228. Xiaodong Li,Qingshan Huang,Shenglin Wang,Zhen Huang,Fengqiang Yu,Jianhua LinHER4 promotes the growth and metastasis of osteosarcoma via the PI3K/AKT pathway ACTA BIOCH BIOPH SIN. 2020 Apr 20;52(4):345-362.;doi: 10.1093/abbs/gmaa004

229. Kun Yao,Leye He,Yu Gan,Jianye Liu,Jin Tang,Zhi Long,Jing Tan.HMGN5 promotes IL-6-induced epithelial-mesenchymal transition of bladder cancer by interacting with Hsp27 AGING-US. 2020 Apr 21;12(8):7282-7298.;doi: 10.18632/aging.103076

230. 230. Ruofei Huang,Wei Mao,Guoliang Wang,Jian Ding,Ying Sun,Gang Gao,Ping Dong,Zhenfeng Sun.Synergistic relationship between TSLP and IL-33/ST2 signaling pathways in allergic rhinitis and the effects of hypoxia INT FORUM ALLERGY RH. 2020 Apr;10(4):511-520.;doi: 10.1002/alr.22504

231. Xiaojin Yang,Hanchuan Tao,Cheng Wang,Weijun Chen,Fu Hua,Haixin Qian.lncRNA-ATB promotes stemness maintenance in colorectal cancer by regulating transcriptional activity of the β-catenin pathway Exp Ther Med. 2020 Apr;19(4):3097-3103.;doi: 10.3892/etm.2020.8558

232. Shihua Zhang,Kejian Wang.miR-383 Down-Regulates the Oncogene CIP2A to Influence Glioma Proliferation and Invasion ONCOTARGETS THER. 2020 May 12;13:4063-4074.;doi: 10.2147/OTT.S248116

233. Jianjun Li,Zhihao Li,Chengqiang Wang,Zhijia Li,Haixia Xu,Yunteng Hu,Zhiwen Tan,Fu Zhang,Chun Liu,Minsheng Yang,Yihan Wang,Yanglei Jin,Ziyue Peng,Sourabh Biswas,Lixin Zhu.The Regulatory Effect of VEGF-Ax on Rat Bone Marrow Mesenchymal Stem Cells' Angioblastic Differentiation and Its Proangiogenic Ability Stem Cells Dev. 2020 May 15;29(10):667-677.;doi: 10.1089/scd.2019.0198

234. Hui Feng,Qi Wang,Wenjing Xiao,Biyuan Zhang,Yonglong Jin,Haijun Lu.LncRNA TTN-AS1 Regulates miR-524-5p and RRM2 to Promote Breast Cancer Progression ONCOTARGETS THER. 2020 May 27;13:4799-4811.;doi: 10.2147/OTT.S243482

235. Xiaojiao Zheng,Kejun Xu,Linyan Zhu,Meiya Mao,Fubin Zhang,Lining Cui.MiR-486-5p Act as a Biomarker in Endometrial Carcinoma: Promotes Cell Proliferation, Migration, Invasion by Targeting MARK1 ONCOTARGETS THER. 2020 May 28;13:4843-4853.;doi: 10.2147/OTT.S246841

236. Wenkai Wang,Rui Zuo,Haixia Long,Yanqiu Wang,Yang Zhang,Chao Sun,Gang Luo,Yuan Zhang,Changqing Li,Yue Zhou,Jie Li.Advances in the Masquelet technique: Myeloid-derived suppressor cells promote angiogenesis in PMMA-induced membranes Acta Biomater. 2020 May;108:223-236.;doi: 10.1016/j.actbio.2020.03.010

237. Fanhua Kong,Xi Liu,Yan Zhou,Xuyang Hou,Jun He,Qinglong Li,Xiongying Miao,Leping Yang.Downregulation of METTL14 increases apoptosis and autophagy induced by cisplatin in pancreatic cancer cells INT J BIOCHEM CELL B. 2020 May;122:105731.;doi: 10.1016/j.biocel.2020.105731

238. Xiaoting Xi,Yanni Yang,Jia Ma,Qianbo Chen,Yong Zeng,Junxian Li,Lin Chen,Yan Li.MiR-130a alleviated high-glucose induced retinal pigment epithelium (RPE) death by modulating TNF-α/SOD1/ROS cascade mediated pyroptosis Biomed Pharmacother. 2020 May;125:109924.;doi: 10.1016/j.biopha.2020.109924

239. Ya-Sai Sun,Kiran Thakur,Fei Hu,Carlos L Cespedes-Acuña,Jian-Guo Zhang,Zhao-Jun Wei.Icariside II suppresses cervical cancer cell migration through JNK modulated matrix metalloproteinase-2/9 inhibition in vitro and in vivo Biomed Pharmacother. 2020 May;125:110013.;doi: 10.1016/j.biopha.2020.110013

240. L Zheng,Y-T Liu,C-P Wu,J-T Jiang,L Zhang,Z-L Wang,Q-Y Wang.Long non-coding RNA linc01433 promotes tumorigenesis and progression in esophageal squamous cell carcinoma by sponging miR-1301 EUR REV MED PHARMACO. 2020 May;24(9):4785-4792.;doi: 10.26355/eurrev_202005_21167

241. Dandan Yi,Ru Wang,Xianbiao Shi,Lei Xu,Yiminu'er Yilihamu,Jianfeng Sang.METTL14 promotes the migration and invasion of breast cancer cells by modulating N6-methyladenosine and hsa-miR-146a-5p expression Oncol Rep. 2020 May;43(5):1375-1386.;doi: 10.3892/or.2020.7515

242. Yang Yang,Qin-Qin Wang,Oliver Bozinov,Ru-Xiang Xu,Yi-Lin Sun,Shan-Shan Wang.GSK-3 inhibitor CHIR99021 enriches glioma stem-like cells Oncol Rep. 2020 May;43(5):1479-1490.;doi: 10.3892/or.2020.7525

243. Xue-Yuan Li,Guo-Hui Huang,Qian-Kun Liu,Xi-Tao Yang,Kang Wang,Wen-Zheng Luo,Tian-Song Liang,Shan-Peng Yuan,Ying-Wei Zhen,Dong-Ming Yan.Porf-2 Inhibits

Tumor Cell Migration Through the MMP-2/9 Signaling Pathway in Neuroblastoma and Glioma Front Oncol. 2020 Jun 26;10:975.;doi: 10.3389/fonc.2020.00975

244. Hongwei Yang,Yonggang Huang,Jian He,Guangrui Chai,Yu Di,Aiyuan Wang,Dongmei Gui.MiR-486-3p inhibits the proliferation, migration and invasion of retinoblastoma cells by targeting ECM1 BIOSCIENCE REP. 2020 Jun 26;40(6):BSR20200392.;doi: 10.1042/BSR20200392

245. Yong-Mei Liu,Shuo Cong,Zhuo Cheng,Ya-Xin Hu,Yu Lei,Li-Li Zhu,Xue-Ke Zhao,Mao Mu,Bao-Fang Zhang,Lin-da Fan,Lei Yu,Ming-Liang Cheng.Platycodin D alleviates liver fibrosis and activation of hepatic stellate cells by regulating JNK/c-JUN signal pathway Eur J Pharmacol. 2020 Jun 5;876:172946.;doi: 10.1016/j.ejphar.2020.172946

246. Jia Xu,Hong-Bo Tan,Ya-Jun Zhang,Dian-Yong Tang,Fenghuang Zhan,Hong-Yu Li,Zhong-Zhu Chen,Zhi-Gang Xu.Catalyst-Free One-Pot Synthesis of Densely Substituted Pyrazole-Pyrazines as Anti-Colorectal Cancer Agents SCI REP-UK. 2020 Jun 9;10(1):9281.;doi: 10.1038/s41598-020-66137-z.

247. Xidan Zhu,Jia Feng,Wenguang Fu,Xiaojia Shu,Xue Wan,Jinbo Liu.Effects of cisplatin on the proliferation, invasion and apoptosis of breast cancer cells following β-catenin silencing Int J Mol Med. 2020 Jun;45(6):1838-1850.;doi: 10.3892/ijmm.2020.4543

248. Ping Wang,Jiming Xu,Weijing You,Yongfeng Hou,Shuiliang Wang,Yujie Ma,Jianming Tan,Zengli Zhang,Wentao Hu,Bingyan Li.Knockdown of CYP24A1 Aggravates 1 α,25(OH) 2 D 3-Inhibited Migration and Invasion of Mouse Ovarian Epithelial Cells by Suppressing EMT Front Oncol. 2020 Jul 29;10:1258.;doi: 10.3389/fonc.2020.01258

249. Jingyi Wang,Minghui Li,Xu Han,Hui Wang,Xinyang Wang,Ge Ma,Tiansong Xia,Shui Wang.MiR-1976 knockdown promotes epithelial-mesenchymal transition and cancer stem cell properties inducing triple-negative breast cancer metastasis Cell Death Dis. 2020 Jul 3;11(7):500.;doi: 10.1038/s41419-020-2711-x.

250. Dyah Ari Nugrahaningrum,Olivia Marcelina,Caiping Liu,Shourong Wu,Vivi Kasim.Dapagliflozin Promotes Neovascularization by Improving Paracrine Function of Skeletal Muscle Cells in Diabetic Hindlimb Ischemia Mice Through PHD2/HIF-1α Axis Front Pharmacol. 2020 Aug 10;11:1104.;doi: 10.3389/fphar.2020.01104

251. Chen Zhao,Ye Wang,Zhonglan Su,Wenyuan Pu,Mengyuan Niu,Shiyu Song,Lulu Wei,Yibing Ding,Lizhi Xu,Man Tian,Hongwei Wang.Respiratory exposure to PM2.5 soluble extract disrupts mucosal barrier function and promotes the development of experimental asthma Sci Total Environ. 2020 Aug 15;730:139145.;doi: 10.1016/j.scitotenv.2020.139145

252. Zhong Wang,Zhiyu Li,Qi Wu,Chenyuan Li,Juanjuan Li,Yimin Zhang,Changhua Wang,Si Sun,Shengrong Sun.DNER promotes epithelial-mesenchymal transition and prevents chemosensitivity through the Wnt/β-catenin pathway in breast cancer Cell Death Dis. 2020 Aug 18;11(8):642.;doi: 10.1038/s41419-020-02903-1

253. Boshi Wang,Tiantian Jing,Weilin Jin,Jinnan Chen,Chengsi Wu,Mingrong Wang,Yizhen Liu.KIAA1522 potentiates TNFα-NFκB signaling to antagonize platinum-based chemotherapy in lung adenocarcinoma J EXP CLIN CANC RES. 2020 Aug 27;39(1):170.;doi: 10.1186/s13046-020-01684-x

254. Su-Wei Fu,Yan Zhang,Shen Li,Zhi-Yan Shi,Juan Zhao,Qing-Li He.LncRNA TTN-AS1 promotes the progression of oral squamous cell carcinoma via miR-411-3p/NFAT5 axis Cancer Cell Int. 2020 Aug 28;20:415.;doi: 10.1186/s12935-020-01378-6

255. Jinghua Chen,Meiqin Zhu,Liqiu Zou,Junxian Xia,Jiacheng Huang,Quantong Deng,Ruilian Xu.Long non-coding RNA LINC-PINT attenuates paclitaxel resistance in triple-negative breast cancer cells via targeting the RNA-binding protein NONO ACTA BIOCH BIOPH SIN. 2020 Aug 5;52(8):801-809.;doi: 10.1093/abbs/gmaa072

256. Hao Shen,Zaikai Lin,Haiyan Shi,Lingling Wu,Baojin Ma,Hong Li,Baobing Yin,Jun Tang,Hongjin Yu,Xiaoxing Yin.MiR-221/222 promote migration and invasion, and inhibit autophagy and apoptosis by modulating ATG10 in aggressive papillary thyroid carcinoma 3 Biotech. 2020 Aug;10(8):339.;doi: 10.1007/s13205-020-02326-x

257. Yijun Shi,Na Fang,Yadong Li,Zizhang Guo,Wei Jiang,Yaozhou He,Zijian Ma,Yijiang Chen.Circular RNA LPAR3 sponges microRNA-198 to facilitate esophageal cancer migration, invasion, and metastasis Cancer Sci. 2020 Aug;111(8):2824-2836.;doi: 10.1111/cas.14511

258. Kun Shan,Rong-Mei Zhou,Jun Xiang,Ya-Nan Sun,Chang Liu,Meng-Wei Lv,Jian-Jiang Xu.FTO regulates ocular angiogenesis via m 6 A-YTHDF2-dependent mechanism Exp Eye Res. 2020 Aug;197:108107.;doi: 10.1016/j.exer.2020.108107

259. Yufeng Liu,Zhimin Zhang,Qing Li,Liang Zhang,Yi Cheng,Zhaoyang Zhong.Mitochondrial APE1 promotes cisplatin resistance by downregulating ROS in osteosarcoma Oncol Rep. 2020 Aug;44(2):499-508.;doi: 10.3892/or.2020.7633

260. Wei Chen,Denghui Gao,Long Xie,Anling Wang,Hui Zhao,Chaowan Guo,Yunqi Sun,Yanfeng Nie,An Hong,Sheng Xiong.SCF-FBXO24 regulates cell proliferation by mediating ubiquitination and degradation of PRMT6 BIOCHEM BIOPH RES CO. 2020 Sep 10;530(1):75-81.;doi: 10.1016/j.bbrc.2020.06.007

261. Maoyu Wang,Yongliang Yang,Jing Yang,Juanjuan Yang,Shumei Han.circ_KIAA1429 accelerates hepatocellular carcinoma advancement through the mechanism of m 6 A-YTHDF3-Zeb1 Life Sci. 2020 Sep 15;257:118082.;doi: 10.1016/j.lfs.2020.118082

262. Xiaojing Zhang,Yuzhong Chen,Kangwei Wang,Jingwei Tang,Yansong Chen,Gongsheng Jin,Xianfu Liu.The knockdown of the sepiapterin reductase gene suppresses the proliferation of breast cancer by inducing ROS-mediated apoptosis INT J CLIN EXP PATHO. 2020 Sep 1;13(9):2228-2239

263. Chen Wang,Didi Zhao,Kexin Wang,Lei Gao,Yue He,Hanhan Wu,Liang Ruan,Wenjun Chen,Daoming Zhang,Tao Xia,Shiqing Qian,Zhining Liu,Yi Yang,Wanshui Yang,Anla Hu,Qihong Zhao.All-Trans Retinoic Acid Rescues the Tumor Suppressive Role of RAR-β by Inhibiting LncHOXA10 Expression in Gastric Tumorigenesis Nutr Cancer. 2020 Sep 22;1-13.;doi: 10.1080/01635581.2020.1823006

264. Yunyan Zhou,Hongwei An,Gang Wu.MicroRNA-6071 Suppresses Glioblastoma Progression Through the Inhibition of PI3K/AKT/mTOR Pathway by Binding to ULBP2 ONCOTARGETS THER. 2020 Sep 23;13:9429-9441.;doi: 10.2147/OTT.S265791

265. Jie Lian,Chao Liu,Xin Guan,Bojun Wang,Yuanfei Yao,Dan Su,Yue Ma,Lin Fang,Yanqiao Zhang.Ubiquitin specific peptidase 5 enhances STAT3 signaling and promotes migration and invasion in Pancreatic Cancer J Cancer. 2020 Sep 30;11(23):6802-6811.;doi: 10.7150/jca.48536

266. Rui Yu,Jie Yao,Yu Ren.A novel circRNA, circNUP98, a potential biomarker, acted as an oncogene via the miR-567/ PRDX3 axis in renal cell carcinoma J Cell Mol Med. 2020 Sep;24(17):10177-10188.;doi: 10.1111/jcmm.15629

267. Yang Fu,Chiyuan Piao,Zhe Zhang,Yuyan Zhu,Shanshan Sun,Jianbin Bi,Chuize Kong,Min Ju.Decreased expression and hypomethylation of HDAC9 lead to poor prognosis and inhibit immune cell infiltration in clear cell renal cell carcinoma UROL ONCOL-SEMIN ORI. 2020 Sep;38(9):740.e1-740.e9.;doi: 10.1016/j.urolonc.2020.03.006

268. Chenlei Zheng,Tan Zhang,Ding Li,Chongchu Huang,Hengjie Tang,Xiao-Feng Ni,Bicheng Chen.Upregulation of CENPM facilitates tumor metastasis via the mTOR/p70S6K signaling pathway in pancreatic cancer Oncol Rep. 2020 Sep;44(3):1003-1012.;doi: 10.3892/or.2020.7673

269. Bin Zhan,Linjin Huang,Yachun Chen,Wen Ye,Jingkun Li,Jianhui Chen,Sheng Yang,Wei Jiang.miR-196a-mediated downregulation of p27 kip1 protein promotes prostate cancer proliferation and relates to biochemical recurrence after radical prostatectomy Prostate. 2020 Sep;80(12):1024-1037.;doi: 10.1002/pros.24036

270. Long Li,Jun-Dong Liu,Guo-Dong Gao,Kai Zhang,Yu-Wei Song,Hong-Bo Li.Puerarin 6″-O-xyloside suppressed HCC via regulating proliferation, stemness, and apoptosis with inhibited PI3K/AKT/mTOR CANCER MED-US. 2020 Sep;9(17):6399-6410.;doi: 10.1002/cam4.3285

271. Wenkai Ni,Shengli Lin,Saiyan Bian,Wenjie Zheng,Lishuai Qu,Yihui Fan,Cuihua Lu,Mingbing Xiao,Pinghong Zhou.USP7 mediates pathological hepatic de novo lipogenesis through promoting stabilization and transcription of ZNF638 Cell Death Dis. 2020 Oct 10;11(10):843.;doi: 10.1038/s41419-020-03075-8

272. Kiren Mustafa,Hassan Mohamed,Aabid Manzoor Shah,Shaoxuan Yu,Muhammad Akhlaq,Haifang Xiao,Shaoqi Li,Tahira Naz,Shaista Nosheen,Xueyuan Bai,Yuanda Song.In Vitro Anticancer Potential of Berberis lycium Royle Extracts against Human Hepatocarcinoma (HepG2) Cells Biomed Res Int. 2020 Oct 14;2020:8256809.;doi: 10.1155/2020/8256809

273. Qingling Kong,Qing Fan,Xianbin Ma,Jian Li,Rong Ma.CircRNA circUGGT2 Contributes to Hepatocellular Carcinoma Development via Regulation of the miR-526b-5p/RAB1A Axis Cancer Manag Res. 2020 Oct 15;12:10229-10241.;doi: 10.2147/CMAR.S263985

274. Dianbao Zhang,Chunhe Li,Luting Zhang,Bo Li,Yu Wang,Rui Wang,Zaixing Chen,Liang Xu,Tao Liu.Cannabisin D from Sinomenium Acutum Inhibits Proliferation and Migration of Glioblastoma Cells through MAPKs Signaling Nutr Cancer. 2020 Oct 19;1-11.;doi: 10.1080/01635581.2020.1836240

275. Xiaohong Guo,Chao Song,Lei Fang,Min Li,Longtao Yue,Qing Sun.FLRT2 functions as Tumor Suppressor gene inactivated by promoter methylation in Colorectal Cancer J Cancer. 2020 Oct 23;11(24):7329-7338.;doi: 10.7150/jca.47558

276. Zi-Zhang Guo,Zi-Jian Ma,Yao-Zhou He,Wei Jiang,Yang Xia,Chun-Feng Pan,Ke Wei,Yi-Jun Shi,Liang Chen,Yi-Jiang Chen.miR-550a-5p Functions as a Tumor Promoter by Targeting LIMD1 in Lung Adenocarcinoma Front Oncol. 2020 Oct 28;10:570733.;doi: 10.3389/fonc.2020.570733

277. Shengming Yi,Guiyuan Li,Biaofeng Sun.Overexpression of LINC00852 promotes prostate cancer cell proliferation and metastasis ASIA-PAC J CLIN ONCO. 2020 Oct 30.;doi: 10.1111/ajco.13418

278. Liang Zhang,Zhuang Tong,Zhe Sun,Guolian Zhu,Erdong Shen,Yanfeng Huang.MiR-25-3p targets PTEN to regulate the migration, invasion, and apoptosis of esophageal cancer cells via the PI3K/AKT pathway BIOSCIENCE REP. 2020 Oct 30;40(10):BSR20201901.;doi: 10.1042/BSR20201901

279. 279. Liu-Jun He,Dong-Lin Yang,He-Ying Chen,Jiu-Hong Huang,Ya-Jun Zhang,Hong-Xia Qin,Juan-Li Wang,Dian-Yong Tang,Zhong-Zhu Chen.A Novel Imidazopyridine Derivative Exhibits Anticancer Activity in Breast Cancer by Inhibiting Wnt/β-catenin Signaling ONCOTARGETS THER. 2020 Oct 9;13:10111-10121.;doi: 10.2147/OTT.S266752

280. Na Li.CircTBL1XR1/miR-424 axis regulates Smad7 to promote the proliferation and metastasis of colorectal cancer J Gastrointest Oncol. 2020 Oct;11(5):918-931.;doi: 10.21037/jgo-20-395.

281. Wenzhuo Ma,Fanfan Liang,Heqin Zhan,Xixi Jiang,Chenying Gao,Xin Zhang,Kaina Zhang,Qiang Sun,Hao Hu,Zhenghang Zhao.Activated FMS-like tyrosine kinase 3 ameliorates angiotensin II-induced cardiac remodelling ACTA PHYSIOL. 2020 Oct;230(2):e13519.;doi: 10.1111/apha.13519

282. Y Chen,X-H Zhao,D-D Zhang,Y Zhao.MiR-513a-3p inhibits EMT mediated by HOXB7 and promotes sensitivity to cisplatin in ovarian cancer cells EUR REV MED PHARMACO. 2020 Oct;24(20):10391-10402.;doi: 10.26355/eurrev_202010_23389

283. Chenhui Bao,Lin Guo.MicroRNA-148a-3p inhibits cancer progression and is a novel screening biomarker for gastric cancer J Clin Lab Anal. 2020 Oct;34(10):e23454.;doi: 10.1002/jcla.23454

284. Na-Sha Song,Zhi-Dong Pei,Gui Fu.MiR-1224-5p acts as a tumor suppressor via inhibiting the malignancy of rectal cancer through targeting SLC29A3 IUBMB Life. 2020 Oct;72(10):2204-2213.;doi: 10.1002/iub.2352

285. Zhi-Teng Chen,Hai-Feng Zhang,Meng Wang,Shao-Hua Wang,Zhu-Zhi Wen,Qing-Yuan Gao,Mao-Xiong Wu,Wen-Hao Liu,Yong Xie,Jing-Ting Mai,Ying Yang,Jing-Feng Wang,Yang-Xin Chen.Long non-coding RNA Linc00092 inhibits cardiac fibroblast activation by altering glycolysis in an ERK-dependent manner Cell Signal. 2020 Oct;74:109708.;doi: 10.1016/j.cellsig.2020.109708

286. Xiaolin Wang,Jialun Shi,Zhigao Niu,Jianwu Wang,Wenping Zhang.MiR-216a-3p regulates the proliferation, apoptosis, migration, and invasion of lung cancer cells via targeting COPB2 BIOSCI BIOTECH BIOCH. 2020 Oct;84(10):2014-2027.;doi: 10.1080/09168451.2020.1783197

287. Shuiping Liu,Qiujie Li,Guohua Li,Qin Zhang,Lvjia Zhuo,Xuemeng Han,Mingming Zhang,Xiaying Chen,Ting Pan,Lili Yan,Ting Jin,Jianjun Wang,Qun Lv,Xinbing Sui,Tian Xie.The mechanism of m 6 A methyltransferase METTL3-mediated autophagy in reversing gefitinib resistance in NSCLC cells by β-elemene Cell Death Dis. 2020 Nov 11;11(11):969.;doi: 10.1038/s41419-020-03148-8

288. Chenlin Pei,Xuejun Gong,Yi Zhang.LncRNA MALAT-1 promotes growth and metastasis of epithelial ovarian cancer via sponging microrna-22 Am J Transl Res. 2020 Nov 15;12(11):6977-6987

289. Yong Xia,Shuzhi Liu,Changlin Li,Zhiying Ai,Wenzhi Shen,Wenqi Ren,Xiaolong Yang.Discovery of a novel ferroptosis inducer-talaroconvolutin A-killing colorectal cancer

cells in vitro and in vivo Cell Death Dis. 2020 Nov 17;11(11):988.;doi: 10.1038/s41419-020-03194-2

290. Shuiqing Wu,Ran Xu,Xuan Zhu,Haiqing He,Jinhua Zhang,Qi Zeng,Yinhuai Wang,Xiaokun Zhao.The long noncoding RNA LINC01140/miR-140-5p/FGF9 axis modulates bladder cancer cell aggressiveness and macrophage M2 polarization AGING-US. 2020 Nov 21;12(24):25845-25864.;doi: 10.18632/aging.202147
291. Hongzao Ni,Daofei Ji,Zhixiong Huang,Jing Li.SMAGP knockdown inhibits the malignant phenotypes of glioblastoma cells by inactivating the PI3K/Akt pathway Arch Biochem Biophys. 2020 Nov 30;695:108628.;doi: 10.1016/j.abb.2020.108628
292. Haiming Ma,Tao Liu,Yanhua Xu,Xinying Wang,Jin Wang,Xiaokang Liu.MiR-519d and miR-328-3p Combinatorially Suppress Breast Cancer Progression ONCOTARGETS THER. 2020 Dec 18;13:12987-12997.;doi: 10.2147/OTT.S281962
293. Zerui Wu,Yongzhi Zheng,Wanqun Xie,Qun Li,Yong Zhang,Bohan Ren,Lin Cai,Yijun Cheng,Hao Tang,Zhipeng Su,Zhe Bao Wu.The long noncoding RNA-H19/miRNA-93a/ATG7 axis regulates the sensitivity of pituitary adenomas to dopamine agonists Mol Cell Endocrinol. 2020 Dec 1;518:111033.;doi: 10.1016/j.mce.2020.111033
294. Bing Wang,Xiaowei Wang,Xing Tong,Yingang Zhang.Schisandrin B Inhibits Cell Viability and Migration, and Induces Cell Apoptosis by circ_0009112/miR-708-5p Axis Through PI3K/AKT Pathway in Osteosarcoma Front Genet. 2020 Dec 22;11:588670.;doi: 10.3389/fgene.2020.588670
295. Nuo Li,Zhifeng Zhao,Pengliang Liu,Yan Zheng,Shuang Cai,Yin Sun,Baoming Wang.Upregulation of deubiquitinase USP7 by transcription factor FOXO6 promotes EC progression via targeting the JMJD3/CLU axis Mol Ther Oncolytics. 2020 Dec 25;20:583-595.;doi: 10.1016/j.omto.2020.12.008.
296. Min Xue,Weijun Hong,Jun Jiang,Fang Zhao,Xiwen Gao.Circular RNA circ-LDLRAD3 serves as an oncogene to promote non-small cell lung cancer progression by upregulating SLC1A5 through sponging miR-137 RNA Biol. 2020 Dec;17(12):1811-1822.;doi: 10.1080/15476286.2020.1789819
297. Shanshan Li,Hui Yang,Min Zhao,Linli Gong,Yahong Wang,Zhiyong Lv,Yuhang Quan,Zhonghui Wang.Demethylation of HACE1 gene promoter by propofol promotes autophagy of human A549 cells Oncol Lett. 2020 Dec;20(6):280.;doi: 10.3892/ol.2020.12143
298. Lijiang Yu,Lingli Huo,Xiaolin Shao,Jizhi Zhao.lncRNA SNHG5 promotes cell proliferation, migration and invasion in oral squamous cell carcinoma by sponging miR-655-3p/FZD4 axis Oncol Lett. 2020 Dec;20(6):310.;doi: 10.3892/ol.2020.12173
299. Nan Pang,Zhixiao Lin,Xiaolin Wang,Lirong Xu,Xiaoli Xu,Rong Huang,Xingxing Li,Xueyong Li,Jinqing Li.Endothelial cell-derived CCL15 mediates the transmigration of fibrocytes through the CCL15-CCR1 axis in vitro Mol Med Rep. 2020 Dec;22(6):5339-5347.;doi: 10.3892/mmr.2020.11610
300. J Yang,H Wang,W Xu,Z Chen,Z-Y Yan,L-J Zhang.Inhibition of miR-133b indicates poor prognosis and promotes progression of OSCC via SOX4 EUR REV MED PHARMACO. 2020 Dec;24(24):12717-12726.;doi: 10.26355/eurrev_202012_24170. (IF 3.024)
301. Cheng Chen,Hongjin Wu,Deshengyue Kong,Yu Xu,Zunyue Zhang,Fengrong Chen,Lei Zou,Ziwei Li,Jin Shui,Huayou Luo,Shi-He Liu,Juehua Yu,Kunhua Wang,F Charles Brunicardi.Transcriptome sequencing analysis reveals unique and shared antitumor effects of three statins in pancreatic cancer Oncol Rep. 2020 Dec;44(6):2569-2580.;doi: 10.3892/or.2020.7810
302. Wangao Zhang,Pengju Chen,Huimin Zong,Yikun Ding,Ruhu Yan.MiR-143-3p targets ATG2B to inhibit autophagy and promote endothelial progenitor cells tube formation in deep vein thrombosis Tissue Cell. 2020 Dec;67:101453.;doi: 10.1016/j.tice.2020.101453
303. Xiaomin Su,Qianjing Zhang,Jianmei Yue,Yachen Wang,Yuan Zhang,Rongcun Yang.TRIM59 suppresses NO production by promoting the binding of PIAS1 and STAT1 in macrophages Int Immunopharmacol. 2020 Dec;89(Pt A):107030.;doi: 10.1016/j.intimp.2020.107030
304. Yingxin Wang,Xia Liu,Liwei Wang,Zhenduo Zhang,Zhong Li,Ming Li.Circ_PGPEP1 Serves as a Sponge of miR-1297 to Promote Gastric Cancer Progression via Regulating E2F3 DIGEST DIS SCI. 2021 Jan 1.;doi: 10.1007/s10620-020-06783-5
305. Na Li,Rui Hou,Tian Yang,Caixia Liu,Jun Wei.miR-193a-3p Mediates Placenta Accreta Spectrum Development by Targeting EFNB2 via Epithelial-Mesenchymal Transition Pathway Under Decidua Defect Conditions Front Mol Biosci. 2021 Jan 13;7:613802.;doi: 10.3389/fmolb.2020.613802
306. Li-Li Zhu,Zheng Wu,Rong-Kun Li,Xin Xing,Yong-Sheng Jiang,Jun Li,Ya-Hui Wang,Li-Peng Hu,Xu Wang,Wei-Ting Qin,Yong-Wei Sun,Zhi-Gang Zhang,Qin Yang,Shu-Heng Jiang.Deciphering the genomic and lncRNA landscapes of aerobic glycolysis identifies potential therapeutic targets in pancreatic cancer Int J Biol Sci. 2021 Jan 1;17(1):107-118.;doi: 10.7150/ijbs.49243
307. Jinglin Li,Canghai Guan,Zengtao Hu,Lang Liu,Zhilei Su,Pengcheng Kang,Xingming Jiang,Yunfu Cui.Yin Yang 1-induced LINC00667 up-regulates pyruvate dehydrogenase kinase 1 to promote proliferation, migration and invasion of cholangiocarcinoma cells by sponging miR-200c-3p Hum Cell. 2021 Jan;34(1):187-200.;doi: 10.1007/s13577-020-00448-1
308. Xuehan Yi,Wei Chen,Chen Li,Xiaoqiang Chen,Qin Lin,Shuchun Lin,Desheng Wang.Circular RNA circ_0004507 contributes to laryngeal cancer progression and cisplatin resistance by sponging miR-873 to upregulate multidrug resistance 1 and multidrug resistance protein 1 Head Neck. 2021 Mar;43(3):928-941.;doi: 10.1002/hed.26549.
309. Xuehan Bi,Xiao Lv,Dajiang Liu,Hongtao Guo,Guang Yao,Lijuan Wang,Xiaolei Liang,Yongxiu Yang.METTL3-mediated maturation of miR-126-5p promotes ovarian cancer progression via PTEN-mediated PI3K/Akt/mTOR pathway Cancer Gene Ther. 2021 Apr;28(3-4):335-349.;doi: 10.1038/s41417-020-00222-3
310. Ping Yu,Rui Wu,Zunchun Zhou,Xin Zhang,Ruoshu Wang,Xueting Wang,Sen Lin,Jihong Wang,Li Lv.rAj-Tspin, a novel recombinant peptide from Apostichopus japonicus, suppresses the proliferation, migration, and invasion of BEL-7402 cells via a mechanism associated with the ITGB1-FAK-AKT pathway INVEST NEW DRUG. 2021 Apr;39(2):377-385.;doi: 10.1007/s10637-020-01008-y
311. Yi He,Qimei Zhang,Huan Chen,Qingxi Guo,Liming Zhang,Zhuo Zhang,Yingchuan Li.Astragaloside IV enhanced carboplatin sensitivity in prostate cancer by suppressing AKT/NF-κB signaling pathway Biochem Cell Biol. 2021 Apr;99(2):214-222.;doi: 10.1139/bcb-2020-0026

Version 2021.09.01
