# Supplementary material for: Combination decoction of Astragalus mongholicus and Salvia miltiorrhiza mitigates pressure-overload cardiac dysfunction by inhibiting multiple ferroptosis pathways
Source: Front Pharmacol. 2024 Dec 16;15:1447546. doi: 10.3389/fphar.2024.1447546 (PMC11683366; doi:10.3389/fphar.2024.1447546)
Supplement: Supplementary file 4 [file DataSheet1.ZIP › C2006 Mitochondrial Membrane Potential Assay Kit (JC-1).pdf]

碧云天网站 微信公众号
碧云天生物技术/Beyotime Biotechnology
订货热线：400-168-3301或800-8283301
订货e-mail：order@beyotime.com
技术咨询：info@beyotime.com
网址：http://www.beyotime.com

线粒体膜电位检测试剂盒(JC-1)

产品编号	产品名称	包装
C2006	线粒体膜电位检测试剂盒(JC-1)	>100次

产品简介：

- 线粒体膜电位检测试剂盒(JC-1) (Mitochondrial membrane potential assay kit with JC-1)是一种以JC-1为荧光探针，快速灵敏地检测细胞、组织或纯化的线粒体膜电位变化的试剂盒，可以用于早期的细胞凋亡检测。
- JC-1是一种广泛用于检测线粒体膜电位(mitochondrial membrane potential) ΔΨm的理想荧光探针。可以检测细胞、组织或纯化的线粒体膜电位。在线粒体膜电位较高时，JC-1聚集在线粒体的基质(matrix)中，形成聚合物(J-aggregates)，可以产生红色荧光；在线粒体膜电位较低时，JC-1不能聚集在线粒体的基质中，此时JC-1为单体(monomer)，可以产生绿色荧光。这样就可以非常方便地通过荧光颜色的转变来检测线粒体膜电位的变化。常用红绿荧光的相对比例来衡量线粒体去极化的比例。
- 线粒体膜电位的下降是细胞凋亡早期的一个标志性事件。通过JC-1从红色荧光到绿色荧光的转变可以很容易地检测到细胞膜电位的下降，同时也可以用JC-1从红色荧光到绿色荧光的转变作为细胞凋亡早期的一个检测指标。JC-1检测线粒体膜电位的效果参考图1。

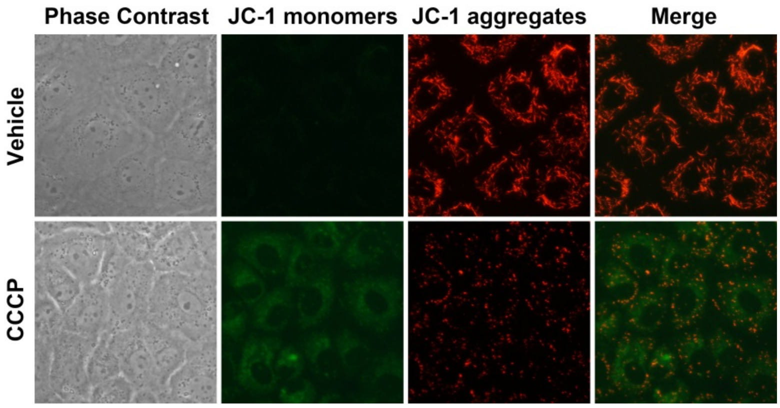

图1. 本试剂盒检测NRK-52E细胞(大鼠肾小管上皮细胞)线粒体膜电位的效果图。正常的NRK-52E细胞线粒体中JC-1以聚合物形式存在，呈明亮的红色荧光，细胞中的绿色荧光非常弱；使用CCCP处理使线粒体膜电位下降后，JC-1便不能以聚合物形式存在线粒体基质中，此时线粒体内红色荧光强度显著降低，而细胞浆中的绿色荧光显著增强。

- JC-1单体的最大激发波长为514nm，最大发射波长为529nm；JC-1聚合物(J-aggregates)的最大激发波长为585nm，最大发射波长为590nm。实际观察时，使用常规的观察红色荧光和绿色荧光的设置即可。JC-1单体和聚合物的激发光和发射光光谱参考图2。

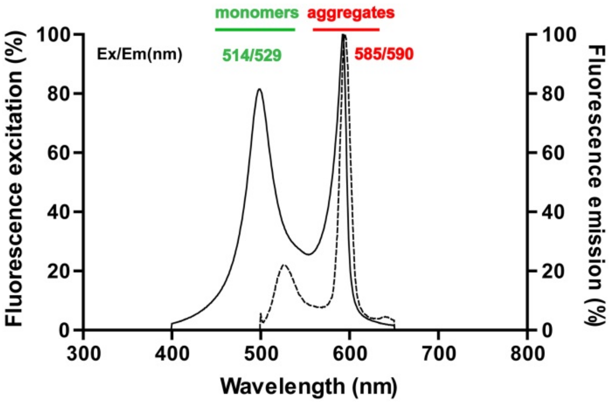

图2. JC-1单体和聚合物的激发光和发射光光谱。

- 本试剂盒提供了CCCP作为诱导线粒体膜电位下降的阳性对照。
- 对于六孔板中的样品，本试剂盒共可以检测100个样品；对于12孔中的样品，本试剂盒共可以检测200个样品。

包装清单：

产品编号	产品名称	包装
C2006-1	JC-1(200X)	100µl/管，共5管
C2006-2	超纯水	90ml
C2006-3	JC-1染色缓冲液(5X)	80ml
C2006-4	CCCP(10mM)	20µl
—	说明书	1份

保存条件：

-20ºC保存。JC-1(200X)需避光保存，并尽量避免反复冻融。超纯水和JC-1染色缓冲液(5X)也可4ºC保存。

注意事项：

- JC-1(200X)在4ºC、冰浴等较低温度情况下会凝固而粘在离心管管底、管壁或管盖内，可以20-25ºC水浴温育片刻至全部融解后使用。
- 必须先把JC-1(200X)用试剂盒提供的超纯水充分溶解混匀后，才可以加入JC-1染色缓冲液(5X)。不可先配制JC-1染色缓冲液(1X)再加入JC-1(200X)，这样JC-1会很难充分溶解，会严重影响后续的检测。
- 装载完JC-1后用JC-1染色缓冲液(1X)洗涤时，使JC-1染色缓冲液(1X)保持4ºC左右，此时的洗涤效果较好。
- JC-1探针装载完并洗涤后尽量在30分钟内完成后续检测。在检测前需冰浴保存。
- 请勿把JC-1染色缓冲液(5X)全部配制成JC-1染色缓冲液(1X)，本试剂盒使用过程中需直接使用JC-1染色缓冲液(5X)。
- 如果发现JC-1染色缓冲液(5X)中有沉淀，必须全部溶解后才能使用，为促进溶解可以在37ºC加热。
- CCCP为线粒体电子传递链抑制剂，对人体有害，操作时请小心，并注意有效防护以避免直接接触人体或吸入体内。
- 本产品仅限于专业人员的科学研究用，不得用于临床诊断或治疗，不得用于食品或药品，不得存放于普通住宅内。
- 为了您的安全和健康，请穿实验服并戴一次性手套操作。

使用说明：

1. **JC-1染色工作液的配制：**
   六孔板每孔所需JC-1染色工作液的量为1ml，其它培养器皿的JC-1染色工作液的用量以此类推；对于细胞悬液每50-100万细胞需0.5ml JC-1染色工作液。取适量JC-1(200X)，按照每50µl JC-1(200X)加入8ml超纯水的比例稀释JC-1。剧烈Vortex充分溶解并混匀JC-1。然后再加入2ml JC-1染色缓冲液(5X)，混匀后即为JC-1染色工作液。
2. **阳性对照的设置：**
   把试剂盒中提供的CCCP(10mM)推荐按照1:1000的比例加入到细胞培养液中，稀释至10µM，处理细胞20分钟。随后按照下述方法装载JC-1，进行线粒体膜电位的检测。对于大多数细胞，通常10µM CCCP处理20分钟后线粒体的膜电位会完全丧失，JC-1染色后观察应呈绿色荧光；而正常的细胞经JC-1染色后应显示红色荧光。对于特定的细胞，CCCP的作用浓度和作用时间可能有所不同，需自行参考相关文献资料确定。
3. **对于悬浮细胞：**
   a. 取10-60万细胞，重悬于0.5ml细胞培养液中，细胞培养液中可以含血清和酚红。
   b. 加入0.5ml JC-1染色工作液，颠倒数次混匀。细胞培养箱中37ºC孵育20分钟。
   c. 在孵育期间，按照每1ml JC-1染色缓冲液(5X)加入4ml蒸馏水的比例，配制适量的JC-1染色缓冲液(1X)，并放置于冰浴。
   d. 37ºC孵育结束后， 600g 4ºC离心3-4分钟，沉淀细胞。弃上清，注意尽量不要吸除细胞。
   e. 用JC-1染色缓冲液(1X)洗涤2次：加入1ml JC-1染色缓冲液(1X)重悬细胞，600g 4ºC离心3-4分钟，沉淀细胞，弃上清。再加入1ml JC-1染色缓冲液(1X)重悬细胞，600g 4ºC离心3-4分钟，沉淀细胞，弃上清。
   f. 再用适量JC-1染色缓冲液(1X)重悬后，用荧光显微镜或激光共聚焦显微镜观察，也可以用荧光分光光度计检测或流式细胞仪分析。
4. **对于贴壁细胞：**
   注意：对于贴壁细胞，如果希望采用荧光分光光度计或流式细胞仪检测，可以先收集细胞，重悬后参考悬浮细胞的检测方法。
   a. 对于六孔板的一个孔，吸除培养液，根据具体实验如有必要可以用PBS或其它适当溶液洗涤细胞一次，加入1ml细胞培养液。细胞培养液中可以含有血清和酚红。
   b. 加入1ml JC-1染色工作液，充分混匀。细胞培养箱中37ºC孵育20分钟。
   c. 在孵育期间，按照每1ml JC-1染色缓冲液(5X)加入4ml蒸馏水的比例，配制适量的JC-1染色缓冲液(1X)，并放置于冰浴。
   d. 37ºC孵育结束后， 吸除上清，用JC-1染色缓冲液(1X)洗涤2次。
   e. 加入2ml细胞培养液，培养液中可以含有血清和酚红。
   f. 荧光显微镜或激光共聚焦显微镜下观察。
5. **对于纯化的线粒体：**
   a. 把配制好的JC-1染色工作液再用JC-1染色缓冲液(1X)稀释5倍。
   b. 0.9ml 5倍稀释的JC-1染色工作液中加入0.1ml总蛋白量为10-100µg纯化的线粒体。
   c. 用荧光分光光度计或荧光酶标仪检测：混匀后直接用荧光分光光度计进行时间扫描(time scan)，激发波长为485nm，发射波长为590nm。如果使用荧光酶标仪，激发波长不能设置为485nm时，可以在475-520nm范围内设置激发波长。另外，也可以参考下面步骤6中的波长设置进行荧光检测。

d. 用荧光显微镜或激光共聚焦显微镜观察：方法同下面的步骤6。

6. 荧光观测和结果分析：

a. 检测JC-1单体时可以把激发光设置为490nm，发射光设置为530nm；检测JC-1聚合物时，可以把激发光设置为525nm，发射光设置为590nm。**注意：**此处测定荧光时不必把激发光和发射光设置在最大激发波长和最大发射波长。如使用荧光显微镜观察，检测JC-1单体时可以参考观察其它绿色荧光时的设置，如观察GFP或FITC时的设置；检测JC-1聚合物时可以参考观察其它红色荧光，如碘化丙啶或Cy3时的设置。出现绿色荧光说明线粒体膜电位下降，并且该细胞很可能处于细胞凋亡早期。出现红色荧光说明线粒体膜电位比较正常，细胞的状态也比较正常。

相关产品：

产品编号	产品名称	包装
C1002	DAPI	5mg/ml×0.2ml
C1005/C1006	DAPI 染色液	10ml/50ml
C1011	Hoechst 33258	10mg
C1017/C1018	Hoechst 33258 染色液	10ml/50ml
C1022	Hoechst 33342	10mg
C1025/C1026	Hoechst 33342 染色液	10ml/50ml
C1027/C1028	Hoechst 33342 活细胞染色液(100X)	0.1ml/0.5ml
C1031	CFDA SE (细胞增殖示踪荧光探针)	5mg
C1033	Actin-Tracker Green (微丝绿色荧光探针)	0.2ml
C1036	DiI (细胞膜红色荧光探针)	10mg
C1038	DiO (细胞膜绿色荧光探针)	10mg
C1041	ER-Tracker Red (内质网红色荧光探针)	20μl
C1043	Golgi-Tracker Red (高尔基体红色荧光探针)	1mg
C1046	Lyso-Tracker Red (溶酶体红色荧光探针)	50μl
C1048	Mito-Tracker Green (线粒体绿色荧光探针)	50μg
C1050	Tubulin-Tracker Red (微管红色荧光探针)	40μl
C2005	JC-1	1mg
C2006	线粒体膜电位检测试剂盒(JC-1)	>100 次
C2007	Rhodamine 123	5mg
S0019	DAF-FM DA (NO 荧光探针)	>100 次
S0063	Dihydroethidium (超氧化物阴离子荧光探针)	5mg
S1006	BCECF AM (pH 荧光探针, 5mM)	50 微升
S1052	Fura-2 AM (钙离子荧光探针, 2mM)	50 微升
S1056	Fluo-3 AM (钙离子荧光探针, 5mM)	20 微升
S1060	Fluo-4 AM (钙离子荧光探针, 2mM)	25 微升
S1082	MQAE (氯离子荧光探针)	20mg

使用本产品的文献：

1. Wang Z, Tang X, Li Y, Leu C, Guo L, Zheng X, Zhu D. 20-Hydroxyeicosatetraenoic acid inhibits the apoptotic responses in pulmonary artery smooth muscle cells. Eur J Pharmacol. 2008 Jun 24;588(1):9-17.
2. Xin H, Liu XH, Zhu YZ. Herba leonurine attenuates doxorubicin-induced apoptosis in H9c2 cardiac muscle cells. Eur J Pharmacol. 2009 Jun 10;612(1-3):75-9.
3. Chen K, Zhang Q, Wang J, Liu F, Mi M, Xu H, Chen F, Zeng K. Taurine protects transformed rat retinal ganglion cells from hypoxia-induced apoptosis by preventing mitochondrial dysfunction. Brain Res. 2009 Jul 7;1279:131-8.
4. Shen L, Liu Q, Ni J, Hong G. A proteomic investigation into the human cervical cancer cell line HeLa treated with dicitratoytterbium (III) complex. Chem Biol Interact. 2009 Oct 30;181(3):455-62.
5. Su X, Zheng X, Ni J. Lanthanum citrate induces anoikis of Hela cells. Cancer Lett. 2009 Nov 28;285(2):200-9.
6. Zhu XJ, Shi Y, Peng J, Guo CS, Shan NN, Qin P, Ji XB, Hou M. The effects of BAFF and BAFF-R-Fc fusion protein in immune thrombocytopenia. Blood. 2009 Dec 17;114(26):5362-7.
7. Wu F, Wang J, Wang Y, Kwok T, Kong S, Wong C. Estrogen-related receptor (ERR) inverse agonist XCT-790 induces cell death in chemotherapeutic resistant cancer cells. Chemico-Biological Interactions. 2009 Oct 7;181(2):236-42.
8. Shen LM, Liu Q , Ni JZ. Comparative proteomics analysis of lanthanum citrate complex-induced apoptosis in HeLa cells. Sci China Ser B-Chem. 2009 Nov;52(11):1814-20.
9. Ma J, Zhang L, Li S, Liu S, Ma C, Li W, Falck JR, Manthati VL, Reddy DS, Medhora M, Jacobs ER, Zhu D. 8,9-Epoxyeicosatrienoic acid analog protects pulmonary artery smooth muscle cells from apoptosis via ROCK pathway. Exp Cell Res. 2010;316(14):2340-53.
10. Ma H, Quan F, Chen D, Zhang B, Zhang Y. Alterations in mitochondrial function and spermatozoal motility in goat spermatozoa following incubation with a human lysozyme plasmid. Anim Reprod Sci. 2010;121(1-2):106-14.
11. Wu J, Sun J, Xue Y. Involvement of JNK and P53 activation in G2/M cell cycle arrest and apoptosis induced by titaniumdioxide nanoparticles in neuron cells. Toxicol Lett. 2010 Dec 15;199(3):269-76.
12. Wei H, Li Z, Hu S, Chen X, Cong X. Apoptosis of mesenchymal stem cells induced by hydrogen peroxide concerns both endoplasmicreticulum stress and mitochondrial death pathway through regulation of caspases, p38 and JNK. J Cell Biochem. 2010 Nov 1;111(4):967-78.
13. Zhang CM, Zeng XQ, Zhang R, Ji CB, Tong ML, Chi X, Li XL, Dai JZ, Zhang M, Cui Y, Guo XR. Effects of NYGGF4 knockdown on insulin sensitivity and mitochondrial function in 3T3-L1adipocytes. J Bioenerg Biomembr. 2010 Oct;42(5):433-9.
14. Shen L, Lan Z, Sun X, Shi L, Liu Q, Ni J. Proteomic analysis of lanthanum citrate-induced apoptosis in human cervical carcinoma SiHacells. Biometals. 2010 Dec;23(6):1179-89.
15. Peng X, Wang K, Hu C, Zhu Y, Wang T, Yang J, Tong J, Li S, Zhu Y. The mitochondrial gene orfH79 plays a critical role in impairing both male gametophytedevelopment and root growth in CMS-Honglian rice. BMC Plant Biol. 2010 Jun 24;10:125.
16. Zhang M, Sun C, Shan X, Yang X, Li-Ling J, Deng Y. Inhibition of pancreatic cancer cell growth by cucurbitacin B through modulation of signaltransducer and activator of transcription 3 signaling. Pancreas. 2010 Aug;39(6):923-9.

17. Zhu MT, Wang Y, Feng WY, Wang B, Wang M, Ouyang H, Chai ZF. Oxidative stress and apoptosis induced by iron oxide nanoparticles in cultured human umbilicalendothelial cells. J Nanosci Nanotechnol. 2010 Dec;10(12):8584-90.

18. He J, Yang D, Wang C, Liu W, Liao J, Xu T, Bai C, Chen J, Lin K, Huang C, Dong Q. Chronic zebrafish low dose decabrominated diphenyl ether (BDE-209) exposure affected parentalgonad development and locomotion in F1 offspring. Ecotoxicology. 2011 Nov;20(8):1813-22.

19. Shu D, Qing Y, Tong Q, He Y, Xing Z, Zhao Y, Li Y, Wei Y, Huang W, Wu X. Deltonin isolated from Dioscorea zingiberensis inhibits cancer cell growth through inducingmitochondrial apoptosis and suppressing Akt and mitogen activated protein kinase signals. Biol Pharm Bull. 2011;34(8):1231-9.

20. Li W, Nie S, Chen Y, Wang Y, Li C, Xie M. Enhancement of cyclophosphamide-induced antitumor effect by a novel polysaccharide fromGanoderma atrum in sarcoma 180-bearing mice. J Agric Food Chem. 2011 Apr 27;59(8):3707-16.

21. Xu HN, Huang WD, Cai Y, Ding M, Gu JF, Wei N, Sun LY, Cao X, Li HG, Zhang KJ, Liu XR, Liu XY. HCCS1-armed, quadruple-regulated oncolytic adenovirus specific for liver cancer as a cancertargeting gene-viro-therapy strategy. Mol Cancer. 2011 Nov 1;10:133.

22. Gao Y, Su Y, Qu L, Xu S, Meng L, Cai SQ, Shou C. Mitochondrial apoptosis contributes to the anti-cancer effect of Smilax glabra Roxb. Toxicol Lett. 2011 Nov 30;207(2):112-20.

23. Han M, Diao YY, Jiang HL, Ying XY, Chen DW, Liang WQ, Gao JQ. Molecular mechanism study of chemosensitization of doxorubicin-resistant human myelogenousleukemia cells induced by a composite polymer micelle. Int J Pharm. 2011 Nov 28;420(2):404-11.

24. Li WJ, Chen Y, Nie SP, Xie MY, He M, Zhang SS, Zhu KX. Ganoderma atrum polysaccharide induces anti-tumor activity via the mitochondrial apoptoticpathway related to activation of host immune response. J Cell Biochem. 2011 Mar; 112(3):860-71.

25. Fu L, Liu Q, Shen L, Wang Y. Proteomic study on sodium selenite-induced apoptosis of human cervical cancer HeLa cells. J Trace Elem Med Biol. 2011 Jul;25(3):130-7.

26. Rao J, Xu DR, Zheng FM, Long ZJ, Huang SS, Wu X, Zhou WH, Huang RW, Liu Q. Curcumin reduces expression of Bcl-2, leading to apoptosis in daunorubicin-insensitive CD34+acute myeloid leukemia cell lines and primary sorted CD34+ acute myeloid leukemia cells. J Transl Med. 2011 May 19;9:71.

27. Zhang Q, Zou P, Zhan H, Zhang M, Zhang L, Ge RS, Huang Y. Dihydrolipoamide dehydrogenase and cAMP are associated with cadmium-mediated Leydig celldamage. Toxicol Lett. 2011 Aug 28;205(2):183-9.

28. Xu L, Deng Y, Feng L, Li D, Chen X, Ma C, Liu X, Yin J, Yang M, Teng F, Wu W, Guan S, Jiang B, Guo D. Cardio-protection of salvianolic acid B through inhibition of apoptosis network. PLoS One. 2011;6(9):e24036.

29. Wei A, Zhou D, Xiong C, Cai Y, Ruan J. A novel non-aromatic B-ring flavonoid: isolation, structure elucidation and its induction of apoptosis in human colon HT-29 tumor cell via the reactive oxygen species-mitochondrial dysfunction and MAPK activation. Food Chem Toxicol. 2011 Sep;49(9):2445-52.

30. Meng F, Liu R, Gao M, Wang Y, Yu X, Xuan Z, Sun J, Yang F, Wu C, Du G. Pinocembrin attenuates blood-brain barrier injury induced by global cerebral ischemia-reperfusionin rats. Brain Res. 2011 May 19;1391:93-101.

31. Nie Y, Han BM, Liu XB, Yang JJ, Wang F, Cong XF, Chen X. Identification of MicroRNAs involved in hypoxia- and serum deprivation-induced apoptosis inmesenchymal stem cells. Int J Biol Sci. 2011;7(6):762-8.

32. Gao M, Wang J, Wang W, Liu J, Wong CW. Phosphatidylinositol 3-kinase affects mitochondrial function in part through inducing peroxisomeproliferator-activated receptor γ coactivator-1β expression. Br J Pharmacol. 2011 Feb;162(4):1000-8.

33. Xu S, Zhao Y, Yu L, Shen X, Ding F, Fu G. Rosiglitazone attenuates endothelial progenitor cell apoptosis induced by TNF-α via ERK/MAPKand NF-κB signal pathways. J Pharmacol Sci. 2011;117(4):265-74.

34. Bao Y, Wang X, Li W, Huo D, Shen X, Han Y, Tan J, Zeng Q, Sun C. 20-Hydroxyeicosatetraenoic acid induces apoptosis in neonatal rat cardiomyocytes throughmitochondrial-dependent pathways. J Cardiovasc Pharmacol. 2011 Mar;57(3):294-301.

35. Chen G, Zhang X, Zhao M, Wang Y, Cheng X, Wang D, Xu Y, Du Z, Yu X. Celastrol targets mitochondrial respiratory chain complex I to induce reactive oxygen species-dependent cytotoxicity in tumor cells. BMC Cancer. 2011 May 14;11:170.

36. Zhang Y, Guo J, Zeng L, Zhang J, Hui Y, Liu J, Qing X, Sun X, Guo G. Tert-butyl-2(4,5-dihydrogen-4,4,5,5-tetramethyl-3-O-1H-imidazole-3-cationic-1-oxyl-2-pyrrolidine-1-carboxylic ester displays novel cytotoxicity through reactive oxygen species-mediated oxidative damage in MCF-7 and MDA-MB-231 cells. Chem Biol Interact. 2011 Jul 15;192(3):287-97.

37. Yang XX, Chen J, Zhou Q, Guo XY, Xiao P, Wu J, Xu JT. Troglitazone induced apoptosis of human pterygium fibroblasts through a mitochondrial-dependent pathway. Int J Ophthalmol. 2011;4(2):143-6.

38. Liu J, Xu X, Feng X, Zhang B, Wang J. Adenovirus-mediated delivery of bFGF small interfering RNA reduces STAT3 phosphorylation andinduces the depolarization of mitochondria and apoptosis in glioma cells U251. J Exp Clin Cancer Res. 2011 Sep 9;30:80.

39. Liu Q, Li Y, Hu L, Wang D. Lycium barbarum Polysaccharides Attenuate Cisplatin-Induced Hair Cell Loss in Rat CochlearOrganotypic Cultures. Int J Mol Sci. 2011;12(12):8982-92.

40. Cui M, Zhang Y, Liu S, Xie W, Ji M, Lou H, Li X. 1-oxoeudesm-11(13)-ene-12,8α-lactone-induced apoptosis via ROS generation and mitochondriaactivation in MCF-7 cells. Arch Pharm Res. 2011 Aug;34(8):1323-9.

41. Zhang YH, Zhao CQ, Jiang LS, Dai LY. Cyclic stretch-induced apoptosis in rat annulus fibrosus cells is mediated in part by endoplasmicreticulum stress through nitric oxide production. Eur Spine J. 2011 Aug;20(8):1233-43.

42. Chen Y, Xie X. Tacrolimus attenuates myocardium damage to the total hepatic ischemia-reperfusion viaregulation of the mitochondrial function. J Surg Res. 2012 Jan;172(1):e47-54.

43. Yang X, Chen L, Liu Y, Yang Y, Chen T, Zheng W, Liu J, He QY. Ruthenium methylimidazole complexes induced apoptosis in lung cancer A549 cells throughintrinsic mitochondrial pathway. Biochimie. 2012 Feb;94(2):345-53.

44. Nie X, Song S, Zhang L, Qiu Z, Shi S, Liu Y, Yao L, Zhu D. 15-Hydroxyeicosatetraenoic acid (15-HETE) protects pulmonary artery smooth muscle cells fromapoptosis via inducible nitric oxide synthase (iNOS) pathway. Prostaglandins Other Lipid Mediat. 2012 Jan;97(1-2):50-9.

45. Li C, Li C, Zhu X, Wang C, Liu Z, Li W, Lu C, Zhou X. The expression and putative role of brain-derived neurotrophic factor and its receptor in bovinesperm. Theriogenology. 2012 Feb;77(3):636-43.

46. Hua S, Lu C, Song Y, Li R, Liu X, Quan F, Wang Y, Liu J, Su F, Zhang Y. High levels of mitochondrial heteroplasmy modify the development of ovine-bovine interspeciesnuclear transferred embryos. Reprod Fertil Dev. 2012;24(3):501-9.

47. Li XX, He GR, Mu X, Xu B, Tian S, Yu X, Meng FR, Xuan ZH, Du GH. Protective effects of baicalein against rotenone-induced neurotoxicity in PC12 cells and isolatedrat brain mitochondria. Eur J Pharmacol. 2012 Jan 15;674(2-3):227-33.

48. Zhuang XJ, Huang Y, Duan YP, Zhang M, Lu YQ, Lu KH. Translocation of active mitochondria during buffalo (Bubalus bubalis) oocytes in vitro maturation fertilization and preimplantation embryo development. Reprod Domest Anim. 2012 Jun;47(3):443-8.

49. Gu L, Liang X, Wang L, Yan Y, Ni Z, Dai H, Gao J, Mou S, Wang Q, Chen X, Wang L, Qian J. Functional metabotropic glutamate receptors 1 and 5 are expressed in murine podocytes. Kidney Int. 2012 Mar;81(5):458-68.

50. Ma J, Zhang L, Han W, Shen T, Ma C, Liu Y, Nie X, Liu M, Ran Y, Zhu D. Activation of JNK/c-Jun is required for the proliferation, survival, and angiogenesis induced by EETin pulmonary artery endothelial cells. J Lipid Res. 2012 Jun;53(6):1093-105.

51. Wang SW, Wang YJ, Su YJ, Zhou WW, Yang SG, Zhang R, Zhao M, Li YN, Zhang ZP, Zhan DW, Liu RT. Rutin inhibits β-amyloid aggregation and cytotoxicity, attenuates oxidative stress and decreasesthe production of nitric oxide and proinflammatory cytokines. Neurotoxicology. 2012 Jun;33(3):482-90.

52. Zhu Y, Li YH, Hu RH, Zheng YN,Feng N, Chen F, Wang J, Li HY. Origin of hydrogen peroxide during the cowpea-Xanthomonas interaction. AUSTRALASIAN PLANT PATHOLOGY. 2012;41(1):1-11.

53. Ou XH, Li S, Wang ZB, Li M, Quan S, Xing F, Guo L, Chao SB, Chen Z, Liang XW, Hou Y, Schatten H, Sun QY. Maternal insulin resistance causes oxidative stress and mitochondrial dysfunction in mouseoocytes. Hum Reprod. 2012 Jul;27(7):2130-45.

54. Quan X, Wang Y, Ma X, Liang Y, Tian W, Ma Q, Jiang H, Zhao Y. α-Mangostin induces apoptosis and suppresses differentiation of 3T3-L1 cells via inhibiting fattyacid synthase. PLoS One. 2012;7(3):e33376.

55. Zhang X, Xie P, Zhang X, Zhou W, Zhao S, Zhao Y, Cai Y. Toxic effects of microcystin-LR on the HepG2 cell line under hypoxic and normoxic conditions. J Appl Toxicol. 2013 Oct;33(10):1180-6.

56. Tao Q, Fan X, Li T, Tang Y, Yang D, Le W. Gender segregation in gene expression and vulnerability to oxidative stress induced injury inventral mesencephalic cultures of dopamine neurons. J Neurosci Res. 2012 Jan;90(1):167-78.

57. Ge H, Tollner TL, Hu Z, Dai M, Li X, Guan H, Shan D, Zhang X, Lv J, Huang C, Dong Q. The importance of mitochondrial metabolic activity and mitochondrial DNA replication duringoocyte maturation in vitro on oocyte quality and subsequent embryo developmental competence. Mol Reprod Dev. 2012 Jun;79(6):392-401.

58. Yu GR, Qin WW, Li JP, Hua W, Meng YL, Chen R, Yan B, Wang L, Zhang X, Jia LT, Zhao J, Zhang R, Yang AG. HIV-TAT-fused FHIT protein functions as a potential pro-apoptotic molecule in hepatocellularcarcinoma cells. Biosci Rep. 2012 Jun;32(3):271-9.

59. Shi ZY, Li YQ, Kang YH, Hu GQ, Huang-fu CS, Deng JB, Liu B. Piperonal ciprofloxacin hydrazone induces growth arrest and apoptosis of humanhepatocarcinoma SMMC-7721 cells. Acta Pharmacol Sin. 2012 Feb;33(2):271-8.

60. Su Y, Wang X, Xu W, Xue L, He C, Yang D, An R. Arsenic trioxide increases the sensitivity of 786-0 renal carcinoma cells to radiotherapy. Cancer Invest. 2012 Feb; 30(2):114-8.

61. Zhang HA, Gao M, Zhang L, Zhao Y, Shi LL, Chen BN, Wang YH, Wang SB, Du GH. Salvianolic acid A protects human SH-SY5Y neuroblastoma cells against H_2O_2-induced injury by increasing stress tolerance ability. Biochem Biophys Res Commun. 2012 May 11;421(3):479-83.

62. Shen X, Liu L, Yin F, Ma H, Zou S. Effect of dehydroepiandrosterone on cell growth and mitochondrial function in TM-3 cells. Gen Comp Endocrinol. 2012 May 15;177(1):177-86.

63. Sang H, Zhang L, Li J. Anti-benzopyrene-7,8-diol-9,10-epoxide induces apoptosis via mitochondrial pathway in humanbronchiolar epithelium cells independent of the mitochondria permeability transition pore. Food Chem Toxicol. 2012 Jul;50(7):2417-23.

64. Wang CQ, Zhang J, Sun YF, Wang D, Li CJ, Li WH, Lu C, Liu Z, Wu H, Hou XF, Chen C, Zhou X. Detection of Neurotrophin-4 (NT-4) in Ejaculated Bull Spermatozoa and its Effect on SpermatozoaMitochondrial Activity. Reprod Domest Anim. 2013 Feb;48(1):131-6.

65. Zhang J, Song W, Guo J, Zhang J, Sun Z, Ding F, Gao M. Toxic effect of different ZnO particles on mouse alveolar macrophages. J Hazard Mater. 2012 Jun 15;219-220:148-55.

66. Zhao P, Han T, Guo JJ, Zhu SL, Wang J, Ao F, Jing MZ, She YL, Wu ZH, Ye LB. HCV NS4B induces apoptosis through the mitochondrial death pathway. Virus Res. 2012 Oct;169(1):1-7.

67. Li Z, Chen J, Lei T, Zhang H. Tamoxifen induces apoptosis of mouse microglia cell line BV-2 cells via both mitochondrial anddeath receptor pathways. J Huazhong Univ Sci Technolog Med Sci. 2012 Apr;32(2):221-6.

68. Khan M, Ding C, Rasul A, Yi F, Li T, Gao H, Gao R, Zhong L, Zhang K, Fang X, Ma T. Isoalantolactone induces reactive oxygen species mediated apoptosis in pancreatic carcinomaPANC-1 cells. Int J Biol Sci. 2012;8(4):533-47.

69. Ding F, Shao ZW, Yang SH, Wu Q, Gao F, Xiong LM. Role of mitochondrial pathway in compression-induced apoptosis of nucleus pulposus cells. Apoptosis. 2012 Jun;17(6):579-90.

70. Lin CJ, Huang HC, Liu WJ, Jiang ZF. Biomedical Imaging and Visualization Model of Mitochondrial Dysfunction and Oxidative Stress in Alzheimer Disease. Applied Mechanics and Materials. 2012;1179-1182.

71. Hua S, Zhang H, Song Y, Li R, Liu J, Wang Y, Quan F, Zhang Y. High expression of Mfn1 promotes early development of bovine SCNT embryos: improvement of mitochondrial membrane potential and oxidative metabolism. Mitochondrion. 2012 Mar;12(2):320-7.

72. Cheng L, Wang C, Liu H, Wang F, Zheng L, Zhao J, Chu E, Lin X. A novel polypeptide extracted from Ciona savignyi induces apoptosis through a mitochondrial-mediated pathwayin human colorectal carcinoma cells. Clin Colorectal Cancer. 2012 Sep;11(3):207-14.

73. Lu YS, Huang BT, Huang YX. Reactive oxygen species formation and apoptosis in human peripheral blood mononuclear cell induced by 900MHz mobile phone radiation. Oxid Med Cell Longev. 2012;2012:740280.

74. Li S, Wu C, Zhu L, Gao J, Fang J, Li D, Fu M, Liang R, Wang L, Cheng M, Yang H. By improving regional cortical blood flow, attenuating mitochondrial dysfunction and sequential apoptosis galangin acts as a potential neuroprotective agent after acute ischemic stroke. Molecules. 2012 Nov 9;17(11):13403-23.

75. Li XY, Jing CQ, Lei WL, Li J, Wang JJ. Apoptosis caused by imidazolium-based ionic liquids in PC12 cells. Ecotoxicol Environ Saf. 2012 Sep;83:102-7.

76. Liu Q, Peng YB, Qi LW, Cheng XL, Xu XJ, Liu LL, Liu EH, Li P. The Cytotoxicity Mechanism of 6-Shogaol-Treated HeLa Human Cervical Cancer Cells Revealed by Label-FreeShotgun Proteomics and Bioinformatics Analysis. Evid Based Complement Alternat Med. 2012;2012:278652.

77. Luo GX, Cai J, Lin JZ, Luo WS, Luo HS, Jiang YY, Zhang Y. Autophagy Inhibition Promotes Gambogic Acid-induced Suppression of Growth and Apoptosis in GlioblastomaCells. Asian Pac J Cancer Prev. 2012;13(12):6211-6.

78. Liu W, Zheng X, Qu Z, Zhang M, Zhou C, Ma L, Zhang Y. Effect of 935-MHz phone-simulating electromagnetic radiation on endometrial glandular cells during mouseembryo implantation. J Huazhong Univ Sci Technolog Med Sci. 2012 Oct;32(5):755-9.

79. Zhang L, Li K, Bing Ma L, Gong SB, Wang GY, Liu Y, Ji XY, Xu L, Liu SK, Chen P, Ouyang RY, Xiang XD. Effects and mechanism of arsenic trioxide on reversing the asthma pathologies including Th17-IL-17 axis in amouse model. Iran J Allergy Asthma Immunol. 2012 Jun;11(2):133-45.

80. Fu J, Dang Z, Deng Y, Lu G. Regulation of c-Myc and Bcl-2 induced apoptosis of human bronchial epithelial cells by zinc oxide nanoparticles. J Biomed Nanotechnol. 2012 Aug;8(4):669-75.

81. Zhou ZR, Zhong BS, Jia RX, Wan YJ, Zhang YL, Fan YX, Wang LZ, You JH, Wang ZY, Wang F. Production of myostatin-targeted goat by nuclear transfer from cultured adult somatic cells. Theriogenology. 2013 Jan 15;79(2):225-33.

82. Yue B, Zhao CR, Xu HM, Li YY, Cheng YN, Ke HN, Yuan Y, Wang RQ, Shi YQ, Lou HX, Qu XJ. Riccardin D-26, a synthesized macrocyclic bisbibenzyl compound, inhibits human oral squamous carcinoma cells KB and KB/VCR: In vitro and in vivo studies. Biochim Biophys Acta. 2013 Jan;1830(1):2194-203.

83. Zhang W, Lan Y, Huang Q, Hua Z. Galangin induces B16F10 melanoma cell apoptosis via mitochondrial pathway and sustained activation of p38MAPK. Cytotechnology. 2013 May;65(3):447-55.

84. Zhang Z, Liu Z, Ma L, Jiang S, Wang Y, Yu H, Yin Q, Cui J, Li Y. Reversal of multidrug resistance by mitochondrial targeted self-assembled nanocarrier based on stearylamine. Mol Pharm. 2013 Jun 3;10(6):2426-34.

85. Mo R, Sun Q, Li N, Zhang C. Intracellular delivery and antitumor effects of pH-sensitive liposomes based on zwitterionic oligopeptide lipids. Biomaterials. 2013 Apr;34(11):2773-86.

86. Kong D, Zhang F, Wei D, Zhu X, Zhang X, Chen L, Lu Y, Zheng S. Paeonol inhibits hepatic fibrogenesis via disrupting nuclear factor-κB pathway in activated stellate cells: In vivoand in vitro studies. J Gastroenterol Hepatol. 2013 Jul;28(7):1223-33.

87. Zhang F, Kong DS, Zhang ZL, Lei N, Zhu XJ, Zhang XP, Chen L, Lu Y, Zheng SZ. Tetramethylpyrazine induces G0/G1 cell cycle arrest and stimulates mitochondrial-mediated and caspase-dependent apoptosis through modulating ERK/p53 signaling in hepatic stellate cells in vitro. Apoptosis. 2013 Feb;18(2):135-49.

88. Chen G, Zhang P, Huang T, Yu W, Lin J, Li P, Chen K. Polysaccharides from Rhizopus nigricans mycelia induced apoptosis and G2/M arrest in BGC-823 cells. Carbohydrate Polymers. 2013 Sep;97(2):800–8.

89. Zhu K，Guo X, Guo X, Peng W,Zhou H. Protective effects of wheat germ protein isolate hydrolysates (WGPIH) against hydrogen peroxide-induced oxidative stress in PC12 cells. Food Research International. 2013 Aug;53(1): 297-303.

90. Sun B, Cai Y, Li Y, Li J, Liu K, Li Y, Yang Y. The nonstructural protein NP1 of human bocavirus 1 induces cell cycle arrest and apoptosis in Hela cells. Virology. 2013 May 25;440(1):75-83.

91. Li Z,Gao Q. Induction of apoptosis in HT-29 cells by quercetin through mitochondria-mediated apoptotic pathway. Animal Cells and Systems. 2013;17(3):147-153.

92. Ge YL, Weng XC, Tian T, Ding F, Huang R, Yuan LB, Wu J, Wang TL, Guo PG, Zhou X. A mitochondria-targeted zinc(II) phthalocyanine for photodynamic therapy. RSC Advances. 2013;31:12839-12846.

93. Liu Z,Yu Y, Yuan Z, Zhang F, Jing C. Dexamethasone-induced apoptosis in PC12 cellson Orphan Drugs Expert opinion on orpahn Drugs. 2013 May;32(2):34-38.

94. Huang YY, Liu H, Li Y, Pu LJ, Jiang CC, Xu JC, Jiang ZW. Down-regulation of RIP1 by 2-deoxy-D-glucose sensitizes breast cancer cells to TRAIL-induced apoptosis. Eur J Pharmacol. 2013 Apr 5;705(1-3):26-34.

95. Wang Y, Xuan Z, Tian S, He G, Du G. Myricitrin attenuates 6-hydroxydopamine-induced mitochondrial damage and apoptosis in PC12 cells via inhibition of mitochondrial oxidation. Journal of Functional Foods. 2013 Jan;5(1):337-345.

96. Wen YD, Wang H, Kho SH, Rinkiko S, Sheng X, Shen HM, Zhu YZ. Hydrogen sulfide protects HUVECs against hydrogen peroxide induced mitochondrial dysfunction and oxidativestress. PLoS One. 2013;8(2):e53147.

97. Yan H, Zhang S, He J, Yin Y, Wang X, Chen X, Cui F, Li Y, Nie Y, Tian W. Self-assembled monolayers with different chemical group substrates for the study of MCF-7 breast cancer cell linebehavior. Biomed Mater. 2013 Jun;8(3):035008.

98. Liu L, Gu L, Ma Q, Zhu D, Huang X. Resveratrol attenuates hydrogen peroxide-induced apoptosis in human umbilical vein endothelial cells. Eur Rev Med Pharmacol Sci. 2013 Jan;17(1):88-94.

99. Huang L, Wan J, Chen Y, Wang Z, Hui L, Li Y, Xu D, Zhou W. Inhibitory effects of p38 inhibitor against mitochondrial dysfunction in the early brain injury after subarachnoidhemorrhage in mice. Brain Res. 2013 Jun 23;1517:133-40.

100. Yang F, Sun X, Shen J, Yu LP, Liang JY, Zheng HQ, Wu ZD. A recombined protein (rSj16) derived from Schistosoma japonicum induces cell cycle arrest and apoptosis ofmurine myeloid leukemia cells. Parasitol Res. 2013 Mar;112(3):1261-72.

101. Zhang D, Gao X, Wang Q, Qin M, Liu K, Huang F, Liu B. Kakkalide ameliorates endothelial insulin resistance by suppressing reactive oxygen species-associatedinflammation. J Diabetes. 2013 Mar;5(1):13-24.

102. Liu Z, Dong X, Song L, Zhang H, Liu L, Zhu D, Song C, Leng X. Carboxylation of multiwalled carbon nanotube enhanced its biocompatibility with L02 cells through decreasedactivation of mitochondrial apoptotic pathway. J Biomed Mater Res A. 2014 Mar;102(3):665-73.

103. Li X, Tao H, Xie K, Ni Z, Yan Y, Wei K, Chuang PY, He JC, Gu L. cAMP signaling prevents podocyte apoptosis via activation of protein kinase A and mitochondrial fusion. PLoS One. 2014 Mar 18;9(3):e92003.

104. Sun J, Bai S, Bai W, Zou F, Zhang L, Li G, Hu Y, Li M, Yan R, Su Z, Huang Y. 1,3-Dichloro-2-propanol inhibits progesterone production through the expression of steroidogenic enzymes and cAMP concentration in Leydig cells. Food Chem. 2014 Jul 1;154:330-6.

105. Chang C, Wu G, Gao P, Yang L, Liu W, Zuo J. Upregulated Parkin expression protects mitochondrial homeostasis in DJ-1 konckdown cells and cells overexpressing the DJ-1 L166P mutation. Mol Cell Biochem. 2014 Feb;387(1-2):187-95.

106. Yang L, Di G, Qi X, Qu M, Wang Y, Duan H, Danielson P, Xie L, Zhou Q. Substance P promotes diabetic corneal epithelial wound healing through molecular mechanisms mediated via the neurokinin-1 receptor. Diabetes. 2014 Dec;63(12):4262-74.

107. Li D, Ye Y, Lin S, Deng L, Fan X, Zhang Y, Deng X, Li Y, Yan H, Ma Y. Evaluation of deoxynivalenol-induced toxic effects on DF-1 cells in vitro: cell-cycle arrest, oxidative stress, and apoptosis. Environ Toxicol Pharmacol. 2014 Jan;37(1):141-9.

108. He M, Dong C, Xie Y, Li J, Yuan D, Bai Y, Shao C. Reciprocal bystander effect between α-irradiated macrophage and hepatocyte is mediated by cAMP through a membrane signaling pathway. Mutat Res Fundam Mol Mech Mutagen. 2014 Mar 20;763-764C:1-9.

109. Tang C, Liang J, Qian J, Jin L, Du M, Li M, Li D. Opposing role of JNK-p38 kinase and ERK1/2 in hydrogen peroxide-induced oxidative damage of human trophoblast-like JEG-3 cells. Int J Clin Exp Pathol. 2014 Feb 15;7(3):959-68.

110. Chu JH, Zhao CR, Song ZY, Wang RQ, Qin YZ, Li WB, Qu XJ. 1082-39, an analogue of sorafenib, inhibited human cancer cell growth more potently than sorafenib. Biomed Pharmacother. 2014 Apr;68(3):335-41. .

111. Pan H, Cheng L, Yang H, Zou W, Cheng R, Hu T. Lysophosphatidic acid rescues human dental pulp cells from ischemia-induced apoptosis. J Endod. 2014 Feb;40(2):217-22.

112. Zheng L, Sun X, Zhu X, Lv F, Zhong Z, Zhang F, Guo W, Cao W, Yang L, Tian Y. Apoptosis of THP-1 derived macrophages induced by sonodynamic therapy using a new sonosensitizer hydroxyl acetylated curcumin. PLoS One. 2014 Mar 27; 9(3):e93133.

113. Cui SX, Zhang HL, Xu WF, Qu XJ. 13F-1, a novel 5-fluorouracil prodrug containing an Asn-Gly-Arg (NO2) COOCH3 tripeptide, inhibits human colonic carcinoma growth by targeting Aminopeptidase N (APN/CD13). Eur J Pharmacol. 2014 Jul 5;734:50-9.

114. Fang L, Bai C, Chen Y, Dai J, Xiang Y, Ji X, Huang C, Dong Q. Inhibition of ROS production through mitochondria-targeted antioxidant and mitochondrial uncoupling increases post-thaw sperm viability in yellow catfish. Cryobiology. 2014 Dec;69(3):386-93.

115. Wang C, Xia W, Jiang Q, Xu Y, Yu P. Differential effects of lipid fractions from silver carp brain on human cervical carcinoma cells in vitro. Food Funct. 2014 Sep;5(9):2194-201.

116. Fan S, Li X, Lin J, Chen S, Shan J, Qi G. Honokiol inhibits tumor necrosis factor-α-stimulated rat aortic smooth muscle cell proliferation via caspase- and mitochondrial-dependent apoptosis. Inflammation. 2014 Feb;37(1):17-26.

117. Guo P, Pi H, Xu S, Zhang L, Li Y, Li M, Cao Z, Tian L, Xie J, Li R, He M, Lu Y, Liu C, Duan W, Yu Z, Zhou Z. Melatonin Improves Mitochondrial Function by Promoting MT1/SIRT1/PGC-1 Alpha-Dependent Mitochondrial Biogenesis in Cadmium-Induced Hepatotoxicity In Vitro. Toxicol Sci. 2014 Nov;142(1):182-95.

118. Li D, Ma H, Ye Y, Ji C, Tang X, Ouyang D, Chen J, Li Y, Ma Y. Deoxynivalenol induces apoptosis in mouse thymic epithelial cells through mitochondria-mediated pathway. Environ Toxicol Pharmacol. 2014 Jul;38(1):163-71.

119. Liu L, Li T, Tan J, Fu J, Guo Q, Ji H, Zhang Y. NG as a novel nitric oxide donor induces apoptosis by increasing reactive oxygen species and inhibiting mitochondrial function in MGC803 cells. Int Immunopharmacol. 2014 Aug 16;23(1):27-36.

120. Zhang X, Yu Q, Jiang W, Bi Y, Zhang Y, Gong M, Wei X, Li T, Chen J. All-trans retinoic acid suppresses apoptosis in PC12 cells injured by oxygen and glucose deprivation via the retinoic acid receptor α signaling pathway. Mol Med Rep. 2014 Nov;10(5):2549-55.

121. Jiang L, Zhao MN, Liu TY, Wu XS, Weng H, Ding Q, Shu YJ, Bao RF, Li ML, Mu JS, Wu WG, Ding QC, Cao Y, Hu YP, Shen BY, Tan ZJ, Liu YB. Bufalin induces cell cycle arrest and apoptosis in gallbladder carcinoma cells. Tumour Biol. 2014 Nov;35(11):10931-41.

122. Sun ZB, Wang JW, Xiao H, Zhang QS, Kan WS, Mo FB, Hu S, Ye SN. Icariin may benefit the mesenchymal stem cells of patients with steroid-associated osteonecrosis by ABCB1-promoter demethylation: a preliminary study. Osteoporos Int. 2015 Jan;26(1):187-97.

123. Pan HY, Yang H, Shao MY, Xu J, Zhang P, Cheng R, Hu T. Sphingosine-1-phosphate mediates AKT/ERK maintenance of dental pulp homoeostasis. Int Endod J. 2015 May;48(5):460-8.

124. Yan X, Yang X, Hao X, Ren Q, Gao J, Wang Y, Chang N, Qiu Y, Song G.Sodium Fluoride Induces Apoptosis in H9c2 Cardiomyocytes by Altering Mitochondrial Membrane Potential and Intracellular ROS Level.Biol Trace Elem Res. 2015 Aug;166(2):210-5.

125. Li H, Jia Z, Li G, Zhao X, Sun P, Wang J, Fan Z, Lv G.Neuroprotective effects of exendin-4 in rat model of spinal cord injury via inhibiting mitochondrial apoptotic pathway.Int J Clin Exp Pathol. 2015 May 1;8(5):4837-43.

126. Lin P, Liu J, Ren M, Ji K, Li L, Zhang B, Gong Y, Yan C. Idebenone protects against oxidized low density lipoprotein induced mitochondrial dysfunction in vascular endothelial cells via GSK3β/β-catenin signalling pathways.Biochem Biophys Res Commun. 2015 Sep 25;465(3):548-55.

127. Ye X, Han Y, Zhang L, Liu W, Zuo J. MTERF4 regulates the mitochondrial dysfunction induced by MPP(+) in SH-SY5Y cells.Biochem Biophys Res Commun. 2015 Aug 14;464(1):214-20.

128. Zhang B, Peng X, Li G, Xu Y, Xia X, Wang Q. Oxidative stress is involved in Patulin induced apoptosis in HEK293 cells.Toxicon. 2015 Feb;94:1-7.

129. Wang W, Xie Q, Zhou X, Yao J, Zhu X, Huang P, Zhang L, Wei J, Xie H, Zhou L, Zheng S. Mitofusin-2 triggers mitochondria Ca2+ influx from the endoplasmic reticulum to induce apoptosis in hepatocellular carcinoma cells.Cancer Lett. 2015 Mar 1;358(1):47-58.

130. Xiong Y, Wu X, Rao L. Tetrastigma hemsleyanum (Sanyeqing) root tuber extracts induces apoptosis in human cervical carcinoma HeLa cells.J Ethnopharmacol. 2015 May 13;165:46-53.

131. Zhao L, Feng Y, Shi A, Zong Y, Wan M. Apoptosis Induced by Microbubble-Assisted Acoustic Cavitation in K562 Cells: The Predominant Role of the Cyclosporin A-Dependent Mitochondrial Permeability Transition Pore. Ultrasound Med Biol. 2015 Oct;41(10):2755-64.

132. Wang P, Peng X, Wei ZF, Wei FY, Wang W, Ma WD, Yao LP, Fu YJ, Zu YG. Geraniin exerts cytoprotective effect against cellular oxidative stress by upregulation of Nrf2-mediated antioxidant enzyme expression via PI3K/AKT and ERK1/2 pathway.Biochim Biophys Acta. 2015 Sep;1850(9):1751-61.

133. Yang J, Liu D, Dahms HU, Wang L. Cadmium inhibits the vitellogenesis of freshwater crab Sinopotamon henanense.Environ Toxicol Chem. 2015 Jul;34(7):1609-16.

134. Zong Y, Huang Y, Chen S, Zhu M, Chen Q, Feng S, Sun Y, Zhang Q, Tang C, Du J, Jin H.Downregulation of Endogenous Hydrogen Sulfide Pathway Is Involved in Mitochondrion-Related Endothelial Cell Apoptosis Induced by High Salt.Oxid Med Cell Longev. 2015;2015:754670.

135. Zhang J, Wu D, Xing Z, Liang S, Han H, Shi H, Zhang Y, Yang Y, Li Q. N-Isopropylacrylamide-modified polyethylenimine-mediated p53 gene delivery to prevent the proliferation of cancer cells.Colloids Surf B Biointerfaces. 2015 May 1;129:54-62.

136. Ding C, Wu Z, Huang L, Wang Y, Xue J, Chen S, Deng Z, Wang L, Song Z, Chen S. Mitofilin and CHCHD6 physically interact with Sam50 to sustain cristae structure.Sci Rep. 2015 Nov 4;5:16064.

137. Xin L, Ma X, Xiao Z, Yao H, Liu Z.
Coxsackievirus B3 induces autophagy in HeLa cells via the AMPK/MEK/ERK and Ras/Raf/MEK/ERK signaling pathways.
Infect Genet Evol. 2015 Dec;36:46-54.

138. Wu J, Zhu D, Zhang J, Li G, Liu Z, Sun J.
Lithium protects against methamphetamine-induced neurotoxicity in PC12 cells via Akt/GSK3 β /mTOR pathway.
Biochem Biophys Res Commun.2015 Sep 25;465(3):368-73.

139. Wang AT, Liang DS, Liu YJ, Qi XR.
Roles of ligand and TPGS of micelles in regulating internalization, penetration and accumulation against sensitive or resistant tumor and therapy for multidrug resistant tumors.
Biomaterials.2015 Jun;53:160-72.

140. Lu Z, Lu F, Zheng Y, Zeng Y, Zou C, Liu X.
Grape seed proanthocyanidin extract protects human umbilical vein endothelial cells from indoxyl sulfate-induced injury via ameliorating mitochondrial dysfunction.
Ren Fail.2015 Oct 29:1-9.

141. Yang J, Liu D, Dahms HU, Wang L.
Cadmium inhibits the vitellogenesis of freshwater crab Sinopotamon henanense.
Environ Toxicol Chem.2015 Jul;34(7):1609-16.

142. Li X, Song Y, Zhang P, Zhu H, Chen L, Xiao Y1, Xing Y.
Oleanolic acid inhibits cell survival and proliferation of prostate cancer cells in vitro and in vivo through the PI3K/Akt pathway.
Tumour Biol. 2016;37(6):7599-7613.

143. Peng X, Zhang YY, Wang J, Ji Q.
Ethylacetate extract from Tetrastigma hemsleyanum induces apoptosis via the mitochondrial caspase-dependent intrinsic pathway in HepG2 cells.
Tumour Biol. 2016;37(1): 865-76.

144. Qiu J, Liu X, Li X, Zhang X, Han P, Zhou H, Shao L, Hou Y, Min Y, Kong Z, Wang Y, Wei Y, Liu X, Ni H, Peng J, Hou M.
CD8(+) T cells induce platelet clearance in the liver via platelet desialylation in immunethrombocytopenia.
Sci Rep. 2016 Jun 20;6:27445.

145. Peng X, Gan J, Wang Q, Shi Z, Xia X.
3-Monochloro-1,2-propanediol (3-MCPD) induces apoptosis via mitochondrial oxidative phosphorylationsystem impairment and the caspase cascade pathway.
Toxicology. 2016 Nov 30;372:1-11.

146. Peng X, Zhang YY, Wang J, Ji Q.
Ethylacetate extract from Tetrastigma hemsleyanum induces apoptosis via the mitochondrial caspase-dependent intrinsic pathway in HepG2 cells.
Tumour Biol. 2016 Jan;37(1):865-76.

147. Yang H, Yang R, Liu H, Ren Z, Wang C, Li D, Ma X.
Knockdown of peroxisome proliferator-activated receptor gamma coactivator-1 alpha increasedapoptosis of human endometrial cancer HEC-1A cells.
Onco Targets Ther. 2016 Aug 24;9:5329-38.

148. Bai L, Guo Y, Du Y, Wang H, Zhao Z, Huang Y, Tang J.
47kDa isoform of Annexin A7 affecting the apoptosis of mouse hepatocarcinoma cells line.
Biomed Pharmacother. 2016 Oct;83:1127-1131.

149. Yu S, Zheng S, Leng J, Wang S, Zhao T, Liu J.
Inhibition of mitochondrial calcium uniporter protects neurocytes from ischemia/reperfusion injury viathe inhibition of excessive mitophagy.
Neurosci Lett. 2016 Aug 15;628:24-9.

150. Jiang K, Mei SQ, Wang TT, Pan JH, Chen YH, Cai J.
Vip3Aa induces apoptosis in cultured Spodoptera frugiperda (Sf9) cells.
Toxicon. 2016 Sep 15;120:49-56.

151. Li X, Song Y, Zhang P, Zhu H, Chen L, Xiao Y, Xing Y.
Oleanolic acid inhibits cell survival and proliferation of prostate cancer cells in vitro and in vivo through the PI3K/Akt pathway.
Tumour Biol.2016 Jun;37(6):7599-613.

152. Fu X, Gao X, Ge L, Cui X, Su C, Yang W, Sun X, Zhang W, Yao Z, Yang X, Yang J.
Malonate induces the assembly of cytoplasmic stress granules.
FEBS Lett. 2016 Jan;590(1):22-33.

153. Liu Y, Zhi D, Li M, Liu D, Wang X, Wu Z, Zhang Z, Fei D, Li Y, Zhu H, Xie Q, Yang H, Li H.
Shengmai Formula suppressed over-activated Ras/MAPK pathway in C. elegans by openingmitochondrial permeability transition pore via regulating cyclophilin D.
Sci Rep. 2016 Dec 16;6:38934.

154. Liu Y, Gao M, Ma MM, Tang YB, Zhou JG, Wang GL, Du YH, Guan YY.
Endophilin A2 protects H2O2-induced apoptosis by blockade of Bax translocation in rat basilar artery smooth muscle cells.
J Mol Cell Cardiol. 2016 Mar;92:122-33.

155. Liu Q, Sun Y, Lv Y, Le Z, Xin Y, Zhang P, Liu Y.
TERT alleviates irradiation-induced late rectal injury by reducing hypoxia-induced ROS levels through the activation of NF- κ B and autophagy.
Int J Mol Med. 2016 Sep;38(3):785-93.

156. Zhang E, Yin S, Song X, Fan L, Hu H.
Glycycoumarin inhibits hepatocyte lipoapoptosis through activation of autophagy and inhibition of ERstress/GSK-3-mediated mitochondrial pathway.

Sci Rep. 2016 Nov 30;6:38138.

157. Zhang X, Wang L, Wang R, Luo X, Li Y, Chen Z.
Protective effects of rice dreg protein hydrolysates against hydrogen peroxide-induced oxidative stressin HepG-2 cells.
Food Funct. 2016 Mar;7(3):1429-37.

158. Zhang F, Zhu X, Gong J, Sun Y, Chen D, Wang J, Wang Y, Guo M, Li W.
Lysosome-mitochondria-mediated apoptosis specifically evoked in cancer cells induced by goldnanorods.
Nanomedicine (Lond). 2016 Aug;11(15):1993-2006.

159. Zhang JY, Deng YN, Zhang M, Su H, Qu QM .
SIRT3 Acts as a Neuroprotective Agent in Rotenone-Induced Parkinson Cell Model.
Neurochem Res. 2016 Jul;41(7):1761-73.

160. Liu MP, Liao M, Dai C, Chen JF, Yang CJ, Liu M, Chen ZG, Yao MC.
Sanguisorba officinalis L synergistically enhanced 5-fluorouracil cytotoxicity in colorectal cancer cellsby promoting a reactive oxygen species-mediated, mitochondria-caspase-dependent apoptotic pathway.
Sci Rep.2016 Sep 27;6:34245.

161. Liu T, Yuan X, Jia T, Liu C, Ni Z, Qin Z, Yuan Y.
Polymeric prodrug of bufalin for increasing solubility and stability: Synthesis and anticancer study invitro and in vivo.
Int J Pharm. 2016 Jun 15;506(1-2):382-93.

162. Liu X, Zhang Y, Wang Y, Yan Y, Wang J, Gu J, Chun B, Liu Z.
Investigation of cadmium-induced apoptosis and the protective effect of N-acetylcysteine in BRL 3Acells.
Mol Med Rep. 2016 Jul;14(1):373-9.

163. Kang WL, Xu GS.
Atrasentan increased the expression of klotho by mediating miR-199b-5p and prevented renal tubularinjury in diabetic nephropathy.
Sci Rep. 2016 Jan 27;6:19979.

164. Dong K, Yan Y, Wang P, Shi X, Zhang L, Wang K, Xing J, Dong Y.
Biodegradable mixed MPEG-SS-2SA/TPGS micelles for triggered intracellular release of paclitaxel andreversing multidrug resistance.
Int J Nanomedicine. 2016 Oct 6;11:5109-5123.

165. Wang M, Li Y, HuangFu M, Xiao Y, Zhang T, Han M, Xu D, Li F, Ling D, Jin Y, Gao J.
Pluronic-attached polyamidoamine dendrimer conjugates overcome drug resistance in breast cancer.
Nanomedicine (Lond). 2016 Nov;11(22):2917-2934.

166. Lu Z, Lu F, Zheng Y, Zeng Y, Zou C, Liu X.
Grape seed proanthocyanidin extract protects human umbilical vein endothelial cells from indoxylsulfate-induced injury via ameliorating mitochondrial dysfunction.
Ren Fail. 2016;38(1):100-8.

167. Duan YT, Man RJ, Tang DJ, Yao YF, Tao XX, Yu C, Liang XY, Makawana JA, Zou MJ, Wang ZC, Zhu HL.
Design, Synthesis and Antitumor Activity of Novel link-bridge and B-Ring Modified Combretastatin A-4(CA-4) Analogues as Potent Antitubulin Agents.
Sci Rep. 2016 May 3;6:25387.

168. Luo SW, Wang WN, Sun ZM, Xie FX, Kong JR, Liu Y, Cheng CH.
Molecular cloning, characterization and expression analysis of (B-cell lymphoma-2 associated X protein)Bax in the orange-spotted grouper (Epinephelus coioides) after the Vibrio alginolyticus challenge.
Dev Comp Immunol. 2016 Jul;60:66-79.

169. He L, Xiao D, Feng J, Yao C, Tang L.
Induction of apoptosis of liver cancer cells by nanosecond pulsed electric fields (nsPEFs).
Med Oncol. 2017 Feb;34(2):24.

170. Wang B, Zhao XH.
Apigenin induces both intrinsic and extrinsic pathways of apoptosis in human colon carcinoma HCT-116cells.
Oncol Rep. 2017 Feb;37(2):1132-1140.

171. Zhang A, Sheng Y, Zou M.
Antiproliferative activity of Alisol B in MDA-MB-231 cells is mediated by apoptosis, dysregulation ofmitochondrial functions, cell cycle arrest and generation of reactive oxygen species.
Biomed Pharmacother. 2017 Mar;87:110-117.

172. Tong J, Mo QG, Ma BX, Ge LL, Zhou G, Wang YW.
The protective effects of Cichorium glandulosum seed and cynarin against cyclophosphamide and itsmetabolite acrolein-induced hepatotoxicity in vivo and in vitro.
Food Funct. 2017 Jan 25;8(1):209-219.

173. Xue F, Shi C, Chen Q, Hang W, Xia L, Wu Y, Tao SZ, Zhou J, Shi A, Chen J.
Melatonin Mediates Protective Effects against Kainic Acid-Induced Neuronal Death through Safeguarding ER Stress and Mitochondrial Disturbance.
Front Mol Neurosci. 2017 Feb 28;10:49.

174. Su S, Zhu X, Lin L, Chen X, Wang Y, Zi J, Dong Y, Xie Y, Zhu Y, Zhang J, Zhu J, Xu D, Xu N, Lou X, Liu S.
Lowering Endogenous Cathepsin D Abundance Results in Reactive Oxygen SpeciesAccumulation and Cell Senescence.
Mol Cell Proteomics. 2017 Jul;16(7):1217-1232.

175. Qie X, Wen D, Guo H, Xu G, Liu S, Shen Q, Liu Y, Zhang W, Cong B, Ma C.
Endoplasmic Reticulum Stress Mediates Methamphetamine-Induced Blood-Brain BarrierDamage.
Front Pharmacol. 2017 Sep 14;8:639.

176. Xu D, Chen L, Chen X, Wen Y, Yu C, Yao J, Wu H, Wang X, Xia Q, Kong X.
The triterpenoid CDDO-imidazolide ameliorates mouse liver ischemia-reperfusion injury through activating the Nrf2/HO-1 pathway enhanced autophagy.
Cell Death Dis. 2017 Aug 10;8(8):e2983.

177. Huang HL, Shi YP, He HJ, Wang YH, Chen T, Yang LW, Yang T, Chen J, Cao J, Yao WM, Liu G.
MiR-4673 Modulates Paclitaxel-Induced Oxidative Stress and Loss of Mitochondrial MembranePotential by Targeting 8-Oxoguanine-DNA Glycosylase-1.
Cell Physiol Biochem. 2017;42(3):889-900.

178. Tian F, Wu CL, Yu BL, Liu L, Hu JR.
Apolipoprotein O expression in mouse liver enhances hepatic lipid accumulation by impairingmitochondrial function.
Biochem Biophys Res Commun. 2017 Sep 9;491(1):8-14.

179. Hu CH, Zhuang XJ, Wei YM, Zhang M, Lu SS, Lu YQ, Yang XG, Lu KH.
Comparison of Mitochondrial Function in Boar and Bull Spermatozoa ThroughoutCryopreservation Based on JC-1 Staining.
Cryo Letters. 2017 Jan/Feb;38(1):75-79.

180. Zhang A, Sheng Y, Zou M.
Antiproliferative activity of Alisol B in MDA-MB-231 cells is mediated by apoptosis, dysregulation of mitochondrial functions, cell cycle arrest and generation of reactive oxygen species.
Biomed Pharmacother. 2017 Mar;87:110-117.

181. Zhong Y, Jin C, Gan J, Wang X, Shi Z, Xia X, Peng X.
Apigenin attenuates patulin-induced apoptosis in HEK293 cells by modulating ROS-mediatedmitochondrial dysfunction and caspase signal pathway.
Toxicon. 2017 Oct;137:106-113.

182. Feng Y, Wang Y, Jiang C, Fang Z, Zhang Z, Lin X, Sun L, Jiang W.
Nicotinamide induces mitochondrial-mediated apoptosis through oxidative stress in humancervical cancer HeLa cells.
Life Sci. 2017 Jul 15;181:62-69.

183. Wang T, Gao YY, Chen L, Nie ZW, Cheng W, Liu X, Schatten H4, Zhang X, Miao YL.
Melatonin prevents postovulatory oocyte aging and promotes subsequent embryonicdevelopment in the pig.
Aging (Albany NY). 2017 Jun 26;9(6):1552-1564.

184. You P, Wu H, Deng M, Peng J, Li F, Yang Y.
Brevilin A induces apoptosis and autophagy of colon adenocarcinoma cell CT26 viamitochondrial pathway and PI3K/AKT/mTOR inactivation.
Biomed Pharmacother. 2017 Dec 28;98:619-625.

185. Yang X, Tang S, Li D, Yu X, Wang F, Xiao X.
DIDS inhibits overexpression BAK1-induced mitochondrial apoptosis throughGSK3β/β-catenin signaling pathway.
J Cell Physiol. 2017 Dec 12.

186. Mehmood T, Maryam A, Tian X, Khan M, Ma T.
Santamarine Inhibits NF-κBand STAT3 Activation and Induces Apoptosis in HepG2 Liver CancerCells via Oxidative Stress.
J Cancer. 2017 Oct 17;8(18):3707-3717.

187. Wang P, Gao YM, Sun X, Guo N, Li J, Wang W, Yao LP, Fu YJ.
Hepatoprotective effect of 2'-O-galloylhyperin against oxidative stress-induced liver damage through induction of Nrf2/ARE-mediated antioxidant pathway.
Food Chem Toxicol. 2017 Apr;102:129-142.

188. Shan M, Qin J, Jin F, Han X, Guan H, Li X, Zhang J, Zhang H, Wang Y.
Autophagy suppresses isoprenaline-induced M2 macrophage polarization via the ROS/ERK and mTOR signaling pathway.
Free Radic Biol Med. 2017 Sep;110:432-443.

189. He Z, Pu L, Yuan C, Jia M, Wang J.
Nutrition deficiency promotes apoptosis of cartilage endplate stem cells ina caspase-independent manner partially through upregulating BNIP3.
Acta Biochim Biophys Sin (Shanghai). 2017 Jan;49(1):25-32.

190. Chen W, Liu Y, Zhang L, Gu X, Liu G, Shahid M, Gao J, Ali T, Han B.
Nocardia cyriacigeogica from Bovine Mastitis Induced In vitro Apoptosis of Bovine MammaryEpithelial Cells via Activation of Mitochondrial-Caspase Pathway.
Front Cell Infect Microbiol. 2017 May 18;7:194.

191. An J, Zhang Y, He J, Zang Z, Zhou Z, Pei X, Zheng X, Zhang W, Yang H, Li S.
Lactate dehydrogenase A promotes the invasion and proliferation of pituitary adenoma.
Sci Rep. 2017 Jul 5;7(1):4734.

192. Huo X, Wang C, Yu Z, Peng Y, Wang S, Feng S, Zhang S, Tian X, Sun C, Liu K, Deng S, Ma X.
Human transporters, PEPT1/2, facilitate melatonin transportation into mitochondria of cancercells: An implication of the therapeutic potential.
J Pineal Res. 2017 May;62(4).

193. Yan Y, Wang L, He J, Liu P, Lv X, Zhang Y, Xu X, Zhang L, Zhang Y.
Synergy with interferon-lambda 3 and sorafenib suppresses hepatocellular carcinomaproliferation.
Biomed Pharmacother. 2017 Apr;88:395-402.

194. Liu X, Nie ZW, Gao YY, Chen L, Yin SY, Zhang X, Hao C, Miao YL.
Sodium fluoride disturbs DNA methylation of NNAT and declines oocyte quality by impairingglucose transport in porcine oocytes.
Environ Mol Mutagen. 2017 Dec 29.

195. Zhao H, Liu Q, Wang S, Dai F, Cheng X, Cheng X, Chen W, Zhang M, Chen D.
In vitro additive antitumor effects of dimethoxycurcumin and 5-fluorouracil in colon cancer cells.
Cancer Med. 2017 Jul;6(7):1698-1706.

196. Zhang Q, Wu D, Yang Y, Liu T, Liu H.
Dexmedetomidine Alleviates Hyperoxia-Induced Acute Lung Injury via Inhibiting NLRP3Inflammasome Activation.
Cell Physiol Biochem. 2017;42(5):1907-1919.

197. Xiao J, Zhang R, Huang F, Liu L, Deng Y, Ma Y, Wei Z, Tang X, Zhang Y, Zhang M.
Lychee (Litchi chinensis Sonn.) Pulp Phenolic Extract Confers a Protective Activity againstAlcoholic Liver Disease in Mice by Alleviating Mitochondrial Dysfunction.
J Agric Food Chem. 2017 Jun 21;65(24):5000-5009.

198. Luo SW, Kang H, Kong JR, Xie RC, Liu Y, Wang WN, Xie FX, Wang C, Sun ZM.
Molecularcloning, characterization and expression analysis of(B-cell lymphoma-2) Bcl-2 inthe orange-spotted grouper (Epinephelus coioides).
Dev Comp Immunol. 2017 Nov;76:150-162.

199. Yang J, Yu J, Li D, Yu S, Ke J, Wang L, Wang Y, Qiu Y, Gao X, Zhang J, Huang L.
Store-operated calcium entry-activated autophagy protects EPC proliferation via the CAMKK2-MTOR pathway in ox-LDL exposure.
Autophagy. 2017 Jan 2;13(1):82-98.

200. Zhao J, Liao Y, Chen J, Dong X, Gao Z, Zhang H, Wu X, Liu Z, Wu Y.
Aberrant Buildup of All-Trans-Retinal Dimer,a Nonpyridinium Bisretinoid Lipofuscin Fluorophore, Contributes tothe Degeneration ofthe Retinal Pigment Epithelium.
Invest Ophthalmol Vis Sci. 2017 Feb 1;58(2):1063-1075.

201. Hu F, Zhao Y, Yu Y, Fang JM, Cui R, Liu ZQ, Guo XL, Xu Q.
Docetaxel-mediated autophagy promotes chemoresistance in castration-resistant prostatecancer cells by inhibiting STAT3.
Cancer Lett. 2018 Mar 1;416:24-30.

202. Jiang HY, Yang Y, Zhang YY, Xie Z, Zhao XY, Sun Y, Kong WJ.
The dual role of poly(ADP-ribose) polymerase-1 in modulating parthanatos and autophagy underoxidative stress in rat cochlear marginal cells of the stria vascularis.
Redox Biol. 2018 Apr;14:361-370.

203. Jia D, Li T, Chen X, Ding X, Chai Y, Chen AF, Zhu Z, Zhang C.
Salvianic acid A sodium protects HUVEC cells against tert-butyl hydroperoxide inducedoxidative injury via mitochondria-dependent pathway.
Chem Biol Interact. 2018 Jan 5;279:234-242.

204. Wang Z, Zhou F, Dou Y, Tian X, Liu C, Li H, Shen H, Chen G.
Melatonin Alleviates Intracerebral Hemorrhage-Induced Secondary Brain Injury in Rats viaSuppressing Apoptosis, Inflammation, Oxidative Stress, DNA Damage, and Mitochondria Injury.
Transl Stroke Res. 2018 Feb;9(1):74-91.

205. Zhang Y,Wang C,Yu B,Jiang JD,Kong WJ
Gastrodin Protects against Ethanol-Induced Liver Injury and Apoptosis in HepG2 Cells and Animal Models of Alcoholic Liver Disease.
Biol Pharm Bull. 2018;41(5):670-679.

206. Qiu H,Li J,Liu Q,Tang M,Wang Y
Apatinib, a novel tyrosine kinase inhibitor, suppresses tumor growth in cervical cancer and synergizes with Paclitaxel.
Cell Cycle. 2018;17(10):1235-1244.

207. Zhang H,Chen S,Zeng M,Lin D,Wang Y,Wen X,Xu C,Yang L,Fan X,Gong Y,Zhang H,Kong X
Apelin-13 Administration Protects Against LPS-Induced Acute Lung Injury by Inhibiting NF-κB Pathway and NLRP3 Inflammasome Activation.
Cell Physiol Biochem. 2018;49(5):1918-1932.

208. Xue P,Yang R,Sun L,Li Q,Zhang L,Xu Z,Kang Y
Indocyanine Green-Conjugated Magnetic Prussian Blue Nanoparticles for Synchronous Photothermal/Photodynamic Tumor Therapy.
Nanomicro Lett. 2018;10(4):74.

209. Li S,Yang X,Feng Z,Wang P,Zhu W,Cui S
Catalase Enhances Viability of Human Chondrocytes in Culture by Reducing Reactive Oxygen Species and Counteracting Tumor Necrosis Factor-α-Induced Apoptosis.
Cell Physiol Biochem. 2018;49(6):2427-2442.

210. Hu Y,Yu K,Wang G,Zhang D,Shi C,Ding Y,Hong D,Zhang D,He H,Sun L,Zheng JN,Sun S,Qian F
Lanatoside C inhibits cell proliferation and induces apoptosis through attenuating Wnt/β-catenin/c-Myc signaling pathway in human gastric cancer cell.
Biochem Pharmacol. 2018 Apr;150:280-292.

211. Jiang HY,Yang Y,Zhang YY,Xie Z,Zhao XY,Sun Y,Kong WJ
The dual role of poly(ADP-ribose) polymerase-1 in modulating parthanatos and autophagy under oxidative stress in rat cochlear marginal cells of the stria vascularis.
Redox Biol. 2018 Apr;14:361-370.

212. Liu X,Nie ZW,Gao YY,Chen L,Yin SY,Zhang X,Hao C,Miao YL
Sodium fluoride disturbs DNA methylation of NNAT and declines oocyte quality by impairing glucose transport in porcine oocytes.
Environ Mol Mutagen. 2018 Apr;59(3):223-233.

213. Liu X,Zhang N,Wang D,Zhu D,Yuan Q,Zhang X,Qian L,Niu H,Lu Y,Ren G,Tian K,Yuan H
Downregulation of reticulocalbin-1 differentially facilitates apoptosis and necroptosis in human prostate cancer cells.
Cancer Sci. 2018 Apr;109(4):1147-1157.

214. Zhou C,Ma J,Su M,Shao D,Zhao J,Zhao T,Song Z,Meng Y,Jiao P
Down-regulation of STAT3 induces the apoptosis and G1 cell cycle arrest in esophageal carcinoma ECA109 cells.
Cancer Cell Int. 2018 Apr 4;18:53.

215. Peng J,Zhou Y,Zhang Z,Wang Z,Gao L,Zhang X,Fang Z,Li G,Chen H,Yang H,Gao L
The detrimental effects of glucocorticoids exposure during pregnancy on offspring's cardiac functions mediated by hypermethylation of bone morphogenetic protein-4.
Cell Death Dis. 2018 Aug 6;9(8):834.

216. Dong A,Yu Y,Wang Y,Li C,Chen H,Bian Y,Zhang P,Zhao Y,Yu Y,Xie K
Protective effects of hydrogen gas against sepsis-induced acute lung injury via regulation of mitochondrial function and dynamics.
Int Immunopharmacol. 2018 Dec;65:366-372.

217. Ding M,Shu P,Gao S,Wang F,Gao Y,Chen Y,Deng W,He G,Hu Z,Li T
Schisandrin B protects human keratinocyte-derived HaCaT cells from tert-butyl hydroperoxide-induced oxidative damage through activating the Nrf2 signaling pathway.
Int J Mol Med. 2018 Dec;42(6):3571-3581.

218. Yin S,Zhang L,Ding L,Huang Z,Xu B,Li X,Wang P,Mao J
Transient receptor potential ankyrin 1 (trpa1) mediates il-1β-induced apoptosis in rat chondrocytes via calcium overload and mitochondrial dysfunction.
J Inflamm (Lond). 2018 Dec 17;15:27.

219. GongSun X,Zhao Y,Jiang B,Xin Z,Shi M,Song L,Qin Q,Wang Q,Liu X
Inhibition of MUC1-C regulates metabolism by AKT pathway in esophageal squamous cell carcinoma.
J Cell Physiol. 2018 Dec 6.

220. Wan GX,Cheng L,Qin HL,Zhang YZ,Wang LY,Zhang YG
MiR-15b-5p is Involved in Doxorubicin-Induced Cardiotoxicity via Inhibiting Bmpr1a Signal in H9c2 Cardiomyocyte.
Cardiovasc Toxicol. 2018 Dec 7.

221. Wang Z,Zhou F,Dou Y,Tian X,Liu C,Li H,Shen H,Chen G
Melatonin Alleviates Intracerebral Hemorrhage-Induced Secondary Brain Injury in Rats via Suppressing Apoptosis, Inflammation, Oxidative Stress, DNA Damage, and Mitochondria Injury.
Transl Stroke Res. 2018 Feb;9(1):74-91.

222. You P,Wu H,Deng M,Peng J,Li F,Yang Y
Brevilin A induces apoptosis and autophagy of colon adenocarcinoma cell CT26 via mitochondrial pathway and PI3K/AKT/mTOR inactivation.
Biomed Pharmacother. 2018 Feb;98:619-625.

223. Xie Z,Xia W,Hou M
Long intergenic non-coding RNA-p21 mediates cardiac senescence via the Wnt/β-catenin signaling pathway in doxorubicin-induced cardiotoxicity.
Mol Med Rep. 2018 Feb;17(2):2695-2704.

224. Jia J,Qin Y,Zhang L,Guo C,Wang Y,Yue X,Qian J
Sijunzi decoction-treated rat serum induces apoptosis of side population cells in gastric carcinoma.
Exp Ther Med. 2018 Feb;15(2):1718-1727.

225. Guo XL,Hu F,Wang H,Fang JM,Zhu ZＺ,Wei LＸ,Xu Q
Inhibition of autophagy in hepatocarcinoma cells promotes chemotherapeutic agent-induced apoptosis during nutrient deprivation.
Oncol Rep. 2018 Feb;39(2):773-783.

226. Wang Z,Zhou F,Dou Y,Tian X,Liu C,Li H,Shen H,Chen G
Melatonin Alleviates Intracerebral Hemorrhage-Induced Secondary Brain Injury in Rats via Suppressing Apoptosis, Inflammation, Oxidative Stress, DNA Damage, and Mitochondria Injury.
Transl Stroke Res. 2018 Feb;9(1):74-91.

227. Jiang ZM,Qiu HB,Wang SQ,Guo J,Yang ZW,Zhou SB
Ganoderic acid A potentiates the antioxidant effect and protection of mitochondrial membranes and reduces the apoptosis rate in primary hippocampal neurons in magnesium free medium.
Pharmazie. 2018 Feb 1;73(2):87-91.

228. Cao W,Zhang J,Wang G,Lu J,Wang T,Chen X

Reducing-Autophagy Derived Mitochondrial Dysfunction during Resveratrol Promotes Fibroblast-Like Synovial Cell Apoptosis.

Anat Rec (Hoboken). 2018 Feb 20.

229. Tao J,Xu J,Chen F,Xu B,Gao J,Hu Y

Folate acid-Cyclodextrin/Docetaxel induces apoptosis in KB cells via the intrinsic mitochondrial pathway and displays antitumor activity in vivo.

Eur J Pharm Sci. 2018 Jan 1;111:540-548.

230. Maryam A,Mehmood T,Yan Q,Li Y,Khan M,Ma T

Proscillaridin A Promotes Oxidative Stress and ER Stress, Inhibits STAT3 Activation, and Induces Apoptosis in A549 Lung Adenocarcinoma Cells.

Oxid Med Cell Longev. 2018 Jan 11;2018:3853409.

231. Jia D,Li T,Chen X,Ding X,Chai Y,Chen AF,Zhu Z,Zhang C

Salvianic acid A sodium protects HUVEC cells against tert-butyl hydroperoxide induced oxidative injury via mitochondria-dependent pathway.

Chem Biol Interact. 2018 Jan 5;279:234-242.

232. Wang D,Wang Z,Zhang L,Li Z,Tian X,Fang J,Lu Q,Zhang X

Cellular ATP levels are affected by moderate and strong static magnetic fields.

Bioelectromagnetics. 2018 Jul;39(5):352-360.

233. Li W,Li C,Chen S,Sun L,Li H,Chen L,Zhou X

Effect of inhibin A on proliferation of porcine granulosa cells in vitro.

Theriogenology. 2018 Jul 1;114:136-142.

234. Zha ZM,Wang JH,Li SL,Guo Y

Pitavastatin attenuates AGEs-induced mitophagy via inhibition of ROS generation in the mitochondria of cardiomyocytes.

J Biomed Res. 2018 Jul 23;32(4):281-287.

235. Li X,Chen M,Yang Z,Wang W,Lin H,Xu S

Selenoprotein S silencing triggers mouse hepatoma cells apoptosis and necrosis involving in intracellular calcium imbalance and ROS-mPTP-ATP.

Biochim Biophys Acta Gen Subj. 2018 Jul 7;1862(10):2113-2123.

236. Tao H,Qian P,Lu J,Guo Y,Zhu H,Wang F

Autophagy inhibition enhances radiosensitivity of Eca-109 cells via the mitochondrial apoptosis pathway.

Int J Oncol. 2018 Jun;52(6):1853-1862.

237. Qi G,Guo R,Tian H,Li L,Liu H,Mi Y,Liu X

Nobiletin protects against insulin resistance and disorders of lipid metabolism by reprogramming of circadian clock in hepatocytes.

Biochim Biophys Acta Mol Cell Biol Lipids. 2018 Jun;1863(6):549-562.

238. Yang X,Tang S,Li D,Yu X,Wang F,Xiao X

DIDS inhibits overexpression BAK1-induced mitochondrial apoptosis through GSK3β/β-catenin signaling pathway.

J Cell Physiol. 2018 Jun;233(6):5070-5077.

239. Zhang X,Zhuang R,Wu H,Chen J,Wang F,Li G,Wu C

A novel role of endocan in alleviating LPS-induced acute lung injury.

Life Sci. 2018 Jun 1;202:89-97.

240. Zeng X,Chen S,Lin Y,Ke Z

Acylated and unacylated ghrelin inhibit apoptosis in myoblasts cocultured with colon carcinoma cells.

Oncol Rep. 2018 Mar;39(3):1387-1395.

241. Han H,Chen W,Yang J,Liang X,Wang Y,Li Q,Yang Y,Li K

Inhibition of cell proliferation and migration through nucleobase-modified polyamidoamine-mediated p53 delivery.

Int J Nanomedicine. 2018 Mar 6;13:1297-1311.

242. Zhang X,Yan Z,Xu T,An Z,Chen W,Wang X,Huang M,Zhu F

Solamargine derived from Solanum nigrum induces apoptosis of human cholangiocarcinoma QBC939 cells.

Oncol Lett. 2018 May;15(5):6329-6335.

243. Zhao H,Yan L,Xu X,Jiang C,Shi J,Zhang Y,Liu L,Lei S,Shao D,Huang Q

Potential of Bacillus subtilis lipopeptides in anti-cancer I: induction of apoptosis and paraptosis and inhibition of autophagy in K562 cells.

AMB Express. 2018 May 9;8(1):78.

244. Zhao Y,Zhao R,Wu J,Wang Q,Pang K,Shi Q,Gao Q,Hu Y,Dong X,Zhang J,Sun J

Melatonin protects against Aβ-induced neurotoxicity in primary neurons via miR-132/PTEN/AKT/FOXO3a pathway.

Biofactors. 2018 Nov;44(6):609-618.

245. Wu S,Lei L,Song Y,Liu M,Lu S,Lou D,Shi Y,Wang Z,He D

Mutation of hop-1 and pink-1 attenuates vulnerability of neurotoxicity in C. elegans: the role of mitochondria-associated membrane proteins in Parkinsonism.

Exp Neurol. 2018 Nov;309:67-78.

246. Gao X,Guo S,Zhang S,Liu A,Shi L,Zhang Y

Matrine attenuates endoplasmic reticulum stress and mitochondrion dysfunction in nonalcoholic fatty liver disease by regulating SERCA pathway.

J Transl Med. 2018 Nov 20;16(1):319.

247. Liu HN,Guo NN,Guo WW,Huang-Fu MY,Vakili MR,Chen JJ,Xu WH,Wei QC,Han M,Lavasanifar A,Gao JQ

Delivery of mitochondriotropic doxorubicin derivatives using self-assembling hyaluronic acid nanocarriers in doxorubicin-resistant breast cancer.

Acta Pharmacol Sin. 2018 Oct;39(10):1681-1692.

248. Liu Y,Ren L,Liu W,Xiao Z

MiR-21 regulates the apoptosis of keloid fibroblasts by caspase-8 and the mitochondria-mediated apoptotic signaling pathway via targeting FasL.

Biochem Cell Biol. 2018 Oct;96(5):548-555.

249. Liu L,Zuo Z,Lu S,Wang L,Liu A,Liu X

Silencing of PINK1 represses cell growth, migration and induces apoptosis of lung cancer cells.

Biomed Pharmacother. 2018 Oct;106:333-341.

250. Xia W,Zhuang L,Hou M

Role of lincRNA-p21 in the protective effect of macrophage inhibition factor against hypoxia/serum deprivation-induced apoptosis in mesenchymal stem cells.

Int J Mol Med. 2018 Oct;42(4):2175-2184.

251. Chen L,Li W,Qi D,Lu L,Zhang Z,Wang D

Honokiol protects pulmonary microvascular endothelial barrier against lipopolysaccharide-induced ARDS partially via the Sirt3/AMPK signaling axis.

Life Sci. 2018 Oct 1;210:86-95.

252. Du X,Yin S,Zhou F,Du X,Xu J,Gu X,Wang G,Li J

Reduction-sensitive mixed micelles for selective intracellular drug delivery to tumor cells and reversal of multidrug resistance.

Int J Pharm. 2018 Oct 25;550(1-2):1-13.

253. Jin L,Cai Q,Wang S,Wang S,Mondal T,Wang J,Quan Z

Long noncoding RNA MEG3 regulates LATS2 by promoting the ubiquitination of EZH2 and inhibits proliferation and invasion in gallbladder cancer.

Cell Death Dis. 2018 Oct 3;9(10):1017.

注： 更多使用本产品的文献请参考产品网页。

Version 2019.06.13
