# Supplementary material for: Combination decoction of Astragalus mongholicus and Salvia miltiorrhiza mitigates pressure-overload cardiac dysfunction by inhibiting multiple ferroptosis pathways
Source: Front Pharmacol. 2024 Dec 16;15:1447546. doi: 10.3389/fphar.2024.1447546 (PMC11683366; doi:10.3389/fphar.2024.1447546)
Supplement: Supplementary file 4 [file DataSheet1.ZIP › E-BC-K773-M-Elabscience.pdf]

（本试剂盒仅供体外研究使用，不用于临床诊断！）

Elabscience®亚铁离子比色法测试盒

Ferrous Iron Colorimetric Assay Kit

产品货号：E-BC-K773-M

产品规格：48T(32 samples)/96T(80 samples)

检测仪器：酶标仪(590-600 nm)

使用前请仔细阅读说明书。如果有任何问题，请通过以下方式联系我们：

销售部电话	027-87879180，027-87854967
技术部电话	13129966790
电子邮箱（销售）	Perry@elabscience.cn
电子邮箱（技术）	biochemical@elabscience.cn
网址	www.elabscience.cn

具体保质期请见试剂盒外包装标签。请在保质期内使用试剂盒。

联系时请提供产品批号(见试剂盒标签)，以便我们更高效地为您服务。

用途

本试剂盒适用于检测血清、动植物组织样本中亚铁离子的含量。

检测原理

样本中的亚铁离子与探针结合，生成的物质在 593 nm 波长处有强吸收峰，在一定范围内其光密度值与亚铁离子浓度成线性相关。

提供试剂和物品

编号	名称	规格 1 (Size 1)(48 T)	规格 2 (Size 2)(96 T)	保存方式 (Storage)
试剂一 (Reagent 1)	缓冲液 (Buffer Solution)	35 mL×2 瓶	60 mL×2 瓶	2-8°C 避光保存 6 个月
试剂二 (Reagent 2)	显色液 (Chromogenic Solution)	10 mL×1 瓶	20 mL×1 瓶	2-8°C 避光保存 6 个月
试剂三 (Reagent 3)	10 mmol/L 铁标准品 (10 mmol/L Iron Standard)	2 mL×1 瓶	2 mL×1 瓶	2-8°C 避光保存 6 个月
试剂四 (Reagent 4)	标准品保护剂 (Standard Protectant)	粉剂×1 瓶	粉剂×1 瓶	2-8°C 避光保存 6 个月
试剂五 (Reagent 5)	提取剂（Extracting Solution）	40mL×1 瓶	40 mL×2 瓶	2-8°C 避光保存 6 个月
	96 孔酶标板	1 板		
	96 孔覆膜	2 张		
	样本位置标记表	1 张		

说明：试剂严格按上表中的保存条件保存，不同测试盒中的试剂不能混用。

对于体积较少的试剂，使用前请先离心，以免量取不到足够量的试剂。

所需自备物品

仪器：酶标仪(590 - 600 nm，最佳检测波长为 593 nm)

试剂准备

① 检测前，试剂盒中的试剂平衡至室温。

② 标准品保护液的配制：

取一瓶试剂四，加入20 mL试剂一混合均匀。配制好的试剂可在2-8℃保存一个月。

③ 100 μmol/L铁标准品的配制：

取20 μL试剂三，与1980 μL标准品保护液混合均匀。按需配置，现配现用。

④ 不同浓度标准品的稀释：

编号	①	②	③	④	⑤	⑥	⑦	⑧
**标准品浓度(μmol/L)**	**0**	**5**	**10**	**15**	**20**	**30**	**40**	**50**
**100 μmol/L 标准品(μL)**	0	50	100	150	200	300	400	500
**标准品保护液(μL)**	1000	950	900	850	800	700	600	500

样本准备

① 样本处理

组织样本：取 0.1 g 新鲜组织块，加入 0.9 mL 试剂五匀浆，12000×g 离心 10 min，取上清液备用。

血清样本：取血清样本 55 μL 与试剂一 165 μL 混合均匀备用。如果血清样本浑浊，5000×g 离心 5 min，取澄清部分使用。

② 样本的稀释

在正式检测前，需选择2-3个预期差异大的样本稀释成不同浓度进行预实验，根据预实验的结果，结合本试剂盒的线性范围：0.4 - 50 μmol/L，请参考下表稀释(仅供参考)：

样本	稀释倍数	样本	稀释倍数
10%小鼠肝组织	1-3	10%大鼠肺组织	不稀释
10%小鼠心组织	不稀释	10%大鼠脾组织	2-3
10%绿萝叶片组织	不稀释	大鼠血清	不稀释
人血清	不稀释	小鼠血清	1-2

注： 组织样本稀释液为试剂五；

血清样本稀释液为试剂一；血清样本在样本处理过程中已稀释 4 倍。

实验关键点

① 为避免污染试剂二，可用 EP 管将试剂二分装一部分使用。

② 在使用移液枪向酶标板中加入液体时避免气泡产生。

③ 尽量选取新鲜的样本进行实验。

操作步骤

动植物组织的测定

① 标准管：取 300 μL 不同浓度标准品，分别加入对应的 1.5 mL 的 EP 管中。

测定管：取 300 μL 样本，加入对应的 1.5 mL 的 EP 管中。

② 向步骤①各管中加入 150 μL 试剂二。

③ 混合均匀，37 ℃ 孵育 10 min。

④ 将各管以 12000×g 离心 10 min。

⑤ 取 300 μL 步骤④中各管上清液加入酶标板各对应孔中。

⑥ 在酶标仪 593 nm 处测定各孔 OD 值。

血清（浆）的测定

① 标准孔：取 200 μL 不同浓度标准品，分别加入酶标板相应孔中。

测定孔：取 200 μL 样本，加入酶标板相应孔中。

② 向步骤①各孔中加入 100 μL 试剂二。

③ 混匀，37 ℃ 孵育 10 min。

④ 在酶标仪 593 nm 处测定各孔 OD 值。

操作表

动植物组织的测定

	标准管	测定管
不同浓度标准品(μL)	300	--
待测样本(μL)	--	300
试剂二(μL)	150	150
混匀，37℃孵育 10 min，12000×g 离心 10 min，取 300 μL 上清液于酶标板各对应孔中，在酶标仪 593 nm 处测定各孔 OD 值。		

血清（浆）的测定

	标准孔	测定孔
不同浓度标准品(μL)	200	--
待测样本(μL)	--	200
试剂二(μL)	100	100
混匀，37℃孵育 10 min，在酶标仪 593 nm 处测定各孔 OD 值。		

结果计算

标准品拟合曲线： y = ax + b

血清样本：

$$\frac{Fe^{2+}\text{含量}}{(\mu mol/L)} = \frac{\Delta A - b}{a} \times 4^{*} \times f$$

组织样本(以组织湿重进行计算)：

$$\frac{Fe^{2+}\text{含量}}{(\mu mol/kg\ wet\ weight)} = \frac{\Delta A - b}{a} \times f \div \frac{m}{V}$$

注解：

y：标准品 OD 值-空白 OD 值(标准品浓度为 0 时的 OD 值)

x：标准品的浓度

a：标曲的斜率

b：标曲的截距

ΔA：样本的绝对 OD 值(测定孔 OD 值-空白孔 OD 值)

4*：血清与试剂一混合后被稀释了四倍

V：样本匀浆液加入量(mL)

f：样本加入检测体系前的稀释倍数

m：组织湿重质量（g）

附录1 关键数据

1. 技术参数

检测范围	0.4 - 50 μmol/L	平均批间差	1.5 %
灵敏度	0.4 μmol/L	平均批内差	1.3 %
平均回收率	99%		

2. 标准曲线(数据仅供参考)

①不同浓度的标准品，按照操作步骤进行实验，测得各浓度标准品OD值如下表所示：

标准品浓度 (μmol/L)	0	5	10	15	20	30	40	50
OD 值	0.048	0.135	0.233	0.327	0.416	0.613	0.797	0.980
	0.048	0.137	0.230	0.324	0.418	0.609	0.792	0.976
平均 OD 值	0.048	0.136	0.232	0.326	0.417	0.611	0.795	0.978
绝对 OD 值	0.000	0.088	0.184	0.278	0.369	0.563	0.747	0.930

②按上表数据绘制标准曲线，如下图所示：

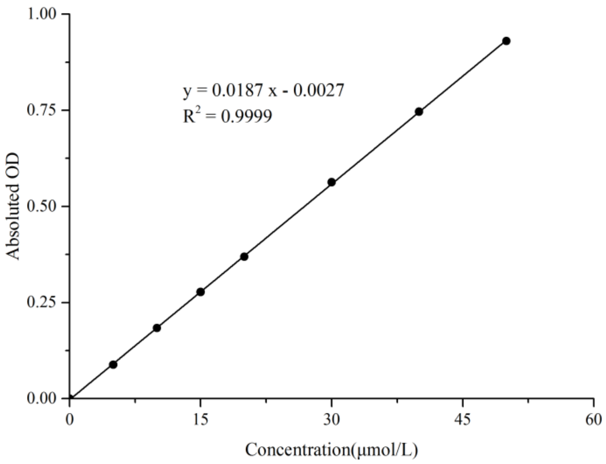

附录2 实例分析

例如检测大鼠肝组织(数据仅供参考):

将10%大鼠肝组织匀浆稀释2倍，取300 μL按操作表进行检测，其结果如下：

标准曲线： y = 0.0187 x - 0.0027，测定孔平均OD值为0.110，空白孔平均OD值为0.042，亚铁离子含量计算结果为：

$$\text{Fe}^{2+}\text{含量}\ (\mu\text{mol/kg wet weight}) = \frac{0.110 - 0.042 + 0.0027}{0.0187} \times 2 \div \frac{0.1}{0.9} = 68.05\ \mu\text{mol/kg wet weight}$$

按照说明书操作，测定大鼠肝组织(稀释2倍，加样量为300 μL)、大鼠肾组织(稀释2倍，加样量为300 μL)、大鼠脑组织(稀释2倍，加样量为300 μL)和人血清(加样量为200 μL))中的亚铁含量(如下图)：

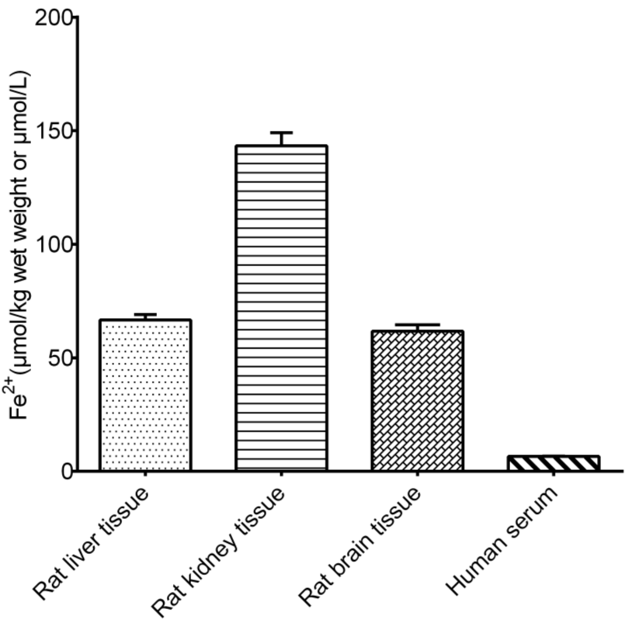

附录3 问题答疑

问题	可能原因	建议解决方案
无法检测出样本含量	细胞中 Fe^{2+}含量低于检出限	增加样本匀浆浓度
复孔差异大	样本中有固体杂质未去除干净	延长孵育后离心时间
	酶标板孔中有气泡	加液时将枪头贴近孔内壁缓慢加入

声明

1. 试剂盒仅供研究使用，如将其用于临床诊断或任何其他用途，我公司将不对因此产生的问题负责，亦不承担任何法律责任。
2. 实验前请仔细阅读说明书并调整好仪器，严格按照说明书进行实验。
3. 实验中请穿着实验服并戴乳胶手套做好防护工作。
4. 试剂盒检测范围不等同于样本中待测物的浓度范围。如果样品中待测物浓度过高或过低，请对样本做适当的稀释或浓缩。
5. 若所检样本不在说明书所列样本类型之中，建议先做预实验验证其检测有效性。
6. 最终的实验结果与试剂的有效性、实验者的相关操作以及实验环境等因素密切相关。本公司只对试剂盒本身负责，不对因使用试剂盒所造成的样本消耗负责，使用前请充分考虑样本可能的使用量，预留充足的样本。

附录4 客户发表文献

1. Du S, Zhou N, Xie G, et al. Surface-engineered triboelectric nanogenerator patches with drug loading and electrical stimulation capabilities: Toward promoting infected wounds healing[J]. Nano Energy, 2021, 85:106004. IF:17.087
2. Bartolini D, Arato I, Mancuso F, et al. Melatonin modulates Nrf2 activity to protect porcine pre-pubertal Sertoli cells from the abnormal H_2O_2 generation and reductive stress effects of cadmium. J Pineal Res. 2022;73 (1):e12806. IF:13.007
3. Yang Z, Wang J, Ai S, et al. Self-generating oxygen enhanced mitochondrion-targeted photodynamic therapy for tumor treatment with hypoxia scavenging[J]. Theranostics, 2019, 9(23): 6809. IF:11.556
4. Wan Q, Cao R, Wen G, et al. Sequential use of UV-LEDs irradiation and chlorine to disinfect waterborne fungal spores: Efficiency, mechanism and photoreactivation[J]. Journal of Hazardous Materials, 2022, 423:127102-. IF:10.588
5. Tian J, Wang L, Hui S, et al. Cadmium accumulation regulated by a rice heavy-metal importer is harmful for host plant and leaf bacteria. J Adv Res. 2022. IF:10.479
6. Jg A, Jie S B, Jy B, et al. Comparative toxicity reduction potential of UV/sodium percarbonate and UV/hydrogen peroxide treatments for bisphenol A in water: An integrated analysis using chemical, computational, biological, and metabolomic approaches[J]. Water Research, 2020, 190. IF:9.702
7. Yang X X, Xu X, Wang M F, et al. A nanoreactor boosts chemodynamic therapy and ferroptosis for synergistic cancer therapy using molecular amplifier dihydroartemisinin[J]. Journal of Nanobiotechnology, 2022, 20(1):1-19. IF:9.464
8. Liu Z, Liu X, Yang Q, et al. Neutrophil membrane-enveloped nanoparticles for the amelioration of renal ischemia-reperfusion injury in mice[J]. Acta Biomaterialia, 2020, 104: 158-166. IF:8.947
9. Huang S, Le H, Hong G, et al. An all-in-one biomimetic iron-small interfering RNA nanoplatform induces ferroptosis for cancer therapy. Acta Biomater. 2022;148:244-257. IF:8.291
10. Alharbi YM, Sakr SS, Albarrak SM, et al. Antioxidative, Antidiabetic, and Hypolipidemic Properties of Probiotic-Enriched Fermented Camel Milk Combined with Salvia officinalis Leaves Hydroalcoholic Extract in Streptozotocin-Induced Diabetes in Rats. Antioxidants (Basel). 2022;11 (4):. IF:7.675
11. Wang H, Huang Q, Zhang Z, et al. Transient post-operative overexpression of CXCR2 on

monocytes of traumatic brain injury patients drives monocyte chemotaxis toward cerebrospinal fluid and enhances monocyte-mediated immunogenic cell death of neurons in vitro[J]. Journal of Neuroinflammation, 2022. IF:7.573

12. Liu P, Yin Z, Chen M, et al. Cytotoxicity of adducts formed between quercetin and methylglyoxal in PC-12 cells[J]. Food Chemistry, 2021, 352(2):129424. IF:7.514
13. Adhikari B, Adhikari M, Ghimire B, et al. Cold plasma seed priming modulates growth, redox homeostasis and stress response by inducing reactive species in tomato (Solanum lycopersicum)[J]. Free Radical Biology and Medicine, 2020, 156: 57-69. IF:7.376
14. Zhao X, Wang C,Dai S, et al. Quercetin Protects Ethanol-Induced Hepatocyte Pyroptosis via Scavenging Mitochondrial ROS and Promoting PGC-1 α -Regulated Mitochondrial Homeostasis in L02 Cells. Oxid Med Cell Longev. 2022;2022:4591134. IF:7.31
15. Chagas TQ, Freitas ÍN, Montalvão MF, Nobrega RH, Machado MRF, Charlie-Silva I, Araújo APDC, Guimarães ATB, Alvarez TGDS, Malafaia G. Multiple endpoints of polylactic acid biomicroplastic toxicity in adult zebrafish (Danio rerio)[J]. Chemosphere. 2021 Aug;277:130279. IF:7.086
16. Mlindeli Gamede, Lindokuhle Mabuza, Phikelelani Ngubane, et al. Preventing the onset of diabetes-induced chronic kidney disease during prediabetes: The effects of oleanolic acid on selected markers of chronic kidney disease in a diet-induced prediabetic rat model[J]. Biomedicine & Pharmacotherapy.2021 Jul;139:111570. IF:6.529
17. Yang H, Zhu Y, Ye Y, et al. Nitric oxide protects against cochlear hair cell damage and noise-induced hearing loss through glucose metabolic reprogramming.[J]. Free radical biology & medicine, 2021. IF:6.525
18. Rao M J, Xu Y, Tang X, et al. CsCYT75B1, a Citrus CYTOCHROME P450 Gene, Is Involved in Accumulation of Antioxidant Flavonoids and Induces Drought Tolerance in Transgenic Arabidopsis[J]. Antioxidants & Redox Signaling, 2020, 9(2):161. IF:6.313
19. Liou G G, Hsieh C C, Lee Y J, et al. N-Acetyl Cysteine Overdose Inducing Hepatic Steatosis and Systemic Inflammation in Both Propacetamol-Induced Hepatotoxic and Normal Mice[J]. Antioxidants, 2021, 10(3):442. IF:6.312
20. Wang Y, Chi H, Xu F, et al. Cadmium chloride-induced apoptosis of HK-2 cells via interfering with mitochondrial respiratory chain[J]. Ecotoxicology and Environmental Safety, 2022, 236:113494-. IF:6.233
