# Supplementary material for: Combination decoction of Astragalus mongholicus and Salvia miltiorrhiza mitigates pressure-overload cardiac dysfunction by inhibiting multiple ferroptosis pathways
Source: Front Pharmacol. 2024 Dec 16;15:1447546. doi: 10.3389/fphar.2024.1447546 (PMC11683366; doi:10.3389/fphar.2024.1447546)
Supplement: Supplementary file 4 [file DataSheet1.ZIP › E-BC-K881-M-Elabscience.pdf]

（本试剂盒仅供体外研究使用，不用于临床诊断！）

Elabscience®细胞亚铁比色法测试盒

Cell Ferrous Iron Colorimetric Assay Kit

产品货号：E-BC-K881-M

产品规格：48T(16 samples)/96T(40 samples)

检测仪器：酶标仪(590-600 nm)

使用前请仔细阅读说明书。如果有任何问题，请通过以下方式联系我们：

销售部电话	027-87879180，027-87854967
技术部电话	13129966790
电子邮箱（销售）	Perry@elabscience.cn
电子邮箱（技术）	biochemical@elabscience.cn
网址	www.elabscience.cn

具体保质期请见试剂盒外包装标签。请在保质期内使用试剂盒。

联系时请提供产品批号(见试剂盒标签)，以便我们更高效地为您服务。

用途

本试剂盒适用于检测细胞样本中亚铁的含量。

检测原理

铁是生物体内重要的金属元素之一，具有重要的生理作用。亚铁离子是血红素和血红蛋白中的关键元素，同时也在许多生化反应中起到重要作用。样本中的亚铁离子与探针结合，生成的物质在 593 nm 波长处有强吸收峰，在一定范围内其光密度值与亚铁离子浓度成线性相关。

提供试剂和物品

编号	名称	规格 1<br>(Size 1)(48 T)	规格 2<br>(Size 2)(96 T)	保存方式<br>(Storage)
试剂一<br>(Reagent 1)	缓冲液<br>(Buffer Solution)	45 mL×1 瓶	35 mL×2 瓶	2-8°C 避光<br>保存 6 个月
试剂二<br>(Reagent 2)	对照液<br>(Control Solution)	5 mL×1 瓶	10 mL×1 瓶	2-8°C 避光<br>保存 6 个月
试剂三<br>(Reagent 3)	显色液<br>(Chromogenic Solution)	5 mL×1 瓶	10 mL×1 瓶	2-8°C 避光<br>保存 6 个月
试剂四<br>(Reagent 4)	10 mmol/L 铁标准品<br>(10 mmol/L Iron Standard)	2 mL×1 瓶	2 mL×1 瓶	2-8°C 避光<br>保存 6 个月
试剂五<br>(Reagent 5)	标准品保护剂<br>(Standard Protectant)	粉剂×1 瓶	粉剂×1 瓶	2-8°C 避光<br>保存 6 个月
	96 孔酶标板	1 板		
	96 孔覆膜	2 张		
	样本位置标记表	1 张		

说明：试剂严格按上表中的保存条件保存，不同测试盒中的试剂不能混用。

对于体积较少的试剂，使用前请先离心，以免量取不到足够量的试剂。

所需自备物品

仪器：酶标仪(590 - 600 nm，最佳检测波长为 593 nm)

试剂准备

① 检测前，试剂盒中的试剂平衡至室温。

② 标准品保护液的配制：

取一瓶试剂五，加入15 mL试剂一混合均匀。配制好的试剂可在2-8℃保存一个月。

③ 100 μmol/L铁标准品的配制：

取10 μL试剂四，与990 μL标准品保护液混合均匀。按需配置，现配现用。

④ 不同浓度标准品的稀释：

编号	①	②	③	④	⑤	⑥	⑦	⑧
**标准品浓度(μmol/L)**	**0**	**5**	**10**	**15**	**20**	**30**	**40**	**50**
**100 μmol/L 标准品(μL)**	0	25	50	75	100	150	200	250
**标准品保护液(μL)**	500	475	450	425	400	350	300	250

样本准备

① 样本处理

细胞样本:取收集好的细胞按约每 1×10^6 个细胞加入 0.2 mL 的试剂一，混匀后放置在冰盒上裂解 10 min，再 15000×g 离心 10 min，取上清液备用。

② 样本的稀释

在正式检测前，需选择2-3个预期差异大的样本稀释成不同浓度进行预实验，根据预实验的结果，结合本试剂盒的线性范围：0.4 - 50 μmol/L，请参考下表稀释(仅供参考):

样本	稀释倍数	样本	稀释倍数
HepG2 细胞	不稀释	molt-4 细胞	不稀释
Jurkat 细胞	不稀释	HEL 细胞	不稀释

注：稀释液为试剂一。

实验关键点

① 为避免污染试剂三，可用 EP 管将试剂三分装一部分使用。

② 在使用移液枪向酶标板中加入液体时避免气泡产生。

③ 尽量选取新鲜的细胞样本进行实验。

操作步骤

① 标准孔：取 80 μL 不同浓度标准品，分别加入酶标板相应孔中。
测定孔：取 80 μL 待测样本，加入酶标板相应孔中。
对照孔：取 80 μL 待测样本，加入酶标板相应孔中。

② 向对照孔中加入 80 μL 试剂二。

③ 向测定孔、标准孔中加入 80 μL 试剂三。

④ 混匀，37 ℃ 孵育 10 min。

⑤ 在酶标仪 593 nm 处测定各孔 OD 值。

操作表

	标准孔	对照孔	测定孔
不同浓度标准品(μL)	80	--	--
待测样本(μL)	--	80	80
试剂二(μL)	--	80	--
试剂三(μL)	80	--	80
37 ℃ 孵育 10 min，在酶标仪 593 nm 处测定各孔 OD 值。			

结果计算

标准品拟合曲线： y = ax + b

细胞样本(以细胞个数进行计算)：

$$\text{Fe}^{2+}\text{含量}\ (\text{nmol}/10\text{\^{}}6) = \frac{\Delta A - b}{a} \div \frac{N}{V} \times f$$

注解:

y：标准品 OD 值-空白 OD 值(标准品浓度为 0 时的 OD 值)

x：标准品的浓度

a：标曲的斜率

b：标曲的截距

ΔA：样本的绝对 OD 值(测定孔 OD 值-对照孔 OD 值)

N：用于裂解的细胞样本数量/10^6

V：细胞样本处理时试剂一加入量(mL)

f：样本加入检测体系前的稀释倍数

附录1 关键数据

1. 技术参数

检测范围	0.4 - 50 μmol/L	平均批间差	1.5 %
灵敏度	0.4 μmol/L	平均批内差	1.3 %
平均回收率	99%		

2. 标准曲线(数据仅供参考)

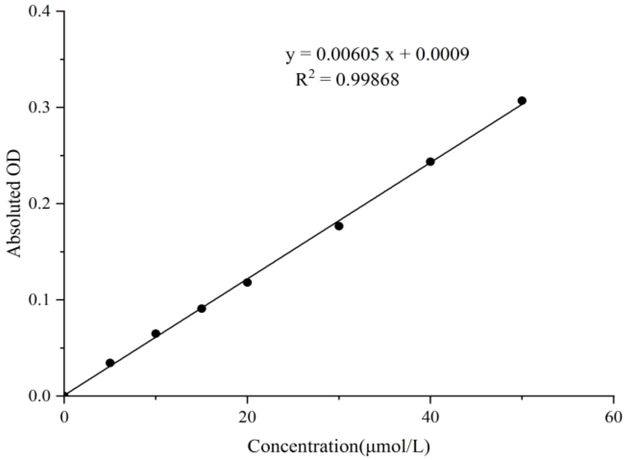

附录2 实例分析

例如检测HepG2细胞(数据仅供参考):

取1.5×10^6个HepG2细胞加入0.2 mL试剂一，经过样本处理后，取80 μL按操作表进行检测，其结果如下： 标准曲线： y = 0.00605 x + 0.0009，测定孔平均OD值为0.055，对照孔平均OD值为0.043，亚铁离子含量计算结果为：

$$Fe^{2+}\text{含量}\ (\text{nmol}/10\text{\^{}}6) = \frac{0.055 - 0.043 - 0.0009}{0.00605} \div \frac{1.5}{0.2} = 0.24\ \text{nmol}/10\text{\^{}}6$$

按照说明书操作，HepG2细胞（约1.5×10^6个细胞加入0.2 mL试剂一）、Jurkat、Molt-4、HEL细胞(约1×10^6个细胞加入0.2mL试剂一)中的亚铁含量(如下图)：

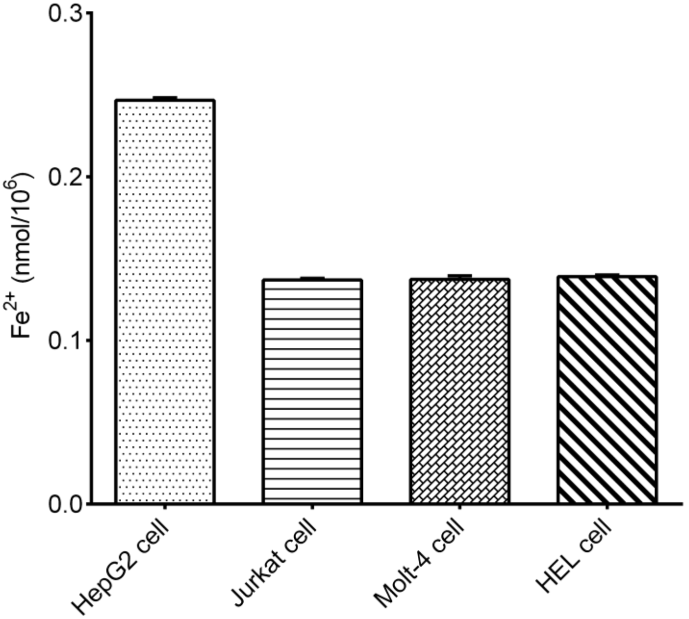

附录3 问题答疑

问题	可能原因	建议解决方案
无法检测出样本含量	细胞中 Fe^{2+}含量低于检出限	增加细胞个数
复孔差异大	样本中有固体杂质未去除干净	延长离心时间
	酶标板孔中有气泡	加液时将枪头贴近孔内壁缓慢加入

声明

1. 试剂盒仅供研究使用，如将其用于临床诊断或任何其他用途，我公司将不对因此产生的问题负责，亦不承担任何法律责任。
2. 实验前请仔细阅读说明书并调整好仪器，严格按照说明书进行实验。
3. 实验中请穿着实验服并戴乳胶手套做好防护工作。
4. 试剂盒检测范围不等同于样本中待测物的浓度范围。如果样品中待测物浓度过高或过低，请对样本做适当的稀释或浓缩。
5. 若所检样本不在说明书所列样本类型之中，建议先做预实验验证其检测有效性。
6. 最终的实验结果与试剂的有效性、实验者的相关操作以及实验环境等因素密切相关。本公司只对试剂盒本身负责，不对因使用试剂盒所造成的样本消耗负责，使用前请充分考虑样本可能的使用量，预留充足的样本。

附录4 客户发表文献

1. Du S, Zhou N, Xie G, et al. Surface-engineered triboelectric nanogenerator patches with drug loading and electrical stimulation capabilities: Toward promoting infected wounds healing[J]. Nano Energy, 2021, 85:106004. IF:17.087
2. Bartolini D, Arato I, Mancuso F, et al. Melatonin modulates Nrf2 activity to protect porcine pre-pubertal Sertoli cells from the abnormal H_2O_2 generation and reductive stress effects of cadmium. J Pineal Res. 2022;73 (1):e12806. IF:13.007
3. Yang Z, Wang J, Ai S, et al. Self-generating oxygen enhanced mitochondrion-targeted photodynamic therapy for tumor treatment with hypoxia scavenging[J]. Theranostics, 2019, 9(23): 6809. IF:11.556
4. Wan Q, Cao R, Wen G, et al. Sequential use of UV-LEDs irradiation and chlorine to disinfect waterborne fungal spores: Efficiency, mechanism and photoreactivation[J]. Journal of Hazardous Materials, 2022, 423:127102-. IF:10.588
5. Tian J, Wang L, Hui S, et al. Cadmium accumulation regulated by a rice heavy-metal importer is harmful for host plant and leaf bacteria. J Adv Res. 2022. IF:10.479
6. Jg A, Jie S B, Jy B, et al. Comparative toxicity reduction potential of UV/sodium percarbonate and UV/hydrogen peroxide treatments for bisphenol A in water: An integrated analysis using chemical, computational, biological, and metabolomic approaches[J]. Water Research, 2020, 190. IF:9.702
7. Yang X X, Xu X, Wang M F, et al. A nanoreactor boosts chemodynamic therapy and ferroptosis for synergistic cancer therapy using molecular amplifier dihydroartemisinin[J]. Journal of Nanobiotechnology, 2022, 20(1):1-19. IF:9.464
8. Liu Z, Liu X, Yang Q, et al. Neutrophil membrane-enveloped nanoparticles for the amelioration of renal ischemia-reperfusion injury in mice[J]. Acta Biomaterialia, 2020, 104: 158-166. IF:8.947
9. Huang S, Le H, Hong G, et al. An all-in-one biomimetic iron-small interfering RNA nanoplatform induces ferroptosis for cancer therapy. Acta Biomater. 2022;148:244-257. IF:8.291
10. Alharbi YM, Sakr SS, Albarrak SM, et al. Antioxidative, Antidiabetic, and Hypolipidemic Properties of Probiotic-Enriched Fermented Camel Milk Combined with Salvia officinalis Leaves Hydroalcoholic Extract in Streptozotocin-Induced Diabetes in Rats. Antioxidants (Basel). 2022;11 (4):. IF:7.675
11. Wang H, Huang Q, Zhang Z, et al. Transient post-operative overexpression of CXCR2 on

monocytes of traumatic brain injury patients drives monocyte chemotaxis toward cerebrospinal fluid and enhances monocyte-mediated immunogenic cell death of neurons in vitro[J]. Journal of Neuroinflammation, 2022. IF:7.573

12. Liu P, Yin Z, Chen M, et al. Cytotoxicity of adducts formed between quercetin and methylglyoxal in PC-12 cells[J]. Food Chemistry, 2021, 352(2):129424. IF:7.514

13. Adhikari B, Adhikari M, Ghimire B, et al. Cold plasma seed priming modulates growth, redox homeostasis and stress response by inducing reactive species in tomato (Solanum lycopersicum)[J]. Free Radical Biology and Medicine, 2020, 156: 57-69. IF:7.376

14. Zhao X, Wang C,Dai S, et al. Quercetin Protects Ethanol-Induced Hepatocyte Pyroptosis via Scavenging Mitochondrial ROS and Promoting PGC-1 α -Regulated Mitochondrial Homeostasis in L02 Cells. Oxid Med Cell Longev. 2022;2022:4591134. IF:7.31

15. Chagas TQ, Freitas ÍN, Montalvão MF, Nobrega RH, Machado MRF, Charlie-Silva I, Araújo APDC, Guimarães ATB, Alvarez TGDS, Malafaia G. Multiple endpoints of polylactic acid biomicroplastic toxicity in adult zebrafish (Danio rerio)[J]. Chemosphere. 2021 Aug;277:130279. IF:7.086

16. Mlindeli Gamede, Lindokuhle Mabuza, Phikelelani Ngubane, et al. Preventing the onset of diabetes-induced chronic kidney disease during prediabetes: The effects of oleanolic acid on selected markers of chronic kidney disease in a diet-induced prediabetic rat model[J]. Biomedicine & Pharmacotherapy.2021 Jul;139:111570. IF:6.529

17. Yang H, Zhu Y, Ye Y, et al. Nitric oxide protects against cochlear hair cell damage and noise-induced hearing loss through glucose metabolic reprogramming.[J]. Free radical biology & medicine, 2021. IF:6.525

18. Rao M J, Xu Y, Tang X, et al. CsCYT75B1, a Citrus CYTOCHROME P450 Gene, Is Involved in Accumulation of Antioxidant Flavonoids and Induces Drought Tolerance in Transgenic Arabidopsis[J]. Antioxidants & Redox Signaling, 2020, 9(2):161. IF:6.313

19. Liou G G, Hsieh C C, Lee Y J, et al. N-Acetyl Cysteine Overdose Inducing Hepatic Steatosis and Systemic Inflammation in Both Propacetamol-Induced Hepatotoxic and Normal Mice[J]. Antioxidants, 2021, 10(3):442. IF:6.312

20. Wang Y, Chi H, Xu F, et al. Cadmium chloride-induced apoptosis of HK-2 cells via interfering with mitochondrial respiratory chain[J]. Ecotoxicology and Environmental Safety, 2022, 236:113494-. IF:6.233

21. Obaid QA, Al-Shammari AM, Khudair KK. Glucose Deprivation Induced by Acarbose and Oncolytic Newcastle Disease Virus Promote Metabolic Oxidative Stress and Cell

Death in a Breast Cancer Model. Front Mol Biosci. 2022;9:816510. IF:6.113

22. Abdel-Wahab BA, Walbi IA, Albarqi HA, Ali FEM, Hassanein EHM. Roflumilast protects from cisplatin-induced testicular toxicity in male rats and enhances its cytotoxicity in prostate cancer cell line. Role of NF-κB-p65, cAMP/PKA and Nrf2/HO-1, NQO1 signaling[J]. Food Chem Toxicol. 2021 May;151:112133. IF:6.023
23. Aljutaily T. Evaluating the Nutritional and Immune Potentiating Characteristics of Unfermented and Fermented Turmeric Camel Milk in Cyclophosphamide-Induced Immunosuppression in Rats. Antioxidants (Basel). 2022;11 (4):. IF:5.952
24. Jabbari N, Nawaz M, Rezaie J. Ionizing Radiation Increases the Activity of Exosomal Secretory Pathway in MCF-7 Human Breast Cancer Cells: A Possible Way to Communicate Resistance against Radiotherapy[J]. International Journal of Molecular Sciences, 2019, 20(15):3649-. IF:5.923
25. Shanmugarajan D, Girish C, Harivenkatesh N, et al. Antihypertensive and pleiotropic effects of Phyllanthus emblica extract as an add-on therapy in patients with essential hypertension—A randomized double-blind placebo-controlled trial[J]. Phytotherapy Research, 2021. IF:5.878
26. Marzocco S, Fazeli G, Di Micco L, et al. Supplementation of Short-Chain Fatty Acid, Sodium Propionate, in Patients on Maintenance Hemodialysis: Beneficial Effects on Inflammatory Parameters and Gut-Derived Uremic Toxins, A Pilot Study (PLAN Study)[J]. Journal of Clinical Medicine, 2018. IF:5.688
27. Peng J, Pan J, Mo J, et al. MPO/HOCl Facilitates Apoptosis and Ferroptosis in the SOD1 G93A Motor Neuron of Amyotrophic Lateral Sclerosis. Oxid Med Cell Longev. 2022;2022:8217663. IF:5.604
28. Treadmill Exercise Alleviates Brain Iron Dyshomeostasis Accelerating Neuronal Amyloid-β Production, Neuronal Cell Death, and Cognitive Impairment in Transgenic Mice Model of Alzheimer's Disease[J]. Molecular neurobiology, 2021, 58(7):3208-3223. IF:5.59
29. Aboulhoda, B. E., Rashed, L. A., Ahmed, H., et al. Hydrogen sulfide and mesenchymal stem cells‐extracted microvesicles attenuate LPS‐induced Alzheimer's disease[J]. Journal of Cellular Physiology, 2021, 236(8):5994-6010 IF:5.546
30. Liu W, Jia H, Guan M, et al. Discovery of novel tubulin inhibitors targeting the colchicine binding site via virtual screening, structural optimization and antitumor evaluation. Bioorg Chem. 2022;118:105486. IF:5.508
