# Supplementary material for: Combination decoction of Astragalus mongholicus and Salvia miltiorrhiza mitigates pressure-overload cardiac dysfunction by inhibiting multiple ferroptosis pathways
Source: Front Pharmacol. 2024 Dec 16;15:1447546. doi: 10.3389/fphar.2024.1447546 (PMC11683366; doi:10.3389/fphar.2024.1447546)
Supplement: Supplementary file 4 [file DataSheet1.ZIP › G1340 Masson's Trichrome Stain Kit.pdf]

Masson 三色染色试剂盒

货号：G1340
规格：7×50mL/7×100mL
保存：室温，避光保存，有效期 1 年。

产品组成：

名称		7×50mL	7×100mL	保存
试剂(A):Weigert 铁苏木素染色液	A1:Weigert 染液 A	25mL	50mL	室温，避光
	A2:Weigert 染液 B	25mL	50mL	室温，避光
临用时，取 A1、A2 等量混合配成 Weigert 铁苏木素染色液，建议 4 小时内使用。				
试剂(B): 酸性分化液		50mL	100mL	室温
试剂(C): Masson 蓝化液		50mL	100mL	室温
试剂(D): 丽春红品红染色液		50mL	100mL	室温，避光
试剂(E): 弱酸溶液		50mL	100mL	室温
试剂(F): 磷钼酸溶液		50mL	100mL	室温，避光
试剂(G): 苯胺蓝染色液		50mL	100mL	室温，避光

产品介绍：

结缔组织狭义上是指其含有的三种纤维：胶原纤维、网状纤维、弹力纤维。而胶原纤维是分布最广、含量最多的一种纤维。Masson 三色染色又称马松染色，是结缔组织染色中最经典的一种方法，是胶原纤维染色权威而经典的技术方法。所谓三色染色通常是指染胞核和能选择性的显示胶原纤维和肌纤维。该法染色原理与阴离子染料分子的大小和组织的渗透有关：分子的大小由分子量来体现，小分子量易穿透结构致密、渗透性低的组织，而大分子量则只能进入结构疏松的、渗透性高的组织。然而，淡绿或苯胺蓝的分子量很大，因此 Masson 染色后肌纤维呈红色，胶原纤维呈绿色或蓝色，主要用于区分胶原纤维和肌纤维。

Masson 三色染色试剂盒的特点：◆染色稳定；◆容错率高，能在较大染色时间跨度内取得令人满意的结果；◆色彩清楚鲜艳；◆使用范围广，适宜于组织的石蜡切片、冰冻切片等染色；◆所染切片保存时间长且不易褪色。

自备材料：

G2161-中性福尔马林固定液或 P1110-组织细胞固定液、Bouin 固定液、蒸馏水、系列乙醇、二甲苯或 G2150-环保组织透明脱蜡液、染缸

操作步骤：*(仅供参考)*

一、石蜡切片：

1. 切片推荐厚度 3-8μm，石蜡切片常规脱蜡至蒸馏水。
2. （可选）临用前 1：1 混合试剂 A1 和 A2 配制 Weigert 铁苏木素染色液，滴加覆盖切片染色 5-10min。
3. （可选）蒸馏水洗去多余染色液，滴加酸性乙醇分化液分化 5-15s，蒸馏水洗 30s。
4. （可选）Masson 蓝化液返蓝 3-5min，蒸馏水洗 30s。
5. 丽春红品红染色液染色 5-10min。
6. 在上述操作过程中按蒸馏水：弱酸溶液=2:1 比例配置弱酸工作液，滴加弱酸工作液洗 30s。
7. 倾去多余液体，滴加磷钼酸溶液处理 1-2min。滴加弱酸工作液洗 30s。
8. 倾去多余液体，滴加苯胺蓝染色液染色 1-2min。滴加弱酸工作液洗 30s。（见注意事项 3）
9. 95%乙醇快速脱水 2-3s，无水乙醇脱水 2 次，每次 5-10s。
10. 二甲苯透明 2 次，每次 1-2min，中性树胶封固。

二、冰冻切片

1. 切片推荐厚度 8-12μm，冰冻切片复温复水后用 Bouin 固定液室温过夜处理。
2. 75%乙醇清洗切片至黄色完全褪去。
3. 后续操作同石蜡切片第 2-10 步。（见注意事项 4）

专业生化试剂供应商　　专业分子试剂生产商

染色结果：

细胞核	红褐色到棕褐色
胶原纤维、弹力纤维	蓝色
肌肉纤维、红细胞	红色
细胞浆及其他组织背景	紫红色到红色

注意事项：

1. 切片脱蜡应尽量干净，避免着色不均。本染色原理与结构有关，须避免因组织切片过薄导致的不上色和组织切片过厚导致的整体颜色发暗，因此推荐切片厚度 3-12μm。
2. 组织类型和固定液选择对染色结果有很大影响，因此不同样本染色建议取少量切片进行预实验。
3. 如无 bouin 固定液也可使用 G2310-Carnoy 固定液II代替进行过夜处理，使用 Carnoy 固定液II过夜处理后 75%乙醇浸洗 2 次，每次 1min 后即可正常染色。
4. 冰冻切片由于细胞成分保留较完整，更容易吸附染料，因此建议使用弱酸工作液将苯胺蓝染色液稀释 2-4 倍后染色 0.5-1min，弱酸工作液洗 30s 来避免过度蓝染。
5. 本染液采用 Weigert 铁苏木素染细胞核，与常规苏木素核染色结果不一致属正常现象，因为染色的目的主要在于区分胶原纤维和肌纤维，一般也可以省略该染色步骤。
6. 酸性分化液分化时间应该依据切片薄厚，组织的类别和新旧而定。
7. Masson 蓝化液亦可自行购买 G1865-Scott 促蓝液或配制 0.05-1%碳酸锂水溶液予以替代。
8. 弱酸溶液可使色彩更清晰鲜艳，如使用量大可购买 G2940-弱酸溶液或自行配置 0.5-1%乙酸溶液予以替代。若苯胺蓝过染，可将第 7 步的弱酸清洗步骤改为蒸馏水清洗。
9. 磷钼酸溶液易失效变色，正常呈淡黄色，如使用时已呈黄绿或发蓝建议弃用。
10. 本试剂盒内除试剂 A、F 外试剂均可循环使用，循环次数按照 0.8-1ml/切片计算。
11. 为了您的安全和健康，请穿实验服并戴一次性手套操作。

本产品仅供科研使用。请勿用于医药、临床医疗、食品及化妆品用途。

Masson's Trichrome Stain Kit

Cat: G1340
Size: 7×50mL/7×100mL
Storage: RT, avoid light, valid for 1 year.

Kit Components

Reagent		7×50mL	7×100mL	Storage
Reagent (A):Weigert's Iron Hematoxylin Solution	A1:Weigert's Solution A	25mL	50mL	RT, avoid light
	A2:Weigert's Solution B	25mL	50mL	RT, avoid light
Mix equal parts of A1 and A2 to form Weigert's Iron Hematoxylin Solution,which is stable for about 4 h.				
Reagent (B): Acid Differentiation		50mL	100mL	RT
Reagent (C): Bluing Solution		50mL	100mL	RT
Reagent (D): Ponceau-Acid Fuchsin Solution		50mL	100mL	RT, avoid light
Reagent (E): Weak Acid Solution		50mL	100mL	RT
Reagent (F): Phosphmolybic Acid Solution		50mL	100mL	RT, avoid light
Reagent (G): Aniline Blue Solution		50mL	100mL	RT, avoid light

Introduction

Narrowly speaking, connective tissue refers to the three types of fibers it contains: collagen fibers, reticular fibers, and elastic fibers. Collagen fibers are the most widely distributed and abundant type of fiber. Masson trichrome staining, also known as Masson staining, is the most classic method in connective tissue staining and an authoritative and classic technical method for collagen fiber staining. The so-called trichrome staining usually refers to staining the cell nucleus and selectively displaying collagen and muscle fibers. The dyeing principle of this method is related to the size of anionic dye molecules and the permeability of tissues: the size of molecules is reflected by their molecular weight. Small molecular weights are easy to penetrate tissues with dense structures and low permeability, while large molecular weights can only enter tissues with loose structures and high permeability. However, the molecular weight of light green or aniline blue is large, so after Masson staining, muscle fibers appear red, while collagen fibers appear green or blue, mainly used to distinguish between collagen fibers and muscle fibers.

The characteristics of the Masson's Trichrome Stain Kit include: ◆Stable staining; ◆High fault tolerance, able to achieve satisfactory results within a large dyeing time span; ◆Clear and bright colors; ◆Widely used, suitable for staining tissues such as paraffin sections and frozen sections; ◆The stained slices are stored for a long time and are not easily faded.

Self Prepared Materials

G2161-Neutral Buffered Formalin Fixative, 10% or P1110-Paraformaldehyde,4%, Bouin fixative, distilled water, series ethanol, xylene, or G2150 environmental protection tissue transparent dewaxing solution, dye tank

Protocol(*for reference only*)

For Paraffin Section

1. The recommended tissue thickness is 3-8μm. Dewax to distilled water.
2. (Optional)Before use, mix A1 with A2 in equal amount to prepare Weigert's Iron Hematoxylin Solution, stain with Weigert's Iron Hematoxylin Solution for 5-10mins.
3. (Optional)wash with distilled water to remove excess stain, differentiate with Acid Differentiation for 5-10s, wash with distilled water for 30s.
4. (Optional)Blue in Bluing Solution for 3-5mins. Rinse in distilled water for 30s.
5. Stain with Ponceau-Acid Fucshin Solution for 5-10mins.
6. In the above step, mix distilled water with Weak Acid Solution in 2:1 ratio to prepare Weak Acid Working Solution. Rinse the section with Weak Acid Working Solution for 30s.
7. Discard the excess solution., differentiate in Phosphomolybic Acid Solution for 1-2mins. Rinse the section with Weak Acid Working Solution for 30s.
8. Discard the excess solution, stain with Aniline Blue Solution for 1-2mins. Rinse the section with Weak Acid Working Solution for 30s.(See Note 3)
9. Dehydrate quickly in 95% ethanol for 2-3s, then dehydrate with absolute ethanol twice for each time 5-10s.

专业生化试剂供应商　　专业分子试剂生产商

10. Transparent in xylene for twice, each for 1-2min, seal with resinene.

For Frozen Section

The recommended tissue thickness is 8-12μm. After thawing and rehydrating the frozen sections, treat overnight with Bouin fixative at room temperature.

Wash the slices with 75% ethanol until the yellow color completely fades away.

The subsequent operation is the same as steps 2-10 of paraffin section. (See Note 4)

Result

Nucleus	Reddish Brown to Brown
Collagen fibers and reticular fibers	Blue
Muscle fibers、erythrocytes	Red
Cytoplasm and the background	Purple Red to Red

Note

1. Slice dewaxing should be as clean as possible to avoid uneven color. The principle of this staining is related to the structure, and it is necessary to avoid non coloring caused by too thin tissue slices and overall darkening caused by too thick tissue slices. Therefore, it is recommended to have a slice thickness of 3-12μm.
2. The type of tissue and the choice of fixative have a significant impact on the staining results, so it is recommended to take a small number of sections for pre experiments for staining different samples.
3. If there is no Bouin fixative, G2310-Carnoy fixative II can be used instead for overnight treatment. After overnight treatment with Carnoy fixative II, soak twice in 75% ethanol and each for 1min, then conduct normal staining.
4. Frozen sections are more prone to dye adsorption due to the complete retention of cell components. Therefore, it is recommended to use Weak Acid Working Solution to dilute Aniline Blue Solution 2-4 times and stain for 0.5-1 minutes, followed by washing with Weak Acid Working Solution for 30s to avoid excessive blue staining.
5. This staining solution uses Weigert iron hematoxylin to stain the nucleus, which is a normal phenomenon that is inconsistent with the results of conventional hematoxylin staining. The purpose of staining is mainly to distinguish between collagen fibers and muscle fibers, and this staining step can generally be omitted.
6. The differentiation time of Acid Differentiation Solution should be determined according to the thickness of slice, the type of tissue and the old and new.
7. Masson Bluing Solution can also be replaced by G1865-Scott Bluing Solution or G1840/G1841-Lithium Carbonate Solution, 0.05%/1%.
8. Weak Acid Working Solution can make the color clearer and brighter. If it is used in a large amount, you can buy G2940-Weak Acid Solution or prepare 0.5-1% acetic acid solution to instead of Weak Acid Working Solution. If aniline blue is over dyed, you can replace the step “Rinse the section with Weak Acid Working Solution for 30s” by “Rinse the section with distilled water for 30s”
9. Phosphomolybdic Acid Solution is prone to failure and discoloration, and normally appears light yellow. If it has turned yellow green or blue during use, it is recommended to discard it.
10. Except for Reagent A and F, all reagents in this kit can be recycled for use, with a cycle count of 0.8-1ml for each section.
11. For your safety and health, please wear laboratory clothes and disposable gloves for operation.

本产品仅供科研使用。请勿用于医药、临床医疗、食品及化妆品用途。
