# Supplementary material for: Combination decoction of Astragalus mongholicus and Salvia miltiorrhiza mitigates pressure-overload cardiac dysfunction by inhibiting multiple ferroptosis pathways
Source: Front Pharmacol. 2024 Dec 16;15:1447546. doi: 10.3389/fphar.2024.1447546 (PMC11683366; doi:10.3389/fphar.2024.1447546)
Supplement: Supplementary file 4 [file DataSheet1.ZIP › Rat N-Terminal Pro-Brain Natriuretic Peptide (NT-proBNP) ELISA Kit.pdf]

大鼠 N 端前脑钠素（NT-proBNP）酶联免疫分析(ELISA)
试剂盒使用说明书

本试剂仅供研究使用　　　　目的：本试剂盒用于测定大鼠血清、血浆、组织匀浆及相关液体样本中 N 端前脑钠素（NT-proBNP）的含量。

实验原理：

本试剂盒应用双抗体夹心法测定标本中大鼠 N 端前脑钠素（NT-proBNP）水平。用纯化的大鼠 N 端前脑钠素（NT-proBNP）捕获抗体包被微孔板，制成固相抗体，往包被的微孔中依次加入大鼠 N 端前脑钠素（NT-proBNP），再与 HRP 标记的检测抗体结合，形成抗体-抗原-酶标抗体复合物，经过彻底洗涤后加底物 TMB 显色。TMB 在 HRP 酶的催化下转化成蓝色，并在酸的作用下转化成最终的黄色。颜色的深浅和样品中的大鼠 N 端前脑钠素（NT-proBNP）呈正相关。用酶标仪在 450nm 波长下测定吸光度（OD 值），通过标准曲线计算样品中大鼠 N 端前脑钠素（NT-proBNP）含量。

试剂盒组成：

试剂盒组成	48 孔配置	96 孔配置	保存
说明书	1 份	1 份	
封板膜	2 片	2 片	
密封袋	1 个	1 个	
酶标包被板	1×48	1×96	2-8℃保存
标准品	0.3ml×6 管	0.3ml×6 管	2-8℃保存
酶标试剂	5 ml×1 瓶	10 ml×1 瓶	2-8℃保存
样品稀释液	3 ml×1 瓶	6 ml×1 瓶	2-8℃保存
显色剂 A 液	3 ml×1 瓶	6 ml×1 瓶	2-8℃保存
显色剂 B 液	3 ml×1 瓶	6 ml×1 瓶	2-8℃保存
终止液	3 ml×1 瓶	6 ml×1 瓶	2-8℃保存
20×浓缩洗涤液	15ml×1 瓶	25ml×1 瓶	2-8℃保存

注：标准品浓度依次为：1200、600、300、150、75、0 pg/mL.

样本处理及要求：

1. 血清：室温血液自然凝固 10-20 分钟，离心 20 分钟左右（2000-3000 转/分）。仔细收集上清，保存过程中如出现沉淀，应再次离心。
2. 血浆：应根据标本的要求选择 EDTA 或柠檬酸钠作为抗凝剂，混合 10-20 分钟后，离心 20 分钟左右（2000-3000 转/分）。仔细收集上清，保存过程中如有沉淀形成，应该再次离心。

3. 尿液：用无菌管收集，离心 20 分钟左右（2000-3000 转/分）。仔细收集上清，保存过程中如有沉淀形成，应再次离心。胸腹水、脑脊液参照实行。
4. 细胞培养上清：检测分泌性的成份时，用无菌管收集。离心 20 分钟左右（2000-3000 转/分）。仔细收集上清。检测细胞内的成份时，用 PBS（PH7.2-7.4）稀释细胞悬液，细胞浓度达到 100 万/ml 左右。通过反复冻融，以使细胞破坏并放出细胞内成份。离心 20 分钟左右（2000-3000 转/分）。仔细收集上清。保存过程中如有沉淀形成，应再次离心。
5. 组织标本：切割标本后，称取重量。加入一定量的 PBS，PH7.4。用液氮迅速冷冻保存备用。标本融化后仍然保持 2-8℃的温度。加入一定量的 PBS（PH7.4），用手工或匀浆器将标本匀浆充分。离心 20 分钟左右（2000-3000 转/分）。仔细收集上清。分装后一份待检测，其余冷冻备用。
6. 标本采集后尽早进行提取，提取按相关文献进行，提取后应尽快进行实验。若不能马上进行试验，可将标本放于-20℃保存，但应避免反复冻融.
7. 不能检测含 NaN3 的样品，因 NaN3 抑制辣根过氧化物酶的（HRP）活性。

操作步骤

1. 标准品的加样：设置标准品孔和样本孔，标准品孔各加不同浓度的标准品 50μL；。
2. 加样：分别设空白孔（空白对照孔不加样品及酶标试剂，其余各步操作相同）、待测样品孔。在酶标包被板上待测样品孔中先加样品稀释液 40μl，然后再加待测样品 10μl（样品最终稀释度为 5 倍）。加样将样品加于酶标板孔底部，尽量不触及孔壁，轻轻晃动混匀。
3. 加酶：每孔加入酶标试剂 100μl，空白孔除外。
4. 温育：用封板膜封板后置 37℃温育 60 分钟。
5. 配液：将 20 倍浓缩洗涤液用蒸馏水 20 倍稀释后备用。
6. 洗涤：小心揭掉封板膜，弃去液体，甩干，每孔加满洗涤液，静置 30 秒后弃去，如此重复 5 次，拍干。
7. 显色：每孔先加入显色剂 A50μl，再加入显色剂 B50μl，轻轻震荡混匀，37℃避光显色 15 分钟.
8. 终止：每孔加终止液 50μl，终止反应（此时蓝色立转黄色）。
9. 测定：以空白孔调零，450nm 波长依序测量各孔的吸光度（OD 值）。 测定应在加终止液后 15 分钟以内进行。

注意事项：

1. 试剂盒从冷藏环境中取出应在室温平衡 15-30 分钟后方可使用，酶标包被板开封后如未用完，板条应装入密封袋中保存。样本在使用前也要在室温平衡 60 分钟。
2. 浓洗涤液可能会有结晶析出，稀释时可在水浴中加温助溶，洗涤时不影响结果。
3. 各步加样均应使用加样器，并经常校对其准确性，以避免试验误差。一次加样时间最好控制

在 5 分钟内，如标本数量多，推荐使用排枪加样。

4. 请每次测定的同时做标准曲线，最好做复孔。如标本中待测物质含量过高（样本 OD 值大于标准品孔第一孔的 OD 值），请先用样品稀释液稀释一定倍数（n 倍）后再测定，计算时请最后乘以总稀释倍数（×n×5）。
5. 封板膜只限一次性使用，以避免交叉污染。
6. 底物请避光保存。
7. 严格按照说明书的操作进行，试验结果判定必须以酶标仪读数为准.
8. 所有样品，洗涤液和各种废弃物都应按传染物处理。
9. 本试剂不同批号组分不得混用。
10. 如与英文说明书有异，以英文说明书为准。

计算：

以标准物的浓度为横坐标，OD 值为纵坐标，在坐标纸上绘出标准曲线，根据样品的 OD 值由标准曲线查出相应的浓度；再乘以稀释倍数；或用标准物的浓度与 OD 值计算出标准曲线的直线回归方程式，将样品的 OD 值代入方程式，计算出样品浓度，再乘以稀释倍数，即为样品的实际浓度。

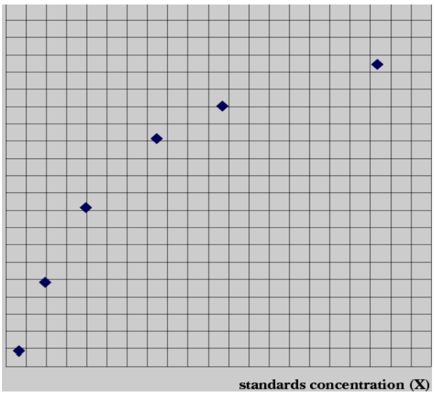

（此图仅供参考）

试剂盒性能：

1.样品线性回归与预期浓度相关系数 R 值为 0.95 以上。

2.批内变异系数与批间变异系数应分别小于 10%和 15% 。

检测范围：

37.5 pg/mL – 1200 pg/mL

灵敏度：

最低检测浓度小于 1.0 pg/mL

保存条件及有效期：

1.试剂盒保存： 2–8℃。

2．有效期： 6 个月

Rat N-terminal pro-brain natriuretic peptide

FOR RESEARCH USE ONLY

Drug Names

Generic Name：**Rat N-terminal pro-brain natriuretic peptide (NT-proBNP) ELISA Kit.**

Purpose

This kit allows for the determination of NT-proBNP concentrations in Rat serum, plasma, tissue homogenates and other biological fluids.

Principle of the assay

The kit assay Rat NT-proBNP level in the sample, use Purified Rat NT-proBNP antibody to coat microtiter plate wells, make solid-phase antibody, then add NT-proBNP to the wells, Combined antibody which With HRP labeled, become antibody-antigen-enzyme-antibody complex, after washing Completely, Add TMB substrate solution,TMB substrate becomes blue color At HRP enzyme-catalyzed, reaction is terminated by the addition of a sulphuric acid solution and the color change is measured spectrophotometrically at a wavelength of 450 nm. The concentration of NT-proBNP in the samples is then determined by comparing the O.D. of the samples to the standard curve.

Materials provided with the kit

Materials provided with the kit	48determinations	96 determinations	Storage
User manual	1	1	
Closure plate membrane	2	2	
Sealed bags	1	1	
Microelisa stripplate	1	1	2-8℃
Standard	0.3ml×6 bottle	0.3ml×6 bottle	2-8℃
HRP-Conjugate reagent	5ml×1 bottle	10ml×1 bottle	2-8℃
Sample diluent	3ml×1 bottle	6ml×1 bottle	2-8℃
Chromogen Solution A	3ml×1 bottle	6ml×1 bottle	2-8℃
Chromogen Solution B	3ml×1 bottle	6ml×1 bottle	2-8℃
Stop Solution	3ml×1 bottle	6ml×1 bottle	2-8℃
20×Wash solution	15ml×1 bottle	25ml×1 bottle	2-8℃

Note: Standard concentration was followed by:

1200、600、300、150、75、0 pg/mL.

Specimen requirements

1. **serum-** coagulation at room temperature 10-20 mins，centrifugation 20-min at the speed of 2000-3000 r.p.m. remove supernatant, If precipitation appeared, Centrifugal again.
2. **plasma-**use suited EDTA or citrate plasma as an anticoagulant,mix 10-20 mins ,centrifugation 20-min at the speed of 2000-3000 r.p.m. remove supernatant, If precipitation appeared, Centrifugal again.
3. **Urine**-collect sue a sterile container, centrifugation 20-min at the speed of 2000-3000 r.p.m. remove supernatant, If precipitation appeared, Centrifugal again. The Operation of Hydrothorax and cerebrospinal fluid Reference to it.
4. **cell culture supernatant**-detect secretory components, collect sue a sterile container, centrifugation 20-min at the speed of 2000-3000 r.p.m. remove supernatant,detect the composition of cells, Dilut cell suspension with PBS（PH7.2-7.4）, Cell concentration reached 1 million / ml, repeated freeze-thaw cycles, damage cells and release of intracellular components, centrifugation 20-min at the speed of 2000-3000 r.p.m. remove supernatant, If precipitation appeared, Centrifugal again.
5. **Tissue samples**- After cutting samples, check the weight,add PBS（PH7.2-7.4）, Rapidly frozen with liquid nitrogen, maintain samples at 2-8℃ after melting,add PBS（PH7.4）, Homogenized by hand or Grinders, centrifugation 20-min at the speed of 2000-3000 r.p.m. remove supernatant.

6. extract as soon as possible after Specimen collection,and according to the relevant literature, and should be experiment as soon as possible after the extraction. If it can't, specimen can be kept in -20 ℃ to preserve, Avoid repeated freeze-thaw cycles.
7. Can't detect the sample which contain NaN3, because NaN3 inhibits HRP active.

Assay procedure

1. Add standard: Set Standard wells, testing sample wells. Add standard 50μl to standard well.

2.add sample： Set blank wells separately (blank comparison wells don't add sample and HRP-Conjugate reagent, other each step operation is same). testing sample well. add Sample dilution 40μl to testing sample well, then add testing sample 10μl (sample final dilution is 5-fold), add sample to wells , don't touch the well wall as far as possible, and Gently mix.

3.add enzyme： Add HRP-Conjugate reagent 100μl to each well, except blank well.

4.Incubate: After closing plate with Closure plate membrane ,incubate for 60 min at 37℃.

5.Configurate liquid: 20-fold wash solution diluted 20-fold with distilled water and reserve.

6.washing： Uncover Closure plate membrane, discard Liquid, dry by swing, add washing buffer to every well, still for 30s then drain, repeat 5 times, dry by pat.

7.color：Add Chromogen Solution A 50ul and Chromogen Solution B to each well, evade the light preservation for 15 min at 37℃

8.Stop the reaction：Add Stop Solution 50μl to each well, Stop the reaction(the blue color change to yellow color).

9.assay：take blank well as zero , Read absorbance at 450nm after Adding Stop Solution and within 15min.

Important notes

1. The kit takes out from the refrigeration environment should be balanced 15-30 minutes in the room temperature, ELISA plates coated if has not use up after opened, the plate should be stored in Sealed bag.
2. washing buffer will Crystallization separation, it can be heated the water helps dissolve when dilute . Washing does not affect the result.
3. add Sample with sampler Each step, And proofread its accuracy frequently, avoids the experimental error. add sample within 5 mins, if the number of sample is much , recommend to use Volley .
4. if the testing material content is excessively higher (The sample OD is bigger than the first standard well),please dilute Sample (n-fold), Please diluente and multiplied by the dilution factor. （×n×5） .
5. Closure plate membrane only limits the disposable use, to avoid cross-contamination.
6. The substrate evade the light preservation**.**
7. Please according to use instruction strictly, The test result determination must take the microtiter plate reader as a standard.
8. All samples, washing buffer and each kind of reject should according to infective material process.
9. Do not mix reagents with those from other lots.

Calculate

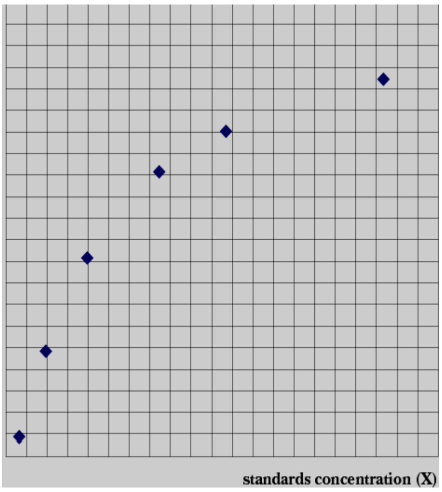

This chartis for reference only

Take the standard density as the horizontal, the OD value for the vertical ,draw the standard curve on graph paper, Find out the corresponding density according to the sample OD value by the Sample curve, multiplied by the dilution multiple, or calculate the straight line regression equation of the standard curve with the standard density and the OD value ,with the sample OD value in the equation, calculate the sample density, multiplied by the dilution factor, the result is the sample actual density.

37.5 pg/mL - 1200 pg/mL

Sensitivity

The minimum detectable dose is typically less than 1.0 pg/mL

Storage and validity

1. Storage： 2-8℃.
2. validity： six months.
