# Supplementary material for: Combination decoction of Astragalus mongholicus and Salvia miltiorrhiza mitigates pressure-overload cardiac dysfunction by inhibiting multiple ferroptosis pathways
Source: Front Pharmacol. 2024 Dec 16;15:1447546. doi: 10.3389/fphar.2024.1447546 (PMC11683366; doi:10.3389/fphar.2024.1447546)
Supplement: Supplementary file 4 [file DataSheet1.ZIP › S0053-GSH and GSSG Assay Kit.pdf]

碧云天 Beyotime

碧云天网站 微信公众号

碧云天生物技术/Beyotime Biotechnology
订货热线：400-168-3301或800-8283301
订货e-mail：order@beyotime.com
技术咨询：info@beyotime.com
网址：http://www.beyotime.com

GSH和GSSG检测试剂盒

产品编号	产品名称	包装
S0053	GSH和GSSG检测试剂盒	共100次

产品简介：

- GSH和GSSG检测试剂盒(GSH and GSSG Assay Kit)是一种简单易行的可以分别检测出GSH(还原型谷胱甘肽)和GSSG(氧化型谷胱甘肽，oxidized glutathione disulfide)含量的检测试剂盒。
- 谷胱甘肽(glutathione)是一种由3个氨基酸残基组成的小肽，全称为谷氨酰-半胱氨酰-甘氨酸，英文名称为glutamyl-cysteinyl-glycine，简称为glutathione。由于半胱氨酸上的巯基(SH)为谷胱甘肽的活性基团，所以常简写为G-SH或GSH。谷胱甘肽包括还原型谷胱甘肽(reduced glutathione, 常称为GSH)和氧化型谷胱甘肽(oxidized glutathione disulfide)两种形式。由于氧化型谷胱甘肽是由两个GSH通过巯基脱氢而成，所以常简写为G-S-S-G或GSSG。还原型谷胱甘肽是绝大多数活细胞中巯基的主要来源，对于维持蛋白质中巯基适当的氧化还原状态有重要作用，并且是动物细胞中关键的抗氧化剂。总谷胱甘肽中通常90-95%为还原型谷胱甘肽。
- 通过谷胱甘肽还原酶把GSSG还原成GSH，而GSH可以和生色底物DTNB反应产生黄色的TNB和GSSG。适当配制反应体系，前后两个反应合并起来后，总谷胱甘肽(GSSG+GSH)就相当于一个颜色产生的限速因素，总谷胱甘肽的量就决定了黄色的TNB形成量。从而通过测定A_{412}就可以计算出总谷胱甘肽的量。用适当试剂先清除样品中的GSH，然后利用上述反应原理就可以测定出GSSG的含量。用总谷胱甘肽(GSSG+GSH)的量扣除GSSG的含量，就可以计算出GSH的含量。
- 本试剂盒的具体反应原理如下：

$$2GSH + DTNB \longrightarrow GSSG + 2TNB$$

$$NADPH + H^{+} + GSSG \xrightarrow{GR} NADP^{+} + 2GSH$$

两个反应相合并：

$$DTNB + H^{+} + NADPH \xrightarrow[GSSG/GSH]{GR} 2TNB + NADP^{+}$$

- 本试剂盒可以检测动物组织、血浆、红细胞、和培养细胞或其它适当样品中GSH和GSSG的含量。
- 本试剂盒提供了蛋白去除试剂M，可以更加准确地测定出含有蛋白的样品中的GSH和GSSG的量。
- 本试剂盒的检测下限为0.5μM。一个试剂盒共可以进行100次检测，可以测定100个样品的总谷胱甘肽或GSSG的含量，或可以测定出50个样品中GSH和GSSG的各自含量。

包装清单：

产品编号	产品名称	包装
S0053-1	总谷胱甘肽检测缓冲液	60ml
S0053-2	谷胱甘肽还原酶	150μl
S0053-3	氧化型谷胱甘肽 (GSSG)	5mg
S0053-4	DTNB	4.5mg
S0053-5	蛋白去除试剂M	1g
S0053-6	NADPH	4mg
S0053-7	DMSO	1.5ml
S0053-8	GSH清除辅助液	2ml
S0053-9	GSH清除试剂	500μl
—	说明书	1份

保存条件：

-20ºC保存，一年有效。GSSG配制成溶液后，需适当分装，-20ºC保存至少3个月有效。DTNB溶解于DMSO后，需适当分装，-20ºC保存至少3个月有效。蛋白去除试剂M配制成溶液后仅限当天使用。NADPH溶解后，适当分装，-70ºC保存。稀释的GSH清除辅助液和GSH清除试剂溶液均须新鲜配制使用。

注意事项：

- 本试剂盒检测时牵涉到氧化还原反应，所有氧化剂或还原剂都会干扰本试剂盒的测定。特别是DTT、巯基乙醇等含有巯基的试剂会严重干扰本试剂盒的测定，请尽量避免。

- 一定要严格控制反应时的温度和反应时间，否则每次都需做标准曲线。
- NADPH等试剂不太稳定，要严格按照后续说明操作，谨防失活。
- 蛋白去除试剂M溶液必须新鲜配制并限当日使用。GSH清除试剂也须新鲜稀释后使用。
- 蛋白去除试剂M较难溶解，可以通过剧烈vortex并适当加热(不超过37ºC)以促进溶解。
- DMSO在4ºC、冰浴等较低温度情况下会凝固，可以20-25ºC水浴温育片刻至全部融解后使用。
- 本产品仅限于专业人员的科学研究用，不得用于临床诊断或治疗，不得用于食品或药品，不得存放于普通住宅内。
- 为了您的安全和健康，请穿实验服并戴一次性手套操作。

使用说明：

1. 试剂盒的准备工作：

a. GSSG储备液的配制：在本试剂盒提供的5mg GSSG中加入816微升Milli-Q级纯水，溶解并混匀，即为GSSG储备液，浓度为10mM。除立即待用部分外，其余GSSG储备液适当分装后-20ºC保存。

b. DTNB储备液的配制：在本试剂盒提供的4.5mg DTNB中加入1.5毫升本试剂盒提供的DMSO，溶解并混匀，即为DTNB储备液。除立即待用部分外，其余DTNB储备液适当分装后-20ºC保存。

c. 蛋白去除试剂M溶液的配制：称取0.2克蛋白去除试剂M，加入4毫升总谷胱甘肽检测缓冲液，配制成4毫升5%的水溶液。蛋白去除试剂M溶液必须新鲜配制并限当天使用。

d. NADPH储备液(40mg/ml)的配制：在本试剂盒提供的4mg NADPH中加入100微升Milli-Q级纯水，溶解并混匀，即为NADPH储备液。除立即待用部分外，其余NADPH储备液适当分装后-70ºC保存。

e. 5倍稀释谷胱甘肽还原酶的配制：取50微升谷胱甘肽还原酶，加入200微升总谷胱甘肽检测缓冲液，混匀，即成5倍稀释的谷胱甘肽还原酶。

f. 总谷胱甘肽检测工作液的配制：根据待检测的样品数参考下表配制适当量的总谷胱甘肽检测工作液，表中三种试剂按比例混合后即为总谷胱甘肽检测工作液。

	1个样品	10个样品	20个样品
5倍稀释谷胱甘肽还原酶	6.6 µl	66 µl	132 µl
DTNB储备液	6.6 µl	66 µl	132 µl
总谷胱甘肽检测缓冲液	150 µl	1.5 ml	3 ml

g. 0.5mg/ml NADPH的配制：取10微升NADPH储备液，加入790微升总谷胱甘肽检测缓冲液，混匀即为0.5mg/ml NADPH。每检测一个样品需50微升0.5mg/ml NADPH。

h. 稀释的GSH清除辅助液的配制：在47微升Milli-Q级纯水中加入53微升GSH清除辅助液，立即混匀。稀释后的GSH清除辅助液不太稳定，每次使用时均须新鲜配制，并仅限当日使用。

i. GSH清除试剂工作液的配制：10.8微升GSH清除试剂中加入89.2微升无水乙醇，立即混匀。GSH清除剂工作液每次也须新鲜配制。

2. 标准品的准备：

a. 把10mM GSSG储备液用蛋白去除试剂M溶液稀释成15µM GSSG溶液。然后依次稀释成10、5、2、1、0.5µM GSSG溶液。取15、10、5、2、1、0.5µM GSSG溶液六个点做标准曲线。注意：由于GSSG在蛋白去除试剂M溶液中不太稳定，用蛋白去除试剂M溶液配制的GSSG溶液必须新鲜配制后使用，不可冻存后再使用。

b. 如果需要测定样品中GSSG含量，按照每100微升标准品加入20微升稀释的GSH清除辅助液的比例加入稀释的GSH清除辅助液，立即vortex混匀。然后按照每100微升加入4微升GSH清除试剂工作液的比例加入GSH清除试剂工作液，立即vortex混匀，25ºC反应60分钟。即可用于后续的GSSG含量的检测。

3. 待测总谷胱甘肽含量样品和标准品的准备：

a. **组织样品的准备。**取组织用液氮速冻，然后研成粉末。每10毫克研碎的组织粉末，加入30微升蛋白去除试剂M溶液，充分Vortex。再加入70微升蛋白去除试剂M溶液，用玻璃匀浆器充分匀浆(对于比较容易匀浆的组织可以不用液氮速冻等处理，而直接加入适量蛋白去除试剂M溶液进行匀浆)。4ºC放置10分钟后，10,000g 4ºC离心10分钟，取上清用于总谷胱甘肽的测定。样品需暂时4ºC保存，不立即测定的样品可以-70ºC保存，但不宜超过10天。对于处理好的组织样品通常需用蛋白去除试剂M溶液进行适当稀释后再进行测定，稀释倍数通常为5-20倍。

b. **细胞样品的准备。**请尽量使用新鲜的细胞进行测定，而不要使用冻存的细胞进行测定。PBS洗涤细胞一次，离心收集细胞，吸尽上清。加入细胞沉淀体积3倍量的蛋白去除试剂M溶液，即如果细胞沉淀为10微升，则加入30微升蛋白去除试剂M溶液，充分Vortex。(细胞沉淀的体积可以根据细胞沉淀的重量进行估算。收集细胞前后分别对离心管进行称重，从而就可以计算出细胞沉淀的重量。10毫克细胞沉淀的体积可以粗略地看做10微升。) 然后利用液氮和37ºC水浴对样品进行两次快速的冻融。4ºC或冰浴放置5分钟。4ºC，10,000g离心10分钟。取上清用于总谷胱甘肽的测定。样品需暂时4ºC保存，不立即测定的样品可以-70ºC保存，但不宜超过10天。对于处理好的细胞样品通常需用蛋白去除试剂M溶液进行适当稀释后再进行测定，稀释倍数可以高达20倍。

c. **红细胞或血浆样品的准备。**请尽量使用新鲜的血液进行测定。600g离心10分钟，沉淀为红细胞，上清为血浆。对于红细胞，用PBS洗涤两次。取约50微升红细胞沉淀或血浆，加入50微升蛋白去除试剂M溶液，充分Vortex。4ºC或冰浴放置10分钟。4ºC，10,000g离心10分钟。取上清用于总谷胱甘肽的测定。样品需暂时4ºC保存，不立即测定的样品可以-70ºC保存，但不宜超过10天。对于处理好的红细胞样品最后需用蛋白去除试剂M溶液稀释10倍后再进行后续的测定，而对于血浆样品，应直接取10微升进行测定。

d. **说明：**对于一些谷胱甘肽含量特别低的样品，可以通过冷冻干燥进行浓缩后再进行测定。

4. **待测GSSG含量样品的准备：**
取部分上述准备好的待测总谷胱甘肽含量的样品，按照每100微升样品加入20微升稀释的GSH清除辅助液的比例加入稀释的GSH清除辅助液，立即vortex混匀。再按照每100微升样品加入4微升GSH清除试剂工作液的比例加入GSH清除工作液，立即vortex混匀，25ºC反应60分钟。通过上述反应可以清除高达50µM的GSH，如果样品中GSH含量过高需进行适当稀释后再进行去除GSH的操作。通过上述处理就可以用于后续的测定。

5. **样品和标准品的测定：**
a. 参考下表，使用96孔板，依次加入样品或标准品，混匀。加入150微升总谷胱甘肽检测工作液后，混匀，25ºC或室温孵育5分钟。

	空白对照 (blank)	标准曲线 (standard)	样品(sample)
样品或标准品	0 µl	10 µl	x µl
蛋白去除试剂M溶液	10 µl	0 µl	10 - x µl
总谷胱甘肽检测工作液	150 µl	150 µl	150 µl
25ºC或室温孵育	5 min	5 min	5 min
0.5mg/ml NADPH	50 µl	50 µl	50 µl

b. 加入50微升0.5mg/ml NADPH溶液，混匀。
c. 立即用酶标仪测定A_{412}，每5分钟测定一次或实时测定，共测定25分钟，测得5个数据。(说明：为了简化实验步骤，可以在加入NADPH溶液混匀后25分钟，仅测定一次A_{412})。如果仪器可以设置温度，把温度设置在25ºC，否则就在室温状况下测定。如果酶标仪不能测定A_{412}，可以测定405-414nm附近范围的吸光度。如果标准曲线良好，但样品的吸光度比较低，可以延长孵育时间至30-60分钟，标准品和样品的吸光度在一定范围内会随时间的延长接近于线性增加的。
注意：如果进行GSSG含量测定，标准品也须平行地进行去除GSH的相关操作，以减小误差。如果样品需同时测定总谷胱甘肽含量和GSSG含量，由于两者的检测体系不同，须分别单独做标准曲线。
d. 标准品的实测效果参考图1。

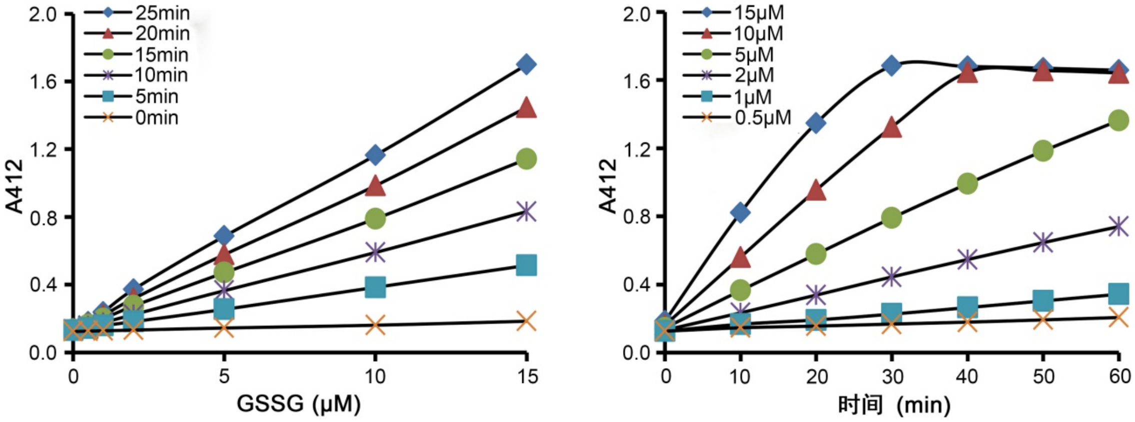

图1. GSSG标准曲线实测效果图。图中数据仅供参考，实际的检测效果可能会因具体反应条件的不同而有所不同。

6. **样品中总谷胱甘肽含量的计算：**
a. **单点测定法：**即反应25分钟(或30-60分钟)后仅测定一次吸光度。根据不同浓度标准品测得的不同吸光度作出标准曲线。样品对照标准曲线即可计算出总谷胱甘肽(标准曲线计算得到的GSSG浓度乘以2)或GSSG的含量。实际计算出来的总谷胱甘肽的含量相当于把氧化型谷胱甘肽的含量乘以2再加上还原型谷胱甘肽的含量。单点法测定相对比较便捷，而动力学法测定则相对比较精确。注意：由于1个GSSG分子反应后可以还原成2个GSH分子，所以GSSG的浓度如果换算成GSH的浓度时需乘以2，例如完全清除样品中内源GSH的情况下，GSSG的浓度为5µM，则相当于GSH的浓度为10µM。
b. **动力学测定法：**先根据不同时间点测定得到的吸光度值计算出ΔA_{412}/min。然后以标准品的浓度为横坐标，以ΔA_{412}/min为纵坐标，做出标准曲线。根据样品的ΔA_{412}/min，对照标准曲线就可以计算出测定时样品中总谷胱甘肽或GSSG的含量。
c. 同时根据样品的稀释倍数以及最初样品的使用量，可以计算出每毫克组织或细胞中的总谷胱甘肽或GSSG的含量。对于细胞样品，也可以根据最初细胞的使用数量，然后另外取一定数量的细胞裂解后测定蛋白浓度，从而计算出细胞样品的蛋白量，最后计算出每毫克蛋白中总谷胱甘肽或GSSG的含量。
d. 根据测定得到的总谷胱甘肽的含量和GSSG的含量就可以计算出GSH的含量。
计算公式为：GSH=Total Glutathione-GSSG×2 (注意：Total Glutathione为通过标准曲线计算得到的GSSG浓度乘以2，同时清除GSH后得到的GSSG也要乘以2，因为1个GSSG分子在反应后可以还原成2个GSH分子)。例如通过本试剂盒测定的总谷胱甘肽(Total Glutathione)的浓度是15µM (即在测定总谷胱甘肽时通过标准曲线计算得到的GSSG浓度为7.5µM，乘以2即为总谷胱甘肽浓度)，测定的GSSG的浓度是1.2µM (即在单独测定GSSG含量时通过标准曲线计算得到的GSSG浓度为1.2µM)，那么样品中GSH的浓度为15-1.2×2=12.6µM。

相关产品：

产品编号	产品名称	包装
S0052	总谷胱甘肽检测试剂盒	100次
S0052B	蛋白去除试剂S	3g
S0053	GSH和GSSG检测试剂盒	共100次
S0054	蛋白去除试剂M	5g
S0055	谷胱甘肽还原酶检测试剂盒	100次
S0056	谷胱甘肽过氧化物酶检测试剂盒	100次
S0058	总谷胱甘肽过氧化物酶检测试剂盒	100次

使用本产品的文献：

1. Jia JH, Wang Y, Cao YB, Gao PH, Jia XM, Ma ZP, Xu YG, Dai BD, Jiang YY. CaIPF7817 is involved in the regulation of redox homeostasis in Candida albicans. Biochem Biophys Res Commun. 2007 Jul 20; 359(1):163-7.
2. Wang B, Feng WY, Zhu MT, Wang Y, Wang M, Gu YQ, Ouyang H, Wang HJ, Li M, Zhao YL, Chai ZF, Wang HF. Neurotoxicity of low-dose repeatedly intranasal instillation of nano- and submicron-sized ferric oxide particles in mice. J Nanopart Res. 2007 Sep;118(3):233-43.
3. Ji LL, Chen Y, Wang ZT. Yamaoka Ligularia base of HEK293 cells glutathione content and the impact of related activity. J Toxicol. 2007 Jun; 21(3):205-7.
4. Zhu MT, Feng WY, Wang B, Wang TC, Gu YQ, Wang M, Wang Y, Ouyang H, Zhao YL, Chai ZF. Comparative study of pulmonary responses to nano- and submicron-sized ferric oxide in rats. Toxicology. 2008 May 21;247(2-3):102-11.
5. Bi J, Jiang B, Liu JH, Zhang XL, An LJ. Protective effects of catalpol against H(2)O(2)-induced oxidative stress in astrocytes primary cultures. Neurosci Lett. 2008 Sep 19;442(3):224-7.
6. Fan J, Cai H, Yang S, Yan L, Tan W. Comparison between the effects of normoxia and hypoxia on antioxidant enzymes and glutathione redox state in ex vivo culture of CD34(+) cells. Comp Biochem Physiol B Biochem Mol Biol. 2008 Oct;151(2):153-8.
7. Wang L, Yan B, Liu N, Li Y, Wang Q. Effects of cadmium on glutathione synthesis in hepatopancreas of freshwater crab, Sinopotamon yangtsekiense. Chemosphere. 2008 Dec;74(1):51-6.
8. Zhang P, Xua Y, Li L, Jiang Q, Wang M, Jin L. In vitro protective effects of pyrroloquinoline quinone on methylmercury-induced neurotoxicity. Environmental Toxicology and Pharmacology. 2009 Jan;27(1):103-10.
9. Liu Z, Zhang X, Bai J, Suo B, Xu P, Wang L. Exogenous paraquat changes antioxidant enzyme activities and lipid peroxidation in drought-stressed cucumber leaves. Scientia Horticulturae. 2009; 121:138-43.
10. Dong HS, Li L, Song ZH, Tang J, Xu B, Zhai XW, Sun LL, Zhang P, Li ZB, Pan QJ, Shi QH, Shen W. Premeiotic fetal murine germ cells cultured in vitro form typical oocyte-like cells but do not progress through meiosis. Theriogenology. 2009 Jul 15;72(2):219-31.
11. LIU JJ, LIN SH, XU PL, WANG XJ, BAI JG. Effects of Exogenous Silicon on the Activities of Antioxidant Enzymes and Lipid Peroxidation in Chilling-Stressed Cucumber Leaves. Agricultural Sciences in China. 2009;8(9):1075-86.
12. Sun ZW, Zhang L, Zhu SJ, Chen WC, Mei B. Excitotoxicity effects of glutamate on human neuroblastoma SH-SY5Y cells via oxidative damage. Neurosci Bull. 2010;26(1):8-16.
13. Xu JY, Su YY, Cheng JS, Li SX, Liu RL, Li WX, Xu GT, Li QN. Protective effects of fullerenol on carbon tetrachloride-induced acute hepatotoxicity and nephrotoxicity in rats. Sciencedirect Carbon.2010 Apr;48(5):1388-96.
14. Cao X, Xiao H, Zhang Y, Zou L, Chu Y, Chu X. 1, 5-Dicaffeoylquinic acid-mediated glutathione synthesis through activation of Nrf2 protects against OGD/reperfusion-induced oxidative stress in astrocytes. Brain Res. 2010;1347:142-8.
15. Sun L, Xu S, Zhou M, Wang C, Wu Y, Chan P. Effects of cysteamine on MPTP-induced dopaminergic neurodegeneration in mice. Brain Res. 2010;1335:74-82.
16. Huang JZ, Chen YZ, Su M, Zheng HF, Yang YP, Chen J, Liu CF. dl-3-n-Butylphthalide prevents oxidative damage and reduces mitochondrial dysfunction in an MPP(+)-induced cellular model of Parkinson's disease. Neurosci Lett. 2010;475(2):89-94.
17. Liu ZJ, Guo YK, Bai JG. Exogenous hydrogen peroxide changes antioxidant enzyme activity and protects ultrastructure in leaves of two cucumber ecotypes under osmotic stress. JOURNAL OF PLANT GROWTH REGULATION.2010;29(7):171-83.
18. Yang CR, Miao DQ, Zhang QH, Guo L, Tong JS, Wei Y, Huang X, Hou Y, Schatten H, Liu Z, Sun QY. Short-term preservation of porcine oocytes in ambient temperature: novel approaches. PLoS One. 2010 Dec 7;5(12):e14242.
19. Wang W, Feng MF, Lin Z. The Glutathione and Metallothionein Contents Variation in Earthworm Exposed by AHTN. Bioinformatics and Biomedical Engineering (iCBBE). 2010 Jun 18-20;1-4.
20. Liu J, Zhou J, An W, Lin Y, Yang Y, Zang W. Apocynin attenuates pressure overload-induced cardiac hypertrophy in rats by reducing levels ofreactive oxygen species. Can J Physiol Pharmacol. 2010 Jul; 88(7):745-52.
21. Ding B, Yuan L, Yu H, Li L, Ma W, Bi Y, Feng J, Xiao R. Genistein and folic acid prevent oxidative injury induced by β-amyloid peptide. Basic Clin Pharmacol Toxicol. 2011 May;108(5):333-40.
22. Zhang J, Zhao QZ, Duan GL, Huang YC. Influence of sulphur on arsenic accumulation and metabolism in rice seedlings. Environmental andExperimentalBotany. 2011 Aug;72(1):34-40.
23. Zhang XL,Jia XF, Yu B, Gao Y, Bai JG. Exogenous hydrogen peroxide influences antioxidant enzyme activity and lipid peroxidation in cucumber leaves at low light. Scientia Horticulturae. 2011 Jul; 129(4):656-2.
24. Li Q, Yu B, Gao Y, Dai AH, Bai JG. Cinnamic acid pretreatment mitigates chilling stress of cucumber leaves through alteringantioxidant enzyme activity. J Plant Physiol. 2011 Jun 15;168(9):927-34.
25. Hu L, Chen L, Yang G, Li L, Sun H, Chang Y, Tu Q, Wu M, Wang H. HBx sensitizes cells to oxidative stress-induced apoptosis by accelerating the loss of Mcl-1 proteinvia caspase-3 cascade. Mol Cancer. 2011 Apr 20;10:43.
26. Sun WJ, Nie YX, Gao Y, Dai AH, Bai JG. Exogenous cinnamic acid regulates antioxidant enzyme activity and reduces lipid peroxidation in drought-stressed cucumber leaves. ACTA PHYSIOLOGIAE PLANTARUM. 2012;34(2):641-55.
27. Jia P, Xu YJ, Zhang ZL, Li K, Li B, Zhang W, Yang H. Ferric ion could facilitate osteoclast differentiation and bone resorption through the production ofreactive oxygen species. J Orthop Res. 2012 Nov; 30(11):1843-52.
28. Wang J, Hou Y, Dong L, Niu X, Fan Y. Influence of TiO2 nanoparticles on glutathione in rat synovial cell line RSC-364. World Congress on Medical Physics and Biomedical Engineering. 2012 May 26-31; 39:75-8.
29. Zhong WX, Wang YB, Peng L, Ge XZ, Zhang J, Liu SS, Zhang XN, Xu ZH, Chen Z, Luo JH. Lanthionine synthetase C-like protein 1 interacts with and inhibits cystathionine β-synthase: a target for neuronal antioxidant defense. J Biol Chem. 2012 Oct 5;287(41):34189-201.
30. Zhang B, Chen N, Chen H, Wang Z, Zheng Q. The critical role of redox homeostasis in shikonin-induced HL-60 cell differentiation via unique modulation of theNrf2/ARE pathway. Oxid Med Cell Longev. 2012; 2012:781516.
31. Zhang J, Li D, Sun W, Wang X, Bai J. Exogenous p-hydroxybenzoic acid regulates antioxidant enzyme activity and mitigates heat stress of cucumber leaves. Scientia Horticulturae. 2012 Dec 4; 148:235-45.
32. Dai A, Nie Y, Yu B, Li Q, Lu L,Bai J. Cinnamic acid pretreatment enhances heat tolerance of cucumber leaves through modulating antioxidant enzyme activity. Environmental and Experimental Botany. 2012 Jul; 79:1-10.
33. Zhang L, Zhang J, Zhao B, Zhao-Wilson X. Quinic acid could be a

potential rejuvenating natural compound by improving survival of Caenorhabditis elegansunder deleterious conditions. Rejuvenation Res. 2012 Dec;15(6):573-83.

34. Liu L, Wang P, Liang C, He D, Yu Y, Liu X. Distinct effects of Nampt inhibition on mild and severe models of lipopolysaccharide-induced myocardialimpairment. Int Immunopharmacol. 2013 Oct;17(2):342-9.
35. Ma WW, Hou CC, Zhou X, Yu HL, Xi YD, Ding J, Zhao X, Xiao R. Genistein alleviates the mitochondria-targeted DNA damage induced by β-amyloid peptides 25-35 in C6 gliomacells. Neurochem Res. 2013 Jul;38(7):1315-23.
36. Wang J, Jiang Z, Ji J, Wang X, Wang T, Zhang Y, Tai T, Chen M, Sun L, Li X, Zhang L. Gene expression profiling and pathway analysis of hepatotoxicity induced by triptolide in Wistar rats. Food Chem Toxicol. 2013 Apr 29;58C:495-505.
37. Ren C, Li Y, Han R, Gao D, Li W, Shi J, Hoogewijs D, Braeckman BP, De Henau S, Lu Y, Qu W, Gao Y, Wu Y, Li Z, Liu H, Wang Z, Zhang C. GLB-13 is associated with oxidative stress resistance in caenorhabditis elegans. IUBMB Life. 2013 May;65(5):423-34.
38. Dai BD, Wang Y, Zhao LX, Li DD, Li MB, Cao YB, Jiang YY. Cap1p attenuates the apoptosis of Candida albicans. FEBS J. 2013 Jun; 280(11):2633-43.
39. Khan M, Li T, Ahmad Khan MK, Rasul A, Nawaz F, Sun M, Zheng Y, Ma T. Alantolactone induces apoptosis in HepG2 cells through GSH depletion, inhibition of STAT3 activation, andmitochondrial dysfunction. Biomed Res Int. 2013;2013:719858.
40. Jiang Q, Zhou Z, Wang L, Shi X, Wang J, Yue F, Yi Q, Yang C, Song L. The immunomodulation of inducible nitric oxide in scallop Chlamys farreri. Fish Shellfish Immunol. 2013 Jan;34(1):100-8.
41. Zhang L, Wang L, Hu Y, Liu Z, Tian Y, Wu X, Zhao Y, Tang H, Chen C, Wang Y. Selective metabolic effects of gold nanorods on normal and cancer cells and their application in anticancer drugscreening. Biomaterials. 2013 Sep;34(29):7117-26.
42. Jing C, Li X, Zhang J, Wang J. Responses of the Antioxidant System in QGY-7701 Cells to the Cytotoxicity and Apoptosis Induced by 1-Octyl-3-methylimidazolium Chloride. J Biochem Mol Toxicol. 2013 Jun;27(6):330-6.
43. Hou Y, Lai M, Chen X, Li J, Hu Y, Luo Z, Ding X, Cai K. Effects of mesoporous SiO2 , Fe3 O4 , and TiO2 nanoparticles on the biological functions of endothelial cells in vitro. J Biomed Mater Res A. 2014 Jun;102(6):1726-36.
44. Wang C, Chen H, Zhang J, Hong Y, Ding X, Ying W. Malate-aspartate shuttle mediates the intracellular ATP levels, antioxidation capacity and survival of differentiated PC12 cells. Int J Physiol Pathophysiol Pharmacol. 2014 Jul 12;6(2):109-14.
45. Meng G, Xia M, Xu C, Yuan D, Schnurr M, Wei J. Multifunctional antitumor molecule 5'-triphosphate siRNA combining glutaminase silencing and RIG-I activation. Int J Cancer. 2014 Apr 15; 134(8):1958-71.
46. Mo C, Wang L, Zhang J, Numazawa S, Tang H, Tang X, Han X, Li J, Yang M, Wang Z, Wei D, Xiao H. The crosstalk between Nrf2 and AMPK signal pathways is important for the anti-inflammatory effect of berberine in LPS-stimulated macrophages and endotoxin-shocked mice. Antioxid Redox Signal. 2014 Feb 1;20(4):574-88.
47. Xie JM, Li B, Yu HP, Gao QG, Li W, Wu HR, Qin ZH. TIGAR has a dual role in cancer cell survival through regulating apoptosis and autophagy. Cancer Res. 2014 Sep 15;74(18):5127-38.
48. He X, Song W, Liu C, Chen S, Hua J. Rapamycin inhibits acrolein-induced apoptosis by alleviating ROS-driven mitochondrial dysfunction in male germ cells. Cell Prolif. 2014 Apr;47(2):161-71.
49. Xu W, Wang S, Chen Q, Zhang Y, Ni P, Wu X, Zhang J, Qiang F, Li A, Røe OD, Xu S, Wang M, Zhang R, Zhou J. TXNL1-XRCC1 pathway regulates cisplatin-induced cell death and contributes to resistance in human gastric cancer. Cell Death Dis. 2014 Feb 13;5:e1055.
50. Zhou H, Wang X, Zhou Y, Yao H, Ahmad F. Evaluation of the toxicity of ZnO nanoparticles to Chlorella vulgaris by use of the chiral perturbation approach. Anal Bioanal Chem. 2014 Jun;406(15):3689-95.
51. Wang Y, Zhao W, Hao J, Xu W, Luo Y, Wu W, Yang Z, Liang Z, Huang K. Changes in biosynthesis and metabolism of glutathione upon ochratoxin A stress in Arabidopsis thaliana. Plant Physiol Biochem. 2014 Jun;79:10-8.
52. Zhang B, Wang XQ, Chen HY, Liu BH. Involvement of the Nrf2 Pathway in the Regulation of Pterostilbene-Induced Apoptosis in HeLa Cells via ER Stress. J Pharmacol Sci. 2014 Nov 20;126(3):216-229.
53. Diao Y, Xu H, Li G, Yu A, Yu X, Hu W, Zheng X, Li S, Wang Y, Hu Z. Cloning a glutathione peroxidase gene from Nelumbo nucifera and enhanced salt tolerance by overexpressing in rice. Mol Biol Rep. 2014 Aug;41(8):4919-27.
54. Shi H, Jiang C, Ye T, Tan D, Reiter RJ, Zhang H, Liu R, Chan Z. Comparative physiological, metabolomic, and transcriptomic analyses reveal mechanisms of improved abiotic stress resistance in bermudagrass [Cynodon dactylon (L). Pers.] by exogenous melatonin. J Exp Bot.2015 Feb;66(3):681-94.
55. Shi H, Ye T, Han N, Bian H, Liu X, Chan Z. Hydrogen sulfide regulates abiotic stress tolerance and biotic stress resistance in Arabidopsis. J Integr Plant Biol. 2015 Jul;57(7):628-40.
56. Zhang C, Zhou Z, Zhi X, Ma Y, Wang K, Wang Y, Zhang Y, Fu H, Jin W, Pan F, Cui D. Insights into the distinguishing stress-induced cytotoxicity of chiral gold nanoclusters and the relationship with GSTP1. Theranostics. 2015 Jan 1;5(2):134-49.
57. Qiu M, Chen L, Tan G, Ke L, Zhang S, Chen H, Liu J. A reactive oxygen species activation mechanism contributes to JS-K-induced apoptosis in human bladder cancer cells. Sci Rep. 2015 Oct 13;5:15104.
58. Ma YC, Su N, Shi XJ, Zhao W, Ke Y, Zi X, Zhao NM, Qin YH, Zhao HW, Liu HM. Jaridonin-induced G2/M phase arrest in human esophageal cancer cells is caused by reactive oxygen species-dependent Cdc2-tyr15 phosphorylation via ATM-Chk1/2-Cdc25C pathway. Toxicol Appl Pharmacol. 2015 Jan 15;282(2):227-36.
59. Shi H, Jiang C, Ye T, Tan DX, Reiter RJ, Zhang H, Liu R, Chan Z. Comparative physiological, metabolomic, and transcriptomic analyses reveal mechanisms of improved abiotic stress resistance in bermudagrass [Cynodon dactylon (L). Pers.] by exogenous melatonin. J Exp Bot. 2015 Feb;66(3):681-94.
60. She T, Qu L, Wang L, Yang X, Xu S, Feng J, Gao Y, Zhao C, Han Y, Cai S, Shou C. Sarsaparilla (Smilax Glabra Rhizome) Extract Inhibits Cancer Cell Growth by S Phase Arrest, Apoptosis, and Autophagy via Redox-Dependent ERK1/2 Pathway. Cancer Prev Res (Phila). 2015 May; 8(5):464-74.
61. Liu H, Li Q, Cheng X, Wang H, Wang G, Hao H. UDP-glucuronosyltransferase 1A determinates intracellular accumulation and anti-cancer effect of β-lapachone in human colon cancer cells. PLoS One. 2015 Feb 18;10(2):e0117051.
62. Jiang F, Chen L, Yang YC, Wang XM, Wang RY, Li L, Wen W, Chang YX, Chen CY, Tang J, Liu GM, Huang WT, Xu L, Wang HY. CYP3A5 Functions as a Tumor Suppressor in Hepatocellular Carcinoma by Regulating mTORC2/Akt Signaling. Cancer Res. 2015 Apr 1; 75(7):1470-81.
63. Xu Y, Zhou X, Shi C, Wang J, Wu Z. α-Lipoic acid protects against the oxidative stress and cytotoxicity induced by cadmium in HepG2 cells through regenerating glutathione regulated by glutamate-cysteine ligase. Toxicol Mech Methods. 2015 Oct;25(8):596-603.
64. Wang G, Wang H, Xiong X, Chen S, Zhang D. Mitochondria thioredoxin's backup role in oxidative stress resistance in Trichoderma reesei. Microbiol Res. 2015 Feb;171:32-8.
65. Du Z, Jia XL, Wang Y, Wu T, Han ZH, Zhang XZ. Redox homeostasis and reactive oxygen species scavengers shift during ontogenetic phase changes in apple. Plant Sci. 2015 Jul;236:283-94.
66. Zheng L, Meng Y, Ma J, Zhao X, Cheng T, Ji J, Chang E, Meng C, Deng N, Chen L, Shi S, Jiang Z. Transcriptomic analysis reveals importance of ROS and phytohormones in response to short-term salinity stress in Populus tomentosa.Front Plant Sci. 2015 Sep 15;6:678.
67. Zheng CP, Han L, Hou WJ, Tang J, Wen YH, Fu R, Wang YJ, Wen WP. MicroRNA-9 suppresses the sensitivity of CNE2 cells to ultraviolet radiation. Mol Med Rep. 2015 Aug;12(2):2367-73.
68. Liu X, Fu Z, Wu Y, Hu X Jr, Zhu T Jr, Jin C Jr. Neuroprotective effect of hydrogen sulfide on acute cauda equina injury in rats. Spine J. 2016 Mar;16(3):402-7.
69. Guo L, Lv G, Qiu L, Yang H, Zhang L, Yu H, Zou M, Lin J. Insights into anticancer activity and mechanism of action of a ruthenium(II) complex in human esophagealsquamous carcinoma EC109 cells. Eur J Pharmacol. 2016 Sep 5;786:60-71.
70. Liu Z, Gan L, Chen Y, Luo D, Zhang Z, Cao W, Zhou Z, Lin X, Sun C. Mark4 promotes oxidative stress and inflammation via binding to PPARγ and activating NF-κB pathway inmice adipocytes. Sci Rep. 2016 Feb 18;6:21382.
71. Zhou Q, Chen B, Wang X, Wu L, Yang Y, Cheng X, Hu Z, Cai X, Yang J, Sun X, Lu W, Yan H, Chen J, Ye J, Shen J, Cao P. Sulforaphane protects

against rotenone-induced neurotoxicity in vivo: Involvement of the mTOR, Nrf2, andautophagy pathways. Sci Rep. 2016 Aug 24;6:32206.

72. Liang W, Cai A, Chen G, Xi H, Wu X, Cui J, Zhang K, Zhao X, Yu J, Wei B, Chen L. Shikonin induces mitochondria-mediated apoptosis and enhances chemotherapeutic sensitivity of gastriccancer through reactive oxygen species. Sci Rep. 2016 Dec 1;6:38267.

73. Lu MC, Ji JA, Jiang YL, Chen ZY, Yuan ZW, You QD, Jiang ZY. An inhibitor of the Keap1-Nrf2 protein-protein interaction protects NCM460 colonic cells and alleviatesexperimental colitis. Sci Rep. 2016 May 24;6:26585.

74. Guo H, Zhang N, Liu D, Wang P, Ma X. Inhibitory effect on the proliferation of human heptoma induced by cell-permeable manganese superoxidedismutase. Biomed Pharmacother. 2016 Oct;83:1379-1386.

75. Liu Q, Sun Y, Lv Y, Le Z, Xin Y, Zhang P, Liu Y. TERT alleviates irradiation-induced late rectal injury by reducing hypoxia-induced ROS levels through theactivation of NF-κB and autophagy. Int J Mol Med. 2016 Sep;38(3):785-93.

76. Yuan-Jing F, Wei W, Jian-Ping L, Yu-Xia J, Zi-Ling D. Genistein promotes the metabolic transformation of acetaminophen to glucuronic acid in human L-O2,HepG2 and Hep3b cells via the Nrf2/Keap1 pathway. Food Funct. 2016 Nov 9;7(11):4683-4692.

77. Deng C, Zhang B, Zhang S, Duan C, Cao Y, Kang W, Yan H, Ding X, Zhou F, Wu L, Duan G, Shen S, Xu G, Zhang W, Chen M, Huang S,Zhang X, Lv Y, Ling T, Wang L, Zou X. Low nanomolar concentrations of Cucurbitacin-I induces G2/M phase arrest and apoptosis by perturbingredox homeostasis in gastric cancer cells in vitro and in vivo. Cell Death Dis. 2016 Feb 18;7:e2106.

78. Wang X, Liang Z, Hou J, Bao X, Shen Y. Identification and functional evaluation of the reductases and dehydrogenases from Saccharomyces cerevisiae involved in vanillin resistance. BMC Biotechnol. 2016 Apr 1;16:31.

79. Zhang J, Cao M, Yang W, Sun F, Xu C, Yin L, Pu Y. Inhibition of Glucos e-6-Phosphate Dehydrogenase Could Enhance 1,4-Benzoquinone-Induce d OxidativeDamage in K562 Cells. Oxid Med Cell Longev. 2016;2016:3 912515.

80. Fang Y, Wang J, Xu L, Cao Y, Xu F, Yan L, Nie M, Yuan N, Zhang S, Zhao R, Wang H, Wu M, Zhang X, Wang J. Autophagy maintains ubiquitination-proteasomal degradation of Sirt3 to limit oxidative stress in K562leukemia cells. Oncotarget. 2016 Jun 14;7(24):35692-35702.

81. Tao J, Shen X, Ai Y, Han X. Tea polyphenols protect against ischemia/reperfusion-induced liver injury in mice through anti-oxidativeand anti-apoptotic properties. Exp Ther Med. 2016 Nov; 12(5):3433-3439.

82. Wang D, Meng G, Zheng M, Zhang Y, Chen A, Wu J, Wei J. The Glutaminase-1 Inhibitor 968 Enhances Dihydroartemisinin-Mediated Antitumor Efficacy in HepatocellularCarcinoma Cells. PLoS One. 2016 Nov 11;11(11):e0166423.

83. Fan XY, Chen XY, Liu YJ, Zhong HM, Jiang FL, Liu Y. Oxidative stress-mediated intrinsic apoptosis in human promyelocytic leukemia HL-60 cells induced byorganic arsenicals. Sci Rep. 2016 Jul 19;6:29865.

84. Xu Y, Zhu J, Hu X, Wang C, Lu D, Gong C, Yang J, Zong L. CLIC1 Inhibition Attenuates Vascular Inflammation, Oxidative Stress, and Endothelial Injury. PLoS One. 2016 Nov 18;11(11):e0166790.

85. Deng C, Zhang B, Zhang S, Duan C, Cao Y, Kang W, Yan H, Ding X, Zhou F, Wu L, Duan G, Shen S, Xu G, Zhang W, Chen M, Huang S, Zhang X, Lv Y, Ling T, Wang L, Zou X. Low nanomolar concentrations of Cucurbitacin-I induces G2/M phase arrest and apoptosis by perturbing redox homeostasis in gastric cancer cells in vitro and in vivo. Cell Death Dis. 2016 Feb 18;7:e2106.

86. Li X, Fan XX, Jiang ZB, Loo WT, Yao XJ, Leung EL, Chow LW, Liu L. Shikonin inhibits gefitinib-resistant non-small cell lung cancer by inhibiting TrxR and activating the EGFRproteasomal degradation pathway. Pharmacol Res. 2017 Jan;115:45-55.

87. Sun X, Wang W, Chen J, Cai X, Yang J, Yang Y, Yan H, Cheng X, Ye J, Lu W, Hu C, Sun H, Pu J, Cao P. The Natural Diterpenoid Isoforretin A Inhibits Thioredoxin-1 and Triggers Potent ROS-Mediated Antitumor Effects. Cancer Res. 2017 Feb 15;77(4):926-936.

88. Wang Y, Gu YH, Liu M, Bai Y, Wang HL. Fluoxetine protects against methamphetamine induced lung inflammation by suppressing oxidative stressthrough the SERT/p38 MAPK/Nrf2 pathway in rats. Mol Med Rep. 2017 Feb;15(2):673-680.

89. Qiu M, Chen L, Tan G, Ke L, Zhang S, Chen H, Liu J. JS-K promotes apoptosis by inducing ROS production in human prostate cancer cells. Oncol Lett. 2017 Mar;13(3):1137-1142.

90. He JH, Yan M, Zuo H, Niu S, Yuan J, Weng SP, He J, Xu X. High reduced/oxidized glutathione ratio in infectious spleen and kidney necrosis virus-infectedcells contributes to degradation of VP08R multimers. Vet Microbiol. 2017 Aug;207:19-24.

91. Chen H, Tang X, Zhou B, Zhou Z, Xu N, Wang Y. A ROS-mediated mitochondrial pathway and Nrf2 pathway activation are involved in BDE-47induced apoptosis in Neuro-2a cells. Chemosphere. 2017 Oct;184:679-686.

92. Qiu M, Ke L, Zhang S, Zeng X, Fang Z, Liu J. JS-K,a GST-activated nitric oxide donor prodrug, enhances chemo-sensitivity in renal carcinomacells and prevents cardiac myocytes toxicity induced by Doxorubicin. Cancer Chemother Pharmacol. 2017 Aug;80(2):275-286.

93. Feng RB, Wang Y, He C, Yang Y, Wan JB. Gallic acid,a natural polyphenol, protects against tert-butyl hydroperoxide-induced hepatotoxicity by activating ERK-Nrf2-Keap1-mediated antioxidative response. Food Chem Toxicol. 2017 Oct 21. pii: S0278-6915(17)30631-2.

94. He X, Wu C, Cui Y, Zhu H, Gao Z, Li B, Hua J, Zhao B. The aldehyde group of gossypol induces mitochondrial apoptosis via ROS-SIRT1-p53-PUMApathway in male germline stem cell. Oncotarget. 2017 Oct 24;8(59):100128-100140.

95. Wang XY, Li YL, Wang HY, Zhu M, Guo D, Wang GL, Gao YT, Yang Z, Li T, Yang CY, Chen YM. Propofol inhibits invasion and proliferation of C6 glioma cells by regulating the Ca2+ permeableAMPA receptor-system xc- pathway. Toxicol In Vitro. 2017 Oct;44:57-65.

96. Sun X, Wang W, Chen J, Cai X, Yang J, Yang Y, Yan H, Cheng X, Ye J, Lu W, Hu C, Sun H, Pu J, Cao P. The Natural Diterpenoid Isoforretin A Inhibits Thioredoxin-1 and Triggers Potent ROS-MediatedAntitumor Effects. Cancer Res. 2017 Feb 15;77(4):926-936.

97. Meng W, Pei Z, Feng Y, Zhao J, Chen Y, Shi W, Xu Q, Lin F, Sun M, Xiao K. Neglected role of hydrogen sulfide in sulfur mustard poisoning: Keap1 S-sulfhydration and subsequent Nrf2 pathway activation. Sci Rep. 2017 Aug 25;7(1):9433.

98. Huang R, Li X, Yu Y, Ma L, Liu S, Zong X, Zheng Q. SETD7 isa prognosis predicting factor of breast cancer and regulates redox homeostasis. Oncotarget. 2017 Oct 6;8(55):94080-94090.

99. Li X, Fan XX, Jiang ZB, Loo WT, Yao XJ, Leung EL, Chow LW, Liu L. Shikonin inhibits gefitinib-resistant non-small cell lung cancer by inhibiting TrxR and activating the EGFR proteasomal degradation pathway. Pharmacol Res. 2017 Jan;115:45-55.

100. Wang Y, Gu YH, Liu M, Bai Y, Wang HL. Fluoxetine protects against methamphetamine induced lung inflammation by suppressing oxidative stress through the SERT/p38 MAPK/Nrf2 pathway in rats. Mol Med Rep. 2017 Feb;15(2):673-680.

101. Zhang XL, Yuan YH, Shao QH, Wang ZZ, Zhu CG, Shi JG, Ma KL, Yan X, Chen NH. DJ-1 regulating PI3K-Nrf2 signaling plays a significant role in bibenzyl compound 20C-mediatedneuroprotection against rotenone-induced oxidative insult. Toxicol Lett. 2017 Apr 5;271:74-83.

102. Xu X, Xia L, Chen W, Huang Q. Detoxification of hexavalent chromate by growing Paecilomyces lilacinus XLA. Environ Pollut. 2017 Jun;225:47-54.

103. Shi H, Li Y, Ren X, Zhang Y, Yang Z, Qi C. A novel quinazoline-based analog induces G2/M cell cycle arrest and apoptosis in human A549lung cancer cells via a ROS-dependent mechanism. Biochem Biophys Res Commun. 2017 Apr 29;486(2):314-320.

104. Zhang GM, Deng MT, Lei ZH, Wan YJ, Nie HT, Wang ZY, Fan YX, Wang F, Zhang YL. Effects of NRF1 on steroidogenesis and apoptosis in goat luteinized granulosa cells. Reproduction. 2017 Aug;154(2):111-122.

105. Dai JP, Wang QW, Su Y, Gu LM, Zhao Y, Chen XX, Chen C, Li WZ, Wang GF, Li KS. Emodin Inhibition of Influenza A Virus Replication and Influenza Viral Pneumonia via the Nrf2, TLR4, p38/JNK and NF-kappaB Pathways. Molecules. 2017 Oct 18;22(10). pii: E1754.

106. Zhao J, Fu B, Peng W, Mao T, Wu H, Zhang Y. Melatonin protect the development of preimplantation mouse embryos from sodium fluoride-induced oxidative injury. Environ Toxicol Pharmacol. 2017 Sep;54:133-141.

107. Xie F, Wu D, Huang SF, Cao JG, Li HN, He L, Liu MQ, Li LF, Chen LX. The endoplasmic reticulum stress-autophagy pathway is involved in apelin-13-inducedcardiomyocyte hypertrophy in vitro. Acta Pharmacol Sin. 2017 Dec;38(12):1589-1600.

108. Wu H, Yu W, Meng F, Mi J, Peng J, Liu J, Zhang X, Hai C, Wang X. Polychlorinated biphenyls-153 induces metabolic dysfunction through

activation of ROS/NF-κB signaling via downregulation of HNF1b. Redox Biol. 2017 Aug;12:300-310.

109.Lin XH, Pan JB, Zhang XJ. WITHDRAWN: Anti-inflammatory and anti-oxidant effects of apigetrin on LPS-induced acute lung injury by regulating Nrf2 and AMPK pathways. Biochem Biophys Res Commun. 2017 Jul 13. pii: S0006-291X(17)31413-4.

110.Zhang J, Zhou X, Wu W, Wang J, Xie H, Wu Z. Regeneration of glutathione by α-lipoic acid via Nrf2/ARE signaling pathway alleviates cadmium-induced HepG2 cell toxicity. Environ Toxicol Pharmacol. 2017 Apr;51:30-37.

111.Jiang Y, Cao Y, Wang Y, Li W, Liu X, Lv Y, Li X, Mi J. Cysteine transporter SLC3A1 promotes breast cancer tumorigenesis. Theranostics. 2017 Feb 26;7(4):1036-1046.

112.Huang L, Zhang K, Guo Y, Huang F, Yang K, Chen L, Huang K, Zhang F, Long Q, Yang Q. Honokiol protects against doxorubicin cardiotoxicity via improving mitochondrial function in mouse hearts. Sci Rep. 2017 Sep 20;7(1):11989.

113.Li X, Li X, Lu J, Huang Y, Lv L, Luan Y, Liu R, Sun R. Saikosaponins induced hepatotoxicity in mice via lipid metabolism dysregulation and oxidativestress: a proteomic study. BMC Complement Altern Med. 2017 Apr 19;17(1):219.

114.Lu S, Zhang Y, Zhong S, Gao F, Chen Y, Li W, Zheng F, Shi G. N-n-butyl Haloperidol Iodide Protects against Hypoxia/Reoxygenation Injury in CardiacMicrovascular EndothelialCells by Regulating the ROS/MAPK/Egr-1 Pathway. Front Pharmacol. 2017 Jan 5;7:520.

115.Liu X, Liu H, Zhai Y, Li Y, Zhu X, Zhang W. Laminarin protects against hydrogen peroxide-induced oxidative damage in MRC-5 cells possiblyvia regulating NRF2. PeerJ. 2017 Jul 31;5:e3642.

116.Xu J, Bian X, Liu Y, Hong L, Teng T, Sun Y, Xu Z. Adenosine A2 receptor activation ameliorates mitochondrial oxidative stress upon reperfusion throughthe posttranslational modification of NDUFV2 subunit of complex I in the heart. Free Radic Biol Med. 2017 May;106:208-218.

117.Jiang LL, Zhao DS, Fan YX, Yu Q, Li P, Li HJ. Detection of Emodin Derived Glutathione Adduct in Normal Rats Administered with Large Dosage of Polygoni Multiflori Radix. Front Pharmacol. 2017 Jul 6;8:446.

118.Guo J, Xu B, Han Q, Zhou H, Xia Y, Gong C, Dai X, Li Z, Wu G. Ferroptosis:A Novel Anti-Tumor Action for Cisplatin. Cancer Res Treat. 2017 May 10.

119.Duan M, Zhang R, Zhu F, Zhang Z, Gou L, Wen J, Dong J, Wang T. A Lipid-Anchored NAC Transcription Factor Is Translocated intothe Nucleus and ActivatesGlyoxalase I Expression during Drought Stress. Plant Cell. 2017 Jul;29(7):1748-1772.

120.Zhang Z, Chen J, Li B, He C, Chen Y, Tian S. Influence of Oxidative Stress on Biocontrol Activity of Cryptococcus laurentii against Blue Mold on Peach Fruit. Front Microbiol. 2017 Feb 2;8:151.

121.An B, Lan J, Deng X, Chen S, Ouyang C, Shi H, Yang J, Li Y. Silencing of D-Lactate Dehydrogenase Impedes Glyoxalase System and Leads to MethylglyoxalAccumulation and Growth Inhibition in Rice. Front Plant Sci. 2017 Dec 5;8:2071.

122.Duan M, Zhang R, Zhu F, Zhang Z, Gou L, Wen J, Dong J, Wang T. A Lipid-Anchored NAC Transcription Factor Is Translocated into the Nucleus and Activates Glyoxalase I Expression during Drought Stress. Plant Cell. 2017 Jul;29(7):1748-1772.

123.You X, Liu L, Li X, Du H, Nie D, Zhang X, Tong H, Wu M, Gao Y, Liao Z. Immune response ofinterferon-γ-inducible lysosomal thiol reductase (GILT)from Chinesesturgeon (Acipenser sinensis)to microbial invasion andits antioxdative activity in lipopolysaccharides-treated mammalian dentritic cells. Fish Shellfish Immunol. 2018 Jan;72:356-366.

124.Luo CQ, Zhou YX, Zhou TJ, Xing L, Cui PF, Sun M, Jin L, Lu N, Jiang HL. Reactive oxygen species-responsive nanoprodrug with quinone methides-mediated GSHdepletion for improved chlorambucil breast cancers therapy. J Control Release. 2018 Mar 28;274:56-68. Zhou Q, Fu X, Wang X, Wu Q, Lu Y, Shi J, Klaunig JE, Zhou S. Autophagy plays a protective role in Mn-induced toxicity in PC12 cells. Toxicology. 2018 Feb 1;394:45-53.

125.Zhou Q, Fu X, Wang X, Wu Q, Lu Y, Shi J, Klaunig JE, Zhou S. Autophagy plays a protective role in Mn-induced toxicity in PC12 cells. Toxicology. 2018 Feb 1;394:45-53.

126.Zhang Y, Li XR, Zhao L, Duan GL, Xiao L, Chen HP. DJ-1 preserving mitochondrial complex I activity plays a critical role in resveratrol-mediatedcardioprotection against hypoxia/reoxygenation-induced oxidative stress. Biomed Pharmacother. 2018 Feb;98:545-552.

127.Dai J, Gu L, Su Y, Wang Q, Zhao Y, Chen X, Deng H, Li W, Wang G, Li K. Inhibition of curcumin on influenza A virus infection and influenzal pneumonia via oxidativestress, TLR2/4, p38/JNK MAPK and NF-κB pathways. Int Immunopharmacol. 2018 Jan;54:177-187.

128.Lu MC, Jiao Q, Liu T, Tan SJ, Zhou HS, You QD, Jiang ZY. Discovery ofa head-to-tail cyclic peptide as the Keap1-Nrf2 protein-protein interaction inhibitor with high cell potency. Eur J Med Chem. 2018 Jan 1;143:1578-1589.

129.Fan XY, Liu YJ, Chen K, Jiang FL, Hu YJ, Liu D, Liu Y, Ge YS. Organic arsenicals target thioredoxin reductase followed by oxidative stress and mitochondrialdysfunction resulting in apoptosis. Eur J Med Chem. 2018 Jan 1;143:1090-1102.

130.Zhang S, An Q, Wang T, Gao S, Zhou G. Autophagy-and MMP-2/9-mediated Reduction and Redistribution of ZO-1 Contribute to Hyperglycemia-increased Blood-Brain Barrier Permeability during Early Reperfusion in Stroke. Neuroscience. 2018 Mar 7. pii: S0306-4522(18)30160-X.

131.Bai Z, Gao M, Xu X, Zhang H, Xu J, Guan Q, Wang Q, Du J, Li Z, Zuo D, Zhang W, Wu Y. Overcoming resistance to mitochondrial apoptosis by BZML-induced mitotic catastrophe is enhanced by inhibition of autophagy in A549/Taxol cells. Cell Prolif. 2018 Mar 1.

132.Feng M, Yin H, Cao Y, Peng H, Lu G, Liu Z, Dang Z. Cadmium-induced stress response of Phanerochaete chrysosporium duringthe biodegradation of 2,2',4,4'-tetrabromodiphenyl ether (BDE-47). Ecotoxicol Environ Saf. 2018 Jun 15;154:45-51.

133.Fu LH, Wei ZZ, Hu KD, Hu LY, Li YH, Chen XY, Han Z, Yao GF, Zhang H. Hydrogen sulfide inhibits the growth of Escherichia coli through oxidative damage. J Microbiol. 2018 Feb 28.

134.Wang Q, Pu Y, Yang D, Yin X, He Z, Yang Y, Yang Y. Molecular cloning and characterization of the glutathione reductase gene from Stipa purpurea. Biochem Biophys Res Commun. 2018 Jan 8;495(2):1851-1857.

135.Liu Y, Xu C, Zhu Y, Zhang L, Chen T, Zhou F, Chen H, Lin Y. The calcium-dependent kinase OsCPK24 functions in cold stress responses in rice. J Integr Plant Biol. 2018 Feb;60(2):173-188.

136.Su YQ, Zhao YJ, Wu N, Chen YE, Zhang WJ, Qiao DR, Cao Y. Chromium removal from solution by five photosynthetic bacteria isolates. Appl Microbiol Biotechnol. 2018 Feb;102(4):1983-1995.

注：更多使用本产品的文献请参考产品网页。

Version 2019.04.12
