# Supplementary material for: Combination decoction of Astragalus mongholicus and Salvia miltiorrhiza mitigates pressure-overload cardiac dysfunction by inhibiting multiple ferroptosis pathways
Source: Front Pharmacol. 2024 Dec 16;15:1447546. doi: 10.3389/fphar.2024.1447546 (PMC11683366; doi:10.3389/fphar.2024.1447546)
Supplement: Supplementary file 4 [file DataSheet1.ZIP › S0131S Lipid Peroxidation MDA (Malondialdehyde) Assay Kit.pdf]

碧云天 Beyotime

碧云天网站 微信公众号

碧云天生物技术/Beyotime Biotechnology
订货热线：400-168-3301 或 800-8283301
订货 e-mail：order@beyotime.com
技术咨询：info@beyotime.com
网址：http://www.beyotime.com

脂质氧化(MDA)检测试剂盒

产品编号	产品名称	包装
S0131S	脂质氧化(MDA)检测试剂盒	100次
S0131M	脂质氧化(MDA)检测试剂盒	500次

产品简介：

- 碧云天的脂质氧化(MDA)检测试剂盒(Lipid Peroxidation MDA Assay Kit)采用一种基于MDA和硫代巴比妥酸(thiobarbituric acid, TBA)反应产生红色产物的显色反应，随后通过比色法用于对血浆、血清、尿液、动植物组织或细胞裂解液中MDA进行定量检测，广泛用于脂质氧化(lipid peroxidation) 水平检测的试剂盒。
- 丙二醛(Malondialdehyde, MDA)是一种生物体脂质氧化的天然产物。动物或植物细胞发生氧化应激(oxidative stress)时，会发生脂质氧化。一些脂肪酸氧化后逐渐分解为一系列复杂的化合物，其中包括MDA。此时通过检测MDA的水平即可检测脂质氧化的水平，因此MDA的测定被广泛用作脂质氧化的指标。生物体内的一些其它生化反应也会产生MDA，例如thromboxane synthase也可以催化产生，但只要在测定时设置适当对照即可观察到脂质氧化水平的变化。
- 丙二醛在较高温度及酸性环境中可与TBA发生反应，形成红色的MDA-TBA加合物，相应的反应原理图如下：

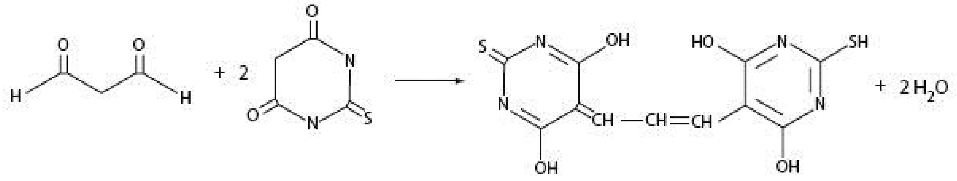

MDA TBA MDA-TBA Adduct

- MDA-TBA加合物在535nm处有最大吸收，据此可以通过比色法进行检测。另外，MDA-TBA加合物也可以在535nm被激发产生最大发射波长553nm，据此也可以进行荧光检测。
- **特点：**本试剂盒中采用了特殊的抗氧化剂，可以有效地抑制样品在检测过程中产生新的MDA，使检测更加准确。同时本检测试剂盒在检测过程中可以把部分MDA天然形成的聚丙二醛分解成MDA，使对脂质氧化的测定更加准确。
- 本试剂盒可以检测低达1μM的MDA，也可检测高达200μM的MDA(参考图1)。血浆、血清样品中的MDA含量通常在约2-4μM，尿液中的MDA含量通常在约5-30μM，在本试剂盒的检测范围内，可以直接用本试剂盒检测血浆、血清、尿液样品等。

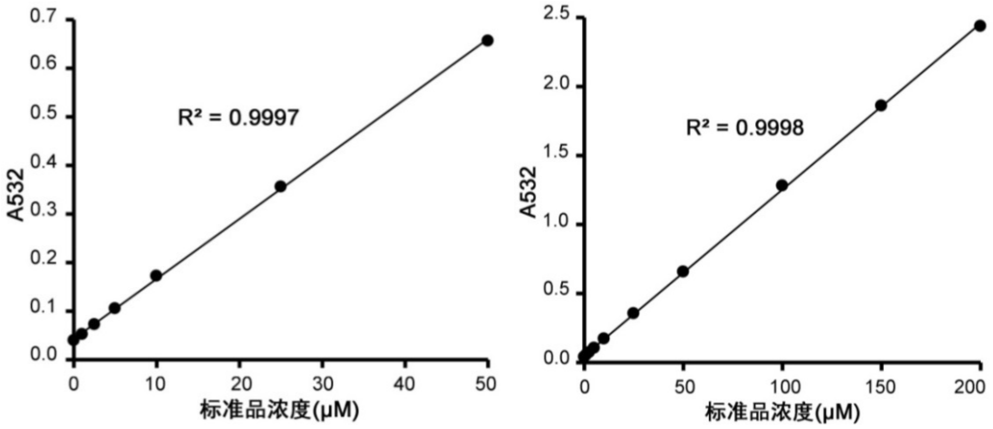

图1. 不同浓度标准品使用本试剂盒的检测效果图。实测数据会因检测仪器等的不同而存在差异，图中数据仅供参考。

- 本试剂盒S0131S包装共可进行100次检测，S0131M包装共可进行500次检测。

包装清单：

产品编号	产品名称	包装
S0131S-1	TBA	25mg
S0131S-2	TBA配制液	6.76ml
S0131S-3	TBA稀释液	15ml
S0131S-4	抗氧化剂	300μl
S0131S-5	标准品(1mM)	200μl

—	说明书	1份

产品编号	产品名称	包装
S0131M-1	TBA	125mg
S0131M-2	TBA配制液	35ml
S0131M-3	TBA稀释液	75ml
S0131M-4	抗氧化剂	1.5ml
S0131M-5	标准品(1mM)	1ml
—	说明书	1份

保存条件：

-20ºC保存，一年有效。S0131-1 TBA和S0131-4抗氧化剂需避光保存。S0131-1 TBA、S0131-2 TBA配制液和S0131-3 TBA稀释液可室温或4ºC存放三个月。

注意事项：

- 醛、较高浓度的可溶性糖(例如250mM蔗糖)对反应有干扰，可溶性糖与TBA显色反应的产物在532nm也有吸收(最大吸收在450nm)。如果可溶性糖对测定有干扰，可以通过测定450nm作为参考波长进行双波长测定，消除其干扰。
- 本产品仅限于专业人员的科学研究用，不得用于临床诊断或治疗，不得用于食品或药品，不得存放于普通住宅内。
- 为了您的安全和健康，请穿实验服并戴一次性手套操作。

使用说明：

1. 样品的准备：

a. 血浆、血清或尿液样品制备后可以直接用于MDA测定。

b. 组织或细胞可以使用PBS或碧云天的Western及IP细胞裂解液(P0013)等裂解液进行匀浆或裂解。对于组织，组织重量占匀浆液或裂解液的比例为10%；对于细胞，每100万细胞使用0.1ml裂解液或匀浆液。匀浆或裂解后，10,000g-12,000g离心10分钟取上清用于后续测定。对于一些特殊样品，离心不能获得澄清的上清溶液的，可以使用0.2微米孔径的过滤器过滤以获得澄清的样品溶液。匀浆或裂解等样品制备步骤宜在冰浴或4ºC进行操作。

c. 对于组织或细胞样品，样品准备完毕后可以用BCA蛋白浓度测定试剂盒(P0009/P0010/P0010S/P0011/P0012/P0012S)测定蛋白浓度，以便于后续计算单位蛋白重量组织或细胞内的MDA含量。

d. 本试剂盒对于样品中的常见化学成分的兼容性参考下表：

试剂类别	化学成分	是否干扰
缓冲试剂	Borate (50mM)	否
	HEPES (100mM)	否
	Phosphate (100mM)	否
	Tris (25mM)	否
去垢剂	CHAPS (≤1%)	否
	Triton X-100 (≤1%)	否
	Tween 20 (≤1%)	否
抑制剂/螯合剂	Antipain (≤100µg/ml)	否
	Chymostatin (≤10µg/ml)	否
	Leupeptin (≤10µg/ml)	否
	PMSF (≤200µM)	否
	Trypsin (≤10µg/ml)	否
	EDTA (≤1mM)	否
	EGTA (≤1mM)	否
其它试剂	Sucrose (250mM)	是
	Glycerol (≤10%)	否

2. 试剂盒的准备工作：

a. **TBA储存液的配制：**称取适量TBA，用TBA配制液配制成浓度为0.37%的TBA储存液。例如18.5mg TBA用5ml TBA配制液配制，或者25mg TBA用6.76ml TBA配制液配制，最终浓度即为0.37%。TBA配制液需完全溶解后再使用，可以加热到70ºC以促进溶解。TBA储存液较难溶解，需加热到70ºC，并通过剧烈Vortex以促进溶解。配制好的TBA储存液室温避光保存，至少3个月内有效。

b. **MDA检测工作液的配制：**根据待测定的样品数(含对照)，参考下表在临检测前新鲜配制适量的MDA检测工作液

检测次数	1次	10次	20次	50次

TBA稀释液	150μl	1500μl	3000μl	7500μl
TBA储存液	50μl	500μl	1000μl	2500μl
抗氧化剂	3μl	30μl	60μl	150μl

注意：MDA检测工作液较难溶解，可以70ºC加热，并剧烈Vortex以促进溶解。也可以通过超声处理以促进溶解。配制好的MDA检测工作液必须当天使用。

c. **标准品的稀释：**取适量标准品用蒸馏水稀释至1、2、5、10、20、50μM，用于后续制作标准曲线。如果样品中MDA的浓度很高，可以增加100、150和200μM的标准品浓度。

3. **样品测定：**

a. 在离心管或其它适当容器内加入0.1ml匀浆液、裂解液或PBS等适当溶液作为空白对照，加入0.1ml上述不同浓度标准品用于制作标准曲线，加入0.1ml样品用于测定；随后加入0.2ml MDA检测工作液。可参考下表设置检测反应体系：

	空白对照	标准品	样品
匀浆液、裂解液或PBS	0.1ml	—	—
标准品	—	0.1ml	—
待测样品	—	—	0.1ml
MDA检测工作液	0.2ml	0.2ml	0.2ml

b. 混匀后，100ºC或沸水浴加热15分钟。加热时务必注意避免液体暴沸溅出。如果使用加热块(Heat block)进行加热注意用重物压紧离心管盖；如果使用沸水浴，则需使用可把盖子锁死的离心管或螺旋盖离心管，或用Parafilm封住离心管口，用针头刺一小孔。最方便和准确的加热方法是使用带有热盖并可以加热0.5ml PCR管的PCR仪。

c. 水浴冷却至室温，1000g室温离心10分钟。取200微升上清加入到96孔板中，随后用酶标仪在532nm测定吸光度。如果不方便测定532nm的吸光度，也可以测定530-540nm之间的吸光度。可以设定450nm为参考波长进行双波长测定。

d. MDA含量的计算：对于血浆、血清或尿液等样品可以直接根据标准曲线计算获得MDA的摩尔浓度，对于细胞、或组织样品，计算出样品溶液中的MDA含量后，可以通过单位重量的蛋白含量或组织重量等来表示最初样品中的MDA含量，例如μmol/mg蛋白或μmol/mg组织。

常见问题：

1. 没有检测到MDA。

可能样品中MDA浓度过低，在检测限之下。在检测组织或细胞的MDA时，请注意使用更多的组织或细胞。并注意尽量不要稀释样品。

使用本产品的文献：

1. Qian J, Jiang F, Wang B, Yu Y, Zhang X, Yin Z, Liu C. Ophiopogonin D prevents H2O2-induced injury in primary human umbilical vein endothelial cells. J Ethnopharmacol. 2010;128(2):438-45.
2. Gong K, Li W. Shikonin, a Chinese plant-derived naphthoquinone, induces apoptosis in hepatocellular carcinomacells through reactive oxygen species:A potential new treatment for hepatocellular carcinoma. Free Radic Biol Med. 2011 Dec 15;51(12):2259-71.
3. Xie C, Liu N, Long J, Tang C, Li J, Huo L, Wang X, Chen P, Liang S. Blue native/SDS-PAGE combined with iTRAQ analysis reveals advanced glycation end-product-induced changes of synaptosome proteins in C57 BL/6 mice. Electrophoresis. 2011 Aug;32(16):2194-205.
4. Liu C, Gong K, Mao X, Li W. Tetrandrine induces apoptosis by activating reactive oxygen species and repressing Akt activity inhuman hepatocellular carcinoma. Int J Cancer. 2011 Sep 15;129(6):1519-31.
5. Jiang F, Qian J, Chen S, Zhang W, Liu C. Ligustrazine improves atherosclerosis in rat via attenuation of oxidative stress. Pharm Biol. 2011 Aug;49(8):856-63.
6. Fan S, Li L, Chen S, Yu Y, Qi M, Tashiro S, Onodera S, Ikejima T. Silibinin induced-autophagic and apoptotic death is associated with an increase in reactive oxygen and nitrogen species in HeLa cells. Free Radic Res. 2011 Nov;45(11-12):1307-24.
7. Wang N, Wang Y, Yu G, Yuan C, Ma J. Quinoprotein adducts accumulate in the substantia nigra of aged rats and correlate with dopamine-induced toxicity in SH-SY5Y cells. Neurochem Res. 2011 Nov;36(11):2169-75.
8. He MD, Xu SC, Lu YH, Li L, Zhong M, Zhang YW, Wang Y, Li M, Yang J, Zhang GB, Yu ZP, Zhou Z. L-carnitine protects against nickel-induced neurotoxicity by maintaining mitochondrial function inNeuro-2a cells. Toxicol Appl Pharmacol. 2011 May 15;253(1):38-44.
9. Min L, He S, Chen Q, Peng F, Peng H, Xie M. Comparative proteomic analysis of cellular response of human airway epithelial cells (A549) tobenzo(a)pyrene. Toxicol Mech Methods. 2011 Jun;21(5):374-82.
10. Shi X, Gu A, Ji G, Li Y, Di J, Jin J, Hu F, Long Y, Xia Y, Lu C, Song L, Wang S, Wang X. Developmental toxicity of cypermethrin in embryo-larval stages of zebrafish. Chemosphere. 2011 Oct;85(6):1010-6.
11. Chen T, Zhang L, Yue JQ, Lv ZQ, Xia W, Wan YJ, Li YY, Xu SQ. Prenatal PFOS exposure induces oxidative stress and apoptosis in the lung of rat off-spring. Reprod Toxicol. 2012 Jul;33(4):538-45.
12. Zhan Y, Gong K, Chen C, Wang H, Li W. P38 MAP kinase functions as a switch in MS-275-induced reactive oxygen species-dependentautophagy and apoptosis in Human colon Cancer cells. Free Radic Biol Med. 2012 Aug 1;53(3):532-43.
13. Gong K, Xie J, Yi H, Li W. CS055 (Chidamide/HBI-8000), a novel histone deacetylase inhibitor, induces G1 arrest, ROS-dependent apoptosis and differentiation in human leukaemia cells. Biochem J. 2012 May 1;443(3):735-46.
14. Wu L, Zhang Y, Ma X, Zhang N, Qin G. The effect of resveratrol on FoxO1 expression in kidneys of diabetic nephropathy rats. Mol Biol Rep. 2012 Sep;39(9):9085-93.
15. Xu SC, Chen YB, Lin H, Pi HF, Zhang NX, Zhao CC, Shuai L, Zhong M, Yu ZP, Zhou Z, Bie P. Damage to mtDNA in liver injury of patients with extrahepatic cholestasis: the protective effects ofmitochondrial transcription factor A. Free Radic Biol Med. 2012 May 1;52(9):1543-51.
16. Guo C, He Z, Wen L, Zhu L, Lu Y, Deng S, Yang Y, Wei Q, Yuan H. Cytoprotective effect of trolox against oxidative damage and apoptosis in the NRK-52e cellsinduced by melamine. Cell Biol Int. 2012 Feb;36(2):183-8.
17. Fang H, Wu Y, Guo J, Rong J, Ma L, Zhao Z, Zuo D, Peng S. T-2 toxin induces apoptosis in differentiated murine embryonic stem cells through reactiveoxygen species-mediated mitochondrial pathway. Apoptosis. 2012 Aug;17(8):895-907.
18. Kang X, Xie Q, Zhou X, Li F, Huang J, Liu D, Huang T. Effects of hepatitis B virus S protein exposure on sperm membrane integrity and functions. PLoS One. 2012;7(3):e33471.
19. Zhang JK, Yang L, Meng GL, Fan J, Chen JZ, He QZ, Chen S, Fan JZ, Luo ZJ, Liu J. Protective effect of tetrahydroxystilbene glucoside against hydrogen peroxide-induceddysfunction and oxidative stress in osteoblastic MC3T3-E1 cells. Eur J Pharmacol. 2012 Aug 15; 689(1-3):31-7.
20. Gong JS, Yao YT, Fang NX, Li LH. Sevoflurane postconditioning

attenuates reperfusion-induced ventricular arrhythmias in isolatedrat hearts exposed to ischemia/reperfusion injury. Mol Biol Rep. 2012 Jun;39(6):6417-25.

21. Gong K, Chen C, Zhan Y, Chen Y, Huang Z, Li W. Autophagy-related gene 7 (ATG7) and reactive oxygen species/extracellular signal-regulated kinase regulatetetrandrine-induced autophagy in human hepatocellular carcinoma. J Biol Chem. 2012 Oct 12;287(42):35576-88.
22. Niu Y, Cao X, Song F, Xie T, Ji X, Miao M, Dong J, Tian M, Lin Y, Lu S. Reduced dermis thickness and AGE accumulation in diabetic abdominal skin. Int J Low Extrem Wounds. 2012 Sep;11(3):224-30.
23. Yue R, Hu H, Yiu KH, Luo T, Zhou Z, Xu L, Zhang S, Li K, Yu Z. Lycopene protects against hypoxia/reoxygenation-induced apoptosis by preventing mitochondrial dysfunction inprimary neonatal mouse cardiomyocytes. PLoS One. 2012;7(11):e50778.
24. Tang Y, Wang F, Jin C, Liang H, Zhong X, Yang Y. Mitochondrial injury induced by nanosized titanium dioxide in A549 cells and rats. Environ Toxicol Pharmacol. 2013 Jul;36(1):66-72.
25. Liu J, Zhu Y, Du G, Zhou J, Chen J. Response of Saccharomyces cerevisiae to D-limonene-induced oxidative stress. Appl Microbiol Biotechnol. 2013 Jul;97(14):6467-75.
26. Wan J, Liu T, Mei L, Li J, Gong K, Yu C, Li W. Synergistic antitumour activity of sorafenib in combination with tetrandrine is mediated by reactive oxygenspecies (ROS)/Akt signaling. Br J Cancer. 2013 Jul 23; 109(2):342-50.
27. Yuan H, Zhang W, Li H, Chen C, Liu H, Li Z. Neuroprotective effects of resveratrol on embryonic dorsal root ganglion neurons with neurotoxicity induced byethanol. Food Chem Toxicol. 2013 May;55:192-201.
28. Yi F, He X, Wang D. Lycopene Protects Against MPP(+)-Induced Cytotoxicity by Maintaining Mitochondrial Function in SH-SY5Y Cells. Neurochem Res. 2013 Aug;38(8):1747-57.
29. Chen Y, Lv L, Jiang Z, Yang H, Li S, Jiang Y. Mitofusin 2 Protects Hepatocyte Mitochondrial Function from Damage Induced by GCDCA. PLoS One. 2013 Jun 6;8(6):e65455.
30. Liu L, Wang P, Liang C, He D, Yu Y, Liu X. Distinct effects of Nampt inhibition on mild and severe models of lipopolysaccharide-induced myocardialimpairment. Int Immunopharmacol. 2013 Oct;17(2):342-9.
31. Wang Y, Gao D, Chen Z, Li S, Gao C, Cao D, Liu F, Liu H, Jiang Y. Acridone derivative 8a induces oxidative stress-mediated apoptosis in CCRF-CEM leukemia cells: application of metabolomics in mechanistic studies of antitumor agents. PLoS One. 2013 May 7;8(5):e63572.
32. Wu J, Tu D, Yuan LY, Yuan H, Wen LX. T-2 toxin exposure induces apoptosis in rat ovarian granulosa cells through oxidative stress. Environ Toxicol Pharmacol. 2013 Apr 11;36(2):493-500.
33. Miao Y, Zhou J, Zhao M, Liu J, Sun L, Yu X, He X, Pan X, Zang W. Acetylcholine attenuates hypoxia/ reoxygenation-induced mitochondrial and cytosolic ROS formation in H9c2cells via M2 acetylcholine receptor. Cell Physiol Biochem. 2013;31(2-3):189-98.
34. Xu S, Pi H, Chen Y, Zhang N, Guo P, Lu Y, He M, Xie J, Zhong M, Zhang Y, Yu Z, Zhou Z. Cadmium induced Drp1-dependent mitochondrial fragmentation by disturbing calcium homeostasis in itshepatotoxicity. Cell Death Dis. 2013 Mar 14;4:e540.
35. He MD, Xu SC, Zhang X, Wang Y, Xiong JC, Zhang X, Lu YH, Zhang L, Yu ZP, Zhou Z. Disturbance of aerobic metabolism accompanies neurobehavioral changes induced by nickel in mice. Neurotoxicology. 2013 May 29;38C:9-16.
36. Sun L, Jin Y, Dong L, Sumi R, Jahan R, Li Z. The neuroprotective effects of Coccomyxa gloeobotrydiformis on the ischemic stroke in a rat model. Int J Biol Sci. 2013 Aug 20;9(8):811-7.
37. Zhao J, Bai Y, Zhang C, Zhang X, Zhang YX, Chen J, Xiong L, Shi M, Zhao G. Cinepazide maleate protects PC12 cells against oxygen-glucose deprivation-induced injury. Neurol Sci. 2014 Jun;35(6):875-81.
38. Zhao H, Wang R, Tao Z, Yan F, Gao L, Liu X, Wang N, Min L, Jia Y, Zhao Y, Ji X, Luo Y. Activation of T-LAK-cell-originated protein kinase-mediated antioxidation protects against focal cerebral ischemia-reperfusion injury. FEBS J. 2014 Oct;281(19):4411-20.
39. Chen XL, Tang WX, Tang XH, Qin W, Gong M. Downregulation of uncoupling protein-2 by genipin exacerbates diabetes-induced kidney proximal tubular cells apoptosis. Ren Fail. 2014 Sep;36(8):1298-303.
40. Wang L, Yuan R, Yao C, Wu Q, Christelle M, Xie W, Zhang X, Sun W, Wang H, Yao S. Effects of resolvin D1 on inflammatory responses and oxidative stress of lipopolysaccharide-induced acute lung injury in mice. Chin Med J (Engl). 2014;127(5):803-9.
41. Fang L, Bai C, Chen Y, Dai J, Xiang Y, Ji X, Huang C, Dong Q. Inhibition of ROS production through mitochondria-targeted antioxidant and mitochondrial uncoupling increases post-thaw sperm viability in yellow catfish. Cryobiology. 2014 Dec;69(3):386-93.
42. She F, Wang W, Wang Y, Tang P, Wei J, Chen H, Zhang B. Melatonin protects MG63 osteoblast-like cells from hydrogen peroxide-induced cytotoxicity by maintaining mitochondrial function. Mol Med Rep. 2014 Feb;9(2):493-8.
43. Jiang D, Chen K, Lu X, Gao HJ, Qin ZH, Lin F. Exercise ameliorates the detrimental effect of chloroquine on skeletal muscles in mice via restoring autophagy flux. Acta Pharmacol Sin. 2014 Jan;35(1):135-42.
44. Gong K, Zhang Z, Chen Y, Shu HB, Li W. Extracellular signal-regulated kinase, receptor interacting protein, and reactive oxygen species regulate shikonin-induced autophagy in human hepatocellular carcinoma. Eur J Pharmacol. 2014 Sep 5;738:142-52.
45. Wang X, Chang Q, Wang Y, Su F, Zhang S. Late-onset temperature reduction can retard the aging process in aged fish via a combined action of an anti-oxidant system and the insulin/insulin-like growth factor 1 signaling pathway. Rejuvenation Res. 2014 Dec;17(6):507-17.
46. Chen H, Zha J, Liang X, Li J, Wang Z. Effects of the human antiepileptic drug carbamazepine on the behavior, biomarkers, and heat shock proteins in the Asian clam Corbicula fluminea. Aquat Toxicol. 2014 Oct;155:1-8.
47. Ji H, Wu L, Ma X, Ma X, Qin G. The effect of resveratrol on the expression of AdipoR1 in kidneys of diabetic nephropathy. Mol Biol Rep. 2014;41(4):2151-9.
48. Zhao H, Zhao W, Lok K, Wang Z, Yin M. A synergic role of caspase-6 and caspase-3 in Tau truncation at D421 induced by H2O2. Cell Mol Neurobiol. 2014 Apr;34(3):369-78.
49. Lei T, Li H, Fang Z, Lin J, Wang S, Xiao L, Yang F, Liu X, Zhang J, Huang Z, Liao W. Polysaccharides from Angelica sinensis alleviate neuronal cell injury caused by oxidative stress. Neural Regen Res. 2014 Feb 1;9(3):260-7.
50. Chen Z, Wang G, Zhai X, Hu Y, Gao D, Ma L, Yao J, Tian X. Selective inhibition of protein kinase C β2 attenuates the adaptor P66 Shc-mediated intestinal ischemia-reperfusion injury. Cell Death Dis. 2014 Apr 10;5:e1164.
51. Yu C, Zhou Z, Wang J, Sun J, Liu W, Sun Y, Kong B, Yang H, Yang S. In depth analysis of apoptosis induced by silica coated manganese oxide nanoparticles in vitro. J Hazard Mater. 2015 Feb 11;283:519-28.
52. Li X, Zhu F, Jiang J, Sun C, Wang X, Shen M, Tian R, Shi C, Xu M, Peng F, Guo X, Wang M, Qin R. Synergistic antitumor activity of withaferin A combined with oxaliplatin triggers reactive oxygen species-mediated inactivation of the PI3K/AKT pathway in human pancreatic cancer cells. Cancer Lett. 2015 Feb 1;357(1):219-30.
53. Ge H, Zhang F, Shan D, Chen H, Wang X, Ling C, Xi H, Huang J, Zhu C, Lv J.Effects of Mitochondrial Uncoupling Protein 2 Inhibition by Genipin in Human Cumulus Cells.Biomed Res Int. 2015;2015:323246.
54. Yuan ZY, Hu YL, Gao JQ.Brain Localization and Neurotoxicity Evaluation of Polysorbate 80-Modified Chitosan Nanoparticles in Rats.PLoS One. 2015 Aug 6;10(8):e0134722.
55. Zhao D, Li Q, Huang Q, Li X, Yin M, Wang Z, Hong J.Cardioprotective effect of propofol against oxygen glucose deprivation and reperfusion injury in H9c2 cells.Oxid Med Cell Longev. 2015;2015:184938.
56. Wang D, Ma Y, Yang X, Xu X, Zhao Y, Zhu Z, Wang X, Deng H, Li C, Gao F, Tong J, Yamanaka K, An Y.Hypermethylation of the Keap1 gene inactivates its function, promotes Nrf2 nuclear accumulation, and is involved in arsenite-induced human keratinocyte transformation.Free Radic Biol Med. 2015 Dec;89:209-19.
57. Wang G, Kang N, Gong H, Luo Y, Bai C, Chen Y, Ji X, Huang C, Dong Q.Upregulation of uncoupling protein Ucp2 through acute cold exposure increases post-thaw sperm quality in zebrafish.Cryobiology. 2015 Dec; 71(3):464-71.
58. Yu C, Zhou Z, Wang J, Sun J, Liu W, Sun Y, Kong B, Yang H, Yang S.In depth analysis of apoptosis induced by silica coated manganese oxide nanoparticles in vitro.J Hazard Mater. 2015;283:519-28.
59. Li X, Xu L, Zhou W, Zhao Q, Wang Y.Chronic exposure to microcystin-LR affected mitochondrial DNA maintenance and caused pathological changes of lung tissue in mice.Environ Pollut. 2015 Dec 16;210:48-56.
60. Liu XW, Zi Y, Liu YE, Zhang YB, Xiang LB, Hou MX.Melatonin exerts protective effect on N2a cells under hypoxia conditions through Zip1/ERK pathway.Neurosci Lett. 2015 May 19;595:74-80.
61. Wang X, Chen L, Wang T, Jiang X, Zhang H, Li P, Lv B, Gao

X.Ginsenoside Rg3 antagonizes adriamycin-induced cardiotoxicity by improving endothelial dysfunction from oxidative stress via upregulating the Nrf2-ARE pathway through the activation of akt.Phytomedicine. 2015 Sep 15;22(10):875-84.

62. Qin G, Zhou Y, Guo F, Ren L, Wu L, Zhang Y, Ma X, Wang Q.Overexpression of the FoxO1 Ameliorates Mesangial Cell Dysfunction in Male Diabetic Rats.Mol Endocrinol. 2015 Jul;29(7):1080-91.
63. Shan W, Gao L, Zeng W, Hu Y, Wang G, Li M, Zhou J, Ma X, Tian X, Yao J.Activation of the SIRT1/p66shc antiapoptosis pathway via carnosic acid-induced inhibition of miR-34a protects rats against nonalcoholic fatty liver disease.Cell Death Dis. 2015 Jul 23;6:e1833.
64. Yang J, Li D, Fan Q, Cai L, Qiu X, Zhou P, Lu Y.The Polymorphisms with Cataract Susceptibility Impair the EPHA2 Receptor Stability and Its Cytoprotective Function.J Ophthalmol. 2015;2015:401894.
65. Wang YG, Yang TL.Liraglutide reduces oxidized LDL-induced oxidative stress and fatty degeneration in Raw 264.7 cells involving the AMPK/SREBP1 pathway.J Geriatr Cardiol. 2015 Jul;12(4):410-6.
66. Yang X, Wang D, Ma Y, Xu X, Zhu Z, Wang X, Deng H, Li C, Chen M, Tong J, Yamanaka K, An Y.Continuous activation of Nrf2 and its target antioxidant enzymes leads to arsenite-induced malignant transformation of human bronchial epithelial cells.Toxicol Appl Pharmacol. 2015 Dec 1;289(2):231-9.
67. Song XY, Xu S, Hu JF, Tang J, Chu SF, Liu H, Han N, Li JW, Zhang DM, Li YT, Chen NH.Piperine prevents cholesterol gallstones formation in mice.Eur J Pharmacol. 2015 Mar 15;751:112-7.
68. Ouyang Z, Cao W, Zhu S, Liu X, Zhong Z, Lai X, Xiao C, Jiang S, Wang Y.Protective effect of 2-deoxy-D-glucose on the cytotoxicity of cyclosporin A in vitro.Mol Med Rep. 2015 Aug;12(2):2814-20.
69. Xie Z, Liu L, Zhu W, Liu H, Wang L, Zhang J, Chen C, Zhu H.The protective effect of polymerized porcine hemoglobin (pPolyHb) on transient focal cerebral ischemia/reperfusion injury.Artif Cells Nanomed Biotechnol. 2015 Jun;43(3):180-5.
70. Yu HC, Wu J, Zhang HX, Zhang HS, Qiao TT, Zhang JX, Zhang GL, Sui J, Li LW, Zhang LR, Lv LX.Antidepressant-like and anti-oxidative efficacy of Campsis grandiflora flower.J Pharm Pharmacol. 2015 Dec;67(12):1705-15.
71. Jiang Z, Zou Y, Ge Z, Zuo Q, Huang SY, Sun L.A Role of sFlt-1 in Oxidative Stress and Apoptosis in Human and Mouse Pre-Eclamptic Trophoblasts.Biol Reprod. 2015 Sep;93(3):73.
72. Zheng YS, Wu ZS, Zhang LY, Ke L, Li WQ, Li N, Li JS.Nicotine ameliorates experimental severe acute pancreatitis via enhancing immunoregulation of CD4+ CD25+ regulatory T cells.Pancreas. 2015 Apr;44(3):500-6.
73. Guo L, Li S, Zhao Y, Qian P, Ji F, Qian L, Wu X, Qian G.Silencing Angiopoietin-Like Protein 4 (ANGPTL4) Protects Against Lipopolysaccharide-Induced Acute Lung Injury Via Regulating SIRT1 /NF-kB Pathway.J Cell Physiol. 2015 Oct;230(10):2390-402.
74. Zheng YS, Wu ZS, Zhang LY, Ke L, Li WQ, Li N, Li JS.Nicotine ameliorates experimental severe acute pancreatitis via enhancing immunoregulation of CD4+ CD25+ regulatory T cells.Pancreas. 2015 Apr;44(3):500-6.
75. Li ZY, Song J, Zheng SL, Fan MB, Guan YF, Qu Y, Xu J, Wang P, Miao CY.Adipocyte Metrnl Antagonizes Insulin Resistance Through PPARγ Signaling.Diabetes. 2015 Dec;64(12):4011-22.
76. Wu L, Wang Q, Guo F, Zhou Y, Ji H, Liu F, Ma X, Zhao Y, Qin G.Activation of FoxO1/ PGC-1α prevents mitochondrial dysfunction and ameliorates mesangial cell injury in diabetic rats.Mol Cell Endocrinol. 2015 Sep 15;413:1-12.
77. Wang LS, Hu Y, Li CL, Li Y, Wei YR, Yin ZF, Du YK, Min Z, Weng D, Chen JM, Li HP.N-acetylcysteine attenuates cigaret smoke-induced pulmonary exacerbation in a mouse model of emphysema.Inhal Toxicol. 2015 Dec;27(14):802-9.
78. Yuan Z, Matias FB, Yi JE, Wu J.T-2 toxin-induced cytotoxicity and damage on TM3 Leydig cells.Comp Biochem Physiol C Toxicol Pharmacol. 2016 Mar-Apr;181-182:47-54.
79. Chen XH, Zhou X, Yang XY, Zhou ZB, Lu DH, Tang Y, Ling ZM, Zhou LH, Feng X.Propofol Protects Against H2O 2-Induced Oxidative Injury in Differentiated PC12 Cells via Inhibition of Ca2+-Dependent NADPH Oxidase.Cell Mol Neurobiol. 2016 May;53(4):2408-88.
80. Du L, Sun W, Zhang H, Chen D.BDE-209 inhibits pluripotent genes expression and induces apoptosis in human embryonic stem cells.J Appl Toxicol. 2016 May;36(5):659-68.
81. Wang X, Yu X, Xie C, Tan Z, Tian Q, Zhu D, Liu M, Guan Y. Rescue of Brain Function Using Tunneling Nanotubes Between Neural Stem Cells and Brain Microvascular Endothelial Cells.Mol Neurobiol. 2016 May;53(4):2408-88.
82. Wei W, Feng W, Xin G, Tingting N, Zhanghe Z, Haimin C, Xiaojun Y. Enhanced effect of κ-carrageenan on TNBS-induced inflammation in mice. Int Immunopharmacol. 2016 Oct;39:218-28.
83. Gu LL, Zhang XY, Xing WM, Xu JD, Lu H. Andrographolide-induced apoptosis in human renal tubular epithelial cells: Roles of endoplasmic reticulumstress and inflammatory response. Environ Toxicol Pharmacol. 2016 Jul;45:257-64.
84. Jiao Y, Ma S, Wang Y, Li J, Shan L, Liu Q, Liu Y, Song Q, Yu F, Yu H, Liu H, Huang L, Chen J. N-Acetyl Cysteine Depletes Reactive Oxygen Species and Prevents Dental Monomer-Induced IntrinsicMitochondrial Apoptosis In Vitro in Human Dental Pulp Cells. PLoS One. 2016 Jan 25;11(1):e0147858.
85. Zhang J, Xia Y, Xu Z, Deng X. Propofol Suppressed Hypoxia/ Reoxygenation-Induced Apoptosis in HBVSMC by Regulation of theExpression of Bcl-2, Bax, Caspase3, Kir6.1, and p-JNK. Oxid Med Cell Longev. 2016;2016:1518738.
86. Yao N, Li YJ, Lei YH, Hu N, Chen WM, Yao Z, Yu M, Liu JS, Ye WC, Zhang DM. A piperazidine derivative of 23-hydroxy betulinic acid induces a mitochondria-derived ROS burst to triggerapoptotic cell death in hepatocellular carcinoma cells. J Exp Clin Cancer Res. 2016 Dec 8;35(1):192.
87. Liu Q, Xu C, Ji G, Liu H, Mo Y, Tollerud DJ, Gu A, Zhang Q. Sublethal effects of zinc oxide nanoparticles on male reproductive cells. Toxicol In Vitro. 2016 Sep;35:131-8.
88. Sun R, Cao M, Zhang J, Yang W, Wei H, Meng X, Yin L, Pu Y. Benzene Exposure Alters Expression of Enzymes Involved in Fatty Acid β-Oxidation in Male C3H/He Mice. Int J Environ Res Public Health. 2016 Oct 31;13(11). pii: E1068.
89. Li X, Xu L, Zhou W, Zhao Q, Wang Y. Chronic exposure to microcystin-LR affected mitochondrial DNA maintenance and caused pathologicalchanges of lung tissue in mice. Environ Pollut. 2016 Mar;210:48-56.
90. Yao C, Li G, Qian Y, Cai M, Yin H, Xiao L, Tang W, Guo F, Shi B. Protection of Pentoxifylline against Testis Injury Induced by Intermittent Hypobaric Hypoxia. Oxid Med Cell Longev. 2016; 2016:3406802.
91. Zhang J, Wang M, Li Z, Bi X, Song J, Weng S, Fu G. NADPH oxidase activation played a critical role in the oxidative stress process in stable coronary arterydisease. Am J Transl Res. 2016 Dec 15;8(12):5199-5210.
92. Dong Y, Cui P, Li Z, Zhang S. Aging asymmetry: systematic survey of changes in age-related biomarkers in the annual fishNothobranchius guentheri. Fish Physiol Biochem. 2016 Sep 10. [Epub ahead of print]
93. Zhang J, Shi R, Li H, Xiang Y, Xiao L, Hu M, Ma F, Ma CW, Huang Z. Antioxidant and neuroprotective effects of Dictyophora indusiata polysaccharide in Caenorhabditis elegans. J Ethnopharmacol. 2016 Nov 4;192:413-422.
94. Zou P, Xia Y, Chen W, Chen X, Ying S, Feng Z, Chen T, Ye Q, Wang Z, Qiu C, Yang S, Liang G. EF24 induces ROS-mediated apoptosis via targeting thioredoxin reductase 1 in gastric cancer cells. Oncotarget. 2016 Apr 5;7(14):18050-64.
95. Sun X, Jiao X, Ma Y, Liu Y, Zhang L, He Y, Chen Y. Trimethylamine N-oxide induces inflammation and endothelial dysfunction in human umbilical vein endothelial cells via activating ROS-TXNIP-NLRP3 inflammasome. Biochem Biophys Res Commun. 2016 Dec 2; 481(1-2):63-70.
96. Yuan XC, Wang P, Li HW, Wu QB, Zhang XY, Li BW, Xiu RJ. Effects of melatonin on spinal cord injury-induced oxidative damage in mice testis. Andrologia. 2016 Sep 5.doi: 10.1111/and.12692. [Epub ahead of print]
97. Cai C, Dai X, Zhu Y, Lian M, Xiao F, Dong F, Zhang Q, Huang Y, Zheng Q. A specific RAGE-binding peptide biopanning from phage display random peptide library that amelioratessymptoms in amyloid β peptide-mediated neuronal disorder. Appl Microbiol Biotechnol. 2016 Jan;100(2):825-35.
98. Zhang G, Zhu J, Zhou Y, Wei Y, Xi L, Qin H, Rao Z, Han M, Ma Y, Wu X. Hesperidin Alleviates Oxidative Stress and Upregulates the Multidrug Resistance Protein 2 in Isoniazid andRifampicin-Induced Liver Injury in Rats. J Biochem Mol Toxicol. 2016 Jul;30(7):342-9.
99. Ju L, Tong W, Qiu M, Shen W, Sun J, Chen Y, Li Z, Wang W, Tian J. Endurance exercise ameliorates low birthweight developed catch-up growth related metabolic dysfunctionsin a mouse model. Endocr J.

2016;63(3):275-85.

100.Chen W, Zou P, Zhao Z, Weng Q, Chen X, Ying S, Ye Q, Wang Z, Ji J, Liang G. Selective killing of gastric cancer cells by a small molecule via targeting TrxR1 and ROS-mediated ER stressactivation. Oncotarget. 2016 Mar 29;7(13):16593-609.

101.Zou P, Xia Y, Ji J, Chen W, Zhang J, Chen X, Rajamanickam V, Chen G, Wang Z, Chen L, Wang Y, Yang S, Liang G. Piperlongumine as a direct TrxR1 inhibitor with suppressive activity against gastric cancer. Cancer Lett. 2016 May 28;375(1):114-26.

102.Zheng H, Cui D, Quan X, Yang W, Li Y, Zhang L, Liu E. Lp-PLA2 silencing protects against ox-LDL-induced oxidative stress and cell apoptosis via Akt/mTORsignaling pathway in human THP1 macrophages. Biochem Biophys Res Commun. 2016 Sep 2; 477(4):1017-23.

103.Fang Y, Wang J, Xu L, Cao Y, Xu F, Yan L, Nie M, Yuan N, Zhang S, Zhao R, Wang H, Wu M, Zhang X, Wang J. Autophagy maintains ubiquitination-proteasomal degradation of Sirt3 to limit oxidative stress in K562leukemia cells. Oncotarget. 2016 Jun 14;7(24):35692-35702.

104.Chen W, Zou P, Zhao Z, Chen X, Fan X, Vinothkumar R, Cui R, Wu F, Zhang Q, Liang G, Ji J. Synergistic antitumor activity of rapamycin and EF24 via increasing ROS for the treatment of gastriccancer. Redox Biol. 2016 Dec;10:78-89.

105.Liu X, Fu Z, Wu Y, Hu X Jr, Zhu T Jr, Jin C Jr. Neuroprotective effect of hydrogen sulfide on acute cauda equina injury in rats. Spine J. 2016 Mar;16(3):402-7.

106.Wang Z, Ding J, Luo X, Zhang S, Yang G, Zhu Q, Liu D. Effect of Allopurinol on Myocardial Energy Metabolism in Chronic Heart Failure Rats After MyocardialInfarct. Int Heart J. 2016 Dec 2;57(6):753-759.

107.Wu F, Li Y, Song H, Zhang Y, Zhang Y, Jiang M, Wang F, Mu Q, Zhang W, Li L, Tang D. Preventive Effect of Dihydromyricetin against Cisplatin-Induced Nephrotoxicity In Vitro and In Vivo. Evid Based Complement Alternat Med. 2016;2016:7937385.

108.Yuan Z, Matias FB, Yi JE, Wu J. T-2 toxin-induced cytotoxicity and damage on TM3 Leydig cells. Comp Biochem Physiol C Toxicol Pharmacol. 2016 Mar-Apr;181-182:47-54.

109.He Z, Zhang L, Zhuo C, Jin F, Wang Y. Apoptosis inhibition effect of Dihydromyricetin against UVA-exposed human keratinocyte cell line. J Photochem Photobiol B. 2016 Aug;161:40-9.

110.Fang L, Su L, Sun X, Li X, Sun M, Karungo SK, Fang S, Chu J, Li S, Xin H. Expression of Vitis amurensis NAC26 in Arabidopsis enhances drought tolerance by modulating jasmonicacid synthesis. J Exp Bot. 2016 Apr;67(9):2829-45.

111.Yin Q, Ge H, Liao CC, Liu D, Zhang S, Pan YH. Antioxidant Defenses in the Brains of Bats during Hibernation. PLoS One. 2016 Mar 24;11(3):e0152135.

112.Ma L, Li J, Zhan Z, Chen L, Li D, Bai Q, Gao C, Li J, Zeng X, He Z, Wang S, Xiao Y, Chen W, Zhang A. Specific histone modification responds to arsenic-induced oxidative stress. Toxicol Appl Pharmacol. 2016 Jul 1;302:52-61.

113.Sun Y, Xiu C, Liu W, Tao Y, Wang J, Qu YI. Grape seed proantho cyanidin extract protects the retina against early diabetic injury by activating the Nrf2pathway. Exp Ther Med. 2016 Apr;11(4):1253-1258.

114.Lu J, Kan S, Zhao Y, Zhang W, Liu J. Novel naproxen/esomeprazole magnesium compound pellets based on acid-independent mechanism: invitro and in vivo evaluation. Drug Dev Ind Pharm. 2016 Sep; 42(9):1495-503.

115.Zhang G, Wang Q, Zhou Q, Wang R, Xu M, Wang H, Wang L, Wilcox CS, Liu R, Lai EY. Protective Effect of Tempol on Acute Kidney Injury Through PI3K/Akt/Nrf2 Signaling Pathway. Kidney Blood Press Res. 2016;41(2):129-38.

116.Duan WJ, Li YF, Liu FL, Deng J, Wu YP, Yuan WL, Tsoi B, Chen JL, Wang Q, Cai SH, Kurihara H, He RR. A SIRT3/AMPK/autophagy network orchestrates the protective effects of trans-resveratrol in stressedperitoneal macrophages and RAW 264.7 macrophages. Free Radic Biol Med. 2016 Jun;95:230-42.

117.Chen HM, Luo H, Zeng WB, Liu B, Huang JC, Liu M, Zeng YJ, Zheng Q, Li JQ, Sun XG, Zhou YC. Salvianolic acid B attenuates oxidized low-density lipoprotein-induced endothelial cell apoptosis throughinhibition of oxidative stress, p53, and caspase-3 pathways. Chin J Integr Med. 2017 Jan 24.doi: 10.1007/s11655-016-2645-4. [Epub ahead of print]

118.Huang W, Guo HL, Deng X, Zhu TT, Xiong JF, Xu YH, Xu Y. Short-Chain Fatty Acids Inhibit Oxidative Stress and Inflammation in Mesangial Cells Induced by HighGlucose and Lipopolysaccharide. Exp Clin Endocrinol Diabetes. 2017 Jan 3.doi: 10.1055/s-0042-121493. [Epub ahead of print]

119.Liu P, Zou D, Chen K, Zhou Q, Gao Y, Huang Y, Zhu J, Zhang Q, Mi M. Erratum to: Dihydromyricetin Improves Hypobaric Hypoxia-Induced Memory Impairment via Modulation ofSIRT3 Signaling. Mol Neurobiol. 2017 Jan 16.doi: 10.1007/s12035-017-0399-4. [Epub ahead of print]

120.Hu W, Wang H, Liu Z, Liu Y, Wang R, Luo X, Huang Y. Neuroprotective effects of lycopene in spinal cord injury in rats via antioxidative and anti-apoptoticpathway. Neurosci Lett. 2017 Feb 3;642:107-112.

121.Wang H, Wang D, Yang L, Wang Y, Jia J, Na D, Chen H, Luo Y, Liu C. Compact bone-derived mesenchymal stem cells attenuate nonalcoholic steatohepatitis in a mouse model bymodulation of CD4 cells differentiation. Int Immunopharmacol. 2017 Jan;42:67-73.

122.Qu XY, Tao LN, Zhang SX, Sun JM, Niu JQ, Ding YH, Song YQ. The role of Ntcp, Oatp2, Bsep and Mrp2 in liver injury induced by Dioscorea bulbifera L.and Diosbulbin B in mice. Environ Toxicol Pharmacol. 2017 Apr;51:16-22.

123.Ju L, Tong W, Qiu M, Shen W, Sun J, Zheng S, Chen Y, Liu W, Tian J. Antioxidant MMCC ameliorates catch-up growth related metabolic dysfunction. Oncotarget. 2017 Oct 23;8(59):99931-99939.

124.Chen Y, Zhou CF, Xiao F, Huang HL, Zhang P, Gu HF, Tang XQ. Inhibition of ALDH2 protects PC12 cells against formaldehyde-induced cytotoxicity: involving the protection of hydrogen sulphide. Clin Exp Pharmacol Physiol. 2017 May;44(5):595-601.

125.Gong W, Li J, Chen Z, Huang J, Chen Q, Cai W, Liu P, Huang H. Polydatin promotes Nrf2-ARE anti-oxidative pathway through activating CKIP-1 to resist HG-induced up-regulation of FN and ICAM-1 in GMCs and diabetic mice kidneys. Free Radic Biol Med. 2017 May;106:393-405.

126.Sun R, Zhang J, Wei H, Meng X, Ding Q, Sun F, Cao M, Yin L, Pu Y. Acetyl-l-carnitine partially prevents benzene-induced hematotoxicity and oxidative stress in C3H/He mice. Environ Toxicol Pharmacol. 2017 Apr;51:108-113.

127.Xu W, Li F, Xu Z, Sun B, Cao J, Liu Y. Tert-butylhydroquinone protects PC12 cells against ferrous sulfate-induced oxidative and inflammatory injury via the Nrf2/ARE pathway. Chem Biol Interact. 2017 Aug 1;273:28-36.

128.Dong Y, Cui P, Li Z, Zhang S. Aging asymmetry: systematic survey of changes in age-related biomarkers inthe annual fishNothobranchius guentheri. Fish Physiol Biochem. 2017 Apr;43(2):309-319.

129.Zhang Y, Zhao L, Wu Z, Chen X, Ma T. Galantamine alleviates senescence of U87 cells induced by beta-amyloid through decreasingROS production. Neurosci Lett. 2017 Jul 13;653:183-188.

130.Peng J, Deng X, Huang W, Yu JH, Wang JX, Wang JP, Yang SB, Liu X, Wang L, Zhang Y, Zhou XY, Yang H, He YZ, Xu FY. Irisin protects against neuronal injury induced by oxygen-glucose deprivation in part depends on the inhibition of ROS-NLRP3 inflammatory signaling pathway. Mol Immunol. 2017 Nov;91:185-194.

131.Wang H, Wang D, Yang L, Wang Y, Jia J, Na D, Chen H, Luo Y, Liu C. Compact bone-derived mesenchymal stem cells attenuate nonalcoholic steatohepatitis ina mouse model by modulation of CD4 cells differentiation. Int Immunopharmacol. 2017 Jan;42:67-73.

132.Fan L, Luo J, Li X, Chen M, Shu W, Qu X. Activation of Na+/H+ exchanger other than formation of transmembrane pore underlies the cytotoxicity of nematocyst venom from Chrysaora helvola Brandt jellyfish. Toxicon. 2017 Jul;133:162-168.

133.Wang D, Wang Y, Long W, Niu M, Zhao Z, Teng X, Zhu X, Zhu J, Hao Y, Wang Y, Liu Y, Jiang L, Wang Y, Wan J. SGD1, a key enzyme in tocopherol biosynthesis, is essential for plant development and coldtolerance in rice. Plant Sci. 2017 Jul;260:90-100.

134.Li XJ, Liang L, Shi HX, Sun XP, Wang J, Zhang LS. Neuroprotective effects of curdione against focal cerebral ischemia reperfusion injury in rats. Neuropsychiatr Dis Treat. 2017 Jun 30;13:1733-1740.

135.Wang X, Du X, Zhou Y, Wang S, Su F, Zhang S. Intermittent food restriction initiated late in life prolongs lifespan and retards the onset of age-related markers inthe annual fish Nothobranchius guentheri. Biogerontology. 2017 Jun;18(3):383-396.

136.Wang Z, Xiong L, Wang G, Wan W, Zhong C, Zu H. Insulin-like growth factor-1 protects SH-SY5Y cells against β-amyloid-induced apoptosis via the PI3K/Akt-Nrf2 pathway. Exp Gerontol. 2017 Jan;87(Pt A):23-32.

137.Hu W, Wang H, Liu Z, Liu Y, Wang R, Luo X, Huang Y. Neuroprotective effects of lycopene in spinal cord injury in rats via antioxidative and

anti-apoptotic pathway. Neurosci Lett. 2017 Mar 6;642:107-112.

138. Rong J, Shan C, Liu S, Zheng H, Liu C, Liu M, Jin F, Wang L. Skin resistance to UVB-induced oxidative stress and hyperpigmentation by the topical use of Lactobacillus helveticus NS8-fermented milk supernatant. J Appl Microbiol. 2017 Aug;123(2):511-523.

139. Yuan XC, Wang P, Li HW, Wu QB, Zhang XY, Li BW, Xiu RJ. Effects of melatonin on spinal cord injury-induced oxidative damage in mice testis. Andrologia. 2017 Sep;49(7).

140. Liang C, Wang X, Hu J, Lian X, Zhu T, Zhang H, Gu N, Li J. PTPRO Promotes Oxidized Low-Density Lipoprotein Induced Oxidative Stress and Cell Apoptosis through Toll-Like Receptor 4/Nuclear Factor κB Pathway. Cell Physiol Biochem. 2017;42(2):495-505.

141. Zhao J, Fu B, Peng W, Mao T, Wu H, Zhang Y. Melatonin protect the development of preimplantation mouse embryos from sodium fluoride-induced oxidative injury. Environ Toxicol Pharmacol. 2017 Sep;54:133-141.

142. Qin SB, Peng DY, Shi Y, Ke ZP. MiR-182-5p Inhibited Oxidative Stress and Apoptosis Triggered by Oxidized Low-DensityLipoprotein via Targeting Toll-Like Receptor 4. J Cell Physiol. 2017 Dec 11.

143. Chen J, Liu J, Wang Y, Hu X, Zhou F, Hu Y, Yuan Y, Xu Y. Wogonin mitigates nonalcoholic fatty liver disease via enhancing PPARα/AdipoR2, in vivo and in vitro. Biomed Pharmacother. 2017 Jul;91:621-631.

144. Liang R, Zhang Z, Lin S. Effects of pulsed electric field on intracellular antioxidant activity and antioxidant enzymeregulating capacities of pine nut (Pinus koraiensis) peptide QDHCH in HepG2 cells. Food Chem. 2017 Dec 15;237:793-802.

145. Xue R, Yang J, Wu J, Meng Q, Hao J. Coenzyme Q10 inhibits the activation of pancreatic stellate cells through PI3K/AKT/mTORsignaling pathway. Oncotarget. 2017 Sep 23;8(54):92300-92311.

146. Chen X, Dai X, Zou P, Chen W, Rajamanickam V, Feng C, Zhuge W, Qiu C, Ye Q, Zhang X, Liang G. Curcuminoid EF24 enhances the anti-tumour activity of Akt inhibitor MK-2206 through ROS-mediated endoplasmic reticulum stress and mitochondrial dysfunction in gastric cancer. Br J Pharmacol. 2017 May;174(10):1131-1146.

147. Kong G, Huang Z, Ji W, Wang X, Liu J, Wu X, Huang Z, Li R, Zhu Q. The Ketone Metabolite β-Hydroxybutyrate Attenuates Oxidative Stress in Spinal Cord Injury by Suppression of Class I Histone Deacetylases. J Neurotrauma. 2017 Sep 15;34(18):2645-2655.

148. Chen HM, Luo H, Zeng WB, Liu B, Huang JC, Liu M, Zeng YJ, Zheng Q, Li JQ, Sun XG, Zhou YC. Salvianolic acid B attenuates oxidized low-density lipoprotein-induced endothelial cell apoptosis through inhibition of oxidative stress, p53,and caspase-3 pathways. Chin J Integr Med. 2017 Jan 24.

149. Wang Z, Hou L, Huang L, Guo J, Zhou X. Exenatide improves liver mitochondrial dysfunction and insulin resistance by reducing oxidativestress in high fat diet-induced obese mice. Biochem Biophys Res Commun. 2017 Apr 22;486(1):116-123.

150. Dong X, Niu Y, Ding Y, Wang Y, Zhao J, Leng W, Qin L. Formulation and Drug Loading Features of Nano-Erythrocytes. Nanoscale Res Lett. 2017 Dec;12(1):202.

151. Chen X, Dong Q, Chen Y, Zhang Z, Huang C, Zhu Y, Zhang Y. Effects of Dechlorane Plus exposure on axonal growth, musculature and motor behavior in embryo-larval zebrafish. Environ Pollut. 2017 May;224:7-15.

152. Ma T, Zhang Y, Zhang C, Luo JG, Kong LY. Downregulation of TIGAR sensitizes the antitumor effect of physapubenolide through increasingintracellular ROS levels to trigger apoptosis and autophagosome formation in human breast carcinoma cells. Biochem Pharmacol. 2017 Nov 1;143:90-106.

153. Cheng Y, Wang X, Wang B, Zhou H, Dang S, Shi Y, Hao L, Luo Q, Jin M, Zhou Q, Zhang Y. Aging-associated oxidative stress inhibits liver progenitor cell activation in mice. Aging (Albany NY). 2017 Apr 29;9(5):1359-1374.

154. Jiao YH, Dou M, Wang G, Li HY, Liu JS, Yang X, Yang WD. Exposure of okadaic acid alters the angiogenesis in developing chick embryos. Toxicon. 2017 Jul;133:74-81.

155. Dai X, Guo G, Zou P, Cui R, Chen W, Chen X, Yin C, He W, Vinothkumar R, Yang F, Zhang X, Liang G. (S)-crizotinib induces apoptosis in human non-small cell lung cancer cells by activating ROSindependent of MTH1. J Exp Clin Cancer Res. 2017 Sep 7;36(1):120.

156. Wang J, Huang J, Wang L, Chen C, Yang D, Jin M, Bai C, Song Y. Urban particulate matter triggers lung inflammation via the ROS-MAPK-NF-κB signaling pathway. J Thorac Dis. 2017 Nov;9(11):4398-4412.

157. Wang L, Ma R, Guo Y, Sun J, Liu H, Zhu R, Liu C, Li J, Li L, Chen B, Sun L, Tang J, Zhao D, Mo F, Niu J, Jiang G, Fu M, Brömme D, Zhang D, Gao S. AntioxidantEffect of Fructus LigustriLucidi Aqueous Extract in Ovariectomized Rats Is Mediated through Nox4-ROS-NF-κB Pathway. Front Pharmacol. 2017 May 22;8:266.

158. Wu J, Chen Y, Yu S, Li L, Zhao X, Li Q, Zhao J, Zhao Y. Neuroprotective effects of sulfiredoxin-1 during cerebral ischemia/reperfusion oxidative stressinjury in rats. Brain Res Bull. 2017 Jun;132:99-108.

159. Shi Q, Liu X, Wang N, Zheng X, Ran J, Liu Z, Fu J, Zheng J. 1400W ameliorates acute hypobaric hypoxia/reoxygenation-induced cognitive deficits by suppressing the induction of inducible nitric oxide synthase in rat cerebral cortex microglia. Behav Brain Res. 2017 Feb 15;319:188-199.

160. Xu W, Li F, Liu Z, Xu Z, Sun B, Cao J, Liu Y. MicroRNA-27b inhibition promotes Nrf2/ARE pathway activation and alleviates intracerebralhemorrhage-induced brain injury. Oncotarget. 2017 Aug 7;8(41):70669-70684.

161. Chen X, Dong Q, Chen Y, Zhang Z, Huang C, Zhu Y, Zhang Y. Effects of Dechlorane Plus exposure on axonal growth, musculature and motor behavior in embryo-larval zebrafish. Environ Pollut. 2017 May;224:7-15.

162. Liu N, Chang Y, Feng Y, Cheng Y, Sun X, Jian H, Feng Y, Li X, Zhang H. {101}-{001} Surface Heterojunction-Enhanced Antibacterial Activity of Titanium DioxideNanocrystals Under Sunlight Irradiation. ACS Appl Mater Interfaces. 2017 Feb 22;9(7):5907-5915.

163. Chen X, Wo F, Jin Y, Tan J, Lai Y, Wu J. Drug-Porous Silicon Dual Luminescent System for Monitoring and Inhibition of Wound Infection. ACS Nano. 2017 Aug 22;11(8):7938-7949.

164. Wang W, Wang R, Zhang Q, Mor G, Zhang H. Benzo(a)pyren-7,8-dihydrodiol-9,10-epoxide induces human trophoblast Swan 71 celldysfunctions due to cell apoptosis through disorder of mitochondrial fission/fusion. Environ Pollut. 2018 Feb;233:820-832.

165. Zhong J, Yu H, Huang C, Zhong Q, Chen Y, Xie J, Zhou Z, Xu J, Wang H. Inhibition of phosphodiesterase 4 by FCPR16 protects SH-SY5Y cells against MPP+-induceddecline of mitochondrial membrane potential and oxidative stress. Redox Biol. 2018 Feb 14;16:47-58.

166. Ling H, Zhu Z, Yang J, He J, Yang S, Wu D, Feng S, Liao D. Dihydromyricetin improves type 2 diabetes-induced cognitive impairment via suppressingoxidative stress and enhancing brain-derived neurotrophic factor-mediated neuroprotection in mice. Acta Biochim Biophys Sin (Shanghai). 2018 Mar 1;50(3):298-306.

167. Zeng X, Yang J, Hu O, Huang J, Ran L, Chen M, Zhang Y, Zhou X, Zhu J, Zhang Q, Yi L, Mi M. Dihydromyricetin Ameliorates Nonalcoholic Fatty Liver Disease by Improving MitochondrialRespiratory Capacity and Redox Homeostasis Through Modulation of SIRT3 Signaling. Antioxid Redox Signal. 2018 Feb 21. doi: 10.1089/ars.2017.7172.

注：更多使用本产品的文献请参考产品网页。

Version 2019.11.19
