# Supplementary material for: Combination decoction of Astragalus mongholicus and Salvia miltiorrhiza mitigates pressure-overload cardiac dysfunction by inhibiting multiple ferroptosis pathways
Source: Front Pharmacol. 2024 Dec 16;15:1447546. doi: 10.3389/fphar.2024.1447546 (PMC11683366; doi:10.3389/fphar.2024.1447546)
Supplement: Supplementary file 7 [file Image1.PDF]

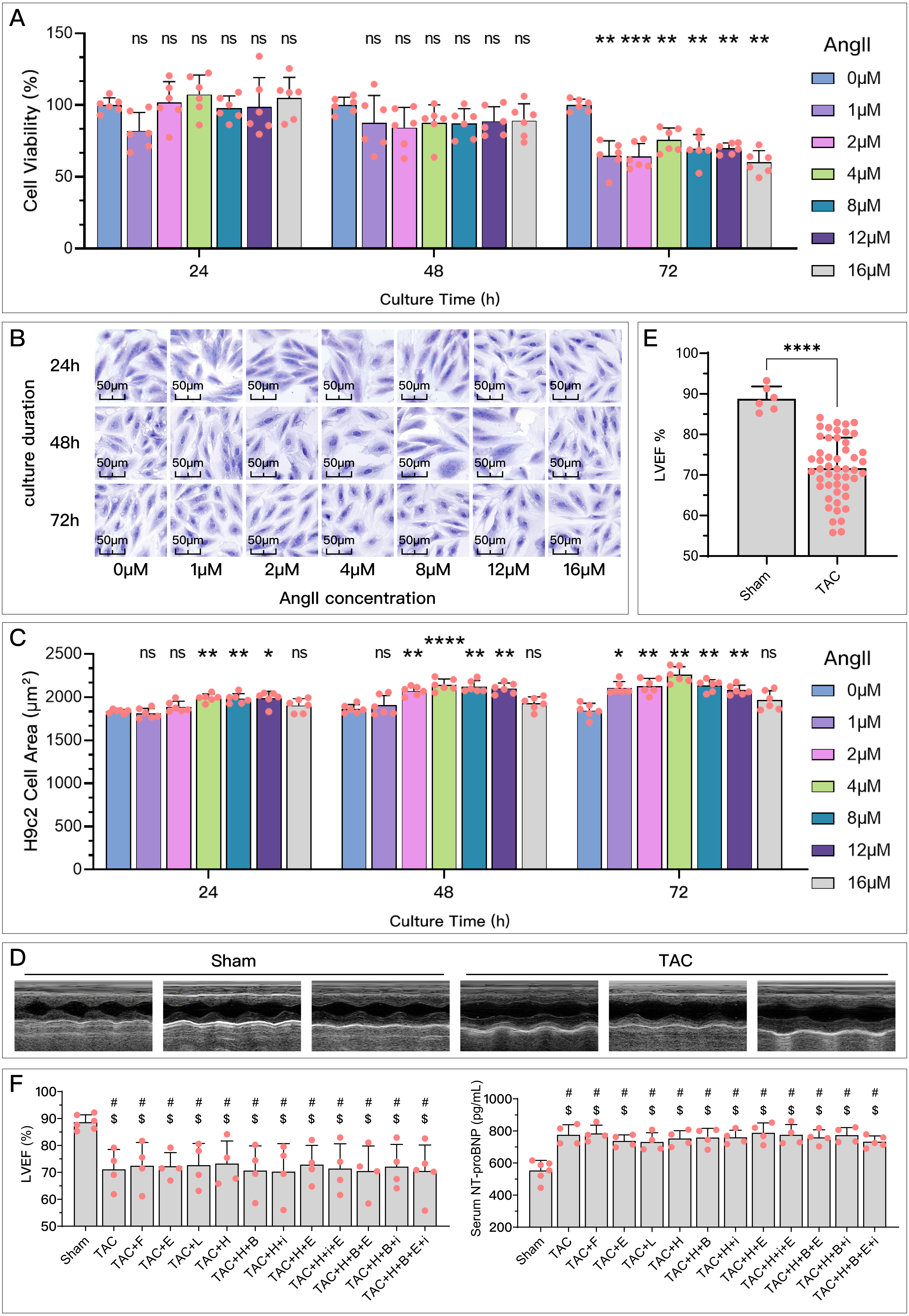
A
Cell Viability (%)
ns ns ns ns ns ns
ns ns ns ns ns ns
** *** ** ** ** **
24
48
72
Culture Time (h)
AngII
0μM
1μM
2μM
4μM
8μM
12μM
16μM
B
culture duration
24h
48h
72h
50μm
0μM 1μM 2μM 4μM 8μM 12μM 16μM
AngII concentration
E
LVEF %
****
Sham
TAC
C
H9c2 Cell Area (μm²)
ns ns ** ** * ns
ns ** **** ** ** ns
* ** ** ** ** ns
Culture Time (h)
D
Sham
TAC
F
LVEF (%)
Serum NT-proBNP (pg/mL)
#
$
Sham
TAC
TAC+F
TAC+E
TAC+L
TAC+H
TAC+H+B
TAC+H+i
TAC+H+E
TAC+H+i+E
TAC+H+B+E
TAC+H+B+i
TAC+H+B+E+i

Supplementary Fig. 1. AngII-induced H9c2 failure and cardiac function decline in TAC model rats. (A) Relative cell viability of H9c2 cells cultured with different concentrations of AngII for various durations, as determined by cell counting kit-8; (B) Microscopic images (400x) of H9c2 cells stained with crystal violet after culture with different concentrations of AngII for various times; (C) Quantitative analysis of the average cell area for each group based on crystal violet-stained microscopic images; (D) Parasternal long-axis M-mode echocardiographic images of the left ventricle in rats 8 weeks after TAC surgery (aortic arch narrowed to 1 mm), comparing the Sham and TAC groups; (E) Quantitative comparison of LVEF differences between the Sham and TAC groups based on images from (D); (F) Comparison of LVEF and serum NT-proBNP levels among 13 groups, after stratifying the TAC group by LVEF prior to drug intervention and then randomly dividing into 12 groups. pns≥0.05, p*＜0.05, p**＜0.01, p***＜0.001, p****＜0.0001 vs. the 0μM AngII group within each group; p#＜0.05 vs. Sham group; p$≥0.05 vs. all other groups except Sham. ● represents an individual sample data point. AngII, Angiotensin II; TAC, Transverse Aortic Constriction; LVEF, Left Ventricular Ejection Fraction; NT-proBNP, N-terminal pro B-type Natriuretic Peptide.
